# Exploring the Potential Mechanism of Chuanxiong Rhizoma Treatment for Migraine Based on Systems Pharmacology

**DOI:** 10.1155/2021/2809004

**Published:** 2021-12-28

**Authors:** Xianhua Wen, Yuncheng Gu, Beili Chen, Feipeng Gong, Wenting Wu, Hengli Tong, Qianfeng Gong, Songhong Yang, Lingyun Zhong, Xuping Liu

**Affiliations:** ^1^Jiangxi University of Chinese Medicine, Nanchang, China; ^2^Jiangxi Provincial People's Hospital Affiliated to Nanchang University, Nanchang, China; ^3^Tiantai County Food and Drug Testing Center, Taizhou, China; ^4^Jiangxi Provincial Institute for Drug Control, NMPA Laboratory of Quality Evaluation of Traditional Chinese Patent Medicine, Jiangxi Province Engineering Research Center of Drug and Medical Device Quality, Nanchang, China

## Abstract

Migraine is a disease whose aetiology and mechanism are not yet clear. Chuanxiong Rhizoma (CR) is employed in traditional Chinese medicine (TCM) to treat various disorders. CR is effective for migraine, but its active compounds, drug targets, and exact molecular mechanism remain unclear. In this study, we used the method of systems pharmacology to address the above issues. We first established the drug-compound-target-disease (D-C-T-D) network and protein-protein interaction (PPI) network related to the treatment of migraine with CR and then established gene ontology (GO) and Kyoto Encyclopedia of Genes and Genomes (KEGG) pathway enrichment analyses. The results suggest that the treatment process may be related to the regulation of inflammation and neural activity. The docking results also revealed that PTGS2 and TRPV1 could directly bind to the active compounds that could regulate them. In addition, we found that CR affected 11 targets that were more highly expressed in the liver or heart but were the lowest in the whole brain. It also expounds the description of CR channel tropism in TCM theory from these angles. These findings not only indicate that CR can be developed as a potential effective drug for the treatment of migraine but also demonstrate the application of systems pharmacology in the discovery of herbal-based disease therapies.

## 1. Introduction

Currently, the medical community considers migraine to be a chronic neurobiological disorder, which is characterized by a long period of unilateral headache (from 4 hours up to 72 hours), recurrent attacks, and other features such as nausea, or photo or sound phobias [1]. In addition, the prevalence of the disease is much higher in women than in men [2]. From the point of view of modern medicine, the pathophysiology of migraine has not been fully elucidated, and its pathogenesis can be divided into vascular theory, neuron theory, inflammatory mediator theory and “microbiota-gut-brain axis” theory [3–6].

Traditional Chinese medicine has been used to treat migraine for more than 2,000 years. Among historical figures, Cao Cao, who was an emperor in the Three Kingdoms period, suffered from migraine symptoms recognized by later doctors. Migraine belongs to the traditional Chinese medicine category “head wind” “head wind,” and differentiated by “external feeling” and “internal injury,” commonly two big categories. Treatment is frequently with dispelling wind, liver, phlegm, or line stasis [7]. Compared with Western medicine, Chinese medicine has many different antimigraine compounds and targets [8]. Therefore, Chinese medicine has accumulated rich experience in the treatment of migraine [9–11]. Chuanxiong Rhizoma (CR), which is derived from the root of *Ligusticum chuanxiong* Hort, is a traditional Chinese herbal medicine. It belongs to *Ligusticum L.* in the Umbelliferous family and is widely found in Sichuan, Yunnan, Guizhou, Guangxi, and other provinces in China. CR has been recorded in “Shen Nong's Herbal Classic” and “Compendium of Materia Medica” and is described as “pungent and warm.” In the Chinese Pharmacopoeia, CR can be used to promote the flow of qi and blood circulation, wind-expelling, and pain alleviation. It is often used to treat migraine, rheumatism, and irregular menstruation.

Although CR can effectively treat migraine, its active compounds, drug targets and exact molecular mechanisms remain unclear. It is gratifying that in recent years, systems pharmacology has been used to study the therapeutic effects and therapeutic targets of active compounds contained in traditional Chinese medicine and biology. Its concept of “network targets, multicompounds” is the most suitable tool for exploring the therapeutic effects of herbal medicine at the molecular level [12, 13]. This novel research model can be used to explain and promote the development of evidence-based medicine and new drug discovery based on herbs. Using a network-based approach, systems pharmacology can systematically determine the actions and mechanisms of drugs used to treat complex diseases at the molecular, cellular, tissue, and biological levels. This strategy has been widely used in the study of *Atractylodes macrocephala* Koidz., Radix *Puerariae*, *Zanthoxylum bungeanum* Maxim., and *Citri Reticulatae* Pericarpium [14–17].

**Figure 1:**
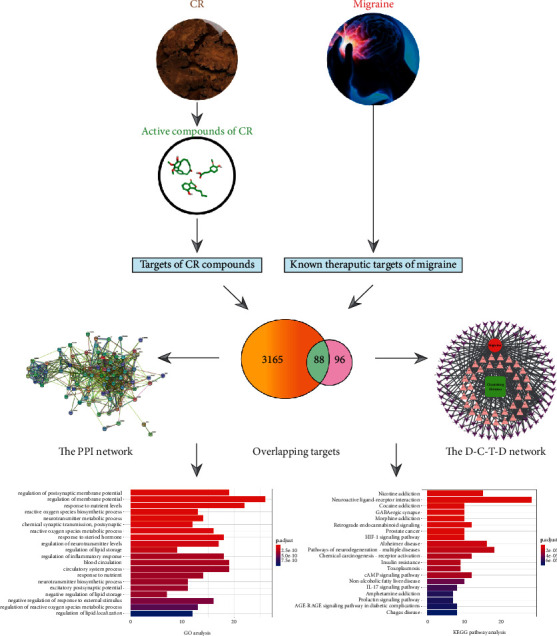
Flowchart of a systems pharmacology-based strategy to investigate the pharmacologic mechanisms of *Chuanxiong* Rhizoma for the treatment of migraine.

In this study, we used systems pharmacology to explore whether CR has a therapeutic effect on migraine and to elucidate its potential mechanism of action. The flowchart of this study is shown in Figure 1.

## 2. Materials and Methods

### 2.1. Plant Materials and Sample Preparation

Pure distilled water was purchased from Watsons (Hong Kong, China). Formic acid was purchased from Sinopharm Chemical Reagent Co., Ltd. (Shanghai, China). HPLC grade acetonitrile and methanol were obtained from Fisher Scientific (Fair Lawn, NJ, USA). The raw material of CR was purchased from herbal medicine markets located in Zhangshu City, Jiangxi Province. These samples were identified by professor Qianfeng Gong, Jiangxi University of Traditional Chinese Medicine. The voucher specimens were deposited at the herbarium of the Jiangxi University of Traditional Chinese Medicine.

Accurately weighed powder (1.0 g) was placed into a 50 mL flask, and each sample was extracted with 30 mL of ethanol in an ultrasonic water bath at room temperature for 1 h. The extraction solutions of the sample were centrifuged for 15 min at 12000 rpm. Finally, 2 *μ*L of the CR filtered supernatants were injected for UHPLC-QTOF-MS/MS analyses.

For UHPLC-TOF-MS/MS analysis, the UHPLC analyses were conducted on a Shimadzu system (Kyoto, Japan), combined with a LC-3AD solvent delivery system, a SIL-30ACXR auto-sampler, a CTO-30AC column oven, a DGU-20A3 degasser, and a CBM-20A controller. The analytical column was a Welch UHPLC C18 (100 mm × 2.1 mm, 1.8 *μ*m). The column oven temperature was set at 40°C. The mobile phases consisted of water containing 0.1% formic acid (solvent A) and acetonitrile (solvent B). The flow rate was set at 0.3 mL/min. The binary gradient was applied with linear interpolation as follows: 0.01 min, 2% B; 3 min, 8% B; 11 min, 16% B; 19 min, 18% B; 26 min, 30% B; 32 min; 60% B; 42 min, 95% B; 42.1 min, 2% B; 45 min, 5% B.

The QTOF-MS/MS detection was operated on a Triple TOF^TM^ 5600+ system (AB SCIEX, Foster City, CA, USA). The electrospray ionization was applied in both the negative and positive mode with the following parameters: ion spray voltage, −4500 V/5500V; ion source temperature, 550°C; curtain gas, 35 psi; nebulizer gas (GS 1), 55 psi; heater gas (GS 2), 55 psi; and declustering potential (DP), 100 V. The mass ranges were set at *m*/*z* 50–1250 Da for the TOF-MS scan and 50–1250 Da for the TOF-MS/MS experiments. In the IDA-MS/MS experiment, the collision energy (CE) was set at 35 eV, and the collision energy spread (CES) was (±) 15 eV for the QTOF-MS/MS detection. The MS/MS data was analyzed by PeakView® 1.2 software (AB SCIEX, Foster City, CA, USA).

### 2.2. Collection of the Candidate Compounds of CR

We searched six databases, including the Traditional Chinese Medicine Systems Pharmacology Database and Analysis Platform (TCMSP; http://lsp.nwu.edu.cn/tcmsp.php), the Chinese Academy of Sciences Chemistry Database (http://www.organchem.csdb.cn), the TCM Database@Taiwan (http://tcm.cmu.edu.tw), the Integrative Pharmacology-based Research Platform of Traditional Chinese Medicine (TCMIP; http://www.tcmip.cn), Symptom Mapping (SYMMAP, http://www.symmap.org), and the Traditional Chinese Medicine Integrated Database (TCMID; http://www.megabionet.org/tcmid) to collect the candidate compounds of CR as comprehensively as possible. In addition, we also reviewed the relevant literature to gather a more comprehensive collection of CR compounds. Finally, we summarized the compounds and established a database of CR for this study.

### 2.3. Screening of Active Compounds

Since the compounds of traditional Chinese medicine are very complex and in order to better select compounds with high potential to become drugs for subsequent targeted research, we screened the compounds in the database that we had established previously. In this study, two parameters, “oral bioavailability” (OB) and “drug likeness” (DL), were used to perform the screening process.

OB is defined as the percentage of a drug capable of invading a primitive culture and that is not modified enough to enter the human circulatory system [18, 19]. OB is usually regarded as an objective and important index to evaluate the internal quality of drugs [20]. The OB of compounds is proportional to their likelihood for clinical use.

DL refers to the similarity between compounds and known drugs [21, 22]. Compounds with DL properties may not necessarily already be drugs but have the potential to be drugs. Such compounds usually include drug-like small molecules or drug-like compounds. Here, we use the classical Tanimoto coefficient to calculate the DL index of the compounds contained in CR; the formula is as follows:

(1) $$\begin{array}{c} T\left(\alpha ,\beta \right)=\frac{\alpha \times \beta }{\alpha^{2}+\beta^{2}-\alpha \times \beta }. \end{array}$$

*α* represents the molecular properties of CR compounds based on computing from Dragon software (http://www.talete.mi.it/products/dragon_description.htm), and *β* for all of the drugs comes from the average molecular properties in the DrugBank database (http://www.drugbank.ca) [23].

Most of the compounds in traditional Chinese medicine have weak pharmacological properties, so they are difficult to combine with specific targets on cells significantly. Therefore, molecules with OB ≥ 15% or DL ≥ 0.10 are generally considered to have stronger pharmacological effects in this kind of study, and, therefore, researchers select these as the active compounds for focused analysis [24–26]. Therefore, in this study, we applied the same principle to further screen the candidate compounds in order to ultimately obtain the active compounds.

### 2.4. Prediction of the Relevant Targets of CR Active Compounds

Traditional Chinese medicine is characterized by multicompounds and multitarget modes of action. Therefore, it is particularly important to predict targets that can be affected by active compounds. Based on the experience accumulated in our previous studies, we ultimately chose the ligand-based screening method for the prediction of this part [14].

### 2.5. Acquisition of Targets for Migraine

The migraine-related targets were from the Therapeutic Target Database (TTD), the Human Phenotype Ontology (HPO), the Integrative Pharmacology-Based Research Platform of Traditional Chinese Medicine (TCMIP), Online Mendelian Inheritance in Man (OMIM), and GeneCards (the Human Gene Database (http://www.genecards.org/) comprehensive collection in five databases).

### 2.6. Drug-Compound-Target-Disease (D-C-T-D) Network Construction

The intersection of the predicted drug-related and disease-related targets was chosen to obtain the Venn diagram of the overlapping targets. Next, complex information networks based on the interactions of drugs (CR), active compounds, overlapping targets, and disease (migraine) were constructed. Finally, Cytoscape 3.7.1 software was used to visualize and analyze the drug-compound-target-disease (D-C-T-D) network.

### 2.7. Protein-Protein Interaction (PPI) Network Construction

The STRING online database (https://string-db.org/) was used to obtain PPI data of the previous overlapping targets in the network. The object was selected as “*Homo sapiens*,” and the others were kept as defaults. Finally, the PPI relationship network was established by Cytoscape 3.7.1 software, and topology analysis was carried out. In addition, the BIOGPS database (https://biogps.org) was used for analysis to identify the high expression of the targets in some major organs.

### 2.8. Enrichment of Gene Ontology (GO) and Kyoto Encyclopedia of Genes and Genomes Pathways (KEGG)

GO analysis and KEGG pathway enrichment were performed using Bioconductor (R) V3.8 bioinformatics software (http://bioconductor.org/). GO items (*p.adjust* ≤ 0.05) were collected for functional annotation clustering. The KEGG database was used for pathway enrichment analysis to verify statistically significant gene function categories (*p.adjust* ≤ 0.05).

### 2.9. Computational Validation of Compound-Target Interactions

We hope to determine the interaction between the active compounds and their targets and explore their binding patterns. Therefore, three active compounds and two targets were selected, and a total of four compound-target interactions were used to verify molecular docking. Docking studies were conducted using AutoDock Vina [27], and input files required by AutoDock programs were prepared using AutoDockTools [28]. The size of the grid box in AutoDock Vina remained 40 × 40 × 40 for *X*, *Y*, and *Z*, and the energy range remained the default setting. The X-ray crystal structures of PTGS2 and TRPV1 were obtained from the RCSB protein database (PDB) (http://www.rcsb.org). The PDB entry codes for these proteins were 5F19 and 6L93, respectively. The program makes calculations based on the different binding energies of each ligand and yields nine possible conformations. We then selected the best model based on binding affinity and molecular contact. The calculation of molecular contact was carried out by the program CONTACT provided in the CCP4 package [29]. The docking complex was analyzed and plotted using PyMol (http://www.pymol.org).

## 3. Results

### 3.1. Chemical Structure Identification of CR

**Figure 2:**
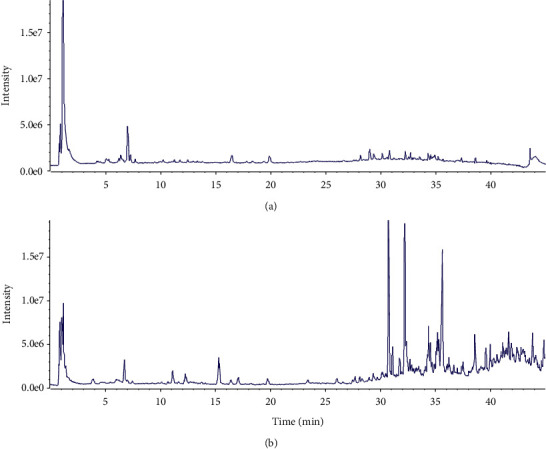
The total ion chromatograms (TICs) from the analysis of a crude extract of CR (a) in negative mode and (b) in positive mode.

An effective and systematic UHPLC-QTOF-MS/MS method was established to screen and identify the constituents of CR. As a result, a total of 33 compounds were efficiently found and identified from an extract of CR. A representative total ion chromatographic (TIC) is shown in Figure 2. The identified 33 compounds are exhibited in Table 1.

### 3.2. Collection the Candidate Compounds of CR

By searching six databases combined with the literature search, we ultimately established a database of CR compounds for the present study (Supplementary Table S1). A total of 248 candidate compounds were included.

### 3.3. Screening of Active Compounds

In order to screen for active compounds with high potential for CR, we used two classical absorption, distribution, metabolism, and excretion (ADME) parameters, OB and DL, to screen our subdatabase. At the same time, we noted that although some compounds did not conform to the above rules, they may also have therapeutic effects on the human body. Therefore, for this reason but also to be able to study this issue more fully, we nevertheless treated them as active compounds, even though they did not conform to the screening rules. For example, although ferulic acid does not conform to the above rules, we attach great importance to it because it is the standard compound for CR in the Chinese pharmacopoeia and has strong biological activity [30]. Studies have shown that it can regulate various inflammatory responses by inhibiting the production of interleukin 8 (IL-8), thus producing anti-inflammatory effects [31]. It shows strong antioxidant activity by scavenging free radicals [32, 33]. In addition, it inhibits vascular smooth muscle cell proliferation induced by angiotensin II [34]. In summary, through this part of the work, we ultimately screened and obtained 38 active compounds of CR that we considered, as shown in Table 2.

### 3.4. Prediction of the Relevant Targets of CR Active Compounds

The relevant target information of CR active compounds was collected from the above six databases. After the UniProt database was converted into standard names and redundant items were deleted, 38 active compounds and 184 targets relevant for CR were obtained (Supplementary Table S2).

### 3.5. Acquisition of Targets for Migraine

We collected targets related to migraine from the above five disease databases. After removing the redundancy, a total of 3253 known targets for migraine treatment were collected (Supplementary Table S3).

### 3.6. Analyses of Drug-Compound-Target-Disease (D-C-T-D) Network

**Figure 3:**
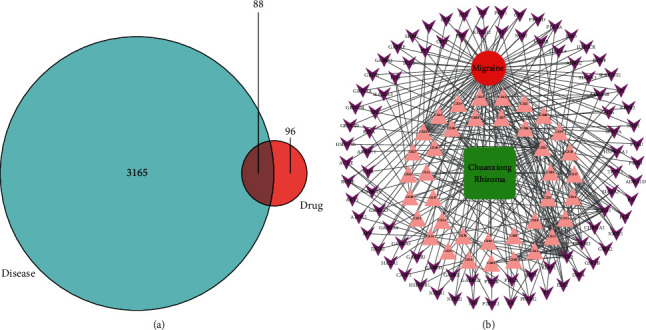
(a) Eighty-eight overlapping targets between the disease and drug. (b) D-C-T-D network. The green square node represents the drug (CR), the red round node represents the disease (migraine), 38 pink triangle nodes represent the active compounds in CR, and 88 purple arrow nodes represent the overlapping targets between CR and migraine.

Figure 3(a) shows that 3253 targets for migraine and 184 targets for CR had 88 overlaps. That is, the 88 overlapping targets may be the key for migraine treatment by CR. The 88 overlapping targets are detailed in Supplementary Table S4.

Chinese medicine has multicompounds and multitargets. To illustrate this feature, we attempted to use these active compounds and outstanding targets. To this end, we used Cytoscape software to build the drug-compound-target-disease (D-C-T-D) network for visualization, as shown in Figure 3(b). The green square node represents the drug (CR), the red round node represents the disease (migraine), 38 pink triangle nodes represent the active compounds in CR, and 88 purple arrow nodes represent the overlapping targets between CR and migraine, which constitute the drug-compound-target-disease (D-C-T-D) network. The centralization and heterogeneity of the network were 0.666 and 1.901, respectively. This network indicates the potential relationship between compounds and targets, which suggests the potential pharmacological mechanism of CR or compounds in the treatment of migraine. The node with the highest degree of connection with other compounds or targets represents the hub in the whole network or, in other words, potential compounds or targets. Here, we use two parameters to help us judge the importance of these nodes: the degree (for connection to the node number of edges) and middle degree of intermediate (betweenness centrality, BC) [62]. For example, the connection degree of the highest compounds is CR32 (clionasterol, degree = 24). CR26 (oleic acid) and CR36 (Xiongterpene) also had high degrees of 22 and seven, respectively. These results suggest that a single compound can act on multiple targets at the same time, suggesting that the active compounds in CR can achieve the goal of treating migraine through multiple targets. Generally, BC can measure the importance of nodes in the network, which can help us find more important nodes [63]. Therefore, if the degree value of some nodes is not high and the BC value is more prominent, then we think that the node is also more important in the network. Generally, there is a positive correlation between degree and BC. However, everything has two sides. Although the degree value of CR31 (ferulic acid, degree = 2, BC = 0.00135) was lower, its BC value was higher than that of other compounds to the same degree. This suggests that we should pay more attention to ferulic acid, which is the quality control compound of the Chinese pharmacopoeia for CR. It has strong biological activity and can not only resist inflammation but also show strong antioxidant activity [64–66]. It can downregulate IL-1*β*, IL-6, and TNF-*α*. Moreover, it can decrease the NLRP3 inflammasome and regulate NF-*κ*B signal transduction, ultimately inhibiting inflammation [67]. For target analysis, PTGS2, PTGS1, and CHRM2 were separately linked to 24, 15, and 10 compounds, respectively. These findings indicate that different compounds can regulate the same target in a cooperative way. These analyses support the view that CR, as a treatment for migraine, has multiple compounds acting on multiple targets. Details of the active compounds of CR and overlapping targets that play key roles are shown in Supplementary Table S5.

### 3.7. Analyses of Protein-Protein Interaction (PPI) Network

**Figure 4:**
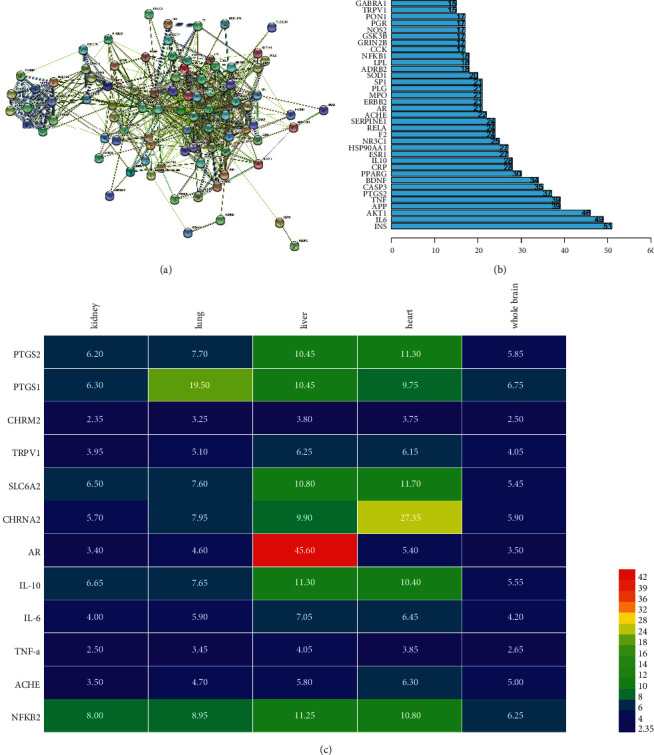
(a) The PPI network. (b) The number of targets that can influence each other in the PPI network. (c) The expression of the core targets in vital organs.

In order to further explore the possible relationship between the overlapping targets, which can help us better analyze the therapeutic mechanism of CR for migraine, we constructed a protein-protein interaction network (PPI network) composed of 88 nodes and 683 edges, as shown in Figures 4(a) and 4(b) (the first 35 targets are intercepted for display). In this PPI network, the degree of the target is proportional to its importance. The results can also provide us with targets worthy of our attention. Details of the PPI network are shown in Supplementary Table S6. As shown in Figure 4(b), we found that targets related to inflammation, such as IL-6, TNF-a, PTGS2, and IL-10, play the more important roles.

To determine the effect of CR on vital organs in the treatment of migraine, we conducted a more in-depth study on the expression of some targets in vital organs, as shown in Figure 4(c). We found that the expression of core targets in various organs of the human body is relatively different. For example, AR was the highest in liver, at 45.6. However, it is much lower in other organs. In the heart, the expression of AR is only 5.40, but it is much higher than the expression in the whole brain, kidney, and lung. The expression of the 11 targets shown in Figure 4(c) in the liver or heart was much higher than that in the other three organs. However, the only exception was PTGS1, which had its highest expression in the lungs. It is, however, worth noting that the expression of this target in the liver and heart was also relatively high. Surprisingly, almost all of the targets in humans were most highly expressed in the liver and in the heart. This also means that CR specifically affects the liver and heart in the treatment of migraine.

### 3.8. Analyses of Gene Ontology (GO) and Kyoto Encyclopedia of Genes and Genomes Pathways (KEGG)

**Figure 5:**
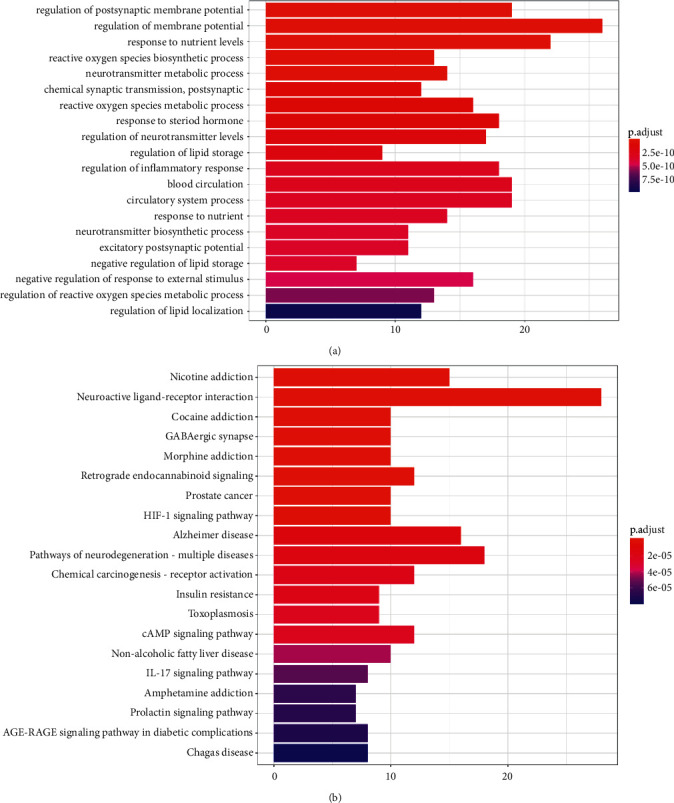
(a) GO enrichment analyses. The *x*-axis represents significant enrichment in the counts of these terms. The *y*-axis represents the categories of “biological process” in the GO of the targets (*P* < 0.01). (b) KEGG pathway enrichment analyses. The *x*-axis represents the counts of the target symbols in each pathway; the *y*-axis represents the main pathways (*P* < 0.01).

To further understand the biological characteristics of 88 key overlapping targets of CR, GO enrichment analysis was performed on the assumed targets to clarify the related biological processes (*P* < 0.01), as shown in Figure 5(a). The results show that the antiobesity effect of ZBM involves several biological processes, including regulation of postsynaptic membrane potential (GO:0060078), regulation of membrane potential (GO:0042391), response to nutrient levels (GO:0031667), and reactive oxygen species biosynthetic process (GO:1903409). Details of the GO enrichment analysis are shown in Supplementary Table S7.

In addition, to further identify potential pathways involved in CR treatment of migraine, we performed KEGG pathway enrichment analysis on these 88 targets. In the end, a total of 98 enrichment pathways associated with CR treatment for migraine were identified, and 20 pathways with higher confidence are presented in Figure 5(b) (*P* < 0.01). Details of the KEGG pathway enrichment analysis are shown in Supplementary Table S8. After analyzing the results, we found that the enriched targets were related to a variety of signalling pathways, mainly neuro-related and inflammation-related pathways. This is particularly manifested in neuroactive ligand-receptor interactions (hsa04080) and the IL-17 signalling pathway (hsa04657). These pathways may be the key pathways responsible for CR in the treatment of migraine. This analysis may provide a new way to explore the mechanism of CG in the treatment of migraine.

### 3.9. Computational Validation of Compounds-Targets Interactions

**Figure 6:**
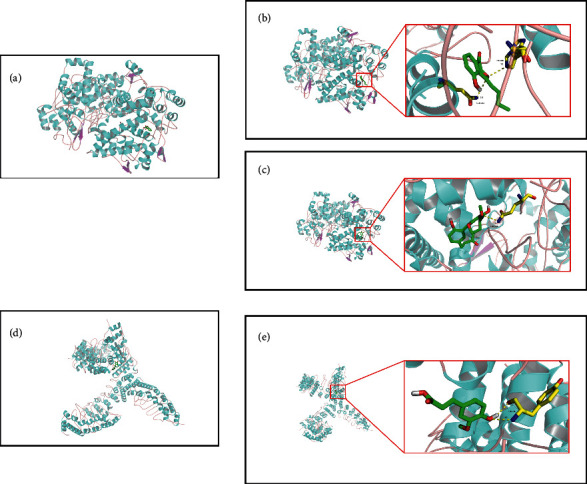
Binding studies of active compounds with PTGS2 and TRPV1 interactions. (a) Aspirin with PTGS2; (b) chuanxiongol with PTGS2; (c) myricanone with PTGS2; (d) capsazepine with TRPV1; and (e) ferulic acid with TRPV1. Molecules are represented by the ball and bar model, hydrogen bonds are represented by dotted lines, and distances are represented by angstroms. Atoms C, O, and N are green, red, and blue, respectively.

It is well known that the binding strength of ligands to receptors is determined by the number of covalent bonds between them and their binding affinity [68]. In order to explore the possible binding mechanism between the active compounds and the predicted targets, we used molecular docking technology. Here, we explored the potential binding modes of the active compounds to PTGS2 and TRPV1, as shown in Figure 6. To verify the robustness of our model, we used the classic PTGS2 inhibitor aspirin and the TRPV1 inhibitor capsazepine as positive controls (as shown in Figures 6(a) and 6(d)). Our results showed that chuanxiongol (Figure 6(b)), myricanone (Figure 6(c)), and the target inhibitor aspirin bind to the same site as PTGS2. Similarly, ferulic acid (Figure 6(e)) and the target corresponding inhibitor capsazepine were combined with TRPV1 in the same pocket. Based on the above results, we believe that the strong interaction between these active compounds and their targets (PTGS2 and TRPV1) is the basis for their effective biological activities. Therefore, from the perspective of computer simulation, these results demonstrate the potential ability of active compounds to treat migraine by affecting their related targets and further verify our prediction results in the D-C-T-D network.

## 4. Discussion

Traditional Chinese medicine always has the characteristics of multicompounds and multitargets when it plays a therapeutic role. It is a great challenge to evaluate the therapeutic efficacy of traditional Chinese medicine given many active compounds. In recent years, systems pharmacology has been an ideal pharmacological research tool for the treatment of diseases with traditional Chinese medicine. We used a knowledge-based and a computing-based strategy to build the network and perform more in-depth research. Systems pharmacology is helpful for discovering the relationship among traditional Chinese medicine, diseases, and molecular targets on the basis of networks and to understand the molecular mechanisms behind therapeutic effects as deeply as possible. In the present study, we first identified the active compounds in CR and their related targets, then obtained the known targets for migraine treatment, and lastly established the D-C-T-D network.

The D-T-C-D network highlighted a total of 88 targets, which may be the key targets for CR in the treatment of migraine. Among these 88 targets, many are related to inflammation, such as IL-6, IL-10, and TNF-*α* [69–71]. Similarly, TRPV1 is closely related to neural activity [72]. In fact, the KEGG pathway enrichment analyses of the 88 targets also highlight the importance of the inflammation module, suggesting that CR may treat migraine through an anti-inflammatory pathway. This conclusion is also supported by the existing literature [73]. In fact, some studies have shown that migraine is closely related to neurogenic neuroinflammation [74, 75]. However, due to the lack of finding standard markers of central nervous system (CNS) inflammation, such as changes in BBB integrity or glial activation or leukocyte infiltration, researchers do not believe that CNS inflammation is involved in migraine attack. Therefore, TRPV1 is also a target of great interest to us. It belongs to the transient receptor potential (TRP) channel family and is a nonselective cation channel [76]. It is mainly expressed in primary afferent sensory neurons, which detect and integrate chemical and thermal stimulation signals to induce pain, convert them into action potentials, upload this information to the central nervous system, and ultimately make the body feel pain or uncomfortable [77]. For example, capsaicin can activate TRPV1 channels, cause calcium influx, and lead to excitation of primary sensory neurons; long-term use leads to neuron desensitization, which blocks the transmission of pain. Furthermore, TRPV1 blockers can also block the initial pathway of pain afferents, providing a new avenue for the clinical treatment of pain [78]. Studies speculate that antagonizing TRPV1 is a promising treatment approach and should receive more attention in future studies and in the development of antimigraine drugs [79, 80]. We think this target is very interesting because in traditional Chinese medicine theory, CR is a drug with the effect of Xin and San. After taking CR, the human body will have a reaction similar to that with pepper, namely, sweating. We believe that this is also the embodiment of TRPV1 macrocontrol.

In this study, we also discussed the channel tropism of CR through Biogps. The theory of traditional Chinese medicine holds that traditional Chinese medicine acts on the whole human body, but the organs that produce curative effects are the focus. In addition, we found that CR affected 11 targets that were more highly expressed in the liver or heart but least expressed in the whole brain. This is a very interesting finding. Migraine is a kind of brain disease, and CR can treat it by acting on the liver and heart. We think that this is a very new and appropriate explanation for the holistic concept of TCM treatment. Of course, more evidence is needed to verify this explanation. Furthermore, we also speculated on the potential mechanism of CR in treating migraine based on the above study, which will be verified in the future.

In conclusion, we systematically explored the mechanism of CR in the treatment of migraine. Our results may provide some unique insights for the treatment of migraine in TCM.

## Figures and Tables

**Table 1:** MS data of (±) ESI- QTOF-MS/MS spectra and the identification of the Chuanxiong Rhizoma extract.

No.	Formula	Precursor ion ()	Error (ppm)	tR (min)	MS/MS (abundance ratio)	Identity
1(−)	C_7_H_12_O_6_	191.0562 ()	0.2	1.15	173.0440, 127.0403, 93.0352, 85.0306	Quinic acid
2(−)	C_7_H_6_O_3_	137.0252	5.8	5.132	108.0230, 108.0230	4-Hydroxybenzoic acid
3(−)	C_13_H_16_O_9_	315.0719	−0.8	4.19	153.0109,152.0107, 109.0297, 108.0223	Protocatechuic acid-3 -glucoside
4(−)	C_16_H_18_O_9_	353.0874	−1.1	5.05	191.0549, 179.0337, 173.0457, 161.0238, 135.0445	Neochlorogenic acid
5(−)	C_16_H_18_O_9_	353.0874	−1.1	6.99	191.0554, 179.0344, 173.0449, 161.0241, 135.0453	Chlorogenic acid
6(−)	C_16_H_18_O_9_	353.0873	−1.5	7.27	191.0555, 179.0342, 173.0451, 161.0243, 135.0450	Cryptochlorogenic acid
7(−)	C_22_H_28_O_14_	515.1404	−0.5	5.28	353.0871, 341.0887, 191.0548, 179.0336, 173.0440, 161.0238, 135.0449	Chlorogenic acid-glucoside
8(−)	C_22_H_28_O_14_	515.1402	−0.8	6.37	353.0877, 341.0882, 323.0765, 191.0550, 179.0336, 173.0438, 161.0232, 135.0441	*iso*-Chlorogenic acid-glucoside
9(−)	C_15_H_18_O_9_	341.0875	−1.0	5.51	179.0337, 135.0444	Caffeic acid 3-glucoside
10(−)	C_15_H_18_O_9_	341.0875	−1.0	5.51	179.0336, 135.0447	Caffeic acid 4-glucoside
11(−)	C_23_H_30_O_14_	529.1566	0.6	6.19	367.0998, 193.0483, 191.0538, 178.0248, 173.0438, 134.0368	Feruloylquinic acid-glucoside
12(−)	C_23_H_30_O_14_	529.1562	−0.2	6.77	355.0989, 337.0346, 193.0493, 178.0262, 173.0450, 134.0365	*iso*-Feruloylquinic acid-glucoside
13(−)	C_16_H_20_O_9_	355.1028	−1.7	6.63	193.0488, 178.0255, 149.0596, 134.0364	Feruloyl-glucose
14(−)	C_8_H_8_O_4_	167.0352	1.4	7.01	123.0447, 79.0561	1-*p*-CoQA^*∗*^
15(−)	C_17_H_20_O_9_	367.1036	0.5	7.27	193.0491, 191.0542, 173.0472, 149.0239, 134.0370	3-O-Feruloylquinic acid
16(−)	C_17_H_20_O_9_	367.1029	−1.4	10.22	193.0492, 191.0545, 173.0440, 134.0361	5-O-Feruloylquinic acid
17(−)	C_9_H_8_O_4_	179.0351	0.7	7.24	135.0444, 134.0365	Caffeic acid
18(−)	C_10_H_10_O_4_	193.0508	0.6	11.24	353.0884(14.9), 335.0774(1.8), 191.0563(100), 178.267, 149.0605, 134.0371	Ferulic acid
19(−)	C_25_H_24_O_12_	515.1190	−0.9	16.46	353.0863, 335.0762, 191.0548, 179.0334, 173.0436, 161.0231, 135.0443	3,5-Dicaffeoylquinic acid
20(−)	C_25_H_24_O_12_	515.1190	−0.9	19.34	353.0862, 335.0756, 191.0538, 179.0334, 173.0431, 161.0246, 135.0441	4,5-Dicaffeoylquinic acid
21(−)	C_25_H_36_O_12_	549.2184	1.2	19.87	503.2132, 311.0974, 293.0872, 251.0751, 221.0654, 191.0551, 149.0448	*trans*-4,5-di-CQA
22(−)	C_12_H_14_O_3_	205.0870	−0.3	28.99	161.0964, 131.0496, 106.0429	Senkyunolide H
23(−)	C_12_H_12_O_3_	203.0713	−0.1	30.15	173.0232, 160.158, 145.0287, 132.0312	Senkyunolide B
24(−)	C_12_H_16_O_3_	207.1026	−0.3	27.74	163.1117, 161.0965	Senkyunolide K
25(+)	C_12_H_16_O_2_	193.1221	−0.9	30.76	147.1163, 105.693, 91.0540, 77.0387	Senkyunolide A
26(+)	C_12_H_14_O_2_	191.1065	−0.9	32.24	173.0956, 128.0618, 115.0537, 91.0541, 77.0388	3-N-Butylphthalide
27(+)	C_24_H_28_O_4_	381.2054	−1.6	35.64	191.1064, 173.0961, 149.0597, 135.0440	Levistilide A
28(+)	C_12_H_12_O_2_	189.0908	−1.1	32.40	171.0803, 152.0619, 128.0620, 115.0546	3-Butylidenephthalide
29(+)	C_12_H_18_O_4_	227.1273	−2.4	12.25	209.1160, 191.1034, 153.0542, 105.0693, 77.0391	Senkyunolide J
30(+)	C_12_H_16_O_4_	225.1119	−1.0	15.32	207.1031, 189.0909, 133.0651, 91.0544	Senkyunolide I
31(+)	C_12_H_14_O_4_	223.0962	−1.3	26.60	205.0865, 149.0233, 121.0281	Senkyunolide D
32(+)	C_12_H_16_O_3_	209.1169	−1.4	12.26	163.1111, 153.0543, 91.0541, 77.0387	Senkyunolide G
33(+)	C_12_H_16_O_4_	207.1014	−0.9	15.31	189.0906, 146.0728, 133.0648, 91.0549, 77.0396	Senkyunolide F

^*∗*^Positive mode: +; negative mode: −

**Table 2:** The active compounds of CR.

No.	Name	CAS no.	OB (%)	DL	References	No.	Name	CAS no.	OB (%)	DL	References

CR01	*α*-Cubebene	17699-14-8	16.73	0.11	[35]	CR20	Aromadendrene oxide	85710-39-0	65.10	0.14	[36]
CR02	Linoleic acid	60-33-3	41.90	0.14	[37]	CR21	(−)-Cedrene	469-61-4	51.14	0.11	[38]
CR03	(−)-Globulol	489-41-8	85.51	0.12	[39]	CR22	(Z)-9-Octadecenoic acid methyl ester	112-62-9	31.90	0.16	[40]
CR04	Mandenol	544-35-4	42.00	0.19	[41]	CR23	Alexandrin	474-58-8	20.63	0.62	[42]
CR05	Methyl linoleate	112-63-0	41.93	0.17	[39]	CR24	Angelicin	83-46-5	36.91	0.75	[43]
CR06	3-Methylchrysazin	481-74-3	18.64	0.21	[42]	CR25	Folacid	59-30-3	68.96	0.71	[43]
CR07	Adenocard	58-61-7	18.82	0.10	[44]	CR26	Oleic acid	112-80-1	33.13	0.14	[45]
CR08	(+)-Aromadendrene	489-39-4	55.74	0.10	[46]	CR27	Cetostearic acid	57-10-3	19.30	0.10	[47]
CR09	Methanoazulene	50894-66-1	52.87	0.10	[48]	CR28	Octadecanoic acid	57-11-4	17.83	0.14	[37]
CR10	*α*-Selinene	473-13-2	31.81	0.10	[49]	CR29	Methyl hexadecanoate	112-39-0	18.09	0.12	[41]
CR11	*β*-Cubebene	13744-15-5	32.16	0.11	[50]	CR30	Isoledene	95910-36-4	49.01	0.10	[39]
CR12	Chuanxiongol	87421-30-5	22.19	0.10	[51]	CR31	Ferulic acid	1135-24-6	Not applied	Not applied	[52]
CR13	Myricanone	32492-74-3	40.60	0.51	[53]	CR32	Clionasterol	83-47-6	Not applied	Not applied	[47]
CR14	Perlolidin	29700-20-7	65.95	0.27	[54]	CR33	Choline	62-49-7	Not applied	Not applied	[55]
CR15	Senkyunolide D	94530-82-2	79.13	0.10	[37]	CR34	Chrysophanic acid	491-59-8	Not applied	Not applied	[42]
CR16	1-Acetyl-beta-carboline	50892-83-6	21.14	0.10	[56]	CR35	Biocolina	67-48-1	Not applied	Not applied	[55]
CR17	Espatulenol	6750-60-3	82.33	0.12	[57]	CR36	Xiongterpene	50627-73-1	23.77	0.42	[58]
CR18	Wallichilide	93236-64-7	42.31	0.71	[53]	CR37	Senkyunolide I	94596-28-8	Not applied	Not applied	[59]
CR19	Pedatisectine C	103805-66-9	25.82	0.12	[60]	CR38	Retinol	68-26-8	Not applied	Not applied	[61]

## Data Availability

The data used to support the findings of our study are included within the manuscript or within the supplementary information files.

## Supplementary Materials

Supplementary Materials Supplementary Table S1: active compounds of CR). A total of 248 candidate compounds were included. Supplementary Table S2: relevant targets of CR active compounds. Supplementary Table S3: targets for Migraine. Supplementary Table S4: overlapping targets between disease and drug. Supplementary Table S5: details of the active compounds of CR and overlapping targets. Supplementary Table S6: the details of the PPI network. Supplementary Table S7: details of the GO enrichment analysis. Supplementary Table S8: details of the KEGG pathway enrichment analysis.

## References

[1] Arnold M. (2018). Headache classification committee of the international headache society (IHS) the international classification of headache disorders. *Cephalalgia*.

[2] Buse D. C., Loder E. W., Gorman J. A. (2013). Sex differences in the prevalence, symptoms, and associated features of migraine, probable migraine and other severe H eadache: results of the A merican migraine prevalence and prevention (AMPP) study. Headache. *Headache: The Journal of Head and Face Pain*.

[3] Noseda R., Burstein R. (2013). Migraine pathophysiology: anatomy of the trigeminovascular pathway and associated neurological symptoms, cortical spreading depression, sensitization, and modulation of pain. *Pain*.

[4] Ferrari M. D., Klever R. R., Terwindt G. M., Ayata C., van den Maagdenberg A. M. J. M. (2015). Migraine pathophysiology: lessons from mouse models and human genetics. *The Lancet Neurology*.

[5] Yılmaz I. A., Özge A., Erdal M. E., Edgünlü T. G., Cakmak S. E., Yalin O. O. (2010). Cytokine polymorphism in patients with migraine: some suggestive clues of migraine and inflammation. *Pain Medicine*.

[6] De Roos N. M., Giezenaar C. G. T., Rovers J. M. P., Witteman B. J. M., Smits M. G., van Hemert S. (2015). The effects of the multispecies probiotic mixture ecologic® barrier on migraine: results of an open-label pilot study. *Beneficial Microbes*.

[7] Chang L., Zeng G., Liu D., Ru W. (2021). Research progress of chuanxiong rhizoma-gastrodiae rhizoma on prevention and treatment of migraine. *Chinese Journal of Modern Applied Pharmacy*.

[8] Guo L., Gong M., Wu S., Qiu F., Ma L. (2020). Identification and quantification of the quality markers and anti-migraine active components in Chuanxiong Rhizoma and Cyperi Rhizoma herbal pair based on chemometric analysis between chemical constituents and pharmacological effects. *Journal of Ethnopharmacology*.

[9] Wang L., Zhang J., Hong Y., Feng Y., Chen M., Wang Y. (2013). Phytochemical and pharmacological review of da chuanxiong formula: a famous herb pair composed of chuanxiong rhizoma and gastrodiae rhizoma for headache. *Evidence-based Complementary and Alternative Medicine*.

[10] Wu S., Guo L., Qiu F., Gong M. (2019). Anti-migraine effect of the herbal combination of chuanxiong rhizoma and cyperi rhizoma and UPLC-MS/MS method for the simultaneous quantification of the active constituents in rat serum and cerebral cortex. *Molecules*.

[11] Huang Y., Ni N., Hong Y., Lin X., Feng Y., Shen L. (2020). Progress in traditional Chinese medicine for the treatment of migraine. *The American Journal of Chinese Medicine*.

[12] Shao L., Zhang B. (2013). Traditional Chinese medicine network pharmacology: theory, methodology and application. *Chinese Journal of Natural Medicines*.

[13] Zhang G.-B., Li Q.-Y., Chen Q.-L., Su S.-bing (2013). Network pharmacology: a new approach for Chinese herbal medicine research. *Evidence-based Complementary and Alternative Medicine*.

[14] Yang S., Zhang J., Yan Y. (2020). Network pharmacology-based strategy to investigate the pharmacologic mechanisms of Atractylodes macrocephala Koidz. for the treatment of chronic gastritis. *Frontiers in Pharmacology*.

[15] Wu W., Yang S., Liu P., Yin L., Gong Q., Zhu W. (2020). Systems pharmacology-based strategy to investigate pharmacological mechanisms of Radix Puerariae for treatment of hypertension. *Frontiers in Pharmacology*.

[16] Wang Y., Yang S. H., Zhong K. (2020). Network pharmacology-based strategy for the investigation of the anti-obesity effects of an ethanolic extract of Zanthoxylum bungeanum Maxim. *Frontiers in Pharmacology*.

[17] Wu J., Ye X., Yang S., Yu H., Zhong L., Gong Q. (2021). Systems pharmacology study of the anti-liver injury mechanism of Citri Reticulatae Pericarpium. *Frontiers in Pharmacology*.

[18] Xu X., Zhang W., Huang C. (2012). A novel chemometric method for the prediction of human oral bioavailability. *International Journal of Molecular Sciences*.

[19] Liu H., Wang J., Zhou W., Wang Y., Yang L. (2013). Systems approaches and polypharmacology for drug discovery from herbal medicines: an example using licorice. *Journal of Ethnopharmacology*.

[20] Alam M. A., Al-Jenoobi F. I., Al-Mohizea A. M., Ali R. (2015). Understanding and managing oral bioavailability: physiological concepts and patents. *Recent Patents on Anti-cancer Drug Discovery*.

[21] Walters W. P., Murcko M. A. (2002). Prediction of ‘drug-likeness’. *Advanced Drug Delivery Reviews*.

[22] Tao W., Xu X., Wang X. (2013). Network pharmacology-based prediction of the active ingredients and potential targets of Chinese herbal Radix Curcumae formula for application to cardiovascular disease. *Journal of Ethnopharmacology*.

[23] Mauri A., Consonni V., Pavan M., Todeschini R., Chemometrics M. (2006). Dragon software: an easy approach to molecular descriptor calculations. *Match*.

[24] Zhu H., Hao J., Niu Y., Liu D., Chen D., Wu X. (2018). Molecular targets of Chinese herbs: a clinical study of metastatic colorectal cancer based on network pharmacology. *Scientific Reports*.

[25] Yang L., Liu W., Hu Z. (2019). A systems pharmacology approach for identifying the multiple mechanisms of action of the Wei Pi Xiao decoction for the treatment of gastric precancerous lesions. *Evidence-based Complementary and Alternative Medicine*.

[26] Liu H., Zeng L., Yang K., Zhang G. (2016). A network pharmacology approach to explore the pharmacological mechanism of xiaoyao powder on anovulatory infertility. *Evidence-based Complementary and Alternative Medicine*.

[27] Trott O., Olson A. J. (2010). AutoDock Vina: improving the speed and accuracy of docking with a new scoring function, efficient optimization, and multithreading. *Journal of Computational Chemistry*.

[28] Morris G. M., Huey R., Lindstrom W. (2009). AutoDock4 and AutoDockTools4: automated docking with selective receptor flexibility. *Journal of Computational Chemistry*.

[29] Winn M. D., Ballard C. C., Cowtan K. D. (2011). Overview of the CCP4 suite and current developments. *Acta Crystallographica Section D Biological Crystallography*.

[30] Li W., Tang Y., Chen Y., Duan J.-A. (2012). Advances in the chemical analysis and biological activities of chuanxiong. *Molecules*.

[31] Chawla A. S., Singh M., Murthy M. S., Gupta M., Singh H. (1987). Anti-inflammatory action of ferulic acid and its esters in carrageenan induced rat paw oedema model. *Indian Journal of Experimental Biology*.

[32] Barone E., Calabrese V., Mancuso C. (2009). Ferulic acid and its therapeutic potential as a hormetin for age-related diseases. *Biogerontology*.

[33] Mathew S., Abraham T. E. (2004). Ferulic acid: an antioxidant found naturally in plant cell walls and feruloyl esterases involved in its release and their applications. *Critical Reviews in Biotechnology*.

[34] Hou Y. Z., Yang J., Zhao G. R., Yuan Y. J. (2004). Ferulic acid inhibits vascular smooth muscle cell proliferation induced by angiotensin II. *European Journal of Pharmacology*.

[35] He M., Yan P., Yang Z.-Y., Zhang Z.-M., Yang T.-B., Hong L. (2018). A modified multiscale peak alignment method combined with trilinear decomposition to study the volatile/heat-labile components in Ligusticum chuanxiong Hort-Cyperus rotundus rhizomes by HS-SPME-GC/MS. *Journal of Chromatography B*.

[36] Li B., Xu X., Wang X. (2012). A systems biology approach to understanding the mechanisms of action of Chinese herbs for treatment of cardiovascular disease. *International Journal of Molecular Sciences*.

[37] He M., Peng G., Xie F., Hong L., Cao Q. (2019). Liquid chromatography–high-resolution mass spectrometry with ROI strategy for non-targeted analysis of the in vivo/in vitro ingredients coming from ligusticum chuanxiong hort. *Chromatographia*.

[38] Hui F., Rong L., Mingming L., Zhou S., Fu R. (2015). Analysis of volatile components of Ligusticum Chuanxiong Hort by solid phase microextraction and gas chromatography-mass. *Journal of Zunyi Medical University*.

[39] Yang G., Sun Q., Hu Z., Liu H., Zhou T., Fan G. (2015). Optimization of an accelerated solvent extraction dispersive liquid–liquid microextraction method for the separation and determination of essential oil from Ligusticum chuanxiong Hort by gas chromatography with mass spectrometry. *Journal of Separation Science*.

[40] Zhang W., Qiu T.-Q. (2011). Extraction for volatile oil from compound recipe of Ligusticum chuanxiong Hort. and Salvia miltiorrhiza Bge. with ultrasonic enhanced supercritical CO_2. *Food Science and Technology*.

[41] Zhang H., Han T., Yu C.-H. (2012). Analysis of the chemical composition, acute toxicity and skin sensitivity of essential oil from rhizomes of Ligusticum chuanxiong. *Journal of Ethnopharmacology*.

[42] Wang W.-X., Gu M., Jiang X.-G., Baba K. (2002). Studies on chemical constituents of Ligusticum chuanxiong. *Chinese Traditional and Herbal Drugs*.

[43] Hu Y., Liu C., Hu Y., Zhang Y.-C. (2012). Determination of chemical composition in extract of ligusticum Chuanxiong by liquid chromatography-electrospray Ionization mass spectrometry. *Lishizhen Med. Mater. Med. Res*.

[44] Zhang Q., Yang Y.-X., Li S.-Y. (2017). An ultrafiltration and high performance liquid chromatography coupled with diode array detector and mass spectrometry approach for screening and characterizing thrombin inhibitors from Rhizoma Chuanxiong. *Journal of Chromatography B*.

[45] Liu Q., Song T., Gan G.-P. (2008). GC Fingerprints of Ligusticum chuanxiong essential oil. *Chinese Traditional and Herbal Drugs*.

[46] Zhong F., Yang L., Ji L., Fu G. (1996). Studies on the essential oils in Ligusticum chuanxiong Hort. of different habitats and species. *China Journal of Chinese Materia Medica*.

[47] Wang Y., Yan Z., Ma Y., Chen X., Song J., Wan D. (2010). Analysis of Ligusticum chuanxiong oil of different varieties and producing areas by GC-MS. *Chinese Traditional Patent Medicine*.

[48] Yuping Y. (2013). The Wu bene experience of treatment with headache from the wind. *Journal of Practical Traditional Chinese Internal Medicine*.

[49] Zhou Y., Yan P., He M., Hong L., Cao Q. (2019). Hyphenated chromatography detection and compound-target-disease investigation on herb-pair Chuanxiong Rhizoma-Xiangfu Rhizoma. *Journal of Ethnopharmacology*.

[50] Shi L., Zheng X., Cai Z., Wu B. (1995). Comparison of influence of essential oil from Ligusticum chuanxiong Hort. on microcirculation in rabbit conjunctiva bulbar before and after decomposition of ligustilide. *Chinese Journal of Pharmacology and Toxicology*.

[51] Lu X., Xu H., Shi D. (2006). Effects of Xiongshao Capsule on blood vessel collagens in rabbits with experimental atherosclerosis. *Chinese Journal of Arteriosclerosis*.

[52] Zhang X., Han B., Feng Z.-M., Yang Y.-N., Jiang J.-S., Zhang P.-C. (2018). Ferulic acid derivatives from Ligusticum chuanxiong. *Fitoterapia*.

[53] Hu S., Chen S., Li Z., Wang Y., Wang Y. (2020). Research on the potential mechanism of Chuanxiong Rhizoma on treating Diabetic Nephropathy based on network pharmacology. *International Journal of Medical Sciences*.

[54] Li F., Kong X., Chen P., Li Z., An-wei D. (2011). Effects of carbonized typhae pollen on hemorheological parameters, clotting time and tongue presentations in rats with blood-stasis. *Chinese Journal of Experimental Traditional Medical Formulae*.

[55] Xie Y., Liu H., Lin L. (2019). Application of natural deep eutectic solvents to extract ferulic acid from Ligusticum chuanxiong Hort with microwave assistance. *RSC Advances*.

[56] Pu Z.-H., Dai M., Xiong L., Peng C. (2020). Total alkaloids from the rhizomes of Ligusticum striatum: a review of chemical analysis and pharmacological activities. *Natural Product Research*.

[57] Chen L., Hou J., Deng W., Jiang G. (2020). Quality comparison of traditional Chuanxiong produced in Dujiangyan City and Sichuan ProvinceC and Chuanxiong from other areas, based on analysis of volatile oil, total alkaloids and total ferulic acid contents. *Tropical Journal of Pharmaceutical Research*.

[58] Chang X., Ma Y., Zhang X., Jiang Z.-Y., Chen J.-J. (2007). Studies on chemical constituents of rhizomes of Ligusticum chuanxiong. *China Journal of Chinese Materia Medica*.

[59] He C.-Y., Wang S., Feng Y. (2012). Pharmacokinetics, tissue distribution and metabolism of senkyunolide I, a major bioactive component in Ligusticum chuanxiong Hort.(Umbelliferae). *Journal of Ethnopharmacology*.

[60] Zhang Q., Su Y., Liu X., Guo Y. (2018). Rapid characterization of nonpolar or low‐polarity solvent extracts from herbal medicines by solvent‐assisted electrospray ionization mass spectrometry. *Rapid Communications in Mass Spectrometry*.

[61] Jin D., Qu L., Dai Q. (2015). Clinical observation on effects of yishen huoxue decoction on urinary retinol binding protein( RBP) and *β*2-microglobubin( *β*2-MG) of diabetic nephropathy patients. *Chinese Archives of Traditional Chinese Medicine*.

[62] Brandes U. (2001). A faster algorithm for betweenness centrality. *Journal of Mathematical Sociology*.

[63] Grobelny B. T., London D., Hill T. C., North E., Dugan P., Doyle W. K. (2018). Betweenness centrality of intracranial electroencephalography networks and surgical epilepsy outcome. *Clinical Neurophysiology*.

[64] Kikuzaki H., Hisamoto M., Hirose K., Akiyama K., Taniguchi H. (2002). Antioxidant properties of ferulic acid and its related compounds. *Journal of Agricultural and Food Chemistry*.

[65] Urbaniak A., Szeląg M., Molski M. (2013). Theoretical investigation of stereochemistry and solvent influence on antioxidant activity of ferulic acid. *Computational and Theoretical Chemistry*.

[66] Chowdhury S., Ghosh S., Das A. K., Sil P. C. (2019). Ferulic acid protects hyperglycemia-induced kidney damage by regulating oxidative insult, inflammation and autophagy. *Frontiers in Pharmacology*.

[67] Liu Y.-M., Shen J.-D., Xu L.-P., Li H.-B., Li Y.-C., Yi L.-T. (2017). Ferulic acid inhibits neuro-inflammation in mice exposed to chronic unpredictable mild stress. *International Immunopharmacology*.

[68] Kuo S. C., Lauffenburger D. A. (1993). Relationship between receptor/ligand binding affinity and adhesion strength. *Biophysical Journal*.

[69] Tanaka T., Narazaki M., Kishimoto T. (2014). IL-6 in inflammation, immunity, and disease. *Cold Spring Harbor Perspectives in Biology*.

[70] Jones C. A., Cayabyab R. G., Kwong K. Y. C. (1996). Undetectable interleukin (IL)-10 and persistent IL-8 expression early in hyaline membrane disease: a possible developmental basis for the predisposition to chronic lung inflammation in preterm newborns. *Pediatric Research*.

[71] Zelová H., Hošek J. (2013). TNF-*α* signalling and inflammation: interactions between old acquaintances. *Inflammation Research: Official Journal of the European Histamine Research Society*.

[72] Gao X., Zhuang J., Zhao L., Wei W., Xu F. (2021). Cross-effect of TRPV1 and EP3 receptor on coughs and bronchopulmonary C-neural activities. *PLoS One*.

[73] Longoni M., Ferrarese C. (2006). Inflammation and excitotoxicity: role in migraine pathogenesis. *Neurological Sciences*.

[74] Williamson D. J., Hargreaves R. J. (2001). Neurogenic inflammation in the context of migraine. *Microscopy Research and Technique*.

[75] Edvinsson L., Haanes K. A., Warfvinge K. (2019). Does inflammation have a role in migraine?. *Nature Reviews Neurology*.

[76] Caterina M. J., Schumacher M. A., Tominaga M., Rosen T. A., Levine J. D., Julius D. (1997). The capsaicin receptor: a heat-activated ion channel in the pain pathway. *Nature*.

[77] Dinh Q. T., Groneberg D. A., Mingomataj E. (2003). Expression of substance P and vanilloid receptor (VR1) in trigeminal sensory neurons projecting to the mouse nasal mucosa. *Neuropeptides*.

[78] Tóth A., Blumberg P. M., Chen Z., Kozikowski A. P. (2004). Design of a high-affinity competitive antagonist of the vanilloid receptor selective for the calcium entry-linked receptor population. *Molecular Pharmacology*.

[79] Meents J. E., Neeb L., Reuter U. (2010). TRPV1 in migraine pathophysiology. *Trends in Molecular Medicine*.

[80] Farajdokht F., Mohaddes G., Shanehbandi D., Karimi P., Babri S. (2018). Ghrelin attenuated hyperalgesia induced by chronic nitroglycerin: CGRP and TRPV1 as targets for migraine management. *Cephalalgia*.

