# Supplementary material for: Exploring the Potential Mechanism of Chuanxiong Rhizoma Treatment for Migraine Based on Systems Pharmacology
Source: Evid Based Complement Alternat Med. 2021 Dec 28;2021:2809004. doi: 10.1155/2021/2809004 (PMC8727101; doi:10.1155/2021/2809004)
Supplement: Supplementary Materials — Supplementary Table S1: active compounds of CR). A total of 248 candidate compounds were included. Supplementary Table S2: relevant targets of CR active compounds. Supplementary Table S3: targets for Migraine. Supplementary Table S4: overlapping targets between disease and drug. Supplementary Table S5: details of the active compounds of CR and overlapping targets. Supplementary Table S6: the details of the PPI network. Supplementary Table S7: details of the GO enrichment analysis. Supplementary Table S8: details of the KEGG pathway enrichment analysis. [file 2809004.f1.zip › 2809004.f1/Supplementary Table S1 active compounds of CR (1).doc]

| No. | Name | CAS No. |
| --- | --- | --- |
| 1 | Paraben-acid | 99-96-7 |
| 2 | (-)-α-Thujene | 3917-48-4 |
| 3 | Vanillic acid | 121-34-6 |
| 4 | Nonaldehyde | 124-19-6 |
| 5 | 4-Cymene | 99-87-6 |
| 6 | α-Cubebene | 17699-14-8 |
| 7 | (-)-α-Terpineol | 10482-56-1 |
| 8 | α-Bergamotene | 17699-05-7 |
| 9 | (-)-Phellandral | 23963-70-4 |
| 10 | 1,8-Cineole | 470-82-6 |
| 11 | Campholenal | 23727-15-3 |
| 12 | (-)-2-Pinene | 7785-26-4 |
| 13 | (-)-Nopinene | 18172-67-3 |
| 14 | 2-Acetyl-4-methylphenol | 1450-72-2 |
| 15 | Linoleic acid | 60-33-3 |
| 16 | Benzylic alcohol | 100-51-6 |
| 17 | (-)-Globulol | 489-41-8 |
| 18 | Mandenol | 544-35-4 |
| 19 | 1-Methyl-5-methylene-8-(1-methylethyl)-1,6-cyclodecadiene | 37839-63-7 |
| 20 | Methyl linoleate | 112-63-0 |
| 21 | Agarospirol | 1460-73-7 |
| 22 | 2-Furanal | 98-01-1 |
| 23 | 3-Methylchrysazin | 481-74-3 |
| 24 | 2,4-Pyrimidinediol | 66-22-8 |
| 25 | 4-Vinylguaiacol | 7786-61-0 |
| 26 | Adenocard | 58-61-7 |
| 27 | Adeninimine | 73-24-5 |
| 28 | Methyl pentadecanoate | 7132-64-1 |
| 29 | (+)-Camphene | 5794-03-6 |
| 30 | (-)-Bornyl acetate | 5655-61-8 |
| 31 | Myrcene | 123-35-3 |
| 32 | Licareol | 126-91-0 |
| 33 | (+)-α-Phellandrene | 2243-33-6 |
| 34 | (E)-Ocimene | 27400-72-2 |
| 35 | Moslene | 99-85-4 |
| 36 | (+)-β-Phellandrene | 6153-16-8 |
| 37 | (1S,2R,4R)-1-Ethenyl-1-methyl-2,4-bis(1-methylethenyl)cyclohexane | 674819-49-9 |
| 38 | Apiguard | 89-83-8 |
| 39 | Methyleugenol | 93-15-2 |
| 40 | (+)-Aromadendrene | 489-39-4 |
| 41 | methanoazulene | 50894-66-1 |
| 42 | 1,1-Dimethylcyclopentane | 1638-26-2 |
| 43 | Senkyunolide B | 93236-67-0 |
| 44 | Senkyunolide K | 114569-33-4 |
| 45 | Senkyunolide M | 114569-34-5 |
| 46 | Senkyunolide N | 140694-58-2 |
| 47 | Senkyunolide O | 142797-35-1 |
| 48 | Senkyunolide P | 142864-23-1 |
| 49 | Senkyunolide Q | 142235-81-2 |
| 50 | Senkyunolide R | 172549-37-0 |
| 51 | 1,1-Diethoxybutane | 3658-95-5 |
| 52 | Pentanophenone | 1009-14-9 |
| 53 | Verbenene | 4080-46-0 |
| 54 | Riligustilide | 89354-45-0 |
| 55 | Alloocimene | 673-84-7 |
| 56 | (E)-3-Butylidenephthalide | 76681-73-7 |
| 57 | α-Selinine | 473-13-2 |
| 58 | rel-(1R,4R,4aR)-1,2,3,4,4a,7-Hexahydro-1,6-dimethyl-4-(1-methylethyl)naphthalene | 1432902-90-3 |
| 59 | Augustic acid | 26707-60-8 |
| 60 | 3-Ethyltoluene | 620-14-4 |
| 61 | β-Acoradiene | 28477-64-7 |
| 62 | (-)-β-Sesquiphellandrene | 20307-83-9 |
| 63 | β-Eudesmene | 18423-23-9 |
| 64 | 2,5,5-Trimethyl-1,3,6-heptatriene | 29548-02-5 |
| 65 | β-Cubebene | 13744-15-5 |
| 66 | Bicyclogermacren | 24703-35-3 |
| 67 | Butylidenephthalide | 551-08-6 |
| 68 | Chuanxiongol | 87421-30-5 |
| 69 | (Z)-Asarone | 5273-86-9 |
| 70 | (Z)-Piperitol | 16721-38-3 |
| 71 | Sabinene hydrate | 546-79-2 |
| 72 | Cnidilide | 3674-03-1 |
| 73 | 1,3,8-p-Menthatriene | 18368-95-1 |
| 74 | (Z)-Cyclododecene | 1129-89-1 |
| 75 | Pyronane | 6783-92-2 |
| 76 | 2,9-Dimethyldecane | 1002-17-1 |
| 77 | Selina-4,11-diene | 17627-30-4 |
| 78 | 1,5,5-Trimethyl-6-methylenecyclohexene | 514-95-4 |
| 79 | 2-Methylpropiophenone | 611-70-1 |
| 80 | Myricanone | 32492-74-3 |
| 81 | Neocnidilide | 4567-33-3 |
| 82 | Octane | 111-65-9 |
| 83 | p-Cymene-8-ol | 1197-01-9 |
| 84 | Hydracetin | 114-83-0 |
| 85 | Perlolidin | 29700-20-7 |
| 86 | Arthenolone | 145-13-1 |
| 87 | Sedanoic acid | 6697-07-0 |
| 88 | Senkyunolide C | 91652-78-7 |
| 89 | Senkyunolide D | 94530-82-2 |
| 90 | Senkyunolide E | 94530-83-3 |
| 91 | Senkyunolide F | 94530-84-4 |
| 92 | Senkyunolide J | 94530-86-6 |
| 93 | Senkyunolide L | 114586-51-5 |
| 94 | Senkyunolide S | 172723-28-3 |
| 95 | 1-Acetyl-β-carboline | 50892-83-6 |
| 96 | Senkyunone | 142182-61-4 |
| 97 | Sinapic acid | 530-59-6 |
| 98 | Espatulenol | 6750-60-3 |
| 99 | 2-Nonenol | 22104-79-6 |
| 100 | (E)-Piperitol | 16721-39-4 |
| 101 | N-Trimethylamine | 75-50-3 |
| 102 | Wallichilide | 93236-64-7 |
| 103 | 2,7-Dimethyl-1-octanol | 15250-22-3 |
| 104 | 1-Terpinenol | 586-82-3 |
| 105 | Pedatisectine C | 103805-66-9 |
| 106 | 2-Propionylfuran | 3194-15-8 |
| 107 | Propriophenone | 93-55-0 |
| 108 | 2,2,3-Trimethyl-3-cyclopentene-1-carboxaldehyde | 33843-21-9 |
| 109 | Methyl 2-pentanoylbenzoate | 64624-87-9 |
| 110 | 2-Methylhexane | 591-76-4 |
| 111 | Methyl 2-furoate | 611-13-2 |
| 112 | Isobutenylbenzene | 768-49-0 |
| 113 | 2-methyl-5-(1-methylene)-1,3-cyclohexadiene | Not found |
| 114 | (2S,4S)-Carveol | 7632-16-8 |
| 115 | 2-Methylbenzoxazole | 95-21-6 |
| 116 | rel-(5R,6S)-Tetrahydro-5,6-dimethyl-2H-pyran-2-one | 24405-16-1 |
| 117 | 3(S)-3-Butyl-4,5-dihydrophthalide | Not found |
| 118 | 3,4-epoxy-2,2,7,7-tetramethyl-octane | Not found |
| 119 | 3-Cyclohexenol | 822-66-2 |
| 120 | Methyl 3-furoate | 13129-23-2 |
| 121 | 4,7-Dihydroxy-3-butylphthalide | Not found |
| 122 | 2,3-Dihydro-4-iodo-1H-indole | 939759-03-2 |
| 123 | 4-Oxooctane | 589-63-9 |
| 124 | 4-Hydroxy-3-butylphthalide | 74459-23-7 |
| 125 | β-Spathulenol | 77171-55-2 |
| 126 | 2-Thio-5-propyluracil | 2954-52-1 |
| 127 | (-)-Dictyotene | 33156-91-1 |
| 128 | 1,4-Cineol | 470-67-7 |
| 129 | Aromadendrene oxide | 85710-39-0 |
| 130 | Amylbenzene | 538-68-1 |
| 131 | (+)-Dimethyl malate | 70681-41-3 |
| 132 | 3-Butylphthalide | 6066-49-5 |
| 133 | (-)-Cedrene | 469-61-4 |
| 134 | (+)-Carotol | 465-28-1 |
| 135 | Coniferyl ferulate | 63644-62-2 |
| 136 | Cerulignol | 2785-87-7 |
| 137 | 1,6-Dimethylidene-4-(propan-2-yl)-decahydronaphthalene | 54932-90-0 |
| 138 | Germacrene D | 23986-74-5 |
| 139 | Caprophenone | 942-92-7 |
| 140 | 1,3,5-Undecatriene | 16356-11-9 |
| 141 | Skellysolve C | 142-82-5 |
| 142 | L-Valyl-L-valyl-L-valyl-L-valyl-L-valine | 350702-73-7 |
| 143 | Diligustilide | 88182-33-6 |
| 144 | (E)-Ligustilide | 81944-08-3 |
| 145 | Ligustrazin | 1124-11-4 |
| 146 | (Z)-9-Octadecenoic acid methyl ester | 112-62-9 |
| 147 | Linoleic alcohol | 506-43-4 |
| 148 | Octatriacontane | 7194-85-6 |
| 149 | o-Amylbenzyl alcohol | 110683-66-4 |
| 150 | Sedanolide | 6415-59-4 |
| 151 | Senkyunolide A | 63038-10-8 |
| 152 | Senkyunolide G | 94530-85-5 |
| 153 | Senkyunolide H | 94596-27-7 |
| 154 | (E)-Caffeic acid | 501-16-6 |
| 155 | (-)-Limonene | 5989-54-8 |
| 156 | Humulene | 6753-98-6 |
| 157 | Isoterpinene | 586-62-9 |
| 158 | (-)-Sabinene | 10408-16-9 |
| 159 | (-)-Curcumene | 4176-17-4 |
| 160 | Isodiprene | 498-15-7 |
| 161 | Hendecanoic acid | 112-37-8 |
| 162 | α-Elemol | 639-99-6 |
| 163 | Selina-4(14),11-diene | 17066-67-0 |
| 164 | Alexandrin | 474-58-8 |
| 165 | Angelicin | 83-46-5 |
| 166 | Folacid | 59-30-3 |
| 167 | Origanol | 2438-10-0 |
| 168 | Vanillin | 121-33-5 |
| 169 | hexanal | 66-25-1 |
| 170 | 2-Pentylfuran | 3777-69-3 |
| 171 | Oleic acid | 112-80-1 |
| 172 | Cetostearic acid | 57-10-3 |
| 173 | (-)-α-Phellandrene | 4221-98-1 |
| 174 | Enanthal | 111-71-7 |
| 175 | Octanal | 124-13-0 |
| 176 | 2-Isopropyltoluene | 527-84-4 |
| 177 | Amerfond | 57-50-1 |
| 178 | Octadecanoic acid | 57-11-4 |
| 179 | Pentadecane | 629-62-9 |
| 180 | Methyl hexadecanoate | 112-39-0 |
| 181 | Tetradecane | 629-59-4 |
| 182 | β-Elemene | 515-13-9 |
| 183 | Terpilene | 99-86-5 |
| 184 | (-)-4-Terpineol | 20126-76-5 |
| 185 | Farnesene | 502-61-4 |
| 186 | Bisabolene | 495-62-5 |
| 187 | Isoledene | 95910-36-4 |
| 188 | Ethyl hexadecanoate | 628-97-7 |
| 189 | 2-Borneol | 507-70-0 |
| 190 | Camphene | 79-92-5 |
| 191 | Camphor | 76-22-2 |
| 192 | Citronellol | 106-22-9 |
| 193 | Butyl phthalate | 84-74-2 |
| 194 | (-)-Fenchone | 7787-20-4 |
| 195 | Menthyl acetate | 89-48-5 |
| 196 | Neocomplanoside | 123442-25-1 |
| 197 | Hyacinthin | 122-78-1 |
| 198 | Sabenene | 3387-41-5 |
| 199 | 1-Terpen-4-ol | 562-74-3 |
| 200 | Tridecane | 629-50-5 |
| 201 | Vallesiachotamine | 5523-37-5 |
| 202 | Thiamine | 59-43-8 |
| 203 | Caryophyllene | 87-44-5 |
| 204 | Cinen | 138-86-3 |
| 205 | 3-Carene | 13466-78-9 |
| 206 | Pentadecanoic acid | 1002-84-2 |
| 207 | 2(10)-Pinene | 127-91-3 |
| 208 | Acintene A | 80-56-8 |
| 209 | Concanavalin A | 11028-71-0 |
| 210 | Pentadecanoic acid, ethyl ester | 41114-00-5 |
| 211 | 1-Heptadecanol | 1454-85-9 |
| 212 | Azelaic acid | 123-99-9 |
| 213 | Methyl linolenate | 301-00-8 |
| 214 | Menthadiene | 99-83-2 |
| 215 | Biofene | 18794-84-8 |
| 216 | Butal | 123-72-8 |
| 217 | Aminobutylcanavalmine | 129225-32-7 |
| 218 | Canaline | 15985-61-2 |
| 219 | Canavalia gibberellin I | 18450-93-6 |
| 220 | Canavalia gibberellin II | 18450-94-7 |
| 221 | Canavanin | 543-38-4 |
| 222 | Chuanxiongzine hydrochloride | 76494-51-4 |
| 223 | Cibarian | 39797-90-5 |
| 224 | Citronellol acetate | 150-84-5 |
| 225 | 5,6-Dihydroergosterol | 17608-76-3 |
| 226 | Dillapiol | 484-31-1 |
| 227 | Linalool oxide II | 34995-77-2 |
| 228 | Homoserine | 672-15-1 |
| 229 | Homospermidine | 4427-76-3 |
| 230 | 2-Methoxy-4-(3-methoxy-1-propenyl)phenol | 63644-71-3 |
| 231 | 1-Methoxy-2-methylanthraquinone | 20460-44-0 |
| 232 | α-Methylbenzenemethanol | 98-85-1 |
| 233 | Trimethylamine oxide | 1184-78-7 |
| 234 | Ligustilide A | 81944-09-4 |
| 235 | 3,4-Dimethoxycinnamic acid | 2316-26-9 |
| 236 | Ferulic acid | 1135-24-6 |
| 237 | Clionasterol | 83-47-6 |
| 238 | Caffeic Acid | 331-39-5 |
| 239 | Protocatechuic Acid | 99-50-3 |
| 240 | Choline | 62-49-7 |
| 241 | Chrysophanic Acid | 491-59-8 |
| 242 | Biocolina | 67-48-1 |
| 243 | 1-Monopalmitin | 542-44-9 |
| 244 | Xiongterpene | 50627-73-1 |
| 245 | Senkyunolide I | 94596-28-8 |
| 246 | Ethyl heptadecanoate | 14010-23-2 |
| 247 | Ethyl octadecanoate | 111-61-5 |
| 248 | Retinol | 68-26-8 |

S1 active compounds of CR
