# Supplementary material for: Exploring the Potential Mechanism of Chuanxiong Rhizoma Treatment for Migraine Based on Systems Pharmacology
Source: Evid Based Complement Alternat Med. 2021 Dec 28;2021:2809004. doi: 10.1155/2021/2809004 (PMC8727101; doi:10.1155/2021/2809004)
Supplement: Supplementary Materials — Supplementary Table S1: active compounds of CR). A total of 248 candidate compounds were included. Supplementary Table S2: relevant targets of CR active compounds. Supplementary Table S3: targets for Migraine. Supplementary Table S4: overlapping targets between disease and drug. Supplementary Table S5: details of the active compounds of CR and overlapping targets. Supplementary Table S6: the details of the PPI network. Supplementary Table S7: details of the GO enrichment analysis. Supplementary Table S8: details of the KEGG pathway enrichment analysis. [file 2809004.f1.zip › 2809004.f1/Supplementary Table S2 The relevant targets of CR active compounds (1).doc]

| No. | Name | Target |
| --- | --- | --- |
| CR01 | α-Cubebene | CHRM3 |
|  |  | CHRM1 |
|  |  | PTGS2 |
|  |  | CHRM2 |
|  |  | CHRNA2 |
| CR02 | Linoleic acid | PTGS1 |
|  |  | PTGS2 |
|  |  | RXRA |
|  |  | NCOA2 |
|  |  | LYZ |
|  |  | SLC6A2 |
|  |  | TRPV1 |
|  |  | CHRM1 |
|  |  | CHRM2 |
| CR03 | (-)-Globulol | CHRM3 |
|  |  | CHRM1 |
|  |  | CHRM2 |
|  |  | PTGS2 |
| CR04 | Mandenol | PTGS1 |
|  |  | PTGS2 |
|  |  | NCOA2 |
| CR05 | Methyl linoleate | PTGS1 |
|  |  | PTGS2 |
|  |  | NCOA2 |
|  |  | RXRA |
| CR06 | 3-Methylchrysazin | PTGS1 |
|  |  | PTGS2 |
|  |  | HSP90AA1 |
|  |  | NCOA2 |
|  |  | SCN5A |
|  |  | PDE3A |
|  |  | PRKACA |
|  |  | PKIA |
| CR07 | Adenocard | PTGS1 |
|  |  | MTAP |
|  |  | PTGS2 |
|  |  | PTPN1 |
|  |  | ADA |
| CR08 | (+)-Aromadendrene | CHRM3 |
|  |  | CHRM2 |
|  |  | CHRM1 |
|  |  | LYZ |
| CR09 | methanoazulene | CHRM3 |
|  |  | CHRM1 |
|  |  | RXRA |
|  |  | CHRM2 |
|  |  | ADRA1B |
|  |  | CHRNA2 |
|  |  | NCOA2 |
|  |  | PTGS2 |
| CR10 | α-Selinine | CHRM3 |
|  |  | CHRM1 |
|  |  | PTGS2 |
|  |  | RXRA |
|  |  | CHRM2 |
|  |  | NCOA2 |
|  |  | ADRA1B |
| CR11 | β-Cubebene | CHRM3 |
|  |  | CHRM1 |
|  |  | PTGS2 |
|  |  | CHRM2 |
| CR12 | Chuanxiongol | PTGS1 |
|  |  | CHRM3 |
|  |  | CHRM1 |
|  |  | SCN5A |
|  |  | PTGS2 |
|  |  | RXRA |
|  |  | PDE3A |
|  |  | SLC6A2 |
|  |  | ADRA1B |
|  |  | SLC6A3 |
|  |  | ADRB2 |
|  |  | SLC6A4 |
|  |  | HSP90AA1 |
|  |  | LTA4H |
|  |  | MAOB |
|  |  | PRKACA |
|  |  | PKIA |
| CR13 | Myricanone | NOS2 |
|  |  | PTGS1 |
|  |  | F2 |
|  |  | KCNH2 |
|  |  | ESR1 |
|  |  | AR |
|  |  | SCN5A |
|  |  | PPARG |
|  |  | PTGS2 |
|  |  | F7 |
|  |  | KDR |
|  |  | RXRA |
|  |  | PDE3A |
|  |  | ADRB2 |
|  |  | ESR2 |
|  |  | DPP4 |
|  |  | MAPK14 |
|  |  | GSK3B |
|  |  | HSP90AA1 |
|  |  | CHEK1 |
|  |  | CCNA2 |
|  |  | NCOA1 |
| CR14 | Perlolidin | F2 |
|  |  | PTGS2 |
|  |  | RXRA |
|  |  | PRKACA |
| CR15 | Senkyunolide D | PTGS2 |
|  |  | GRIA2 |
| CR16 | 1-Acetyl-beta-carboline | PTGS1 |
|  |  | SCN5A |
|  |  | PTGS2 |
|  |  | HSP90AA1 |
|  |  | LTA4H |
|  |  | MAOB |
|  |  | PRKACA |
|  |  | PKIA |
| CR17 | Espatulenol | CHRM3 |
|  |  | CHRM1 |
|  |  | CHRM2 |
| CR18 | Wallichilide | PTGS2 |
|  |  | NR3C2 |
|  |  | NR3C1 |
|  |  | NCOA2 |
| CR19 | Pedatisectine C | PTGS1 |
|  |  | PTGS2 |
|  |  | HSP90AA1 |
| CR20 | Aromadendrene oxide | CHRM3 |
|  |  | CHRM1 |
|  |  | CHRM2 |
| CR21 | (-)-Cedrene | CHRM3 |
|  |  | CHRM1 |
|  |  | PTGS2 |
|  |  | RXRA |
|  |  | CHRM2 |
|  |  | ADRA1B |
|  |  | CHRNA2 |
| CR22 | (Z)-9-Octadecenoic acid methyl ester | PTGS1 |
|  |  | PTGS2 |
|  |  | RXRA |
|  |  | NCOA2 |
| CR23 | Alexandrin | PGR |
|  |  | PTGS1 |
|  |  | CHRM3 |
|  |  | KCNH2 |
|  |  | CHRM1 |
|  |  | SCN5A |
|  |  | F10 |
|  |  | PTGS2 |
|  |  | RXRA |
|  |  | PDE3A |
|  |  | ADRA1B |
|  |  | ADRB2 |
|  |  | ADRA1D |
|  |  | HSP90AA1 |
|  |  | NCOA2 |
| CR24 | Angelicin | PGR |
|  |  | NCOA2 |
|  |  | NR3C2 |
| CR25 | Folacid | F2 |
|  |  | GSK3B |
| CR26 | Oleic acid | PTGS1 |
|  |  | NCOA2 |
|  |  | PTGS2 |
|  |  | LYZ |
|  |  | PRSS3 |
|  |  | RXRA |
|  |  | PLAU |
|  |  | SOD1 |
|  |  | TEP1 |
|  |  | EDN3 |
|  |  | ERBB2 |
|  |  | PPARG |
|  |  | LPL |
|  |  | GAP43 |
|  |  | SERPINE1 |
|  |  | BDNF |
|  |  | HMGCR |
|  |  | MPO |
|  |  | PPARA |
|  |  | PPARD |
|  |  | CRP |
|  |  | PON1 |
|  |  | INS |
|  |  | PLG |
|  |  | FABP1 |
|  |  | RBP2 |
|  |  | ENPEP |
|  |  | UCP2 |
|  |  | SOAT1 |
|  |  | CCK |
|  |  | CITED1 |
|  |  | PDX1 |
|  |  | SLC2A2 |
|  |  | PAM |
|  |  | SCD |
|  |  | UCP3 |
|  |  | CETP |
|  |  | PYY |
|  |  | DNPEP |
| CR27 | Cetostearic acid | CTSD |
|  |  | PTGS1 |
|  |  | PTGS2 |
|  |  | RHO |
|  |  | NCOA2 |
|  |  | BCL2 |
|  |  | IL10 |
|  |  | TNFSF15 |
|  |  | SLC22A5 |
|  |  | PCYT1A |
| CR28 | Octadecanoic acid | PTGS1 |
|  |  | PTGS2 |
|  |  | RXRA |
|  |  | NCOA2 |
|  |  | SP1 |
|  |  | ENPP7 |
| CR29 | Methyl hexadecanoate | PTGS1 |
|  |  | NCOA2 |
|  |  | RELA |
|  |  | IL10 |
|  |  | TNFSF15 |
|  |  | IL6 |
|  |  | PTGS2 |
| CR30 | Isoledene | CHRM2 |
|  |  | CHRM3 |
| CR31 | Ferulic acid | MIF |
|  |  | SHBG |
| CR32 | Clionasterol | AKR1C1 |
|  |  | AKR1C2 |
|  |  | VDR |
|  |  | SULT2B1 |
|  |  | AR |
|  |  | CLEC4E |
|  |  | ESR1 |
|  |  | ESR2 |
|  |  | GABRA1 |
|  |  | GABRA2 |
|  |  | GABRA3 |
|  |  | GABRA4 |
|  |  | GABRA5 |
|  |  | GABRA6 |
|  |  | GABRB1 |
|  |  | GABRB2 |
|  |  | GABRB3 |
|  |  | GABRD |
|  |  | GABRE |
|  |  | GABRG1 |
|  |  | GABRG2 |
|  |  | GABRG3 |
|  |  | GABRP |
|  |  | GABRQ |
|  |  | GRIN1 |
|  |  | GRIN2A |
|  |  | GRIN2B |
|  |  | GRIN2C |
|  |  | GRIN2D |
|  |  | GRIN3A |
|  |  | GRIN3B |
|  |  | HSD17B1 |
|  |  | LIP3 |
|  |  | LSS |
|  |  | NCOA2 |
|  |  | NR1I2 |
|  |  | NR1I3 |
|  |  | NR3C1 |
|  |  | NR3C2 |
|  |  | PGR |
|  |  | PPARA |
|  |  | RORA |
|  |  | SIGMAR1 |
|  |  | SULT2A1 |
| CR33 | Choline | BCHE |
|  |  | ACHE |
| CR34 | Chrysophanic Acid | ALOX5 |
|  |  | AKT1 |
|  |  | APP |
|  |  | PTGS2 |
|  |  | ESR1 |
|  |  | PPARA |
|  |  | SLC2A1 |
|  |  | ITGB3 |
|  |  | PTGS1 |
|  |  | PPARG |
| CR35 | Biocolina | BCHE |
|  |  | ACHE |
| CR36 | Xiongterpene | HSD11B1 |
|  |  | NFKB2 |
|  |  | TNF |
|  |  | NFKB1 |
|  |  | LPL |
|  |  | CASP3 |
| CR37 | Senkyunolide I | PTGIR |
|  |  | PTGIS |
|  |  | P2RY12 |
| CR38 | Retinol | RLBP1 |
|  |  | RBP3 |
