# Supplementary material for: Exploring the Potential Mechanism of Chuanxiong Rhizoma Treatment for Migraine Based on Systems Pharmacology
Source: Evid Based Complement Alternat Med. 2021 Dec 28;2021:2809004. doi: 10.1155/2021/2809004 (PMC8727101; doi:10.1155/2021/2809004)
Supplement: Supplementary Materials — Supplementary Table S1: active compounds of CR). A total of 248 candidate compounds were included. Supplementary Table S2: relevant targets of CR active compounds. Supplementary Table S3: targets for Migraine. Supplementary Table S4: overlapping targets between disease and drug. Supplementary Table S5: details of the active compounds of CR and overlapping targets. Supplementary Table S6: the details of the PPI network. Supplementary Table S7: details of the GO enrichment analysis. Supplementary Table S8: details of the KEGG pathway enrichment analysis. [file 2809004.f1.zip › 2809004.f1/Supplementary Table S3 The targets for Migraine (1).doc]

| Gene Symbol | Description | Category | Gifts | GC Id | Relevance score | GeneCards Link |
| --- | --- | --- | --- | --- | --- | --- |
| ATP1A2 | ATPase Na+/K+ Transporting Subunit Alpha 2 | Protein Coding | 48 | GC01P160115 | 92.49155426 | https://www.genecards.org/cgi-bin/carddisp.pl?gene=ATP1A2 |
| CACNA1A | Calcium Voltage-Gated Channel Subunit Alpha1 A | Protein Coding | 47 | GC19M013206 | 73.929245 | https://www.genecards.org/cgi-bin/carddisp.pl?gene=CACNA1A |
| SCN1A | Sodium Voltage-Gated Channel Alpha Subunit 1 | Protein Coding | 47 | GC02M165989 | 63.40288925 | https://www.genecards.org/cgi-bin/carddisp.pl?gene=SCN1A |
| NOTCH3 | Notch Receptor 3 | Protein Coding | 50 | GC19M015159 | 54.38739777 | https://www.genecards.org/cgi-bin/carddisp.pl?gene=NOTCH3 |
| MGR1 | Migraine With Aura, Susceptibility To | Genetic Locus | 1 | GC04U901855 | 46.53179169 | https://www.genecards.org/cgi-bin/carddisp.pl?gene=MGR1 |
| TNF | Tumor Necrosis Factor | Protein Coding | 51 | GC06P055202 | 45.52923965 | https://www.genecards.org/cgi-bin/carddisp.pl?gene=TNF |
| EDNRA | Endothelin Receptor Type A | Protein Coding | 50 | GC04P147480 | 44.44276428 | https://www.genecards.org/cgi-bin/carddisp.pl?gene=EDNRA |
| ESR1 | Estrogen Receptor 1 | Protein Coding | 53 | GC06P151656 | 44.0993576 | https://www.genecards.org/cgi-bin/carddisp.pl?gene=ESR1 |
| KCNK18 | Potassium Two Pore Domain Channel Subfamily K Member 18 | Protein Coding | 38 | GC10P117197 | 40.93240738 | https://www.genecards.org/cgi-bin/carddisp.pl?gene=KCNK18 |
| LOC102724058 | Uncharacterized LOC102724058 | RNA Gene | 6 | GC02P165958 | 40.57276154 | https://www.genecards.org/cgi-bin/carddisp.pl?gene=LOC102724058 |
| BRCA2 | BRCA2 DNA Repair Associated | Protein Coding | 50 | GC13P032315 | 35.40546417 | https://www.genecards.org/cgi-bin/carddisp.pl?gene=BRCA2 |
| CAPN3 | Calpain 3 | Protein Coding | 48 | GC15P042359 | 35.07770538 | https://www.genecards.org/cgi-bin/carddisp.pl?gene=CAPN3 |
| PPOX | Protoporphyrinogen Oxidase | Protein Coding | 44 | GC01P161184 | 34.46135712 | https://www.genecards.org/cgi-bin/carddisp.pl?gene=PPOX |
| PRRT2 | Proline Rich Transmembrane Protein 2 | Protein Coding | 40 | GC16P029811 | 31.12360001 | https://www.genecards.org/cgi-bin/carddisp.pl?gene=PRRT2 |
| MGR5 | Migraine With Or Without Aura, Susceptibility To, 5 | Genetic Locus | 1 | GC19U900053 | 27.30681992 | https://www.genecards.org/cgi-bin/carddisp.pl?gene=MGR5 |
| MGR6 | Migraine, Several Forms | Genetic Locus | 1 | GC01U904111 | 26.69368362 | https://www.genecards.org/cgi-bin/carddisp.pl?gene=MGR6 |
| MTHFR | Methylenetetrahydrofolate Reductase | Protein Coding | 49 | GC01M011785 | 23.99617577 | https://www.genecards.org/cgi-bin/carddisp.pl?gene=MTHFR |
| MT-TL1 | Mitochondrially Encoded TRNA-Leu (UUA/G) 1 | RNA Gene | 15 | GCMTP003232 | 21.75440598 | https://www.genecards.org/cgi-bin/carddisp.pl?gene=MT-TL1 |
| MGR4 | Migraine, Susceptibility To, 4 | Genetic Locus | 1 | GC14U900003 | 21.65862465 | https://www.genecards.org/cgi-bin/carddisp.pl?gene=MGR4 |
| MGR2 | Migraine, Familial Typical, Susceptibility To | Genetic Locus | 1 | GC0XU900032 | 21.37739754 | https://www.genecards.org/cgi-bin/carddisp.pl?gene=MGR2 |
| ETL4 | Epilepsy, Occipitotemporal Lobe, And Migraine With Aura | Genetic Locus | 2 | GC09U902059 | 20.81025505 | https://www.genecards.org/cgi-bin/carddisp.pl?gene=ETL4 |
| MGR12 | Migraine, With Or Without Aura, Susceptibility To, 12 | Genetic Locus | 1 | GC10U901155 | 20.14535141 | https://www.genecards.org/cgi-bin/carddisp.pl?gene=MGR12 |
| MGR7 | Migraine With Aura, Susceptibility To, 7 | Genetic Locus | 1 | GC15U900492 | 20.01931 | https://www.genecards.org/cgi-bin/carddisp.pl?gene=MGR7 |
| MGR3 | Migraine, Familial, With Or Without Aura, Susceptibility To | Genetic Locus | 1 | GC06U900014 | 19.82473183 | https://www.genecards.org/cgi-bin/carddisp.pl?gene=MGR3 |
| MGR10 | Migraine With Or Without Aura, Susceptibility To, 10 | Genetic Locus | 1 | GC17U902914 | 19.46673775 | https://www.genecards.org/cgi-bin/carddisp.pl?gene=MGR10 |
| SLC1A3 | Solute Carrier Family 1 Member 3 | Protein Coding | 51 | GC05P036620 | 19.14657593 | https://www.genecards.org/cgi-bin/carddisp.pl?gene=SLC1A3 |
| MGR9 | Migraine With Aura, Susceptibility To, 9 | Genetic Locus | 1 | GC11U901393 | 18.87791443 | https://www.genecards.org/cgi-bin/carddisp.pl?gene=MGR9 |
| MGR11 | Migraine With Or Without Aura, Susceptibility To, 11 | Genetic Locus | 1 | GC18U901349 | 18.6617775 | https://www.genecards.org/cgi-bin/carddisp.pl?gene=MGR11 |
| CALCA | Calcitonin Related Polypeptide Alpha | Protein Coding | 43 | GC11M014945 | 18.41447449 | https://www.genecards.org/cgi-bin/carddisp.pl?gene=CALCA |
| MGR8 | Migraine, Susceptibility To, 8 | Genetic Locus | 1 | GC05U901104 | 17.74616814 | https://www.genecards.org/cgi-bin/carddisp.pl?gene=MGR8 |
| SLC6A4 | Solute Carrier Family 6 Member 4 | Protein Coding | 48 | GC17M030194 | 17.52654457 | https://www.genecards.org/cgi-bin/carddisp.pl?gene=SLC6A4 |
| POLG | DNA Polymerase Gamma, Catalytic Subunit | Protein Coding | 46 | GC15M089365 | 17.23581886 | https://www.genecards.org/cgi-bin/carddisp.pl?gene=POLG |
| HTR1A | 5-Hydroxytryptamine Receptor 1A | Protein Coding | 48 | GC05M063960 | 16.76913071 | https://www.genecards.org/cgi-bin/carddisp.pl?gene=HTR1A |
| COL4A1 | Collagen Type IV Alpha 1 Chain | Protein Coding | 49 | GC13M110148 | 16.40789795 | https://www.genecards.org/cgi-bin/carddisp.pl?gene=COL4A1 |
| SLC2A1 | Solute Carrier Family 2 Member 1 | Protein Coding | 53 | GC01M042925 | 16.06446648 | https://www.genecards.org/cgi-bin/carddisp.pl?gene=SLC2A1 |
| HTR1B | 5-Hydroxytryptamine Receptor 1B | Protein Coding | 45 | GC06M077478 | 15.82547665 | https://www.genecards.org/cgi-bin/carddisp.pl?gene=HTR1B |
| ACE | Angiotensin I Converting Enzyme | Protein Coding | 50 | GC17P063477 | 15.80145073 | https://www.genecards.org/cgi-bin/carddisp.pl?gene=ACE |
| HTR1D | 5-Hydroxytryptamine Receptor 1D | Protein Coding | 47 | GC01M023191 | 15.46632195 | https://www.genecards.org/cgi-bin/carddisp.pl?gene=HTR1D |
| KCNA1 | Potassium Voltage-Gated Channel Subfamily A Member 1 | Protein Coding | 46 | GC12P011800 | 15.35053921 | https://www.genecards.org/cgi-bin/carddisp.pl?gene=KCNA1 |
| CSNK1D | Casein Kinase 1 Delta | Protein Coding | 51 | GC17M082239 | 15.12490845 | https://www.genecards.org/cgi-bin/carddisp.pl?gene=CSNK1D |
| DRD2 | Dopamine Receptor D2 | Protein Coding | 51 | GC11M113409 | 14.8064127 | https://www.genecards.org/cgi-bin/carddisp.pl?gene=DRD2 |
| ATP1A3 | ATPase Na+/K+ Transporting Subunit Alpha 3 | Protein Coding | 48 | GC19M041966 | 14.3261404 | https://www.genecards.org/cgi-bin/carddisp.pl?gene=ATP1A3 |
| IL10 | Interleukin 10 | Protein Coding | 48 | GC01M206767 | 14.0554266 | https://www.genecards.org/cgi-bin/carddisp.pl?gene=IL10 |
| NOS3 | Nitric Oxide Synthase 3 | Protein Coding | 52 | GC07P150990 | 13.76813316 | https://www.genecards.org/cgi-bin/carddisp.pl?gene=NOS3 |
| FHL5 | Four And A Half LIM Domains 5 | Protein Coding | 38 | GC06P096562 | 13.57092953 | https://www.genecards.org/cgi-bin/carddisp.pl?gene=FHL5 |
| SCN2A | Sodium Voltage-Gated Channel Alpha Subunit 2 | Protein Coding | 48 | GC02P165194 | 13.50877953 | https://www.genecards.org/cgi-bin/carddisp.pl?gene=SCN2A |
| DBH | Dopamine Beta-Hydroxylase | Protein Coding | 51 | GC09P133636 | 13.44480038 | https://www.genecards.org/cgi-bin/carddisp.pl?gene=DBH |
| HTR2A | 5-Hydroxytryptamine Receptor 2A | Protein Coding | 48 | GC13M046831 | 13.38957596 | https://www.genecards.org/cgi-bin/carddisp.pl?gene=HTR2A |
| MAOA | Monoamine Oxidase A | Protein Coding | 51 | GC0XP043654 | 13.24213409 | https://www.genecards.org/cgi-bin/carddisp.pl?gene=MAOA |
| PRDM16 | PR/SET Domain 16 | Protein Coding | 45 | GC01P003068 | 13.00401306 | https://www.genecards.org/cgi-bin/carddisp.pl?gene=PRDM16 |
| HTR3A | 5-Hydroxytryptamine Receptor 3A | Protein Coding | 47 | GC11P113974 | 12.95221996 | https://www.genecards.org/cgi-bin/carddisp.pl?gene=HTR3A |
| MTDH | Metadherin | Protein Coding | 41 | GC08P097643 | 12.73177433 | https://www.genecards.org/cgi-bin/carddisp.pl?gene=MTDH |
| F2 | Coagulation Factor II, Thrombin | Protein Coding | 49 | GC11P046720 | 12.62685204 | https://www.genecards.org/cgi-bin/carddisp.pl?gene=F2 |
| TRPM8 | Transient Receptor Potential Cation Channel Subfamily M Member 8 | Protein Coding | 44 | GC02P233917 | 12.59396935 | https://www.genecards.org/cgi-bin/carddisp.pl?gene=TRPM8 |
| HTR2C | 5-Hydroxytryptamine Receptor 2C | Protein Coding | 48 | GC0XP114584 | 12.36004448 | https://www.genecards.org/cgi-bin/carddisp.pl?gene=HTR2C |
| CLCN1 | Chloride Voltage-Gated Channel 1 | Protein Coding | 45 | GC07P143316 | 12.31184196 | https://www.genecards.org/cgi-bin/carddisp.pl?gene=CLCN1 |
| PRL | Prolactin | Protein Coding | 44 | GC06M022287 | 12.28409863 | https://www.genecards.org/cgi-bin/carddisp.pl?gene=PRL |
| SLC6A3 | Solute Carrier Family 6 Member 3 | Protein Coding | 50 | GC05M001392 | 12.24495125 | https://www.genecards.org/cgi-bin/carddisp.pl?gene=SLC6A3 |
| SCN9A | Sodium Voltage-Gated Channel Alpha Subunit 9 | Protein Coding | 48 | GC02M166195 | 12.08476067 | https://www.genecards.org/cgi-bin/carddisp.pl?gene=SCN9A |
| CPQ | Carboxypeptidase Q | Protein Coding | 34 | GC08P096645 | 11.99871445 | https://www.genecards.org/cgi-bin/carddisp.pl?gene=CPQ |
| DRD3 | Dopamine Receptor D3 | Protein Coding | 45 | GC03M114128 | 11.97289753 | https://www.genecards.org/cgi-bin/carddisp.pl?gene=DRD3 |
| ASTN2 | Astrotactin 2 | Protein Coding | 37 | GC09M116425 | 11.93460846 | https://www.genecards.org/cgi-bin/carddisp.pl?gene=ASTN2 |
| COMT | Catechol-O-Methyltransferase | Protein Coding | 52 | GC22P019941 | 11.84978008 | https://www.genecards.org/cgi-bin/carddisp.pl?gene=COMT |
| CACNA1E | Calcium Voltage-Gated Channel Subunit Alpha1 E | Protein Coding | 47 | GC01P181317 | 11.70076847 | https://www.genecards.org/cgi-bin/carddisp.pl?gene=CACNA1E |
| DRD5 | Dopamine Receptor D5 | Protein Coding | 48 | GC04P009783 | 11.625453 | https://www.genecards.org/cgi-bin/carddisp.pl?gene=DRD5 |
| IL1B | Interleukin 1 Beta | Protein Coding | 49 | GC02M112829 | 11.5722208 | https://www.genecards.org/cgi-bin/carddisp.pl?gene=IL1B |
| BDNF | Brain Derived Neurotrophic Factor | Protein Coding | 48 | GC11M027654 | 11.56353188 | https://www.genecards.org/cgi-bin/carddisp.pl?gene=BDNF |
| ALPK1 | Alpha Kinase 1 | Protein Coding | 40 | GC04P112285 | 11.50516891 | https://www.genecards.org/cgi-bin/carddisp.pl?gene=ALPK1 |
| EDN1 | Endothelin 1 | Protein Coding | 48 | GC06P012256 | 11.43387127 | https://www.genecards.org/cgi-bin/carddisp.pl?gene=EDN1 |
| EDNRB | Endothelin Receptor Type B | Protein Coding | 50 | GC13M077895 | 11.37123489 | https://www.genecards.org/cgi-bin/carddisp.pl?gene=EDNRB |
| DRD4 | Dopamine Receptor D4 | Protein Coding | 47 | GC11P000979 | 11.37123489 | https://www.genecards.org/cgi-bin/carddisp.pl?gene=DRD4 |
| KCNQ2 | Potassium Voltage-Gated Channel Subfamily Q Member 2 | Protein Coding | 49 | GC20M063400 | 11.360425 | https://www.genecards.org/cgi-bin/carddisp.pl?gene=KCNQ2 |
| OPRM1 | Opioid Receptor Mu 1 | Protein Coding | 49 | GC06P154075 | 11.35661983 | https://www.genecards.org/cgi-bin/carddisp.pl?gene=OPRM1 |
| IL6 | Interleukin 6 | Protein Coding | 50 | GC07P022725 | 11.27690125 | https://www.genecards.org/cgi-bin/carddisp.pl?gene=IL6 |
| TSPAN2 | Tetraspanin 2 | Protein Coding | 38 | GC01M115050 | 11.27461433 | https://www.genecards.org/cgi-bin/carddisp.pl?gene=TSPAN2 |
| KCNQ3 | Potassium Voltage-Gated Channel Subfamily Q Member 3 | Protein Coding | 46 | GC08M132120 | 11.22017574 | https://www.genecards.org/cgi-bin/carddisp.pl?gene=KCNQ3 |
| VIP | Vasoactive Intestinal Peptide | Protein Coding | 45 | GC06P152750 | 11.2187748 | https://www.genecards.org/cgi-bin/carddisp.pl?gene=VIP |
| FLNA | Filamin A | Protein Coding | 50 | GC0XM154348 | 11.20023346 | https://www.genecards.org/cgi-bin/carddisp.pl?gene=FLNA |
| HTR1F | 5-Hydroxytryptamine Receptor 1F | Protein Coding | 45 | GC03P087792 | 11.156353 | https://www.genecards.org/cgi-bin/carddisp.pl?gene=HTR1F |
| MEF2D | Myocyte Enhancer Factor 2D | Protein Coding | 45 | GC01M156463 | 10.87820435 | https://www.genecards.org/cgi-bin/carddisp.pl?gene=MEF2D |
| DRD1 | Dopamine Receptor D1 | Protein Coding | 45 | GC05M175440 | 10.79097366 | https://www.genecards.org/cgi-bin/carddisp.pl?gene=DRD1 |
| PHACTR1 | Phosphatase And Actin Regulator 1 | Protein Coding | 38 | GC06P012717 | 10.62249374 | https://www.genecards.org/cgi-bin/carddisp.pl?gene=PHACTR1 |
| CNR1 | Cannabinoid Receptor 1 | Protein Coding | 47 | GC06M088139 | 10.62039471 | https://www.genecards.org/cgi-bin/carddisp.pl?gene=CNR1 |
| CACNA1B | Calcium Voltage-Gated Channel Subunit Alpha1 B | Protein Coding | 50 | GC09P137877 | 10.59910202 | https://www.genecards.org/cgi-bin/carddisp.pl?gene=CACNA1B |
| ADRB2 | Adrenoceptor Beta 2 | Protein Coding | 50 | GC05P148825 | 10.59348392 | https://www.genecards.org/cgi-bin/carddisp.pl?gene=ADRB2 |
| CRP | C-Reactive Protein | Protein Coding | 47 | GC01M159719 | 10.56958866 | https://www.genecards.org/cgi-bin/carddisp.pl?gene=CRP |
| PTGS2 | Prostaglandin-Endoperoxide Synthase 2 | Protein Coding | 49 | GC01M186640 | 10.48402405 | https://www.genecards.org/cgi-bin/carddisp.pl?gene=PTGS2 |
| MAOB | Monoamine Oxidase B | Protein Coding | 44 | GC0XM043766 | 10.43434525 | https://www.genecards.org/cgi-bin/carddisp.pl?gene=MAOB |
| SCN8A | Sodium Voltage-Gated Channel Alpha Subunit 8 | Protein Coding | 47 | GC12P051590 | 10.35530567 | https://www.genecards.org/cgi-bin/carddisp.pl?gene=SCN8A |
| RAMP1 | Receptor Activity Modifying Protein 1 | Protein Coding | 44 | GC02P237858 | 10.3439436 | https://www.genecards.org/cgi-bin/carddisp.pl?gene=RAMP1 |
| SCN5A | Sodium Voltage-Gated Channel Alpha Subunit 5 | Protein Coding | 51 | GC03M038549 | 10.33272648 | https://www.genecards.org/cgi-bin/carddisp.pl?gene=SCN5A |
| HCRTR2 | Hypocretin Receptor 2 | Protein Coding | 45 | GC06P055106 | 10.32608128 | https://www.genecards.org/cgi-bin/carddisp.pl?gene=HCRTR2 |
| HTR2B | 5-Hydroxytryptamine Receptor 2B | Protein Coding | 44 | GC02M231108 | 10.31333351 | https://www.genecards.org/cgi-bin/carddisp.pl?gene=HTR2B |
| NOS1 | Nitric Oxide Synthase 1 | Protein Coding | 50 | GC12M117208 | 10.31066227 | https://www.genecards.org/cgi-bin/carddisp.pl?gene=NOS1 |
| TAC1 | Tachykinin Precursor 1 | Protein Coding | 44 | GC07P097731 | 10.25264549 | https://www.genecards.org/cgi-bin/carddisp.pl?gene=TAC1 |
| NCS1 | Neuronal Calcium Sensor 1 | Protein Coding | 43 | GC09P130172 | 10.25099468 | https://www.genecards.org/cgi-bin/carddisp.pl?gene=NCS1 |
| TSPAN16 | Tetraspanin 16 | Protein Coding | 31 | GC19P011296 | 10.25099468 | https://www.genecards.org/cgi-bin/carddisp.pl?gene=TSPAN16 |
| CACNA1S | Calcium Voltage-Gated Channel Subunit Alpha1 S | Protein Coding | 49 | GC01M201008 | 10.17899704 | https://www.genecards.org/cgi-bin/carddisp.pl?gene=CACNA1S |
| ADCYAP1 | Adenylate Cyclase Activating Polypeptide 1 | Protein Coding | 43 | GC18P000895 | 10.0172224 | https://www.genecards.org/cgi-bin/carddisp.pl?gene=ADCYAP1 |
| GRIA1 | Glutamate Ionotropic Receptor AMPA Type Subunit 1 | Protein Coding | 50 | GC05P153467 | 10.01546955 | https://www.genecards.org/cgi-bin/carddisp.pl?gene=GRIA1 |
| TPH1 | Tryptophan Hydroxylase 1 | Protein Coding | 45 | GC11M018040 | 10.01146412 | https://www.genecards.org/cgi-bin/carddisp.pl?gene=TPH1 |
| GABRQ | Gamma-Aminobutyric Acid Type A Receptor Subunit Theta | Protein Coding | 38 | GC0XP152638 | 9.989102364 | https://www.genecards.org/cgi-bin/carddisp.pl?gene=GABRQ |
| CYP2D6 | Cytochrome P450 Family 2 Subfamily D Member 6 | Protein Coding | 49 | GC22M042126 | 9.971289635 | https://www.genecards.org/cgi-bin/carddisp.pl?gene=CYP2D6 |
| NGF | Nerve Growth Factor | Protein Coding | 51 | GC01M115285 | 9.936612129 | https://www.genecards.org/cgi-bin/carddisp.pl?gene=NGF |
| HTR7 | 5-Hydroxytryptamine Receptor 7 | Protein Coding | 48 | GC10M090740 | 9.892651558 | https://www.genecards.org/cgi-bin/carddisp.pl?gene=HTR7 |
| APOH | Apolipoprotein H | Protein Coding | 44 | GC17M066212 | 9.892503738 | https://www.genecards.org/cgi-bin/carddisp.pl?gene=APOH |
| SLC1A2 | Solute Carrier Family 1 Member 2 | Protein Coding | 50 | GC11M035252 | 9.829494476 | https://www.genecards.org/cgi-bin/carddisp.pl?gene=SLC1A2 |
| TPM3 | Tropomyosin 3 | Protein Coding | 47 | GC01M154127 | 9.769789696 | https://www.genecards.org/cgi-bin/carddisp.pl?gene=TPM3 |
| ADORA2A | Adenosine A2a Receptor | Protein Coding | 46 | GC22P024417 | 9.73890686 | https://www.genecards.org/cgi-bin/carddisp.pl?gene=ADORA2A |
| LEP | Leptin | Protein Coding | 48 | GC07P128241 | 9.687818527 | https://www.genecards.org/cgi-bin/carddisp.pl?gene=LEP |
| KNG1 | Kininogen 1 | Protein Coding | 45 | GC03P186717 | 9.673669815 | https://www.genecards.org/cgi-bin/carddisp.pl?gene=KNG1 |
| PDE5A | Phosphodiesterase 5A | Protein Coding | 45 | GC04M119494 | 9.67276001 | https://www.genecards.org/cgi-bin/carddisp.pl?gene=PDE5A |
| P2RX3 | Purinergic Receptor P2X 3 | Protein Coding | 43 | GC11P057356 | 9.668503761 | https://www.genecards.org/cgi-bin/carddisp.pl?gene=P2RX3 |
| TWNK | Twinkle MtDNA Helicase | Protein Coding | 34 | GC10P100993 | 9.607898712 | https://www.genecards.org/cgi-bin/carddisp.pl?gene=TWNK |
| TRPV1 | Transient Receptor Potential Cation Channel Subfamily V Member 1 | Protein Coding | 47 | GC17M003565 | 9.585669518 | https://www.genecards.org/cgi-bin/carddisp.pl?gene=TRPV1 |
| CYP3A4 | Cytochrome P450 Family 3 Subfamily A Member 4 | Protein Coding | 51 | GC07M099759 | 9.546943665 | https://www.genecards.org/cgi-bin/carddisp.pl?gene=CYP3A4 |
| SLC20A2 | Solute Carrier Family 20 Member 2 | Protein Coding | 47 | GC08M042416 | 9.504405022 | https://www.genecards.org/cgi-bin/carddisp.pl?gene=SLC20A2 |
| POMC | Proopiomelanocortin | Protein Coding | 48 | GC02M025160 | 9.482848167 | https://www.genecards.org/cgi-bin/carddisp.pl?gene=POMC |
| ADM | Adrenomedullin | Protein Coding | 45 | GC11P010304 | 9.458148003 | https://www.genecards.org/cgi-bin/carddisp.pl?gene=ADM |
| TREX1 | Three Prime Repair Exonuclease 1 | Protein Coding | 43 | GC03P048466 | 9.447630882 | https://www.genecards.org/cgi-bin/carddisp.pl?gene=TREX1 |
| CACNA1H | Calcium Voltage-Gated Channel Subunit Alpha1 H | Protein Coding | 51 | GC16P001153 | 9.426517487 | https://www.genecards.org/cgi-bin/carddisp.pl?gene=CACNA1H |
| GSTM1 | Glutathione S-Transferase Mu 1 | Protein Coding | 43 | GC01P109687 | 9.38927269 | https://www.genecards.org/cgi-bin/carddisp.pl?gene=GSTM1 |
| CACNA1C | Calcium Voltage-Gated Channel Subunit Alpha1 C | Protein Coding | 50 | GC12P001970 | 9.361409187 | https://www.genecards.org/cgi-bin/carddisp.pl?gene=CACNA1C |
| GRIN2B | Glutamate Ionotropic Receptor NMDA Type Subunit 2B | Protein Coding | 51 | GC12M013437 | 9.300731659 | https://www.genecards.org/cgi-bin/carddisp.pl?gene=GRIN2B |
| PNOC | Prepronociceptin | Protein Coding | 38 | GC08P028316 | 9.289979935 | https://www.genecards.org/cgi-bin/carddisp.pl?gene=PNOC |
| S100B | S100 Calcium Binding Protein B | Protein Coding | 46 | GC21M048326 | 9.22451973 | https://www.genecards.org/cgi-bin/carddisp.pl?gene=S100B |
| CACNB4 | Calcium Voltage-Gated Channel Auxiliary Subunit Beta 4 | Protein Coding | 47 | GC02M151832 | 9.17100811 | https://www.genecards.org/cgi-bin/carddisp.pl?gene=CACNB4 |
| HTR1E | 5-Hydroxytryptamine Receptor 1E | Protein Coding | 41 | GC06P086937 | 9.153837204 | https://www.genecards.org/cgi-bin/carddisp.pl?gene=HTR1E |
| SCN3A | Sodium Voltage-Gated Channel Alpha Subunit 3 | Protein Coding | 48 | GC02M165087 | 9.145523071 | https://www.genecards.org/cgi-bin/carddisp.pl?gene=SCN3A |
| SCN4A | Sodium Voltage-Gated Channel Alpha Subunit 4 | Protein Coding | 45 | GC17M063938 | 9.13815403 | https://www.genecards.org/cgi-bin/carddisp.pl?gene=SCN4A |
| PPBP | Pro-Platelet Basic Protein | Protein Coding | 44 | GC04M073986 | 9.13807106 | https://www.genecards.org/cgi-bin/carddisp.pl?gene=PPBP |
| ALB | Albumin | Protein Coding | 50 | GC04P073397 | 9.13758564 | https://www.genecards.org/cgi-bin/carddisp.pl?gene=ALB |
| HCRT | Hypocretin Neuropeptide Precursor | Protein Coding | 41 | GC17M042185 | 9.119826317 | https://www.genecards.org/cgi-bin/carddisp.pl?gene=HCRT |
| KCNK2 | Potassium Two Pore Domain Channel Subfamily K Member 2 | Protein Coding | 40 | GC01P215005 | 9.083436966 | https://www.genecards.org/cgi-bin/carddisp.pl?gene=KCNK2 |
| PEX11B | Peroxisomal Biogenesis Factor 11 Beta | Protein Coding | 41 | GC01M145911 | 9.074781418 | https://www.genecards.org/cgi-bin/carddisp.pl?gene=PEX11B |
| CAV2 | Caveolin 2 | Protein Coding | 43 | GC07P116287 | 9.020904541 | https://www.genecards.org/cgi-bin/carddisp.pl?gene=CAV2 |
| KCNK5 | Potassium Two Pore Domain Channel Subfamily K Member 5 | Protein Coding | 38 | GC06M047054 | 9.014845848 | https://www.genecards.org/cgi-bin/carddisp.pl?gene=KCNK5 |
| NPY | Neuropeptide Y | Protein Coding | 46 | GC07P024290 | 8.937292099 | https://www.genecards.org/cgi-bin/carddisp.pl?gene=NPY |
| STIM1 | Stromal Interaction Molecule 1 | Protein Coding | 49 | GC11P003855 | 8.89848423 | https://www.genecards.org/cgi-bin/carddisp.pl?gene=STIM1 |
| TAB2 | TGF-Beta Activated Kinase 1 (MAP3K7) Binding Protein 2 | Protein Coding | 48 | GC06P149218 | 8.88909626 | https://www.genecards.org/cgi-bin/carddisp.pl?gene=TAB2 |
| CALCRL | Calcitonin Receptor Like Receptor | Protein Coding | 45 | GC02M187341 | 8.868146896 | https://www.genecards.org/cgi-bin/carddisp.pl?gene=CALCRL |
| SLC4A4 | Solute Carrier Family 4 Member 4 | Protein Coding | 47 | GC04P071063 | 8.849839211 | https://www.genecards.org/cgi-bin/carddisp.pl?gene=SLC4A4 |
| CRH | Corticotropin Releasing Hormone | Protein Coding | 44 | GC08M066176 | 8.790266991 | https://www.genecards.org/cgi-bin/carddisp.pl?gene=CRH |
| TACR1 | Tachykinin Receptor 1 | Protein Coding | 47 | GC02M075010 | 8.73157692 | https://www.genecards.org/cgi-bin/carddisp.pl?gene=TACR1 |
| TRPV3 | Transient Receptor Potential Cation Channel Subfamily V Member 3 | Protein Coding | 44 | GC17M003515 | 8.680260658 | https://www.genecards.org/cgi-bin/carddisp.pl?gene=TRPV3 |
| FMR1 | FMRP Translational Regulator 1 | Protein Coding | 45 | GC0XP147925 | 8.677467346 | https://www.genecards.org/cgi-bin/carddisp.pl?gene=FMR1 |
| HRH3 | Histamine Receptor H3 | Protein Coding | 43 | GC20M062214 | 8.652780533 | https://www.genecards.org/cgi-bin/carddisp.pl?gene=HRH3 |
| CCK | Cholecystokinin | Protein Coding | 43 | GC03M042274 | 8.425419807 | https://www.genecards.org/cgi-bin/carddisp.pl?gene=CCK |
| SCN11A | Sodium Voltage-Gated Channel Alpha Subunit 11 | Protein Coding | 42 | GC03M038862 | 8.416870117 | https://www.genecards.org/cgi-bin/carddisp.pl?gene=SCN11A |
| PPIG | Peptidylprolyl Isomerase G | Protein Coding | 43 | GC02P169584 | 8.410837173 | https://www.genecards.org/cgi-bin/carddisp.pl?gene=PPIG |
| ADORA1 | Adenosine A1 Receptor | Protein Coding | 48 | GC01P203090 | 8.375402451 | https://www.genecards.org/cgi-bin/carddisp.pl?gene=ADORA1 |
| ENG | Endoglin | Protein Coding | 47 | GC09M127815 | 8.330716133 | https://www.genecards.org/cgi-bin/carddisp.pl?gene=ENG |
| SNAP25 | Synaptosome Associated Protein 25 | Protein Coding | 51 | GC20P010218 | 8.166046143 | https://www.genecards.org/cgi-bin/carddisp.pl?gene=SNAP25 |
| F5 | Coagulation Factor V | Protein Coding | 45 | GC01M169511 | 8.077937126 | https://www.genecards.org/cgi-bin/carddisp.pl?gene=F5 |
| SV2A | Synaptic Vesicle Glycoprotein 2A | Protein Coding | 44 | GC01M149903 | 8.003930092 | https://www.genecards.org/cgi-bin/carddisp.pl?gene=SV2A |
| ARHGEF16 | Rho Guanine Nucleotide Exchange Factor 16 | Protein Coding | 40 | GC01P003454 | 7.964691162 | https://www.genecards.org/cgi-bin/carddisp.pl?gene=ARHGEF16 |
| SLC24A3 | Solute Carrier Family 24 Member 3 | Protein Coding | 40 | GC20P019212 | 7.964691162 | https://www.genecards.org/cgi-bin/carddisp.pl?gene=SLC24A3 |
| ASIC3 | Acid Sensing Ion Channel Subunit 3 | Protein Coding | 40 | GC07P151048 | 7.962859631 | https://www.genecards.org/cgi-bin/carddisp.pl?gene=ASIC3 |
| RAMP2 | Receptor Activity Modifying Protein 2 | Protein Coding | 42 | GC17P042758 | 7.957805157 | https://www.genecards.org/cgi-bin/carddisp.pl?gene=RAMP2 |
| IAPP | Islet Amyloid Polypeptide | Protein Coding | 42 | GC12P021354 | 7.927782536 | https://www.genecards.org/cgi-bin/carddisp.pl?gene=IAPP |
| OPN4 | Opsin 4 | Protein Coding | 41 | GC10P086654 | 7.910829544 | https://www.genecards.org/cgi-bin/carddisp.pl?gene=OPN4 |
| MT-TQ | Mitochondrially Encoded TRNA-Gln (CAA/G) | RNA Gene | 12 | GCMTM004331 | 7.909286022 | https://www.genecards.org/cgi-bin/carddisp.pl?gene=MT-TQ |
| ADM2 | Adrenomedullin 2 | Protein Coding | 34 | GC22P050481 | 7.893705368 | https://www.genecards.org/cgi-bin/carddisp.pl?gene=ADM2 |
| OXT | Oxytocin/Neurophysin I Prepropeptide | Protein Coding | 41 | GC20P003068 | 7.835682392 | https://www.genecards.org/cgi-bin/carddisp.pl?gene=OXT |
| INS | Insulin | Protein Coding | 48 | GC11M002159 | 7.71086216 | https://www.genecards.org/cgi-bin/carddisp.pl?gene=INS |
| AMACR | Alpha-Methylacyl-CoA Racemase | Protein Coding | 46 | GC05M033986 | 7.698304653 | https://www.genecards.org/cgi-bin/carddisp.pl?gene=AMACR |
| FOS | Fos Proto-Oncogene, AP-1 Transcription Factor Subunit | Protein Coding | 51 | GC14P075278 | 7.655459404 | https://www.genecards.org/cgi-bin/carddisp.pl?gene=FOS |
| H2AC18 | H2A Clustered Histone 18 | Protein Coding | 26 | GC01M150124 | 7.628994465 | https://www.genecards.org/cgi-bin/carddisp.pl?gene=H2AC18 |
| LOC110806262 | Solute Carrier Family 6 Member 4 Gene Promoter | Biological Region | 1 | GC17P030235 | 7.586698055 | https://www.genecards.org/cgi-bin/carddisp.pl?gene=LOC110806262 |
| CALCR | Calcitonin Receptor | Protein Coding | 48 | GC07M093424 | 7.580960751 | https://www.genecards.org/cgi-bin/carddisp.pl?gene=CALCR |
| RAMP3 | Receptor Activity Modifying Protein 3 | Protein Coding | 41 | GC07P045163 | 7.580960751 | https://www.genecards.org/cgi-bin/carddisp.pl?gene=RAMP3 |
| CALCB | Calcitonin Related Polypeptide Beta | Protein Coding | 38 | GC11P014904 | 7.580960751 | https://www.genecards.org/cgi-bin/carddisp.pl?gene=CALCB |
| REN | Renin | Protein Coding | 48 | GC01M204154 | 7.553023815 | https://www.genecards.org/cgi-bin/carddisp.pl?gene=REN |
| SCN10A | Sodium Voltage-Gated Channel Alpha Subunit 10 | Protein Coding | 47 | GC03M038713 | 7.528282166 | https://www.genecards.org/cgi-bin/carddisp.pl?gene=SCN10A |
| TRPA1 | Transient Receptor Potential Cation Channel Subfamily A Member 1 | Protein Coding | 46 | GC08M072019 | 7.528282166 | https://www.genecards.org/cgi-bin/carddisp.pl?gene=TRPA1 |
| GRIK1 | Glutamate Ionotropic Receptor Kainate Type Subunit 1 | Protein Coding | 45 | GC21M029536 | 7.528282166 | https://www.genecards.org/cgi-bin/carddisp.pl?gene=GRIK1 |
| ATP12A | ATPase H+/K+ Transporting Non-Gastric Alpha2 Subunit | Protein Coding | 44 | GC13P024680 | 7.4258461 | https://www.genecards.org/cgi-bin/carddisp.pl?gene=ATP12A |
| ATP4A | ATPase H+/K+ Transporting Subunit Alpha | Protein Coding | 42 | GC19M047080 | 7.4258461 | https://www.genecards.org/cgi-bin/carddisp.pl?gene=ATP4A |
| SCT | Secretin | Protein Coding | 35 | GC11M000626 | 7.4258461 | https://www.genecards.org/cgi-bin/carddisp.pl?gene=SCT |
| TRPV4 | Transient Receptor Potential Cation Channel Subfamily V Member 4 | Protein Coding | 50 | GC12M109783 | 7.40110445 | https://www.genecards.org/cgi-bin/carddisp.pl?gene=TRPV4 |
| PLG | Plasminogen | Protein Coding | 49 | GC06P160702 | 7.40110445 | https://www.genecards.org/cgi-bin/carddisp.pl?gene=PLG |
| GRM5 | Glutamate Metabotropic Receptor 5 | Protein Coding | 48 | GC11M088504 | 7.40110445 | https://www.genecards.org/cgi-bin/carddisp.pl?gene=GRM5 |
| FAAH | Fatty Acid Amide Hydrolase | Protein Coding | 48 | GC01P046394 | 7.40110445 | https://www.genecards.org/cgi-bin/carddisp.pl?gene=FAAH |
| KCNK9 | Potassium Two Pore Domain Channel Subfamily K Member 9 | Protein Coding | 47 | GC08M139585 | 7.40110445 | https://www.genecards.org/cgi-bin/carddisp.pl?gene=KCNK9 |
| VIPR1 | Vasoactive Intestinal Peptide Receptor 1 | Protein Coding | 47 | GC03P042490 | 7.40110445 | https://www.genecards.org/cgi-bin/carddisp.pl?gene=VIPR1 |
| ADCYAP1R1 | ADCYAP Receptor Type I | Protein Coding | 46 | GC07P031058 | 7.40110445 | https://www.genecards.org/cgi-bin/carddisp.pl?gene=ADCYAP1R1 |
| VIPR2 | Vasoactive Intestinal Peptide Receptor 2 | Protein Coding | 45 | GC07M159028 | 7.40110445 | https://www.genecards.org/cgi-bin/carddisp.pl?gene=VIPR2 |
| P2RX2 | Purinergic Receptor P2X 2 | Protein Coding | 44 | GC12P132618 | 7.40110445 | https://www.genecards.org/cgi-bin/carddisp.pl?gene=P2RX2 |
| PANX1 | Pannexin 1 | Protein Coding | 44 | GC11P094128 | 7.40110445 | https://www.genecards.org/cgi-bin/carddisp.pl?gene=PANX1 |
| PDYN | Prodynorphin | Protein Coding | 44 | GC20M001978 | 7.40110445 | https://www.genecards.org/cgi-bin/carddisp.pl?gene=PDYN |
| P2RX4 | Purinergic Receptor P2X 4 | Protein Coding | 44 | GC12P123577 | 7.40110445 | https://www.genecards.org/cgi-bin/carddisp.pl?gene=P2RX4 |
| ANK1 | Ankyrin 1 | Protein Coding | 43 | GC08M041653 | 7.40110445 | https://www.genecards.org/cgi-bin/carddisp.pl?gene=ANK1 |
| KCNK4 | Potassium Two Pore Domain Channel Subfamily K Member 4 | Protein Coding | 42 | GC11P064292 | 7.40110445 | https://www.genecards.org/cgi-bin/carddisp.pl?gene=KCNK4 |
| KCNK10 | Potassium Two Pore Domain Channel Subfamily K Member 10 | Protein Coding | 40 | GC14M088180 | 7.40110445 | https://www.genecards.org/cgi-bin/carddisp.pl?gene=KCNK10 |
| KCNS1 | Potassium Voltage-Gated Channel Modifier Subfamily S Member 1 | Protein Coding | 40 | GC20M045099 | 7.40110445 | https://www.genecards.org/cgi-bin/carddisp.pl?gene=KCNS1 |
| LAPTM4A | Lysosomal Protein Transmembrane 4 Alpha | Protein Coding | 38 | GC02M020032 | 7.40110445 | https://www.genecards.org/cgi-bin/carddisp.pl?gene=LAPTM4A |
| LIPN | Lipase Family Member N | Protein Coding | 37 | GC10P088760 | 7.40110445 | https://www.genecards.org/cgi-bin/carddisp.pl?gene=LIPN |
| MTRNR2L5 | MT-RNR2 Like 5 | Protein Coding | 20 | GC10P055618 | 7.40110445 | https://www.genecards.org/cgi-bin/carddisp.pl?gene=MTRNR2L5 |
| MT-ND4 | Mitochondrially Encoded NADH:Ubiquinone Oxidoreductase Core Subunit 4 | Protein Coding | 33 | GCMTP010762 | 7.085105896 | https://www.genecards.org/cgi-bin/carddisp.pl?gene=MT-ND4 |
| PDGFRB | Platelet Derived Growth Factor Receptor Beta | Protein Coding | 55 | GC05M150113 | 7.059975147 | https://www.genecards.org/cgi-bin/carddisp.pl?gene=PDGFRB |
| ATRIP | ATR Interacting Protein | Protein Coding | 43 | GC03P048449 | 6.972230911 | https://www.genecards.org/cgi-bin/carddisp.pl?gene=ATRIP |
| ATRIP-TREX1 | ATRIP-TREX1 Readthrough | RNA Gene | 5 | GC03P048450 | 6.972230911 | https://www.genecards.org/cgi-bin/carddisp.pl?gene=ATRIP-TREX1 |
| MT-ND5 | Mitochondrially Encoded NADH:Ubiquinone Oxidoreductase Core Subunit 5 | Protein Coding | 33 | GCMTP012339 | 6.950043201 | https://www.genecards.org/cgi-bin/carddisp.pl?gene=MT-ND5 |
| MPEG1 | Macrophage Expressed 1 | Protein Coding | 34 | GC11M059208 | 6.885075092 | https://www.genecards.org/cgi-bin/carddisp.pl?gene=MPEG1 |
| MT-ND1 | Mitochondrially Encoded NADH:Ubiquinone Oxidoreductase Core Subunit 1 | Protein Coding | 33 | GCMTP003309 | 6.845521927 | https://www.genecards.org/cgi-bin/carddisp.pl?gene=MT-ND1 |
| MT-TF | Mitochondrially Encoded TRNA-Phe (UUU/C) | RNA Gene | 14 | GCMTP000003 | 6.845521927 | https://www.genecards.org/cgi-bin/carddisp.pl?gene=MT-TF |
| MT-CO1 | Mitochondrially Encoded Cytochrome C Oxidase I | Protein Coding | 34 | GCMTP005906 | 6.842588902 | https://www.genecards.org/cgi-bin/carddisp.pl?gene=MT-CO1 |
| TGFBR2 | Transforming Growth Factor Beta Receptor 2 | Protein Coding | 51 | GC03P030623 | 6.832559109 | https://www.genecards.org/cgi-bin/carddisp.pl?gene=TGFBR2 |
| MT-TS1 | Mitochondrially Encoded TRNA-Ser (UCN) 1 | RNA Gene | 15 | GCMTM005917 | 6.768115997 | https://www.genecards.org/cgi-bin/carddisp.pl?gene=MT-TS1 |
| MT-TK | Mitochondrially Encoded TRNA-Lys (AAA/G) | RNA Gene | 14 | GCMTP008297 | 6.495792866 | https://www.genecards.org/cgi-bin/carddisp.pl?gene=MT-TK |
| PRKCSH | Protein Kinase C Substrate 80K-H | Protein Coding | 44 | GC19P011435 | 6.46983242 | https://www.genecards.org/cgi-bin/carddisp.pl?gene=PRKCSH |
| LOC102723566 | Uncharacterized LOC102723566 | RNA Gene | 11 | GC09P127816 | 6.464584351 | https://www.genecards.org/cgi-bin/carddisp.pl?gene=LOC102723566 |
| MT-ND6 | Mitochondrially Encoded NADH:Ubiquinone Oxidoreductase Core Subunit 6 | Protein Coding | 33 | GCMTM014151 | 6.461716175 | https://www.genecards.org/cgi-bin/carddisp.pl?gene=MT-ND6 |
| MT-TV | Mitochondrially Encoded TRNA-Val (GUN) | RNA Gene | 14 | GCMTP001605 | 6.461716175 | https://www.genecards.org/cgi-bin/carddisp.pl?gene=MT-TV |
| MT-TW | Mitochondrially Encoded TRNA-Trp (UGA/G) | RNA Gene | 13 | GCMTP005514 | 6.421294212 | https://www.genecards.org/cgi-bin/carddisp.pl?gene=MT-TW |
| LOC108663985 | Calcium Voltage-Gated Channel Subunit Alpha1 A Repeat Instability Region | Biological Region | 2 | GC19P013210 | 6.253407001 | https://www.genecards.org/cgi-bin/carddisp.pl?gene=LOC108663985 |
| PDGFB | Platelet Derived Growth Factor Subunit B | Protein Coding | 50 | GC22M049027 | 6.241437912 | https://www.genecards.org/cgi-bin/carddisp.pl?gene=PDGFB |
| PGK1 | Phosphoglycerate Kinase 1 | Protein Coding | 49 | GC0XP077944 | 6.241437912 | https://www.genecards.org/cgi-bin/carddisp.pl?gene=PGK1 |
| MT-TH | Mitochondrially Encoded TRNA-His (CAU/C) | RNA Gene | 13 | GCMTP012140 | 6.241437912 | https://www.genecards.org/cgi-bin/carddisp.pl?gene=MT-TH |
| TLR4 | Toll Like Receptor 4 | Protein Coding | 51 | GC09P117704 | 6.078215599 | https://www.genecards.org/cgi-bin/carddisp.pl?gene=TLR4 |
| SUGCT | Succinyl-CoA:Glutarate-CoA Transferase | Protein Coding | 37 | GC07P040134 | 6.027004242 | https://www.genecards.org/cgi-bin/carddisp.pl?gene=SUGCT |
| APP | Amyloid Beta Precursor Protein | Protein Coding | 52 | GC21M025880 | 6.007852077 | https://www.genecards.org/cgi-bin/carddisp.pl?gene=APP |
| NAXE | NAD(P)HX Epimerase | Protein Coding | 35 | GC01P156591 | 5.902402401 | https://www.genecards.org/cgi-bin/carddisp.pl?gene=NAXE |
| SLC6A1 | Solute Carrier Family 6 Member 1 | Protein Coding | 48 | GC03P011049 | 5.88776207 | https://www.genecards.org/cgi-bin/carddisp.pl?gene=SLC6A1 |
| ATP1B1 | ATPase Na+/K+ Transporting Subunit Beta 1 | Protein Coding | 48 | GC01P169105 | 5.789009094 | https://www.genecards.org/cgi-bin/carddisp.pl?gene=ATP1B1 |
| DGUOK | Deoxyguanosine Kinase | Protein Coding | 45 | GC02P073926 | 5.74362421 | https://www.genecards.org/cgi-bin/carddisp.pl?gene=DGUOK |
| MT-ATP6 | Mitochondrially Encoded ATP Synthase Membrane Subunit 6 | Protein Coding | 33 | GCMTP008531 | 5.69773531 | https://www.genecards.org/cgi-bin/carddisp.pl?gene=MT-ATP6 |
| AFG3L2 | AFG3 Like Matrix AAA Peptidase Subunit 2 | Protein Coding | 45 | GC18M012328 | 5.6863904 | https://www.genecards.org/cgi-bin/carddisp.pl?gene=AFG3L2 |
| MYORG | Myogenesis Regulating Glycosidase (Putative) | Protein Coding | 27 | GC09M034366 | 5.538733482 | https://www.genecards.org/cgi-bin/carddisp.pl?gene=MYORG |
| RRM2B | Ribonucleotide Reductase Regulatory TP53 Inducible Subunit M2B | Protein Coding | 50 | GC08M102204 | 5.465931892 | https://www.genecards.org/cgi-bin/carddisp.pl?gene=RRM2B |
| GABRG2 | Gamma-Aminobutyric Acid Type A Receptor Subunit Gamma2 | Protein Coding | 48 | GC05P162000 | 5.464947224 | https://www.genecards.org/cgi-bin/carddisp.pl?gene=GABRG2 |
| SPTAN1 | Spectrin Alpha, Non-Erythrocytic 1 | Protein Coding | 48 | GC09P128552 | 5.453195095 | https://www.genecards.org/cgi-bin/carddisp.pl?gene=SPTAN1 |
| ATP1B2 | ATPase Na+/K+ Transporting Subunit Beta 2 | Protein Coding | 42 | GC17P009113 | 5.420302868 | https://www.genecards.org/cgi-bin/carddisp.pl?gene=ATP1B2 |
| ACVRL1 | Activin A Receptor Like Type 1 | Protein Coding | 50 | GC12P051906 | 5.405187607 | https://www.genecards.org/cgi-bin/carddisp.pl?gene=ACVRL1 |
| INSR | Insulin Receptor | Protein Coding | 54 | GC19M007112 | 5.344673157 | https://www.genecards.org/cgi-bin/carddisp.pl?gene=INSR |
| GNAI2 | G Protein Subunit Alpha I2 | Protein Coding | 48 | GC03P050226 | 5.329061031 | https://www.genecards.org/cgi-bin/carddisp.pl?gene=GNAI2 |
| GNB1 | G Protein Subunit Beta 1 | Protein Coding | 47 | GC01M001785 | 5.288639069 | https://www.genecards.org/cgi-bin/carddisp.pl?gene=GNB1 |
| GNG2 | G Protein Subunit Gamma 2 | Protein Coding | 44 | GC14P051826 | 5.288639069 | https://www.genecards.org/cgi-bin/carddisp.pl?gene=GNG2 |
| LTA | Lymphotoxin Alpha | Protein Coding | 43 | GC06P055200 | 5.27145052 | https://www.genecards.org/cgi-bin/carddisp.pl?gene=LTA |
| PRICKLE2 | Prickle Planar Cell Polarity Protein 2 | Protein Coding | 39 | GC03M064079 | 5.193843842 | https://www.genecards.org/cgi-bin/carddisp.pl?gene=PRICKLE2 |
| MMP2 | Matrix Metallopeptidase 2 | Protein Coding | 54 | GC16P055390 | 5.149697781 | https://www.genecards.org/cgi-bin/carddisp.pl?gene=MMP2 |
| C1QTNF3-AMACR | C1QTNF3-AMACR Readthrough (NMD Candidate) | RNA Gene | 16 | GC05M033989 | 5.119224548 | https://www.genecards.org/cgi-bin/carddisp.pl?gene=C1QTNF3-AMACR |
| APOE | Apolipoprotein E | Protein Coding | 51 | GC19P044906 | 5.056949139 | https://www.genecards.org/cgi-bin/carddisp.pl?gene=APOE |
| CXCL12 | C-X-C Motif Chemokine Ligand 12 | Protein Coding | 46 | GC10M044294 | 5.02746439 | https://www.genecards.org/cgi-bin/carddisp.pl?gene=CXCL12 |
| MTR | 5-Methyltetrahydrofolate-Homocysteine Methyltransferase | Protein Coding | 47 | GC01P236795 | 4.96623373 | https://www.genecards.org/cgi-bin/carddisp.pl?gene=MTR |
| HLA-DQB1 | Major Histocompatibility Complex, Class II, DQ Beta 1 | Protein Coding | 43 | GC06M046939 | 4.945202351 | https://www.genecards.org/cgi-bin/carddisp.pl?gene=HLA-DQB1 |
| MT-CO3 | Mitochondrially Encoded Cytochrome C Oxidase III | Protein Coding | 31 | GCMTP009209 | 4.869398594 | https://www.genecards.org/cgi-bin/carddisp.pl?gene=MT-CO3 |
| TNFRSF1A | TNF Receptor Superfamily Member 1A | Protein Coding | 50 | GC12M006328 | 4.865983009 | https://www.genecards.org/cgi-bin/carddisp.pl?gene=TNFRSF1A |
| STXBP1 | Syntaxin Binding Protein 1 | Protein Coding | 49 | GC09P127582 | 4.837934971 | https://www.genecards.org/cgi-bin/carddisp.pl?gene=STXBP1 |
| SMARCB1 | SWI/SNF Related, Matrix Associated, Actin Dependent Regulator Of Chromatin, Subfamily B, Member 1 | Protein Coding | 46 | GC22P023786 | 4.828901768 | https://www.genecards.org/cgi-bin/carddisp.pl?gene=SMARCB1 |
| SPAST | Spastin | Protein Coding | 40 | GC02P032063 | 4.802659035 | https://www.genecards.org/cgi-bin/carddisp.pl?gene=SPAST |
| SLC25A4 | Solute Carrier Family 25 Member 4 | Protein Coding | 50 | GC04P185143 | 4.763226986 | https://www.genecards.org/cgi-bin/carddisp.pl?gene=SLC25A4 |
| POLG2 | DNA Polymerase Gamma 2, Accessory Subunit | Protein Coding | 42 | GC17M064477 | 4.763226986 | https://www.genecards.org/cgi-bin/carddisp.pl?gene=POLG2 |
| SOD2 | Superoxide Dismutase 2 | Protein Coding | 51 | GC06M159669 | 4.69814682 | https://www.genecards.org/cgi-bin/carddisp.pl?gene=SOD2 |
| HLA-B | Major Histocompatibility Complex, Class I, B | Protein Coding | 46 | GC06M046832 | 4.68954277 | https://www.genecards.org/cgi-bin/carddisp.pl?gene=HLA-B |
| NPFF | Neuropeptide FF-Amide Peptide Precursor | Protein Coding | 37 | GC12M053516 | 4.664049149 | https://www.genecards.org/cgi-bin/carddisp.pl?gene=NPFF |
| COL4A2 | Collagen Type IV Alpha 2 Chain | Protein Coding | 45 | GC13P110305 | 4.65717268 | https://www.genecards.org/cgi-bin/carddisp.pl?gene=COL4A2 |
| PMM2 | Phosphomannomutase 2 | Protein Coding | 47 | GC16P008788 | 4.61831665 | https://www.genecards.org/cgi-bin/carddisp.pl?gene=PMM2 |
| FGFR3 | Fibroblast Growth Factor Receptor 3 | Protein Coding | 55 | GC04P001795 | 4.612111092 | https://www.genecards.org/cgi-bin/carddisp.pl?gene=FGFR3 |
| PIK3CA | Phosphatidylinositol-4,5-Bisphosphate 3-Kinase Catalytic Subunit Alpha | Protein Coding | 53 | GC03P179148 | 4.597136497 | https://www.genecards.org/cgi-bin/carddisp.pl?gene=PIK3CA |
| GABRA5 | Gamma-Aminobutyric Acid Type A Receptor Subunit Alpha5 | Protein Coding | 47 | GC15P026866 | 4.529427052 | https://www.genecards.org/cgi-bin/carddisp.pl?gene=GABRA5 |
| NF1 | Neurofibromin 1 | Protein Coding | 50 | GC17P031094 | 4.509355068 | https://www.genecards.org/cgi-bin/carddisp.pl?gene=NF1 |
| RNASEH1 | Ribonuclease H1 | Protein Coding | 44 | GC02M003544 | 4.509355068 | https://www.genecards.org/cgi-bin/carddisp.pl?gene=RNASEH1 |
| SCN3B | Sodium Voltage-Gated Channel Beta Subunit 3 | Protein Coding | 44 | GC11M123629 | 4.507394791 | https://www.genecards.org/cgi-bin/carddisp.pl?gene=SCN3B |
| ATP1A4 | ATPase Na+/K+ Transporting Subunit Alpha 4 | Protein Coding | 41 | GC01P160151 | 4.504966736 | https://www.genecards.org/cgi-bin/carddisp.pl?gene=ATP1A4 |
| MT-TT | Mitochondrially Encoded TRNA-Thr (ACN) | RNA Gene | 15 | GCMTP015890 | 4.502484322 | https://www.genecards.org/cgi-bin/carddisp.pl?gene=MT-TT |
| MT-TI | Mitochondrially Encoded TRNA-Ile (AUU/C) | RNA Gene | 13 | GCMTP004265 | 4.502484322 | https://www.genecards.org/cgi-bin/carddisp.pl?gene=MT-TI |
| MT-TP | Mitochondrially Encoded TRNA-Pro (CCN) | RNA Gene | 11 | GCMTM015957 | 4.502484322 | https://www.genecards.org/cgi-bin/carddisp.pl?gene=MT-TP |
| AQP1 | Aquaporin 1 (Colton Blood Group) | Protein Coding | 47 | GC07P030911 | 4.499663353 | https://www.genecards.org/cgi-bin/carddisp.pl?gene=AQP1 |
| RAB3GAP1 | RAB3 GTPase Activating Protein Catalytic Subunit 1 | Protein Coding | 41 | GC02P135052 | 4.491138935 | https://www.genecards.org/cgi-bin/carddisp.pl?gene=RAB3GAP1 |
| PRICKLE2-AS1 | PRICKLE2 Antisense RNA 1 | RNA Gene | 13 | GC03P064085 | 4.491138935 | https://www.genecards.org/cgi-bin/carddisp.pl?gene=PRICKLE2-AS1 |
| MYD88 | MYD88 Innate Immune Signal Transduction Adaptor | Protein Coding | 50 | GC03P038139 | 4.47728157 | https://www.genecards.org/cgi-bin/carddisp.pl?gene=MYD88 |
| SLC1A7 | Solute Carrier Family 1 Member 7 | Protein Coding | 44 | GC01M053087 | 4.467217445 | https://www.genecards.org/cgi-bin/carddisp.pl?gene=SLC1A7 |
| SMAD3 | SMAD Family Member 3 | Protein Coding | 50 | GC15P067063 | 4.460811615 | https://www.genecards.org/cgi-bin/carddisp.pl?gene=SMAD3 |
| MT-CO2 | Mitochondrially Encoded Cytochrome C Oxidase II | Protein Coding | 34 | GCMTP007587 | 4.445171356 | https://www.genecards.org/cgi-bin/carddisp.pl?gene=MT-CO2 |
| MT-TS2 | Mitochondrially Encoded TRNA-Ser (AGU/C) 2 | RNA Gene | 13 | GCMTP012215 | 4.445171356 | https://www.genecards.org/cgi-bin/carddisp.pl?gene=MT-TS2 |
| ORAI1 | ORAI Calcium Release-Activated Calcium Modulator 1 | Protein Coding | 44 | GC12P123580 | 4.393100739 | https://www.genecards.org/cgi-bin/carddisp.pl?gene=ORAI1 |
| MT-TE | Mitochondrially Encoded TRNA-Glu (GAA/G) | RNA Gene | 13 | GCMTM012350 | 4.375306606 | https://www.genecards.org/cgi-bin/carddisp.pl?gene=MT-TE |
| MT-TL2 | Mitochondrially Encoded TRNA-Leu (CUN) 2 | RNA Gene | 13 | GCMTP012268 | 4.375306606 | https://www.genecards.org/cgi-bin/carddisp.pl?gene=MT-TL2 |
| MT-TN | Mitochondrially Encoded TRNA-Asn (AAU/C) | RNA Gene | 12 | GCMTM005659 | 4.375306606 | https://www.genecards.org/cgi-bin/carddisp.pl?gene=MT-TN |
| MT-TA | Mitochondrially Encoded TRNA-Ala (GCN) | RNA Gene | 11 | GCMTM005589 | 4.375306606 | https://www.genecards.org/cgi-bin/carddisp.pl?gene=MT-TA |
| TGFB1 | Transforming Growth Factor Beta 1 | Protein Coding | 52 | GC19M041301 | 4.355340481 | https://www.genecards.org/cgi-bin/carddisp.pl?gene=TGFB1 |
| HLA-DRB1 | Major Histocompatibility Complex, Class II, DR Beta 1 | Protein Coding | 48 | GC06M032578 | 4.342678547 | https://www.genecards.org/cgi-bin/carddisp.pl?gene=HLA-DRB1 |
| COL3A1 | Collagen Type III Alpha 1 Chain | Protein Coding | 47 | GC02P188974 | 4.3336339 | https://www.genecards.org/cgi-bin/carddisp.pl?gene=COL3A1 |
| IL23R | Interleukin 23 Receptor | Protein Coding | 45 | GC01P067138 | 4.3336339 | https://www.genecards.org/cgi-bin/carddisp.pl?gene=IL23R |
| RASA1 | RAS P21 Protein Activator 1 | Protein Coding | 47 | GC05P087267 | 4.272550583 | https://www.genecards.org/cgi-bin/carddisp.pl?gene=RASA1 |
| ADA2 | Adenosine Deaminase 2 | Protein Coding | 34 | GC22M017179 | 4.265315056 | https://www.genecards.org/cgi-bin/carddisp.pl?gene=ADA2 |
| LRP1 | LDL Receptor Related Protein 1 | Protein Coding | 48 | GC12P057128 | 4.199330807 | https://www.genecards.org/cgi-bin/carddisp.pl?gene=LRP1 |
| SLC5A1 | Solute Carrier Family 5 Member 1 | Protein Coding | 50 | GC22P032043 | 4.191282749 | https://www.genecards.org/cgi-bin/carddisp.pl?gene=SLC5A1 |
| SLC34A1 | Solute Carrier Family 34 Member 1 | Protein Coding | 45 | GC05P177380 | 4.191282749 | https://www.genecards.org/cgi-bin/carddisp.pl?gene=SLC34A1 |
| SPTB | Spectrin Beta, Erythrocytic | Protein Coding | 41 | GC14M064746 | 4.191282749 | https://www.genecards.org/cgi-bin/carddisp.pl?gene=SPTB |
| CTU2 | Cytosolic Thiouridylase Subunit 2 | Protein Coding | 34 | GC16P088706 | 4.191282749 | https://www.genecards.org/cgi-bin/carddisp.pl?gene=CTU2 |
| SMAD4 | SMAD Family Member 4 | Protein Coding | 51 | GC18P051028 | 4.16327858 | https://www.genecards.org/cgi-bin/carddisp.pl?gene=SMAD4 |
| MVK | Mevalonate Kinase | Protein Coding | 50 | GC12P109573 | 4.16327858 | https://www.genecards.org/cgi-bin/carddisp.pl?gene=MVK |
| MEFV | MEFV Innate Immuity Regulator, Pyrin | Protein Coding | 44 | GC16M003757 | 4.16327858 | https://www.genecards.org/cgi-bin/carddisp.pl?gene=MEFV |
| FBN1 | Fibrillin 1 | Protein Coding | 47 | GC15M048408 | 4.163045406 | https://www.genecards.org/cgi-bin/carddisp.pl?gene=FBN1 |
| SPOP | Speckle Type BTB/POZ Protein | Protein Coding | 44 | GC17M049598 | 4.126197338 | https://www.genecards.org/cgi-bin/carddisp.pl?gene=SPOP |
| OPA1 | OPA1 Mitochondrial Dynamin Like GTPase | Protein Coding | 45 | GC03P193594 | 4.089262486 | https://www.genecards.org/cgi-bin/carddisp.pl?gene=OPA1 |
| KCNV2 | Potassium Voltage-Gated Channel Modifier Subfamily V Member 2 | Protein Coding | 41 | GC09P002717 | 4.055215836 | https://www.genecards.org/cgi-bin/carddisp.pl?gene=KCNV2 |
| NAV1 | Neuron Navigator 1 | Protein Coding | 37 | GC01P201547 | 4.055215836 | https://www.genecards.org/cgi-bin/carddisp.pl?gene=NAV1 |
| NLRP3 | NLR Family Pyrin Domain Containing 3 | Protein Coding | 48 | GC01P247415 | 4.053053856 | https://www.genecards.org/cgi-bin/carddisp.pl?gene=NLRP3 |
| LGI1 | Leucine Rich Glioma Inactivated 1 | Protein Coding | 43 | GC10P093757 | 4.03712368 | https://www.genecards.org/cgi-bin/carddisp.pl?gene=LGI1 |
| EPHB4 | EPH Receptor B4 | Protein Coding | 52 | GC07M100803 | 4.019398212 | https://www.genecards.org/cgi-bin/carddisp.pl?gene=EPHB4 |
| AGTR1 | Angiotensin II Receptor Type 1 | Protein Coding | 51 | GC03P148697 | 4.002402306 | https://www.genecards.org/cgi-bin/carddisp.pl?gene=AGTR1 |
| FAS | Fas Cell Surface Death Receptor | Protein Coding | 51 | GC10P088969 | 3.997188091 | https://www.genecards.org/cgi-bin/carddisp.pl?gene=FAS |
| MSH6 | MutS Homolog 6 | Protein Coding | 51 | GC02P047695 | 3.997188091 | https://www.genecards.org/cgi-bin/carddisp.pl?gene=MSH6 |
| PMS2 | PMS1 Homolog 2, Mismatch Repair System Component | Protein Coding | 49 | GC07M005973 | 3.997188091 | https://www.genecards.org/cgi-bin/carddisp.pl?gene=PMS2 |
| MSH2 | MutS Homolog 2 | Protein Coding | 49 | GC02P047402 | 3.997188091 | https://www.genecards.org/cgi-bin/carddisp.pl?gene=MSH2 |
| MLH1 | MutL Homolog 1 | Protein Coding | 49 | GC03P036993 | 3.997188091 | https://www.genecards.org/cgi-bin/carddisp.pl?gene=MLH1 |
| EPCAM | Epithelial Cell Adhesion Molecule | Protein Coding | 48 | GC02P047345 | 3.997188091 | https://www.genecards.org/cgi-bin/carddisp.pl?gene=EPCAM |
| GP1BA | Glycoprotein Ib Platelet Subunit Alpha | Protein Coding | 46 | GC17P004932 | 3.997188091 | https://www.genecards.org/cgi-bin/carddisp.pl?gene=GP1BA |
| ADAMTSL1 | ADAMTS Like 1 | Protein Coding | 42 | GC09P017906 | 3.997188091 | https://www.genecards.org/cgi-bin/carddisp.pl?gene=ADAMTSL1 |
| STAT4 | Signal Transducer And Activator Of Transcription 4 | Protein Coding | 47 | GC02M191029 | 3.986838102 | https://www.genecards.org/cgi-bin/carddisp.pl?gene=STAT4 |
| ADRA2B | Adrenoceptor Alpha 2B | Protein Coding | 47 | GC02M096112 | 3.976143837 | https://www.genecards.org/cgi-bin/carddisp.pl?gene=ADRA2B |
| NOS2 | Nitric Oxide Synthase 2 | Protein Coding | 50 | GC17M027756 | 3.962589264 | https://www.genecards.org/cgi-bin/carddisp.pl?gene=NOS2 |
| MYH7 | Myosin Heavy Chain 7 | Protein Coding | 48 | GC14M023412 | 3.944924831 | https://www.genecards.org/cgi-bin/carddisp.pl?gene=MYH7 |
| ATP13A1 | ATPase 13A1 | Protein Coding | 40 | GC19M019645 | 3.939987659 | https://www.genecards.org/cgi-bin/carddisp.pl?gene=ATP13A1 |
| NOP53 | NOP53 Ribosome Biogenesis Factor | Protein Coding | 28 | GC19P047749 | 3.939987659 | https://www.genecards.org/cgi-bin/carddisp.pl?gene=NOP53 |
| IL1A | Interleukin 1 Alpha | Protein Coding | 44 | GC02M112773 | 3.921948195 | https://www.genecards.org/cgi-bin/carddisp.pl?gene=IL1A |
| BMPR1A | Bone Morphogenetic Protein Receptor Type 1A | Protein Coding | 51 | GC10P086756 | 3.909406424 | https://www.genecards.org/cgi-bin/carddisp.pl?gene=BMPR1A |
| SH2B1 | SH2B Adaptor Protein 1 | Protein Coding | 44 | GC16P032268 | 3.909406424 | https://www.genecards.org/cgi-bin/carddisp.pl?gene=SH2B1 |
| MLH3 | MutL Homolog 3 | Protein Coding | 43 | GC14M075013 | 3.909406424 | https://www.genecards.org/cgi-bin/carddisp.pl?gene=MLH3 |
| FAN1 | FANCD2 And FANCI Associated Nuclease 1 | Protein Coding | 37 | GC15P032043 | 3.909406424 | https://www.genecards.org/cgi-bin/carddisp.pl?gene=FAN1 |
| ZFTA | Zinc Finger Translocation Associated | Protein Coding | 24 | GC11M071193 | 3.909406424 | https://www.genecards.org/cgi-bin/carddisp.pl?gene=ZFTA |
| ITGB3 | Integrin Subunit Beta 3 | Protein Coding | 50 | GC17P047254 | 3.876300812 | https://www.genecards.org/cgi-bin/carddisp.pl?gene=ITGB3 |
| NRP1 | Neuropilin 1 | Protein Coding | 47 | GC10M033177 | 3.856100559 | https://www.genecards.org/cgi-bin/carddisp.pl?gene=NRP1 |
| GNB3 | G Protein Subunit Beta 3 | Protein Coding | 48 | GC12P006839 | 3.804099321 | https://www.genecards.org/cgi-bin/carddisp.pl?gene=GNB3 |
| MTRR | 5-Methyltetrahydrofolate-Homocysteine Methyltransferase Reductase | Protein Coding | 43 | GC05P007851 | 3.781676054 | https://www.genecards.org/cgi-bin/carddisp.pl?gene=MTRR |
| PGR | Progesterone Receptor | Protein Coding | 51 | GC11M101030 | 3.748556852 | https://www.genecards.org/cgi-bin/carddisp.pl?gene=PGR |
| TP53 | Tumor Protein P53 | Protein Coding | 54 | GC17M007661 | 3.744144201 | https://www.genecards.org/cgi-bin/carddisp.pl?gene=TP53 |
| NFIX | Nuclear Factor I X | Protein Coding | 43 | GC19P012996 | 3.74246645 | https://www.genecards.org/cgi-bin/carddisp.pl?gene=NFIX |
| TWIST1 | Twist Family BHLH Transcription Factor 1 | Protein Coding | 47 | GC07M019020 | 3.689787865 | https://www.genecards.org/cgi-bin/carddisp.pl?gene=TWIST1 |
| CCNH | Cyclin H | Protein Coding | 47 | GC05M087311 | 3.672602177 | https://www.genecards.org/cgi-bin/carddisp.pl?gene=CCNH |
| PLOD1 | Procollagen-Lysine,2-Oxoglutarate 5-Dioxygenase 1 | Protein Coding | 43 | GC01P011934 | 3.672602177 | https://www.genecards.org/cgi-bin/carddisp.pl?gene=PLOD1 |
| CTLA4 | Cytotoxic T-Lymphocyte Associated Protein 4 | Protein Coding | 46 | GC02P203867 | 3.631596565 | https://www.genecards.org/cgi-bin/carddisp.pl?gene=CTLA4 |
| FGFR2 | Fibroblast Growth Factor Receptor 2 | Protein Coding | 54 | GC10M121478 | 3.562610388 | https://www.genecards.org/cgi-bin/carddisp.pl?gene=FGFR2 |
| GRIN2A | Glutamate Ionotropic Receptor NMDA Type Subunit 2A | Protein Coding | 52 | GC16M009753 | 3.562610388 | https://www.genecards.org/cgi-bin/carddisp.pl?gene=GRIN2A |
| KRAS | KRAS Proto-Oncogene, GTPase | Protein Coding | 52 | GC12M025204 | 3.562610388 | https://www.genecards.org/cgi-bin/carddisp.pl?gene=KRAS |
| RELA | RELA Proto-Oncogene, NF-KB Subunit | Protein Coding | 51 | GC11M065653 | 3.562610388 | https://www.genecards.org/cgi-bin/carddisp.pl?gene=RELA |
| DNM1L | Dynamin 1 Like | Protein Coding | 48 | GC12P032679 | 3.562610388 | https://www.genecards.org/cgi-bin/carddisp.pl?gene=DNM1L |
| GATA2 | GATA Binding Protein 2 | Protein Coding | 48 | GC03M128479 | 3.562610388 | https://www.genecards.org/cgi-bin/carddisp.pl?gene=GATA2 |
| GP9 | Glycoprotein IX Platelet | Protein Coding | 47 | GC03P131453 | 3.562610388 | https://www.genecards.org/cgi-bin/carddisp.pl?gene=GP9 |
| CCR1 | C-C Motif Chemokine Receptor 1 | Protein Coding | 45 | GC03M046218 | 3.562610388 | https://www.genecards.org/cgi-bin/carddisp.pl?gene=CCR1 |
| AIP | Aryl Hydrocarbon Receptor Interacting Protein | Protein Coding | 45 | GC11P067468 | 3.562610388 | https://www.genecards.org/cgi-bin/carddisp.pl?gene=AIP |
| SLC6A19 | Solute Carrier Family 6 Member 19 | Protein Coding | 45 | GC05P001201 | 3.562610388 | https://www.genecards.org/cgi-bin/carddisp.pl?gene=SLC6A19 |
| RPS20 | Ribosomal Protein S20 | Protein Coding | 45 | GC08M056067 | 3.562610388 | https://www.genecards.org/cgi-bin/carddisp.pl?gene=RPS20 |
| SEMA4A | Semaphorin 4A | Protein Coding | 45 | GC01P156147 | 3.562610388 | https://www.genecards.org/cgi-bin/carddisp.pl?gene=SEMA4A |
| C4A | Complement C4A (Rodgers Blood Group) | Protein Coding | 44 | GC06P055224 | 3.562610388 | https://www.genecards.org/cgi-bin/carddisp.pl?gene=C4A |
| IL12A | Interleukin 12A | Protein Coding | 44 | GC03P159988 | 3.562610388 | https://www.genecards.org/cgi-bin/carddisp.pl?gene=IL12A |
| IL12B | Interleukin 12B | Protein Coding | 44 | GC05M159314 | 3.562610388 | https://www.genecards.org/cgi-bin/carddisp.pl?gene=IL12B |
| ERAP1 | Endoplasmic Reticulum Aminopeptidase 1 | Protein Coding | 44 | GC05M096760 | 3.562610388 | https://www.genecards.org/cgi-bin/carddisp.pl?gene=ERAP1 |
| MLX | MAX Dimerization Protein MLX | Protein Coding | 44 | GC17P042567 | 3.562610388 | https://www.genecards.org/cgi-bin/carddisp.pl?gene=MLX |
| NOP56 | NOP56 Ribonucleoprotein | Protein Coding | 44 | GC20P003251 | 3.562610388 | https://www.genecards.org/cgi-bin/carddisp.pl?gene=NOP56 |
| GDF2 | Growth Differentiation Factor 2 | Protein Coding | 43 | GC10P047322 | 3.562610388 | https://www.genecards.org/cgi-bin/carddisp.pl?gene=GDF2 |
| GP1BB | Glycoprotein Ib Platelet Subunit Beta | Protein Coding | 43 | GC22P020083 | 3.562610388 | https://www.genecards.org/cgi-bin/carddisp.pl?gene=GP1BB |
| ACSF3 | Acyl-CoA Synthetase Family Member 3 | Protein Coding | 43 | GC16P089088 | 3.562610388 | https://www.genecards.org/cgi-bin/carddisp.pl?gene=ACSF3 |
| NDP | Norrin Cystine Knot Growth Factor NDP | Protein Coding | 43 | GC0XM043948 | 3.562610388 | https://www.genecards.org/cgi-bin/carddisp.pl?gene=NDP |
| PMS1 | PMS1 Homolog 1, Mismatch Repair System Component | Protein Coding | 42 | GC02P189784 | 3.562610388 | https://www.genecards.org/cgi-bin/carddisp.pl?gene=PMS1 |
| SRPX2 | Sushi Repeat Containing Protein X-Linked 2 | Protein Coding | 40 | GC0XP100648 | 3.562610388 | https://www.genecards.org/cgi-bin/carddisp.pl?gene=SRPX2 |
| GPR101 | G Protein-Coupled Receptor 101 | Protein Coding | 39 | GC0XM137030 | 3.562610388 | https://www.genecards.org/cgi-bin/carddisp.pl?gene=GPR101 |
| UBAC2 | UBA Domain Containing 2 | Protein Coding | 38 | GC13P099200 | 3.562610388 | https://www.genecards.org/cgi-bin/carddisp.pl?gene=UBAC2 |
| KLRC4 | Killer Cell Lectin Like Receptor C4 | Protein Coding | 37 | GC12M015687 | 3.562610388 | https://www.genecards.org/cgi-bin/carddisp.pl?gene=KLRC4 |
| IL12A-AS1 | IL12A Antisense RNA 1 | RNA Gene | 15 | GC03M159913 | 3.562610388 | https://www.genecards.org/cgi-bin/carddisp.pl?gene=IL12A-AS1 |
| GABRE | Gamma-Aminobutyric Acid Type A Receptor Subunit Epsilon | Protein Coding | 39 | GC0XM151955 | 3.534742117 | https://www.genecards.org/cgi-bin/carddisp.pl?gene=GABRE |
| IL13 | Interleukin 13 | Protein Coding | 45 | GC05P132656 | 3.457297564 | https://www.genecards.org/cgi-bin/carddisp.pl?gene=IL13 |
| ICAM1 | Intercellular Adhesion Molecule 1 | Protein Coding | 51 | GC19P010270 | 3.430363417 | https://www.genecards.org/cgi-bin/carddisp.pl?gene=ICAM1 |
| NPC1 | NPC Intracellular Cholesterol Transporter 1 | Protein Coding | 47 | GC18M023506 | 3.422745705 | https://www.genecards.org/cgi-bin/carddisp.pl?gene=NPC1 |
| FKRP | Fukutin Related Protein | Protein Coding | 41 | GC19P046746 | 3.422745705 | https://www.genecards.org/cgi-bin/carddisp.pl?gene=FKRP |
| SELE | Selectin E | Protein Coding | 44 | GC01M169722 | 3.374500275 | https://www.genecards.org/cgi-bin/carddisp.pl?gene=SELE |
| STX1A | Syntaxin 1A | Protein Coding | 48 | GC07M073700 | 3.354101181 | https://www.genecards.org/cgi-bin/carddisp.pl?gene=STX1A |
| CYP1A2 | Cytochrome P450 Family 1 Subfamily A Member 2 | Protein Coding | 46 | GC15P074748 | 3.348272085 | https://www.genecards.org/cgi-bin/carddisp.pl?gene=CYP1A2 |
| ITPR1 | Inositol 1,4,5-Trisphosphate Receptor Type 1 | Protein Coding | 49 | GC03P004486 | 3.311575174 | https://www.genecards.org/cgi-bin/carddisp.pl?gene=ITPR1 |
| IL4 | Interleukin 4 | Protein Coding | 46 | GC05P132673 | 3.293929815 | https://www.genecards.org/cgi-bin/carddisp.pl?gene=IL4 |
| CD40LG | CD40 Ligand | Protein Coding | 48 | GC0XP136649 | 3.271596909 | https://www.genecards.org/cgi-bin/carddisp.pl?gene=CD40LG |
| MMP3 | Matrix Metallopeptidase 3 | Protein Coding | 51 | GC11M102835 | 3.254332542 | https://www.genecards.org/cgi-bin/carddisp.pl?gene=MMP3 |
| KCNN3 | Potassium Calcium-Activated Channel Subfamily N Member 3 | Protein Coding | 45 | GC01M154697 | 3.210311413 | https://www.genecards.org/cgi-bin/carddisp.pl?gene=KCNN3 |
| RNR2 | RNA, Ribosomal 45S Cluster 2 | RNA Gene | 8 | GC14U990054 | 3.194432259 | https://www.genecards.org/cgi-bin/carddisp.pl?gene=RNR2 |
| IL1RN | Interleukin 1 Receptor Antagonist | Protein Coding | 48 | GC02P116694 | 3.194206238 | https://www.genecards.org/cgi-bin/carddisp.pl?gene=IL1RN |
| AGT | Angiotensinogen | Protein Coding | 50 | GC01M230702 | 3.176037073 | https://www.genecards.org/cgi-bin/carddisp.pl?gene=AGT |
| EIF2B3 | Eukaryotic Translation Initiation Factor 2B Subunit Gamma | Protein Coding | 43 | GC01M044850 | 3.175885439 | https://www.genecards.org/cgi-bin/carddisp.pl?gene=EIF2B3 |
| MT-CYB | Mitochondrially Encoded Cytochrome B | Protein Coding | 32 | GCMTP014749 | 3.163807154 | https://www.genecards.org/cgi-bin/carddisp.pl?gene=MT-CYB |
| NOTCH4 | Notch Receptor 4 | Protein Coding | 45 | GC06M046911 | 3.154106617 | https://www.genecards.org/cgi-bin/carddisp.pl?gene=NOTCH4 |
| HTRA1 | HtrA Serine Peptidase 1 | Protein Coding | 44 | GC10P122461 | 3.113222599 | https://www.genecards.org/cgi-bin/carddisp.pl?gene=HTRA1 |
| GRIA3 | Glutamate Ionotropic Receptor AMPA Type Subunit 3 | Protein Coding | 51 | GC0XP123184 | 3.091184378 | https://www.genecards.org/cgi-bin/carddisp.pl?gene=GRIA3 |
| SELP | Selectin P | Protein Coding | 46 | GC01M169558 | 3.027704477 | https://www.genecards.org/cgi-bin/carddisp.pl?gene=SELP |
| SERPINC1 | Serpin Family C Member 1 | Protein Coding | 49 | GC01M174251 | 2.976000309 | https://www.genecards.org/cgi-bin/carddisp.pl?gene=SERPINC1 |
| PON1 | Paraoxonase 1 | Protein Coding | 46 | GC07M095297 | 2.974488258 | https://www.genecards.org/cgi-bin/carddisp.pl?gene=PON1 |
| VCAM1 | Vascular Cell Adhesion Molecule 1 | Protein Coding | 46 | GC01P100719 | 2.939922571 | https://www.genecards.org/cgi-bin/carddisp.pl?gene=VCAM1 |
| F8 | Coagulation Factor VIII | Protein Coding | 47 | GC0XM154835 | 2.916763306 | https://www.genecards.org/cgi-bin/carddisp.pl?gene=F8 |
| CYP19A1 | Cytochrome P450 Family 19 Subfamily A Member 1 | Protein Coding | 50 | GC15M051208 | 2.904658794 | https://www.genecards.org/cgi-bin/carddisp.pl?gene=CYP19A1 |
| MTHFD1 | Methylenetetrahydrofolate Dehydrogenase, Cyclohydrolase And Formyltetrahydrofolate Synthetase 1 | Protein Coding | 45 | GC14P064388 | 2.899031878 | https://www.genecards.org/cgi-bin/carddisp.pl?gene=MTHFD1 |
| ADIPOQ | Adiponectin, C1Q And Collagen Domain Containing | Protein Coding | 45 | GC03P186842 | 2.860485554 | https://www.genecards.org/cgi-bin/carddisp.pl?gene=ADIPOQ |
| RELN | Reelin | Protein Coding | 44 | GC07M103471 | 2.833761215 | https://www.genecards.org/cgi-bin/carddisp.pl?gene=RELN |
| HNMT | Histamine N-Methyltransferase | Protein Coding | 46 | GC02P137964 | 2.824909449 | https://www.genecards.org/cgi-bin/carddisp.pl?gene=HNMT |
| F7 | Coagulation Factor VII | Protein Coding | 48 | GC13P113105 | 2.810889721 | https://www.genecards.org/cgi-bin/carddisp.pl?gene=F7 |
| TPH2 | Tryptophan Hydroxylase 2 | Protein Coding | 48 | GC12P071938 | 2.80303359 | https://www.genecards.org/cgi-bin/carddisp.pl?gene=TPH2 |
| HLA-DQA1 | Major Histocompatibility Complex, Class II, DQ Alpha 1 | Protein Coding | 43 | GC06P055231 | 2.797069311 | https://www.genecards.org/cgi-bin/carddisp.pl?gene=HLA-DQA1 |
| GNAQ | G Protein Subunit Alpha Q | Protein Coding | 50 | GC09M077716 | 2.794660091 | https://www.genecards.org/cgi-bin/carddisp.pl?gene=GNAQ |
| TANGO2 | Transport And Golgi Organization 2 Homolog | Protein Coding | 36 | GC22P020017 | 2.794660091 | https://www.genecards.org/cgi-bin/carddisp.pl?gene=TANGO2 |
| LDLR | Low Density Lipoprotein Receptor | Protein Coding | 50 | GC19P011091 | 2.785491943 | https://www.genecards.org/cgi-bin/carddisp.pl?gene=LDLR |
| AQP4 | Aquaporin 4 | Protein Coding | 47 | GC18M026852 | 2.773803711 | https://www.genecards.org/cgi-bin/carddisp.pl?gene=AQP4 |
| MMP9 | Matrix Metallopeptidase 9 | Protein Coding | 54 | GC20P046008 | 2.773236036 | https://www.genecards.org/cgi-bin/carddisp.pl?gene=MMP9 |
| CSF2 | Colony Stimulating Factor 2 | Protein Coding | 44 | GC05P132073 | 2.747124195 | https://www.genecards.org/cgi-bin/carddisp.pl?gene=CSF2 |
| BRAF | B-Raf Proto-Oncogene, Serine/Threonine Kinase | Protein Coding | 54 | GC07M140717 | 2.745979548 | https://www.genecards.org/cgi-bin/carddisp.pl?gene=BRAF |
| C3 | Complement C3 | Protein Coding | 48 | GC19M006677 | 2.736774206 | https://www.genecards.org/cgi-bin/carddisp.pl?gene=C3 |
| SERPINE1 | Serpin Family E Member 1 | Protein Coding | 51 | GC07P101127 | 2.680908442 | https://www.genecards.org/cgi-bin/carddisp.pl?gene=SERPINE1 |
| PF4 | Platelet Factor 4 | Protein Coding | 42 | GC04M073980 | 2.677725792 | https://www.genecards.org/cgi-bin/carddisp.pl?gene=PF4 |
| CNTNAP2 | Contactin Associated Protein 2 | Protein Coding | 44 | GC07P146116 | 2.643223524 | https://www.genecards.org/cgi-bin/carddisp.pl?gene=CNTNAP2 |
| TYMS | Thymidylate Synthetase | Protein Coding | 48 | GC18P000657 | 2.628059626 | https://www.genecards.org/cgi-bin/carddisp.pl?gene=TYMS |
| KCNE2 | Potassium Voltage-Gated Channel Subfamily E Regulatory Subunit 2 | Protein Coding | 42 | GC21P034364 | 2.624014854 | https://www.genecards.org/cgi-bin/carddisp.pl?gene=KCNE2 |
| NOD2 | Nucleotide Binding Oligomerization Domain Containing 2 | Protein Coding | 48 | GC16P050693 | 2.611150265 | https://www.genecards.org/cgi-bin/carddisp.pl?gene=NOD2 |
| YARS2 | Tyrosyl-TRNA Synthetase 2 | Protein Coding | 45 | GC12M032725 | 2.611150265 | https://www.genecards.org/cgi-bin/carddisp.pl?gene=YARS2 |
| ITGA2 | Integrin Subunit Alpha 2 | Protein Coding | 47 | GC05P052989 | 2.603476763 | https://www.genecards.org/cgi-bin/carddisp.pl?gene=ITGA2 |
| VDR | Vitamin D Receptor | Protein Coding | 52 | GC12M047841 | 2.602544785 | https://www.genecards.org/cgi-bin/carddisp.pl?gene=VDR |
| IL9 | Interleukin 9 | Protein Coding | 44 | GC05M135891 | 2.593126774 | https://www.genecards.org/cgi-bin/carddisp.pl?gene=IL9 |
| SERPINA3 | Serpin Family A Member 3 | Protein Coding | 44 | GC14P094612 | 2.580903053 | https://www.genecards.org/cgi-bin/carddisp.pl?gene=SERPINA3 |
| CYP2C19 | Cytochrome P450 Family 2 Subfamily C Member 19 | Protein Coding | 48 | GC10P094762 | 2.553319454 | https://www.genecards.org/cgi-bin/carddisp.pl?gene=CYP2C19 |
| SMARCAL1 | SWI/SNF Related, Matrix Associated, Actin Dependent Regulator Of Chromatin, Subfamily A Like 1 | Protein Coding | 44 | GC02P216412 | 2.539710045 | https://www.genecards.org/cgi-bin/carddisp.pl?gene=SMARCAL1 |
| B9D2 | B9 Domain Containing 2 | Protein Coding | 40 | GC19M041354 | 2.539710045 | https://www.genecards.org/cgi-bin/carddisp.pl?gene=B9D2 |
| CCL2 | C-C Motif Chemokine Ligand 2 | Protein Coding | 49 | GC17P034255 | 2.528849363 | https://www.genecards.org/cgi-bin/carddisp.pl?gene=CCL2 |
| CXCL8 | C-X-C Motif Chemokine Ligand 8 | Protein Coding | 42 | GC04P073740 | 2.52558732 | https://www.genecards.org/cgi-bin/carddisp.pl?gene=CXCL8 |
| CASZ1 | Castor Zinc Finger 1 | Protein Coding | 37 | GC01M010642 | 2.525341034 | https://www.genecards.org/cgi-bin/carddisp.pl?gene=CASZ1 |
| STAT6 | Signal Transducer And Activator Of Transcription 6 | Protein Coding | 50 | GC12M057095 | 2.464353323 | https://www.genecards.org/cgi-bin/carddisp.pl?gene=STAT6 |
| HP | Haptoglobin | Protein Coding | 45 | GC16P072089 | 2.463502884 | https://www.genecards.org/cgi-bin/carddisp.pl?gene=HP |
| ENO2 | Enolase 2 | Protein Coding | 48 | GC12P006913 | 2.432664633 | https://www.genecards.org/cgi-bin/carddisp.pl?gene=ENO2 |
| CACNB2 | Calcium Voltage-Gated Channel Auxiliary Subunit Beta 2 | Protein Coding | 47 | GC10P018165 | 2.417116165 | https://www.genecards.org/cgi-bin/carddisp.pl?gene=CACNB2 |
| ATRX | ATRX Chromatin Remodeler | Protein Coding | 47 | GC0XM077504 | 2.399183512 | https://www.genecards.org/cgi-bin/carddisp.pl?gene=ATRX |
| MEN1 | Menin 1 | Protein Coding | 47 | GC11M064803 | 2.399183512 | https://www.genecards.org/cgi-bin/carddisp.pl?gene=MEN1 |
| DEPDC5 | DEP Domain Containing 5, GATOR1 Subcomplex Subunit | Protein Coding | 40 | GC22P031753 | 2.399183512 | https://www.genecards.org/cgi-bin/carddisp.pl?gene=DEPDC5 |
| LOC101927870 | Uncharacterized LOC101927870 | RNA Gene | 11 | GC07P103445 | 2.399183512 | https://www.genecards.org/cgi-bin/carddisp.pl?gene=LOC101927870 |
| HHT4 | Telangiectasia, Hereditary Hemorrhagic, Type 4 | Genetic Locus | 1 | GC07U901803 | 2.399183512 | https://www.genecards.org/cgi-bin/carddisp.pl?gene=HHT4 |
| PNKD2 | Paroxysmal Nonkinesigenic Dyskinesia 2 | Genetic Locus | 1 | GC02U901605 | 2.399183512 | https://www.genecards.org/cgi-bin/carddisp.pl?gene=PNKD2 |
| ESR2 | Estrogen Receptor 2 | Protein Coding | 50 | GC14M064084 | 2.390331745 | https://www.genecards.org/cgi-bin/carddisp.pl?gene=ESR2 |
| TH | Tyrosine Hydroxylase | Protein Coding | 52 | GC11M002163 | 2.380675793 | https://www.genecards.org/cgi-bin/carddisp.pl?gene=TH |
| GFRA1 | GDNF Family Receptor Alpha 1 | Protein Coding | 44 | GC10M116056 | 2.372825146 | https://www.genecards.org/cgi-bin/carddisp.pl?gene=GFRA1 |
| ABCB1 | ATP Binding Cassette Subfamily B Member 1 | Protein Coding | 52 | GC07M087504 | 2.358950138 | https://www.genecards.org/cgi-bin/carddisp.pl?gene=ABCB1 |
| FANCI | FA Complementation Group I | Protein Coding | 43 | GC15P089243 | 2.32291913 | https://www.genecards.org/cgi-bin/carddisp.pl?gene=FANCI |
| PACS1 | Phosphofurin Acidic Cluster Sorting Protein 1 | Protein Coding | 41 | GC11P066320 | 2.32291913 | https://www.genecards.org/cgi-bin/carddisp.pl?gene=PACS1 |
| EA3 | Episodic Ataxia, Type 3 | Genetic Locus | 1 | GC01U901580 | 2.296427488 | https://www.genecards.org/cgi-bin/carddisp.pl?gene=EA3 |
| EA7 | Episodic Ataxia, Type 7 | Genetic Locus | 1 | GC19U901047 | 2.296427488 | https://www.genecards.org/cgi-bin/carddisp.pl?gene=EA7 |
| EA8 | Episodic Ataxia, Type 8 | Genetic Locus | 1 | GC01U904285 | 2.296427488 | https://www.genecards.org/cgi-bin/carddisp.pl?gene=EA8 |
| STIN2-VNTR | Serotonin Transporter Intronic VNTR Enhancer | Biological Region | 1 | GC17P030221 | 2.295639992 | https://www.genecards.org/cgi-bin/carddisp.pl?gene=STIN2-VNTR |
| PPARA | Peroxisome Proliferator Activated Receptor Alpha | Protein Coding | 47 | GC22P046150 | 2.283615589 | https://www.genecards.org/cgi-bin/carddisp.pl?gene=PPARA |
| PLAUR | Plasminogen Activator, Urokinase Receptor | Protein Coding | 45 | GC19M043646 | 2.277554989 | https://www.genecards.org/cgi-bin/carddisp.pl?gene=PLAUR |
| VEGFA | Vascular Endothelial Growth Factor A | Protein Coding | 49 | GC06P043770 | 2.274986506 | https://www.genecards.org/cgi-bin/carddisp.pl?gene=VEGFA |
| RYR2 | Ryanodine Receptor 2 | Protein Coding | 48 | GC01P237042 | 2.271455526 | https://www.genecards.org/cgi-bin/carddisp.pl?gene=RYR2 |
| GSTP1 | Glutathione S-Transferase Pi 1 | Protein Coding | 51 | GC11P067583 | 2.26651597 | https://www.genecards.org/cgi-bin/carddisp.pl?gene=GSTP1 |
| GAL | Galanin And GMAP Prepropeptide | Protein Coding | 45 | GC11P068684 | 2.263552904 | https://www.genecards.org/cgi-bin/carddisp.pl?gene=GAL |
| POLR1C | RNA Polymerase I And III Subunit C | Protein Coding | 44 | GC06P055413 | 2.260065794 | https://www.genecards.org/cgi-bin/carddisp.pl?gene=POLR1C |
| RBFOX3 | RNA Binding Fox-1 Homolog 3 | Protein Coding | 36 | GC17M079089 | 2.260065794 | https://www.genecards.org/cgi-bin/carddisp.pl?gene=RBFOX3 |
| CCL11 | C-C Motif Chemokine Ligand 11 | Protein Coding | 44 | GC17P034285 | 2.256680727 | https://www.genecards.org/cgi-bin/carddisp.pl?gene=CCL11 |
| MYH6 | Myosin Heavy Chain 6 | Protein Coding | 47 | GC14M023380 | 2.231515646 | https://www.genecards.org/cgi-bin/carddisp.pl?gene=MYH6 |
| CCL5 | C-C Motif Chemokine Ligand 5 | Protein Coding | 44 | GC17M035871 | 2.224834442 | https://www.genecards.org/cgi-bin/carddisp.pl?gene=CCL5 |
| IGLL1 | Immunoglobulin Lambda Like Polypeptide 1 | Protein Coding | 45 | GC22M023573 | 2.213063955 | https://www.genecards.org/cgi-bin/carddisp.pl?gene=IGLL1 |
| AGBL1 | AGBL Carboxypeptidase 1 | Protein Coding | 36 | GC15P086141 | 2.213063955 | https://www.genecards.org/cgi-bin/carddisp.pl?gene=AGBL1 |
| SCN1B | Sodium Voltage-Gated Channel Beta Subunit 1 | Protein Coding | 45 | GC19P035030 | 2.189079762 | https://www.genecards.org/cgi-bin/carddisp.pl?gene=SCN1B |
| PSTPIP1 | Proline-Serine-Threonine Phosphatase Interacting Protein 1 | Protein Coding | 46 | GC15P076993 | 2.18692255 | https://www.genecards.org/cgi-bin/carddisp.pl?gene=PSTPIP1 |
| NBEA | Neurobeachin | Protein Coding | 40 | GC13P034942 | 2.177765131 | https://www.genecards.org/cgi-bin/carddisp.pl?gene=NBEA |
| CCR3 | C-C Motif Chemokine Receptor 3 | Protein Coding | 47 | GC03P046163 | 2.168899059 | https://www.genecards.org/cgi-bin/carddisp.pl?gene=CCR3 |
| CCR5 | C-C Motif Chemokine Receptor 5 | Protein Coding | 47 | GC03P046383 | 2.168899059 | https://www.genecards.org/cgi-bin/carddisp.pl?gene=CCR5 |
| NPPA | Natriuretic Peptide A | Protein Coding | 47 | GC01M011846 | 2.167967081 | https://www.genecards.org/cgi-bin/carddisp.pl?gene=NPPA |
| ADH1B | Alcohol Dehydrogenase 1B (Class I), Beta Polypeptide | Protein Coding | 44 | GC04M099304 | 2.167967081 | https://www.genecards.org/cgi-bin/carddisp.pl?gene=ADH1B |
| ADH1C | Alcohol Dehydrogenase 1C (Class I), Gamma Polypeptide | Protein Coding | 43 | GC04M099336 | 2.167967081 | https://www.genecards.org/cgi-bin/carddisp.pl?gene=ADH1C |
| GPT | Glutamic--Pyruvic Transaminase | Protein Coding | 43 | GC08P144502 | 2.162655115 | https://www.genecards.org/cgi-bin/carddisp.pl?gene=GPT |
| LPA | Lipoprotein(A) | Protein Coding | 42 | GC06M160531 | 2.147768497 | https://www.genecards.org/cgi-bin/carddisp.pl?gene=LPA |
| CCR2 | C-C Motif Chemokine Receptor 2 | Protein Coding | 45 | GC03P046356 | 2.14548254 | https://www.genecards.org/cgi-bin/carddisp.pl?gene=CCR2 |
| SMARCA4 | SWI/SNF Related, Matrix Associated, Actin Dependent Regulator Of Chromatin, Subfamily A, Member 4 | Protein Coding | 50 | GC19P010932 | 2.120706558 | https://www.genecards.org/cgi-bin/carddisp.pl?gene=SMARCA4 |
| MAF | MAF BZIP Transcription Factor | Protein Coding | 45 | GC16M079204 | 2.120706558 | https://www.genecards.org/cgi-bin/carddisp.pl?gene=MAF |
| GC | GC Vitamin D Binding Protein | Protein Coding | 44 | GC04M071741 | 2.110348225 | https://www.genecards.org/cgi-bin/carddisp.pl?gene=GC |
| SLC6A2 | Solute Carrier Family 6 Member 2 | Protein Coding | 49 | GC16P055656 | 2.103300571 | https://www.genecards.org/cgi-bin/carddisp.pl?gene=SLC6A2 |
| XPR1 | Xenotropic And Polytropic Retrovirus Receptor 1 | Protein Coding | 44 | GC01P180632 | 2.103300571 | https://www.genecards.org/cgi-bin/carddisp.pl?gene=XPR1 |
| AHNAK2 | AHNAK Nucleoprotein 2 | Protein Coding | 34 | GC14M104937 | 2.087940216 | https://www.genecards.org/cgi-bin/carddisp.pl?gene=AHNAK2 |
| PNKD | PNKD Metallo-Beta-Lactamase Domain Containing | Protein Coding | 43 | GC02P218270 | 2.082231283 | https://www.genecards.org/cgi-bin/carddisp.pl?gene=PNKD |
| SOD1 | Superoxide Dismutase 1 | Protein Coding | 52 | GC21P031659 | 2.079111814 | https://www.genecards.org/cgi-bin/carddisp.pl?gene=SOD1 |
| HFE | Homeostatic Iron Regulator | Protein Coding | 44 | GC06P026087 | 2.07370472 | https://www.genecards.org/cgi-bin/carddisp.pl?gene=HFE |
| LTC4S | Leukotriene C4 Synthase | Protein Coding | 43 | GC05P179793 | 2.069795132 | https://www.genecards.org/cgi-bin/carddisp.pl?gene=LTC4S |
| NNMT | Nicotinamide N-Methyltransferase | Protein Coding | 46 | GC11P114257 | 2.067199707 | https://www.genecards.org/cgi-bin/carddisp.pl?gene=NNMT |
| KCNB2 | Potassium Voltage-Gated Channel Subfamily B Member 2 | Protein Coding | 43 | GC08P072532 | 2.060427904 | https://www.genecards.org/cgi-bin/carddisp.pl?gene=KCNB2 |
| KCNS3 | Potassium Voltage-Gated Channel Modifier Subfamily S Member 3 | Protein Coding | 41 | GC02P017877 | 2.060427904 | https://www.genecards.org/cgi-bin/carddisp.pl?gene=KCNS3 |
| F2R | Coagulation Factor II Thrombin Receptor | Protein Coding | 47 | GC05P076716 | 2.051805735 | https://www.genecards.org/cgi-bin/carddisp.pl?gene=F2R |
| TBXAS1 | Thromboxane A Synthase 1 | Protein Coding | 51 | GC07P139777 | 2.048318863 | https://www.genecards.org/cgi-bin/carddisp.pl?gene=TBXAS1 |
| TSC1 | TSC Complex Subunit 1 | Protein Coding | 48 | GC09M132891 | 2.048318863 | https://www.genecards.org/cgi-bin/carddisp.pl?gene=TSC1 |
| MT-TC | Mitochondrially Encoded TRNA-Cys (UGU/C) | RNA Gene | 10 | GCMTM005763 | 2.045987606 | https://www.genecards.org/cgi-bin/carddisp.pl?gene=MT-TC |
| COL1A1 | Collagen Type I Alpha 1 Chain | Protein Coding | 51 | GC17M050183 | 2.043274879 | https://www.genecards.org/cgi-bin/carddisp.pl?gene=COL1A1 |
| ASS1 | Argininosuccinate Synthase 1 | Protein Coding | 49 | GC09P130444 | 2.043274879 | https://www.genecards.org/cgi-bin/carddisp.pl?gene=ASS1 |
| CSTB | Cystatin B | Protein Coding | 47 | GC21M043772 | 2.043274879 | https://www.genecards.org/cgi-bin/carddisp.pl?gene=CSTB |
| ARID1B | AT-Rich Interaction Domain 1B | Protein Coding | 45 | GC06P156777 | 2.043274879 | https://www.genecards.org/cgi-bin/carddisp.pl?gene=ARID1B |
| EIF2B2 | Eukaryotic Translation Initiation Factor 2B Subunit Beta | Protein Coding | 45 | GC14P075002 | 2.043274879 | https://www.genecards.org/cgi-bin/carddisp.pl?gene=EIF2B2 |
| RNF135 | Ring Finger Protein 135 | Protein Coding | 41 | GC17P030974 | 2.043274879 | https://www.genecards.org/cgi-bin/carddisp.pl?gene=RNF135 |
| PCDH19 | Protocadherin 19 | Protein Coding | 41 | GC0XM100291 | 2.043274879 | https://www.genecards.org/cgi-bin/carddisp.pl?gene=PCDH19 |
| IER3IP1 | Immediate Early Response 3 Interacting Protein 1 | Protein Coding | 38 | GC18M047152 | 2.043274879 | https://www.genecards.org/cgi-bin/carddisp.pl?gene=IER3IP1 |
| BMIQ16 | Chromosome 16p11.2 Deletion Syndrome, 220kb | Genetic Locus | 1 | GC16U901374 | 2.043274879 | https://www.genecards.org/cgi-bin/carddisp.pl?gene=BMIQ16 |
| DEL17Q11.2 | Chromosome 17q11.2 Deletion Syndrome | Genetic Locus | 1 | GC17U901703 | 2.043274879 | https://www.genecards.org/cgi-bin/carddisp.pl?gene=DEL17Q11.2 |
| FBN2 | Fibrillin 2 | Protein Coding | 41 | GC05M128257 | 2.037968636 | https://www.genecards.org/cgi-bin/carddisp.pl?gene=FBN2 |
| SHBG | Sex Hormone Binding Globulin | Protein Coding | 41 | GC17P007613 | 2.03305006 | https://www.genecards.org/cgi-bin/carddisp.pl?gene=SHBG |
| ATP2C2 | ATPase Secretory Pathway Ca2+ Transporting 2 | Protein Coding | 41 | GC16P084368 | 2.029128313 | https://www.genecards.org/cgi-bin/carddisp.pl?gene=ATP2C2 |
| KCNK12 | Potassium Two Pore Domain Channel Subfamily K Member 12 | Protein Coding | 40 | GC02M047516 | 2.029128313 | https://www.genecards.org/cgi-bin/carddisp.pl?gene=KCNK12 |
| KCNN1 | Potassium Calcium-Activated Channel Subfamily N Member 1 | Protein Coding | 40 | GC19P026648 | 2.028924942 | https://www.genecards.org/cgi-bin/carddisp.pl?gene=KCNN1 |
| MMP1 | Matrix Metallopeptidase 1 | Protein Coding | 52 | GC11M102810 | 2.022052288 | https://www.genecards.org/cgi-bin/carddisp.pl?gene=MMP1 |
| FSHR | Follicle Stimulating Hormone Receptor | Protein Coding | 50 | GC02M048953 | 2.02018857 | https://www.genecards.org/cgi-bin/carddisp.pl?gene=FSHR |
| FASLG | Fas Ligand | Protein Coding | 48 | GC01P172628 | 2.02018857 | https://www.genecards.org/cgi-bin/carddisp.pl?gene=FASLG |
| NRIP1 | Nuclear Receptor Interacting Protein 1 | Protein Coding | 44 | GC21M014961 | 2.02018857 | https://www.genecards.org/cgi-bin/carddisp.pl?gene=NRIP1 |
| JAG1 | Jagged Canonical Notch Ligand 1 | Protein Coding | 51 | GC20M010637 | 2.012710571 | https://www.genecards.org/cgi-bin/carddisp.pl?gene=JAG1 |
| DLL3 | Delta Like Canonical Notch Ligand 3 | Protein Coding | 42 | GC19P039498 | 2.012710571 | https://www.genecards.org/cgi-bin/carddisp.pl?gene=DLL3 |
| HEY2 | Hes Related Family BHLH Transcription Factor With YRPW Motif 2 | Protein Coding | 40 | GC06P125730 | 2.012710571 | https://www.genecards.org/cgi-bin/carddisp.pl?gene=HEY2 |
| HTR4 | 5-Hydroxytryptamine Receptor 4 | Protein Coding | 45 | GC05M148451 | 2.006143093 | https://www.genecards.org/cgi-bin/carddisp.pl?gene=HTR4 |
| DDC | Dopa Decarboxylase | Protein Coding | 53 | GC07M050458 | 1.991741896 | https://www.genecards.org/cgi-bin/carddisp.pl?gene=DDC |
| ABCC9 | ATP Binding Cassette Subfamily C Member 9 | Protein Coding | 45 | GC12M021797 | 1.980856538 | https://www.genecards.org/cgi-bin/carddisp.pl?gene=ABCC9 |
| RHAG | Rh Associated Glycoprotein | Protein Coding | 45 | GC06M049605 | 1.976956367 | https://www.genecards.org/cgi-bin/carddisp.pl?gene=RHAG |
| HTR6 | 5-Hydroxytryptamine Receptor 6 | Protein Coding | 44 | GC01P019666 | 1.976956367 | https://www.genecards.org/cgi-bin/carddisp.pl?gene=HTR6 |
| RLBP1 | Retinaldehyde Binding Protein 1 | Protein Coding | 45 | GC15M089209 | 1.976123095 | https://www.genecards.org/cgi-bin/carddisp.pl?gene=RLBP1 |
| JAM2 | Junctional Adhesion Molecule 2 | Protein Coding | 43 | GC21P025639 | 1.976123095 | https://www.genecards.org/cgi-bin/carddisp.pl?gene=JAM2 |
| OFD1 | OFD1 Centriole And Centriolar Satellite Protein | Protein Coding | 43 | GC0XP013734 | 1.976123095 | https://www.genecards.org/cgi-bin/carddisp.pl?gene=OFD1 |
| INPP5E | Inositol Polyphosphate-5-Phosphatase E | Protein Coding | 42 | GC09M136428 | 1.976123095 | https://www.genecards.org/cgi-bin/carddisp.pl?gene=INPP5E |
| EDAR | Ectodysplasin A Receptor | Protein Coding | 42 | GC02M108894 | 1.976123095 | https://www.genecards.org/cgi-bin/carddisp.pl?gene=EDAR |
| AHI1 | Abelson Helper Integration Site 1 | Protein Coding | 42 | GC06M135283 | 1.976123095 | https://www.genecards.org/cgi-bin/carddisp.pl?gene=AHI1 |
| CEP290 | Centrosomal Protein 290 | Protein Coding | 41 | GC12M088049 | 1.976123095 | https://www.genecards.org/cgi-bin/carddisp.pl?gene=CEP290 |
| CBY1 | Chibby Family Member 1, Beta Catenin Antagonist | Protein Coding | 41 | GC22P038656 | 1.976123095 | https://www.genecards.org/cgi-bin/carddisp.pl?gene=CBY1 |
| MKS1 | MKS Transition Zone Complex Subunit 1 | Protein Coding | 41 | GC17M058205 | 1.976123095 | https://www.genecards.org/cgi-bin/carddisp.pl?gene=MKS1 |
| IFT172 | Intraflagellar Transport 172 | Protein Coding | 40 | GC02M027537 | 1.976123095 | https://www.genecards.org/cgi-bin/carddisp.pl?gene=IFT172 |
| C2CD3 | C2 Domain Containing 3 Centriole Elongation Regulator | Protein Coding | 40 | GC11M074012 | 1.976123095 | https://www.genecards.org/cgi-bin/carddisp.pl?gene=C2CD3 |
| TCTN2 | Tectonic Family Member 2 | Protein Coding | 39 | GC12P123671 | 1.976123095 | https://www.genecards.org/cgi-bin/carddisp.pl?gene=TCTN2 |
| RPGRIP1L | RPGRIP1 Like | Protein Coding | 39 | GC16M053597 | 1.976123095 | https://www.genecards.org/cgi-bin/carddisp.pl?gene=RPGRIP1L |
| CC2D2A | Coiled-Coil And C2 Domain Containing 2A | Protein Coding | 38 | GC04P015471 | 1.976123095 | https://www.genecards.org/cgi-bin/carddisp.pl?gene=CC2D2A |
| TMEM67 | Transmembrane Protein 67 | Protein Coding | 38 | GC08P093754 | 1.976123095 | https://www.genecards.org/cgi-bin/carddisp.pl?gene=TMEM67 |
| B9D1 | B9 Domain Containing 1 | Protein Coding | 37 | GC17M019334 | 1.976123095 | https://www.genecards.org/cgi-bin/carddisp.pl?gene=B9D1 |
| TCTN1 | Tectonic Family Member 1 | Protein Coding | 37 | GC12P110614 | 1.976123095 | https://www.genecards.org/cgi-bin/carddisp.pl?gene=TCTN1 |
| STARD7 | StAR Related Lipid Transfer Domain Containing 7 | Protein Coding | 37 | GC02M096184 | 1.976123095 | https://www.genecards.org/cgi-bin/carddisp.pl?gene=STARD7 |
| TMEM237 | Transmembrane Protein 237 | Protein Coding | 37 | GC02M201620 | 1.976123095 | https://www.genecards.org/cgi-bin/carddisp.pl?gene=TMEM237 |
| KIAA0586 | KIAA0586 | Protein Coding | 36 | GC14P058427 | 1.976123095 | https://www.genecards.org/cgi-bin/carddisp.pl?gene=KIAA0586 |
| TMEM216 | Transmembrane Protein 216 | Protein Coding | 36 | GC11P061391 | 1.976123095 | https://www.genecards.org/cgi-bin/carddisp.pl?gene=TMEM216 |
| KIAA0753 | KIAA0753 | Protein Coding | 34 | GC17M006578 | 1.976123095 | https://www.genecards.org/cgi-bin/carddisp.pl?gene=KIAA0753 |
| C12orf29 | Chromosome 12 Open Reading Frame 29 | Protein Coding | 33 | GC12P088033 | 1.976123095 | https://www.genecards.org/cgi-bin/carddisp.pl?gene=C12orf29 |
| TMEM218 | Transmembrane Protein 218 | Protein Coding | 31 | GC11M125094 | 1.976123095 | https://www.genecards.org/cgi-bin/carddisp.pl?gene=TMEM218 |
| CPLANE1 | Ciliogenesis And Planar Polarity Effector 1 | Protein Coding | 27 | GC05M037084 | 1.976123095 | https://www.genecards.org/cgi-bin/carddisp.pl?gene=CPLANE1 |
| TOGARAM1 | TOG Array Regulator Of Axonemal Microtubules 1 | Protein Coding | 24 | GC14P044963 | 1.976123095 | https://www.genecards.org/cgi-bin/carddisp.pl?gene=TOGARAM1 |
| COL4A2-AS2 | COL4A2 Antisense RNA 2 | RNA Gene | 17 | GC13M110455 | 1.976123095 | https://www.genecards.org/cgi-bin/carddisp.pl?gene=COL4A2-AS2 |
| COL4A2-AS1 | COL4A2 Antisense RNA 1 | RNA Gene | 12 | GC13M110502 | 1.976123095 | https://www.genecards.org/cgi-bin/carddisp.pl?gene=COL4A2-AS1 |
| ECT | Centralopathic Epilepsy | Genetic Locus | 2 | GC11U901791 | 1.976123095 | https://www.genecards.org/cgi-bin/carddisp.pl?gene=ECT |
| BFIS1 | Benign Familial Infantile Convulsions | Genetic Locus | 1 | GC19U901862 | 1.976123095 | https://www.genecards.org/cgi-bin/carddisp.pl?gene=BFIS1 |
| EFHC1 | EF-Hand Domain Containing 1 | Protein Coding | 41 | GC06P052362 | 1.960974097 | https://www.genecards.org/cgi-bin/carddisp.pl?gene=EFHC1 |
| SST | Somatostatin | Protein Coding | 43 | GC03M187668 | 1.947730064 | https://www.genecards.org/cgi-bin/carddisp.pl?gene=SST |
| VWC2 | Von Willebrand Factor C Domain Containing 2 | Protein Coding | 34 | GC07P049783 | 1.945365906 | https://www.genecards.org/cgi-bin/carddisp.pl?gene=VWC2 |
| CACNA1F | Calcium Voltage-Gated Channel Subunit Alpha1 F | Protein Coding | 45 | GC0XM049205 | 1.941547632 | https://www.genecards.org/cgi-bin/carddisp.pl?gene=CACNA1F |
| GNAS | GNAS Complex Locus | Protein Coding | 51 | GC20P058839 | 1.922588944 | https://www.genecards.org/cgi-bin/carddisp.pl?gene=GNAS |
| UTS2 | Urotensin 2 | Protein Coding | 41 | GC01M007843 | 1.872713804 | https://www.genecards.org/cgi-bin/carddisp.pl?gene=UTS2 |
| ADA | Adenosine Deaminase | Protein Coding | 52 | GC20M044620 | 1.865604997 | https://www.genecards.org/cgi-bin/carddisp.pl?gene=ADA |
| IL1RAPL2 | Interleukin 1 Receptor Accessory Protein Like 2 | Protein Coding | 38 | GC0XP104566 | 1.865604997 | https://www.genecards.org/cgi-bin/carddisp.pl?gene=IL1RAPL2 |
| ITGAL | Integrin Subunit Alpha L | Protein Coding | 47 | GC16P030472 | 1.862555146 | https://www.genecards.org/cgi-bin/carddisp.pl?gene=ITGAL |
| HPSE2 | Heparanase 2 (Inactive) | Protein Coding | 42 | GC10M098457 | 1.847186327 | https://www.genecards.org/cgi-bin/carddisp.pl?gene=HPSE2 |
| IRAG1 | Inositol 1,4,5-Triphosphate Receptor Associated 1 | Protein Coding | 29 | GC11M010714 | 1.847186327 | https://www.genecards.org/cgi-bin/carddisp.pl?gene=IRAG1 |
| METTL5 | Methyltransferase Like 5 | Protein Coding | 37 | GC02M169811 | 1.835141301 | https://www.genecards.org/cgi-bin/carddisp.pl?gene=METTL5 |
| CDC42EP2 | CDC42 Effector Protein 2 | Protein Coding | 37 | GC11P065332 | 1.835141301 | https://www.genecards.org/cgi-bin/carddisp.pl?gene=CDC42EP2 |
| SYTL1 | Synaptotagmin Like 1 | Protein Coding | 37 | GC01P027376 | 1.835141301 | https://www.genecards.org/cgi-bin/carddisp.pl?gene=SYTL1 |
| SCPEP1 | Serine Carboxypeptidase 1 | Protein Coding | 37 | GC17P056978 | 1.835141301 | https://www.genecards.org/cgi-bin/carddisp.pl?gene=SCPEP1 |
| AHDC1 | AT-Hook DNA Binding Motif Containing 1 | Protein Coding | 36 | GC01M027534 | 1.835141301 | https://www.genecards.org/cgi-bin/carddisp.pl?gene=AHDC1 |
| RAB40B | RAB40B, Member RAS Oncogene Family | Protein Coding | 36 | GC17M082654 | 1.835141301 | https://www.genecards.org/cgi-bin/carddisp.pl?gene=RAB40B |
| ROPN1L | Rhophilin Associated Tail Protein 1 Like | Protein Coding | 36 | GC05P010441 | 1.835141301 | https://www.genecards.org/cgi-bin/carddisp.pl?gene=ROPN1L |
| RILPL1 | Rab Interacting Lysosomal Protein Like 1 | Protein Coding | 36 | GC12M123470 | 1.835141301 | https://www.genecards.org/cgi-bin/carddisp.pl?gene=RILPL1 |
| ARMC3 | Armadillo Repeat Containing 3 | Protein Coding | 34 | GC10P022928 | 1.835141301 | https://www.genecards.org/cgi-bin/carddisp.pl?gene=ARMC3 |
| ARHGAP36 | Rho GTPase Activating Protein 36 | Protein Coding | 34 | GC0XP131058 | 1.835141301 | https://www.genecards.org/cgi-bin/carddisp.pl?gene=ARHGAP36 |
| PLEKHA2 | Pleckstrin Homology Domain Containing A2 | Protein Coding | 34 | GC08P038877 | 1.835141301 | https://www.genecards.org/cgi-bin/carddisp.pl?gene=PLEKHA2 |
| OTUD1 | OTU Deubiquitinase 1 | Protein Coding | 33 | GC10P023439 | 1.835141301 | https://www.genecards.org/cgi-bin/carddisp.pl?gene=OTUD1 |
| GSTT1 | Glutathione S-Transferase Theta 1 | Protein Coding | 33 | GC22Mi00270 | 1.831938267 | https://www.genecards.org/cgi-bin/carddisp.pl?gene=GSTT1 |
| CBS | Cystathionine Beta-Synthase | Protein Coding | 49 | GC21M043053 | 1.822103024 | https://www.genecards.org/cgi-bin/carddisp.pl?gene=CBS |
| IL4R | Interleukin 4 Receptor | Protein Coding | 48 | GC16P027325 | 1.822103024 | https://www.genecards.org/cgi-bin/carddisp.pl?gene=IL4R |
| SCNN1A | Sodium Channel Epithelial 1 Subunit Alpha | Protein Coding | 48 | GC12M006346 | 1.822103024 | https://www.genecards.org/cgi-bin/carddisp.pl?gene=SCNN1A |
| C5 | Complement C5 | Protein Coding | 47 | GC09M120952 | 1.822103024 | https://www.genecards.org/cgi-bin/carddisp.pl?gene=C5 |
| IL5RA | Interleukin 5 Receptor Subunit Alpha | Protein Coding | 47 | GC03M003066 | 1.822103024 | https://www.genecards.org/cgi-bin/carddisp.pl?gene=IL5RA |
| FGB | Fibrinogen Beta Chain | Protein Coding | 47 | GC04P154572 | 1.822103024 | https://www.genecards.org/cgi-bin/carddisp.pl?gene=FGB |
| CD14 | CD14 Molecule | Protein Coding | 45 | GC05M140631 | 1.822103024 | https://www.genecards.org/cgi-bin/carddisp.pl?gene=CD14 |
| ADD1 | Adducin 1 | Protein Coding | 45 | GC04P002855 | 1.822103024 | https://www.genecards.org/cgi-bin/carddisp.pl?gene=ADD1 |
| TCF7 | Transcription Factor 7 | Protein Coding | 45 | GC05P134114 | 1.822103024 | https://www.genecards.org/cgi-bin/carddisp.pl?gene=TCF7 |
| SCGB1A1 | Secretoglobin Family 1A Member 1 | Protein Coding | 41 | GC11P062405 | 1.822103024 | https://www.genecards.org/cgi-bin/carddisp.pl?gene=SCGB1A1 |
| IL5 | Interleukin 5 | Protein Coding | 45 | GC05M132541 | 1.82117784 | https://www.genecards.org/cgi-bin/carddisp.pl?gene=IL5 |
| IFIH1 | Interferon Induced With Helicase C Domain 1 | Protein Coding | 48 | GC02M162267 | 1.776456594 | https://www.genecards.org/cgi-bin/carddisp.pl?gene=IFIH1 |
| CD40 | CD40 Molecule | Protein Coding | 48 | GC20P046118 | 1.771674991 | https://www.genecards.org/cgi-bin/carddisp.pl?gene=CD40 |
| PRKG1 | Protein Kinase CGMP-Dependent 1 | Protein Coding | 51 | GC10P050991 | 1.760617971 | https://www.genecards.org/cgi-bin/carddisp.pl?gene=PRKG1 |
| IGHE | Immunoglobulin Heavy Constant Epsilon | Protein Coding | 26 | GC14M110308 | 1.733782768 | https://www.genecards.org/cgi-bin/carddisp.pl?gene=IGHE |
| MMP17 | Matrix Metallopeptidase 17 | Protein Coding | 44 | GC12P131828 | 1.729943275 | https://www.genecards.org/cgi-bin/carddisp.pl?gene=MMP17 |
| DLG2 | Discs Large MAGUK Scaffold Protein 2 | Protein Coding | 43 | GC11M083455 | 1.729943275 | https://www.genecards.org/cgi-bin/carddisp.pl?gene=DLG2 |
| INA | Internexin Neuronal Intermediate Filament Protein Alpha | Protein Coding | 40 | GC10P103277 | 1.729943275 | https://www.genecards.org/cgi-bin/carddisp.pl?gene=INA |
| MARCHF4 | Membrane Associated Ring-CH-Type Finger 4 | Protein Coding | 27 | GC02M216260 | 1.729943275 | https://www.genecards.org/cgi-bin/carddisp.pl?gene=MARCHF4 |
| PLCE1 | Phospholipase C Epsilon 1 | Protein Coding | 44 | GC10P093993 | 1.704983234 | https://www.genecards.org/cgi-bin/carddisp.pl?gene=PLCE1 |
| PTX3 | Pentraxin 3 | Protein Coding | 43 | GC03P157436 | 1.701852322 | https://www.genecards.org/cgi-bin/carddisp.pl?gene=PTX3 |
| RET | Ret Proto-Oncogene | Protein Coding | 54 | GC10P043081 | 1.696478963 | https://www.genecards.org/cgi-bin/carddisp.pl?gene=RET |
| FZD4 | Frizzled Class Receptor 4 | Protein Coding | 51 | GC11M086945 | 1.696478963 | https://www.genecards.org/cgi-bin/carddisp.pl?gene=FZD4 |
| GRIN1 | Glutamate Ionotropic Receptor NMDA Type Subunit 1 | Protein Coding | 51 | GC09P137138 | 1.696478963 | https://www.genecards.org/cgi-bin/carddisp.pl?gene=GRIN1 |
| PLCB1 | Phospholipase C Beta 1 | Protein Coding | 49 | GC20P008061 | 1.696478963 | https://www.genecards.org/cgi-bin/carddisp.pl?gene=PLCB1 |
| RB1 | RB Transcriptional Corepressor 1 | Protein Coding | 49 | GC13P048303 | 1.696478963 | https://www.genecards.org/cgi-bin/carddisp.pl?gene=RB1 |
| WWOX | WW Domain Containing Oxidoreductase | Protein Coding | 48 | GC16P078099 | 1.696478963 | https://www.genecards.org/cgi-bin/carddisp.pl?gene=WWOX |
| TLR5 | Toll Like Receptor 5 | Protein Coding | 48 | GC01M223132 | 1.696478963 | https://www.genecards.org/cgi-bin/carddisp.pl?gene=TLR5 |
| ASAH1 | N-Acylsphingosine Amidohydrolase 1 | Protein Coding | 47 | GC08M018055 | 1.696478963 | https://www.genecards.org/cgi-bin/carddisp.pl?gene=ASAH1 |
| SLC25A13 | Solute Carrier Family 25 Member 13 | Protein Coding | 47 | GC07M096120 | 1.696478963 | https://www.genecards.org/cgi-bin/carddisp.pl?gene=SLC25A13 |
| STRADA | STE20 Related Adaptor Alpha | Protein Coding | 46 | GC17M063683 | 1.696478963 | https://www.genecards.org/cgi-bin/carddisp.pl?gene=STRADA |
| DKK1 | Dickkopf WNT Signaling Pathway Inhibitor 1 | Protein Coding | 45 | GC10P052314 | 1.696478963 | https://www.genecards.org/cgi-bin/carddisp.pl?gene=DKK1 |
| EFEMP1 | EGF Containing Fibulin Extracellular Matrix Protein 1 | Protein Coding | 45 | GC02M055865 | 1.696478963 | https://www.genecards.org/cgi-bin/carddisp.pl?gene=EFEMP1 |
| EIF2B4 | Eukaryotic Translation Initiation Factor 2B Subunit Delta | Protein Coding | 45 | GC02M027364 | 1.696478963 | https://www.genecards.org/cgi-bin/carddisp.pl?gene=EIF2B4 |
| CHD2 | Chromodomain Helicase DNA Binding Protein 2 | Protein Coding | 44 | GC15P092900 | 1.696478963 | https://www.genecards.org/cgi-bin/carddisp.pl?gene=CHD2 |
| EIF2B1 | Eukaryotic Translation Initiation Factor 2B Subunit Alpha | Protein Coding | 44 | GC12M123620 | 1.696478963 | https://www.genecards.org/cgi-bin/carddisp.pl?gene=EIF2B1 |
| EIF2B5 | Eukaryotic Translation Initiation Factor 2B Subunit Epsilon | Protein Coding | 44 | GC03P184135 | 1.696478963 | https://www.genecards.org/cgi-bin/carddisp.pl?gene=EIF2B5 |
| TSPAN12 | Tetraspanin 12 | Protein Coding | 44 | GC07M120787 | 1.696478963 | https://www.genecards.org/cgi-bin/carddisp.pl?gene=TSPAN12 |
| SCARB2 | Scavenger Receptor Class B Member 2 | Protein Coding | 44 | GC04M076158 | 1.696478963 | https://www.genecards.org/cgi-bin/carddisp.pl?gene=SCARB2 |
| COL11A1 | Collagen Type XI Alpha 1 Chain | Protein Coding | 43 | GC01M102876 | 1.696478963 | https://www.genecards.org/cgi-bin/carddisp.pl?gene=COL11A1 |
| KCNT1 | Potassium Sodium-Activated Channel Subfamily T Member 1 | Protein Coding | 43 | GC09P135702 | 1.696478963 | https://www.genecards.org/cgi-bin/carddisp.pl?gene=KCNT1 |
| CPA6 | Carboxypeptidase A6 | Protein Coding | 43 | GC08M067422 | 1.696478963 | https://www.genecards.org/cgi-bin/carddisp.pl?gene=CPA6 |
| AARS2 | Alanyl-TRNA Synthetase 2, Mitochondrial | Protein Coding | 43 | GC06M044297 | 1.696478963 | https://www.genecards.org/cgi-bin/carddisp.pl?gene=AARS2 |
| EPM2A | EPM2A Glucan Phosphatase, Laforin | Protein Coding | 43 | GC06M145382 | 1.696478963 | https://www.genecards.org/cgi-bin/carddisp.pl?gene=EPM2A |
| PRICKLE1 | Prickle Planar Cell Polarity Protein 1 | Protein Coding | 43 | GC12M042456 | 1.696478963 | https://www.genecards.org/cgi-bin/carddisp.pl?gene=PRICKLE1 |
| FKBP14 | FKBP Prolyl Isomerase 14 | Protein Coding | 40 | GC07M030010 | 1.696478963 | https://www.genecards.org/cgi-bin/carddisp.pl?gene=FKBP14 |
| SNIP1 | Smad Nuclear Interacting Protein 1 | Protein Coding | 40 | GC01M037534 | 1.696478963 | https://www.genecards.org/cgi-bin/carddisp.pl?gene=SNIP1 |
| ANKLE2 | Ankyrin Repeat And LEM Domain Containing 2 | Protein Coding | 39 | GC12M132725 | 1.696478963 | https://www.genecards.org/cgi-bin/carddisp.pl?gene=ANKLE2 |
| PRSS23 | Serine Protease 23 | Protein Coding | 39 | GC11P086791 | 1.696478963 | https://www.genecards.org/cgi-bin/carddisp.pl?gene=PRSS23 |
| KLHDC8B | Kelch Domain Containing 8B | Protein Coding | 38 | GC03P049171 | 1.696478963 | https://www.genecards.org/cgi-bin/carddisp.pl?gene=KLHDC8B |
| TBC1D24 | TBC1 Domain Family Member 24 | Protein Coding | 38 | GC16P002475 | 1.696478963 | https://www.genecards.org/cgi-bin/carddisp.pl?gene=TBC1D24 |
| RBFOX1 | RNA Binding Fox-1 Homolog 1 | Protein Coding | 38 | GC16P005688 | 1.696478963 | https://www.genecards.org/cgi-bin/carddisp.pl?gene=RBFOX1 |
| SZT2 | SZT2 Subunit Of KICSTOR Complex | Protein Coding | 36 | GC01P043389 | 1.696478963 | https://www.genecards.org/cgi-bin/carddisp.pl?gene=SZT2 |
| SQOR | Sulfide Quinone Oxidoreductase | Protein Coding | 30 | GC15P045632 | 1.696478963 | https://www.genecards.org/cgi-bin/carddisp.pl?gene=SQOR |
| CLTRN | Collectrin, Amino Acid Transport Regulator | Protein Coding | 29 | GC0XM015629 | 1.696478963 | https://www.genecards.org/cgi-bin/carddisp.pl?gene=CLTRN |
| OPA1-AS1 | OPA1 Antisense RNA 1 | RNA Gene | 15 | GC03M193618 | 1.696478963 | https://www.genecards.org/cgi-bin/carddisp.pl?gene=OPA1-AS1 |
| NDP-AS1 | NDP Antisense RNA 1 | RNA Gene | 12 | GC0XP043949 | 1.696478963 | https://www.genecards.org/cgi-bin/carddisp.pl?gene=NDP-AS1 |
| SEPT5-GP1BB | SEPT5-GP1BB Readthrough | RNA Gene | 12 | GC22P019717 | 1.696478963 | https://www.genecards.org/cgi-bin/carddisp.pl?gene=SEPT5-GP1BB |
| SCN1A-AS1 | SCN1A And SCN9A Antisense RNA 1 | RNA Gene | 12 | GC02P165959 | 1.696478963 | https://www.genecards.org/cgi-bin/carddisp.pl?gene=SCN1A-AS1 |
| SIK1B | Salt Inducible Kinase 1B (Putative) | Protein Coding | 12 | GC21P006112 | 1.696478963 | https://www.genecards.org/cgi-bin/carddisp.pl?gene=SIK1B |
| ARFGEF1-DT | ARFGEF1 Divergent Transcript | RNA Gene | 8 | GC08P067381 | 1.696478963 | https://www.genecards.org/cgi-bin/carddisp.pl?gene=ARFGEF1-DT |
| FKBP14-AS1 | FKBP14 Antisense RNA 1 | RNA Gene | 8 | GC07P029991 | 1.696478963 | https://www.genecards.org/cgi-bin/carddisp.pl?gene=FKBP14-AS1 |
| LOC109504727 | NOP56 Ribonucleoprotein Repeat Instability Region | Biological Region | 2 | GC20P003299 | 1.696478963 | https://www.genecards.org/cgi-bin/carddisp.pl?gene=LOC109504727 |
| BACE1 | Beta-Secretase 1 | Protein Coding | 47 | GC11M117285 | 1.682332397 | https://www.genecards.org/cgi-bin/carddisp.pl?gene=BACE1 |
| CLIC5 | Chloride Intracellular Channel 5 | Protein Coding | 43 | GC06M045880 | 1.682332397 | https://www.genecards.org/cgi-bin/carddisp.pl?gene=CLIC5 |
| IRX4 | Iroquois Homeobox 4 | Protein Coding | 38 | GC05M001877 | 1.682332397 | https://www.genecards.org/cgi-bin/carddisp.pl?gene=IRX4 |
| MEPE | Matrix Extracellular Phosphoglycoprotein | Protein Coding | 36 | GC04P087821 | 1.682332397 | https://www.genecards.org/cgi-bin/carddisp.pl?gene=MEPE |
| RNF214 | Ring Finger Protein 214 | Protein Coding | 33 | GC11P117232 | 1.682332397 | https://www.genecards.org/cgi-bin/carddisp.pl?gene=RNF214 |
| TFAM | Transcription Factor A, Mitochondrial | Protein Coding | 45 | GC10P058385 | 1.681406498 | https://www.genecards.org/cgi-bin/carddisp.pl?gene=TFAM |
| ADRA1B | Adrenoceptor Alpha 1B | Protein Coding | 47 | GC05P159867 | 1.67784071 | https://www.genecards.org/cgi-bin/carddisp.pl?gene=ADRA1B |
| SV2C | Synaptic Vesicle Glycoprotein 2C | Protein Coding | 40 | GC05P075847 | 1.67784071 | https://www.genecards.org/cgi-bin/carddisp.pl?gene=SV2C |
| KCNMA1 | Potassium Calcium-Activated Channel Subfamily M Alpha 1 | Protein Coding | 50 | GC10M076869 | 1.659805179 | https://www.genecards.org/cgi-bin/carddisp.pl?gene=KCNMA1 |
| KCNJ10 | Potassium Inwardly Rectifying Channel Subfamily J Member 10 | Protein Coding | 45 | GC01M159998 | 1.649477005 | https://www.genecards.org/cgi-bin/carddisp.pl?gene=KCNJ10 |
| HTR5A | 5-Hydroxytryptamine Receptor 5A | Protein Coding | 43 | GC07P155070 | 1.649477005 | https://www.genecards.org/cgi-bin/carddisp.pl?gene=HTR5A |
| KCNJ9 | Potassium Inwardly Rectifying Channel Subfamily J Member 9 | Protein Coding | 43 | GC01P160079 | 1.649477005 | https://www.genecards.org/cgi-bin/carddisp.pl?gene=KCNJ9 |
| SRR | Serine Racemase | Protein Coding | 44 | GC17P002303 | 1.647818208 | https://www.genecards.org/cgi-bin/carddisp.pl?gene=SRR |
| SHMT1 | Serine Hydroxymethyltransferase 1 | Protein Coding | 46 | GC17M021636 | 1.64466536 | https://www.genecards.org/cgi-bin/carddisp.pl?gene=SHMT1 |
| IGF1 | Insulin Like Growth Factor 1 | Protein Coding | 50 | GC12M102395 | 1.63519454 | https://www.genecards.org/cgi-bin/carddisp.pl?gene=IGF1 |
| TYMP | Thymidine Phosphorylase | Protein Coding | 47 | GC22M050525 | 1.63519454 | https://www.genecards.org/cgi-bin/carddisp.pl?gene=TYMP |
| IL18 | Interleukin 18 | Protein Coding | 44 | GC11M112143 | 1.634264827 | https://www.genecards.org/cgi-bin/carddisp.pl?gene=IL18 |
| GSR | Glutathione-Disulfide Reductase | Protein Coding | 50 | GC08M030678 | 1.624166369 | https://www.genecards.org/cgi-bin/carddisp.pl?gene=GSR |
| SPTBN2 | Spectrin Beta, Non-Erythrocytic 2 | Protein Coding | 43 | GC11M069493 | 1.623914719 | https://www.genecards.org/cgi-bin/carddisp.pl?gene=SPTBN2 |
| ATXN7 | Ataxin 7 | Protein Coding | 40 | GC03P063864 | 1.623914719 | https://www.genecards.org/cgi-bin/carddisp.pl?gene=ATXN7 |
| AR | Androgen Receptor | Protein Coding | 53 | GC0XP067544 | 1.614178181 | https://www.genecards.org/cgi-bin/carddisp.pl?gene=AR |
| TNFRSF21 | TNF Receptor Superfamily Member 21 | Protein Coding | 45 | GC06M047231 | 1.614178181 | https://www.genecards.org/cgi-bin/carddisp.pl?gene=TNFRSF21 |
| MEP1A | Meprin A Subunit Alpha | Protein Coding | 43 | GC06P046793 | 1.614178181 | https://www.genecards.org/cgi-bin/carddisp.pl?gene=MEP1A |
| SLC25A27 | Solute Carrier Family 25 Member 27 | Protein Coding | 39 | GC06P046652 | 1.614178181 | https://www.genecards.org/cgi-bin/carddisp.pl?gene=SLC25A27 |
| NR3C1 | Nuclear Receptor Subfamily 3 Group C Member 1 | Protein Coding | 51 | GC05M143277 | 1.61374104 | https://www.genecards.org/cgi-bin/carddisp.pl?gene=NR3C1 |
| CHRM2 | Cholinergic Receptor Muscarinic 2 | Protein Coding | 49 | GC07P136868 | 1.61374104 | https://www.genecards.org/cgi-bin/carddisp.pl?gene=CHRM2 |
| ACTN4 | Actinin Alpha 4 | Protein Coding | 47 | GC19P038647 | 1.61374104 | https://www.genecards.org/cgi-bin/carddisp.pl?gene=ACTN4 |
| NEDD4L | NEDD4 Like E3 Ubiquitin Protein Ligase | Protein Coding | 47 | GC18P058044 | 1.61374104 | https://www.genecards.org/cgi-bin/carddisp.pl?gene=NEDD4L |
| FOXP1 | Forkhead Box P1 | Protein Coding | 46 | GC03M070926 | 1.61374104 | https://www.genecards.org/cgi-bin/carddisp.pl?gene=FOXP1 |
| EYA1 | EYA Transcriptional Coactivator And Phosphatase 1 | Protein Coding | 46 | GC08M071210 | 1.61374104 | https://www.genecards.org/cgi-bin/carddisp.pl?gene=EYA1 |
| INHBA | Inhibin Subunit Beta A | Protein Coding | 45 | GC07M041668 | 1.61374104 | https://www.genecards.org/cgi-bin/carddisp.pl?gene=INHBA |
| MMP16 | Matrix Metallopeptidase 16 | Protein Coding | 45 | GC08M088032 | 1.61374104 | https://www.genecards.org/cgi-bin/carddisp.pl?gene=MMP16 |
| PTPRD | Protein Tyrosine Phosphatase Receptor Type D | Protein Coding | 45 | GC09M008307 | 1.61374104 | https://www.genecards.org/cgi-bin/carddisp.pl?gene=PTPRD |
| GFRA2 | GDNF Family Receptor Alpha 2 | Protein Coding | 44 | GC08M021690 | 1.61374104 | https://www.genecards.org/cgi-bin/carddisp.pl?gene=GFRA2 |
| CDH13 | Cadherin 13 | Protein Coding | 44 | GC16P082626 | 1.61374104 | https://www.genecards.org/cgi-bin/carddisp.pl?gene=CDH13 |
| SUV39H2 | Suppressor Of Variegation 3-9 Homolog 2 | Protein Coding | 44 | GC10P014878 | 1.61374104 | https://www.genecards.org/cgi-bin/carddisp.pl?gene=SUV39H2 |
| STK10 | Serine/Threonine Kinase 10 | Protein Coding | 44 | GC05M172042 | 1.61374104 | https://www.genecards.org/cgi-bin/carddisp.pl?gene=STK10 |
| NSD1 | Nuclear Receptor Binding SET Domain Protein 1 | Protein Coding | 44 | GC05P177134 | 1.61374104 | https://www.genecards.org/cgi-bin/carddisp.pl?gene=NSD1 |
| CDH4 | Cadherin 4 | Protein Coding | 43 | GC20P061252 | 1.61374104 | https://www.genecards.org/cgi-bin/carddisp.pl?gene=CDH4 |
| CNNM2 | Cyclin And CBS Domain Divalent Metal Cation Transport Mediator 2 | Protein Coding | 43 | GC10P102918 | 1.61374104 | https://www.genecards.org/cgi-bin/carddisp.pl?gene=CNNM2 |
| WASL | WASP Like Actin Nucleation Promoting Factor | Protein Coding | 43 | GC07M123681 | 1.61374104 | https://www.genecards.org/cgi-bin/carddisp.pl?gene=WASL |
| SMYD3 | SET And MYND Domain Containing 3 | Protein Coding | 43 | GC01M245749 | 1.61374104 | https://www.genecards.org/cgi-bin/carddisp.pl?gene=SMYD3 |
| TBC1D7 | TBC1 Domain Family Member 7 | Protein Coding | 42 | GC06M013266 | 1.61374104 | https://www.genecards.org/cgi-bin/carddisp.pl?gene=TBC1D7 |
| SORBS1 | Sorbin And SH3 Domain Containing 1 | Protein Coding | 42 | GC10M095311 | 1.61374104 | https://www.genecards.org/cgi-bin/carddisp.pl?gene=SORBS1 |
| ATF7 | Activating Transcription Factor 7 | Protein Coding | 41 | GC12M053527 | 1.61374104 | https://www.genecards.org/cgi-bin/carddisp.pl?gene=ATF7 |
| KANK1 | KN Motif And Ankyrin Repeat Domains 1 | Protein Coding | 41 | GC09P000474 | 1.61374104 | https://www.genecards.org/cgi-bin/carddisp.pl?gene=KANK1 |
| KSR2 | Kinase Suppressor Of Ras 2 | Protein Coding | 40 | GC12M117453 | 1.61374104 | https://www.genecards.org/cgi-bin/carddisp.pl?gene=KSR2 |
| ADARB2 | Adenosine Deaminase RNA Specific B2 (Inactive) | Protein Coding | 40 | GC10M001187 | 1.61374104 | https://www.genecards.org/cgi-bin/carddisp.pl?gene=ADARB2 |
| CTNNA3 | Catenin Alpha 3 | Protein Coding | 40 | GC10M065912 | 1.61374104 | https://www.genecards.org/cgi-bin/carddisp.pl?gene=CTNNA3 |
| RSU1 | Ras Suppressor Protein 1 | Protein Coding | 40 | GC10M016672 | 1.61374104 | https://www.genecards.org/cgi-bin/carddisp.pl?gene=RSU1 |
| PHF20 | PHD Finger Protein 20 | Protein Coding | 39 | GC20P035771 | 1.61374104 | https://www.genecards.org/cgi-bin/carddisp.pl?gene=PHF20 |
| FARP1 | FERM, ARH/RhoGEF And Pleckstrin Domain Protein 1 | Protein Coding | 38 | GC13P098142 | 1.61374104 | https://www.genecards.org/cgi-bin/carddisp.pl?gene=FARP1 |
| FRMD4A | FERM Domain Containing 4A | Protein Coding | 37 | GC10M013643 | 1.61374104 | https://www.genecards.org/cgi-bin/carddisp.pl?gene=FRMD4A |
| ZDHHC6 | Zinc Finger DHHC-Type Palmitoyltransferase 6 | Protein Coding | 37 | GC10M112430 | 1.61374104 | https://www.genecards.org/cgi-bin/carddisp.pl?gene=ZDHHC6 |
| LEPROTL1 | Leptin Receptor Overlapping Transcript Like 1 | Protein Coding | 36 | GC08P030095 | 1.61374104 | https://www.genecards.org/cgi-bin/carddisp.pl?gene=LEPROTL1 |
| LIMCH1 | LIM And Calponin Homology Domains 1 | Protein Coding | 36 | GC04P041362 | 1.61374104 | https://www.genecards.org/cgi-bin/carddisp.pl?gene=LIMCH1 |
| ARL15 | ADP Ribosylation Factor Like GTPase 15 | Protein Coding | 36 | GC05M053883 | 1.61374104 | https://www.genecards.org/cgi-bin/carddisp.pl?gene=ARL15 |
| SNX24 | Sorting Nexin 24 | Protein Coding | 36 | GC05P122843 | 1.61374104 | https://www.genecards.org/cgi-bin/carddisp.pl?gene=SNX24 |
| MRPL37 | Mitochondrial Ribosomal Protein L37 | Protein Coding | 36 | GC01P054185 | 1.61374104 | https://www.genecards.org/cgi-bin/carddisp.pl?gene=MRPL37 |
| RABGAP1L | RAB GTPase Activating Protein 1 Like | Protein Coding | 36 | GC01P174128 | 1.61374104 | https://www.genecards.org/cgi-bin/carddisp.pl?gene=RABGAP1L |
| SLC35D2 | Solute Carrier Family 35 Member D2 | Protein Coding | 36 | GC09M096313 | 1.61374104 | https://www.genecards.org/cgi-bin/carddisp.pl?gene=SLC35D2 |
| HAUS1 | HAUS Augmin Like Complex Subunit 1 | Protein Coding | 35 | GC18P046104 | 1.61374104 | https://www.genecards.org/cgi-bin/carddisp.pl?gene=HAUS1 |
| AJAP1 | Adherens Junctions Associated Protein 1 | Protein Coding | 34 | GC01P004654 | 1.61374104 | https://www.genecards.org/cgi-bin/carddisp.pl?gene=AJAP1 |
| CTIF | Cap Binding Complex Dependent Translation Initiation Factor | Protein Coding | 33 | GC18P048539 | 1.61374104 | https://www.genecards.org/cgi-bin/carddisp.pl?gene=CTIF |
| COX7B2 | Cytochrome C Oxidase Subunit 7B2 | Protein Coding | 32 | GC04M046734 | 1.61374104 | https://www.genecards.org/cgi-bin/carddisp.pl?gene=COX7B2 |
| VSTM4 | V-Set And Transmembrane Domain Containing 4 | Protein Coding | 32 | GC10M049014 | 1.61374104 | https://www.genecards.org/cgi-bin/carddisp.pl?gene=VSTM4 |
| WAPL | WAPL Cohesin Release Factor | Protein Coding | 31 | GC10M086436 | 1.61374104 | https://www.genecards.org/cgi-bin/carddisp.pl?gene=WAPL |
| BPIFC | BPI Fold Containing Family C | Protein Coding | 29 | GC22M032413 | 1.61374104 | https://www.genecards.org/cgi-bin/carddisp.pl?gene=BPIFC |
| TRMT9B | TRNA Methyltransferase 9B (Putative) | Protein Coding | 27 | GC08P012945 | 1.61374104 | https://www.genecards.org/cgi-bin/carddisp.pl?gene=TRMT9B |
| CDKN2B-AS1 | CDKN2B Antisense RNA 1 | RNA Gene | 22 | GC09P021994 | 1.61374104 | https://www.genecards.org/cgi-bin/carddisp.pl?gene=CDKN2B-AS1 |
| INHBA-AS1 | INHBA Antisense RNA 1 | RNA Gene | 15 | GC07P041693 | 1.61374104 | https://www.genecards.org/cgi-bin/carddisp.pl?gene=INHBA-AS1 |
| NR2F2-AS1 | NR2F2 Antisense RNA 1 | RNA Gene | 14 | GC15M107462 | 1.61374104 | https://www.genecards.org/cgi-bin/carddisp.pl?gene=NR2F2-AS1 |
| OTUD6B-AS1 | OTUD6B Antisense RNA 1 (Head To Head) | RNA Gene | 12 | GC08M091059 | 1.61374104 | https://www.genecards.org/cgi-bin/carddisp.pl?gene=OTUD6B-AS1 |
| LOC349160 | Uncharacterized LOC349160 | RNA Gene | 12 | GC07M136684 | 1.61374104 | https://www.genecards.org/cgi-bin/carddisp.pl?gene=LOC349160 |
| LOC102723445 | Uncharacterized LOC102723445 | RNA Gene | 1 | GC06M131087 | 1.61374104 | https://www.genecards.org/cgi-bin/carddisp.pl?gene=LOC102723445 |
| S100A12 | S100 Calcium Binding Protein A12 | Protein Coding | 40 | GC01M153373 | 1.604697347 | https://www.genecards.org/cgi-bin/carddisp.pl?gene=S100A12 |
| CYP1A1 | Cytochrome P450 Family 1 Subfamily A Member 1 | Protein Coding | 48 | GC15M074719 | 1.598472357 | https://www.genecards.org/cgi-bin/carddisp.pl?gene=CYP1A1 |
| OGG1 | 8-Oxoguanine DNA Glycosylase | Protein Coding | 47 | GC03P009751 | 1.578842878 | https://www.genecards.org/cgi-bin/carddisp.pl?gene=OGG1 |
| XRCC3 | X-Ray Repair Cross Complementing 3 | Protein Coding | 43 | GC14M103697 | 1.578842878 | https://www.genecards.org/cgi-bin/carddisp.pl?gene=XRCC3 |
| GATA4 | GATA Binding Protein 4 | Protein Coding | 49 | GC08P011676 | 1.57585144 | https://www.genecards.org/cgi-bin/carddisp.pl?gene=GATA4 |
| DIDO1 | Death Inducer-Obliterator 1 | Protein Coding | 38 | GC20M062877 | 1.575793028 | https://www.genecards.org/cgi-bin/carddisp.pl?gene=DIDO1 |
| CACNA1G | Calcium Voltage-Gated Channel Subunit Alpha1 G | Protein Coding | 50 | GC17P050561 | 1.569998622 | https://www.genecards.org/cgi-bin/carddisp.pl?gene=CACNA1G |
| CD79A | CD79a Molecule | Protein Coding | 47 | GC19P041877 | 1.561330199 | https://www.genecards.org/cgi-bin/carddisp.pl?gene=CD79A |
| PPARG | Peroxisome Proliferator Activated Receptor Gamma | Protein Coding | 53 | GC03P012287 | 1.548831582 | https://www.genecards.org/cgi-bin/carddisp.pl?gene=PPARG |
| PPARD | Peroxisome Proliferator Activated Receptor Delta | Protein Coding | 48 | GC06P055324 | 1.548831582 | https://www.genecards.org/cgi-bin/carddisp.pl?gene=PPARD |
| TNFSF10 | TNF Superfamily Member 10 | Protein Coding | 47 | GC03M172505 | 1.548831582 | https://www.genecards.org/cgi-bin/carddisp.pl?gene=TNFSF10 |
| LEPQTL1 | Leptin, Serum Levels Of | Genetic Locus | 2 | GC02U903086 | 1.548831582 | https://www.genecards.org/cgi-bin/carddisp.pl?gene=LEPQTL1 |
| CFLAR | CASP8 And FADD Like Apoptosis Regulator | Protein Coding | 47 | GC02P201117 | 1.542047262 | https://www.genecards.org/cgi-bin/carddisp.pl?gene=CFLAR |
| DDIT3 | DNA Damage Inducible Transcript 3 | Protein Coding | 46 | GC12M057516 | 1.537916422 | https://www.genecards.org/cgi-bin/carddisp.pl?gene=DDIT3 |
| GABRA3 | Gamma-Aminobutyric Acid Type A Receptor Subunit Alpha3 | Protein Coding | 45 | GC0XM152166 | 1.521786928 | https://www.genecards.org/cgi-bin/carddisp.pl?gene=GABRA3 |
| CYP20A1 | Cytochrome P450 Family 20 Subfamily A Member 1 | Protein Coding | 37 | GC02P203238 | 1.520640612 | https://www.genecards.org/cgi-bin/carddisp.pl?gene=CYP20A1 |
| TNFRSF1B | TNF Receptor Superfamily Member 1B | Protein Coding | 48 | GC01P012167 | 1.513152361 | https://www.genecards.org/cgi-bin/carddisp.pl?gene=TNFRSF1B |
| MAP2K2 | Mitogen-Activated Protein Kinase Kinase 2 | Protein Coding | 54 | GC19M004090 | 1.487991691 | https://www.genecards.org/cgi-bin/carddisp.pl?gene=MAP2K2 |
| DSP | Desmoplakin | Protein Coding | 50 | GC06P007541 | 1.487991691 | https://www.genecards.org/cgi-bin/carddisp.pl?gene=DSP |
| AHSG | Alpha 2-HS Glycoprotein | Protein Coding | 44 | GC03P186612 | 1.487775564 | https://www.genecards.org/cgi-bin/carddisp.pl?gene=AHSG |
| MAPK3 | Mitogen-Activated Protein Kinase 3 | Protein Coding | 50 | GC16M031188 | 1.484731913 | https://www.genecards.org/cgi-bin/carddisp.pl?gene=MAPK3 |
| SAMHD1 | SAM And HD Domain Containing Deoxynucleoside Triphosphate Triphosphohydrolase 1 | Protein Coding | 43 | GC20M036890 | 1.479406357 | https://www.genecards.org/cgi-bin/carddisp.pl?gene=SAMHD1 |
| HLA-A | Major Histocompatibility Complex, Class I, A | Protein Coding | 48 | GC06P055161 | 1.478258133 | https://www.genecards.org/cgi-bin/carddisp.pl?gene=HLA-A |
| RYR1 | Ryanodine Receptor 1 | Protein Coding | 47 | GC19P040452 | 1.461788058 | https://www.genecards.org/cgi-bin/carddisp.pl?gene=RYR1 |
| MIR30A | MicroRNA 30a | RNA Gene | 20 | GC06M071403 | 1.461049795 | https://www.genecards.org/cgi-bin/carddisp.pl?gene=MIR30A |
| VWF | Von Willebrand Factor | Protein Coding | 49 | GC12M005917 | 1.45947361 | https://www.genecards.org/cgi-bin/carddisp.pl?gene=VWF |
| EGF | Epidermal Growth Factor | Protein Coding | 51 | GC04P109912 | 1.449123621 | https://www.genecards.org/cgi-bin/carddisp.pl?gene=EGF |
| NOTCH1 | Notch Receptor 1 | Protein Coding | 51 | GC09M136705 | 1.449123621 | https://www.genecards.org/cgi-bin/carddisp.pl?gene=NOTCH1 |
| NOTCH2 | Notch Receptor 2 | Protein Coding | 50 | GC01M119911 | 1.449123621 | https://www.genecards.org/cgi-bin/carddisp.pl?gene=NOTCH2 |
| RBPJ | Recombination Signal Binding Protein For Immunoglobulin Kappa J Region | Protein Coding | 48 | GC04P026165 | 1.449123621 | https://www.genecards.org/cgi-bin/carddisp.pl?gene=RBPJ |
| DLL1 | Delta Like Canonical Notch Ligand 1 | Protein Coding | 46 | GC06M170282 | 1.449123621 | https://www.genecards.org/cgi-bin/carddisp.pl?gene=DLL1 |
| DLL4 | Delta Like Canonical Notch Ligand 4 | Protein Coding | 46 | GC15P040929 | 1.449123621 | https://www.genecards.org/cgi-bin/carddisp.pl?gene=DLL4 |
| TIMP3 | TIMP Metallopeptidase Inhibitor 3 | Protein Coding | 46 | GC22P032800 | 1.449123621 | https://www.genecards.org/cgi-bin/carddisp.pl?gene=TIMP3 |
| HEY1 | Hes Related Family BHLH Transcription Factor With YRPW Motif 1 | Protein Coding | 44 | GC08M079764 | 1.449123621 | https://www.genecards.org/cgi-bin/carddisp.pl?gene=HEY1 |
| KCNC3 | Potassium Voltage-Gated Channel Subfamily C Member 3 | Protein Coding | 44 | GC19M050322 | 1.449123621 | https://www.genecards.org/cgi-bin/carddisp.pl?gene=KCNC3 |
| JAG2 | Jagged Canonical Notch Ligand 2 | Protein Coding | 43 | GC14M105140 | 1.449123621 | https://www.genecards.org/cgi-bin/carddisp.pl?gene=JAG2 |
| DARS2 | Aspartyl-TRNA Synthetase 2, Mitochondrial | Protein Coding | 43 | GC01P173824 | 1.449123621 | https://www.genecards.org/cgi-bin/carddisp.pl?gene=DARS2 |
| HEYL | Hes Related Family BHLH Transcription Factor With YRPW Motif Like | Protein Coding | 40 | GC01M039623 | 1.449123621 | https://www.genecards.org/cgi-bin/carddisp.pl?gene=HEYL |
| SRRT | Serrate, RNA Effector Molecule | Protein Coding | 38 | GC07P100875 | 1.449123621 | https://www.genecards.org/cgi-bin/carddisp.pl?gene=SRRT |
| IRF3 | Interferon Regulatory Factor 3 | Protein Coding | 47 | GC19M049659 | 1.445152521 | https://www.genecards.org/cgi-bin/carddisp.pl?gene=IRF3 |
| DDX58 | DExD/H-Box Helicase 58 | Protein Coding | 47 | GC09M032455 | 1.445152521 | https://www.genecards.org/cgi-bin/carddisp.pl?gene=DDX58 |
| EXO1 | Exonuclease 1 | Protein Coding | 44 | GC01P241847 | 1.445152521 | https://www.genecards.org/cgi-bin/carddisp.pl?gene=EXO1 |
| CTNNB1 | Catenin Beta 1 | Protein Coding | 54 | GC03P041236 | 1.434746265 | https://www.genecards.org/cgi-bin/carddisp.pl?gene=CTNNB1 |
| HTR3D | 5-Hydroxytryptamine Receptor 3D | Protein Coding | 36 | GC03P184031 | 1.43251133 | https://www.genecards.org/cgi-bin/carddisp.pl?gene=HTR3D |
| PRKCG | Protein Kinase C Gamma | Protein Coding | 52 | GC19P053879 | 1.428375721 | https://www.genecards.org/cgi-bin/carddisp.pl?gene=PRKCG |
| ATXN1 | Ataxin 1 | Protein Coding | 44 | GC06M016299 | 1.428375721 | https://www.genecards.org/cgi-bin/carddisp.pl?gene=ATXN1 |
| MIR9-1 | MicroRNA 9-1 | RNA Gene | 20 | GC01M156420 | 1.39770031 | https://www.genecards.org/cgi-bin/carddisp.pl?gene=MIR9-1 |
| HLA-DPB1 | Major Histocompatibility Complex, Class II, DP Beta 1 | Protein Coding | 44 | GC06P055237 | 1.385457516 | https://www.genecards.org/cgi-bin/carddisp.pl?gene=HLA-DPB1 |
| MECP2 | Methyl-CpG Binding Protein 2 | Protein Coding | 46 | GC0XM154021 | 1.381322265 | https://www.genecards.org/cgi-bin/carddisp.pl?gene=MECP2 |
| PAFAH1B1 | Platelet Activating Factor Acetylhydrolase 1b Regulatory Subunit 1 | Protein Coding | 45 | GC17P002593 | 1.372342587 | https://www.genecards.org/cgi-bin/carddisp.pl?gene=PAFAH1B1 |
| CACNA1I | Calcium Voltage-Gated Channel Subunit Alpha1 I | Protein Coding | 44 | GC22P039570 | 1.366850257 | https://www.genecards.org/cgi-bin/carddisp.pl?gene=CACNA1I |
| EGFR | Epidermal Growth Factor Receptor | Protein Coding | 54 | GC07P055019 | 1.356528401 | https://www.genecards.org/cgi-bin/carddisp.pl?gene=EGFR |
| PSMC4 | Proteasome 26S Subunit, ATPase 4 | Protein Coding | 41 | GC19P040529 | 1.352404952 | https://www.genecards.org/cgi-bin/carddisp.pl?gene=PSMC4 |
| TBK1 | TANK Binding Kinase 1 | Protein Coding | 50 | GC12P064451 | 1.35222888 | https://www.genecards.org/cgi-bin/carddisp.pl?gene=TBK1 |
| ISG15 | ISG15 Ubiquitin Like Modifier | Protein Coding | 47 | GC01P001001 | 1.35222888 | https://www.genecards.org/cgi-bin/carddisp.pl?gene=ISG15 |
| ADAR | Adenosine Deaminase RNA Specific | Protein Coding | 45 | GC01M154582 | 1.35222888 | https://www.genecards.org/cgi-bin/carddisp.pl?gene=ADAR |
| DNASE1L3 | Deoxyribonuclease 1 Like 3 | Protein Coding | 44 | GC03M058192 | 1.35222888 | https://www.genecards.org/cgi-bin/carddisp.pl?gene=DNASE1L3 |
| RNASEH2A | Ribonuclease H2 Subunit A | Protein Coding | 44 | GC19P012896 | 1.35222888 | https://www.genecards.org/cgi-bin/carddisp.pl?gene=RNASEH2A |
| IFIT1 | Interferon Induced Protein With Tetratricopeptide Repeats 1 | Protein Coding | 40 | GC10P089396 | 1.35222888 | https://www.genecards.org/cgi-bin/carddisp.pl?gene=IFIT1 |
| RNASEH2C | Ribonuclease H2 Subunit C | Protein Coding | 40 | GC11M065714 | 1.35222888 | https://www.genecards.org/cgi-bin/carddisp.pl?gene=RNASEH2C |
| RNASEH2B | Ribonuclease H2 Subunit B | Protein Coding | 37 | GC13P050909 | 1.35222888 | https://www.genecards.org/cgi-bin/carddisp.pl?gene=RNASEH2B |
| ARMC8 | Armadillo Repeat Containing 8 | Protein Coding | 34 | GC03P138187 | 1.35222888 | https://www.genecards.org/cgi-bin/carddisp.pl?gene=ARMC8 |
| ZNF621 | Zinc Finger Protein 621 | Protein Coding | 34 | GC03P040524 | 1.35222888 | https://www.genecards.org/cgi-bin/carddisp.pl?gene=ZNF621 |
| STING1 | Stimulator Of Interferon Response CGAMP Interactor 1 | Protein Coding | 34 | GC05M139476 | 1.35222888 | https://www.genecards.org/cgi-bin/carddisp.pl?gene=STING1 |
| KLHDC7A | Kelch Domain Containing 7A | Protein Coding | 31 | GC01P018480 | 1.35222888 | https://www.genecards.org/cgi-bin/carddisp.pl?gene=KLHDC7A |
| CGAS | Cyclic GMP-AMP Synthase | Protein Coding | 30 | GC06M073414 | 1.35222888 | https://www.genecards.org/cgi-bin/carddisp.pl?gene=CGAS |
| IL2RA | Interleukin 2 Receptor Subunit Alpha | Protein Coding | 51 | GC10M006010 | 1.349249005 | https://www.genecards.org/cgi-bin/carddisp.pl?gene=IL2RA |
| THBD | Thrombomodulin | Protein Coding | 44 | GC20M023026 | 1.349249005 | https://www.genecards.org/cgi-bin/carddisp.pl?gene=THBD |
| HLA-C | Major Histocompatibility Complex, Class I, C | Protein Coding | 45 | GC06M046831 | 1.334610462 | https://www.genecards.org/cgi-bin/carddisp.pl?gene=HLA-C |
| HLA-G | Major Histocompatibility Complex, Class I, G | Protein Coding | 45 | GC06P055150 | 1.334610462 | https://www.genecards.org/cgi-bin/carddisp.pl?gene=HLA-G |
| HLA-E | Major Histocompatibility Complex, Class I, E | Protein Coding | 42 | GC06P055176 | 1.334610462 | https://www.genecards.org/cgi-bin/carddisp.pl?gene=HLA-E |
| F3 | Coagulation Factor III, Tissue Factor | Protein Coding | 45 | GC01M094530 | 1.332778931 | https://www.genecards.org/cgi-bin/carddisp.pl?gene=F3 |
| HCRTR1 | Hypocretin Receptor 1 | Protein Coding | 44 | GC01P031587 | 1.324208617 | https://www.genecards.org/cgi-bin/carddisp.pl?gene=HCRTR1 |
| LOC110973015 | NOS3 5' Regulatory Region | Biological Region | 2 | GC07P150988 | 1.324208617 | https://www.genecards.org/cgi-bin/carddisp.pl?gene=LOC110973015 |
| SLC1A1 | Solute Carrier Family 1 Member 1 | Protein Coding | 48 | GC09P004490 | 1.30185771 | https://www.genecards.org/cgi-bin/carddisp.pl?gene=SLC1A1 |
| DNA2 | DNA Replication Helicase/Nuclease 2 | Protein Coding | 42 | GC10M068414 | 1.293540478 | https://www.genecards.org/cgi-bin/carddisp.pl?gene=DNA2 |
| MS4A2 | Membrane Spanning 4-Domains A2 | Protein Coding | 42 | GC11P060088 | 1.288421392 | https://www.genecards.org/cgi-bin/carddisp.pl?gene=MS4A2 |
| GABRR1 | Gamma-Aminobutyric Acid Type A Receptor Subunit Rho1 | Protein Coding | 43 | GC06M089177 | 1.270432115 | https://www.genecards.org/cgi-bin/carddisp.pl?gene=GABRR1 |
| CAT | Catalase | Protein Coding | 50 | GC11P034460 | 1.254196167 | https://www.genecards.org/cgi-bin/carddisp.pl?gene=CAT |
| ALDH2 | Aldehyde Dehydrogenase 2 Family Member | Protein Coding | 51 | GC12P111766 | 1.251676321 | https://www.genecards.org/cgi-bin/carddisp.pl?gene=ALDH2 |
| GABRA4 | Gamma-Aminobutyric Acid Type A Receptor Subunit Alpha4 | Protein Coding | 45 | GC04M046836 | 1.251676321 | https://www.genecards.org/cgi-bin/carddisp.pl?gene=GABRA4 |
| DAO | D-Amino Acid Oxidase | Protein Coding | 45 | GC12P108859 | 1.251676321 | https://www.genecards.org/cgi-bin/carddisp.pl?gene=DAO |
| ANK3 | Ankyrin 3 | Protein Coding | 44 | GC10M060026 | 1.251676321 | https://www.genecards.org/cgi-bin/carddisp.pl?gene=ANK3 |
| AGTR2 | Angiotensin II Receptor Type 2 | Protein Coding | 44 | GC0XP116170 | 1.240678668 | https://www.genecards.org/cgi-bin/carddisp.pl?gene=AGTR2 |
| ERCC2 | ERCC Excision Repair 2, TFIIH Core Complex Helicase Subunit | Protein Coding | 48 | GC19M045349 | 1.232046843 | https://www.genecards.org/cgi-bin/carddisp.pl?gene=ERCC2 |
| APEX1 | Apurinic/Apyrimidinic Endodeoxyribonuclease 1 | Protein Coding | 45 | GC14P020455 | 1.232046843 | https://www.genecards.org/cgi-bin/carddisp.pl?gene=APEX1 |
| MEIS1 | Meis Homeobox 1 | Protein Coding | 43 | GC02P066433 | 1.232046843 | https://www.genecards.org/cgi-bin/carddisp.pl?gene=MEIS1 |
| CACNA1D | Calcium Voltage-Gated Channel Subunit Alpha1 D | Protein Coding | 48 | GC03P053328 | 1.225227356 | https://www.genecards.org/cgi-bin/carddisp.pl?gene=CACNA1D |
| RNF138 | Ring Finger Protein 138 | Protein Coding | 40 | GC18P032091 | 1.225227356 | https://www.genecards.org/cgi-bin/carddisp.pl?gene=RNF138 |
| CLDN5 | Claudin 5 | Protein Coding | 43 | GC22M019523 | 1.211408854 | https://www.genecards.org/cgi-bin/carddisp.pl?gene=CLDN5 |
| CD160 | CD160 Molecule | Protein Coding | 40 | GC01P145719 | 1.211408854 | https://www.genecards.org/cgi-bin/carddisp.pl?gene=CD160 |
| ESM1 | Endothelial Cell Specific Molecule 1 | Protein Coding | 40 | GC05M054977 | 1.211408854 | https://www.genecards.org/cgi-bin/carddisp.pl?gene=ESM1 |
| VWA8 | Von Willebrand Factor A Domain Containing 8 | Protein Coding | 30 | GC13M041569 | 1.211408854 | https://www.genecards.org/cgi-bin/carddisp.pl?gene=VWA8 |
| C1S | Complement C1s | Protein Coding | 47 | GC12P011876 | 1.205601454 | https://www.genecards.org/cgi-bin/carddisp.pl?gene=C1S |
| SUCLA2 | Succinate-CoA Ligase ADP-Forming Subunit Beta | Protein Coding | 47 | GC13M047745 | 1.200616717 | https://www.genecards.org/cgi-bin/carddisp.pl?gene=SUCLA2 |
| SUCLG1 | Succinate-CoA Ligase GDP/ADP-Forming Subunit Alpha | Protein Coding | 46 | GC02M084423 | 1.200616717 | https://www.genecards.org/cgi-bin/carddisp.pl?gene=SUCLG1 |
| MPV17 | Mitochondrial Inner Membrane Protein MPV17 | Protein Coding | 42 | GC02M027309 | 1.200616717 | https://www.genecards.org/cgi-bin/carddisp.pl?gene=MPV17 |
| SSBP1 | Single Stranded DNA Binding Protein 1 | Protein Coding | 40 | GC07P146002 | 1.200616717 | https://www.genecards.org/cgi-bin/carddisp.pl?gene=SSBP1 |
| MGME1 | Mitochondrial Genome Maintenance Exonuclease 1 | Protein Coding | 35 | GC20P017952 | 1.200616717 | https://www.genecards.org/cgi-bin/carddisp.pl?gene=MGME1 |
| NAMPT | Nicotinamide Phosphoribosyltransferase | Protein Coding | 48 | GC07M106248 | 1.19703114 | https://www.genecards.org/cgi-bin/carddisp.pl?gene=NAMPT |
| CD70 | CD70 Molecule | Protein Coding | 43 | GC19M006583 | 1.190670252 | https://www.genecards.org/cgi-bin/carddisp.pl?gene=CD70 |
| HEPH | Hephaestin | Protein Coding | 39 | GC0XP066162 | 1.189588547 | https://www.genecards.org/cgi-bin/carddisp.pl?gene=HEPH |
| MDFI | MyoD Family Inhibitor | Protein Coding | 36 | GC06P055387 | 1.189588547 | https://www.genecards.org/cgi-bin/carddisp.pl?gene=MDFI |
| FLJ16779 | Uncharacterized LOC100192386 | RNA Gene | 12 | GC20P063253 | 1.189588547 | https://www.genecards.org/cgi-bin/carddisp.pl?gene=FLJ16779 |
| SLC1A4 | Solute Carrier Family 1 Member 4 | Protein Coding | 45 | GC02P064988 | 1.167022467 | https://www.genecards.org/cgi-bin/carddisp.pl?gene=SLC1A4 |
| ROCK2 | Rho Associated Coiled-Coil Containing Protein Kinase 2 | Protein Coding | 48 | GC02M011227 | 1.166356325 | https://www.genecards.org/cgi-bin/carddisp.pl?gene=ROCK2 |
| PRDX2 | Peroxiredoxin 2 | Protein Coding | 47 | GC19M012796 | 1.166356325 | https://www.genecards.org/cgi-bin/carddisp.pl?gene=PRDX2 |
| ACSL5 | Acyl-CoA Synthetase Long Chain Family Member 5 | Protein Coding | 43 | GC10P112374 | 1.166356325 | https://www.genecards.org/cgi-bin/carddisp.pl?gene=ACSL5 |
| AOC1 | Amine Oxidase Copper Containing 1 | Protein Coding | 40 | GC07P150824 | 1.166356325 | https://www.genecards.org/cgi-bin/carddisp.pl?gene=AOC1 |
| TXNDC9 | Thioredoxin Domain Containing 9 | Protein Coding | 37 | GC02M099301 | 1.166356325 | https://www.genecards.org/cgi-bin/carddisp.pl?gene=TXNDC9 |
| LOC111365141 | NOS2 5' Regulatory Region | Biological Region | 2 | GC17P027800 | 1.166356325 | https://www.genecards.org/cgi-bin/carddisp.pl?gene=LOC111365141 |
| TGFB2 | Transforming Growth Factor Beta 2 | Protein Coding | 50 | GC01P218345 | 1.152073383 | https://www.genecards.org/cgi-bin/carddisp.pl?gene=TGFB2 |
| GRIA2 | Glutamate Ionotropic Receptor AMPA Type Subunit 2 | Protein Coding | 51 | GC04P157204 | 1.141396284 | https://www.genecards.org/cgi-bin/carddisp.pl?gene=GRIA2 |
| GRIA4 | Glutamate Ionotropic Receptor AMPA Type Subunit 4 | Protein Coding | 50 | GC11P105609 | 1.141396284 | https://www.genecards.org/cgi-bin/carddisp.pl?gene=GRIA4 |
| ZEB2 | Zinc Finger E-Box Binding Homeobox 2 | Protein Coding | 48 | GC02M144384 | 1.141396284 | https://www.genecards.org/cgi-bin/carddisp.pl?gene=ZEB2 |
| PRNP | Prion Protein | Protein Coding | 47 | GC20P004686 | 1.141396284 | https://www.genecards.org/cgi-bin/carddisp.pl?gene=PRNP |
| ANKK1 | Ankyrin Repeat And Kinase Domain Containing 1 | Protein Coding | 38 | GC11P113387 | 1.141396284 | https://www.genecards.org/cgi-bin/carddisp.pl?gene=ANKK1 |
| CASP1 | Caspase 1 | Protein Coding | 51 | GC11M105025 | 1.128169656 | https://www.genecards.org/cgi-bin/carddisp.pl?gene=CASP1 |
| IL2 | Interleukin 2 | Protein Coding | 45 | GC04M122451 | 1.117819548 | https://www.genecards.org/cgi-bin/carddisp.pl?gene=IL2 |
| TF | Transferrin | Protein Coding | 50 | GC03P133666 | 1.114253879 | https://www.genecards.org/cgi-bin/carddisp.pl?gene=TF |
| CACNA2D1 | Calcium Voltage-Gated Channel Auxiliary Subunit Alpha2delta 1 | Protein Coding | 47 | GC07M081946 | 1.114253879 | https://www.genecards.org/cgi-bin/carddisp.pl?gene=CACNA2D1 |
| SLC5A6 | Solute Carrier Family 5 Member 6 | Protein Coding | 47 | GC02M027201 | 1.114253879 | https://www.genecards.org/cgi-bin/carddisp.pl?gene=SLC5A6 |
| SYNE1 | Spectrin Repeat Containing Nuclear Envelope Protein 1 | Protein Coding | 41 | GC06M152121 | 1.114253879 | https://www.genecards.org/cgi-bin/carddisp.pl?gene=SYNE1 |
| FRAXA | Fragile Site, Folic Acid Type, Rare, Fra(X)(Q27.3) A (Macroorchidism, Mental Retardation) | Biological Region | 6 | GC0XP147926 | 1.114253879 | https://www.genecards.org/cgi-bin/carddisp.pl?gene=FRAXA |
| LOC101927066 | Uncharacterized LOC101927066 | RNA Gene | 6 | GC08M096951 | 1.114253879 | https://www.genecards.org/cgi-bin/carddisp.pl?gene=LOC101927066 |
| NF2 | Neurofibromin 2 | Protein Coding | 49 | GC22P029603 | 1.090392113 | https://www.genecards.org/cgi-bin/carddisp.pl?gene=NF2 |
| PPP2R2B | Protein Phosphatase 2 Regulatory Subunit Bbeta | Protein Coding | 47 | GC05M146582 | 1.090392113 | https://www.genecards.org/cgi-bin/carddisp.pl?gene=PPP2R2B |
| ACADVL | Acyl-CoA Dehydrogenase Very Long Chain | Protein Coding | 46 | GC17P007219 | 1.090392113 | https://www.genecards.org/cgi-bin/carddisp.pl?gene=ACADVL |
| PLP1 | Proteolipid Protein 1 | Protein Coding | 44 | GC0XP103773 | 1.090392113 | https://www.genecards.org/cgi-bin/carddisp.pl?gene=PLP1 |
| PGM1 | Phosphoglucomutase 1 | Protein Coding | 49 | GC01P063593 | 1.084231257 | https://www.genecards.org/cgi-bin/carddisp.pl?gene=PGM1 |
| GNAI1 | G Protein Subunit Alpha I1 | Protein Coding | 48 | GC07P079769 | 1.084231257 | https://www.genecards.org/cgi-bin/carddisp.pl?gene=GNAI1 |
| BDKRB2 | Bradykinin Receptor B2 | Protein Coding | 45 | GC14P096205 | 1.084231257 | https://www.genecards.org/cgi-bin/carddisp.pl?gene=BDKRB2 |
| ACP1 | Acid Phosphatase 1 | Protein Coding | 44 | GC02P000254 | 1.084231257 | https://www.genecards.org/cgi-bin/carddisp.pl?gene=ACP1 |
| CDKN3 | Cyclin Dependent Kinase Inhibitor 3 | Protein Coding | 43 | GC14P054398 | 1.084231257 | https://www.genecards.org/cgi-bin/carddisp.pl?gene=CDKN3 |
| NTS | Neurotensin | Protein Coding | 41 | GC12P085876 | 1.084231257 | https://www.genecards.org/cgi-bin/carddisp.pl?gene=NTS |
| SCP2 | Sterol Carrier Protein 2 | Protein Coding | 48 | GC01P052927 | 1.081579804 | https://www.genecards.org/cgi-bin/carddisp.pl?gene=SCP2 |
| MSX2 | Msh Homeobox 2 | Protein Coding | 48 | GC05P174724 | 1.081579804 | https://www.genecards.org/cgi-bin/carddisp.pl?gene=MSX2 |
| HSD17B4 | Hydroxysteroid 17-Beta Dehydrogenase 4 | Protein Coding | 47 | GC05P119452 | 1.081579804 | https://www.genecards.org/cgi-bin/carddisp.pl?gene=HSD17B4 |
| AKR1D1 | Aldo-Keto Reductase Family 1 Member D1 | Protein Coding | 47 | GC07P138003 | 1.081579804 | https://www.genecards.org/cgi-bin/carddisp.pl?gene=AKR1D1 |
| ACOX1 | Acyl-CoA Oxidase 1 | Protein Coding | 47 | GC17M075941 | 1.081579804 | https://www.genecards.org/cgi-bin/carddisp.pl?gene=ACOX1 |
| ACOX2 | Acyl-CoA Oxidase 2 | Protein Coding | 44 | GC03M058490 | 1.081579804 | https://www.genecards.org/cgi-bin/carddisp.pl?gene=ACOX2 |
| SLC1A6 | Solute Carrier Family 1 Member 6 | Protein Coding | 44 | GC19M014921 | 1.081579804 | https://www.genecards.org/cgi-bin/carddisp.pl?gene=SLC1A6 |
| PEX6 | Peroxisomal Biogenesis Factor 6 | Protein Coding | 43 | GC06M042963 | 1.081579804 | https://www.genecards.org/cgi-bin/carddisp.pl?gene=PEX6 |
| ACOX3 | Acyl-CoA Oxidase 3, Pristanoyl | Protein Coding | 41 | GC04M008380 | 1.081579804 | https://www.genecards.org/cgi-bin/carddisp.pl?gene=ACOX3 |
| HACL1 | 2-Hydroxyacyl-CoA Lyase 1 | Protein Coding | 40 | GC03M016952 | 1.081579804 | https://www.genecards.org/cgi-bin/carddisp.pl?gene=HACL1 |
| SLC45A2 | Solute Carrier Family 45 Member 2 | Protein Coding | 40 | GC05M033944 | 1.081579804 | https://www.genecards.org/cgi-bin/carddisp.pl?gene=SLC45A2 |
| ACOT8 | Acyl-CoA Thioesterase 8 | Protein Coding | 39 | GC20M045841 | 1.081579804 | https://www.genecards.org/cgi-bin/carddisp.pl?gene=ACOT8 |
| PEX16 | Peroxisomal Biogenesis Factor 16 | Protein Coding | 39 | GC11M068965 | 1.081579804 | https://www.genecards.org/cgi-bin/carddisp.pl?gene=PEX16 |
| PXMP4 | Peroxisomal Membrane Protein 4 | Protein Coding | 35 | GC20M033734 | 1.081579804 | https://www.genecards.org/cgi-bin/carddisp.pl?gene=PXMP4 |
| TMEM176A | Transmembrane Protein 176A | Protein Coding | 34 | GC07P150800 | 1.081579804 | https://www.genecards.org/cgi-bin/carddisp.pl?gene=TMEM176A |
| SERF1A | Small EDRK-Rich Factor 1A | Protein Coding | 31 | GC05P070900 | 1.081579804 | https://www.genecards.org/cgi-bin/carddisp.pl?gene=SERF1A |
| SERF1B | Small EDRK-Rich Factor 1B | Protein Coding | 29 | GC05P070025 | 1.081579804 | https://www.genecards.org/cgi-bin/carddisp.pl?gene=SERF1B |
| CDKL5 | Cyclin Dependent Kinase Like 5 | Protein Coding | 44 | GC0XP018425 | 1.073922038 | https://www.genecards.org/cgi-bin/carddisp.pl?gene=CDKL5 |
| IRF4 | Interferon Regulatory Factor 4 | Protein Coding | 44 | GC06P000391 | 1.054030418 | https://www.genecards.org/cgi-bin/carddisp.pl?gene=IRF4 |
| ELANE | Elastase, Neutrophil Expressed | Protein Coding | 47 | GC19P000855 | 1.052198768 | https://www.genecards.org/cgi-bin/carddisp.pl?gene=ELANE |
| PSAT1 | Phosphoserine Aminotransferase 1 | Protein Coding | 48 | GC09P078297 | 1.050154209 | https://www.genecards.org/cgi-bin/carddisp.pl?gene=PSAT1 |
| RETN | Resistin | Protein Coding | 44 | GC19P007669 | 1.050154209 | https://www.genecards.org/cgi-bin/carddisp.pl?gene=RETN |
| DBT | Dihydrolipoamide Branched Chain Transacylase E2 | Protein Coding | 43 | GC01M100186 | 1.050154209 | https://www.genecards.org/cgi-bin/carddisp.pl?gene=DBT |
| EPN1 | Epsin 1 | Protein Coding | 41 | GC19P056494 | 1.050154209 | https://www.genecards.org/cgi-bin/carddisp.pl?gene=EPN1 |
| LOC101927558 | Uncharacterized LOC101927558 | RNA Gene | 1 | GC07P015715 | 1.050154209 | https://www.genecards.org/cgi-bin/carddisp.pl?gene=LOC101927558 |
| TGFBR1 | Transforming Growth Factor Beta Receptor 1 | Protein Coding | 54 | GC09P099104 | 1.041848779 | https://www.genecards.org/cgi-bin/carddisp.pl?gene=TGFBR1 |
| ACTA2 | Actin Alpha 2, Smooth Muscle | Protein Coding | 49 | GC10M088935 | 1.041848779 | https://www.genecards.org/cgi-bin/carddisp.pl?gene=ACTA2 |
| IFNG | Interferon Gamma | Protein Coding | 48 | GC12M068154 | 1.041848779 | https://www.genecards.org/cgi-bin/carddisp.pl?gene=IFNG |
| CCND1 | Cyclin D1 | Protein Coding | 52 | GC11P069641 | 1.035245895 | https://www.genecards.org/cgi-bin/carddisp.pl?gene=CCND1 |
| FN1 | Fibronectin 1 | Protein Coding | 50 | GC02M215360 | 1.034526348 | https://www.genecards.org/cgi-bin/carddisp.pl?gene=FN1 |
| TLR2 | Toll Like Receptor 2 | Protein Coding | 52 | GC04P153684 | 1.024895906 | https://www.genecards.org/cgi-bin/carddisp.pl?gene=TLR2 |
| CD4 | CD4 Molecule | Protein Coding | 50 | GC12P006786 | 1.024895906 | https://www.genecards.org/cgi-bin/carddisp.pl?gene=CD4 |
| NCAM1 | Neural Cell Adhesion Molecule 1 | Protein Coding | 47 | GC11P112961 | 1.024895906 | https://www.genecards.org/cgi-bin/carddisp.pl?gene=NCAM1 |
| COL5A1 | Collagen Type V Alpha 1 Chain | Protein Coding | 46 | GC09P134641 | 1.024895906 | https://www.genecards.org/cgi-bin/carddisp.pl?gene=COL5A1 |
| SYP | Synaptophysin | Protein Coding | 44 | GC0XM049187 | 1.024895906 | https://www.genecards.org/cgi-bin/carddisp.pl?gene=SYP |
| ATXN2 | Ataxin 2 | Protein Coding | 43 | GC12M111443 | 1.024895906 | https://www.genecards.org/cgi-bin/carddisp.pl?gene=ATXN2 |
| COQ2 | Coenzyme Q2, Polyprenyltransferase | Protein Coding | 41 | GC04M083261 | 1.024895906 | https://www.genecards.org/cgi-bin/carddisp.pl?gene=COQ2 |
| ICOSLG | Inducible T Cell Costimulator Ligand | Protein Coding | 39 | GC21M044222 | 1.024895906 | https://www.genecards.org/cgi-bin/carddisp.pl?gene=ICOSLG |
| MRRF | Mitochondrial Ribosome Recycling Factor | Protein Coding | 39 | GC09P122264 | 1.024895906 | https://www.genecards.org/cgi-bin/carddisp.pl?gene=MRRF |
| MYOM2 | Myomesin 2 | Protein Coding | 38 | GC08P002045 | 1.024895906 | https://www.genecards.org/cgi-bin/carddisp.pl?gene=MYOM2 |
| ATXN8OS | ATXN8 Opposite Strand LncRNA | RNA Gene | 26 | GC13P070107 | 1.024895906 | https://www.genecards.org/cgi-bin/carddisp.pl?gene=ATXN8OS |
| NFKB1 | Nuclear Factor Kappa B Subunit 1 | Protein Coding | 52 | GC04P102501 | 1.009732485 | https://www.genecards.org/cgi-bin/carddisp.pl?gene=NFKB1 |
| ITGB2 | Integrin Subunit Beta 2 | Protein Coding | 51 | GC21M044885 | 1.009732485 | https://www.genecards.org/cgi-bin/carddisp.pl?gene=ITGB2 |
| ITGA4 | Integrin Subunit Alpha 4 | Protein Coding | 49 | GC02P181456 | 1.009732485 | https://www.genecards.org/cgi-bin/carddisp.pl?gene=ITGA4 |
| TPT1 | Tumor Protein, Translationally-Controlled 1 | Protein Coding | 47 | GC13M045333 | 1.009732485 | https://www.genecards.org/cgi-bin/carddisp.pl?gene=TPT1 |
| LOC111258525 | NOS1 1f And 1g Alternate Promoter Region | Biological Region | 1 | GC12P117361 | 1.009732485 | https://www.genecards.org/cgi-bin/carddisp.pl?gene=LOC111258525 |
| SLC5A3 | Solute Carrier Family 5 Member 3 | Protein Coding | 44 | GC21P034119 | 0.998164654 | https://www.genecards.org/cgi-bin/carddisp.pl?gene=SLC5A3 |
| NFKBIB | NFKB Inhibitor Beta | Protein Coding | 43 | GC19P038899 | 0.998164654 | https://www.genecards.org/cgi-bin/carddisp.pl?gene=NFKBIB |
| COQ8A | Coenzyme Q8A | Protein Coding | 36 | GC01P226939 | 0.991301537 | https://www.genecards.org/cgi-bin/carddisp.pl?gene=COQ8A |
| ICA1L | Islet Cell Autoantigen 1 Like | Protein Coding | 36 | GC02M202807 | 0.987814546 | https://www.genecards.org/cgi-bin/carddisp.pl?gene=ICA1L |
| NAB2 | NGFI-A Binding Protein 2 | Protein Coding | 41 | GC12P057088 | 0.976161122 | https://www.genecards.org/cgi-bin/carddisp.pl?gene=NAB2 |
| NXPH4 | Neurexophilin 4 | Protein Coding | 37 | GC12P057218 | 0.976161122 | https://www.genecards.org/cgi-bin/carddisp.pl?gene=NXPH4 |
| MBD6 | Methyl-CpG Binding Domain Protein 6 | Protein Coding | 36 | GC12P057520 | 0.976161122 | https://www.genecards.org/cgi-bin/carddisp.pl?gene=MBD6 |
| NABP2 | Nucleic Acid Binding Protein 2 | Protein Coding | 36 | GC12P056398 | 0.976161122 | https://www.genecards.org/cgi-bin/carddisp.pl?gene=NABP2 |
| LRP1-AS | LRP1 Antisense RNA | RNA Gene | 11 | GC12M057145 | 0.976161122 | https://www.genecards.org/cgi-bin/carddisp.pl?gene=LRP1-AS |
| HSPD1P4 | Heat Shock Protein Family D (Hsp60) Member 1 Pseudogene 4 | Pseudogene | 7 | GC12P056617 | 0.976161122 | https://www.genecards.org/cgi-bin/carddisp.pl?gene=HSPD1P4 |
| OTC | Ornithine Transcarbamylase | Protein Coding | 48 | GC0XP038353 | 0.96122992 | https://www.genecards.org/cgi-bin/carddisp.pl?gene=OTC |
| SLCO1A2 | Solute Carrier Organic Anion Transporter Family Member 1A2 | Protein Coding | 40 | GC12M021264 | 0.957053781 | https://www.genecards.org/cgi-bin/carddisp.pl?gene=SLCO1A2 |
| CREBBP | CREB Binding Protein | Protein Coding | 52 | GC16M003745 | 0.946744442 | https://www.genecards.org/cgi-bin/carddisp.pl?gene=CREBBP |
| HPD | 4-Hydroxyphenylpyruvate Dioxygenase | Protein Coding | 46 | GC12M121839 | 0.946744442 | https://www.genecards.org/cgi-bin/carddisp.pl?gene=HPD |
| ALS2 | Alsin Rho Guanine Nucleotide Exchange Factor ALS2 | Protein Coding | 45 | GC02M201701 | 0.946744442 | https://www.genecards.org/cgi-bin/carddisp.pl?gene=ALS2 |
| CLCN6 | Chloride Voltage-Gated Channel 6 | Protein Coding | 43 | GC01P011806 | 0.946744442 | https://www.genecards.org/cgi-bin/carddisp.pl?gene=CLCN6 |
| MYH1 | Myosin Heavy Chain 1 | Protein Coding | 41 | GC17M010492 | 0.946744442 | https://www.genecards.org/cgi-bin/carddisp.pl?gene=MYH1 |
| KIF7 | Kinesin Family Member 7 | Protein Coding | 40 | GC15M089608 | 0.946744442 | https://www.genecards.org/cgi-bin/carddisp.pl?gene=KIF7 |
| STOM | Stomatin | Protein Coding | 40 | GC09M121338 | 0.946744442 | https://www.genecards.org/cgi-bin/carddisp.pl?gene=STOM |
| RAB9B | RAB9B, Member RAS Oncogene Family | Protein Coding | 36 | GC0XM103777 | 0.946744442 | https://www.genecards.org/cgi-bin/carddisp.pl?gene=RAB9B |
| BUD23 | BUD23 RRNA Methyltransferase And Ribosome Maturation Factor | Protein Coding | 31 | GC07P073686 | 0.946744442 | https://www.genecards.org/cgi-bin/carddisp.pl?gene=BUD23 |
| H2AC12 | H2A Clustered Histone 12 | Protein Coding | 28 | GC06P055578 | 0.946744442 | https://www.genecards.org/cgi-bin/carddisp.pl?gene=H2AC12 |
| HDAC9 | Histone Deacetylase 9 | Protein Coding | 47 | GC07P018086 | 0.927178502 | https://www.genecards.org/cgi-bin/carddisp.pl?gene=HDAC9 |
| ABAT | 4-Aminobutyrate Aminotransferase | Protein Coding | 46 | GC16P008674 | 0.927178502 | https://www.genecards.org/cgi-bin/carddisp.pl?gene=ABAT |
| JAK2 | Janus Kinase 2 | Protein Coding | 54 | GC09P004985 | 0.925021291 | https://www.genecards.org/cgi-bin/carddisp.pl?gene=JAK2 |
| MAPT | Microtubule Associated Protein Tau | Protein Coding | 51 | GC17P045894 | 0.925021291 | https://www.genecards.org/cgi-bin/carddisp.pl?gene=MAPT |
| IL1R1 | Interleukin 1 Receptor Type 1 | Protein Coding | 46 | GC02P102136 | 0.925021291 | https://www.genecards.org/cgi-bin/carddisp.pl?gene=IL1R1 |
| BMP6 | Bone Morphogenetic Protein 6 | Protein Coding | 44 | GC06P007726 | 0.925021291 | https://www.genecards.org/cgi-bin/carddisp.pl?gene=BMP6 |
| NPPB | Natriuretic Peptide B | Protein Coding | 44 | GC01M011858 | 0.925021291 | https://www.genecards.org/cgi-bin/carddisp.pl?gene=NPPB |
| RPS27A | Ribosomal Protein S27a | Protein Coding | 44 | GC02P055231 | 0.925021291 | https://www.genecards.org/cgi-bin/carddisp.pl?gene=RPS27A |
| SELL | Selectin L | Protein Coding | 43 | GC01M169690 | 0.925021291 | https://www.genecards.org/cgi-bin/carddisp.pl?gene=SELL |
| PTPRC | Protein Tyrosine Phosphatase Receptor Type C | Protein Coding | 51 | GC01P198607 | 0.914671183 | https://www.genecards.org/cgi-bin/carddisp.pl?gene=PTPRC |
| FOXP3 | Forkhead Box P3 | Protein Coding | 47 | GC0XM049250 | 0.914671183 | https://www.genecards.org/cgi-bin/carddisp.pl?gene=FOXP3 |
| ITPR3 | Inositol 1,4,5-Trisphosphate Receptor Type 3 | Protein Coding | 47 | GC06P033620 | 0.914671183 | https://www.genecards.org/cgi-bin/carddisp.pl?gene=ITPR3 |
| ERCC6 | ERCC Excision Repair 6, Chromatin Remodeling Factor | Protein Coding | 46 | GC10M049454 | 0.914671183 | https://www.genecards.org/cgi-bin/carddisp.pl?gene=ERCC6 |
| MYH11 | Myosin Heavy Chain 11 | Protein Coding | 46 | GC16M015704 | 0.914671183 | https://www.genecards.org/cgi-bin/carddisp.pl?gene=MYH11 |
| CCR6 | C-C Motif Chemokine Receptor 6 | Protein Coding | 45 | GC06P167111 | 0.914671183 | https://www.genecards.org/cgi-bin/carddisp.pl?gene=CCR6 |
| SURF1 | SURF1 Cytochrome C Oxidase Assembly Factor | Protein Coding | 44 | GC09M133351 | 0.914671183 | https://www.genecards.org/cgi-bin/carddisp.pl?gene=SURF1 |
| SPG7 | SPG7 Matrix AAA Peptidase Subunit, Paraplegin | Protein Coding | 44 | GC16P089790 | 0.914671183 | https://www.genecards.org/cgi-bin/carddisp.pl?gene=SPG7 |
| IL17A | Interleukin 17A | Protein Coding | 43 | GC06P052186 | 0.914671183 | https://www.genecards.org/cgi-bin/carddisp.pl?gene=IL17A |
| CACNA2D2 | Calcium Voltage-Gated Channel Auxiliary Subunit Alpha2delta 2 | Protein Coding | 43 | GC03M050433 | 0.914671183 | https://www.genecards.org/cgi-bin/carddisp.pl?gene=CACNA2D2 |
| RARS2 | Arginyl-TRNA Synthetase 2, Mitochondrial | Protein Coding | 43 | GC06M087514 | 0.914671183 | https://www.genecards.org/cgi-bin/carddisp.pl?gene=RARS2 |
| PGK2 | Phosphoglycerate Kinase 2 | Protein Coding | 42 | GC06M049785 | 0.914671183 | https://www.genecards.org/cgi-bin/carddisp.pl?gene=PGK2 |
| NBEAL2 | Neurobeachin Like 2 | Protein Coding | 41 | GC03P046979 | 0.914671183 | https://www.genecards.org/cgi-bin/carddisp.pl?gene=NBEAL2 |
| TRMU | TRNA Mitochondrial 2-Thiouridylase | Protein Coding | 40 | GC22P046330 | 0.914671183 | https://www.genecards.org/cgi-bin/carddisp.pl?gene=TRMU |
| SH2D1B | SH2 Domain Containing 1B | Protein Coding | 40 | GC01M162395 | 0.914671183 | https://www.genecards.org/cgi-bin/carddisp.pl?gene=SH2D1B |
| EPPIN | Epididymal Peptidase Inhibitor | Protein Coding | 36 | GC20M045570 | 0.914671183 | https://www.genecards.org/cgi-bin/carddisp.pl?gene=EPPIN |
| C3orf20 | Chromosome 3 Open Reading Frame 20 | Protein Coding | 33 | GC03P014674 | 0.914671183 | https://www.genecards.org/cgi-bin/carddisp.pl?gene=C3orf20 |
| CATSPERD | Cation Channel Sperm Associated Auxiliary Subunit Delta | Protein Coding | 32 | GC19P005720 | 0.914671183 | https://www.genecards.org/cgi-bin/carddisp.pl?gene=CATSPERD |
| SPDYC | Speedy/RINGO Cell Cycle Regulator Family Member C | Protein Coding | 29 | GC11P065170 | 0.914671183 | https://www.genecards.org/cgi-bin/carddisp.pl?gene=SPDYC |
| RAD21L1 | RAD21 Cohesin Complex Component Like 1 | Protein Coding | 29 | GC20P001226 | 0.914671183 | https://www.genecards.org/cgi-bin/carddisp.pl?gene=RAD21L1 |
| ANKRD34C | Ankyrin Repeat Domain 34C | Protein Coding | 27 | GC15P079282 | 0.914671183 | https://www.genecards.org/cgi-bin/carddisp.pl?gene=ANKRD34C |
| C11orf94 | Chromosome 11 Open Reading Frame 94 | Protein Coding | 27 | GC11M068964 | 0.914671183 | https://www.genecards.org/cgi-bin/carddisp.pl?gene=C11orf94 |
| CFAP73 | Cilia And Flagella Associated Protein 73 | Protein Coding | 23 | GC12P113150 | 0.914671183 | https://www.genecards.org/cgi-bin/carddisp.pl?gene=CFAP73 |
| TDRD15 | Tudor Domain Containing 15 | Protein Coding | 23 | GC02P021123 | 0.914671183 | https://www.genecards.org/cgi-bin/carddisp.pl?gene=TDRD15 |
| MT-TD | Mitochondrially Encoded TRNA-Asp (GAU/C) | RNA Gene | 12 | GCMTP007520 | 0.914671183 | https://www.genecards.org/cgi-bin/carddisp.pl?gene=MT-TD |
| MT-TG | Mitochondrially Encoded TRNA-Gly (GGN) | RNA Gene | 12 | GCMTP009993 | 0.914671183 | https://www.genecards.org/cgi-bin/carddisp.pl?gene=MT-TG |
| MT-TR | Mitochondrially Encoded TRNA-Arg (CGN) | RNA Gene | 12 | GCMTP010407 | 0.914671183 | https://www.genecards.org/cgi-bin/carddisp.pl?gene=MT-TR |
| MT-TM | Mitochondrially Encoded TRNA-Met (AUA/G) | RNA Gene | 11 | GCMTP004404 | 0.914671183 | https://www.genecards.org/cgi-bin/carddisp.pl?gene=MT-TM |
| ERBB4 | Erb-B2 Receptor Tyrosine Kinase 4 | Protein Coding | 55 | GC02M211375 | 0.910382867 | https://www.genecards.org/cgi-bin/carddisp.pl?gene=ERBB4 |
| BCL2 | BCL2 Apoptosis Regulator | Protein Coding | 51 | GC18M063123 | 0.910382867 | https://www.genecards.org/cgi-bin/carddisp.pl?gene=BCL2 |
| PHB | Prohibitin | Protein Coding | 48 | GC17M049404 | 0.910382867 | https://www.genecards.org/cgi-bin/carddisp.pl?gene=PHB |
| RUNX2 | RUNX Family Transcription Factor 2 | Protein Coding | 48 | GC06P055427 | 0.910382867 | https://www.genecards.org/cgi-bin/carddisp.pl?gene=RUNX2 |
| BIRC3 | Baculoviral IAP Repeat Containing 3 | Protein Coding | 47 | GC11P102317 | 0.910382867 | https://www.genecards.org/cgi-bin/carddisp.pl?gene=BIRC3 |
| NLRP1 | NLR Family Pyrin Domain Containing 1 | Protein Coding | 45 | GC17M005499 | 0.910382867 | https://www.genecards.org/cgi-bin/carddisp.pl?gene=NLRP1 |
| ITPKC | Inositol-Trisphosphate 3-Kinase C | Protein Coding | 44 | GC19P041025 | 0.910382867 | https://www.genecards.org/cgi-bin/carddisp.pl?gene=ITPKC |
| MICA | MHC Class I Polypeptide-Related Sequence A | Protein Coding | 39 | GC06P031399 | 0.910382867 | https://www.genecards.org/cgi-bin/carddisp.pl?gene=MICA |
| AKAP7 | A-Kinase Anchoring Protein 7 | Protein Coding | 39 | GC06P131126 | 0.910382867 | https://www.genecards.org/cgi-bin/carddisp.pl?gene=AKAP7 |
| TRIM39 | Tripartite Motif Containing 39 | Protein Coding | 38 | GC06P055171 | 0.910382867 | https://www.genecards.org/cgi-bin/carddisp.pl?gene=TRIM39 |
| PSORS1C1 | Psoriasis Susceptibility 1 Candidate 1 | Protein Coding | 30 | GC06P031114 | 0.910382867 | https://www.genecards.org/cgi-bin/carddisp.pl?gene=PSORS1C1 |
| CASP3 | Caspase 3 | Protein Coding | 51 | GC04M184627 | 0.908551216 | https://www.genecards.org/cgi-bin/carddisp.pl?gene=CASP3 |
| FGA | Fibrinogen Alpha Chain | Protein Coding | 48 | GC04M154583 | 0.886756778 | https://www.genecards.org/cgi-bin/carddisp.pl?gene=FGA |
| IDO1 | Indoleamine 2,3-Dioxygenase 1 | Protein Coding | 45 | GC08P039891 | 0.861961424 | https://www.genecards.org/cgi-bin/carddisp.pl?gene=IDO1 |
| RPS6KA3 | Ribosomal Protein S6 Kinase A3 | Protein Coding | 53 | GC0XM020149 | 0.858805418 | https://www.genecards.org/cgi-bin/carddisp.pl?gene=RPS6KA3 |
| CALR | Calreticulin | Protein Coding | 51 | GC19P012938 | 0.858805418 | https://www.genecards.org/cgi-bin/carddisp.pl?gene=CALR |
| F10 | Coagulation Factor X | Protein Coding | 50 | GC13P113122 | 0.858805418 | https://www.genecards.org/cgi-bin/carddisp.pl?gene=F10 |
| PLAT | Plasminogen Activator, Tissue Type | Protein Coding | 50 | GC08M042174 | 0.858805418 | https://www.genecards.org/cgi-bin/carddisp.pl?gene=PLAT |
| SLC22A7 | Solute Carrier Family 22 Member 7 | Protein Coding | 43 | GC06P055408 | 0.858805418 | https://www.genecards.org/cgi-bin/carddisp.pl?gene=SLC22A7 |
| SULT1A3 | Sulfotransferase Family 1A Member 3 | Protein Coding | 38 | GC16P030199 | 0.858805418 | https://www.genecards.org/cgi-bin/carddisp.pl?gene=SULT1A3 |
| CRCP | CGRP Receptor Component | Protein Coding | 38 | GC07P066114 | 0.843667746 | https://www.genecards.org/cgi-bin/carddisp.pl?gene=CRCP |
| APOA1 | Apolipoprotein A1 | Protein Coding | 49 | GC11M116835 | 0.834863663 | https://www.genecards.org/cgi-bin/carddisp.pl?gene=APOA1 |
| COX5A | Cytochrome C Oxidase Subunit 5A | Protein Coding | 45 | GC15M074919 | 0.834078133 | https://www.genecards.org/cgi-bin/carddisp.pl?gene=COX5A |
| GNRH1 | Gonadotropin Releasing Hormone 1 | Protein Coding | 43 | GC08M025419 | 0.798974991 | https://www.genecards.org/cgi-bin/carddisp.pl?gene=GNRH1 |
| ITGB5 | Integrin Subunit Beta 5 | Protein Coding | 47 | GC03M124761 | 0.797032177 | https://www.genecards.org/cgi-bin/carddisp.pl?gene=ITGB5 |
| UMPS | Uridine Monophosphate Synthetase | Protein Coding | 47 | GC03P124730 | 0.797032177 | https://www.genecards.org/cgi-bin/carddisp.pl?gene=UMPS |
| KALRN | Kalirin RhoGEF Kinase | Protein Coding | 44 | GC03P124033 | 0.797032177 | https://www.genecards.org/cgi-bin/carddisp.pl?gene=KALRN |
| LYVE1 | Lymphatic Vessel Endothelial Hyaluronan Receptor 1 | Protein Coding | 43 | GC11M010713 | 0.797032177 | https://www.genecards.org/cgi-bin/carddisp.pl?gene=LYVE1 |
| CCT3 | Chaperonin Containing TCP1 Subunit 3 | Protein Coding | 42 | GC01M156308 | 0.797032177 | https://www.genecards.org/cgi-bin/carddisp.pl?gene=CCT3 |
| ZNF148 | Zinc Finger Protein 148 | Protein Coding | 41 | GC03M125225 | 0.797032177 | https://www.genecards.org/cgi-bin/carddisp.pl?gene=ZNF148 |
| FGF6 | Fibroblast Growth Factor 6 | Protein Coding | 40 | GC12M004429 | 0.797032177 | https://www.genecards.org/cgi-bin/carddisp.pl?gene=FGF6 |
| RHBG | Rh Family B Glycoprotein | Protein Coding | 40 | GC01P156366 | 0.797032177 | https://www.genecards.org/cgi-bin/carddisp.pl?gene=RHBG |
| SDR9C7 | Short Chain Dehydrogenase/Reductase Family 9C Member 7 | Protein Coding | 38 | GC12M056923 | 0.797032177 | https://www.genecards.org/cgi-bin/carddisp.pl?gene=SDR9C7 |
| HJURP | Holliday Junction Recognition Protein | Protein Coding | 37 | GC02M233834 | 0.797032177 | https://www.genecards.org/cgi-bin/carddisp.pl?gene=HJURP |
| UFL1 | UFM1 Specific Ligase 1 | Protein Coding | 34 | GC06P096521 | 0.797032177 | https://www.genecards.org/cgi-bin/carddisp.pl?gene=UFL1 |
| MTRNR2L8 | MT-RNR2 Like 8 | Protein Coding | 23 | GC11M010711 | 0.797032177 | https://www.genecards.org/cgi-bin/carddisp.pl?gene=MTRNR2L8 |
| MIR9-1HG | MIR9-1 Host Gene | RNA Gene | 22 | GC01M156408 | 0.797032177 | https://www.genecards.org/cgi-bin/carddisp.pl?gene=MIR9-1HG |
| MSL3P1 | MSL Complex Subunit 3 Pseudogene 1 | Pseudogene | 19 | GC02M233866 | 0.797032177 | https://www.genecards.org/cgi-bin/carddisp.pl?gene=MSL3P1 |
| IRAG1-AS1 | IRAG1 Antisense RNA 1 | RNA Gene | 12 | GC11P010541 | 0.797032177 | https://www.genecards.org/cgi-bin/carddisp.pl?gene=IRAG1-AS1 |
| ITGB5-AS1 | ITGB5 Antisense RNA 1 | RNA Gene | 9 | GC03P124781 | 0.797032177 | https://www.genecards.org/cgi-bin/carddisp.pl?gene=ITGB5-AS1 |
| TBC1D7-LOC100130357 | TBC1D7-LOC100130357 Readthrough | Protein Coding | 9 | GC06M013267 | 0.797032177 | https://www.genecards.org/cgi-bin/carddisp.pl?gene=TBC1D7-LOC100130357 |
| LINC01765 | Long Intergenic Non-Protein Coding RNA 1765 | RNA Gene | 8 | GC01P115101 | 0.797032177 | https://www.genecards.org/cgi-bin/carddisp.pl?gene=LINC01765 |
| ENSG00000272379 |  | RNA Gene | 7 | GC06M013290 | 0.797032177 | https://www.genecards.org/cgi-bin/carddisp.pl?gene=ENSG00000272379 |
| ENSG00000207502 |  | RNA Gene | 7 | GC01M115621 | 0.797032177 | https://www.genecards.org/cgi-bin/carddisp.pl?gene=ENSG00000207502 |
| ENO1P3 | Enolase 1 Pseudogene 3 | Pseudogene | 6 | GC03M124862 | 0.797032177 | https://www.genecards.org/cgi-bin/carddisp.pl?gene=ENO1P3 |
| RNU4-76P | RNA, U4 Small Nuclear 76, Pseudogene | Pseudogene | 6 | GC06M121542 | 0.797032177 | https://www.genecards.org/cgi-bin/carddisp.pl?gene=RNU4-76P |
| SNORD158 | Small Nucleolar RNA, C/D Box 158 | RNA Gene | 6 | GC10M116195 | 0.797032177 | https://www.genecards.org/cgi-bin/carddisp.pl?gene=SNORD158 |
| ENSG00000285698 |  | RNA Gene | 5 | GC01M115270 | 0.797032177 | https://www.genecards.org/cgi-bin/carddisp.pl?gene=ENSG00000285698 |
| ENSG00000285901 |  | Protein Coding | 5 | GC12P012056 | 0.797032177 | https://www.genecards.org/cgi-bin/carddisp.pl?gene=ENSG00000285901 |
| lnc-IQGAP3-2-002 |  | RNA Gene | 5 | GC01M156467 | 0.797032177 | https://www.genecards.org/cgi-bin/carddisp.pl?gene=lnc-IQGAP3-2-002 |
| HSALNG0082653-001 |  | RNA Gene | 4 | GC11P010542 | 0.797032177 | https://www.genecards.org/cgi-bin/carddisp.pl?gene=HSALNG0082653-001 |
| lnc-LYVE1-1 |  | RNA Gene | 4 | GC11M010629 | 0.797032177 | https://www.genecards.org/cgi-bin/carddisp.pl?gene=lnc-LYVE1-1 |
| HSALNG0082654 |  | RNA Gene | 4 | GC11M010717 | 0.797032177 | https://www.genecards.org/cgi-bin/carddisp.pl?gene=HSALNG0082654 |
| hsa-miR-5096-052 |  | RNA Gene | 4 | GC06M012930 | 0.797032177 | https://www.genecards.org/cgi-bin/carddisp.pl?gene=hsa-miR-5096-052 |
| lnc-MUC13-5 |  | RNA Gene | 4 | GC03M124863 | 0.797032177 | https://www.genecards.org/cgi-bin/carddisp.pl?gene=lnc-MUC13-5 |
| HSALNG0006263-001 |  | RNA Gene | 3 | GC01M115132 | 0.797032177 | https://www.genecards.org/cgi-bin/carddisp.pl?gene=HSALNG0006263-001 |
| HSALNG0028586 |  | RNA Gene | 3 | GC03M124818 | 0.797032177 | https://www.genecards.org/cgi-bin/carddisp.pl?gene=HSALNG0028586 |
| HSALNG0007592 |  | RNA Gene | 3 | GC01P156481 | 0.797032177 | https://www.genecards.org/cgi-bin/carddisp.pl?gene=HSALNG0007592 |
| HSALNG0082653-002 |  | RNA Gene | 3 | GC11P010645 | 0.797032177 | https://www.genecards.org/cgi-bin/carddisp.pl?gene=HSALNG0082653-002 |
| ENSG00000219784 |  | Pseudogene | 2 | GC06M121479 | 0.797032177 | https://www.genecards.org/cgi-bin/carddisp.pl?gene=ENSG00000219784 |
| HSALNG0006263-002 |  | RNA Gene | 2 | GC01M115133 | 0.797032177 | https://www.genecards.org/cgi-bin/carddisp.pl?gene=HSALNG0006263-002 |
| HSALNG0006262 |  | RNA Gene | 2 | GC01P115117 | 0.797032177 | https://www.genecards.org/cgi-bin/carddisp.pl?gene=HSALNG0006262 |
| HSALNG0048142 |  | RNA Gene | 2 | GC06M012885 | 0.797032177 | https://www.genecards.org/cgi-bin/carddisp.pl?gene=HSALNG0048142 |
| MN308815 |  | RNA Gene | 2 | GC01P156480 | 0.797032177 | https://www.genecards.org/cgi-bin/carddisp.pl?gene=MN308815 |
| piR-52079-019 |  | RNA Gene | 2 | GC01P156473 | 0.797032177 | https://www.genecards.org/cgi-bin/carddisp.pl?gene=piR-52079-019 |
| CXCR4 | C-X-C Motif Chemokine Receptor 4 | Protein Coding | 52 | GC02M136114 | 0.781373739 | https://www.genecards.org/cgi-bin/carddisp.pl?gene=CXCR4 |
| TLR3 | Toll Like Receptor 3 | Protein Coding | 52 | GC04P186059 | 0.781373739 | https://www.genecards.org/cgi-bin/carddisp.pl?gene=TLR3 |
| SRC | SRC Proto-Oncogene, Non-Receptor Tyrosine Kinase | Protein Coding | 52 | GC20P037344 | 0.781373739 | https://www.genecards.org/cgi-bin/carddisp.pl?gene=SRC |
| NFKBIA | NFKB Inhibitor Alpha | Protein Coding | 51 | GC14M035401 | 0.781373739 | https://www.genecards.org/cgi-bin/carddisp.pl?gene=NFKBIA |
| MYC | MYC Proto-Oncogene, BHLH Transcription Factor | Protein Coding | 51 | GC08P127735 | 0.781373739 | https://www.genecards.org/cgi-bin/carddisp.pl?gene=MYC |
| CREB1 | CAMP Responsive Element Binding Protein 1 | Protein Coding | 50 | GC02P207529 | 0.781373739 | https://www.genecards.org/cgi-bin/carddisp.pl?gene=CREB1 |
| CYP17A1 | Cytochrome P450 Family 17 Subfamily A Member 1 | Protein Coding | 50 | GC10M102830 | 0.781373739 | https://www.genecards.org/cgi-bin/carddisp.pl?gene=CYP17A1 |
| APC | APC Regulator Of WNT Signaling Pathway | Protein Coding | 49 | GC05P112707 | 0.781373739 | https://www.genecards.org/cgi-bin/carddisp.pl?gene=APC |
| P2RY12 | Purinergic Receptor P2Y12 | Protein Coding | 49 | GC03M151336 | 0.781373739 | https://www.genecards.org/cgi-bin/carddisp.pl?gene=P2RY12 |
| MPL | MPL Proto-Oncogene, Thrombopoietin Receptor | Protein Coding | 48 | GC01P043337 | 0.781373739 | https://www.genecards.org/cgi-bin/carddisp.pl?gene=MPL |
| HADHA | Hydroxyacyl-CoA Dehydrogenase Trifunctional Multienzyme Complex Subunit Alpha | Protein Coding | 47 | GC02M026190 | 0.781373739 | https://www.genecards.org/cgi-bin/carddisp.pl?gene=HADHA |
| CD59 | CD59 Molecule (CD59 Blood Group) | Protein Coding | 47 | GC11M033704 | 0.781373739 | https://www.genecards.org/cgi-bin/carddisp.pl?gene=CD59 |
| IGFBP3 | Insulin Like Growth Factor Binding Protein 3 | Protein Coding | 45 | GC07M045912 | 0.781373739 | https://www.genecards.org/cgi-bin/carddisp.pl?gene=IGFBP3 |
| GP6 | Glycoprotein VI Platelet | Protein Coding | 45 | GC19M055013 | 0.781373739 | https://www.genecards.org/cgi-bin/carddisp.pl?gene=GP6 |
| CTSG | Cathepsin G | Protein Coding | 45 | GC14M024573 | 0.781373739 | https://www.genecards.org/cgi-bin/carddisp.pl?gene=CTSG |
| TIMP1 | TIMP Metallopeptidase Inhibitor 1 | Protein Coding | 45 | GC0XP047583 | 0.781373739 | https://www.genecards.org/cgi-bin/carddisp.pl?gene=TIMP1 |
| IGFBP1 | Insulin Like Growth Factor Binding Protein 1 | Protein Coding | 44 | GC07P046838 | 0.781373739 | https://www.genecards.org/cgi-bin/carddisp.pl?gene=IGFBP1 |
| CD9 | CD9 Molecule | Protein Coding | 44 | GC12P011813 | 0.781373739 | https://www.genecards.org/cgi-bin/carddisp.pl?gene=CD9 |
| NAT2 | N-Acetyltransferase 2 | Protein Coding | 44 | GC08P018391 | 0.781373739 | https://www.genecards.org/cgi-bin/carddisp.pl?gene=NAT2 |
| HSPA4 | Heat Shock Protein Family A (Hsp70) Member 4 | Protein Coding | 43 | GC05P133051 | 0.781373739 | https://www.genecards.org/cgi-bin/carddisp.pl?gene=HSPA4 |
| THPO | Thrombopoietin | Protein Coding | 43 | GC03M184371 | 0.781373739 | https://www.genecards.org/cgi-bin/carddisp.pl?gene=THPO |
| RNASE3 | Ribonuclease A Family Member 3 | Protein Coding | 41 | GC14P020891 | 0.781373739 | https://www.genecards.org/cgi-bin/carddisp.pl?gene=RNASE3 |
| PECAM1 | Platelet And Endothelial Cell Adhesion Molecule 1 | Protein Coding | 41 | GC17M064319 | 0.781373739 | https://www.genecards.org/cgi-bin/carddisp.pl?gene=PECAM1 |
| GP5 | Glycoprotein V Platelet | Protein Coding | 40 | GC03M194395 | 0.781373739 | https://www.genecards.org/cgi-bin/carddisp.pl?gene=GP5 |
| CD19 | CD19 Molecule | Protein Coding | 50 | GC16P032270 | 0.771023631 | https://www.genecards.org/cgi-bin/carddisp.pl?gene=CD19 |
| ANXA2 | Annexin A2 | Protein Coding | 49 | GC15M060347 | 0.771023631 | https://www.genecards.org/cgi-bin/carddisp.pl?gene=ANXA2 |
| COL1A2 | Collagen Type I Alpha 2 Chain | Protein Coding | 48 | GC07P094394 | 0.771023631 | https://www.genecards.org/cgi-bin/carddisp.pl?gene=COL1A2 |
| TNFAIP3 | TNF Alpha Induced Protein 3 | Protein Coding | 48 | GC06P137866 | 0.771023631 | https://www.genecards.org/cgi-bin/carddisp.pl?gene=TNFAIP3 |
| PRKACG | Protein Kinase CAMP-Activated Catalytic Subunit Gamma | Protein Coding | 48 | GC09M069013 | 0.771023631 | https://www.genecards.org/cgi-bin/carddisp.pl?gene=PRKACG |
| PTPN22 | Protein Tyrosine Phosphatase Non-Receptor Type 22 | Protein Coding | 48 | GC01M113813 | 0.771023631 | https://www.genecards.org/cgi-bin/carddisp.pl?gene=PTPN22 |
| CD28 | CD28 Molecule | Protein Coding | 47 | GC02P203706 | 0.771023631 | https://www.genecards.org/cgi-bin/carddisp.pl?gene=CD28 |
| CD8A | CD8a Molecule | Protein Coding | 47 | GC02M086784 | 0.771023631 | https://www.genecards.org/cgi-bin/carddisp.pl?gene=CD8A |
| DCN | Decorin | Protein Coding | 47 | GC12M091140 | 0.771023631 | https://www.genecards.org/cgi-bin/carddisp.pl?gene=DCN |
| GZMB | Granzyme B | Protein Coding | 46 | GC14M024630 | 0.771023631 | https://www.genecards.org/cgi-bin/carddisp.pl?gene=GZMB |
| TRPS1 | Transcriptional Repressor GATA Binding 1 | Protein Coding | 46 | GC08M115408 | 0.771023631 | https://www.genecards.org/cgi-bin/carddisp.pl?gene=TRPS1 |
| TBX21 | T-Box Transcription Factor 21 | Protein Coding | 46 | GC17P047733 | 0.771023631 | https://www.genecards.org/cgi-bin/carddisp.pl?gene=TBX21 |
| RASGRP2 | RAS Guanyl Releasing Protein 2 | Protein Coding | 46 | GC11M064726 | 0.771023631 | https://www.genecards.org/cgi-bin/carddisp.pl?gene=RASGRP2 |
| GRID2 | Glutamate Ionotropic Receptor Delta Type Subunit 2 | Protein Coding | 45 | GC04P092304 | 0.771023631 | https://www.genecards.org/cgi-bin/carddisp.pl?gene=GRID2 |
| ELN | Elastin | Protein Coding | 45 | GC07P074027 | 0.771023631 | https://www.genecards.org/cgi-bin/carddisp.pl?gene=ELN |
| PRF1 | Perforin 1 | Protein Coding | 45 | GC10M070597 | 0.771023631 | https://www.genecards.org/cgi-bin/carddisp.pl?gene=PRF1 |
| SLC2A10 | Solute Carrier Family 2 Member 10 | Protein Coding | 45 | GC20P046709 | 0.771023631 | https://www.genecards.org/cgi-bin/carddisp.pl?gene=SLC2A10 |
| TNFRSF10A | TNF Receptor Superfamily Member 10a | Protein Coding | 45 | GC08M023190 | 0.771023631 | https://www.genecards.org/cgi-bin/carddisp.pl?gene=TNFRSF10A |
| DAB1 | DAB Adaptor Protein 1 | Protein Coding | 44 | GC01M056994 | 0.771023631 | https://www.genecards.org/cgi-bin/carddisp.pl?gene=DAB1 |
| GHRL | Ghrelin And Obestatin Prepropeptide | Protein Coding | 44 | GC03M010285 | 0.771023631 | https://www.genecards.org/cgi-bin/carddisp.pl?gene=GHRL |
| CASQ1 | Calsequestrin 1 | Protein Coding | 44 | GC01P160190 | 0.771023631 | https://www.genecards.org/cgi-bin/carddisp.pl?gene=CASQ1 |
| TIA1 | TIA1 Cytotoxic Granule Associated RNA Binding Protein | Protein Coding | 44 | GC02M070209 | 0.771023631 | https://www.genecards.org/cgi-bin/carddisp.pl?gene=TIA1 |
| COL5A2 | Collagen Type V Alpha 2 Chain | Protein Coding | 43 | GC02M189031 | 0.771023631 | https://www.genecards.org/cgi-bin/carddisp.pl?gene=COL5A2 |
| ASPH | Aspartate Beta-Hydroxylase | Protein Coding | 43 | GC08M061500 | 0.771023631 | https://www.genecards.org/cgi-bin/carddisp.pl?gene=ASPH |
| CD80 | CD80 Molecule | Protein Coding | 42 | GC03M119524 | 0.771023631 | https://www.genecards.org/cgi-bin/carddisp.pl?gene=CD80 |
| MICB | MHC Class I Polypeptide-Related Sequence B | Protein Coding | 42 | GC06P055197 | 0.771023631 | https://www.genecards.org/cgi-bin/carddisp.pl?gene=MICB |
| POLRMT | RNA Polymerase Mitochondrial | Protein Coding | 42 | GC19M000617 | 0.771023631 | https://www.genecards.org/cgi-bin/carddisp.pl?gene=POLRMT |
| IL15 | Interleukin 15 | Protein Coding | 41 | GC04P141636 | 0.771023631 | https://www.genecards.org/cgi-bin/carddisp.pl?gene=IL15 |
| ERP44 | Endoplasmic Reticulum Protein 44 | Protein Coding | 41 | GC09M099979 | 0.771023631 | https://www.genecards.org/cgi-bin/carddisp.pl?gene=ERP44 |
| DDX4 | DEAD-Box Helicase 4 | Protein Coding | 41 | GC05P055738 | 0.771023631 | https://www.genecards.org/cgi-bin/carddisp.pl?gene=DDX4 |
| KLRK1 | Killer Cell Lectin Like Receptor K1 | Protein Coding | 41 | GC12M015686 | 0.771023631 | https://www.genecards.org/cgi-bin/carddisp.pl?gene=KLRK1 |
| SIL1 | SIL1 Nucleotide Exchange Factor | Protein Coding | 41 | GC05M138957 | 0.771023631 | https://www.genecards.org/cgi-bin/carddisp.pl?gene=SIL1 |
| STIM2 | Stromal Interaction Molecule 2 | Protein Coding | 40 | GC04P026859 | 0.771023631 | https://www.genecards.org/cgi-bin/carddisp.pl?gene=STIM2 |
| NDUFAF3 | NADH:Ubiquinone Oxidoreductase Complex Assembly Factor 3 | Protein Coding | 40 | GC03P049020 | 0.771023631 | https://www.genecards.org/cgi-bin/carddisp.pl?gene=NDUFAF3 |
| ESYT1 | Extended Synaptotagmin 1 | Protein Coding | 38 | GC12P056379 | 0.771023631 | https://www.genecards.org/cgi-bin/carddisp.pl?gene=ESYT1 |
| ORAI2 | ORAI Calcium Release-Activated Calcium Modulator 2 | Protein Coding | 38 | GC07P102433 | 0.771023631 | https://www.genecards.org/cgi-bin/carddisp.pl?gene=ORAI2 |
| ZNHIT1 | Zinc Finger HIT-Type Containing 1 | Protein Coding | 37 | GC07P101218 | 0.771023631 | https://www.genecards.org/cgi-bin/carddisp.pl?gene=ZNHIT1 |
| NRIP2 | Nuclear Receptor Interacting Protein 2 | Protein Coding | 37 | GC12M002824 | 0.771023631 | https://www.genecards.org/cgi-bin/carddisp.pl?gene=NRIP2 |
| REXO4 | REX4 Homolog, 3'-5' Exonuclease | Protein Coding | 37 | GC09M133406 | 0.771023631 | https://www.genecards.org/cgi-bin/carddisp.pl?gene=REXO4 |
| NDUFB1 | NADH:Ubiquinone Oxidoreductase Subunit B1 | Protein Coding | 37 | GC14M092116 | 0.771023631 | https://www.genecards.org/cgi-bin/carddisp.pl?gene=NDUFB1 |
| NPY4R | Neuropeptide Y Receptor Y4 | Protein Coding | 37 | GC10M046461 | 0.771023631 | https://www.genecards.org/cgi-bin/carddisp.pl?gene=NPY4R |
| MICU2 | Mitochondrial Calcium Uptake 2 | Protein Coding | 36 | GC13M021492 | 0.771023631 | https://www.genecards.org/cgi-bin/carddisp.pl?gene=MICU2 |
| SYPL2 | Synaptophysin Like 2 | Protein Coding | 36 | GC01P109466 | 0.771023631 | https://www.genecards.org/cgi-bin/carddisp.pl?gene=SYPL2 |
| RBM4B | RNA Binding Motif Protein 4B | Protein Coding | 36 | GC11M066664 | 0.771023631 | https://www.genecards.org/cgi-bin/carddisp.pl?gene=RBM4B |
| JSRP1 | Junctional Sarcoplasmic Reticulum Protein 1 | Protein Coding | 35 | GC19M002252 | 0.771023631 | https://www.genecards.org/cgi-bin/carddisp.pl?gene=JSRP1 |
| TMCC1 | Transmembrane And Coiled-Coil Domain Family 1 | Protein Coding | 35 | GC03M129647 | 0.771023631 | https://www.genecards.org/cgi-bin/carddisp.pl?gene=TMCC1 |
| EGR4 | Early Growth Response 4 | Protein Coding | 34 | GC02M073290 | 0.771023631 | https://www.genecards.org/cgi-bin/carddisp.pl?gene=EGR4 |
| ORAI3 | ORAI Calcium Release-Activated Calcium Modulator 3 | Protein Coding | 34 | GC16P030949 | 0.771023631 | https://www.genecards.org/cgi-bin/carddisp.pl?gene=ORAI3 |
| RBM12B | RNA Binding Motif Protein 12B | Protein Coding | 34 | GC08M093729 | 0.771023631 | https://www.genecards.org/cgi-bin/carddisp.pl?gene=RBM12B |
| TMEM178A | Transmembrane Protein 178A | Protein Coding | 32 | GC02P039664 | 0.771023631 | https://www.genecards.org/cgi-bin/carddisp.pl?gene=TMEM178A |
| SARAF | Store-Operated Calcium Entry Associated Regulatory Factor | Protein Coding | 32 | GC08M030063 | 0.771023631 | https://www.genecards.org/cgi-bin/carddisp.pl?gene=SARAF |
| TMEM240 | Transmembrane Protein 240 | Protein Coding | 32 | GC01M002715 | 0.771023631 | https://www.genecards.org/cgi-bin/carddisp.pl?gene=TMEM240 |
| CRACR2A | Calcium Release Activated Channel Regulator 2A | Protein Coding | 31 | GC12M004349 | 0.771023631 | https://www.genecards.org/cgi-bin/carddisp.pl?gene=CRACR2A |
| MICOS13 | Mitochondrial Contact Site And Cristae Organizing System Subunit 13 | Protein Coding | 27 | GC19M005692 | 0.771023631 | https://www.genecards.org/cgi-bin/carddisp.pl?gene=MICOS13 |
| STIMATE | STIM Activating Enhancer | Protein Coding | 24 | GC03M052875 | 0.771023631 | https://www.genecards.org/cgi-bin/carddisp.pl?gene=STIMATE |
| VHL | Von Hippel-Lindau Tumor Suppressor | Protein Coding | 47 | GC03P011015 | 0.734783888 | https://www.genecards.org/cgi-bin/carddisp.pl?gene=VHL |
| CACNB1 | Calcium Voltage-Gated Channel Auxiliary Subunit Beta 1 | Protein Coding | 44 | GC17M039173 | 0.734783888 | https://www.genecards.org/cgi-bin/carddisp.pl?gene=CACNB1 |
| CACNB3 | Calcium Voltage-Gated Channel Auxiliary Subunit Beta 3 | Protein Coding | 43 | GC12P048813 | 0.734783888 | https://www.genecards.org/cgi-bin/carddisp.pl?gene=CACNB3 |
| PSEN1 | Presenilin 1 | Protein Coding | 52 | GC14P073136 | 0.728328526 | https://www.genecards.org/cgi-bin/carddisp.pl?gene=PSEN1 |
| MPO | Myeloperoxidase | Protein Coding | 51 | GC17M058269 | 0.727845728 | https://www.genecards.org/cgi-bin/carddisp.pl?gene=MPO |
| KCNA6 | Potassium Voltage-Gated Channel Subfamily A Member 6 | Protein Coding | 40 | GC12P004806 | 0.727126062 | https://www.genecards.org/cgi-bin/carddisp.pl?gene=KCNA6 |
| FXN | Frataxin | Protein Coding | 47 | GC09P069035 | 0.693591893 | https://www.genecards.org/cgi-bin/carddisp.pl?gene=FXN |
| MME | Membrane Metalloendopeptidase | Protein Coding | 51 | GC03P155024 | 0.690764427 | https://www.genecards.org/cgi-bin/carddisp.pl?gene=MME |
| ECM1 | Extracellular Matrix Protein 1 | Protein Coding | 44 | GC01P150508 | 0.690764427 | https://www.genecards.org/cgi-bin/carddisp.pl?gene=ECM1 |
| ZMIZ1 | Zinc Finger MIZ-Type Containing 1 | Protein Coding | 41 | GC10P079068 | 0.690764427 | https://www.genecards.org/cgi-bin/carddisp.pl?gene=ZMIZ1 |
| TAAR7P | Trace Amine Associated Receptor 7, Pseudogene | Pseudogene | 6 | GC06P132559 | 0.690764427 | https://www.genecards.org/cgi-bin/carddisp.pl?gene=TAAR7P |
| ADRA2A | Adrenoceptor Alpha 2A | Protein Coding | 47 | GC10P111077 | 0.678582728 | https://www.genecards.org/cgi-bin/carddisp.pl?gene=ADRA2A |
| CX3CR1 | C-X3-C Motif Chemokine Receptor 1 | Protein Coding | 45 | GC03M039279 | 0.63947773 | https://www.genecards.org/cgi-bin/carddisp.pl?gene=CX3CR1 |
| ACTG2 | Actin Gamma 2, Smooth Muscle | Protein Coding | 47 | GC02P073892 | 0.61911881 | https://www.genecards.org/cgi-bin/carddisp.pl?gene=ACTG2 |
| CHRNA7 | Cholinergic Receptor Nicotinic Alpha 7 Subunit | Protein Coding | 46 | GC15P031923 | 0.61911881 | https://www.genecards.org/cgi-bin/carddisp.pl?gene=CHRNA7 |
| GABRA1 | Gamma-Aminobutyric Acid Type A Receptor Subunit Alpha1 | Protein Coding | 47 | GC05P161847 | 0.617621064 | https://www.genecards.org/cgi-bin/carddisp.pl?gene=GABRA1 |
| MT-ND2 | Mitochondrially Encoded NADH:Ubiquinone Oxidoreductase Core Subunit 2 | Protein Coding | 34 | GCMTP004472 | 0.617621064 | https://www.genecards.org/cgi-bin/carddisp.pl?gene=MT-ND2 |
| MT-ND4L | Mitochondrially Encoded NADH:Ubiquinone Oxidoreductase Core Subunit 4L | Protein Coding | 29 | GCMTP010472 | 0.617621064 | https://www.genecards.org/cgi-bin/carddisp.pl?gene=MT-ND4L |
| MT-RNR2 | Mitochondrially Encoded 16S RRNA | RNA Gene | 18 | GCMTP001674 | 0.617621064 | https://www.genecards.org/cgi-bin/carddisp.pl?gene=MT-RNR2 |
| AKR1B1 | Aldo-Keto Reductase Family 1 Member B | Protein Coding | 48 | GC07M134442 | 0.614585757 | https://www.genecards.org/cgi-bin/carddisp.pl?gene=AKR1B1 |
| COG2 | Component Of Oligomeric Golgi Complex 2 | Protein Coding | 41 | GC01P230642 | 0.614585757 | https://www.genecards.org/cgi-bin/carddisp.pl?gene=COG2 |
| GABBR2 | Gamma-Aminobutyric Acid Type B Receptor Subunit 2 | Protein Coding | 49 | GC09M098288 | 0.608802915 | https://www.genecards.org/cgi-bin/carddisp.pl?gene=GABBR2 |
| CYCS | Cytochrome C, Somatic | Protein Coding | 49 | GC07M025118 | 0.600668192 | https://www.genecards.org/cgi-bin/carddisp.pl?gene=CYCS |
| MBL2 | Mannose Binding Lectin 2 | Protein Coding | 48 | GC10M052760 | 0.600668192 | https://www.genecards.org/cgi-bin/carddisp.pl?gene=MBL2 |
| HSPD1 | Heat Shock Protein Family D (Hsp60) Member 1 | Protein Coding | 48 | GC02M197486 | 0.600668192 | https://www.genecards.org/cgi-bin/carddisp.pl?gene=HSPD1 |
| GFAP | Glial Fibrillary Acidic Protein | Protein Coding | 48 | GC17M044905 | 0.600668192 | https://www.genecards.org/cgi-bin/carddisp.pl?gene=GFAP |
| COX4I1 | Cytochrome C Oxidase Subunit 4I1 | Protein Coding | 47 | GC16P085798 | 0.600668192 | https://www.genecards.org/cgi-bin/carddisp.pl?gene=COX4I1 |
| FCGR2A | Fc Fragment Of IgG Receptor IIa | Protein Coding | 46 | GC01P161505 | 0.600668192 | https://www.genecards.org/cgi-bin/carddisp.pl?gene=FCGR2A |
| CXCL10 | C-X-C Motif Chemokine Ligand 10 | Protein Coding | 45 | GC04M076021 | 0.600668192 | https://www.genecards.org/cgi-bin/carddisp.pl?gene=CXCL10 |
| ATXN10 | Ataxin 10 | Protein Coding | 43 | GC22P045673 | 0.600668192 | https://www.genecards.org/cgi-bin/carddisp.pl?gene=ATXN10 |
| NDUFAF2 | NADH:Ubiquinone Oxidoreductase Complex Assembly Factor 2 | Protein Coding | 41 | GC05P060945 | 0.600668192 | https://www.genecards.org/cgi-bin/carddisp.pl?gene=NDUFAF2 |
| EPRS1 | Glutamyl-Prolyl-TRNA Synthetase 1 | Protein Coding | 37 | GC01M219969 | 0.600668192 | https://www.genecards.org/cgi-bin/carddisp.pl?gene=EPRS1 |
| TSC2 | TSC Complex Subunit 2 | Protein Coding | 50 | GC16P005579 | 0.599948525 | https://www.genecards.org/cgi-bin/carddisp.pl?gene=TSC2 |
| SERPINA1 | Serpin Family A Member 1 | Protein Coding | 50 | GC14M094376 | 0.599948525 | https://www.genecards.org/cgi-bin/carddisp.pl?gene=SERPINA1 |
| PDHA1 | Pyruvate Dehydrogenase E1 Subunit Alpha 1 | Protein Coding | 50 | GC0XP019343 | 0.599948525 | https://www.genecards.org/cgi-bin/carddisp.pl?gene=PDHA1 |
| CYP1B1 | Cytochrome P450 Family 1 Subfamily B Member 1 | Protein Coding | 49 | GC02M038066 | 0.599948525 | https://www.genecards.org/cgi-bin/carddisp.pl?gene=CYP1B1 |
| GNA11 | G Protein Subunit Alpha 11 | Protein Coding | 48 | GC19P003094 | 0.599948525 | https://www.genecards.org/cgi-bin/carddisp.pl?gene=GNA11 |
| OTX2 | Orthodenticle Homeobox 2 | Protein Coding | 47 | GC14M056799 | 0.599948525 | https://www.genecards.org/cgi-bin/carddisp.pl?gene=OTX2 |
| KCND3 | Potassium Voltage-Gated Channel Subfamily D Member 3 | Protein Coding | 47 | GC01M111770 | 0.599948525 | https://www.genecards.org/cgi-bin/carddisp.pl?gene=KCND3 |
| KCNA2 | Potassium Voltage-Gated Channel Subfamily A Member 2 | Protein Coding | 46 | GC01M110519 | 0.599948525 | https://www.genecards.org/cgi-bin/carddisp.pl?gene=KCNA2 |
| FGF14 | Fibroblast Growth Factor 14 | Protein Coding | 45 | GC13M101710 | 0.599948525 | https://www.genecards.org/cgi-bin/carddisp.pl?gene=FGF14 |
| GNA14 | G Protein Subunit Alpha 14 | Protein Coding | 45 | GC09M077423 | 0.599948525 | https://www.genecards.org/cgi-bin/carddisp.pl?gene=GNA14 |
| SYNGAP1 | Synaptic Ras GTPase Activating Protein 1 | Protein Coding | 45 | GC06P033419 | 0.599948525 | https://www.genecards.org/cgi-bin/carddisp.pl?gene=SYNGAP1 |
| PNPLA6 | Patatin Like Phospholipase Domain Containing 6 | Protein Coding | 45 | GC19P007534 | 0.599948525 | https://www.genecards.org/cgi-bin/carddisp.pl?gene=PNPLA6 |
| TOR1A | Torsin Family 1 Member A | Protein Coding | 45 | GC09M129812 | 0.599948525 | https://www.genecards.org/cgi-bin/carddisp.pl?gene=TOR1A |
| NEUROD1 | Neuronal Differentiation 1 | Protein Coding | 44 | GC02M181673 | 0.599948525 | https://www.genecards.org/cgi-bin/carddisp.pl?gene=NEUROD1 |
| KCNA4 | Potassium Voltage-Gated Channel Subfamily A Member 4 | Protein Coding | 44 | GC11M030009 | 0.599948525 | https://www.genecards.org/cgi-bin/carddisp.pl?gene=KCNA4 |
| KCNC1 | Potassium Voltage-Gated Channel Subfamily C Member 1 | Protein Coding | 44 | GC11P017756 | 0.599948525 | https://www.genecards.org/cgi-bin/carddisp.pl?gene=KCNC1 |
| TNFRSF25 | TNF Receptor Superfamily Member 25 | Protein Coding | 44 | GC01M006460 | 0.599948525 | https://www.genecards.org/cgi-bin/carddisp.pl?gene=TNFRSF25 |
| NLGN3 | Neuroligin 3 | Protein Coding | 43 | GC0XP071144 | 0.599948525 | https://www.genecards.org/cgi-bin/carddisp.pl?gene=NLGN3 |
| GNA15 | G Protein Subunit Alpha 15 | Protein Coding | 43 | GC19P003173 | 0.599948525 | https://www.genecards.org/cgi-bin/carddisp.pl?gene=GNA15 |
| PREP | Prolyl Endopeptidase | Protein Coding | 43 | GC06M105277 | 0.599948525 | https://www.genecards.org/cgi-bin/carddisp.pl?gene=PREP |
| KCNAB2 | Potassium Voltage-Gated Channel Subfamily A Regulatory Beta Subunit 2 | Protein Coding | 43 | GC01P006020 | 0.599948525 | https://www.genecards.org/cgi-bin/carddisp.pl?gene=KCNAB2 |
| ENAH | ENAH Actin Regulator | Protein Coding | 42 | GC01M225486 | 0.599948525 | https://www.genecards.org/cgi-bin/carddisp.pl?gene=ENAH |
| EIF4H | Eukaryotic Translation Initiation Factor 4H | Protein Coding | 42 | GC07P074174 | 0.599948525 | https://www.genecards.org/cgi-bin/carddisp.pl?gene=EIF4H |
| KCNAB1 | Potassium Voltage-Gated Channel Subfamily A Member Regulatory Beta Subunit 1 | Protein Coding | 42 | GC03P156037 | 0.599948525 | https://www.genecards.org/cgi-bin/carddisp.pl?gene=KCNAB1 |
| BSND | Barttin CLCNK Type Accessory Subunit Beta | Protein Coding | 41 | GC01P054998 | 0.599948525 | https://www.genecards.org/cgi-bin/carddisp.pl?gene=BSND |
| MVP | Major Vault Protein | Protein Coding | 41 | GC16P032377 | 0.599948525 | https://www.genecards.org/cgi-bin/carddisp.pl?gene=MVP |
| TSHZ1 | Teashirt Zinc Finger Homeobox 1 | Protein Coding | 40 | GC18P075210 | 0.599948525 | https://www.genecards.org/cgi-bin/carddisp.pl?gene=TSHZ1 |
| NALCN | Sodium Leak Channel, Non-Selective | Protein Coding | 40 | GC13M101053 | 0.599948525 | https://www.genecards.org/cgi-bin/carddisp.pl?gene=NALCN |
| UBR4 | Ubiquitin Protein Ligase E3 Component N-Recognin 4 | Protein Coding | 39 | GC01M019074 | 0.599948525 | https://www.genecards.org/cgi-bin/carddisp.pl?gene=UBR4 |
| VAX2 | Ventral Anterior Homeobox 2 | Protein Coding | 38 | GC02P070900 | 0.599948525 | https://www.genecards.org/cgi-bin/carddisp.pl?gene=VAX2 |
| CHRAC1 | Chromatin Accessibility Complex Subunit 1 | Protein Coding | 37 | GC08P140511 | 0.599948525 | https://www.genecards.org/cgi-bin/carddisp.pl?gene=CHRAC1 |
| ZC4H2 | Zinc Finger C4H2-Type Containing | Protein Coding | 37 | GC0XM064915 | 0.599948525 | https://www.genecards.org/cgi-bin/carddisp.pl?gene=ZC4H2 |
| EFHD1 | EF-Hand Domain Family Member D1 | Protein Coding | 36 | GC02P232606 | 0.599948525 | https://www.genecards.org/cgi-bin/carddisp.pl?gene=EFHD1 |
| OR3A3 | Olfactory Receptor Family 3 Subfamily A Member 3 | Protein Coding | 34 | GC17P003422 | 0.599948525 | https://www.genecards.org/cgi-bin/carddisp.pl?gene=OR3A3 |
| ACSM4 | Acyl-CoA Synthetase Medium Chain Family Member 4 | Protein Coding | 33 | GC12P007304 | 0.599948525 | https://www.genecards.org/cgi-bin/carddisp.pl?gene=ACSM4 |
| ACSM6 | Acyl-CoA Synthetase Medium Chain Family Member 6 | Protein Coding | 31 | GC10P095194 | 0.599948525 | https://www.genecards.org/cgi-bin/carddisp.pl?gene=ACSM6 |
| LINC01622 | Long Intergenic Non-Protein Coding RNA 1622 | RNA Gene | 12 | GC06M000960 | 0.599948525 | https://www.genecards.org/cgi-bin/carddisp.pl?gene=LINC01622 |
| GH-LCR | Growth Hormone Locus Control Region | Biological Region | 2 | GC17P063917 | 0.599948525 | https://www.genecards.org/cgi-bin/carddisp.pl?gene=GH-LCR |
| TRIM5 | Tripartite Motif Containing 5 | Protein Coding | 42 | GC11M005753 | 0.570168376 | https://www.genecards.org/cgi-bin/carddisp.pl?gene=TRIM5 |
| MTOR | Mechanistic Target Of Rapamycin Kinase | Protein Coding | 54 | GC01M011106 | 0.563586891 | https://www.genecards.org/cgi-bin/carddisp.pl?gene=MTOR |
| MAPK10 | Mitogen-Activated Protein Kinase 10 | Protein Coding | 52 | GC04M085990 | 0.563586891 | https://www.genecards.org/cgi-bin/carddisp.pl?gene=MAPK10 |
| AXL | AXL Receptor Tyrosine Kinase | Protein Coding | 52 | GC19P041219 | 0.563586891 | https://www.genecards.org/cgi-bin/carddisp.pl?gene=AXL |
| ITGB1 | Integrin Subunit Beta 1 | Protein Coding | 51 | GC10M032890 | 0.563586891 | https://www.genecards.org/cgi-bin/carddisp.pl?gene=ITGB1 |
| GJA1 | Gap Junction Protein Alpha 1 | Protein Coding | 51 | GC06P121436 | 0.563586891 | https://www.genecards.org/cgi-bin/carddisp.pl?gene=GJA1 |
| HCK | HCK Proto-Oncogene, Src Family Tyrosine Kinase | Protein Coding | 50 | GC20P032052 | 0.563586891 | https://www.genecards.org/cgi-bin/carddisp.pl?gene=HCK |
| CSNK2B | Casein Kinase 2 Beta | Protein Coding | 50 | GC06P055205 | 0.563586891 | https://www.genecards.org/cgi-bin/carddisp.pl?gene=CSNK2B |
| TUBB | Tubulin Beta Class I | Protein Coding | 50 | GC06P055181 | 0.563586891 | https://www.genecards.org/cgi-bin/carddisp.pl?gene=TUBB |
| TNC | Tenascin C | Protein Coding | 50 | GC09M115019 | 0.563586891 | https://www.genecards.org/cgi-bin/carddisp.pl?gene=TNC |
| PAK4 | P21 (RAC1) Activated Kinase 4 | Protein Coding | 49 | GC19P039125 | 0.563586891 | https://www.genecards.org/cgi-bin/carddisp.pl?gene=PAK4 |
| RPS19 | Ribosomal Protein S19 | Protein Coding | 49 | GC19P041859 | 0.563586891 | https://www.genecards.org/cgi-bin/carddisp.pl?gene=RPS19 |
| MYLK2 | Myosin Light Chain Kinase 2 | Protein Coding | 49 | GC20P031819 | 0.563586891 | https://www.genecards.org/cgi-bin/carddisp.pl?gene=MYLK2 |
| LMNA | Lamin A/C | Protein Coding | 48 | GC01P156082 | 0.563586891 | https://www.genecards.org/cgi-bin/carddisp.pl?gene=LMNA |
| BUB3 | BUB3 Mitotic Checkpoint Protein | Protein Coding | 48 | GC10P123154 | 0.563586891 | https://www.genecards.org/cgi-bin/carddisp.pl?gene=BUB3 |
| USP9X | Ubiquitin Specific Peptidase 9 X-Linked | Protein Coding | 48 | GC0XP041085 | 0.563586891 | https://www.genecards.org/cgi-bin/carddisp.pl?gene=USP9X |
| YAP1 | Yes1 Associated Transcriptional Regulator | Protein Coding | 48 | GC11P102110 | 0.563586891 | https://www.genecards.org/cgi-bin/carddisp.pl?gene=YAP1 |
| ARHGEF1 | Rho Guanine Nucleotide Exchange Factor 1 | Protein Coding | 47 | GC19P041883 | 0.563586891 | https://www.genecards.org/cgi-bin/carddisp.pl?gene=ARHGEF1 |
| MAP2K5 | Mitogen-Activated Protein Kinase Kinase 5 | Protein Coding | 47 | GC15P077101 | 0.563586891 | https://www.genecards.org/cgi-bin/carddisp.pl?gene=MAP2K5 |
| GLO1 | Glyoxalase I | Protein Coding | 47 | GC06M047047 | 0.563586891 | https://www.genecards.org/cgi-bin/carddisp.pl?gene=GLO1 |
| FECH | Ferrochelatase | Protein Coding | 47 | GC18M057544 | 0.563586891 | https://www.genecards.org/cgi-bin/carddisp.pl?gene=FECH |
| DDR1 | Discoidin Domain Receptor Tyrosine Kinase 1 | Protein Coding | 47 | GC06P055184 | 0.563586891 | https://www.genecards.org/cgi-bin/carddisp.pl?gene=DDR1 |
| ETV1 | ETS Variant Transcription Factor 1 | Protein Coding | 47 | GC07M013891 | 0.563586891 | https://www.genecards.org/cgi-bin/carddisp.pl?gene=ETV1 |
| ETV6 | ETS Variant Transcription Factor 6 | Protein Coding | 47 | GC12P011649 | 0.563586891 | https://www.genecards.org/cgi-bin/carddisp.pl?gene=ETV6 |
| TARDBP | TAR DNA Binding Protein | Protein Coding | 47 | GC01P011013 | 0.563586891 | https://www.genecards.org/cgi-bin/carddisp.pl?gene=TARDBP |
| PGD | Phosphogluconate Dehydrogenase | Protein Coding | 47 | GC01P010398 | 0.563586891 | https://www.genecards.org/cgi-bin/carddisp.pl?gene=PGD |
| NT5C2 | 5'-Nucleotidase, Cytosolic II | Protein Coding | 47 | GC10M103088 | 0.563586891 | https://www.genecards.org/cgi-bin/carddisp.pl?gene=NT5C2 |
| RPA1 | Replication Protein A1 | Protein Coding | 47 | GC17P001829 | 0.563586891 | https://www.genecards.org/cgi-bin/carddisp.pl?gene=RPA1 |
| FGF23 | Fibroblast Growth Factor 23 | Protein Coding | 46 | GC12M004368 | 0.563586891 | https://www.genecards.org/cgi-bin/carddisp.pl?gene=FGF23 |
| CTSS | Cathepsin S | Protein Coding | 46 | GC01M150730 | 0.563586891 | https://www.genecards.org/cgi-bin/carddisp.pl?gene=CTSS |
| BIRC2 | Baculoviral IAP Repeat Containing 2 | Protein Coding | 46 | GC11P102347 | 0.563586891 | https://www.genecards.org/cgi-bin/carddisp.pl?gene=BIRC2 |
| TCF7L2 | Transcription Factor 7 Like 2 | Protein Coding | 46 | GC10P112950 | 0.563586891 | https://www.genecards.org/cgi-bin/carddisp.pl?gene=TCF7L2 |
| MMP11 | Matrix Metallopeptidase 11 | Protein Coding | 46 | GC22P023768 | 0.563586891 | https://www.genecards.org/cgi-bin/carddisp.pl?gene=MMP11 |
| MOG | Myelin Oligodendrocyte Glycoprotein | Protein Coding | 46 | GC06P055140 | 0.563586891 | https://www.genecards.org/cgi-bin/carddisp.pl?gene=MOG |
| NFS1 | NFS1 Cysteine Desulfurase | Protein Coding | 46 | GC20M035668 | 0.563586891 | https://www.genecards.org/cgi-bin/carddisp.pl?gene=NFS1 |
| FZD9 | Frizzled Class Receptor 9 | Protein Coding | 45 | GC07P073433 | 0.563586891 | https://www.genecards.org/cgi-bin/carddisp.pl?gene=FZD9 |
| HABP2 | Hyaluronan Binding Protein 2 | Protein Coding | 45 | GC10P113550 | 0.563586891 | https://www.genecards.org/cgi-bin/carddisp.pl?gene=HABP2 |
| FBXW11 | F-Box And WD Repeat Domain Containing 11 | Protein Coding | 45 | GC05M171861 | 0.563586891 | https://www.genecards.org/cgi-bin/carddisp.pl?gene=FBXW11 |
| DCLRE1C | DNA Cross-Link Repair 1C | Protein Coding | 45 | GC10M014897 | 0.563586891 | https://www.genecards.org/cgi-bin/carddisp.pl?gene=DCLRE1C |
| DFFA | DNA Fragmentation Factor Subunit Alpha | Protein Coding | 45 | GC01M010456 | 0.563586891 | https://www.genecards.org/cgi-bin/carddisp.pl?gene=DFFA |
| ARL3 | ADP Ribosylation Factor Like GTPase 3 | Protein Coding | 45 | GC10M102673 | 0.563586891 | https://www.genecards.org/cgi-bin/carddisp.pl?gene=ARL3 |
| BCAR1 | BCAR1 Scaffold Protein, Cas Family Member | Protein Coding | 45 | GC16M075228 | 0.563586891 | https://www.genecards.org/cgi-bin/carddisp.pl?gene=BCAR1 |
| XRCC5 | X-Ray Repair Cross Complementing 5 | Protein Coding | 45 | GC02P216107 | 0.563586891 | https://www.genecards.org/cgi-bin/carddisp.pl?gene=XRCC5 |
| POLR2A | RNA Polymerase II Subunit A | Protein Coding | 45 | GC17P009102 | 0.563586891 | https://www.genecards.org/cgi-bin/carddisp.pl?gene=POLR2A |
| REST | RE1 Silencing Transcription Factor | Protein Coding | 45 | GC04P056907 | 0.563586891 | https://www.genecards.org/cgi-bin/carddisp.pl?gene=REST |
| NCOR2 | Nuclear Receptor Corepressor 2 | Protein Coding | 45 | GC12M124324 | 0.563586891 | https://www.genecards.org/cgi-bin/carddisp.pl?gene=NCOR2 |
| RBBP8 | RB Binding Protein 8, Endonuclease | Protein Coding | 45 | GC18P022798 | 0.563586891 | https://www.genecards.org/cgi-bin/carddisp.pl?gene=RBBP8 |
| FSHB | Follicle Stimulating Hormone Subunit Beta | Protein Coding | 44 | GC11P030210 | 0.563586891 | https://www.genecards.org/cgi-bin/carddisp.pl?gene=FSHB |
| DPP6 | Dipeptidyl Peptidase Like 6 | Protein Coding | 44 | GC07P153748 | 0.563586891 | https://www.genecards.org/cgi-bin/carddisp.pl?gene=DPP6 |
| MAP4K1 | Mitogen-Activated Protein Kinase Kinase Kinase Kinase 1 | Protein Coding | 44 | GC19M038587 | 0.563586891 | https://www.genecards.org/cgi-bin/carddisp.pl?gene=MAP4K1 |
| GPD1L | Glycerol-3-Phosphate Dehydrogenase 1 Like | Protein Coding | 44 | GC03P032123 | 0.563586891 | https://www.genecards.org/cgi-bin/carddisp.pl?gene=GPD1L |
| GSTO1 | Glutathione S-Transferase Omega 1 | Protein Coding | 44 | GC10P104235 | 0.563586891 | https://www.genecards.org/cgi-bin/carddisp.pl?gene=GSTO1 |
| EIF4A3 | Eukaryotic Translation Initiation Factor 4A3 | Protein Coding | 44 | GC17M080135 | 0.563586891 | https://www.genecards.org/cgi-bin/carddisp.pl?gene=EIF4A3 |
| TRIM32 | Tripartite Motif Containing 32 | Protein Coding | 44 | GC09P116687 | 0.563586891 | https://www.genecards.org/cgi-bin/carddisp.pl?gene=TRIM32 |
| TLE3 | TLE Family Member 3, Transcriptional Corepressor | Protein Coding | 44 | GC15M070047 | 0.563586891 | https://www.genecards.org/cgi-bin/carddisp.pl?gene=TLE3 |
| RPS24 | Ribosomal Protein S24 | Protein Coding | 44 | GC10P078033 | 0.563586891 | https://www.genecards.org/cgi-bin/carddisp.pl?gene=RPS24 |
| MYO1C | Myosin IC | Protein Coding | 44 | GC17M001464 | 0.563586891 | https://www.genecards.org/cgi-bin/carddisp.pl?gene=MYO1C |
| DLX3 | Distal-Less Homeobox 3 | Protein Coding | 43 | GC17M049990 | 0.563586891 | https://www.genecards.org/cgi-bin/carddisp.pl?gene=DLX3 |
| DOCK8 | Dedicator Of Cytokinesis 8 | Protein Coding | 43 | GC09P000214 | 0.563586891 | https://www.genecards.org/cgi-bin/carddisp.pl?gene=DOCK8 |
| IQGAP2 | IQ Motif Containing GTPase Activating Protein 2 | Protein Coding | 43 | GC05P076403 | 0.563586891 | https://www.genecards.org/cgi-bin/carddisp.pl?gene=IQGAP2 |
| INPP5A | Inositol Polyphosphate-5-Phosphatase A | Protein Coding | 43 | GC10P132537 | 0.563586891 | https://www.genecards.org/cgi-bin/carddisp.pl?gene=INPP5A |
| KLF10 | Kruppel Like Factor 10 | Protein Coding | 43 | GC08M102648 | 0.563586891 | https://www.genecards.org/cgi-bin/carddisp.pl?gene=KLF10 |
| HPS1 | HPS1 Biogenesis Of Lysosomal Organelles Complex 3 Subunit 1 | Protein Coding | 43 | GC10M098416 | 0.563586891 | https://www.genecards.org/cgi-bin/carddisp.pl?gene=HPS1 |
| CENPF | Centromere Protein F | Protein Coding | 43 | GC01P214603 | 0.563586891 | https://www.genecards.org/cgi-bin/carddisp.pl?gene=CENPF |
| CIC | Capicua Transcriptional Repressor | Protein Coding | 43 | GC19P042268 | 0.563586891 | https://www.genecards.org/cgi-bin/carddisp.pl?gene=CIC |
| VARS2 | Valyl-TRNA Synthetase 2, Mitochondrial | Protein Coding | 43 | GC06P055186 | 0.563586891 | https://www.genecards.org/cgi-bin/carddisp.pl?gene=VARS2 |
| TRPM3 | Transient Receptor Potential Cation Channel Subfamily M Member 3 | Protein Coding | 43 | GC09M070529 | 0.563586891 | https://www.genecards.org/cgi-bin/carddisp.pl?gene=TRPM3 |
| TARS2 | Threonyl-TRNA Synthetase 2, Mitochondrial | Protein Coding | 43 | GC01P150502 | 0.563586891 | https://www.genecards.org/cgi-bin/carddisp.pl?gene=TARS2 |
| MLXIPL | MLX Interacting Protein Like | Protein Coding | 43 | GC07M073593 | 0.563586891 | https://www.genecards.org/cgi-bin/carddisp.pl?gene=MLXIPL |
| ROBO2 | Roundabout Guidance Receptor 2 | Protein Coding | 43 | GC03P075955 | 0.563586891 | https://www.genecards.org/cgi-bin/carddisp.pl?gene=ROBO2 |
| FLOT1 | Flotillin 1 | Protein Coding | 42 | GC06M046803 | 0.563586891 | https://www.genecards.org/cgi-bin/carddisp.pl?gene=FLOT1 |
| JPH3 | Junctophilin 3 | Protein Coding | 42 | GC16P087601 | 0.563586891 | https://www.genecards.org/cgi-bin/carddisp.pl?gene=JPH3 |
| ITPK1 | Inositol-Tetrakisphosphate 1-Kinase | Protein Coding | 42 | GC14M092936 | 0.563586891 | https://www.genecards.org/cgi-bin/carddisp.pl?gene=ITPK1 |
| ARHGEF11 | Rho Guanine Nucleotide Exchange Factor 11 | Protein Coding | 42 | GC01M156904 | 0.563586891 | https://www.genecards.org/cgi-bin/carddisp.pl?gene=ARHGEF11 |
| CAMK1D | Calcium/Calmodulin Dependent Protein Kinase ID | Protein Coding | 42 | GC10P012349 | 0.563586891 | https://www.genecards.org/cgi-bin/carddisp.pl?gene=CAMK1D |
| ASCC1 | Activating Signal Cointegrator 1 Complex Subunit 1 | Protein Coding | 42 | GC10M072096 | 0.563586891 | https://www.genecards.org/cgi-bin/carddisp.pl?gene=ASCC1 |
| SVIL | Supervillin | Protein Coding | 42 | GC10M031105 | 0.563586891 | https://www.genecards.org/cgi-bin/carddisp.pl?gene=SVIL |
| TPD52 | Tumor Protein D52 | Protein Coding | 42 | GC08M079920 | 0.563586891 | https://www.genecards.org/cgi-bin/carddisp.pl?gene=TPD52 |
| PRPF8 | Pre-MRNA Processing Factor 8 | Protein Coding | 42 | GC17M001650 | 0.563586891 | https://www.genecards.org/cgi-bin/carddisp.pl?gene=PRPF8 |
| PLEKHA1 | Pleckstrin Homology Domain Containing A1 | Protein Coding | 42 | GC10P122374 | 0.563586891 | https://www.genecards.org/cgi-bin/carddisp.pl?gene=PLEKHA1 |
| MLLT10 | MLLT10 Histone Lysine Methyltransferase DOT1L Cofactor | Protein Coding | 42 | GC10P021524 | 0.563586891 | https://www.genecards.org/cgi-bin/carddisp.pl?gene=MLLT10 |
| PABPC4 | Poly(A) Binding Protein Cytoplasmic 4 | Protein Coding | 42 | GC01M039560 | 0.563586891 | https://www.genecards.org/cgi-bin/carddisp.pl?gene=PABPC4 |
| COL13A1 | Collagen Type XIII Alpha 1 Chain | Protein Coding | 41 | GC10P069801 | 0.563586891 | https://www.genecards.org/cgi-bin/carddisp.pl?gene=COL13A1 |
| CSTF2T | Cleavage Stimulation Factor Subunit 2 Tau Variant | Protein Coding | 41 | GC10M051695 | 0.563586891 | https://www.genecards.org/cgi-bin/carddisp.pl?gene=CSTF2T |
| DPH1 | Diphthamide Biosynthesis 1 | Protein Coding | 41 | GC17P002030 | 0.563586891 | https://www.genecards.org/cgi-bin/carddisp.pl?gene=DPH1 |
| DNAJA3 | DnaJ Heat Shock Protein Family (Hsp40) Member A3 | Protein Coding | 41 | GC16P004425 | 0.563586891 | https://www.genecards.org/cgi-bin/carddisp.pl?gene=DNAJA3 |
| AS3MT | Arsenite Methyltransferase | Protein Coding | 41 | GC10P102869 | 0.563586891 | https://www.genecards.org/cgi-bin/carddisp.pl?gene=AS3MT |
| ASAP1 | ArfGAP With SH3 Domain, Ankyrin Repeat And PH Domain 1 | Protein Coding | 41 | GC08M130052 | 0.563586891 | https://www.genecards.org/cgi-bin/carddisp.pl?gene=ASAP1 |
| BAZ1B | Bromodomain Adjacent To Zinc Finger Domain 1B | Protein Coding | 41 | GC07M073440 | 0.563586891 | https://www.genecards.org/cgi-bin/carddisp.pl?gene=BAZ1B |
| HIC1 | HIC ZBTB Transcriptional Repressor 1 | Protein Coding | 41 | GC17P002054 | 0.563586891 | https://www.genecards.org/cgi-bin/carddisp.pl?gene=HIC1 |
| FUT9 | Fucosyltransferase 9 | Protein Coding | 41 | GC06P096015 | 0.563586891 | https://www.genecards.org/cgi-bin/carddisp.pl?gene=FUT9 |
| GTF2H4 | General Transcription Factor IIH Subunit 4 | Protein Coding | 41 | GC06P055185 | 0.563586891 | https://www.genecards.org/cgi-bin/carddisp.pl?gene=GTF2H4 |
| MDC1 | Mediator Of DNA Damage Checkpoint 1 | Protein Coding | 41 | GC06M046797 | 0.563586891 | https://www.genecards.org/cgi-bin/carddisp.pl?gene=MDC1 |
| DHX16 | DEAH-Box Helicase 16 | Protein Coding | 41 | GC06M030653 | 0.563586891 | https://www.genecards.org/cgi-bin/carddisp.pl?gene=DHX16 |
| BCAN | Brevican | Protein Coding | 41 | GC01P156641 | 0.563586891 | https://www.genecards.org/cgi-bin/carddisp.pl?gene=BCAN |
| MSH5 | MutS Homolog 5 | Protein Coding | 41 | GC06P055215 | 0.563586891 | https://www.genecards.org/cgi-bin/carddisp.pl?gene=MSH5 |
| PHLPP1 | PH Domain And Leucine Rich Repeat Protein Phosphatase 1 | Protein Coding | 41 | GC18P062715 | 0.563586891 | https://www.genecards.org/cgi-bin/carddisp.pl?gene=PHLPP1 |
| PAPPA | Pappalysin 1 | Protein Coding | 41 | GC09P116231 | 0.563586891 | https://www.genecards.org/cgi-bin/carddisp.pl?gene=PAPPA |
| RPS16 | Ribosomal Protein S16 | Protein Coding | 41 | GC19M039433 | 0.563586891 | https://www.genecards.org/cgi-bin/carddisp.pl?gene=RPS16 |
| SNRPA | Small Nuclear Ribonucleoprotein Polypeptide A | Protein Coding | 41 | GC19P040750 | 0.563586891 | https://www.genecards.org/cgi-bin/carddisp.pl?gene=SNRPA |
| CPNE1 | Copine 1 | Protein Coding | 40 | GC20M035626 | 0.563586891 | https://www.genecards.org/cgi-bin/carddisp.pl?gene=CPNE1 |
| DOCK4 | Dedicator Of Cytokinesis 4 | Protein Coding | 40 | GC07M111726 | 0.563586891 | https://www.genecards.org/cgi-bin/carddisp.pl?gene=DOCK4 |
| IMMP2L | Inner Mitochondrial Membrane Peptidase Subunit 2 | Protein Coding | 40 | GC07M110663 | 0.563586891 | https://www.genecards.org/cgi-bin/carddisp.pl?gene=IMMP2L |
| MED14 | Mediator Complex Subunit 14 | Protein Coding | 40 | GC0XM040648 | 0.563586891 | https://www.genecards.org/cgi-bin/carddisp.pl?gene=MED14 |
| LRP1B | LDL Receptor Related Protein 1B | Protein Coding | 40 | GC02M140231 | 0.563586891 | https://www.genecards.org/cgi-bin/carddisp.pl?gene=LRP1B |
| LTBP4 | Latent Transforming Growth Factor Beta Binding Protein 4 | Protein Coding | 40 | GC19P040592 | 0.563586891 | https://www.genecards.org/cgi-bin/carddisp.pl?gene=LTBP4 |
| HNRNPL | Heterogeneous Nuclear Ribonucleoprotein L | Protein Coding | 40 | GC19M038836 | 0.563586891 | https://www.genecards.org/cgi-bin/carddisp.pl?gene=HNRNPL |
| EXOC2 | Exocyst Complex Component 2 | Protein Coding | 40 | GC06M000485 | 0.563586891 | https://www.genecards.org/cgi-bin/carddisp.pl?gene=EXOC2 |
| DSTN | Destrin, Actin Depolymerizing Factor | Protein Coding | 40 | GC20P017550 | 0.563586891 | https://www.genecards.org/cgi-bin/carddisp.pl?gene=DSTN |
| DDX39B | DExD-Box Helicase 39B | Protein Coding | 40 | GC06M031530 | 0.563586891 | https://www.genecards.org/cgi-bin/carddisp.pl?gene=DDX39B |
| ABCF1 | ATP Binding Cassette Subfamily F Member 1 | Protein Coding | 40 | GC06P030571 | 0.563586891 | https://www.genecards.org/cgi-bin/carddisp.pl?gene=ABCF1 |
| ADAM28 | ADAM Metallopeptidase Domain 28 | Protein Coding | 40 | GC08P024294 | 0.563586891 | https://www.genecards.org/cgi-bin/carddisp.pl?gene=ADAM28 |
| CDCA2 | Cell Division Cycle Associated 2 | Protein Coding | 40 | GC08P025458 | 0.563586891 | https://www.genecards.org/cgi-bin/carddisp.pl?gene=CDCA2 |
| CDC5L | Cell Division Cycle 5 Like | Protein Coding | 40 | GC06P044387 | 0.563586891 | https://www.genecards.org/cgi-bin/carddisp.pl?gene=CDC5L |
| EPB41L2 | Erythrocyte Membrane Protein Band 4.1 Like 2 | Protein Coding | 40 | GC06M130820 | 0.563586891 | https://www.genecards.org/cgi-bin/carddisp.pl?gene=EPB41L2 |
| RNF213 | Ring Finger Protein 213 | Protein Coding | 40 | GC17P080260 | 0.563586891 | https://www.genecards.org/cgi-bin/carddisp.pl?gene=RNF213 |
| SUPT5H | SPT5 Homolog, DSIF Elongation Factor Subunit | Protein Coding | 40 | GC19P039436 | 0.563586891 | https://www.genecards.org/cgi-bin/carddisp.pl?gene=SUPT5H |
| SMG6 | SMG6 Nonsense Mediated MRNA Decay Factor | Protein Coding | 40 | GC17M002059 | 0.563586891 | https://www.genecards.org/cgi-bin/carddisp.pl?gene=SMG6 |
| NMUR2 | Neuromedin U Receptor 2 | Protein Coding | 40 | GC05M152391 | 0.563586891 | https://www.genecards.org/cgi-bin/carddisp.pl?gene=NMUR2 |
| NOVA1 | NOVA Alternative Splicing Regulator 1 | Protein Coding | 40 | GC14M026443 | 0.563586891 | https://www.genecards.org/cgi-bin/carddisp.pl?gene=NOVA1 |
| RRBP1 | Ribosome Binding Protein 1 | Protein Coding | 40 | GC20M017613 | 0.563586891 | https://www.genecards.org/cgi-bin/carddisp.pl?gene=RRBP1 |
| SBF2 | SET Binding Factor 2 | Protein Coding | 40 | GC11M009789 | 0.563586891 | https://www.genecards.org/cgi-bin/carddisp.pl?gene=SBF2 |
| PSMD8 | Proteasome 26S Subunit, Non-ATPase 8 | Protein Coding | 40 | GC19P038374 | 0.563586891 | https://www.genecards.org/cgi-bin/carddisp.pl?gene=PSMD8 |
| ZMYND8 | Zinc Finger MYND-Type Containing 8 | Protein Coding | 40 | GC20M047209 | 0.563586891 | https://www.genecards.org/cgi-bin/carddisp.pl?gene=ZMYND8 |
| MFSD2A | Major Facilitator Superfamily Domain Containing 2A | Protein Coding | 39 | GC01P039955 | 0.563586891 | https://www.genecards.org/cgi-bin/carddisp.pl?gene=MFSD2A |
| EIF3K | Eukaryotic Translation Initiation Factor 3 Subunit K | Protein Coding | 39 | GC19P038619 | 0.563586891 | https://www.genecards.org/cgi-bin/carddisp.pl?gene=EIF3K |
| EXOSC5 | Exosome Component 5 | Protein Coding | 39 | GC19M046803 | 0.563586891 | https://www.genecards.org/cgi-bin/carddisp.pl?gene=EXOSC5 |
| EXOSC10 | Exosome Component 10 | Protein Coding | 39 | GC01M011067 | 0.563586891 | https://www.genecards.org/cgi-bin/carddisp.pl?gene=EXOSC10 |
| BFSP1 | Beaded Filament Structural Protein 1 | Protein Coding | 39 | GC20M017493 | 0.563586891 | https://www.genecards.org/cgi-bin/carddisp.pl?gene=BFSP1 |
| WDR12 | WD Repeat Domain 12 | Protein Coding | 39 | GC02M202874 | 0.563586891 | https://www.genecards.org/cgi-bin/carddisp.pl?gene=WDR12 |
| TSR1 | TSR1 Ribosome Maturation Factor | Protein Coding | 39 | GC17M002322 | 0.563586891 | https://www.genecards.org/cgi-bin/carddisp.pl?gene=TSR1 |
| YY1AP1 | YY1 Associated Protein 1 | Protein Coding | 39 | GC01M155659 | 0.563586891 | https://www.genecards.org/cgi-bin/carddisp.pl?gene=YY1AP1 |
| SPTBN4 | Spectrin Beta, Non-Erythrocytic 4 | Protein Coding | 39 | GC19P040466 | 0.563586891 | https://www.genecards.org/cgi-bin/carddisp.pl?gene=SPTBN4 |
| MSTO1 | Misato Mitochondrial Distribution And Morphology Regulator 1 | Protein Coding | 39 | GC01P155610 | 0.563586891 | https://www.genecards.org/cgi-bin/carddisp.pl?gene=MSTO1 |
| SCARF1 | Scavenger Receptor Class F Member 1 | Protein Coding | 39 | GC17M001668 | 0.563586891 | https://www.genecards.org/cgi-bin/carddisp.pl?gene=SCARF1 |
| NUMBL | NUMB Like Endocytic Adaptor Protein | Protein Coding | 39 | GC19M040665 | 0.563586891 | https://www.genecards.org/cgi-bin/carddisp.pl?gene=NUMBL |
| PPP1R10 | Protein Phosphatase 1 Regulatory Subunit 10 | Protein Coding | 39 | GC06M030600 | 0.563586891 | https://www.genecards.org/cgi-bin/carddisp.pl?gene=PPP1R10 |
| NCOA6 | Nuclear Receptor Coactivator 6 | Protein Coding | 39 | GC20M034700 | 0.563586891 | https://www.genecards.org/cgi-bin/carddisp.pl?gene=NCOA6 |
| MRPS12 | Mitochondrial Ribosomal Protein S12 | Protein Coding | 39 | GC19P038930 | 0.563586891 | https://www.genecards.org/cgi-bin/carddisp.pl?gene=MRPS12 |
| NOP58 | NOP58 Ribonucleoprotein | Protein Coding | 39 | GC02P202265 | 0.563586891 | https://www.genecards.org/cgi-bin/carddisp.pl?gene=NOP58 |
| ARID4A | AT-Rich Interaction Domain 4A | Protein Coding | 38 | GC14P058298 | 0.563586891 | https://www.genecards.org/cgi-bin/carddisp.pl?gene=ARID4A |
| CTR9 | CTR9 Homolog, Paf1/RNA Polymerase II Complex Component | Protein Coding | 38 | GC11P010772 | 0.563586891 | https://www.genecards.org/cgi-bin/carddisp.pl?gene=CTR9 |
| BMP8A | Bone Morphogenetic Protein 8a | Protein Coding | 38 | GC01P039492 | 0.563586891 | https://www.genecards.org/cgi-bin/carddisp.pl?gene=BMP8A |
| LGALS7 | Galectin 7 | Protein Coding | 38 | GC19M038770 | 0.563586891 | https://www.genecards.org/cgi-bin/carddisp.pl?gene=LGALS7 |
| IER3 | Immediate Early Response 3 | Protein Coding | 38 | GC06M030743 | 0.563586891 | https://www.genecards.org/cgi-bin/carddisp.pl?gene=IER3 |
| LRIG3 | Leucine Rich Repeats And Immunoglobulin Like Domains 3 | Protein Coding | 38 | GC12M058872 | 0.563586891 | https://www.genecards.org/cgi-bin/carddisp.pl?gene=LRIG3 |
| CLVS1 | Clavesin 1 | Protein Coding | 38 | GC08P060966 | 0.563586891 | https://www.genecards.org/cgi-bin/carddisp.pl?gene=CLVS1 |
| AMIGO2 | Adhesion Molecule With Ig Like Domain 2 | Protein Coding | 38 | GC12M047077 | 0.563586891 | https://www.genecards.org/cgi-bin/carddisp.pl?gene=AMIGO2 |
| ADAMTSL4 | ADAMTS Like 4 | Protein Coding | 38 | GC01P150549 | 0.563586891 | https://www.genecards.org/cgi-bin/carddisp.pl?gene=ADAMTSL4 |
| CCHCR1 | Coiled-Coil Alpha-Helical Rod Protein 1 | Protein Coding | 38 | GC06M046823 | 0.563586891 | https://www.genecards.org/cgi-bin/carddisp.pl?gene=CCHCR1 |
| SPINK2 | Serine Peptidase Inhibitor Kazal Type 2 | Protein Coding | 38 | GC04M056809 | 0.563586891 | https://www.genecards.org/cgi-bin/carddisp.pl?gene=SPINK2 |
| SMYD4 | SET And MYND Domain Containing 4 | Protein Coding | 38 | GC17M001779 | 0.563586891 | https://www.genecards.org/cgi-bin/carddisp.pl?gene=SMYD4 |
| SCAND1 | SCAN Domain Containing 1 | Protein Coding | 38 | GC20M035953 | 0.563586891 | https://www.genecards.org/cgi-bin/carddisp.pl?gene=SCAND1 |
| NCOA7 | Nuclear Receptor Coactivator 7 | Protein Coding | 38 | GC06P125781 | 0.563586891 | https://www.genecards.org/cgi-bin/carddisp.pl?gene=NCOA7 |
| PLEKHG2 | Pleckstrin Homology And RhoGEF Domain Containing G2 | Protein Coding | 38 | GC19P039412 | 0.563586891 | https://www.genecards.org/cgi-bin/carddisp.pl?gene=PLEKHG2 |
| SEMA6C | Semaphorin 6C | Protein Coding | 38 | GC01M151131 | 0.563586891 | https://www.genecards.org/cgi-bin/carddisp.pl?gene=SEMA6C |
| NFKBIL1 | NFKB Inhibitor Like 1 | Protein Coding | 38 | GC06P055199 | 0.563586891 | https://www.genecards.org/cgi-bin/carddisp.pl?gene=NFKBIL1 |
| HNRNPUL1 | Heterogeneous Nuclear Ribonucleoprotein U Like 1 | Protein Coding | 38 | GC19P041262 | 0.563586891 | https://www.genecards.org/cgi-bin/carddisp.pl?gene=HNRNPUL1 |
| RASGRP4 | RAS Guanyl Releasing Protein 4 | Protein Coding | 38 | GC19M038409 | 0.563586891 | https://www.genecards.org/cgi-bin/carddisp.pl?gene=RASGRP4 |
| RBM39 | RNA Binding Motif Protein 39 | Protein Coding | 38 | GC20M035703 | 0.563586891 | https://www.genecards.org/cgi-bin/carddisp.pl?gene=RBM39 |
| SLC35B1 | Solute Carrier Family 35 Member B1 | Protein Coding | 38 | GC17M049700 | 0.563586891 | https://www.genecards.org/cgi-bin/carddisp.pl?gene=SLC35B1 |
| DNAJC1 | DnaJ Heat Shock Protein Family (Hsp40) Member C1 | Protein Coding | 37 | GC10M021762 | 0.563586891 | https://www.genecards.org/cgi-bin/carddisp.pl?gene=DNAJC1 |
| KLF9 | Kruppel Like Factor 9 | Protein Coding | 37 | GC09M070384 | 0.563586891 | https://www.genecards.org/cgi-bin/carddisp.pl?gene=KLF9 |
| HORMAD1 | HORMA Domain Containing 1 | Protein Coding | 37 | GC01M150835 | 0.563586891 | https://www.genecards.org/cgi-bin/carddisp.pl?gene=HORMAD1 |
| LINGO2 | Leucine Rich Repeat And Ig Domain Containing 2 | Protein Coding | 37 | GC09M027940 | 0.563586891 | https://www.genecards.org/cgi-bin/carddisp.pl?gene=LINGO2 |
| GPR26 | G Protein-Coupled Receptor 26 | Protein Coding | 37 | GC10P123666 | 0.563586891 | https://www.genecards.org/cgi-bin/carddisp.pl?gene=GPR26 |
| KCNK17 | Potassium Two Pore Domain Channel Subfamily K Member 17 | Protein Coding | 37 | GC06M047057 | 0.563586891 | https://www.genecards.org/cgi-bin/carddisp.pl?gene=KCNK17 |
| LYZL1 | Lysozyme Like 1 | Protein Coding | 37 | GC10P029297 | 0.563586891 | https://www.genecards.org/cgi-bin/carddisp.pl?gene=LYZL1 |
| CMTM8 | CKLF Like MARVEL Transmembrane Domain Containing 8 | Protein Coding | 37 | GC03P032238 | 0.563586891 | https://www.genecards.org/cgi-bin/carddisp.pl?gene=CMTM8 |
| CAPN12 | Calpain 12 | Protein Coding | 37 | GC19M038730 | 0.563586891 | https://www.genecards.org/cgi-bin/carddisp.pl?gene=CAPN12 |
| BICC1 | BicC Family RNA Binding Protein 1 | Protein Coding | 37 | GC10P058513 | 0.563586891 | https://www.genecards.org/cgi-bin/carddisp.pl?gene=BICC1 |
| CFDP1 | Craniofacial Development Protein 1 | Protein Coding | 37 | GC16M075294 | 0.563586891 | https://www.genecards.org/cgi-bin/carddisp.pl?gene=CFDP1 |
| TCF19 | Transcription Factor 19 | Protein Coding | 37 | GC06P055190 | 0.563586891 | https://www.genecards.org/cgi-bin/carddisp.pl?gene=TCF19 |
| RBP7 | Retinol Binding Protein 7 | Protein Coding | 37 | GC01P009997 | 0.563586891 | https://www.genecards.org/cgi-bin/carddisp.pl?gene=RBP7 |
| ZNF607 | Zinc Finger Protein 607 | Protein Coding | 37 | GC19M037696 | 0.563586891 | https://www.genecards.org/cgi-bin/carddisp.pl?gene=ZNF607 |
| WDR81 | WD Repeat Domain 81 | Protein Coding | 37 | GC17P001716 | 0.563586891 | https://www.genecards.org/cgi-bin/carddisp.pl?gene=WDR81 |
| TRIM26 | Tripartite Motif Containing 26 | Protein Coding | 37 | GC06M030184 | 0.563586891 | https://www.genecards.org/cgi-bin/carddisp.pl?gene=TRIM26 |
| UACA | Uveal Autoantigen With Coiled-Coil Domains And Ankyrin Repeats | Protein Coding | 37 | GC15M070654 | 0.563586891 | https://www.genecards.org/cgi-bin/carddisp.pl?gene=UACA |
| ST7 | Suppression Of Tumorigenicity 7 | Protein Coding | 37 | GC07P117037 | 0.563586891 | https://www.genecards.org/cgi-bin/carddisp.pl?gene=ST7 |
| SNX16 | Sorting Nexin 16 | Protein Coding | 37 | GC08M081799 | 0.563586891 | https://www.genecards.org/cgi-bin/carddisp.pl?gene=SNX16 |
| PMF1 | Polyamine Modulated Factor 1 | Protein Coding | 37 | GC01P156212 | 0.563586891 | https://www.genecards.org/cgi-bin/carddisp.pl?gene=PMF1 |
| PLAGL2 | PLAG1 Like Zinc Finger 2 | Protein Coding | 37 | GC20M032192 | 0.563586891 | https://www.genecards.org/cgi-bin/carddisp.pl?gene=PLAGL2 |
| MOAP1 | Modulator Of Apoptosis 1 | Protein Coding | 37 | GC14M093182 | 0.563586891 | https://www.genecards.org/cgi-bin/carddisp.pl?gene=MOAP1 |
| PRCC | Proline Rich Mitotic Checkpoint Control Factor | Protein Coding | 37 | GC01P156750 | 0.563586891 | https://www.genecards.org/cgi-bin/carddisp.pl?gene=PRCC |
| PAF1 | PAF1 Homolog, Paf1/RNA Polymerase II Complex Component | Protein Coding | 37 | GC19M039385 | 0.563586891 | https://www.genecards.org/cgi-bin/carddisp.pl?gene=PAF1 |
| MUC13 | Mucin 13, Cell Surface Associated | Protein Coding | 37 | GC03M124905 | 0.563586891 | https://www.genecards.org/cgi-bin/carddisp.pl?gene=MUC13 |
| ODF1 | Outer Dense Fiber Of Sperm Tails 1 | Protein Coding | 37 | GC08P102551 | 0.563586891 | https://www.genecards.org/cgi-bin/carddisp.pl?gene=ODF1 |
| MEGF8 | Multiple EGF Like Domains 8 | Protein Coding | 37 | GC19P042325 | 0.563586891 | https://www.genecards.org/cgi-bin/carddisp.pl?gene=MEGF8 |
| RPRD2 | Regulation Of Nuclear Pre-MRNA Domain Containing 2 | Protein Coding | 37 | GC01P150363 | 0.563586891 | https://www.genecards.org/cgi-bin/carddisp.pl?gene=RPRD2 |
| SGSM2 | Small G Protein Signaling Modulator 2 | Protein Coding | 37 | GC17P002337 | 0.563586891 | https://www.genecards.org/cgi-bin/carddisp.pl?gene=SGSM2 |
| MRPS21 | Mitochondrial Ribosomal Protein S21 | Protein Coding | 37 | GC01P150294 | 0.563586891 | https://www.genecards.org/cgi-bin/carddisp.pl?gene=MRPS21 |
| MPPED2 | Metallophosphoesterase Domain Containing 2 | Protein Coding | 37 | GC11M030406 | 0.563586891 | https://www.genecards.org/cgi-bin/carddisp.pl?gene=MPPED2 |
| KBTBD8 | Kelch Repeat And BTB Domain Containing 8 | Protein Coding | 37 | GC03P066998 | 0.563586891 | https://www.genecards.org/cgi-bin/carddisp.pl?gene=KBTBD8 |
| RBM12 | RNA Binding Motif Protein 12 | Protein Coding | 37 | GC20M035648 | 0.563586891 | https://www.genecards.org/cgi-bin/carddisp.pl?gene=RBM12 |
| ISG20L2 | Interferon Stimulated Exonuclease Gene 20 Like 2 | Protein Coding | 36 | GC01M156723 | 0.563586891 | https://www.genecards.org/cgi-bin/carddisp.pl?gene=ISG20L2 |
| FGD6 | FYVE, RhoGEF And PH Domain Containing 6 | Protein Coding | 36 | GC12M095076 | 0.563586891 | https://www.genecards.org/cgi-bin/carddisp.pl?gene=FGD6 |
| GPR149 | G Protein-Coupled Receptor 149 | Protein Coding | 36 | GC03M154346 | 0.563586891 | https://www.genecards.org/cgi-bin/carddisp.pl?gene=GPR149 |
| ETV3 | ETS Variant Transcription Factor 3 | Protein Coding | 36 | GC01M157121 | 0.563586891 | https://www.genecards.org/cgi-bin/carddisp.pl?gene=ETV3 |
| EYS | Eyes Shut Homolog | Protein Coding | 36 | GC06M063719 | 0.563586891 | https://www.genecards.org/cgi-bin/carddisp.pl?gene=EYS |
| ALPK2 | Alpha Kinase 2 | Protein Coding | 36 | GC18M058481 | 0.563586891 | https://www.genecards.org/cgi-bin/carddisp.pl?gene=ALPK2 |
| BNIP1 | BCL2 Interacting Protein 1 | Protein Coding | 36 | GC05P173144 | 0.563586891 | https://www.genecards.org/cgi-bin/carddisp.pl?gene=BNIP1 |
| ZNF526 | Zinc Finger Protein 526 | Protein Coding | 36 | GC19P042220 | 0.563586891 | https://www.genecards.org/cgi-bin/carddisp.pl?gene=ZNF526 |
| ONECUT2 | One Cut Homeobox 2 | Protein Coding | 36 | GC18P057436 | 0.563586891 | https://www.genecards.org/cgi-bin/carddisp.pl?gene=ONECUT2 |
| TM9SF4 | Transmembrane 9 Superfamily Member 4 | Protein Coding | 36 | GC20P032109 | 0.563586891 | https://www.genecards.org/cgi-bin/carddisp.pl?gene=TM9SF4 |
| YIPF5 | Yip1 Domain Family Member 5 | Protein Coding | 36 | GC05M144158 | 0.563586891 | https://www.genecards.org/cgi-bin/carddisp.pl?gene=YIPF5 |
| TMEM91 | Transmembrane Protein 91 | Protein Coding | 36 | GC19P041365 | 0.563586891 | https://www.genecards.org/cgi-bin/carddisp.pl?gene=TMEM91 |
| UBTD2 | Ubiquitin Domain Containing 2 | Protein Coding | 36 | GC05M172209 | 0.563586891 | https://www.genecards.org/cgi-bin/carddisp.pl?gene=UBTD2 |
| OXCT2 | 3-Oxoacid CoA-Transferase 2 | Protein Coding | 36 | GC01M039776 | 0.563586891 | https://www.genecards.org/cgi-bin/carddisp.pl?gene=OXCT2 |
| SERPINB12 | Serpin Family B Member 12 | Protein Coding | 36 | GC18P063556 | 0.563586891 | https://www.genecards.org/cgi-bin/carddisp.pl?gene=SERPINB12 |
| RASGEF1C | RasGEF Domain Family Member 1C | Protein Coding | 36 | GC05M180101 | 0.563586891 | https://www.genecards.org/cgi-bin/carddisp.pl?gene=RASGEF1C |
| SAMD4B | Sterile Alpha Motif Domain Containing 4B | Protein Coding | 36 | GC19P039342 | 0.563586891 | https://www.genecards.org/cgi-bin/carddisp.pl?gene=SAMD4B |
| KIF18B | Kinesin Family Member 18B | Protein Coding | 35 | GC17M044924 | 0.563586891 | https://www.genecards.org/cgi-bin/carddisp.pl?gene=KIF18B |
| GON4L | Gon-4 Like | Protein Coding | 35 | GC01M155754 | 0.563586891 | https://www.genecards.org/cgi-bin/carddisp.pl?gene=GON4L |
| NBEAL1 | Neurobeachin Like 1 | Protein Coding | 35 | GC02P203014 | 0.563586891 | https://www.genecards.org/cgi-bin/carddisp.pl?gene=NBEAL1 |
| ZBTB10 | Zinc Finger And BTB Domain Containing 10 | Protein Coding | 35 | GC08P080485 | 0.563586891 | https://www.genecards.org/cgi-bin/carddisp.pl?gene=ZBTB10 |
| TAAR6 | Trace Amine Associated Receptor 6 | Protein Coding | 35 | GC06P132570 | 0.563586891 | https://www.genecards.org/cgi-bin/carddisp.pl?gene=TAAR6 |
| PPP1R18 | Protein Phosphatase 1 Regulatory Subunit 18 | Protein Coding | 35 | GC06M046794 | 0.563586891 | https://www.genecards.org/cgi-bin/carddisp.pl?gene=PPP1R18 |
| SMG5 | SMG5 Nonsense Mediated MRNA Decay Factor | Protein Coding | 35 | GC01M156274 | 0.563586891 | https://www.genecards.org/cgi-bin/carddisp.pl?gene=SMG5 |
| SLC25A44 | Solute Carrier Family 25 Member 44 | Protein Coding | 35 | GC01P156195 | 0.563586891 | https://www.genecards.org/cgi-bin/carddisp.pl?gene=SLC25A44 |
| NXPH3 | Neurexophilin 3 | Protein Coding | 35 | GC17P049575 | 0.563586891 | https://www.genecards.org/cgi-bin/carddisp.pl?gene=NXPH3 |
| C1orf127 | Chromosome 1 Open Reading Frame 127 | Protein Coding | 34 | GC01M010946 | 0.563586891 | https://www.genecards.org/cgi-bin/carddisp.pl?gene=C1orf127 |
| C1QL3 | Complement C1q Like 3 | Protein Coding | 34 | GC10M016513 | 0.563586891 | https://www.genecards.org/cgi-bin/carddisp.pl?gene=C1QL3 |
| HUS1B | HUS1 Checkpoint Clamp Component B | Protein Coding | 34 | GC06M000655 | 0.563586891 | https://www.genecards.org/cgi-bin/carddisp.pl?gene=HUS1B |
| DIP2C | Disco Interacting Protein 2 Homolog C | Protein Coding | 34 | GC10M000274 | 0.563586891 | https://www.genecards.org/cgi-bin/carddisp.pl?gene=DIP2C |
| CARF | Calcium Responsive Transcription Factor | Protein Coding | 34 | GC02P202912 | 0.563586891 | https://www.genecards.org/cgi-bin/carddisp.pl?gene=CARF |
| CTAGE1 | Cutaneous T Cell Lymphoma-Associated Antigen 1 | Protein Coding | 34 | GC18M022413 | 0.563586891 | https://www.genecards.org/cgi-bin/carddisp.pl?gene=CTAGE1 |
| WSCD1 | WSC Domain Containing 1 | Protein Coding | 34 | GC17P006057 | 0.563586891 | https://www.genecards.org/cgi-bin/carddisp.pl?gene=WSCD1 |
| SPAG4 | Sperm Associated Antigen 4 | Protein Coding | 34 | GC20P035615 | 0.563586891 | https://www.genecards.org/cgi-bin/carddisp.pl?gene=SPAG4 |
| NRM | Nurim | Protein Coding | 34 | GC06M046796 | 0.563586891 | https://www.genecards.org/cgi-bin/carddisp.pl?gene=NRM |
| POC5 | POC5 Centriolar Protein | Protein Coding | 34 | GC05M075674 | 0.563586891 | https://www.genecards.org/cgi-bin/carddisp.pl?gene=POC5 |
| OR4C3 | Olfactory Receptor Family 4 Subfamily C Member 3 | Protein Coding | 34 | GC11P048346 | 0.563586891 | https://www.genecards.org/cgi-bin/carddisp.pl?gene=OR4C3 |
| RPAP3 | RNA Polymerase II Associated Protein 3 | Protein Coding | 34 | GC12M047661 | 0.563586891 | https://www.genecards.org/cgi-bin/carddisp.pl?gene=RPAP3 |
| COQ8B | Coenzyme Q8B | Protein Coding | 33 | GC19M046773 | 0.563586891 | https://www.genecards.org/cgi-bin/carddisp.pl?gene=COQ8B |
| LARP4B | La Ribonucleoprotein 4B | Protein Coding | 33 | GC10M000806 | 0.563586891 | https://www.genecards.org/cgi-bin/carddisp.pl?gene=LARP4B |
| GPANK1 | G-Patch Domain And Ankyrin Repeats 1 | Protein Coding | 33 | GC06M046885 | 0.563586891 | https://www.genecards.org/cgi-bin/carddisp.pl?gene=GPANK1 |
| CCM2L | CCM2 Like Scaffold Protein | Protein Coding | 33 | GC20P032010 | 0.563586891 | https://www.genecards.org/cgi-bin/carddisp.pl?gene=CCM2L |
| CNPY1 | Canopy FGF Signaling Regulator 1 | Protein Coding | 33 | GC07M155474 | 0.563586891 | https://www.genecards.org/cgi-bin/carddisp.pl?gene=CNPY1 |
| CATSPERG | Cation Channel Sperm Associated Auxiliary Subunit Gamma | Protein Coding | 33 | GC19P040442 | 0.563586891 | https://www.genecards.org/cgi-bin/carddisp.pl?gene=CATSPERG |
| CALHM2 | Calcium Homeostasis Modulator Family Member 2 | Protein Coding | 33 | GC10M103446 | 0.563586891 | https://www.genecards.org/cgi-bin/carddisp.pl?gene=CALHM2 |
| ZNF574 | Zinc Finger Protein 574 | Protein Coding | 33 | GC19P042068 | 0.563586891 | https://www.genecards.org/cgi-bin/carddisp.pl?gene=ZNF574 |
| ZNF573 | Zinc Finger Protein 573 | Protein Coding | 33 | GC19M037735 | 0.563586891 | https://www.genecards.org/cgi-bin/carddisp.pl?gene=ZNF573 |
| ZCCHC14 | Zinc Finger CCHC-Type Containing 14 | Protein Coding | 33 | GC16M087406 | 0.563586891 | https://www.genecards.org/cgi-bin/carddisp.pl?gene=ZCCHC14 |
| TMEM51 | Transmembrane Protein 51 | Protein Coding | 33 | GC01P015225 | 0.563586891 | https://www.genecards.org/cgi-bin/carddisp.pl?gene=TMEM51 |
| TMEM68 | Transmembrane Protein 68 | Protein Coding | 33 | GC08M055696 | 0.563586891 | https://www.genecards.org/cgi-bin/carddisp.pl?gene=TMEM68 |
| PRRC2A | Proline Rich Coiled-Coil 2A | Protein Coding | 33 | GC06P055204 | 0.563586891 | https://www.genecards.org/cgi-bin/carddisp.pl?gene=PRRC2A |
| ROMO1 | Reactive Oxygen Species Modulator 1 | Protein Coding | 33 | GC20P035699 | 0.563586891 | https://www.genecards.org/cgi-bin/carddisp.pl?gene=ROMO1 |
| RPP21 | Ribonuclease P/MRP Subunit P21 | Protein Coding | 33 | GC06P030345 | 0.563586891 | https://www.genecards.org/cgi-bin/carddisp.pl?gene=RPP21 |
| RSPH14 | Radial Spoke Head 14 Homolog | Protein Coding | 33 | GC22M023059 | 0.563586891 | https://www.genecards.org/cgi-bin/carddisp.pl?gene=RSPH14 |
| RINL | Ras And Rab Interactor Like | Protein Coding | 33 | GC19M038867 | 0.563586891 | https://www.genecards.org/cgi-bin/carddisp.pl?gene=RINL |
| ZFAND1 | Zinc Finger AN1-Type Containing 1 | Protein Coding | 33 | GC08M081701 | 0.563586891 | https://www.genecards.org/cgi-bin/carddisp.pl?gene=ZFAND1 |
| FHAD1 | Forkhead Associated Phosphopeptide Binding Domain 1 | Protein Coding | 32 | GC01P015248 | 0.563586891 | https://www.genecards.org/cgi-bin/carddisp.pl?gene=FHAD1 |
| FBXO17 | F-Box Protein 17 | Protein Coding | 32 | GC19M038941 | 0.563586891 | https://www.genecards.org/cgi-bin/carddisp.pl?gene=FBXO17 |
| CCDC97 | Coiled-Coil Domain Containing 97 | Protein Coding | 32 | GC19P041327 | 0.563586891 | https://www.genecards.org/cgi-bin/carddisp.pl?gene=CCDC97 |
| CEACAM21 | CEA Cell Adhesion Molecule 21 | Protein Coding | 32 | GC19P041550 | 0.563586891 | https://www.genecards.org/cgi-bin/carddisp.pl?gene=CEACAM21 |
| XKR4 | XK Related 4 | Protein Coding | 32 | GC08P055102 | 0.563586891 | https://www.genecards.org/cgi-bin/carddisp.pl?gene=XKR4 |
| RNF113B | Ring Finger Protein 113B | Protein Coding | 32 | GC13M098175 | 0.563586891 | https://www.genecards.org/cgi-bin/carddisp.pl?gene=RNF113B |
| POLR1H | RNA Polymerase I Subunit H | Protein Coding | 32 | GC06P057843 | 0.563586891 | https://www.genecards.org/cgi-bin/carddisp.pl?gene=POLR1H |
| PRR3 | Proline Rich 3 | Protein Coding | 32 | GC06P055177 | 0.563586891 | https://www.genecards.org/cgi-bin/carddisp.pl?gene=PRR3 |
| PSTK | Phosphoseryl-TRNA Kinase | Protein Coding | 32 | GC10P122954 | 0.563586891 | https://www.genecards.org/cgi-bin/carddisp.pl?gene=PSTK |
| C1orf56 | Chromosome 1 Open Reading Frame 56 | Protein Coding | 31 | GC01P151047 | 0.563586891 | https://www.genecards.org/cgi-bin/carddisp.pl?gene=C1orf56 |
| LGALS7B | Galectin 7B | Protein Coding | 31 | GC19P040456 | 0.563586891 | https://www.genecards.org/cgi-bin/carddisp.pl?gene=LGALS7B |
| IGSF9B | Immunoglobulin Superfamily Member 9B | Protein Coding | 31 | GC11M133934 | 0.563586891 | https://www.genecards.org/cgi-bin/carddisp.pl?gene=IGSF9B |
| LRRIQ3 | Leucine Rich Repeats And IQ Motif Containing 3 | Protein Coding | 31 | GC01M074026 | 0.563586891 | https://www.genecards.org/cgi-bin/carddisp.pl?gene=LRRIQ3 |
| ENDOV | Endonuclease V | Protein Coding | 31 | GC17P080415 | 0.563586891 | https://www.genecards.org/cgi-bin/carddisp.pl?gene=ENDOV |
| FBXO27 | F-Box Protein 27 | Protein Coding | 31 | GC19M046737 | 0.563586891 | https://www.genecards.org/cgi-bin/carddisp.pl?gene=FBXO27 |
| FAM98C | Family With Sequence Similarity 98 Member C | Protein Coding | 31 | GC19P038403 | 0.563586891 | https://www.genecards.org/cgi-bin/carddisp.pl?gene=FAM98C |
| ARMS2 | Age-Related Maculopathy Susceptibility 2 | Protein Coding | 31 | GC10P122454 | 0.563586891 | https://www.genecards.org/cgi-bin/carddisp.pl?gene=ARMS2 |
| BTBD16 | BTB Domain Containing 16 | Protein Coding | 31 | GC10P122271 | 0.563586891 | https://www.genecards.org/cgi-bin/carddisp.pl?gene=BTBD16 |
| ZNF438 | Zinc Finger Protein 438 | Protein Coding | 31 | GC10M030820 | 0.563586891 | https://www.genecards.org/cgi-bin/carddisp.pl?gene=ZNF438 |
| SAYSD1 | SAYSVFN Motif Domain Containing 1 | Protein Coding | 31 | GC06M047053 | 0.563586891 | https://www.genecards.org/cgi-bin/carddisp.pl?gene=SAYSD1 |
| C6orf136 | Chromosome 6 Open Reading Frame 136 | Protein Coding | 30 | GC06P055179 | 0.563586891 | https://www.genecards.org/cgi-bin/carddisp.pl?gene=C6orf136 |
| C8orf48 | Chromosome 8 Open Reading Frame 48 | Protein Coding | 30 | GC08P013566 | 0.563586891 | https://www.genecards.org/cgi-bin/carddisp.pl?gene=C8orf48 |
| C19orf33 | Chromosome 19 Open Reading Frame 33 | Protein Coding | 30 | GC19P038304 | 0.563586891 | https://www.genecards.org/cgi-bin/carddisp.pl?gene=C19orf33 |
| MBOAT4 | Membrane Bound O-Acyltransferase Domain Containing 4 | Protein Coding | 30 | GC08M030131 | 0.563586891 | https://www.genecards.org/cgi-bin/carddisp.pl?gene=MBOAT4 |
| ATP5MC2 | ATP Synthase Membrane Subunit C Locus 2 | Protein Coding | 30 | GC12M053654 | 0.563586891 | https://www.genecards.org/cgi-bin/carddisp.pl?gene=ATP5MC2 |
| SERTAD3 | SERTA Domain Containing 3 | Protein Coding | 30 | GC19M046764 | 0.563586891 | https://www.genecards.org/cgi-bin/carddisp.pl?gene=SERTAD3 |
| NOL4L | Nucleolar Protein 4 Like | Protein Coding | 30 | GC20M032443 | 0.563586891 | https://www.genecards.org/cgi-bin/carddisp.pl?gene=NOL4L |
| TMEM251 | Transmembrane Protein 251 | Protein Coding | 30 | GC14P093185 | 0.563586891 | https://www.genecards.org/cgi-bin/carddisp.pl?gene=TMEM251 |
| CYRIB | CYFIP Related Rac1 Interactor B | Protein Coding | 29 | GC08M129840 | 0.563586891 | https://www.genecards.org/cgi-bin/carddisp.pl?gene=CYRIB |
| LY6G5B | Lymphocyte Antigen 6 Family Member G5B | Protein Coding | 29 | GC06P055208 | 0.563586891 | https://www.genecards.org/cgi-bin/carddisp.pl?gene=LY6G5B |
| CNBD2 | Cyclic Nucleotide Binding Domain Containing 2 | Protein Coding | 29 | GC20P035954 | 0.563586891 | https://www.genecards.org/cgi-bin/carddisp.pl?gene=CNBD2 |
| ANKDD1B | Ankyrin Repeat And Death Domain Containing 1B | Protein Coding | 29 | GC05P075611 | 0.563586891 | https://www.genecards.org/cgi-bin/carddisp.pl?gene=ANKDD1B |
| TSACC | TSSK6 Activating Cochaperone | Protein Coding | 29 | GC01P156337 | 0.563586891 | https://www.genecards.org/cgi-bin/carddisp.pl?gene=TSACC |
| PCED1B | PC-Esterase Domain Containing 1B | Protein Coding | 29 | GC12P047079 | 0.563586891 | https://www.genecards.org/cgi-bin/carddisp.pl?gene=PCED1B |
| PRR13 | Proline Rich 13 | Protein Coding | 29 | GC12P053442 | 0.563586891 | https://www.genecards.org/cgi-bin/carddisp.pl?gene=PRR13 |
| METTL16 | Methyltransferase Like 16 | Protein Coding | 29 | GC17M002405 | 0.563586891 | https://www.genecards.org/cgi-bin/carddisp.pl?gene=METTL16 |
| KIAA0040 | KIAA0040 | Protein Coding | 29 | GC01M175126 | 0.563586891 | https://www.genecards.org/cgi-bin/carddisp.pl?gene=KIAA0040 |
| FAM86B2 | Family With Sequence Similarity 86 Member B2 | Protein Coding | 28 | GC08M012425 | 0.563586891 | https://www.genecards.org/cgi-bin/carddisp.pl?gene=FAM86B2 |
| TMEM145 | Transmembrane Protein 145 | Protein Coding | 28 | GC19P042313 | 0.563586891 | https://www.genecards.org/cgi-bin/carddisp.pl?gene=TMEM145 |
| OR4S1 | Olfactory Receptor Family 4 Subfamily S Member 1 | Protein Coding | 28 | GC11P048327 | 0.563586891 | https://www.genecards.org/cgi-bin/carddisp.pl?gene=OR4S1 |
| DRICH1 | Aspartate Rich 1 | Protein Coding | 27 | GC22M023690 | 0.563586891 | https://www.genecards.org/cgi-bin/carddisp.pl?gene=DRICH1 |
| ATP23 | ATP23 Metallopeptidase And ATP Synthase Assembly Factor Homolog | Protein Coding | 27 | GC12P057942 | 0.563586891 | https://www.genecards.org/cgi-bin/carddisp.pl?gene=ATP23 |
| BORCS7 | BLOC-1 Related Complex Subunit 7 | Protein Coding | 27 | GC10P102857 | 0.563586891 | https://www.genecards.org/cgi-bin/carddisp.pl?gene=BORCS7 |
| JCAD | Junctional Cadherin 5 Associated | Protein Coding | 26 | GC10M030012 | 0.563586891 | https://www.genecards.org/cgi-bin/carddisp.pl?gene=JCAD |
| EFCAB9 | EF-Hand Calcium Binding Domain 9 | Protein Coding | 26 | GC05P172198 | 0.563586891 | https://www.genecards.org/cgi-bin/carddisp.pl?gene=EFCAB9 |
| RPEL1 | Ribulose-5-Phosphate-3-Epimerase Like 1 | Protein Coding | 26 | GC10P103246 | 0.563586891 | https://www.genecards.org/cgi-bin/carddisp.pl?gene=RPEL1 |
| SAPCD1 | Suppressor APC Domain Containing 1 | Protein Coding | 26 | GC06P055217 | 0.563586891 | https://www.genecards.org/cgi-bin/carddisp.pl?gene=SAPCD1 |
| METTL25B | Methyltransferase Like 25B | Protein Coding | 25 | GC01P156731 | 0.563586891 | https://www.genecards.org/cgi-bin/carddisp.pl?gene=METTL25B |
| KHDC4 | KH Domain Containing 4, Pre-MRNA Splicing Factor | Protein Coding | 25 | GC01M155913 | 0.563586891 | https://www.genecards.org/cgi-bin/carddisp.pl?gene=KHDC4 |
| HMHB1 | Histocompatibility Minor HB-1 | Protein Coding | 25 | GC05P143812 | 0.563586891 | https://www.genecards.org/cgi-bin/carddisp.pl?gene=HMHB1 |
| HLA-H | Major Histocompatibility Complex, Class I, H (Pseudogene) | Pseudogene | 25 | GC06P055152 | 0.563586891 | https://www.genecards.org/cgi-bin/carddisp.pl?gene=HLA-H |
| SKIDA1 | SKI/DACH Domain Containing 1 | Protein Coding | 25 | GC10M021513 | 0.563586891 | https://www.genecards.org/cgi-bin/carddisp.pl?gene=SKIDA1 |
| DOCK8-AS1 | DOCK8 Antisense RNA 1 | RNA Gene | 21 | GC09M000213 | 0.563586891 | https://www.genecards.org/cgi-bin/carddisp.pl?gene=DOCK8-AS1 |
| TRIM39-RPP21 | TRIM39-RPP21 Readthrough | Protein Coding | 21 | GC06P055173 | 0.563586891 | https://www.genecards.org/cgi-bin/carddisp.pl?gene=TRIM39-RPP21 |
| PSORS1C3 | Psoriasis Susceptibility 1 Candidate 3 | RNA Gene | 20 | GC06M046824 | 0.563586891 | https://www.genecards.org/cgi-bin/carddisp.pl?gene=PSORS1C3 |
| TMEM51-AS1 | TMEM51 Antisense RNA 1 | RNA Gene | 19 | GC01M015111 | 0.563586891 | https://www.genecards.org/cgi-bin/carddisp.pl?gene=TMEM51-AS1 |
| HLA-J | Major Histocompatibility Complex, Class I, J (Pseudogene) | Pseudogene | 18 | GC06P055164 | 0.563586891 | https://www.genecards.org/cgi-bin/carddisp.pl?gene=HLA-J |
| CYP2G1P | Cytochrome P450 Family 2 Subfamily G Member 1, Pseudogene | Pseudogene | 18 | GC19P040890 | 0.563586891 | https://www.genecards.org/cgi-bin/carddisp.pl?gene=CYP2G1P |
| PAPPA-AS1 | PAPPA Antisense RNA 1 | RNA Gene | 18 | GC09M116398 | 0.563586891 | https://www.genecards.org/cgi-bin/carddisp.pl?gene=PAPPA-AS1 |
| LINC00200 | Long Intergenic Non-Protein Coding RNA 200 | RNA Gene | 17 | GC10P001158 | 0.563586891 | https://www.genecards.org/cgi-bin/carddisp.pl?gene=LINC00200 |
| LINC00342 | Long Intergenic Non-Protein Coding RNA 342 | RNA Gene | 17 | GC02M095807 | 0.563586891 | https://www.genecards.org/cgi-bin/carddisp.pl?gene=LINC00342 |
| HCG18 | HLA Complex Group 18 | RNA Gene | 16 | GC06M046781 | 0.563586891 | https://www.genecards.org/cgi-bin/carddisp.pl?gene=HCG18 |
| LINC00877 | Long Intergenic Non-Protein Coding RNA 877 | RNA Gene | 16 | GC03M071897 | 0.563586891 | https://www.genecards.org/cgi-bin/carddisp.pl?gene=LINC00877 |
| LINC00243 | Long Intergenic Non-Protein Coding RNA 243 | RNA Gene | 16 | GC06M046801 | 0.563586891 | https://www.genecards.org/cgi-bin/carddisp.pl?gene=LINC00243 |
| SNORA55 | Small Nucleolar RNA, H/ACA Box 55 | RNA Gene | 16 | GC01M039567 | 0.563586891 | https://www.genecards.org/cgi-bin/carddisp.pl?gene=SNORA55 |
| HCG20 | HLA Complex Group 20 | RNA Gene | 15 | GC06P055183 | 0.563586891 | https://www.genecards.org/cgi-bin/carddisp.pl?gene=HCG20 |
| HCG4B | HLA Complex Group 4B | RNA Gene | 15 | GC06M046769 | 0.563586891 | https://www.genecards.org/cgi-bin/carddisp.pl?gene=HCG4B |
| ZMIZ1-AS1 | ZMIZ1 Antisense RNA 1 | RNA Gene | 15 | GC10M078943 | 0.563586891 | https://www.genecards.org/cgi-bin/carddisp.pl?gene=ZMIZ1-AS1 |
| LINC00707 | Long Intergenic Non-Protein Coding RNA 707 | RNA Gene | 15 | GC10P006780 | 0.563586891 | https://www.genecards.org/cgi-bin/carddisp.pl?gene=LINC00707 |
| FALEC | Focally Amplified Long Non-Coding RNA In Epithelial Cancer | RNA Gene | 14 | GC01P150515 | 0.563586891 | https://www.genecards.org/cgi-bin/carddisp.pl?gene=FALEC |
| ZNRD1ASP | Zinc Ribbon Domain Containing 1 Antisense, Pseudogene | Pseudogene | 14 | GC06M046773 | 0.563586891 | https://www.genecards.org/cgi-bin/carddisp.pl?gene=ZNRD1ASP |
| SNORD91A | Small Nucleolar RNA, C/D Box 91A | RNA Gene | 14 | GC17M002330 | 0.563586891 | https://www.genecards.org/cgi-bin/carddisp.pl?gene=SNORD91A |
| SNORD91B | Small Nucleolar RNA, C/D Box 91B | RNA Gene | 14 | GC17M002329 | 0.563586891 | https://www.genecards.org/cgi-bin/carddisp.pl?gene=SNORD91B |
| LMCD1-AS1 | LMCD1 Antisense RNA 1 | RNA Gene | 13 | GC03M007996 | 0.563586891 | https://www.genecards.org/cgi-bin/carddisp.pl?gene=LMCD1-AS1 |
| FAM230I | Family With Sequence Similarity 230 Member I | RNA Gene | 13 | GC22M023464 | 0.563586891 | https://www.genecards.org/cgi-bin/carddisp.pl?gene=FAM230I |
| MSTO2P | Misato Family Member 2, Pseudogene | Pseudogene | 13 | GC01P155745 | 0.563586891 | https://www.genecards.org/cgi-bin/carddisp.pl?gene=MSTO2P |
| PLCE1-AS1 | PLCE1 Antisense RNA 1 | RNA Gene | 13 | GC10M094272 | 0.563586891 | https://www.genecards.org/cgi-bin/carddisp.pl?gene=PLCE1-AS1 |
| PPIEL | Peptidylprolyl Isomerase E Like Pseudogene | Pseudogene | 13 | GC01M039522 | 0.563586891 | https://www.genecards.org/cgi-bin/carddisp.pl?gene=PPIEL |
| MCHR2-AS1 | MCHR2 Antisense RNA 1 | RNA Gene | 13 | GC06P099993 | 0.563586891 | https://www.genecards.org/cgi-bin/carddisp.pl?gene=MCHR2-AS1 |
| ASTN2-AS1 | ASTN2 Antisense RNA 1 | RNA Gene | 12 | GC09P116577 | 0.563586891 | https://www.genecards.org/cgi-bin/carddisp.pl?gene=ASTN2-AS1 |
| HCG21 | HLA Complex Group 21 | RNA Gene | 12 | GC06M030945 | 0.563586891 | https://www.genecards.org/cgi-bin/carddisp.pl?gene=HCG21 |
| LINC01252 | Long Intergenic Non-Protein Coding RNA 1252 | RNA Gene | 12 | GC12P012038 | 0.563586891 | https://www.genecards.org/cgi-bin/carddisp.pl?gene=LINC01252 |
| HLA-K | Major Histocompatibility Complex, Class I, K (Pseudogene) | Pseudogene | 12 | GC06P055159 | 0.563586891 | https://www.genecards.org/cgi-bin/carddisp.pl?gene=HLA-K |
| ASH1L-AS1 | ASH1L Antisense RNA 1 | RNA Gene | 12 | GC01P155561 | 0.563586891 | https://www.genecards.org/cgi-bin/carddisp.pl?gene=ASH1L-AS1 |
| LOC100288866 | Uncharacterized LOC100288866 | RNA Gene | 12 | GC17M049505 | 0.563586891 | https://www.genecards.org/cgi-bin/carddisp.pl?gene=LOC100288866 |
| RNF213-AS1 | RNF213 Antisense RNA 1 | RNA Gene | 12 | GC17M080335 | 0.563586891 | https://www.genecards.org/cgi-bin/carddisp.pl?gene=RNF213-AS1 |
| LOC101927839 | Uncharacterized LOC101927839 | RNA Gene | 12 | GC17P002232 | 0.563586891 | https://www.genecards.org/cgi-bin/carddisp.pl?gene=LOC101927839 |
| UFL1-AS1 | UFL1 Antisense RNA 1 | RNA Gene | 12 | GC06M096037 | 0.563586891 | https://www.genecards.org/cgi-bin/carddisp.pl?gene=UFL1-AS1 |
| LINC01828 | Long Intergenic Non-Protein Coding RNA 1828 | RNA Gene | 12 | GC02P067056 | 0.563586891 | https://www.genecards.org/cgi-bin/carddisp.pl?gene=LINC01828 |
| LINC01517 | Long Intergenic Non-Protein Coding RNA 1517 | RNA Gene | 12 | GC10P028744 | 0.563586891 | https://www.genecards.org/cgi-bin/carddisp.pl?gene=LINC01517 |
| LINC01484 | Long Intergenic Non-Protein Coding RNA 1484 | RNA Gene | 12 | GC05M173707 | 0.563586891 | https://www.genecards.org/cgi-bin/carddisp.pl?gene=LINC01484 |
| MICD | MHC Class I Polypeptide-Related Sequence D (Pseudogene) | Pseudogene | 11 | GC06M046772 | 0.563586891 | https://www.genecards.org/cgi-bin/carddisp.pl?gene=MICD |
| LINC02156 | Long Intergenic Non-Protein Coding RNA 2156 | RNA Gene | 11 | GC12P047416 | 0.563586891 | https://www.genecards.org/cgi-bin/carddisp.pl?gene=LINC02156 |
| DDR1-DT | DDR1 Divergent Transcript | RNA Gene | 11 | GC06M046807 | 0.563586891 | https://www.genecards.org/cgi-bin/carddisp.pl?gene=DDR1-DT |
| WSPAR | WNT Signaling Pathway Activating Non-Coding RNA | RNA Gene | 11 | GC05P133914 | 0.563586891 | https://www.genecards.org/cgi-bin/carddisp.pl?gene=WSPAR |
| LOC100506869 | Uncharacterized LOC100506869 | RNA Gene | 11 | GC12P058591 | 0.563586891 | https://www.genecards.org/cgi-bin/carddisp.pl?gene=LOC100506869 |
| LINC01829 | Long Intergenic Non-Protein Coding RNA 1829 | RNA Gene | 11 | GC02M067124 | 0.563586891 | https://www.genecards.org/cgi-bin/carddisp.pl?gene=LINC01829 |
| LINC01440 | Long Intergenic Non-Protein Coding RNA 1440 | RNA Gene | 11 | GC20P055302 | 0.563586891 | https://www.genecards.org/cgi-bin/carddisp.pl?gene=LINC01440 |
| LINC01360 | Long Intergenic Non-Protein Coding RNA 1360 | RNA Gene | 11 | GC01P073306 | 0.563586891 | https://www.genecards.org/cgi-bin/carddisp.pl?gene=LINC01360 |
| LINC01931 | Long Intergenic Non-Protein Coding RNA 1931 | RNA Gene | 10 | GC02M149769 | 0.563586891 | https://www.genecards.org/cgi-bin/carddisp.pl?gene=LINC01931 |
| MIR4527HG | MIR4527 Host Gene | RNA Gene | 10 | GC18P047264 | 0.563586891 | https://www.genecards.org/cgi-bin/carddisp.pl?gene=MIR4527HG |
| LINC02204 | Long Intergenic Non-Protein Coding RNA 2204 | RNA Gene | 10 | GC15M070131 | 0.563586891 | https://www.genecards.org/cgi-bin/carddisp.pl?gene=LINC02204 |
| HNRNPKP3 | Heterogeneous Nuclear Ribonucleoprotein K Pseudogene 3 | Pseudogene | 10 | GC11M043142 | 0.563586891 | https://www.genecards.org/cgi-bin/carddisp.pl?gene=HNRNPKP3 |
| ADAMTSL4-AS2 | ADAMTSL4 Antisense RNA 2 | RNA Gene | 10 | GC01M150550 | 0.563586891 | https://www.genecards.org/cgi-bin/carddisp.pl?gene=ADAMTSL4-AS2 |
| MMADHC-DT | MMADHC Divergent Transcript | RNA Gene | 10 | GC02P149590 | 0.563586891 | https://www.genecards.org/cgi-bin/carddisp.pl?gene=MMADHC-DT |
| ENSG00000264545 |  | Protein Coding | 10 | GC09P021802 | 0.563586891 | https://www.genecards.org/cgi-bin/carddisp.pl?gene=ENSG00000264545 |
| WDR87BP | WD Repeat Domain 87B, Pseudogene | RNA Gene | 10 | GC19M048349 | 0.563586891 | https://www.genecards.org/cgi-bin/carddisp.pl?gene=WDR87BP |
| LOC102724719 | Uncharacterized LOC102724719 | RNA Gene | 10 | GC10M051062 | 0.563586891 | https://www.genecards.org/cgi-bin/carddisp.pl?gene=LOC102724719 |
| OXCT2P1 | 3-Oxoacid CoA-Transferase 2 Pseudogene 1 | Pseudogene | 10 | GC01P039514 | 0.563586891 | https://www.genecards.org/cgi-bin/carddisp.pl?gene=OXCT2P1 |
| RPL21P124 | Ribosomal Protein L21 Pseudogene 124 | Pseudogene | 10 | GC17M049400 | 0.563586891 | https://www.genecards.org/cgi-bin/carddisp.pl?gene=RPL21P124 |
| NGF-AS1 | NGF Antisense RNA 1 | RNA Gene | 10 | GC01P115280 | 0.563586891 | https://www.genecards.org/cgi-bin/carddisp.pl?gene=NGF-AS1 |
| ATF7-NPFF | ATF7-NPFF Readthrough | Protein Coding | 9 | GC12M053535 | 0.563586891 | https://www.genecards.org/cgi-bin/carddisp.pl?gene=ATF7-NPFF |
| LINC01985 | Long Intergenic Non-Protein Coding RNA 1985 | RNA Gene | 9 | GC03P030519 | 0.563586891 | https://www.genecards.org/cgi-bin/carddisp.pl?gene=LINC01985 |
| HLA-W | Major Histocompatibility Complex, Class I, W (Pseudogene) | Pseudogene | 9 | GC06P055155 | 0.563586891 | https://www.genecards.org/cgi-bin/carddisp.pl?gene=HLA-W |
| LINC02388 | Long Intergenic Non-Protein Coding RNA 2388 | RNA Gene | 9 | GC12M058566 | 0.563586891 | https://www.genecards.org/cgi-bin/carddisp.pl?gene=LINC02388 |
| LINC02569 | Long Intergenic Non-Protein Coding RNA 2569 | RNA Gene | 9 | GC06P056137 | 0.563586891 | https://www.genecards.org/cgi-bin/carddisp.pl?gene=LINC02569 |
| FHAD1-AS1 | FHAD1 Antisense RNA 1 | RNA Gene | 9 | GC01M015328 | 0.563586891 | https://www.genecards.org/cgi-bin/carddisp.pl?gene=FHAD1-AS1 |
| ETF1P1 | Eukaryotic Translation Termination Factor 1 Pseudogene 1 | Pseudogene | 9 | GC06P055165 | 0.563586891 | https://www.genecards.org/cgi-bin/carddisp.pl?gene=ETF1P1 |
| CYP2T1P | Cytochrome P450 Family 2 Subfamily T Member 1, Pseudogene | Pseudogene | 9 | GC19M046778 | 0.563586891 | https://www.genecards.org/cgi-bin/carddisp.pl?gene=CYP2T1P |
| TSBP1-AS1 | TSBP1 And BTNL2 Antisense RNA 1 | RNA Gene | 9 | GC06P058058 | 0.563586891 | https://www.genecards.org/cgi-bin/carddisp.pl?gene=TSBP1-AS1 |
| ENSG00000258081 |  | RNA Gene | 9 | GC14P026604 | 0.563586891 | https://www.genecards.org/cgi-bin/carddisp.pl?gene=ENSG00000258081 |
| ZCCHC14-DT | ZCCHC14 Divergent Transcript | RNA Gene | 9 | GC16P087496 | 0.563586891 | https://www.genecards.org/cgi-bin/carddisp.pl?gene=ZCCHC14-DT |
| LINC01752 | Long Intergenic Non-Protein Coding RNA 1752 | RNA Gene | 9 | GC20P010753 | 0.563586891 | https://www.genecards.org/cgi-bin/carddisp.pl?gene=LINC01752 |
| RPF2P1 | Ribosome Production Factor 2 Homolog Pseudogene 1 | Pseudogene | 9 | GC20P035752 | 0.563586891 | https://www.genecards.org/cgi-bin/carddisp.pl?gene=RPF2P1 |
| PABPC4-AS1 | PABPC4 Antisense RNA 1 | RNA Gene | 9 | GC01P039566 | 0.563586891 | https://www.genecards.org/cgi-bin/carddisp.pl?gene=PABPC4-AS1 |
| LINC02163 | Long Intergenic Non-Protein Coding RNA 2163 | RNA Gene | 8 | GC05P104080 | 0.563586891 | https://www.genecards.org/cgi-bin/carddisp.pl?gene=LINC02163 |
| HLA-U | Major Histocompatibility Complex, Class I, U (Pseudogene) | Pseudogene | 8 | GC06P055160 | 0.563586891 | https://www.genecards.org/cgi-bin/carddisp.pl?gene=HLA-U |
| HNRNPA1P16 | Heterogeneous Nuclear Ribonucleoprotein A1 Pseudogene 16 | Pseudogene | 8 | GC17P002306 | 0.563586891 | https://www.genecards.org/cgi-bin/carddisp.pl?gene=HNRNPA1P16 |
| MIR4272 | MicroRNA 4272 | RNA Gene | 8 | GC03P067225 | 0.563586891 | https://www.genecards.org/cgi-bin/carddisp.pl?gene=MIR4272 |
| LINC02109 | Long Intergenic Non-Protein Coding RNA 2109 | RNA Gene | 8 | GC05P028820 | 0.563586891 | https://www.genecards.org/cgi-bin/carddisp.pl?gene=LINC02109 |
| LINC02205 | Long Intergenic Non-Protein Coding RNA 2205 | RNA Gene | 8 | GC15M070505 | 0.563586891 | https://www.genecards.org/cgi-bin/carddisp.pl?gene=LINC02205 |
| LINC02416 | Long Intergenic Non-Protein Coding RNA 2416 | RNA Gene | 8 | GC12P047354 | 0.563586891 | https://www.genecards.org/cgi-bin/carddisp.pl?gene=LINC02416 |
| LINC02641 | Long Intergenic Non-Protein Coding RNA 2641 | RNA Gene | 8 | GC10P123253 | 0.563586891 | https://www.genecards.org/cgi-bin/carddisp.pl?gene=LINC02641 |
| ERVK9-11 | Endogenous Retrovirus Group K9, Member 11 | Uncategorized | 8 | GC19M046735 | 0.563586891 | https://www.genecards.org/cgi-bin/carddisp.pl?gene=ERVK9-11 |
| ASLP1 | Argininosuccinate Lyase Pseudogene 1 | Pseudogene | 8 | GC22M023648 | 0.563586891 | https://www.genecards.org/cgi-bin/carddisp.pl?gene=ASLP1 |
| LOC100128310 | Uncharacterized LOC100128310 | RNA Gene | 8 | GC20M061718 | 0.563586891 | https://www.genecards.org/cgi-bin/carddisp.pl?gene=LOC100128310 |
| RNU11-3P | RNA, U11 Small Nuclear 3, Pseudogene | Pseudogene | 8 | GC10M103312 | 0.563586891 | https://www.genecards.org/cgi-bin/carddisp.pl?gene=RNU11-3P |
| RNA5SP481 | RNA, 5S Ribosomal Pseudogene 481 | Pseudogene | 8 | GC20P032009 | 0.563586891 | https://www.genecards.org/cgi-bin/carddisp.pl?gene=RNA5SP481 |
| ENSG00000254987 |  | RNA Gene | 8 | GC11M103546 | 0.563586891 | https://www.genecards.org/cgi-bin/carddisp.pl?gene=ENSG00000254987 |
| ENSG00000261783 |  | RNA Gene | 8 | GC16M075379 | 0.563586891 | https://www.genecards.org/cgi-bin/carddisp.pl?gene=ENSG00000261783 |
| ENSG00000255730 |  | Protein Coding | 8 | GC19P041350 | 0.563586891 | https://www.genecards.org/cgi-bin/carddisp.pl?gene=ENSG00000255730 |
| ENSG00000263345 |  | RNA Gene | 8 | GC17M002376 | 0.563586891 | https://www.genecards.org/cgi-bin/carddisp.pl?gene=ENSG00000263345 |
| ENSG00000260460 |  | RNA Gene | 8 | GC01P156509 | 0.563586891 | https://www.genecards.org/cgi-bin/carddisp.pl?gene=ENSG00000260460 |
| ENSG00000269843 |  | RNA Gene | 8 | GC19M040831 | 0.563586891 | https://www.genecards.org/cgi-bin/carddisp.pl?gene=ENSG00000269843 |
| ENSG00000250310 |  | RNA Gene | 8 | GC17M049544 | 0.563586891 | https://www.genecards.org/cgi-bin/carddisp.pl?gene=ENSG00000250310 |
| ENSG00000254532 |  | RNA Gene | 8 | GC11M030028 | 0.563586891 | https://www.genecards.org/cgi-bin/carddisp.pl?gene=ENSG00000254532 |
| ENSG00000270792 |  | RNA Gene | 8 | GC20P010672 | 0.563586891 | https://www.genecards.org/cgi-bin/carddisp.pl?gene=ENSG00000270792 |
| ENSG00000272463 |  | RNA Gene | 8 | GC06M000708 | 0.563586891 | https://www.genecards.org/cgi-bin/carddisp.pl?gene=ENSG00000272463 |
| ENSG00000199566 |  | RNA Gene | 8 | GC12M047346 | 0.563586891 | https://www.genecards.org/cgi-bin/carddisp.pl?gene=ENSG00000199566 |
| ENSG00000211683 |  | RNA Gene | 8 | GC22M023687 | 0.563586891 | https://www.genecards.org/cgi-bin/carddisp.pl?gene=ENSG00000211683 |
| ENSG00000234238 |  | RNA Gene | 8 | GC03P188107 | 0.563586891 | https://www.genecards.org/cgi-bin/carddisp.pl?gene=ENSG00000234238 |
| ENSG00000234692 |  | RNA Gene | 8 | GC09M115252 | 0.563586891 | https://www.genecards.org/cgi-bin/carddisp.pl?gene=ENSG00000234692 |
| ENSG00000236457 |  | RNA Gene | 8 | GC17M002215 | 0.563586891 | https://www.genecards.org/cgi-bin/carddisp.pl?gene=ENSG00000236457 |
| ENSG00000224277 |  | RNA Gene | 8 | GC22P023567 | 0.563586891 | https://www.genecards.org/cgi-bin/carddisp.pl?gene=ENSG00000224277 |
| ENSG00000230894 |  | RNA Gene | 8 | GC09P116611 | 0.563586891 | https://www.genecards.org/cgi-bin/carddisp.pl?gene=ENSG00000230894 |
| ENSG00000226849 |  | RNA Gene | 8 | GC01P011068 | 0.563586891 | https://www.genecards.org/cgi-bin/carddisp.pl?gene=ENSG00000226849 |
| ENSG00000227914 |  | RNA Gene | 8 | GC09M000673 | 0.563586891 | https://www.genecards.org/cgi-bin/carddisp.pl?gene=ENSG00000227914 |
| ENSG00000236838 |  | RNA Gene | 8 | GC17P002175 | 0.563586891 | https://www.genecards.org/cgi-bin/carddisp.pl?gene=ENSG00000236838 |
| ENSG00000237742 |  | RNA Gene | 8 | GC06M125578 | 0.563586891 | https://www.genecards.org/cgi-bin/carddisp.pl?gene=ENSG00000237742 |
| SMARCAL1-AS1 | SMARCAL1 Antisense RNA 1 | RNA Gene | 8 | GC02M216387 | 0.563586891 | https://www.genecards.org/cgi-bin/carddisp.pl?gene=SMARCAL1-AS1 |
| SUMO2P1 | SUMO2 Pseudogene 1 | Pseudogene | 8 | GC06M046744 | 0.563586891 | https://www.genecards.org/cgi-bin/carddisp.pl?gene=SUMO2P1 |
| PAIP1P1 | Poly(A) Binding Protein Interacting Protein 1 Pseudogene 1 | Pseudogene | 8 | GC06M030186 | 0.563586891 | https://www.genecards.org/cgi-bin/carddisp.pl?gene=PAIP1P1 |
| RPL3P2 | Ribosomal Protein L3 Pseudogene 2 | Pseudogene | 8 | GC06P031280 | 0.563586891 | https://www.genecards.org/cgi-bin/carddisp.pl?gene=RPL3P2 |
| RPL12P16 | Ribosomal Protein L12 Pseudogene 16 | Pseudogene | 8 | GC02P203193 | 0.563586891 | https://www.genecards.org/cgi-bin/carddisp.pl?gene=RPL12P16 |
| RPS3AP34 | RPS3A Pseudogene 34 | Pseudogene | 8 | GC08M012570 | 0.563586891 | https://www.genecards.org/cgi-bin/carddisp.pl?gene=RPS3AP34 |
| ENSG00000248127 |  | RNA Gene | 8 | GC05M076081 | 0.563586891 | https://www.genecards.org/cgi-bin/carddisp.pl?gene=ENSG00000248127 |
| LINC02730 | Long Intergenic Non-Protein Coding RNA 2730 | RNA Gene | 7 | GC11P134027 | 0.563586891 | https://www.genecards.org/cgi-bin/carddisp.pl?gene=LINC02730 |
| LINC02238 | Long Intergenic Non-Protein Coding RNA 2238 | RNA Gene | 7 | GC01P073636 | 0.563586891 | https://www.genecards.org/cgi-bin/carddisp.pl?gene=LINC02238 |
| LINC02644 | Long Intergenic Non-Protein Coding RNA 2644 | RNA Gene | 7 | GC10P030727 | 0.563586891 | https://www.genecards.org/cgi-bin/carddisp.pl?gene=LINC02644 |
| LOC101928775 | Uncharacterized LOC101928775 | RNA Gene | 7 | GC09M115640 | 0.563586891 | https://www.genecards.org/cgi-bin/carddisp.pl?gene=LOC101928775 |
| ENSG00000248172 |  | RNA Gene | 7 | GC17P049996 | 0.563586891 | https://www.genecards.org/cgi-bin/carddisp.pl?gene=ENSG00000248172 |
| ENSG00000250186 |  | RNA Gene | 7 | GC17P049404 | 0.563586891 | https://www.genecards.org/cgi-bin/carddisp.pl?gene=ENSG00000250186 |
| ENSG00000251867 |  | RNA Gene | 7 | GC08M080387 | 0.563586891 | https://www.genecards.org/cgi-bin/carddisp.pl?gene=ENSG00000251867 |
| ENSG00000253553 |  | RNA Gene | 7 | GC08P088326 | 0.563586891 | https://www.genecards.org/cgi-bin/carddisp.pl?gene=ENSG00000253553 |
| ENSG00000267152 |  | RNA Gene | 7 | GC19M037728 | 0.563586891 | https://www.genecards.org/cgi-bin/carddisp.pl?gene=ENSG00000267152 |
| ENSG00000267504 |  | RNA Gene | 7 | GC18P058371 | 0.563586891 | https://www.genecards.org/cgi-bin/carddisp.pl?gene=ENSG00000267504 |
| ENSG00000267892 |  | RNA Gene | 7 | GC19P040455 | 0.563586891 | https://www.genecards.org/cgi-bin/carddisp.pl?gene=ENSG00000267892 |
| ENSG00000254422 |  | RNA Gene | 7 | GC11M102229 | 0.563586891 | https://www.genecards.org/cgi-bin/carddisp.pl?gene=ENSG00000254422 |
| ENSG00000269688 |  | RNA Gene | 7 | GC19M046733 | 0.563586891 | https://www.genecards.org/cgi-bin/carddisp.pl?gene=ENSG00000269688 |
| ENSG00000234091 |  | RNA Gene | 7 | GC10P013729 | 0.563586891 | https://www.genecards.org/cgi-bin/carddisp.pl?gene=ENSG00000234091 |
| ENSG00000236045 |  | RNA Gene | 7 | GC01M015334 | 0.563586891 | https://www.genecards.org/cgi-bin/carddisp.pl?gene=ENSG00000236045 |
| LOC107986898 | Uncharacterized LOC107986898 | RNA Gene | 7 | GC08P102529 | 0.563586891 | https://www.genecards.org/cgi-bin/carddisp.pl?gene=LOC107986898 |
| ENSG00000244757 |  | RNA Gene | 7 | GC09M116288 | 0.563586891 | https://www.genecards.org/cgi-bin/carddisp.pl?gene=ENSG00000244757 |
| RPSAP11 | Ribosomal Protein SA Pseudogene 11 | Pseudogene | 7 | GC03P032252 | 0.563586891 | https://www.genecards.org/cgi-bin/carddisp.pl?gene=RPSAP11 |
| RNU6-850P | RNA, U6 Small Nuclear 850, Pseudogene | Pseudogene | 7 | GC06M031756 | 0.563586891 | https://www.genecards.org/cgi-bin/carddisp.pl?gene=RNU6-850P |
| RNU6-908P | RNA, U6 Small Nuclear 908, Pseudogene | Pseudogene | 7 | GC10P029711 | 0.563586891 | https://www.genecards.org/cgi-bin/carddisp.pl?gene=RNU6-908P |
| RNU6-1224P | RNA, U6 Small Nuclear 1224, Pseudogene | Pseudogene | 7 | GC08M102533 | 0.563586891 | https://www.genecards.org/cgi-bin/carddisp.pl?gene=RNU6-1224P |
| GAPDHP24 | Glyceraldehyde 3 Phosphate Dehydrogenase Pseudogene 24 | Pseudogene | 6 | GC01P214870 | 0.563586891 | https://www.genecards.org/cgi-bin/carddisp.pl?gene=GAPDHP24 |
| LINC02646 | Long Intergenic Non-Protein Coding RNA 2646 | RNA Gene | 6 | GC10P130215 | 0.563586891 | https://www.genecards.org/cgi-bin/carddisp.pl?gene=LINC02646 |
| LINC02782 | Long Intergenic Non-Protein Coding RNA 2782 | RNA Gene | 6 | GC01M005088 | 0.563586891 | https://www.genecards.org/cgi-bin/carddisp.pl?gene=LINC02782 |
| MTND4P19 | MT-ND4 Pseudogene 19 | Pseudogene | 6 | GC10M094763 | 0.563586891 | https://www.genecards.org/cgi-bin/carddisp.pl?gene=MTND4P19 |
| RNA5SP299 | RNA, 5S Ribosomal Pseudogene 299 | Pseudogene | 6 | GC10M008699 | 0.563586891 | https://www.genecards.org/cgi-bin/carddisp.pl?gene=RNA5SP299 |
| RNA5SP482 | RNA, 5S Ribosomal Pseudogene 482 | Pseudogene | 6 | GC20P032050 | 0.563586891 | https://www.genecards.org/cgi-bin/carddisp.pl?gene=RNA5SP482 |
| LOC107984270 | Uncharacterized LOC107984270 | RNA Gene | 6 | GC10M113444 | 0.563586891 | https://www.genecards.org/cgi-bin/carddisp.pl?gene=LOC107984270 |
| ENSG00000252122 |  | RNA Gene | 6 | GC16M075410 | 0.563586891 | https://www.genecards.org/cgi-bin/carddisp.pl?gene=ENSG00000252122 |
| ENSG00000255122 |  | Pseudogene | 6 | GC08P012581 | 0.563586891 | https://www.genecards.org/cgi-bin/carddisp.pl?gene=ENSG00000255122 |
| ENSG00000259066 |  | Protein Coding | 6 | GC14P093184 | 0.563586891 | https://www.genecards.org/cgi-bin/carddisp.pl?gene=ENSG00000259066 |
| ENSG00000259503 |  | RNA Gene | 6 | GC15P070321 | 0.563586891 | https://www.genecards.org/cgi-bin/carddisp.pl?gene=ENSG00000259503 |
| ENSG00000259560 |  | RNA Gene | 6 | GC15M087305 | 0.563586891 | https://www.genecards.org/cgi-bin/carddisp.pl?gene=ENSG00000259560 |
| ENSG00000272733 |  | RNA Gene | 6 | GC22M023580 | 0.563586891 | https://www.genecards.org/cgi-bin/carddisp.pl?gene=ENSG00000272733 |
| ENSG00000275443 |  | RNA Gene | 6 | GC15P095990 | 0.563586891 | https://www.genecards.org/cgi-bin/carddisp.pl?gene=ENSG00000275443 |
| ENSG00000253238 |  | RNA Gene | 6 | GC08P080265 | 0.563586891 | https://www.genecards.org/cgi-bin/carddisp.pl?gene=ENSG00000253238 |
| ENSG00000253891 |  | RNA Gene | 6 | GC08M024169 | 0.563586891 | https://www.genecards.org/cgi-bin/carddisp.pl?gene=ENSG00000253891 |
| ENSG00000261572 |  | RNA Gene | 6 | GC03M032236 | 0.563586891 | https://www.genecards.org/cgi-bin/carddisp.pl?gene=ENSG00000261572 |
| ENSG00000272235 |  | RNA Gene | 6 | GC01M003306 | 0.563586891 | https://www.genecards.org/cgi-bin/carddisp.pl?gene=ENSG00000272235 |
| ENSG00000283128 |  | RNA Gene | 6 | GC07M155475 | 0.563586891 | https://www.genecards.org/cgi-bin/carddisp.pl?gene=ENSG00000283128 |
| ENSG00000250025 |  | RNA Gene | 6 | GC05M146307 | 0.563586891 | https://www.genecards.org/cgi-bin/carddisp.pl?gene=ENSG00000250025 |
| ENSG00000254092 |  | RNA Gene | 6 | GC08M020952 | 0.563586891 | https://www.genecards.org/cgi-bin/carddisp.pl?gene=ENSG00000254092 |
| ENSG00000267375 |  | RNA Gene | 6 | GC19M038683 | 0.563586891 | https://www.genecards.org/cgi-bin/carddisp.pl?gene=ENSG00000267375 |
| ENSG00000270207 |  | RNA Gene | 6 | GC03M007649 | 0.563586891 | https://www.genecards.org/cgi-bin/carddisp.pl?gene=ENSG00000270207 |
| ENSG00000285708 |  | Protein Coding | 6 | GC03M070959 | 0.563586891 | https://www.genecards.org/cgi-bin/carddisp.pl?gene=ENSG00000285708 |
| ENSG00000249429 |  | RNA Gene | 6 | GC05M143489 | 0.563586891 | https://www.genecards.org/cgi-bin/carddisp.pl?gene=ENSG00000249429 |
| ENSG00000272657 |  | RNA Gene | 6 | GC21P034073 | 0.563586891 | https://www.genecards.org/cgi-bin/carddisp.pl?gene=ENSG00000272657 |
| ENSG00000225058 |  | RNA Gene | 6 | GC03M187997 | 0.563586891 | https://www.genecards.org/cgi-bin/carddisp.pl?gene=ENSG00000225058 |
| ENSG00000228683 |  | RNA Gene | 6 | GC10M078696 | 0.563586891 | https://www.genecards.org/cgi-bin/carddisp.pl?gene=ENSG00000228683 |
| ENSG00000238258 |  | RNA Gene | 6 | GC10P033211 | 0.563586891 | https://www.genecards.org/cgi-bin/carddisp.pl?gene=ENSG00000238258 |
| ENSG00000206651 |  | RNA Gene | 6 | GC01M156485 | 0.563586891 | https://www.genecards.org/cgi-bin/carddisp.pl?gene=ENSG00000206651 |
| ENSG00000207127 |  | RNA Gene | 6 | GC17M049416 | 0.563586891 | https://www.genecards.org/cgi-bin/carddisp.pl?gene=ENSG00000207127 |
| ENSG00000207303 |  | RNA Gene | 6 | GC02P216351 | 0.563586891 | https://www.genecards.org/cgi-bin/carddisp.pl?gene=ENSG00000207303 |
| ENSG00000214955 |  | RNA Gene | 6 | GC21P034205 | 0.563586891 | https://www.genecards.org/cgi-bin/carddisp.pl?gene=ENSG00000214955 |
| ENSG00000237477 |  | RNA Gene | 6 | GC02P179273 | 0.563586891 | https://www.genecards.org/cgi-bin/carddisp.pl?gene=ENSG00000237477 |
| ENSG00000199332 |  | RNA Gene | 6 | GC06M046848 | 0.563586891 | https://www.genecards.org/cgi-bin/carddisp.pl?gene=ENSG00000199332 |
| ENSG00000225339 |  | RNA Gene | 6 | GC06P055310 | 0.563586891 | https://www.genecards.org/cgi-bin/carddisp.pl?gene=ENSG00000225339 |
| ENSG00000228714 |  | Uncategorized | 6 | GC09M115325 | 0.563586891 | https://www.genecards.org/cgi-bin/carddisp.pl?gene=ENSG00000228714 |
| ENSG00000231243 |  | RNA Gene | 6 | GC03P038865 | 0.563586891 | https://www.genecards.org/cgi-bin/carddisp.pl?gene=ENSG00000231243 |
| ENSG00000233581 |  | RNA Gene | 6 | GC02P216303 | 0.563586891 | https://www.genecards.org/cgi-bin/carddisp.pl?gene=ENSG00000233581 |
| ENSG00000237530 |  | RNA Gene | 6 | GC06P055422 | 0.563586891 | https://www.genecards.org/cgi-bin/carddisp.pl?gene=ENSG00000237530 |
| POLD2P1 | DNA Polymerase Delta 2, Accessory Subunit Pseudogene 1 | Pseudogene | 6 | GC05P093267 | 0.563586891 | https://www.genecards.org/cgi-bin/carddisp.pl?gene=POLD2P1 |
| SMU1P1 | SMU1 Pseudogene 1 | Pseudogene | 6 | GC01M157065 | 0.563586891 | https://www.genecards.org/cgi-bin/carddisp.pl?gene=SMU1P1 |
| RNU7-165P | RNA, U7 Small Nuclear 165 Pseudogene | Pseudogene | 6 | GC10P113353 | 0.563586891 | https://www.genecards.org/cgi-bin/carddisp.pl?gene=RNU7-165P |
| RPL5P26 | Ribosomal Protein L5 Pseudogene 26 | Pseudogene | 6 | GC10P069778 | 0.563586891 | https://www.genecards.org/cgi-bin/carddisp.pl?gene=RPL5P26 |
| RNU6ATAC8P | RNA, U6atac Small Nuclear 8, Pseudogene | Pseudogene | 6 | GC08P102421 | 0.563586891 | https://www.genecards.org/cgi-bin/carddisp.pl?gene=RNU6ATAC8P |
| RPL7AP33 | Ribosomal Protein L7a Pseudogene 33 | Pseudogene | 6 | GC05M173225 | 0.563586891 | https://www.genecards.org/cgi-bin/carddisp.pl?gene=RPL7AP33 |
| RPS4XP18 | Ribosomal Protein S4X Pseudogene 18 | Pseudogene | 6 | GC18P022586 | 0.563586891 | https://www.genecards.org/cgi-bin/carddisp.pl?gene=RPS4XP18 |
| RNU6-1080P | RNA, U6 Small Nuclear 1080, Pseudogene | Pseudogene | 6 | GC07M073339 | 0.563586891 | https://www.genecards.org/cgi-bin/carddisp.pl?gene=RNU6-1080P |
| SNRPEP6 | SNRPE Pseudogene 6 | Pseudogene | 6 | GC06M148567 | 0.563586891 | https://www.genecards.org/cgi-bin/carddisp.pl?gene=SNRPEP6 |
| KLF9-DT | KLF9 Divergent Transcript | RNA Gene | 5 | GC09P070414 | 0.563586891 | https://www.genecards.org/cgi-bin/carddisp.pl?gene=KLF9-DT |
| HNRNPA3P8 | Heterogeneous Nuclear Ribonucleoprotein A3 Pseudogene 8 | Pseudogene | 5 | GC03P080266 | 0.563586891 | https://www.genecards.org/cgi-bin/carddisp.pl?gene=HNRNPA3P8 |
| KATNBL1P4 | Katanin Regulatory Subunit B1 Like 1 Pseudogene 4 | Pseudogene | 5 | GC05P051930 | 0.563586891 | https://www.genecards.org/cgi-bin/carddisp.pl?gene=KATNBL1P4 |
| HMGB3P19 | High Mobility Group Box 3 Pseudogene 19 | Pseudogene | 5 | GC06M153938 | 0.563586891 | https://www.genecards.org/cgi-bin/carddisp.pl?gene=HMGB3P19 |
| EEF1DP6 | Eukaryotic Translation Elongation Factor 1 Delta Pseudogene 6 | Pseudogene | 5 | GC01M004175 | 0.563586891 | https://www.genecards.org/cgi-bin/carddisp.pl?gene=EEF1DP6 |
| LOC105372704 | Uncharacterized LOC105372704 | Protein Coding | 5 | GC20M062000 | 0.563586891 | https://www.genecards.org/cgi-bin/carddisp.pl?gene=LOC105372704 |
| MTND2P9 | MT-ND2 Pseudogene 9 | Pseudogene | 5 | GC09M080565 | 0.563586891 | https://www.genecards.org/cgi-bin/carddisp.pl?gene=MTND2P9 |
| ENSG00000273771 |  | RNA Gene | 5 | GC15M096080 | 0.563586891 | https://www.genecards.org/cgi-bin/carddisp.pl?gene=ENSG00000273771 |
| ENSG00000285692 |  | RNA Gene | 5 | GC12P053524 | 0.563586891 | https://www.genecards.org/cgi-bin/carddisp.pl?gene=ENSG00000285692 |
| ENSG00000285955 |  | RNA Gene | 5 | GC10M122435 | 0.563586891 | https://www.genecards.org/cgi-bin/carddisp.pl?gene=ENSG00000285955 |
| ENSG00000286509 |  | RNA Gene | 5 | GC05P093088 | 0.563586891 | https://www.genecards.org/cgi-bin/carddisp.pl?gene=ENSG00000286509 |
| ENSG00000287277 |  | RNA Gene | 5 | GC10M006824 | 0.563586891 | https://www.genecards.org/cgi-bin/carddisp.pl?gene=ENSG00000287277 |
| ENSG00000271267 |  | Pseudogene | 5 | GC01M155568 | 0.563586891 | https://www.genecards.org/cgi-bin/carddisp.pl?gene=ENSG00000271267 |
| ENSG00000248701 |  | RNA Gene | 5 | GC05P086797 | 0.563586891 | https://www.genecards.org/cgi-bin/carddisp.pl?gene=ENSG00000248701 |
| ENSG00000260640 |  | RNA Gene | 5 | GC08M097241 | 0.563586891 | https://www.genecards.org/cgi-bin/carddisp.pl?gene=ENSG00000260640 |
| ENSG00000267640 |  | Pseudogene | 5 | GC19P040422 | 0.563586891 | https://www.genecards.org/cgi-bin/carddisp.pl?gene=ENSG00000267640 |
| ENSG00000276490 |  | Protein Coding | 5 | GC10P094688 | 0.563586891 | https://www.genecards.org/cgi-bin/carddisp.pl?gene=ENSG00000276490 |
| ENSG00000202035 |  | RNA Gene | 5 | GC07M073403 | 0.563586891 | https://www.genecards.org/cgi-bin/carddisp.pl?gene=ENSG00000202035 |
| ENSG00000231601 |  | RNA Gene | 5 | GC10P000743 | 0.563586891 | https://www.genecards.org/cgi-bin/carddisp.pl?gene=ENSG00000231601 |
| ENSG00000236948 |  | RNA Gene | 5 | GC01M005559 | 0.563586891 | https://www.genecards.org/cgi-bin/carddisp.pl?gene=ENSG00000236948 |
| ENSG00000204584 |  | Uncategorized | 5 | GC17P049846 | 0.563586891 | https://www.genecards.org/cgi-bin/carddisp.pl?gene=ENSG00000204584 |
| LOC107987042 | Phosphatidylinositol 4,5-Bisphosphate 5-Phosphatase A-Like | Protein Coding | 5 | GC09M000706 | 0.563586891 | https://www.genecards.org/cgi-bin/carddisp.pl?gene=LOC107987042 |
| lnc-DEC1-10 |  | RNA Gene | 5 | GC09P115473 | 0.563586891 | https://www.genecards.org/cgi-bin/carddisp.pl?gene=lnc-DEC1-10 |
| lnc-DPH1-2 |  | RNA Gene | 5 | GC17P002055 | 0.563586891 | https://www.genecards.org/cgi-bin/carddisp.pl?gene=lnc-DPH1-2 |
| lnc-GPR26-9 |  | RNA Gene | 5 | GC10P123346 | 0.563586891 | https://www.genecards.org/cgi-bin/carddisp.pl?gene=lnc-GPR26-9 |
| lnc-PEX14-7 |  | RNA Gene | 5 | GC01P010698 | 0.563586891 | https://www.genecards.org/cgi-bin/carddisp.pl?gene=lnc-PEX14-7 |
| POU5F1P2 | POU Class 5 Homeobox 1 Pseudogene 2 | Pseudogene | 5 | GC08M102621 | 0.563586891 | https://www.genecards.org/cgi-bin/carddisp.pl?gene=POU5F1P2 |
| RNU6-896P | RNA, U6 Small Nuclear 896, Pseudogene | Pseudogene | 5 | GC06P153681 | 0.563586891 | https://www.genecards.org/cgi-bin/carddisp.pl?gene=RNU6-896P |
| RPL36AP26 | Ribosomal Protein L36a Pseudogene 26 | Pseudogene | 5 | GC07M015702 | 0.563586891 | https://www.genecards.org/cgi-bin/carddisp.pl?gene=RPL36AP26 |
| RPSAP40 | Ribosomal Protein SA Pseudogene 40 | Pseudogene | 5 | GC06M148647 | 0.563586891 | https://www.genecards.org/cgi-bin/carddisp.pl?gene=RPSAP40 |
| RPS19P6 | Ribosomal Protein S19 Pseudogene 6 | Pseudogene | 5 | GC09M080782 | 0.563586891 | https://www.genecards.org/cgi-bin/carddisp.pl?gene=RPS19P6 |
| RPS7P8 | Ribosomal Protein S7 Pseudogene 8 | Pseudogene | 5 | GC06M096648 | 0.563586891 | https://www.genecards.org/cgi-bin/carddisp.pl?gene=RPS7P8 |
| RPS8P7 | Ribosomal Protein S8 Pseudogene 7 | Pseudogene | 5 | GC05M180226 | 0.563586891 | https://www.genecards.org/cgi-bin/carddisp.pl?gene=RPS8P7 |
| SDCBPP3 | Syndecan Binding Protein Pseudogene 3 | Pseudogene | 5 | GC0XM040890 | 0.563586891 | https://www.genecards.org/cgi-bin/carddisp.pl?gene=SDCBPP3 |
| RNU6-1198P | RNA, U6 Small Nuclear 1198, Pseudogene | Pseudogene | 5 | GC07P073507 | 0.563586891 | https://www.genecards.org/cgi-bin/carddisp.pl?gene=RNU6-1198P |
| SAP18P1 | SAP18 Pseudogene 1 | Pseudogene | 5 | GC05P076076 | 0.563586891 | https://www.genecards.org/cgi-bin/carddisp.pl?gene=SAP18P1 |
| ENSG00000287575 |  | RNA Gene | 5 | GC13M110132 | 0.563586891 | https://www.genecards.org/cgi-bin/carddisp.pl?gene=ENSG00000287575 |
| ENSG00000243861 |  | RNA Gene | 5 | GC03M154511 | 0.563586891 | https://www.genecards.org/cgi-bin/carddisp.pl?gene=ENSG00000243861 |
| ENSG00000246203 |  | Pseudogene | 5 | GC01M155614 | 0.563586891 | https://www.genecards.org/cgi-bin/carddisp.pl?gene=ENSG00000246203 |
| HSALNG0000794 |  | RNA Gene | 5 | GC01P010663 | 0.563586891 | https://www.genecards.org/cgi-bin/carddisp.pl?gene=HSALNG0000794 |
| HSALNG0113871 |  | RNA Gene | 5 | GC17M001995 | 0.563586891 | https://www.genecards.org/cgi-bin/carddisp.pl?gene=HSALNG0113871 |
| lnc-A1CF-8 |  | RNA Gene | 5 | GC10M050990 | 0.563586891 | https://www.genecards.org/cgi-bin/carddisp.pl?gene=lnc-A1CF-8 |
| lnc-INHBA-1 |  | RNA Gene | 5 | GC07M041669 | 0.563586891 | https://www.genecards.org/cgi-bin/carddisp.pl?gene=lnc-INHBA-1 |
| lnc-SMG6-6 |  | RNA Gene | 5 | GC17M002054 | 0.563586891 | https://www.genecards.org/cgi-bin/carddisp.pl?gene=lnc-SMG6-6 |
| lnc-NT5C2-1 |  | RNA Gene | 5 | GC10M103198 | 0.563586891 | https://www.genecards.org/cgi-bin/carddisp.pl?gene=lnc-NT5C2-1 |
| lnc-SMC5-10 |  | RNA Gene | 5 | GC09P070420 | 0.563586891 | https://www.genecards.org/cgi-bin/carddisp.pl?gene=lnc-SMC5-10 |
| lnc-JCAD-2 |  | RNA Gene | 5 | GC10M031184 | 0.563586891 | https://www.genecards.org/cgi-bin/carddisp.pl?gene=lnc-JCAD-2 |
| RF00017-1991 |  | RNA Gene | 5 | GC17P001825 | 0.563586891 | https://www.genecards.org/cgi-bin/carddisp.pl?gene=RF00017-1991 |
| LOC101060035 | DnaJ Homolog Subfamily C Member 24 Pseudogene | Pseudogene | 4 | GC05M133720 | 0.563586891 | https://www.genecards.org/cgi-bin/carddisp.pl?gene=LOC101060035 |
| LOC105369212 | Uncharacterized LOC105369212 | RNA Gene | 4 | GC15M107461 | 0.563586891 | https://www.genecards.org/cgi-bin/carddisp.pl?gene=LOC105369212 |
| ENSG00000286749 |  | RNA Gene | 4 | GC05P152374 | 0.563586891 | https://www.genecards.org/cgi-bin/carddisp.pl?gene=ENSG00000286749 |
| ENSG00000287003 |  | RNA Gene | 4 | GC05P173757 | 0.563586891 | https://www.genecards.org/cgi-bin/carddisp.pl?gene=ENSG00000287003 |
| ENSG00000272966 |  | RNA Gene | 4 | GC02P202339 | 0.563586891 | https://www.genecards.org/cgi-bin/carddisp.pl?gene=ENSG00000272966 |
| ENSG00000286376 |  | RNA Gene | 4 | GC07M015736 | 0.563586891 | https://www.genecards.org/cgi-bin/carddisp.pl?gene=ENSG00000286376 |
| ENSG00000287549 |  | RNA Gene | 4 | GC0XM041008 | 0.563586891 | https://www.genecards.org/cgi-bin/carddisp.pl?gene=ENSG00000287549 |
| ENSG00000280333 |  | Uncategorized | 4 | GC17P002072 | 0.563586891 | https://www.genecards.org/cgi-bin/carddisp.pl?gene=ENSG00000280333 |
| ENSG00000230495 |  | Pseudogene | 4 | GC20M017602 | 0.563586891 | https://www.genecards.org/cgi-bin/carddisp.pl?gene=ENSG00000230495 |
| ENSG00000230521 |  | Pseudogene | 4 | GC06M046763 | 0.563586891 | https://www.genecards.org/cgi-bin/carddisp.pl?gene=ENSG00000230521 |
| ENSG00000237669 |  | Pseudogene | 4 | GC06M047187 | 0.563586891 | https://www.genecards.org/cgi-bin/carddisp.pl?gene=ENSG00000237669 |
| ENSG00000237936 |  | Pseudogene | 4 | GC10M029891 | 0.563586891 | https://www.genecards.org/cgi-bin/carddisp.pl?gene=ENSG00000237936 |
| HSALNG0006267-002 |  | RNA Gene | 4 | GC01P115231 | 0.563586891 | https://www.genecards.org/cgi-bin/carddisp.pl?gene=HSALNG0006267-002 |
| TUBB4BP7 | TUBB4B Pseudogene 7 | Pseudogene | 4 | GC06P152897 | 0.563586891 | https://www.genecards.org/cgi-bin/carddisp.pl?gene=TUBB4BP7 |
| ZBTB2P1 | ZBTB2 Pseudogene 1 | Pseudogene | 4 | GC01P015228 | 0.563586891 | https://www.genecards.org/cgi-bin/carddisp.pl?gene=ZBTB2P1 |
| lnc-GATA6-4 |  | RNA Gene | 4 | GC18P022487 | 0.563586891 | https://www.genecards.org/cgi-bin/carddisp.pl?gene=lnc-GATA6-4 |
| lnc-SCAND1-4 |  | RNA Gene | 4 | GC20M035851 | 0.563586891 | https://www.genecards.org/cgi-bin/carddisp.pl?gene=lnc-SCAND1-4 |
| HSALNG0049197 |  | RNA Gene | 4 | GC06P055718 | 0.563586891 | https://www.genecards.org/cgi-bin/carddisp.pl?gene=HSALNG0049197 |
| PDK1P1 | Pyruvate Dehydrogenase Kinase 1 Pseudogene 1 | Pseudogene | 4 | GC09P029318 | 0.563586891 | https://www.genecards.org/cgi-bin/carddisp.pl?gene=PDK1P1 |
| ENSG00000287744 |  | RNA Gene | 4 | GC07M153715 | 0.563586891 | https://www.genecards.org/cgi-bin/carddisp.pl?gene=ENSG00000287744 |
| lnc-C10orf82-2 |  | RNA Gene | 4 | GC10M116272 | 0.563586891 | https://www.genecards.org/cgi-bin/carddisp.pl?gene=lnc-C10orf82-2 |
| HSALNG0000316 |  | RNA Gene | 4 | GC01M003308 | 0.563586891 | https://www.genecards.org/cgi-bin/carddisp.pl?gene=HSALNG0000316 |
| HSALNG0056290 |  | RNA Gene | 4 | GC07M013932 | 0.563586891 | https://www.genecards.org/cgi-bin/carddisp.pl?gene=HSALNG0056290 |
| HSALNG0076986 |  | RNA Gene | 4 | GC10M031161 | 0.563586891 | https://www.genecards.org/cgi-bin/carddisp.pl?gene=HSALNG0076986 |
| HSALNG0125928 |  | RNA Gene | 4 | GC19P040787 | 0.563586891 | https://www.genecards.org/cgi-bin/carddisp.pl?gene=HSALNG0125928 |
| piR-32214-582 |  | RNA Gene | 4 | GC06P056718 | 0.563586891 | https://www.genecards.org/cgi-bin/carddisp.pl?gene=piR-32214-582 |
| piR-50799 |  | RNA Gene | 4 | GC17P002057 | 0.563586891 | https://www.genecards.org/cgi-bin/carddisp.pl?gene=piR-50799 |
| HSALNG0120371 |  | RNA Gene | 4 | GC18P022486 | 0.563586891 | https://www.genecards.org/cgi-bin/carddisp.pl?gene=HSALNG0120371 |
| lnc-ADAMTSL4-1 |  | RNA Gene | 4 | GC01P150533 | 0.563586891 | https://www.genecards.org/cgi-bin/carddisp.pl?gene=lnc-ADAMTSL4-1 |
| MN296886 |  | RNA Gene | 4 | GC01M156505 | 0.563586891 | https://www.genecards.org/cgi-bin/carddisp.pl?gene=MN296886 |
| MN298114-127 |  | RNA Gene | 4 | GC21P034283 | 0.563586891 | https://www.genecards.org/cgi-bin/carddisp.pl?gene=MN298114-127 |
| NONHSAG051616.2 |  | RNA Gene | 4 | GC09P000684 | 0.563586891 | https://www.genecards.org/cgi-bin/carddisp.pl?gene=NONHSAG051616.2 |
| lnc-COL4A3BP-2 |  | RNA Gene | 4 | GC05M075675 | 0.563586891 | https://www.genecards.org/cgi-bin/carddisp.pl?gene=lnc-COL4A3BP-2 |
| lnc-KCNK5-2 |  | RNA Gene | 4 | GC06M047703 | 0.563586891 | https://www.genecards.org/cgi-bin/carddisp.pl?gene=lnc-KCNK5-2 |
| lnc-MEF2D-1 |  | RNA Gene | 4 | GC01M156504 | 0.563586891 | https://www.genecards.org/cgi-bin/carddisp.pl?gene=lnc-MEF2D-1 |
| lnc-SMC5-8 |  | RNA Gene | 4 | GC09P070529 | 0.563586891 | https://www.genecards.org/cgi-bin/carddisp.pl?gene=lnc-SMC5-8 |
| lnc-SMG6-5 |  | RNA Gene | 4 | GC17M002060 | 0.563586891 | https://www.genecards.org/cgi-bin/carddisp.pl?gene=lnc-SMG6-5 |
| RF00017-4625 |  | RNA Gene | 4 | GC05P172150 | 0.563586891 | https://www.genecards.org/cgi-bin/carddisp.pl?gene=RF00017-4625 |
| RF00017-4912 |  | RNA Gene | 4 | GC06M048623 | 0.563586891 | https://www.genecards.org/cgi-bin/carddisp.pl?gene=RF00017-4912 |
| RF00017-5023 |  | RNA Gene | 4 | GC06P057923 | 0.563586891 | https://www.genecards.org/cgi-bin/carddisp.pl?gene=RF00017-5023 |
| lnc-CYP2C8-5 |  | RNA Gene | 4 | GC10M094774 | 0.563586891 | https://www.genecards.org/cgi-bin/carddisp.pl?gene=lnc-CYP2C8-5 |
| lnc-DMRT1-3 |  | RNA Gene | 4 | GC09P000662 | 0.563586891 | https://www.genecards.org/cgi-bin/carddisp.pl?gene=lnc-DMRT1-3 |
| lnc-IPO5-3 |  | RNA Gene | 4 | GC13P098143 | 0.563586891 | https://www.genecards.org/cgi-bin/carddisp.pl?gene=lnc-IPO5-3 |
| lnc-MCL1-8 |  | RNA Gene | 4 | GC01M150535 | 0.563586891 | https://www.genecards.org/cgi-bin/carddisp.pl?gene=lnc-MCL1-8 |
| lnc-NT5C2-2 |  | RNA Gene | 4 | GC10M103188 | 0.563586891 | https://www.genecards.org/cgi-bin/carddisp.pl?gene=lnc-NT5C2-2 |
| lnc-PABPC4-1 |  | RNA Gene | 4 | GC01M039521 | 0.563586891 | https://www.genecards.org/cgi-bin/carddisp.pl?gene=lnc-PABPC4-1 |
| lnc-PDRG1-3 |  | RNA Gene | 4 | GC20M032033 | 0.563586891 | https://www.genecards.org/cgi-bin/carddisp.pl?gene=lnc-PDRG1-3 |
| lnc-SUGCT-7 |  | RNA Gene | 4 | GC07P041660 | 0.563586891 | https://www.genecards.org/cgi-bin/carddisp.pl?gene=lnc-SUGCT-7 |
| lnc-TTC24-5 |  | RNA Gene | 4 | GC01P156501 | 0.563586891 | https://www.genecards.org/cgi-bin/carddisp.pl?gene=lnc-TTC24-5 |
| MK280607-132 |  | RNA Gene | 4 | GC09M000385 | 0.563586891 | https://www.genecards.org/cgi-bin/carddisp.pl?gene=MK280607-132 |
| MN298678-017 |  | RNA Gene | 4 | GC12M053418 | 0.563586891 | https://www.genecards.org/cgi-bin/carddisp.pl?gene=MN298678-017 |
| piR-41306-056 |  | RNA Gene | 4 | GC12P053586 | 0.563586891 | https://www.genecards.org/cgi-bin/carddisp.pl?gene=piR-41306-056 |
| RF00017-1337 |  | RNA Gene | 4 | GC13M098164 | 0.563586891 | https://www.genecards.org/cgi-bin/carddisp.pl?gene=RF00017-1337 |
| RF00017-5085 |  | RNA Gene | 4 | GC06P058009 | 0.563586891 | https://www.genecards.org/cgi-bin/carddisp.pl?gene=RF00017-5085 |
| HSALNG0090755-001 |  | RNA Gene | 4 | GC12M047365 | 0.563586891 | https://www.genecards.org/cgi-bin/carddisp.pl?gene=HSALNG0090755-001 |
| HSALNG0090755-002 |  | RNA Gene | 4 | GC12M047388 | 0.563586891 | https://www.genecards.org/cgi-bin/carddisp.pl?gene=HSALNG0090755-002 |
| HSALNG0106914-001 |  | RNA Gene | 4 | GC15M070132 | 0.563586891 | https://www.genecards.org/cgi-bin/carddisp.pl?gene=HSALNG0106914-001 |
| HSALNG0106914-002 |  | RNA Gene | 4 | GC15M070134 | 0.563586891 | https://www.genecards.org/cgi-bin/carddisp.pl?gene=HSALNG0106914-002 |
| HSALNG0113883 |  | RNA Gene | 4 | GC17P002073 | 0.563586891 | https://www.genecards.org/cgi-bin/carddisp.pl?gene=HSALNG0113883 |
| HSALNG0129808 |  | RNA Gene | 4 | GC20P035772 | 0.563586891 | https://www.genecards.org/cgi-bin/carddisp.pl?gene=HSALNG0129808 |
| HSALNG0134413 |  | RNA Gene | 4 | GC22M023698 | 0.563586891 | https://www.genecards.org/cgi-bin/carddisp.pl?gene=HSALNG0134413 |
| L13304-034 |  | RNA Gene | 4 | GC08P102533 | 0.563586891 | https://www.genecards.org/cgi-bin/carddisp.pl?gene=L13304-034 |
| lnc-ATP5MC2-1 |  | RNA Gene | 4 | GC12M053600 | 0.563586891 | https://www.genecards.org/cgi-bin/carddisp.pl?gene=lnc-ATP5MC2-1 |
| lnc-C8orf48-9 |  | RNA Gene | 4 | GC08P012965 | 0.563586891 | https://www.genecards.org/cgi-bin/carddisp.pl?gene=lnc-C8orf48-9 |
| lnc-CCDC92-4 |  | RNA Gene | 4 | GC12M124325 | 0.563586891 | https://www.genecards.org/cgi-bin/carddisp.pl?gene=lnc-CCDC92-4 |
| lnc-COL4A3BP-3 |  | RNA Gene | 4 | GC05M075694 | 0.563586891 | https://www.genecards.org/cgi-bin/carddisp.pl?gene=lnc-COL4A3BP-3 |
| lnc-ITGB1-4 |  | RNA Gene | 4 | GC10M033194 | 0.563586891 | https://www.genecards.org/cgi-bin/carddisp.pl?gene=lnc-ITGB1-4 |
| lnc-LGALS7B-2 |  | RNA Gene | 4 | GC19P038713 | 0.563586891 | https://www.genecards.org/cgi-bin/carddisp.pl?gene=lnc-LGALS7B-2 |
| lnc-LRRC49-11 |  | RNA Gene | 4 | GC15P077660 | 0.563586891 | https://www.genecards.org/cgi-bin/carddisp.pl?gene=lnc-LRRC49-11 |
| lnc-POLR3A-6 |  | RNA Gene | 4 | GC10M078589 | 0.563586891 | https://www.genecards.org/cgi-bin/carddisp.pl?gene=lnc-POLR3A-6 |
| lnc-PRR26-3 |  | RNA Gene | 4 | GC10P000690 | 0.563586891 | https://www.genecards.org/cgi-bin/carddisp.pl?gene=lnc-PRR26-3 |
| lnc-TMEM251-3 |  | RNA Gene | 4 | GC14P093119 | 0.563586891 | https://www.genecards.org/cgi-bin/carddisp.pl?gene=lnc-TMEM251-3 |
| lnc-TTC24-4 |  | RNA Gene | 4 | GC01P156503 | 0.563586891 | https://www.genecards.org/cgi-bin/carddisp.pl?gene=lnc-TTC24-4 |
| NONHSAG045706.2 |  | RNA Gene | 4 | GC06P056556 | 0.563586891 | https://www.genecards.org/cgi-bin/carddisp.pl?gene=NONHSAG045706.2 |
| piR-39401-017 |  | RNA Gene | 4 | GC19M047784 | 0.563586891 | https://www.genecards.org/cgi-bin/carddisp.pl?gene=piR-39401-017 |
| piR-57461-229 |  | RNA Gene | 4 | GC07P013970 | 0.563586891 | https://www.genecards.org/cgi-bin/carddisp.pl?gene=piR-57461-229 |
| RF00017-291 |  | RNA Gene | 4 | GC01M150516 | 0.563586891 | https://www.genecards.org/cgi-bin/carddisp.pl?gene=RF00017-291 |
| RF00017-6678 |  | RNA Gene | 4 | GC08P102445 | 0.563586891 | https://www.genecards.org/cgi-bin/carddisp.pl?gene=RF00017-6678 |
| RF00017-6679 |  | RNA Gene | 4 | GC08M102527 | 0.563586891 | https://www.genecards.org/cgi-bin/carddisp.pl?gene=RF00017-6679 |
| LOC101927790 | Uncharacterized LOC101927790 | RNA Gene | 3 | GC05M159936 | 0.563586891 | https://www.genecards.org/cgi-bin/carddisp.pl?gene=LOC101927790 |
| LOC107984988 | Uncharacterized LOC107984988 | RNA Gene | 3 | GC17P002070 | 0.563586891 | https://www.genecards.org/cgi-bin/carddisp.pl?gene=LOC107984988 |
| ENSG00000284616 |  | RNA Gene | 3 | GC01M005301 | 0.563586891 | https://www.genecards.org/cgi-bin/carddisp.pl?gene=ENSG00000284616 |
| ENSG00000284703 |  | RNA Gene | 3 | GC01M004012 | 0.563586891 | https://www.genecards.org/cgi-bin/carddisp.pl?gene=ENSG00000284703 |
| ENSG00000251342 |  | Pseudogene | 3 | GC05P076081 | 0.563586891 | https://www.genecards.org/cgi-bin/carddisp.pl?gene=ENSG00000251342 |
| ENSG00000270234 |  | Pseudogene | 3 | GC10P008724 | 0.563586891 | https://www.genecards.org/cgi-bin/carddisp.pl?gene=ENSG00000270234 |
| ENSG00000277135 |  | RNA Gene | 3 | GC15M096139 | 0.563586891 | https://www.genecards.org/cgi-bin/carddisp.pl?gene=ENSG00000277135 |
| ENSG00000286356 |  | RNA Gene | 3 | GC17M005700 | 0.563586891 | https://www.genecards.org/cgi-bin/carddisp.pl?gene=ENSG00000286356 |
| HG975427 |  | RNA Gene | 3 | GC17M007669 | 0.563586891 | https://www.genecards.org/cgi-bin/carddisp.pl?gene=HG975427 |
| HSALNG0000308 |  | RNA Gene | 3 | GC01M003162 | 0.563586891 | https://www.genecards.org/cgi-bin/carddisp.pl?gene=HSALNG0000308 |
| HSALNG0000309 |  | RNA Gene | 3 | GC01M003180 | 0.563586891 | https://www.genecards.org/cgi-bin/carddisp.pl?gene=HSALNG0000309 |
| LOC105378525 | Uncharacterized LOC105378525 | RNA Gene | 3 | GC10M122449 | 0.563586891 | https://www.genecards.org/cgi-bin/carddisp.pl?gene=LOC105378525 |
| SYPL1P1 | Synaptophysin Like 1 Pseudogene 1 | Pseudogene | 3 | GC03M154652 | 0.563586891 | https://www.genecards.org/cgi-bin/carddisp.pl?gene=SYPL1P1 |
| LOC107985291 | Uncharacterized LOC107985291 | RNA Gene | 3 | GC19M047555 | 0.563586891 | https://www.genecards.org/cgi-bin/carddisp.pl?gene=LOC107985291 |
| HSALNG0024898 |  | RNA Gene | 3 | GC03M030234 | 0.563586891 | https://www.genecards.org/cgi-bin/carddisp.pl?gene=HSALNG0024898 |
| HSALNG0042757 |  | RNA Gene | 3 | GC05M075704 | 0.563586891 | https://www.genecards.org/cgi-bin/carddisp.pl?gene=HSALNG0042757 |
| lnc-BMP8A-2 |  | RNA Gene | 3 | GC01P039541 | 0.563586891 | https://www.genecards.org/cgi-bin/carddisp.pl?gene=lnc-BMP8A-2 |
| HSALNG0001062 |  | RNA Gene | 3 | GC01P015236 | 0.563586891 | https://www.genecards.org/cgi-bin/carddisp.pl?gene=HSALNG0001062 |
| HSALNG0006267-001 |  | RNA Gene | 3 | GC01P115230 | 0.563586891 | https://www.genecards.org/cgi-bin/carddisp.pl?gene=HSALNG0006267-001 |
| HSALNG0050293 |  | RNA Gene | 3 | GC06M047644 | 0.563586891 | https://www.genecards.org/cgi-bin/carddisp.pl?gene=HSALNG0050293 |
| HSALNG0050296 |  | RNA Gene | 3 | GC06M047645 | 0.563586891 | https://www.genecards.org/cgi-bin/carddisp.pl?gene=HSALNG0050296 |
| piR-44851 |  | RNA Gene | 3 | GC01P039526 | 0.563586891 | https://www.genecards.org/cgi-bin/carddisp.pl?gene=piR-44851 |
| piR-45012-246 |  | RNA Gene | 3 | GC18M022530 | 0.563586891 | https://www.genecards.org/cgi-bin/carddisp.pl?gene=piR-45012-246 |
| piR-50444-117 |  | RNA Gene | 3 | GC14M096284 | 0.563586891 | https://www.genecards.org/cgi-bin/carddisp.pl?gene=piR-50444-117 |
| HSALNG0077179 |  | RNA Gene | 3 | GC10P033176 | 0.563586891 | https://www.genecards.org/cgi-bin/carddisp.pl?gene=HSALNG0077179 |
| HSALNG0077181 |  | RNA Gene | 3 | GC10P033197 | 0.563586891 | https://www.genecards.org/cgi-bin/carddisp.pl?gene=HSALNG0077181 |
| HSALNG0077182 |  | RNA Gene | 3 | GC10P033213 | 0.563586891 | https://www.genecards.org/cgi-bin/carddisp.pl?gene=HSALNG0077182 |
| HSALNG0077183 |  | RNA Gene | 3 | GC10M033212 | 0.563586891 | https://www.genecards.org/cgi-bin/carddisp.pl?gene=HSALNG0077183 |
| HSALNG0080745 |  | RNA Gene | 3 | GC10M113429 | 0.563586891 | https://www.genecards.org/cgi-bin/carddisp.pl?gene=HSALNG0080745 |
| HSALNG0121809 |  | RNA Gene | 3 | GC18P058359 | 0.563586891 | https://www.genecards.org/cgi-bin/carddisp.pl?gene=HSALNG0121809 |
| HSALNG0122036 |  | RNA Gene | 3 | GC18P063263 | 0.563586891 | https://www.genecards.org/cgi-bin/carddisp.pl?gene=HSALNG0122036 |
| HSALNG0122034 |  | RNA Gene | 3 | GC18P063206 | 0.563586891 | https://www.genecards.org/cgi-bin/carddisp.pl?gene=HSALNG0122034 |
| HSALNG0129537 |  | RNA Gene | 3 | GC20M032039 | 0.563586891 | https://www.genecards.org/cgi-bin/carddisp.pl?gene=HSALNG0129537 |
| MK280008 |  | RNA Gene | 3 | GC10P033179 | 0.563586891 | https://www.genecards.org/cgi-bin/carddisp.pl?gene=MK280008 |
| NONHSAG005377.2 |  | RNA Gene | 3 | GC10P021535 | 0.563586891 | https://www.genecards.org/cgi-bin/carddisp.pl?gene=NONHSAG005377.2 |
| piR-43103-064 |  | RNA Gene | 3 | GC08P102633 | 0.563586891 | https://www.genecards.org/cgi-bin/carddisp.pl?gene=piR-43103-064 |
| piR-49957 |  | RNA Gene | 3 | GC13M098177 | 0.563586891 | https://www.genecards.org/cgi-bin/carddisp.pl?gene=piR-49957 |
| RF00017-4623 |  | RNA Gene | 3 | GC05M172099 | 0.563586891 | https://www.genecards.org/cgi-bin/carddisp.pl?gene=RF00017-4623 |
| HSALNG0081208 |  | RNA Gene | 3 | GC10M122450 | 0.563586891 | https://www.genecards.org/cgi-bin/carddisp.pl?gene=HSALNG0081208 |
| HSALNG0086158 |  | RNA Gene | 3 | GC11P084437 | 0.563586891 | https://www.genecards.org/cgi-bin/carddisp.pl?gene=HSALNG0086158 |
| HSALNG0119185 |  | RNA Gene | 3 | GC17P080289 | 0.563586891 | https://www.genecards.org/cgi-bin/carddisp.pl?gene=HSALNG0119185 |
| lnc-CPXM2-5 |  | RNA Gene | 3 | GC10M123488 | 0.563586891 | https://www.genecards.org/cgi-bin/carddisp.pl?gene=lnc-CPXM2-5 |
| lnc-FAM117A-2 |  | RNA Gene | 3 | GC17M049701 | 0.563586891 | https://www.genecards.org/cgi-bin/carddisp.pl?gene=lnc-FAM117A-2 |
| lnc-JAM3-10 |  | RNA Gene | 3 | GC11P133957 | 0.563586891 | https://www.genecards.org/cgi-bin/carddisp.pl?gene=lnc-JAM3-10 |
| piR-33605-492 |  | RNA Gene | 3 | GC09M116535 | 0.563586891 | https://www.genecards.org/cgi-bin/carddisp.pl?gene=piR-33605-492 |
| HSALNG0068609-002 |  | RNA Gene | 3 | GC08M130022 | 0.563586891 | https://www.genecards.org/cgi-bin/carddisp.pl?gene=HSALNG0068609-002 |
| HSALNG0071783 |  | RNA Gene | 3 | GC09M070463 | 0.563586891 | https://www.genecards.org/cgi-bin/carddisp.pl?gene=HSALNG0071783 |
| HSALNG0073961 |  | RNA Gene | 3 | GC09M115663 | 0.563586891 | https://www.genecards.org/cgi-bin/carddisp.pl?gene=HSALNG0073961 |
| HSALNG0076596 |  | RNA Gene | 3 | GC10P021530 | 0.563586891 | https://www.genecards.org/cgi-bin/carddisp.pl?gene=HSALNG0076596 |
| HSALNG0117200 |  | RNA Gene | 3 | GC17P049688 | 0.563586891 | https://www.genecards.org/cgi-bin/carddisp.pl?gene=HSALNG0117200 |
| HSALNG0125927 |  | RNA Gene | 3 | GC19M047206 | 0.563586891 | https://www.genecards.org/cgi-bin/carddisp.pl?gene=HSALNG0125927 |
| HSALNG0128884 |  | RNA Gene | 3 | GC20M017608 | 0.563586891 | https://www.genecards.org/cgi-bin/carddisp.pl?gene=HSALNG0128884 |
| HSALNG0128885 |  | RNA Gene | 3 | GC20M017615 | 0.563586891 | https://www.genecards.org/cgi-bin/carddisp.pl?gene=HSALNG0128885 |
| HSALNG0128886 |  | RNA Gene | 3 | GC20M017616 | 0.563586891 | https://www.genecards.org/cgi-bin/carddisp.pl?gene=HSALNG0128886 |
| HSALNG0129538 |  | RNA Gene | 3 | GC20P032041 | 0.563586891 | https://www.genecards.org/cgi-bin/carddisp.pl?gene=HSALNG0129538 |
| lnc-NMUR2-2 |  | RNA Gene | 3 | GC05M152395 | 0.563586891 | https://www.genecards.org/cgi-bin/carddisp.pl?gene=lnc-NMUR2-2 |
| piR-33614-122 |  | RNA Gene | 3 | GC17P080277 | 0.563586891 | https://www.genecards.org/cgi-bin/carddisp.pl?gene=piR-33614-122 |
| piR-38051-139 |  | RNA Gene | 3 | GC18P063234 | 0.563586891 | https://www.genecards.org/cgi-bin/carddisp.pl?gene=piR-38051-139 |
| RF00017-3070 |  | RNA Gene | 3 | GC02P202899 | 0.563586891 | https://www.genecards.org/cgi-bin/carddisp.pl?gene=RF00017-3070 |
| RF00017-6382 |  | RNA Gene | 3 | GC08P030115 | 0.563586891 | https://www.genecards.org/cgi-bin/carddisp.pl?gene=RF00017-6382 |
| RF00017-7030 |  | RNA Gene | 3 | GC09M070471 | 0.563586891 | https://www.genecards.org/cgi-bin/carddisp.pl?gene=RF00017-7030 |
| RF00017-6582 |  | RNA Gene | 3 | GC08P080225 | 0.563586891 | https://www.genecards.org/cgi-bin/carddisp.pl?gene=RF00017-6582 |
| LOC105370328 | Uncharacterized LOC105370328 | RNA Gene | 2 | GC13M098066 | 0.563586891 | https://www.genecards.org/cgi-bin/carddisp.pl?gene=LOC105370328 |
| LOC107984909 | Uncharacterized LOC107984909 | RNA Gene | 2 | GC01P003191 | 0.563586891 | https://www.genecards.org/cgi-bin/carddisp.pl?gene=LOC107984909 |
| LOC105377013 | Uncharacterized LOC105377013 | RNA Gene | 2 | GC03M030422 | 0.563586891 | https://www.genecards.org/cgi-bin/carddisp.pl?gene=LOC105377013 |
| LOC107984269 | Uncharacterized LOC107984269 | RNA Gene | 2 | GC10P113454 | 0.563586891 | https://www.genecards.org/cgi-bin/carddisp.pl?gene=LOC107984269 |
| ENSG00000258679 |  | Pseudogene | 2 | GC12P056823 | 0.563586891 | https://www.genecards.org/cgi-bin/carddisp.pl?gene=ENSG00000258679 |
| ENSG00000279470 |  | Uncategorized | 2 | GC05P051963 | 0.563586891 | https://www.genecards.org/cgi-bin/carddisp.pl?gene=ENSG00000279470 |
| ENSG00000251468 |  | Pseudogene | 2 | GC08P012959 | 0.563586891 | https://www.genecards.org/cgi-bin/carddisp.pl?gene=ENSG00000251468 |
| ENSG00000260750 |  | RNA Gene | 2 | GC16P087595 | 0.563586891 | https://www.genecards.org/cgi-bin/carddisp.pl?gene=ENSG00000260750 |
| ENSG00000262961 |  | Pseudogene | 2 | GC17P005731 | 0.563586891 | https://www.genecards.org/cgi-bin/carddisp.pl?gene=ENSG00000262961 |
| ENSG00000263278 |  | RNA Gene | 2 | GC17M079600 | 0.563586891 | https://www.genecards.org/cgi-bin/carddisp.pl?gene=ENSG00000263278 |
| ENSG00000278399 |  | Pseudogene | 2 | GC12M056983 | 0.563586891 | https://www.genecards.org/cgi-bin/carddisp.pl?gene=ENSG00000278399 |
| ENSG00000229834 |  | Pseudogene | 2 | GC09P029636 | 0.563586891 | https://www.genecards.org/cgi-bin/carddisp.pl?gene=ENSG00000229834 |
| ENSG00000230785 |  | Pseudogene | 2 | GC07M117262 | 0.563586891 | https://www.genecards.org/cgi-bin/carddisp.pl?gene=ENSG00000230785 |
| ENSG00000220745 |  | Pseudogene | 2 | GC06M152959 | 0.563586891 | https://www.genecards.org/cgi-bin/carddisp.pl?gene=ENSG00000220745 |
| LOC101927995 | Uncharacterized LOC101927995 | RNA Gene | 2 | GC03P030357 | 0.563586891 | https://www.genecards.org/cgi-bin/carddisp.pl?gene=LOC101927995 |
| LOC105378606 | Uncharacterized LOC105378606 | RNA Gene | 2 | GC01M003161 | 0.563586891 | https://www.genecards.org/cgi-bin/carddisp.pl?gene=LOC105378606 |
| LOC105376475 | Uncharacterized LOC105376475 | RNA Gene | 2 | GC10M029782 | 0.563586891 | https://www.genecards.org/cgi-bin/carddisp.pl?gene=LOC105376475 |
| LOC105376235 | Uncharacterized LOC105376235 | RNA Gene | 2 | GC09P115596 | 0.563586891 | https://www.genecards.org/cgi-bin/carddisp.pl?gene=LOC105376235 |
| lnc-FZD9-2 |  | RNA Gene | 2 | GC07P073437 | 0.563586891 | https://www.genecards.org/cgi-bin/carddisp.pl?gene=lnc-FZD9-2 |
| lnc-PRR26-6 |  | RNA Gene | 2 | GC10P000733 | 0.563586891 | https://www.genecards.org/cgi-bin/carddisp.pl?gene=lnc-PRR26-6 |
| HSALNG0024979 |  | RNA Gene | 2 | GC03M032199 | 0.563586891 | https://www.genecards.org/cgi-bin/carddisp.pl?gene=HSALNG0024979 |
| HSALNG0024981 |  | RNA Gene | 2 | GC03M032207 | 0.563586891 | https://www.genecards.org/cgi-bin/carddisp.pl?gene=HSALNG0024981 |
| HSALNG0053163 |  | RNA Gene | 2 | GC06M121488 | 0.563586891 | https://www.genecards.org/cgi-bin/carddisp.pl?gene=HSALNG0053163 |
| ENSG00000242586 |  | Pseudogene | 2 | GC03P080442 | 0.563586891 | https://www.genecards.org/cgi-bin/carddisp.pl?gene=ENSG00000242586 |
| HSALNG0064340 |  | RNA Gene | 2 | GC08M030124 | 0.563586891 | https://www.genecards.org/cgi-bin/carddisp.pl?gene=HSALNG0064340 |
| lnc-ZFAND1-2 |  | RNA Gene | 2 | GC08M081814 | 0.563586891 | https://www.genecards.org/cgi-bin/carddisp.pl?gene=lnc-ZFAND1-2 |
| HSALNG0000315 |  | RNA Gene | 2 | GC01P003293 | 0.563586891 | https://www.genecards.org/cgi-bin/carddisp.pl?gene=HSALNG0000315 |
| HSALNG0002774 |  | RNA Gene | 2 | GC01M039528 | 0.563586891 | https://www.genecards.org/cgi-bin/carddisp.pl?gene=HSALNG0002774 |
| HSALNG0021649 |  | RNA Gene | 2 | GC02P202913 | 0.563586891 | https://www.genecards.org/cgi-bin/carddisp.pl?gene=HSALNG0021649 |
| HSALNG0053474-001 |  | RNA Gene | 2 | GC06M131086 | 0.563586891 | https://www.genecards.org/cgi-bin/carddisp.pl?gene=HSALNG0053474-001 |
| HSALNG0029973 |  | RNA Gene | 2 | GC03M154558 | 0.563586891 | https://www.genecards.org/cgi-bin/carddisp.pl?gene=HSALNG0029973 |
| HSALNG0029974 |  | RNA Gene | 2 | GC03M154602 | 0.563586891 | https://www.genecards.org/cgi-bin/carddisp.pl?gene=HSALNG0029974 |
| HSALNG0042770 |  | RNA Gene | 2 | GC05M076191 | 0.563586891 | https://www.genecards.org/cgi-bin/carddisp.pl?gene=HSALNG0042770 |
| HSALNG0045544 |  | RNA Gene | 2 | GC05P143849 | 0.563586891 | https://www.genecards.org/cgi-bin/carddisp.pl?gene=HSALNG0045544 |
| HSALNG0049912 |  | RNA Gene | 2 | GC06P055927 | 0.563586891 | https://www.genecards.org/cgi-bin/carddisp.pl?gene=HSALNG0049912 |
| HSALNG0053474-002 |  | RNA Gene | 2 | GC06M131088 | 0.563586891 | https://www.genecards.org/cgi-bin/carddisp.pl?gene=HSALNG0053474-002 |
| HSALNG0075499-001 |  | RNA Gene | 2 | GC10M000694 | 0.563586891 | https://www.genecards.org/cgi-bin/carddisp.pl?gene=HSALNG0075499-001 |
| HSALNG0075499-002 |  | RNA Gene | 2 | GC10M000695 | 0.563586891 | https://www.genecards.org/cgi-bin/carddisp.pl?gene=HSALNG0075499-002 |
| HSALNG0088349 |  | RNA Gene | 2 | GC11P133958 | 0.563586891 | https://www.genecards.org/cgi-bin/carddisp.pl?gene=HSALNG0088349 |
| L13713-043 |  | RNA Gene | 2 | GC11M084506 | 0.563586891 | https://www.genecards.org/cgi-bin/carddisp.pl?gene=L13713-043 |
| piR-32214-606 |  | RNA Gene | 2 | GC07M013938 | 0.563586891 | https://www.genecards.org/cgi-bin/carddisp.pl?gene=piR-32214-606 |
| HSALNG0079113 |  | RNA Gene | 2 | GC10P085456 | 0.563586891 | https://www.genecards.org/cgi-bin/carddisp.pl?gene=HSALNG0079113 |
| HSALNG0079112 |  | RNA Gene | 2 | GC10M078762 | 0.563586891 | https://www.genecards.org/cgi-bin/carddisp.pl?gene=HSALNG0079112 |
| HSALNG0121808 |  | RNA Gene | 2 | GC18P058333 | 0.563586891 | https://www.genecards.org/cgi-bin/carddisp.pl?gene=HSALNG0121808 |
| HSALNG0131452 |  | RNA Gene | 2 | GC20M061697 | 0.563586891 | https://www.genecards.org/cgi-bin/carddisp.pl?gene=HSALNG0131452 |
| piR-33848 |  | RNA Gene | 2 | GC06M000608 | 0.563586891 | https://www.genecards.org/cgi-bin/carddisp.pl?gene=piR-33848 |
| piR-38351-100 |  | RNA Gene | 2 | GC12M058739 | 0.563586891 | https://www.genecards.org/cgi-bin/carddisp.pl?gene=piR-38351-100 |
| piR-43107-111 |  | RNA Gene | 2 | GC16P075404 | 0.563586891 | https://www.genecards.org/cgi-bin/carddisp.pl?gene=piR-43107-111 |
| lnc-MOG-1 |  | RNA Gene | 2 | GC06P056279 | 0.563586891 | https://www.genecards.org/cgi-bin/carddisp.pl?gene=lnc-MOG-1 |
| piR-57133-090 |  | RNA Gene | 2 | GC11M102213 | 0.563586891 | https://www.genecards.org/cgi-bin/carddisp.pl?gene=piR-57133-090 |
| HSALNG0080845 |  | RNA Gene | 2 | GC10P116163 | 0.563586891 | https://www.genecards.org/cgi-bin/carddisp.pl?gene=HSALNG0080845 |
| HSALNG0081207 |  | RNA Gene | 2 | GC10P122444 | 0.563586891 | https://www.genecards.org/cgi-bin/carddisp.pl?gene=HSALNG0081207 |
| HSALNG0081293 |  | RNA Gene | 2 | GC10M123464 | 0.563586891 | https://www.genecards.org/cgi-bin/carddisp.pl?gene=HSALNG0081293 |
| HSALNG0081295 |  | RNA Gene | 2 | GC10M123498 | 0.563586891 | https://www.genecards.org/cgi-bin/carddisp.pl?gene=HSALNG0081295 |
| HSALNG0086159 |  | RNA Gene | 2 | GC11P084466 | 0.563586891 | https://www.genecards.org/cgi-bin/carddisp.pl?gene=HSALNG0086159 |
| lnc-NRAP-3 |  | RNA Gene | 2 | GC10M113474 | 0.563586891 | https://www.genecards.org/cgi-bin/carddisp.pl?gene=lnc-NRAP-3 |
| MN309062 |  | RNA Gene | 2 | GC10P016593 | 0.563586891 | https://www.genecards.org/cgi-bin/carddisp.pl?gene=MN309062 |
| piR-55948-023 |  | RNA Gene | 2 | GC12P058710 | 0.563586891 | https://www.genecards.org/cgi-bin/carddisp.pl?gene=piR-55948-023 |
| piR-57135-091 |  | RNA Gene | 2 | GC08M030135 | 0.563586891 | https://www.genecards.org/cgi-bin/carddisp.pl?gene=piR-57135-091 |
| RF00017-4707 |  | RNA Gene | 2 | GC06M000628 | 0.563586891 | https://www.genecards.org/cgi-bin/carddisp.pl?gene=RF00017-4707 |
| RF00017-5785 |  | RNA Gene | 2 | GC07M073459 | 0.563586891 | https://www.genecards.org/cgi-bin/carddisp.pl?gene=RF00017-5785 |
| HSALNG0066598 |  | RNA Gene | 2 | GC08M081828 | 0.563586891 | https://www.genecards.org/cgi-bin/carddisp.pl?gene=HSALNG0066598 |
| HSALNG0068609-001 |  | RNA Gene | 2 | GC08M130021 | 0.563586891 | https://www.genecards.org/cgi-bin/carddisp.pl?gene=HSALNG0068609-001 |
| HSALNG0075991 |  | RNA Gene | 2 | GC10P006881 | 0.563586891 | https://www.genecards.org/cgi-bin/carddisp.pl?gene=HSALNG0075991 |
| HSALNG0114148 |  | RNA Gene | 2 | GC17P005668 | 0.563586891 | https://www.genecards.org/cgi-bin/carddisp.pl?gene=HSALNG0114148 |
| HSALNG0117204 |  | RNA Gene | 2 | GC17M049695 | 0.563586891 | https://www.genecards.org/cgi-bin/carddisp.pl?gene=HSALNG0117204 |
| lnc-C8orf48-10 |  | RNA Gene | 2 | GC08P012949 | 0.563586891 | https://www.genecards.org/cgi-bin/carddisp.pl?gene=lnc-C8orf48-10 |
| piR-37026-414 |  | RNA Gene | 2 | GC03P030412 | 0.563586891 | https://www.genecards.org/cgi-bin/carddisp.pl?gene=piR-37026-414 |
| piR-39341-230 |  | RNA Gene | 2 | GC03M030429 | 0.563586891 | https://www.genecards.org/cgi-bin/carddisp.pl?gene=piR-39341-230 |
| piR-43106-210 |  | RNA Gene | 2 | GC09M116421 | 0.563586891 | https://www.genecards.org/cgi-bin/carddisp.pl?gene=piR-43106-210 |
| piR-46847-041 |  | RNA Gene | 2 | GC10M103007 | 0.563586891 | https://www.genecards.org/cgi-bin/carddisp.pl?gene=piR-46847-041 |
| piR-50983 |  | RNA Gene | 2 | GC06P131069 | 0.563586891 | https://www.genecards.org/cgi-bin/carddisp.pl?gene=piR-50983 |
| piR-61532-243 |  | RNA Gene | 2 | GC05P075701 | 0.563586891 | https://www.genecards.org/cgi-bin/carddisp.pl?gene=piR-61532-243 |
| RF00017-6587 |  | RNA Gene | 2 | GC08P080363 | 0.563586891 | https://www.genecards.org/cgi-bin/carddisp.pl?gene=RF00017-6587 |
| RF00017-710 |  | RNA Gene | 2 | GC10M102959 | 0.563586891 | https://www.genecards.org/cgi-bin/carddisp.pl?gene=RF00017-710 |
| LOC105375922 | Uncharacterized LOC105375922 | RNA Gene | 1 | GC08M080440 | 0.563586891 | https://www.genecards.org/cgi-bin/carddisp.pl?gene=LOC105375922 |
| LOC390332 | Retinol Dehydrogenase 16 (All-Trans) Pseudogene | Pseudogene | 1 | GC12M056981 | 0.563586891 | https://www.genecards.org/cgi-bin/carddisp.pl?gene=LOC390332 |
| lnc-PTER-3 |  | RNA Gene | 1 | GC10P016567 | 0.563586891 | https://www.genecards.org/cgi-bin/carddisp.pl?gene=lnc-PTER-3 |
| HSALNG0045627 |  | RNA Gene | 1 | GC05P146369 | 0.563586891 | https://www.genecards.org/cgi-bin/carddisp.pl?gene=HSALNG0045627 |
| HSALNG0016992 |  | RNA Gene | 1 | GC02P095824 | 0.563586891 | https://www.genecards.org/cgi-bin/carddisp.pl?gene=HSALNG0016992 |
| HSALNG0042771 |  | RNA Gene | 1 | GC05M076230 | 0.563586891 | https://www.genecards.org/cgi-bin/carddisp.pl?gene=HSALNG0042771 |
| HSALNG0088350 |  | RNA Gene | 1 | GC11M133959 | 0.563586891 | https://www.genecards.org/cgi-bin/carddisp.pl?gene=HSALNG0088350 |
| HSALNG0066504-001 |  | RNA Gene | 1 | GC08P080440 | 0.563586891 | https://www.genecards.org/cgi-bin/carddisp.pl?gene=HSALNG0066504-001 |
| HSALNG0080030-001 |  | RNA Gene | 1 | GC10P098817 | 0.563586891 | https://www.genecards.org/cgi-bin/carddisp.pl?gene=HSALNG0080030-001 |
| HSALNG0080030-002 |  | RNA Gene | 1 | GC10P098878 | 0.563586891 | https://www.genecards.org/cgi-bin/carddisp.pl?gene=HSALNG0080030-002 |
| RF00994-148 |  | RNA Gene | 1 | GC10P098886 | 0.563586891 | https://www.genecards.org/cgi-bin/carddisp.pl?gene=RF00994-148 |
| piR-61532-044 |  | RNA Gene | 1 | GC10M098905 | 0.563586891 | https://www.genecards.org/cgi-bin/carddisp.pl?gene=piR-61532-044 |
| G6PD | Glucose-6-Phosphate Dehydrogenase | Protein Coding | 51 | GC0XM154531 | 0.5617553 | https://www.genecards.org/cgi-bin/carddisp.pl?gene=G6PD |
| TTR | Transthyretin | Protein Coding | 50 | GC18P031557 | 0.5617553 | https://www.genecards.org/cgi-bin/carddisp.pl?gene=TTR |
| CYP2C8 | Cytochrome P450 Family 2 Subfamily C Member 8 | Protein Coding | 49 | GC10M095038 | 0.5617553 | https://www.genecards.org/cgi-bin/carddisp.pl?gene=CYP2C8 |
| PLA2G2A | Phospholipase A2 Group IIA | Protein Coding | 48 | GC01M019975 | 0.5617553 | https://www.genecards.org/cgi-bin/carddisp.pl?gene=PLA2G2A |
| PTGS1 | Prostaglandin-Endoperoxide Synthase 1 | Protein Coding | 47 | GC09P122370 | 0.5617553 | https://www.genecards.org/cgi-bin/carddisp.pl?gene=PTGS1 |
| HSPG2 | Heparan Sulfate Proteoglycan 2 | Protein Coding | 46 | GC01M021822 | 0.5617553 | https://www.genecards.org/cgi-bin/carddisp.pl?gene=HSPG2 |
| ALOX5AP | Arachidonate 5-Lipoxygenase Activating Protein | Protein Coding | 45 | GC13P030713 | 0.5617553 | https://www.genecards.org/cgi-bin/carddisp.pl?gene=ALOX5AP |
| CANT1 | Calcium Activated Nucleotidase 1 | Protein Coding | 45 | GC17M078992 | 0.5617553 | https://www.genecards.org/cgi-bin/carddisp.pl?gene=CANT1 |
| PDE4A | Phosphodiesterase 4A | Protein Coding | 45 | GC19P010416 | 0.5617553 | https://www.genecards.org/cgi-bin/carddisp.pl?gene=PDE4A |
| ALOX12 | Arachidonate 12-Lipoxygenase, 12S Type | Protein Coding | 44 | GC17P006995 | 0.5617553 | https://www.genecards.org/cgi-bin/carddisp.pl?gene=ALOX12 |
| UGT1A | UDP Glucuronosyltransferase Family 1 Member A Complex Locus | Genetic Locus | 10 | GC02P233592 | 0.5617553 | https://www.genecards.org/cgi-bin/carddisp.pl?gene=UGT1A |
| AAAS | Aladin WD Repeat Nucleoporin | Protein Coding | 42 | GC12M053307 | 0.556700468 | https://www.genecards.org/cgi-bin/carddisp.pl?gene=AAAS |
| CA8 | Carbonic Anhydrase 8 | Protein Coding | 48 | GC08M060187 | 0.551405191 | https://www.genecards.org/cgi-bin/carddisp.pl?gene=CA8 |
| TGM6 | Transglutaminase 6 | Protein Coding | 38 | GC20P002380 | 0.551405191 | https://www.genecards.org/cgi-bin/carddisp.pl?gene=TGM6 |
| MARCHF6 | Membrane Associated Ring-CH-Type Finger 6 | Protein Coding | 30 | GC05P010372 | 0.551405191 | https://www.genecards.org/cgi-bin/carddisp.pl?gene=MARCHF6 |
| BCL2L1 | BCL2 Like 1 | Protein Coding | 47 | GC20M031664 | 0.526652098 | https://www.genecards.org/cgi-bin/carddisp.pl?gene=BCL2L1 |
| GHR | Growth Hormone Receptor | Protein Coding | 47 | GC05P042429 | 0.526652098 | https://www.genecards.org/cgi-bin/carddisp.pl?gene=GHR |
| ASL | Argininosuccinate Lyase | Protein Coding | 47 | GC07P066075 | 0.526652098 | https://www.genecards.org/cgi-bin/carddisp.pl?gene=ASL |
| ASPA | Aspartoacylase | Protein Coding | 45 | GC17P003472 | 0.526652098 | https://www.genecards.org/cgi-bin/carddisp.pl?gene=ASPA |
| AURKA | Aurora Kinase A | Protein Coding | 52 | GC20M056370 | 0.490443528 | https://www.genecards.org/cgi-bin/carddisp.pl?gene=AURKA |
| ALK | ALK Receptor Tyrosine Kinase | Protein Coding | 52 | GC02M029190 | 0.490443528 | https://www.genecards.org/cgi-bin/carddisp.pl?gene=ALK |
| CASP8 | Caspase 8 | Protein Coding | 52 | GC02P201233 | 0.490443528 | https://www.genecards.org/cgi-bin/carddisp.pl?gene=CASP8 |
| CHRNA4 | Cholinergic Receptor Nicotinic Alpha 4 Subunit | Protein Coding | 50 | GC20M063343 | 0.490443528 | https://www.genecards.org/cgi-bin/carddisp.pl?gene=CHRNA4 |
| HSD11B1 | Hydroxysteroid 11-Beta Dehydrogenase 1 | Protein Coding | 50 | GC01P209686 | 0.490443528 | https://www.genecards.org/cgi-bin/carddisp.pl?gene=HSD11B1 |
| BMPR2 | Bone Morphogenetic Protein Receptor Type 2 | Protein Coding | 50 | GC02P202376 | 0.490443528 | https://www.genecards.org/cgi-bin/carddisp.pl?gene=BMPR2 |
| TBP | TATA-Box Binding Protein | Protein Coding | 49 | GC06P170554 | 0.490443528 | https://www.genecards.org/cgi-bin/carddisp.pl?gene=TBP |
| IGF2 | Insulin Like Growth Factor 2 | Protein Coding | 48 | GC11M002130 | 0.490443528 | https://www.genecards.org/cgi-bin/carddisp.pl?gene=IGF2 |
| TRAF3 | TNF Receptor Associated Factor 3 | Protein Coding | 48 | GC14P106036 | 0.490443528 | https://www.genecards.org/cgi-bin/carddisp.pl?gene=TRAF3 |
| F9 | Coagulation Factor IX | Protein Coding | 47 | GC0XP139530 | 0.490443528 | https://www.genecards.org/cgi-bin/carddisp.pl?gene=F9 |
| MS4A1 | Membrane Spanning 4-Domains A1 | Protein Coding | 47 | GC11P060475 | 0.490443528 | https://www.genecards.org/cgi-bin/carddisp.pl?gene=MS4A1 |
| PRODH | Proline Dehydrogenase 1 | Protein Coding | 47 | GC22M018912 | 0.490443528 | https://www.genecards.org/cgi-bin/carddisp.pl?gene=PRODH |
| SLC7A7 | Solute Carrier Family 7 Member 7 | Protein Coding | 47 | GC14M022773 | 0.490443528 | https://www.genecards.org/cgi-bin/carddisp.pl?gene=SLC7A7 |
| PAX5 | Paired Box 5 | Protein Coding | 47 | GC09M036828 | 0.490443528 | https://www.genecards.org/cgi-bin/carddisp.pl?gene=PAX5 |
| NDUFS1 | NADH:Ubiquinone Oxidoreductase Core Subunit S1 | Protein Coding | 47 | GC02M206114 | 0.490443528 | https://www.genecards.org/cgi-bin/carddisp.pl?gene=NDUFS1 |
| NDUFS7 | NADH:Ubiquinone Oxidoreductase Core Subunit S7 | Protein Coding | 47 | GC19P001635 | 0.490443528 | https://www.genecards.org/cgi-bin/carddisp.pl?gene=NDUFS7 |
| NDUFS8 | NADH:Ubiquinone Oxidoreductase Core Subunit S8 | Protein Coding | 46 | GC11P068030 | 0.490443528 | https://www.genecards.org/cgi-bin/carddisp.pl?gene=NDUFS8 |
| AMPD1 | Adenosine Monophosphate Deaminase 1 | Protein Coding | 46 | GC01M114673 | 0.490443528 | https://www.genecards.org/cgi-bin/carddisp.pl?gene=AMPD1 |
| FCGR3A | Fc Fragment Of IgG Receptor IIIa | Protein Coding | 45 | GC01M161541 | 0.490443528 | https://www.genecards.org/cgi-bin/carddisp.pl?gene=FCGR3A |
| CD274 | CD274 Molecule | Protein Coding | 45 | GC09P005450 | 0.490443528 | https://www.genecards.org/cgi-bin/carddisp.pl?gene=CD274 |
| LARS2 | Leucyl-TRNA Synthetase 2, Mitochondrial | Protein Coding | 45 | GC03P045606 | 0.490443528 | https://www.genecards.org/cgi-bin/carddisp.pl?gene=LARS2 |
| IL12RB1 | Interleukin 12 Receptor Subunit Beta 1 | Protein Coding | 45 | GC19M018058 | 0.490443528 | https://www.genecards.org/cgi-bin/carddisp.pl?gene=IL12RB1 |
| NDUFA1 | NADH:Ubiquinone Oxidoreductase Subunit A1 | Protein Coding | 45 | GC0XP119871 | 0.490443528 | https://www.genecards.org/cgi-bin/carddisp.pl?gene=NDUFA1 |
| NDUFV2 | NADH:Ubiquinone Oxidoreductase Core Subunit V2 | Protein Coding | 45 | GC18P009092 | 0.490443528 | https://www.genecards.org/cgi-bin/carddisp.pl?gene=NDUFV2 |
| NLRC4 | NLR Family CARD Domain Containing 4 | Protein Coding | 45 | GC02M032224 | 0.490443528 | https://www.genecards.org/cgi-bin/carddisp.pl?gene=NLRC4 |
| APTX | Aprataxin | Protein Coding | 45 | GC09M032886 | 0.490443528 | https://www.genecards.org/cgi-bin/carddisp.pl?gene=APTX |
| ATXN3 | Ataxin 3 | Protein Coding | 45 | GC14M095796 | 0.490443528 | https://www.genecards.org/cgi-bin/carddisp.pl?gene=ATXN3 |
| BCS1L | BCS1 Homolog, Ubiquinol-Cytochrome C Reductase Complex Chaperone | Protein Coding | 45 | GC02P218658 | 0.490443528 | https://www.genecards.org/cgi-bin/carddisp.pl?gene=BCS1L |
| CS | Citrate Synthase | Protein Coding | 45 | GC12M056271 | 0.490443528 | https://www.genecards.org/cgi-bin/carddisp.pl?gene=CS |
| WARS2 | Tryptophanyl TRNA Synthetase 2, Mitochondrial | Protein Coding | 45 | GC01M119031 | 0.490443528 | https://www.genecards.org/cgi-bin/carddisp.pl?gene=WARS2 |
| TNFSF13B | TNF Superfamily Member 13b | Protein Coding | 45 | GC13P108251 | 0.490443528 | https://www.genecards.org/cgi-bin/carddisp.pl?gene=TNFSF13B |
| NDUFS2 | NADH:Ubiquinone Oxidoreductase Core Subunit S2 | Protein Coding | 45 | GC01P161197 | 0.490443528 | https://www.genecards.org/cgi-bin/carddisp.pl?gene=NDUFS2 |
| PRTN3 | Proteinase 3 | Protein Coding | 45 | GC19P000840 | 0.490443528 | https://www.genecards.org/cgi-bin/carddisp.pl?gene=PRTN3 |
| ATN1 | Atrophin 1 | Protein Coding | 44 | GC12P011868 | 0.490443528 | https://www.genecards.org/cgi-bin/carddisp.pl?gene=ATN1 |
| ACAD9 | Acyl-CoA Dehydrogenase Family Member 9 | Protein Coding | 44 | GC03P131450 | 0.490443528 | https://www.genecards.org/cgi-bin/carddisp.pl?gene=ACAD9 |
| COX10 | Cytochrome C Oxidase Assembly Factor Heme A:Farnesyltransferase COX10 | Protein Coding | 44 | GC17P014069 | 0.490443528 | https://www.genecards.org/cgi-bin/carddisp.pl?gene=COX10 |
| IL12RB2 | Interleukin 12 Receptor Subunit Beta 2 | Protein Coding | 44 | GC01P067307 | 0.490443528 | https://www.genecards.org/cgi-bin/carddisp.pl?gene=IL12RB2 |
| TSFM | Ts Translation Elongation Factor, Mitochondrial | Protein Coding | 44 | GC12P057778 | 0.490443528 | https://www.genecards.org/cgi-bin/carddisp.pl?gene=TSFM |
| SSTR1 | Somatostatin Receptor 1 | Protein Coding | 44 | GC14P038207 | 0.490443528 | https://www.genecards.org/cgi-bin/carddisp.pl?gene=SSTR1 |
| TNXB | Tenascin XB | Protein Coding | 44 | GC06M046900 | 0.490443528 | https://www.genecards.org/cgi-bin/carddisp.pl?gene=TNXB |
| PMPCA | Peptidase, Mitochondrial Processing Subunit Alpha | Protein Coding | 44 | GC09P136410 | 0.490443528 | https://www.genecards.org/cgi-bin/carddisp.pl?gene=PMPCA |
| SLC25A24 | Solute Carrier Family 25 Member 24 | Protein Coding | 44 | GC01M108134 | 0.490443528 | https://www.genecards.org/cgi-bin/carddisp.pl?gene=SLC25A24 |
| SMAD7 | SMAD Family Member 7 | Protein Coding | 44 | GC18M048919 | 0.490443528 | https://www.genecards.org/cgi-bin/carddisp.pl?gene=SMAD7 |
| NLRP12 | NLR Family Pyrin Domain Containing 12 | Protein Coding | 44 | GC19M053793 | 0.490443528 | https://www.genecards.org/cgi-bin/carddisp.pl?gene=NLRP12 |
| COQ6 | Coenzyme Q6, Monooxygenase | Protein Coding | 43 | GC14P073949 | 0.490443528 | https://www.genecards.org/cgi-bin/carddisp.pl?gene=COQ6 |
| COX6B1 | Cytochrome C Oxidase Subunit 6B1 | Protein Coding | 43 | GC19P040358 | 0.490443528 | https://www.genecards.org/cgi-bin/carddisp.pl?gene=COX6B1 |
| COX15 | Cytochrome C Oxidase Assembly Homolog COX15 | Protein Coding | 43 | GC10M099696 | 0.490443528 | https://www.genecards.org/cgi-bin/carddisp.pl?gene=COX15 |
| GDF15 | Growth Differentiation Factor 15 | Protein Coding | 43 | GC19P026656 | 0.490443528 | https://www.genecards.org/cgi-bin/carddisp.pl?gene=GDF15 |
| DDAH2 | Dimethylarginine Dimethylaminohydrolase 2 | Protein Coding | 43 | GC06M031727 | 0.490443528 | https://www.genecards.org/cgi-bin/carddisp.pl?gene=DDAH2 |
| BNIP3 | BCL2 Interacting Protein 3 | Protein Coding | 43 | GC10M131966 | 0.490443528 | https://www.genecards.org/cgi-bin/carddisp.pl?gene=BNIP3 |
| CHGA | Chromogranin A | Protein Coding | 43 | GC14P092925 | 0.490443528 | https://www.genecards.org/cgi-bin/carddisp.pl?gene=CHGA |
| ELAC2 | ElaC Ribonuclease Z 2 | Protein Coding | 43 | GC17M012991 | 0.490443528 | https://www.genecards.org/cgi-bin/carddisp.pl?gene=ELAC2 |
| MTO1 | Mitochondrial TRNA Translation Optimization 1 | Protein Coding | 43 | GC06P073461 | 0.490443528 | https://www.genecards.org/cgi-bin/carddisp.pl?gene=MTO1 |
| TRIT1 | TRNA Isopentenyltransferase 1 | Protein Coding | 43 | GC01M039842 | 0.490443528 | https://www.genecards.org/cgi-bin/carddisp.pl?gene=TRIT1 |
| TFB1M | Transcription Factor B1, Mitochondrial | Protein Coding | 43 | GC06M155247 | 0.490443528 | https://www.genecards.org/cgi-bin/carddisp.pl?gene=TFB1M |
| PDSS1 | Decaprenyl Diphosphate Synthase Subunit 1 | Protein Coding | 43 | GC10P026697 | 0.490443528 | https://www.genecards.org/cgi-bin/carddisp.pl?gene=PDSS1 |
| PYCARD | PYD And CARD Domain Containing | Protein Coding | 43 | GC16M031201 | 0.490443528 | https://www.genecards.org/cgi-bin/carddisp.pl?gene=PYCARD |
| NDUFB8 | NADH:Ubiquinone Oxidoreductase Subunit B8 | Protein Coding | 43 | GC10M100523 | 0.490443528 | https://www.genecards.org/cgi-bin/carddisp.pl?gene=NDUFB8 |
| NDUFB3 | NADH:Ubiquinone Oxidoreductase Subunit B3 | Protein Coding | 43 | GC02P201071 | 0.490443528 | https://www.genecards.org/cgi-bin/carddisp.pl?gene=NDUFB3 |
| COQ9 | Coenzyme Q9 | Protein Coding | 42 | GC16P057447 | 0.490443528 | https://www.genecards.org/cgi-bin/carddisp.pl?gene=COQ9 |
| COX5B | Cytochrome C Oxidase Subunit 5B | Protein Coding | 42 | GC02P097628 | 0.490443528 | https://www.genecards.org/cgi-bin/carddisp.pl?gene=COX5B |
| IL17F | Interleukin 17F | Protein Coding | 42 | GC06M052209 | 0.490443528 | https://www.genecards.org/cgi-bin/carddisp.pl?gene=IL17F |
| IARS2 | Isoleucyl-TRNA Synthetase 2, Mitochondrial | Protein Coding | 42 | GC01P220094 | 0.490443528 | https://www.genecards.org/cgi-bin/carddisp.pl?gene=IARS2 |
| MRPS22 | Mitochondrial Ribosomal Protein S22 | Protein Coding | 42 | GC03P139005 | 0.490443528 | https://www.genecards.org/cgi-bin/carddisp.pl?gene=MRPS22 |
| GTPBP3 | GTP Binding Protein 3, Mitochondrial | Protein Coding | 41 | GC19P026637 | 0.490443528 | https://www.genecards.org/cgi-bin/carddisp.pl?gene=GTPBP3 |
| BGLAP | Bone Gamma-Carboxyglutamate Protein | Protein Coding | 41 | GC01P156242 | 0.490443528 | https://www.genecards.org/cgi-bin/carddisp.pl?gene=BGLAP |
| ADI1 | Acireductone Dioxygenase 1 | Protein Coding | 41 | GC02M003501 | 0.490443528 | https://www.genecards.org/cgi-bin/carddisp.pl?gene=ADI1 |
| PDSS2 | Decaprenyl Diphosphate Synthase Subunit 2 | Protein Coding | 41 | GC06M107152 | 0.490443528 | https://www.genecards.org/cgi-bin/carddisp.pl?gene=PDSS2 |
| KDM4C | Lysine Demethylase 4C | Protein Coding | 41 | GC09P006720 | 0.490443528 | https://www.genecards.org/cgi-bin/carddisp.pl?gene=KDM4C |
| COX6C | Cytochrome C Oxidase Subunit 6C | Protein Coding | 40 | GC08M099899 | 0.490443528 | https://www.genecards.org/cgi-bin/carddisp.pl?gene=COX6C |
| IFNA1 | Interferon Alpha 1 | Protein Coding | 40 | GC09P021522 | 0.490443528 | https://www.genecards.org/cgi-bin/carddisp.pl?gene=IFNA1 |
| FASTKD2 | FAST Kinase Domains 2 | Protein Coding | 40 | GC02P206766 | 0.490443528 | https://www.genecards.org/cgi-bin/carddisp.pl?gene=FASTKD2 |
| FGF21 | Fibroblast Growth Factor 21 | Protein Coding | 40 | GC19P048781 | 0.490443528 | https://www.genecards.org/cgi-bin/carddisp.pl?gene=FGF21 |
| AKAP1 | A-Kinase Anchoring Protein 1 | Protein Coding | 40 | GC17P057085 | 0.490443528 | https://www.genecards.org/cgi-bin/carddisp.pl?gene=AKAP1 |
| MRPL44 | Mitochondrial Ribosomal Protein L44 | Protein Coding | 40 | GC02P223957 | 0.490443528 | https://www.genecards.org/cgi-bin/carddisp.pl?gene=MRPL44 |
| NDUFB6 | NADH:Ubiquinone Oxidoreductase Subunit B6 | Protein Coding | 40 | GC09M032553 | 0.490443528 | https://www.genecards.org/cgi-bin/carddisp.pl?gene=NDUFB6 |
| NES | Nestin | Protein Coding | 40 | GC01M156668 | 0.490443528 | https://www.genecards.org/cgi-bin/carddisp.pl?gene=NES |
| FBXL4 | F-Box And Leucine Rich Repeat Protein 4 | Protein Coding | 39 | GC06M098868 | 0.490443528 | https://www.genecards.org/cgi-bin/carddisp.pl?gene=FBXL4 |
| TMEM126B | Transmembrane Protein 126B | Protein Coding | 39 | GC11P085628 | 0.490443528 | https://www.genecards.org/cgi-bin/carddisp.pl?gene=TMEM126B |
| COX7C | Cytochrome C Oxidase Subunit 7C | Protein Coding | 38 | GC05P086617 | 0.490443528 | https://www.genecards.org/cgi-bin/carddisp.pl?gene=COX7C |
| CDK5RAP1 | CDK5 Regulatory Subunit Associated Protein 1 | Protein Coding | 38 | GC20M033358 | 0.490443528 | https://www.genecards.org/cgi-bin/carddisp.pl?gene=CDK5RAP1 |
| TMEM70 | Transmembrane Protein 70 | Protein Coding | 38 | GC08P073972 | 0.490443528 | https://www.genecards.org/cgi-bin/carddisp.pl?gene=TMEM70 |
| GARS1 | Glycyl-TRNA Synthetase 1 | Protein Coding | 37 | GC07P030580 | 0.490443528 | https://www.genecards.org/cgi-bin/carddisp.pl?gene=GARS1 |
| ATP6V0E1 | ATPase H+ Transporting V0 Subunit E1 | Protein Coding | 37 | GC05P172983 | 0.490443528 | https://www.genecards.org/cgi-bin/carddisp.pl?gene=ATP6V0E1 |
| CAPN8 | Calpain 8 | Protein Coding | 37 | GC01M223538 | 0.490443528 | https://www.genecards.org/cgi-bin/carddisp.pl?gene=CAPN8 |
| OSGEPL1 | O-Sialoglycoprotein Endopeptidase Like 1 | Protein Coding | 37 | GC02M189746 | 0.490443528 | https://www.genecards.org/cgi-bin/carddisp.pl?gene=OSGEPL1 |
| MRPL18 | Mitochondrial Ribosomal Protein L18 | Protein Coding | 37 | GC06P159789 | 0.490443528 | https://www.genecards.org/cgi-bin/carddisp.pl?gene=MRPL18 |
| MRPL43 | Mitochondrial Ribosomal Protein L43 | Protein Coding | 37 | GC10M100969 | 0.490443528 | https://www.genecards.org/cgi-bin/carddisp.pl?gene=MRPL43 |
| BLOC1S1 | Biogenesis Of Lysosomal Organelles Complex 1 Subunit 1 | Protein Coding | 36 | GC12P055765 | 0.490443528 | https://www.genecards.org/cgi-bin/carddisp.pl?gene=BLOC1S1 |
| GPR22 | G Protein-Coupled Receptor 22 | Protein Coding | 36 | GC07P107470 | 0.490443528 | https://www.genecards.org/cgi-bin/carddisp.pl?gene=GPR22 |
| ZFYVE21 | Zinc Finger FYVE-Type Containing 21 | Protein Coding | 36 | GC14P103715 | 0.490443528 | https://www.genecards.org/cgi-bin/carddisp.pl?gene=ZFYVE21 |
| RIMBP2 | RIMS Binding Protein 2 | Protein Coding | 36 | GC12M130396 | 0.490443528 | https://www.genecards.org/cgi-bin/carddisp.pl?gene=RIMBP2 |
| COA3 | Cytochrome C Oxidase Assembly Factor 3 | Protein Coding | 35 | GC17M042795 | 0.490443528 | https://www.genecards.org/cgi-bin/carddisp.pl?gene=COA3 |
| MTERF1 | Mitochondrial Transcription Termination Factor 1 | Protein Coding | 35 | GC07M091692 | 0.490443528 | https://www.genecards.org/cgi-bin/carddisp.pl?gene=MTERF1 |
| ARRDC4 | Arrestin Domain Containing 4 | Protein Coding | 34 | GC15P097960 | 0.490443528 | https://www.genecards.org/cgi-bin/carddisp.pl?gene=ARRDC4 |
| TRMT61B | TRNA Methyltransferase 61B | Protein Coding | 34 | GC02M028849 | 0.490443528 | https://www.genecards.org/cgi-bin/carddisp.pl?gene=TRMT61B |
| OR4L1 | Olfactory Receptor Family 4 Subfamily L Member 1 | Protein Coding | 34 | GC14P020060 | 0.490443528 | https://www.genecards.org/cgi-bin/carddisp.pl?gene=OR4L1 |
| MT-ND3 | Mitochondrially Encoded NADH:Ubiquinone Oxidoreductase Core Subunit 3 | Protein Coding | 33 | GCMTP010061 | 0.490443528 | https://www.genecards.org/cgi-bin/carddisp.pl?gene=MT-ND3 |
| YRDC | YrdC N6-Threonylcarbamoyltransferase Domain Containing | Protein Coding | 32 | GC01M037802 | 0.490443528 | https://www.genecards.org/cgi-bin/carddisp.pl?gene=YRDC |
| MRM2 | Mitochondrial RRNA Methyltransferase 2 | Protein Coding | 31 | GC07M002234 | 0.490443528 | https://www.genecards.org/cgi-bin/carddisp.pl?gene=MRM2 |
| RERGL | RERG Like | Protein Coding | 31 | GC12M018080 | 0.490443528 | https://www.genecards.org/cgi-bin/carddisp.pl?gene=RERGL |
| MT-ATP8 | Mitochondrially Encoded ATP Synthase Membrane Subunit 8 | Protein Coding | 30 | GCMTP008368 | 0.490443528 | https://www.genecards.org/cgi-bin/carddisp.pl?gene=MT-ATP8 |
| SLF2 | SMC5-SMC6 Complex Localization Factor 2 | Protein Coding | 26 | GC10P100913 | 0.490443528 | https://www.genecards.org/cgi-bin/carddisp.pl?gene=SLF2 |
| HOTAIR | HOX Transcript Antisense RNA | RNA Gene | 26 | GC12M053962 | 0.490443528 | https://www.genecards.org/cgi-bin/carddisp.pl?gene=HOTAIR |
| MIR155 | MicroRNA 155 | RNA Gene | 19 | GC21P025560 | 0.490443528 | https://www.genecards.org/cgi-bin/carddisp.pl?gene=MIR155 |
| MT-RNR1 | Mitochondrially Encoded 12S RRNA | RNA Gene | 15 | GCMTP000642 | 0.490443528 | https://www.genecards.org/cgi-bin/carddisp.pl?gene=MT-RNR1 |
| MIR4786 | MicroRNA 4786 | RNA Gene | 13 | GC02M239943 | 0.490443528 | https://www.genecards.org/cgi-bin/carddisp.pl?gene=MIR4786 |
| TRL-AAG2-3 | TRNA-Leu (Anticodon AAG) 2-3 | RNA Gene | 10 | GC14P021552 | 0.490443528 | https://www.genecards.org/cgi-bin/carddisp.pl?gene=TRL-AAG2-3 |
| CA2 | Carbonic Anhydrase 2 | Protein Coding | 52 | GC08P085463 | 0.473973453 | https://www.genecards.org/cgi-bin/carddisp.pl?gene=CA2 |
| JUN | Jun Proto-Oncogene, AP-1 Transcription Factor Subunit | Protein Coding | 50 | GC01M058780 | 0.473973453 | https://www.genecards.org/cgi-bin/carddisp.pl?gene=JUN |
| LRP5 | LDL Receptor Related Protein 5 | Protein Coding | 49 | GC11P068298 | 0.473973453 | https://www.genecards.org/cgi-bin/carddisp.pl?gene=LRP5 |
| B2M | Beta-2-Microglobulin | Protein Coding | 49 | GC15P044711 | 0.473973453 | https://www.genecards.org/cgi-bin/carddisp.pl?gene=B2M |
| ABCC2 | ATP Binding Cassette Subfamily C Member 2 | Protein Coding | 48 | GC10P099782 | 0.473973453 | https://www.genecards.org/cgi-bin/carddisp.pl?gene=ABCC2 |
| SMAD2 | SMAD Family Member 2 | Protein Coding | 48 | GC18M047809 | 0.473973453 | https://www.genecards.org/cgi-bin/carddisp.pl?gene=SMAD2 |
| PRLR | Prolactin Receptor | Protein Coding | 48 | GC05M035048 | 0.473973453 | https://www.genecards.org/cgi-bin/carddisp.pl?gene=PRLR |
| CA1 | Carbonic Anhydrase 1 | Protein Coding | 47 | GC08M085327 | 0.473973453 | https://www.genecards.org/cgi-bin/carddisp.pl?gene=CA1 |
| TGFB3 | Transforming Growth Factor Beta 3 | Protein Coding | 47 | GC14M075958 | 0.473973453 | https://www.genecards.org/cgi-bin/carddisp.pl?gene=TGFB3 |
| ANGPT2 | Angiopoietin 2 | Protein Coding | 46 | GC08M006499 | 0.473973453 | https://www.genecards.org/cgi-bin/carddisp.pl?gene=ANGPT2 |
| KCNQ4 | Potassium Voltage-Gated Channel Subfamily Q Member 4 | Protein Coding | 45 | GC01P040784 | 0.473973453 | https://www.genecards.org/cgi-bin/carddisp.pl?gene=KCNQ4 |
| CA14 | Carbonic Anhydrase 14 | Protein Coding | 44 | GC01P150258 | 0.473973453 | https://www.genecards.org/cgi-bin/carddisp.pl?gene=CA14 |
| CA5B | Carbonic Anhydrase 5B | Protein Coding | 41 | GC0XP015688 | 0.473973453 | https://www.genecards.org/cgi-bin/carddisp.pl?gene=CA5B |
| CA7 | Carbonic Anhydrase 7 | Protein Coding | 40 | GC16P066844 | 0.473973453 | https://www.genecards.org/cgi-bin/carddisp.pl?gene=CA7 |
| CA13 | Carbonic Anhydrase 13 | Protein Coding | 38 | GC08P085221 | 0.473973453 | https://www.genecards.org/cgi-bin/carddisp.pl?gene=CA13 |
| SLC9A1 | Solute Carrier Family 9 Member A1 | Protein Coding | 51 | GC01M027109 | 0.452179015 | https://www.genecards.org/cgi-bin/carddisp.pl?gene=SLC9A1 |
| ADRA1A | Adrenoceptor Alpha 1A | Protein Coding | 48 | GC08M026747 | 0.452179015 | https://www.genecards.org/cgi-bin/carddisp.pl?gene=ADRA1A |
| SCN2B | Sodium Voltage-Gated Channel Beta Subunit 2 | Protein Coding | 44 | GC11M118163 | 0.452179015 | https://www.genecards.org/cgi-bin/carddisp.pl?gene=SCN2B |
| SCN4B | Sodium Voltage-Gated Channel Beta Subunit 4 | Protein Coding | 44 | GC11M118134 | 0.452179015 | https://www.genecards.org/cgi-bin/carddisp.pl?gene=SCN4B |
| PPIA | Peptidylprolyl Isomerase A | Protein Coding | 48 | GC07P044807 | 0.446691155 | https://www.genecards.org/cgi-bin/carddisp.pl?gene=PPIA |
| ERBB2 | Erb-B2 Receptor Tyrosine Kinase 2 | Protein Coding | 54 | GC17P039687 | 0.434577763 | https://www.genecards.org/cgi-bin/carddisp.pl?gene=ERBB2 |
| CDK4 | Cyclin Dependent Kinase 4 | Protein Coding | 54 | GC12M057743 | 0.434577763 | https://www.genecards.org/cgi-bin/carddisp.pl?gene=CDK4 |
| RAF1 | Raf-1 Proto-Oncogene, Serine/Threonine Kinase | Protein Coding | 54 | GC03M012583 | 0.434577763 | https://www.genecards.org/cgi-bin/carddisp.pl?gene=RAF1 |
| IKBKB | Inhibitor Of Nuclear Factor Kappa B Kinase Subunit Beta | Protein Coding | 53 | GC08P042271 | 0.434577763 | https://www.genecards.org/cgi-bin/carddisp.pl?gene=IKBKB |
| HMOX1 | Heme Oxygenase 1 | Protein Coding | 53 | GC22P035380 | 0.434577763 | https://www.genecards.org/cgi-bin/carddisp.pl?gene=HMOX1 |
| MAPK1 | Mitogen-Activated Protein Kinase 1 | Protein Coding | 52 | GC22M021754 | 0.434577763 | https://www.genecards.org/cgi-bin/carddisp.pl?gene=MAPK1 |
| GAD1 | Glutamate Decarboxylase 1 | Protein Coding | 52 | GC02P170813 | 0.434577763 | https://www.genecards.org/cgi-bin/carddisp.pl?gene=GAD1 |
| HGF | Hepatocyte Growth Factor | Protein Coding | 52 | GC07M081699 | 0.434577763 | https://www.genecards.org/cgi-bin/carddisp.pl?gene=HGF |
| CHUK | Component Of Inhibitor Of Nuclear Factor Kappa B Kinase Complex | Protein Coding | 52 | GC10M100188 | 0.434577763 | https://www.genecards.org/cgi-bin/carddisp.pl?gene=CHUK |
| CFTR | CF Transmembrane Conductance Regulator | Protein Coding | 52 | GC07P117287 | 0.434577763 | https://www.genecards.org/cgi-bin/carddisp.pl?gene=CFTR |
| PRKCA | Protein Kinase C Alpha | Protein Coding | 52 | GC17P066302 | 0.434577763 | https://www.genecards.org/cgi-bin/carddisp.pl?gene=PRKCA |
| PCNA | Proliferating Cell Nuclear Antigen | Protein Coding | 52 | GC20M005114 | 0.434577763 | https://www.genecards.org/cgi-bin/carddisp.pl?gene=PCNA |
| PLAU | Plasminogen Activator, Urokinase | Protein Coding | 52 | GC10P073909 | 0.434577763 | https://www.genecards.org/cgi-bin/carddisp.pl?gene=PLAU |
| DNMT1 | DNA Methyltransferase 1 | Protein Coding | 51 | GC19M010133 | 0.434577763 | https://www.genecards.org/cgi-bin/carddisp.pl?gene=DNMT1 |
| GSK3B | Glycogen Synthase Kinase 3 Beta | Protein Coding | 51 | GC03M119821 | 0.434577763 | https://www.genecards.org/cgi-bin/carddisp.pl?gene=GSK3B |
| HNF4A | Hepatocyte Nuclear Factor 4 Alpha | Protein Coding | 51 | GC20P044355 | 0.434577763 | https://www.genecards.org/cgi-bin/carddisp.pl?gene=HNF4A |
| ABCG2 | ATP Binding Cassette Subfamily G Member 2 (Junior Blood Group) | Protein Coding | 51 | GC04M088090 | 0.434577763 | https://www.genecards.org/cgi-bin/carddisp.pl?gene=ABCG2 |
| CDH1 | Cadherin 1 | Protein Coding | 51 | GC16P068737 | 0.434577763 | https://www.genecards.org/cgi-bin/carddisp.pl?gene=CDH1 |
| PRKAA2 | Protein Kinase AMP-Activated Catalytic Subunit Alpha 2 | Protein Coding | 51 | GC01P056645 | 0.434577763 | https://www.genecards.org/cgi-bin/carddisp.pl?gene=PRKAA2 |
| PRKAG2 | Protein Kinase AMP-Activated Non-Catalytic Subunit Gamma 2 | Protein Coding | 51 | GC07M151556 | 0.434577763 | https://www.genecards.org/cgi-bin/carddisp.pl?gene=PRKAG2 |
| RPS6KA1 | Ribosomal Protein S6 Kinase A1 | Protein Coding | 51 | GC01P026540 | 0.434577763 | https://www.genecards.org/cgi-bin/carddisp.pl?gene=RPS6KA1 |
| GLUD1 | Glutamate Dehydrogenase 1 | Protein Coding | 50 | GC10M087050 | 0.434577763 | https://www.genecards.org/cgi-bin/carddisp.pl?gene=GLUD1 |
| CYLD | CYLD Lysine 63 Deubiquitinase | Protein Coding | 50 | GC16P050742 | 0.434577763 | https://www.genecards.org/cgi-bin/carddisp.pl?gene=CYLD |
| CYP11A1 | Cytochrome P450 Family 11 Subfamily A Member 1 | Protein Coding | 50 | GC15M074337 | 0.434577763 | https://www.genecards.org/cgi-bin/carddisp.pl?gene=CYP11A1 |
| CYP2C9 | Cytochrome P450 Family 2 Subfamily C Member 9 | Protein Coding | 50 | GC10P094938 | 0.434577763 | https://www.genecards.org/cgi-bin/carddisp.pl?gene=CYP2C9 |
| ALOX5 | Arachidonate 5-Lipoxygenase | Protein Coding | 50 | GC10P045374 | 0.434577763 | https://www.genecards.org/cgi-bin/carddisp.pl?gene=ALOX5 |
| PTK2B | Protein Tyrosine Kinase 2 Beta | Protein Coding | 50 | GC08P027311 | 0.434577763 | https://www.genecards.org/cgi-bin/carddisp.pl?gene=PTK2B |
| THRA | Thyroid Hormone Receptor Alpha | Protein Coding | 50 | GC17P040058 | 0.434577763 | https://www.genecards.org/cgi-bin/carddisp.pl?gene=THRA |
| PLA2G4A | Phospholipase A2 Group IVA | Protein Coding | 50 | GC01P186798 | 0.434577763 | https://www.genecards.org/cgi-bin/carddisp.pl?gene=PLA2G4A |
| PLA2G7 | Phospholipase A2 Group VII | Protein Coding | 50 | GC06M047382 | 0.434577763 | https://www.genecards.org/cgi-bin/carddisp.pl?gene=PLA2G7 |
| NTRK1 | Neurotrophic Receptor Tyrosine Kinase 1 | Protein Coding | 50 | GC01P156815 | 0.434577763 | https://www.genecards.org/cgi-bin/carddisp.pl?gene=NTRK1 |
| IL2RB | Interleukin 2 Receptor Subunit Beta | Protein Coding | 49 | GC22M037125 | 0.434577763 | https://www.genecards.org/cgi-bin/carddisp.pl?gene=IL2RB |
| IMPA1 | Inositol Monophosphatase 1 | Protein Coding | 49 | GC08M081656 | 0.434577763 | https://www.genecards.org/cgi-bin/carddisp.pl?gene=IMPA1 |
| MAPK7 | Mitogen-Activated Protein Kinase 7 | Protein Coding | 49 | GC17P019379 | 0.434577763 | https://www.genecards.org/cgi-bin/carddisp.pl?gene=MAPK7 |
| F12 | Coagulation Factor XII | Protein Coding | 49 | GC05M177402 | 0.434577763 | https://www.genecards.org/cgi-bin/carddisp.pl?gene=F12 |
| BCHE | Butyrylcholinesterase | Protein Coding | 49 | GC03M165772 | 0.434577763 | https://www.genecards.org/cgi-bin/carddisp.pl?gene=BCHE |
| TBXA2R | Thromboxane A2 Receptor | Protein Coding | 49 | GC19M003594 | 0.434577763 | https://www.genecards.org/cgi-bin/carddisp.pl?gene=TBXA2R |
| PLCG1 | Phospholipase C Gamma 1 | Protein Coding | 49 | GC20P041136 | 0.434577763 | https://www.genecards.org/cgi-bin/carddisp.pl?gene=PLCG1 |
| PRKAG1 | Protein Kinase AMP-Activated Non-Catalytic Subunit Gamma 1 | Protein Coding | 49 | GC12M049002 | 0.434577763 | https://www.genecards.org/cgi-bin/carddisp.pl?gene=PRKAG1 |
| ODC1 | Ornithine Decarboxylase 1 | Protein Coding | 49 | GC02M010432 | 0.434577763 | https://www.genecards.org/cgi-bin/carddisp.pl?gene=ODC1 |
| PTGER2 | Prostaglandin E Receptor 2 | Protein Coding | 49 | GC14P052314 | 0.434577763 | https://www.genecards.org/cgi-bin/carddisp.pl?gene=PTGER2 |
| GABBR1 | Gamma-Aminobutyric Acid Type B Receptor Subunit 1 | Protein Coding | 48 | GC06M029555 | 0.434577763 | https://www.genecards.org/cgi-bin/carddisp.pl?gene=GABBR1 |
| CYSLTR2 | Cysteinyl Leukotriene Receptor 2 | Protein Coding | 48 | GC13P048653 | 0.434577763 | https://www.genecards.org/cgi-bin/carddisp.pl?gene=CYSLTR2 |
| IRS1 | Insulin Receptor Substrate 1 | Protein Coding | 48 | GC02M226731 | 0.434577763 | https://www.genecards.org/cgi-bin/carddisp.pl?gene=IRS1 |
| GUSB | Glucuronidase Beta | Protein Coding | 48 | GC07M065960 | 0.434577763 | https://www.genecards.org/cgi-bin/carddisp.pl?gene=GUSB |
| HSPA5 | Heat Shock Protein Family A (Hsp70) Member 5 | Protein Coding | 48 | GC09M125234 | 0.434577763 | https://www.genecards.org/cgi-bin/carddisp.pl?gene=HSPA5 |
| GGT1 | Gamma-Glutamyltransferase 1 | Protein Coding | 48 | GC22P024583 | 0.434577763 | https://www.genecards.org/cgi-bin/carddisp.pl?gene=GGT1 |
| GBA | Glucosylceramidase Beta | Protein Coding | 48 | GC01M155234 | 0.434577763 | https://www.genecards.org/cgi-bin/carddisp.pl?gene=GBA |
| ABCA1 | ATP Binding Cassette Subfamily A Member 1 | Protein Coding | 48 | GC09M104781 | 0.434577763 | https://www.genecards.org/cgi-bin/carddisp.pl?gene=ABCA1 |
| CASP9 | Caspase 9 | Protein Coding | 48 | GC01M015491 | 0.434577763 | https://www.genecards.org/cgi-bin/carddisp.pl?gene=CASP9 |
| EPHX2 | Epoxide Hydrolase 2 | Protein Coding | 48 | GC08P027490 | 0.434577763 | https://www.genecards.org/cgi-bin/carddisp.pl?gene=EPHX2 |
| TNFRSF11B | TNF Receptor Superfamily Member 11b | Protein Coding | 48 | GC08M118923 | 0.434577763 | https://www.genecards.org/cgi-bin/carddisp.pl?gene=TNFRSF11B |
| PRKAA1 | Protein Kinase AMP-Activated Catalytic Subunit Alpha 1 | Protein Coding | 48 | GC05M040759 | 0.434577763 | https://www.genecards.org/cgi-bin/carddisp.pl?gene=PRKAA1 |
| PRKAB1 | Protein Kinase AMP-Activated Non-Catalytic Subunit Beta 1 | Protein Coding | 48 | GC12P119632 | 0.434577763 | https://www.genecards.org/cgi-bin/carddisp.pl?gene=PRKAB1 |
| PLA2G6 | Phospholipase A2 Group VI | Protein Coding | 48 | GC22M049636 | 0.434577763 | https://www.genecards.org/cgi-bin/carddisp.pl?gene=PLA2G6 |
| PTGIR | Prostaglandin I2 Receptor | Protein Coding | 48 | GC19M047072 | 0.434577763 | https://www.genecards.org/cgi-bin/carddisp.pl?gene=PTGIR |
| PTGIS | Prostaglandin I2 Synthase | Protein Coding | 48 | GC20M049503 | 0.434577763 | https://www.genecards.org/cgi-bin/carddisp.pl?gene=PTGIS |
| PTGDR | Prostaglandin D2 Receptor | Protein Coding | 48 | GC14P052267 | 0.434577763 | https://www.genecards.org/cgi-bin/carddisp.pl?gene=PTGDR |
| FPR2 | Formyl Peptide Receptor 2 | Protein Coding | 47 | GC19P051752 | 0.434577763 | https://www.genecards.org/cgi-bin/carddisp.pl?gene=FPR2 |
| ITGAM | Integrin Subunit Alpha M | Protein Coding | 47 | GC16P032553 | 0.434577763 | https://www.genecards.org/cgi-bin/carddisp.pl?gene=ITGAM |
| KLK3 | Kallikrein Related Peptidase 3 | Protein Coding | 47 | GC19P050854 | 0.434577763 | https://www.genecards.org/cgi-bin/carddisp.pl?gene=KLK3 |
| HSP90B1 | Heat Shock Protein 90 Beta Family Member 1 | Protein Coding | 47 | GC12P103930 | 0.434577763 | https://www.genecards.org/cgi-bin/carddisp.pl?gene=HSP90B1 |
| HMGCR | 3-Hydroxy-3-Methylglutaryl-CoA Reductase | Protein Coding | 47 | GC05P075336 | 0.434577763 | https://www.genecards.org/cgi-bin/carddisp.pl?gene=HMGCR |
| F2RL3 | F2R Like Thrombin Or Trypsin Receptor 3 | Protein Coding | 47 | GC19P016888 | 0.434577763 | https://www.genecards.org/cgi-bin/carddisp.pl?gene=F2RL3 |
| F13A1 | Coagulation Factor XIII A Chain | Protein Coding | 47 | GC06M006144 | 0.434577763 | https://www.genecards.org/cgi-bin/carddisp.pl?gene=F13A1 |
| CETP | Cholesteryl Ester Transfer Protein | Protein Coding | 47 | GC16P056961 | 0.434577763 | https://www.genecards.org/cgi-bin/carddisp.pl?gene=CETP |
| BIRC5 | Baculoviral IAP Repeat Containing 5 | Protein Coding | 47 | GC17P078214 | 0.434577763 | https://www.genecards.org/cgi-bin/carddisp.pl?gene=BIRC5 |
| PRKAB2 | Protein Kinase AMP-Activated Non-Catalytic Subunit Beta 2 | Protein Coding | 47 | GC01M147155 | 0.434577763 | https://www.genecards.org/cgi-bin/carddisp.pl?gene=PRKAB2 |
| SLC22A5 | Solute Carrier Family 22 Member 5 | Protein Coding | 47 | GC05P132369 | 0.434577763 | https://www.genecards.org/cgi-bin/carddisp.pl?gene=SLC22A5 |
| P2RY1 | Purinergic Receptor P2Y1 | Protein Coding | 47 | GC03P152835 | 0.434577763 | https://www.genecards.org/cgi-bin/carddisp.pl?gene=P2RY1 |
| PTGER3 | Prostaglandin E Receptor 3 | Protein Coding | 47 | GC01M070852 | 0.434577763 | https://www.genecards.org/cgi-bin/carddisp.pl?gene=PTGER3 |
| HRH1 | Histamine Receptor H1 | Protein Coding | 46 | GC03P011113 | 0.434577763 | https://www.genecards.org/cgi-bin/carddisp.pl?gene=HRH1 |
| BID | BH3 Interacting Domain Death Agonist | Protein Coding | 46 | GC22M017734 | 0.434577763 | https://www.genecards.org/cgi-bin/carddisp.pl?gene=BID |
| ATP2A3 | ATPase Sarcoplasmic/Endoplasmic Reticulum Ca2+ Transporting 3 | Protein Coding | 46 | GC17M003923 | 0.434577763 | https://www.genecards.org/cgi-bin/carddisp.pl?gene=ATP2A3 |
| APOB | Apolipoprotein B | Protein Coding | 46 | GC02M020956 | 0.434577763 | https://www.genecards.org/cgi-bin/carddisp.pl?gene=APOB |
| XBP1 | X-Box Binding Protein 1 | Protein Coding | 46 | GC22M028794 | 0.434577763 | https://www.genecards.org/cgi-bin/carddisp.pl?gene=XBP1 |
| TNFRSF11A | TNF Receptor Superfamily Member 11a | Protein Coding | 46 | GC18P062325 | 0.434577763 | https://www.genecards.org/cgi-bin/carddisp.pl?gene=TNFRSF11A |
| PEPD | Peptidase D | Protein Coding | 46 | GC19M033386 | 0.434577763 | https://www.genecards.org/cgi-bin/carddisp.pl?gene=PEPD |
| SCARB1 | Scavenger Receptor Class B Member 1 | Protein Coding | 46 | GC12M124776 | 0.434577763 | https://www.genecards.org/cgi-bin/carddisp.pl?gene=SCARB1 |
| CYP4F2 | Cytochrome P450 Family 4 Subfamily F Member 2 | Protein Coding | 45 | GC19M015878 | 0.434577763 | https://www.genecards.org/cgi-bin/carddisp.pl?gene=CYP4F2 |
| LGALS3 | Galectin 3 | Protein Coding | 45 | GC14P055124 | 0.434577763 | https://www.genecards.org/cgi-bin/carddisp.pl?gene=LGALS3 |
| HRH2 | Histamine Receptor H2 | Protein Coding | 45 | GC05P175659 | 0.434577763 | https://www.genecards.org/cgi-bin/carddisp.pl?gene=HRH2 |
| KITLG | KIT Ligand | Protein Coding | 45 | GC12M088492 | 0.434577763 | https://www.genecards.org/cgi-bin/carddisp.pl?gene=KITLG |
| MAPK4 | Mitogen-Activated Protein Kinase 4 | Protein Coding | 45 | GC18P050564 | 0.434577763 | https://www.genecards.org/cgi-bin/carddisp.pl?gene=MAPK4 |
| MAPK6 | Mitogen-Activated Protein Kinase 6 | Protein Coding | 45 | GC15P051952 | 0.434577763 | https://www.genecards.org/cgi-bin/carddisp.pl?gene=MAPK6 |
| LOX | Lysyl Oxidase | Protein Coding | 45 | GC05M122063 | 0.434577763 | https://www.genecards.org/cgi-bin/carddisp.pl?gene=LOX |
| ADH7 | Alcohol Dehydrogenase 7 (Class IV), Mu Or Sigma Polypeptide | Protein Coding | 45 | GC04M099412 | 0.434577763 | https://www.genecards.org/cgi-bin/carddisp.pl?gene=ADH7 |
| TFPI | Tissue Factor Pathway Inhibitor | Protein Coding | 45 | GC02M187464 | 0.434577763 | https://www.genecards.org/cgi-bin/carddisp.pl?gene=TFPI |
| TFF1 | Trefoil Factor 1 | Protein Coding | 45 | GC21M042362 | 0.434577763 | https://www.genecards.org/cgi-bin/carddisp.pl?gene=TFF1 |
| SLC17A5 | Solute Carrier Family 17 Member 5 | Protein Coding | 45 | GC06M073593 | 0.434577763 | https://www.genecards.org/cgi-bin/carddisp.pl?gene=SLC17A5 |
| SLC22A6 | Solute Carrier Family 22 Member 6 | Protein Coding | 45 | GC11M069278 | 0.434577763 | https://www.genecards.org/cgi-bin/carddisp.pl?gene=SLC22A6 |
| CPOX | Coproporphyrinogen Oxidase | Protein Coding | 44 | GC03M098576 | 0.434577763 | https://www.genecards.org/cgi-bin/carddisp.pl?gene=CPOX |
| CYSLTR1 | Cysteinyl Leukotriene Receptor 1 | Protein Coding | 44 | GC0XM078271 | 0.434577763 | https://www.genecards.org/cgi-bin/carddisp.pl?gene=CYSLTR1 |
| FCER1G | Fc Fragment Of IgE Receptor Ig | Protein Coding | 44 | GC01P161215 | 0.434577763 | https://www.genecards.org/cgi-bin/carddisp.pl?gene=FCER1G |
| CSF1 | Colony Stimulating Factor 1 | Protein Coding | 44 | GC01P109911 | 0.434577763 | https://www.genecards.org/cgi-bin/carddisp.pl?gene=CSF1 |
| AKR1C1 | Aldo-Keto Reductase Family 1 Member C1 | Protein Coding | 44 | GC10P004963 | 0.434577763 | https://www.genecards.org/cgi-bin/carddisp.pl?gene=AKR1C1 |
| CEBPB | CCAAT Enhancer Binding Protein Beta | Protein Coding | 44 | GC20P050190 | 0.434577763 | https://www.genecards.org/cgi-bin/carddisp.pl?gene=CEBPB |
| P2RX1 | Purinergic Receptor P2X 1 | Protein Coding | 44 | GC17M003896 | 0.434577763 | https://www.genecards.org/cgi-bin/carddisp.pl?gene=P2RX1 |
| TAPBP | TAP Binding Protein | Protein Coding | 44 | GC06M033299 | 0.434577763 | https://www.genecards.org/cgi-bin/carddisp.pl?gene=TAPBP |
| VASP | Vasodilator Stimulated Phosphoprotein | Protein Coding | 44 | GC19P045507 | 0.434577763 | https://www.genecards.org/cgi-bin/carddisp.pl?gene=VASP |
| VTN | Vitronectin | Protein Coding | 44 | GC17M031079 | 0.434577763 | https://www.genecards.org/cgi-bin/carddisp.pl?gene=VTN |
| PRKAG3 | Protein Kinase AMP-Activated Non-Catalytic Subunit Gamma 3 | Protein Coding | 44 | GC02M218823 | 0.434577763 | https://www.genecards.org/cgi-bin/carddisp.pl?gene=PRKAG3 |
| SLC6A12 | Solute Carrier Family 6 Member 12 | Protein Coding | 44 | GC12M000169 | 0.434577763 | https://www.genecards.org/cgi-bin/carddisp.pl?gene=SLC6A12 |
| SERPINB5 | Serpin Family B Member 5 | Protein Coding | 44 | GC18P063476 | 0.434577763 | https://www.genecards.org/cgi-bin/carddisp.pl?gene=SERPINB5 |
| CGA | Glycoprotein Hormones, Alpha Polypeptide | Protein Coding | 43 | GC06M087085 | 0.434577763 | https://www.genecards.org/cgi-bin/carddisp.pl?gene=CGA |
| CHIA | Chitinase Acidic | Protein Coding | 43 | GC01P111291 | 0.434577763 | https://www.genecards.org/cgi-bin/carddisp.pl?gene=CHIA |
| CD63 | CD63 Molecule | Protein Coding | 43 | GC12M055725 | 0.434577763 | https://www.genecards.org/cgi-bin/carddisp.pl?gene=CD63 |
| ATG4A | Autophagy Related 4A Cysteine Peptidase | Protein Coding | 43 | GC0XP108091 | 0.434577763 | https://www.genecards.org/cgi-bin/carddisp.pl?gene=ATG4A |
| NAGLU | N-Acetyl-Alpha-Glucosaminidase | Protein Coding | 43 | GC17P042537 | 0.434577763 | https://www.genecards.org/cgi-bin/carddisp.pl?gene=NAGLU |
| SULT1A1 | Sulfotransferase Family 1A Member 1 | Protein Coding | 43 | GC16M028606 | 0.434577763 | https://www.genecards.org/cgi-bin/carddisp.pl?gene=SULT1A1 |
| SLC22A8 | Solute Carrier Family 22 Member 8 | Protein Coding | 43 | GC11M069279 | 0.434577763 | https://www.genecards.org/cgi-bin/carddisp.pl?gene=SLC22A8 |
| NTF3 | Neurotrophin 3 | Protein Coding | 43 | GC12P005432 | 0.434577763 | https://www.genecards.org/cgi-bin/carddisp.pl?gene=NTF3 |
| ATG12 | Autophagy Related 12 | Protein Coding | 42 | GC05M115828 | 0.434577763 | https://www.genecards.org/cgi-bin/carddisp.pl?gene=ATG12 |
| ATG3 | Autophagy Related 3 | Protein Coding | 42 | GC03M112532 | 0.434577763 | https://www.genecards.org/cgi-bin/carddisp.pl?gene=ATG3 |
| DUSP2 | Dual Specificity Phosphatase 2 | Protein Coding | 42 | GC02M096271 | 0.434577763 | https://www.genecards.org/cgi-bin/carddisp.pl?gene=DUSP2 |
| UGT1A6 | UDP Glucuronosyltransferase Family 1 Member A6 | Protein Coding | 42 | GC02P233691 | 0.434577763 | https://www.genecards.org/cgi-bin/carddisp.pl?gene=UGT1A6 |
| SLC30A9 | Solute Carrier Family 30 Member 9 | Protein Coding | 42 | GC04P041992 | 0.434577763 | https://www.genecards.org/cgi-bin/carddisp.pl?gene=SLC30A9 |
| SMN1 | Survival Of Motor Neuron 1, Telomeric | Protein Coding | 42 | GC05P070924 | 0.434577763 | https://www.genecards.org/cgi-bin/carddisp.pl?gene=SMN1 |
| MAPK15 | Mitogen-Activated Protein Kinase 15 | Protein Coding | 41 | GC08P143716 | 0.434577763 | https://www.genecards.org/cgi-bin/carddisp.pl?gene=MAPK15 |
| GRP | Gastrin Releasing Peptide | Protein Coding | 41 | GC18P059220 | 0.434577763 | https://www.genecards.org/cgi-bin/carddisp.pl?gene=GRP |
| ATG4B | Autophagy Related 4B Cysteine Peptidase | Protein Coding | 41 | GC02P241637 | 0.434577763 | https://www.genecards.org/cgi-bin/carddisp.pl?gene=ATG4B |
| TNFAIP6 | TNF Alpha Induced Protein 6 | Protein Coding | 41 | GC02P151357 | 0.434577763 | https://www.genecards.org/cgi-bin/carddisp.pl?gene=TNFAIP6 |
| TFF2 | Trefoil Factor 2 | Protein Coding | 41 | GC21M042346 | 0.434577763 | https://www.genecards.org/cgi-bin/carddisp.pl?gene=TFF2 |
| TFF3 | Trefoil Factor 3 | Protein Coding | 41 | GC21M042311 | 0.434577763 | https://www.genecards.org/cgi-bin/carddisp.pl?gene=TFF3 |
| ATG4C | Autophagy Related 4C Cysteine Peptidase | Protein Coding | 40 | GC01P062784 | 0.434577763 | https://www.genecards.org/cgi-bin/carddisp.pl?gene=ATG4C |
| OXA1L | OXA1L Mitochondrial Inner Membrane Protein | Protein Coding | 40 | GC14P022766 | 0.434577763 | https://www.genecards.org/cgi-bin/carddisp.pl?gene=OXA1L |
| TTF2 | Transcription Termination Factor 2 | Protein Coding | 40 | GC01P117060 | 0.434577763 | https://www.genecards.org/cgi-bin/carddisp.pl?gene=TTF2 |
| PLEK | Pleckstrin | Protein Coding | 40 | GC02P068365 | 0.434577763 | https://www.genecards.org/cgi-bin/carddisp.pl?gene=PLEK |
| PIGF | Phosphatidylinositol Glycan Anchor Biosynthesis Class F | Protein Coding | 40 | GC02M046580 | 0.434577763 | https://www.genecards.org/cgi-bin/carddisp.pl?gene=PIGF |
| SERPINA7 | Serpin Family A Member 7 | Protein Coding | 40 | GC0XM106032 | 0.434577763 | https://www.genecards.org/cgi-bin/carddisp.pl?gene=SERPINA7 |
| ATG4D | Autophagy Related 4D Cysteine Peptidase | Protein Coding | 38 | GC19P010543 | 0.434577763 | https://www.genecards.org/cgi-bin/carddisp.pl?gene=ATG4D |
| CCL22 | C-C Motif Chemokine Ligand 22 | Protein Coding | 38 | GC16P057359 | 0.434577763 | https://www.genecards.org/cgi-bin/carddisp.pl?gene=CCL22 |
| CEP68 | Centrosomal Protein 68 | Protein Coding | 38 | GC02P065056 | 0.434577763 | https://www.genecards.org/cgi-bin/carddisp.pl?gene=CEP68 |
| PEAR1 | Platelet Endothelial Aggregation Receptor 1 | Protein Coding | 37 | GC01P156893 | 0.434577763 | https://www.genecards.org/cgi-bin/carddisp.pl?gene=PEAR1 |
| AHSP | Alpha Hemoglobin Stabilizing Protein | Protein Coding | 36 | GC16P031527 | 0.434577763 | https://www.genecards.org/cgi-bin/carddisp.pl?gene=AHSP |
| ZBTB22 | Zinc Finger And BTB Domain Containing 22 | Protein Coding | 36 | GC06M046988 | 0.434577763 | https://www.genecards.org/cgi-bin/carddisp.pl?gene=ZBTB22 |
| FSIP1 | Fibrous Sheath Interacting Protein 1 | Protein Coding | 35 | GC15M039594 | 0.434577763 | https://www.genecards.org/cgi-bin/carddisp.pl?gene=FSIP1 |
| COL26A1 | Collagen Type XXVI Alpha 1 Chain | Protein Coding | 34 | GC07P101362 | 0.434577763 | https://www.genecards.org/cgi-bin/carddisp.pl?gene=COL26A1 |
| CGB5 | Chorionic Gonadotropin Subunit Beta 5 | Protein Coding | 33 | GC19P049043 | 0.434577763 | https://www.genecards.org/cgi-bin/carddisp.pl?gene=CGB5 |
| H2BS1 | H2B.S Histone 1 | Protein Coding | 23 | GC21P043567 | 0.434577763 | https://www.genecards.org/cgi-bin/carddisp.pl?gene=H2BS1 |
| TNXA | Tenascin XA (Pseudogene) | Pseudogene | 22 | GC06M046903 | 0.434577763 | https://www.genecards.org/cgi-bin/carddisp.pl?gene=TNXA |
| HLA-DPB2 | Major Histocompatibility Complex, Class II, DP Beta 2 (Pseudogene) | Pseudogene | 19 | GC06P055247 | 0.434577763 | https://www.genecards.org/cgi-bin/carddisp.pl?gene=HLA-DPB2 |
| IGES | Immunoglobulin E Concentration, Serum | Genetic Locus | 4 | GC05U990033 | 0.434577763 | https://www.genecards.org/cgi-bin/carddisp.pl?gene=IGES |
| STAT3 | Signal Transducer And Activator Of Transcription 3 | Protein Coding | 53 | GC17M042313 | 0.424227685 | https://www.genecards.org/cgi-bin/carddisp.pl?gene=STAT3 |
| GRIN2D | Glutamate Ionotropic Receptor NMDA Type Subunit 2D | Protein Coding | 50 | GC19P048394 | 0.424227685 | https://www.genecards.org/cgi-bin/carddisp.pl?gene=GRIN2D |
| DPP4 | Dipeptidyl Peptidase 4 | Protein Coding | 50 | GC02M161992 | 0.424227685 | https://www.genecards.org/cgi-bin/carddisp.pl?gene=DPP4 |
| SMARCA2 | SWI/SNF Related, Matrix Associated, Actin Dependent Regulator Of Chromatin, Subfamily A, Member 2 | Protein Coding | 50 | GC09P001980 | 0.424227685 | https://www.genecards.org/cgi-bin/carddisp.pl?gene=SMARCA2 |
| ADRB1 | Adrenoceptor Beta 1 | Protein Coding | 49 | GC10P114044 | 0.424227685 | https://www.genecards.org/cgi-bin/carddisp.pl?gene=ADRB1 |
| TFRC | Transferrin Receptor | Protein Coding | 49 | GC03M196027 | 0.424227685 | https://www.genecards.org/cgi-bin/carddisp.pl?gene=TFRC |
| TPO | Thyroid Peroxidase | Protein Coding | 49 | GC02P001374 | 0.424227685 | https://www.genecards.org/cgi-bin/carddisp.pl?gene=TPO |
| ECE1 | Endothelin Converting Enzyme 1 | Protein Coding | 48 | GC01M021217 | 0.424227685 | https://www.genecards.org/cgi-bin/carddisp.pl?gene=ECE1 |
| CHN1 | Chimerin 1 | Protein Coding | 48 | GC02M174799 | 0.424227685 | https://www.genecards.org/cgi-bin/carddisp.pl?gene=CHN1 |
| TSHR | Thyroid Stimulating Hormone Receptor | Protein Coding | 48 | GC14P080954 | 0.424227685 | https://www.genecards.org/cgi-bin/carddisp.pl?gene=TSHR |
| TTN | Titin | Protein Coding | 48 | GC02M178525 | 0.424227685 | https://www.genecards.org/cgi-bin/carddisp.pl?gene=TTN |
| TGM2 | Transglutaminase 2 | Protein Coding | 48 | GC20M038127 | 0.424227685 | https://www.genecards.org/cgi-bin/carddisp.pl?gene=TGM2 |
| GAD2 | Glutamate Decarboxylase 2 | Protein Coding | 47 | GC10P026216 | 0.424227685 | https://www.genecards.org/cgi-bin/carddisp.pl?gene=GAD2 |
| IL7R | Interleukin 7 Receptor | Protein Coding | 47 | GC05P035852 | 0.424227685 | https://www.genecards.org/cgi-bin/carddisp.pl?gene=IL7R |
| IRF1 | Interferon Regulatory Factor 1 | Protein Coding | 47 | GC05M132440 | 0.424227685 | https://www.genecards.org/cgi-bin/carddisp.pl?gene=IRF1 |
| GOT2 | Glutamic-Oxaloacetic Transaminase 2 | Protein Coding | 47 | GC16M058707 | 0.424227685 | https://www.genecards.org/cgi-bin/carddisp.pl?gene=GOT2 |
| AGPAT2 | 1-Acylglycerol-3-Phosphate O-Acyltransferase 2 | Protein Coding | 47 | GC09M136673 | 0.424227685 | https://www.genecards.org/cgi-bin/carddisp.pl?gene=AGPAT2 |
| ACHE | Acetylcholinesterase (Cartwright Blood Group) | Protein Coding | 47 | GC07M100889 | 0.424227685 | https://www.genecards.org/cgi-bin/carddisp.pl?gene=ACHE |
| ADAMTS13 | ADAM Metallopeptidase With Thrombospondin Type 1 Motif 13 | Protein Coding | 47 | GC09P133414 | 0.424227685 | https://www.genecards.org/cgi-bin/carddisp.pl?gene=ADAMTS13 |
| ANXA5 | Annexin A5 | Protein Coding | 47 | GC04M121667 | 0.424227685 | https://www.genecards.org/cgi-bin/carddisp.pl?gene=ANXA5 |
| VCAN | Versican | Protein Coding | 47 | GC05P083471 | 0.424227685 | https://www.genecards.org/cgi-bin/carddisp.pl?gene=VCAN |
| PKD2 | Polycystin 2, Transient Receptor Potential Cation Channel | Protein Coding | 47 | GC04P088007 | 0.424227685 | https://www.genecards.org/cgi-bin/carddisp.pl?gene=PKD2 |
| MUSK | Muscle Associated Receptor Tyrosine Kinase | Protein Coding | 47 | GC09P110668 | 0.424227685 | https://www.genecards.org/cgi-bin/carddisp.pl?gene=MUSK |
| PTH | Parathyroid Hormone | Protein Coding | 47 | GC11M013492 | 0.424227685 | https://www.genecards.org/cgi-bin/carddisp.pl?gene=PTH |
| PTPN2 | Protein Tyrosine Phosphatase Non-Receptor Type 2 | Protein Coding | 47 | GC18M019091 | 0.424227685 | https://www.genecards.org/cgi-bin/carddisp.pl?gene=PTPN2 |
| SH2B3 | SH2B Adaptor Protein 3 | Protein Coding | 47 | GC12P111405 | 0.424227685 | https://www.genecards.org/cgi-bin/carddisp.pl?gene=SH2B3 |
| ADRA2C | Adrenoceptor Alpha 2C | Protein Coding | 46 | GC04P003766 | 0.424227685 | https://www.genecards.org/cgi-bin/carddisp.pl?gene=ADRA2C |
| HEXA | Hexosaminidase Subunit Alpha | Protein Coding | 46 | GC15M072340 | 0.424227685 | https://www.genecards.org/cgi-bin/carddisp.pl?gene=HEXA |
| LMX1B | LIM Homeobox Transcription Factor 1 Beta | Protein Coding | 46 | GC09P126614 | 0.424227685 | https://www.genecards.org/cgi-bin/carddisp.pl?gene=LMX1B |
| CFH | Complement Factor H | Protein Coding | 46 | GC01P196621 | 0.424227685 | https://www.genecards.org/cgi-bin/carddisp.pl?gene=CFH |
| UBE2L3 | Ubiquitin Conjugating Enzyme E2 L3 | Protein Coding | 46 | GC22P021549 | 0.424227685 | https://www.genecards.org/cgi-bin/carddisp.pl?gene=UBE2L3 |
| PKD1 | Polycystin 1, Transient Receptor Potential Channel Interacting | Protein Coding | 46 | GC16M003057 | 0.424227685 | https://www.genecards.org/cgi-bin/carddisp.pl?gene=PKD1 |
| ADH4 | Alcohol Dehydrogenase 4 (Class II), Pi Polypeptide | Protein Coding | 45 | GC04M099123 | 0.424227685 | https://www.genecards.org/cgi-bin/carddisp.pl?gene=ADH4 |
| FUT2 | Fucosyltransferase 2 | Protein Coding | 45 | GC19P048695 | 0.424227685 | https://www.genecards.org/cgi-bin/carddisp.pl?gene=FUT2 |
| ITGAX | Integrin Subunit Alpha X | Protein Coding | 45 | GC16P032567 | 0.424227685 | https://www.genecards.org/cgi-bin/carddisp.pl?gene=ITGAX |
| HTR3B | 5-Hydroxytryptamine Receptor 3B | Protein Coding | 45 | GC11P113905 | 0.424227685 | https://www.genecards.org/cgi-bin/carddisp.pl?gene=HTR3B |
| CYP21A2 | Cytochrome P450 Family 21 Subfamily A Member 2 | Protein Coding | 45 | GC06P055225 | 0.424227685 | https://www.genecards.org/cgi-bin/carddisp.pl?gene=CYP21A2 |
| CXCR3 | C-X-C Motif Chemokine Receptor 3 | Protein Coding | 45 | GC0XM071615 | 0.424227685 | https://www.genecards.org/cgi-bin/carddisp.pl?gene=CXCR3 |
| DDOST | Dolichyl-Diphosphooligosaccharide--Protein Glycosyltransferase Non-Catalytic Subunit | Protein Coding | 45 | GC01M020651 | 0.424227685 | https://www.genecards.org/cgi-bin/carddisp.pl?gene=DDOST |
| AIRE | Autoimmune Regulator | Protein Coding | 45 | GC21P044285 | 0.424227685 | https://www.genecards.org/cgi-bin/carddisp.pl?gene=AIRE |
| B3GAT3 | Beta-1,3-Glucuronyltransferase 3 | Protein Coding | 45 | GC11M069250 | 0.424227685 | https://www.genecards.org/cgi-bin/carddisp.pl?gene=B3GAT3 |
| CIITA | Class II Major Histocompatibility Complex Transactivator | Protein Coding | 45 | GC16P010879 | 0.424227685 | https://www.genecards.org/cgi-bin/carddisp.pl?gene=CIITA |
| WRN | WRN RecQ Like Helicase | Protein Coding | 45 | GC08P031033 | 0.424227685 | https://www.genecards.org/cgi-bin/carddisp.pl?gene=WRN |
| MMP12 | Matrix Metallopeptidase 12 | Protein Coding | 45 | GC11M102862 | 0.424227685 | https://www.genecards.org/cgi-bin/carddisp.pl?gene=MMP12 |
| OCLN | Occludin | Protein Coding | 45 | GC05P069492 | 0.424227685 | https://www.genecards.org/cgi-bin/carddisp.pl?gene=OCLN |
| PTPRN | Protein Tyrosine Phosphatase Receptor Type N | Protein Coding | 45 | GC02M219289 | 0.424227685 | https://www.genecards.org/cgi-bin/carddisp.pl?gene=PTPRN |
| FARS2 | Phenylalanyl-TRNA Synthetase 2, Mitochondrial | Protein Coding | 44 | GC06P005261 | 0.424227685 | https://www.genecards.org/cgi-bin/carddisp.pl?gene=FARS2 |
| CD86 | CD86 Molecule | Protein Coding | 44 | GC03P122055 | 0.424227685 | https://www.genecards.org/cgi-bin/carddisp.pl?gene=CD86 |
| ATG16L1 | Autophagy Related 16 Like 1 | Protein Coding | 44 | GC02P233220 | 0.424227685 | https://www.genecards.org/cgi-bin/carddisp.pl?gene=ATG16L1 |
| CLOCK | Clock Circadian Regulator | Protein Coding | 44 | GC04M055427 | 0.424227685 | https://www.genecards.org/cgi-bin/carddisp.pl?gene=CLOCK |
| AHCYL1 | Adenosylhomocysteinase Like 1 | Protein Coding | 44 | GC01P109984 | 0.424227685 | https://www.genecards.org/cgi-bin/carddisp.pl?gene=AHCYL1 |
| MYO9B | Myosin IXB | Protein Coding | 44 | GC19P026629 | 0.424227685 | https://www.genecards.org/cgi-bin/carddisp.pl?gene=MYO9B |
| TSPO | Translocator Protein | Protein Coding | 44 | GC22P043151 | 0.424227685 | https://www.genecards.org/cgi-bin/carddisp.pl?gene=TSPO |
| TJP1 | Tight Junction Protein 1 | Protein Coding | 44 | GC15M029699 | 0.424227685 | https://www.genecards.org/cgi-bin/carddisp.pl?gene=TJP1 |
| PRSS3 | Serine Protease 3 | Protein Coding | 44 | GC09P033750 | 0.424227685 | https://www.genecards.org/cgi-bin/carddisp.pl?gene=PRSS3 |
| MUS81 | MUS81 Structure-Specific Endonuclease Subunit | Protein Coding | 44 | GC11P066258 | 0.424227685 | https://www.genecards.org/cgi-bin/carddisp.pl?gene=MUS81 |
| SEC63 | SEC63 Homolog, Protein Translocation Regulator | Protein Coding | 44 | GC06M107867 | 0.424227685 | https://www.genecards.org/cgi-bin/carddisp.pl?gene=SEC63 |
| MPZ | Myelin Protein Zero | Protein Coding | 44 | GC01M161304 | 0.424227685 | https://www.genecards.org/cgi-bin/carddisp.pl?gene=MPZ |
| NARS2 | Asparaginyl-TRNA Synthetase 2, Mitochondrial | Protein Coding | 44 | GC11M078435 | 0.424227685 | https://www.genecards.org/cgi-bin/carddisp.pl?gene=NARS2 |
| CTNND2 | Catenin Delta 2 | Protein Coding | 43 | GC05M010971 | 0.424227685 | https://www.genecards.org/cgi-bin/carddisp.pl?gene=CTNND2 |
| IL21 | Interleukin 21 | Protein Coding | 43 | GC04M122612 | 0.424227685 | https://www.genecards.org/cgi-bin/carddisp.pl?gene=IL21 |
| LCT | Lactase | Protein Coding | 43 | GC02M135787 | 0.424227685 | https://www.genecards.org/cgi-bin/carddisp.pl?gene=LCT |
| IL18R1 | Interleukin 18 Receptor 1 | Protein Coding | 43 | GC02P102311 | 0.424227685 | https://www.genecards.org/cgi-bin/carddisp.pl?gene=IL18R1 |
| FABP2 | Fatty Acid Binding Protein 2 | Protein Coding | 43 | GC04M119317 | 0.424227685 | https://www.genecards.org/cgi-bin/carddisp.pl?gene=FABP2 |
| BSCL2 | BSCL2 Lipid Droplet Biogenesis Associated, Seipin | Protein Coding | 43 | GC11M069260 | 0.424227685 | https://www.genecards.org/cgi-bin/carddisp.pl?gene=BSCL2 |
| EARS2 | Glutamyl-TRNA Synthetase 2, Mitochondrial | Protein Coding | 43 | GC16M023527 | 0.424227685 | https://www.genecards.org/cgi-bin/carddisp.pl?gene=EARS2 |
| TRIM21 | Tripartite Motif Containing 21 | Protein Coding | 43 | GC11M004384 | 0.424227685 | https://www.genecards.org/cgi-bin/carddisp.pl?gene=TRIM21 |
| TRPC1 | Transient Receptor Potential Cation Channel Subfamily C Member 1 | Protein Coding | 43 | GC03P142724 | 0.424227685 | https://www.genecards.org/cgi-bin/carddisp.pl?gene=TRPC1 |
| TG | Thyroglobulin | Protein Coding | 43 | GC08P132866 | 0.424227685 | https://www.genecards.org/cgi-bin/carddisp.pl?gene=TG |
| TGM3 | Transglutaminase 3 | Protein Coding | 43 | GC20P002296 | 0.424227685 | https://www.genecards.org/cgi-bin/carddisp.pl?gene=TGM3 |
| PROCR | Protein C Receptor | Protein Coding | 43 | GC20P035233 | 0.424227685 | https://www.genecards.org/cgi-bin/carddisp.pl?gene=PROCR |
| PROX1 | Prospero Homeobox 1 | Protein Coding | 43 | GC01P213983 | 0.424227685 | https://www.genecards.org/cgi-bin/carddisp.pl?gene=PROX1 |
| SLC30A8 | Solute Carrier Family 30 Member 8 | Protein Coding | 43 | GC08P116950 | 0.424227685 | https://www.genecards.org/cgi-bin/carddisp.pl?gene=SLC30A8 |
| SLC30A10 | Solute Carrier Family 30 Member 10 | Protein Coding | 43 | GC01M219685 | 0.424227685 | https://www.genecards.org/cgi-bin/carddisp.pl?gene=SLC30A10 |
| PARS2 | Prolyl-TRNA Synthetase 2, Mitochondrial | Protein Coding | 43 | GC01M054756 | 0.424227685 | https://www.genecards.org/cgi-bin/carddisp.pl?gene=PARS2 |
| PYY | Peptide YY | Protein Coding | 43 | GC17M043952 | 0.424227685 | https://www.genecards.org/cgi-bin/carddisp.pl?gene=PYY |
| NOX4 | NADPH Oxidase 4 | Protein Coding | 43 | GC11M089324 | 0.424227685 | https://www.genecards.org/cgi-bin/carddisp.pl?gene=NOX4 |
| ZMPSTE24 | Zinc Metallopeptidase STE24 | Protein Coding | 43 | GC01P040258 | 0.424227685 | https://www.genecards.org/cgi-bin/carddisp.pl?gene=ZMPSTE24 |
| FLVCR2 | FLVCR Heme Transporter 2 | Protein Coding | 42 | GC14P075578 | 0.424227685 | https://www.genecards.org/cgi-bin/carddisp.pl?gene=FLVCR2 |
| CLDN2 | Claudin 2 | Protein Coding | 42 | GC0XP106900 | 0.424227685 | https://www.genecards.org/cgi-bin/carddisp.pl?gene=CLDN2 |
| BACH2 | BTB Domain And CNC Homolog 2 | Protein Coding | 42 | GC06M089926 | 0.424227685 | https://www.genecards.org/cgi-bin/carddisp.pl?gene=BACH2 |
| CIDEC | Cell Death Inducing DFFA Like Effector C | Protein Coding | 42 | GC03M009866 | 0.424227685 | https://www.genecards.org/cgi-bin/carddisp.pl?gene=CIDEC |
| NPHS2 | NPHS2 Stomatin Family Member, Podocin | Protein Coding | 42 | GC01M179519 | 0.424227685 | https://www.genecards.org/cgi-bin/carddisp.pl?gene=NPHS2 |
| RAPGEF2 | Rap Guanine Nucleotide Exchange Factor 2 | Protein Coding | 42 | GC04P159106 | 0.424227685 | https://www.genecards.org/cgi-bin/carddisp.pl?gene=RAPGEF2 |
| KLRD1 | Killer Cell Lectin Like Receptor D1 | Protein Coding | 42 | GC12P010226 | 0.424227685 | https://www.genecards.org/cgi-bin/carddisp.pl?gene=KLRD1 |
| AGAP1 | ArfGAP With GTPase Domain, Ankyrin Repeat And PH Domain 1 | Protein Coding | 41 | GC02P235494 | 0.424227685 | https://www.genecards.org/cgi-bin/carddisp.pl?gene=AGAP1 |
| CCR9 | C-C Motif Chemokine Receptor 9 | Protein Coding | 41 | GC03P045903 | 0.424227685 | https://www.genecards.org/cgi-bin/carddisp.pl?gene=CCR9 |
| CCL4 | C-C Motif Chemokine Ligand 4 | Protein Coding | 41 | GC17P036103 | 0.424227685 | https://www.genecards.org/cgi-bin/carddisp.pl?gene=CCL4 |
| KLRC1 | Killer Cell Lectin Like Receptor C1 | Protein Coding | 41 | GC12M015692 | 0.424227685 | https://www.genecards.org/cgi-bin/carddisp.pl?gene=KLRC1 |
| HLA-DPA1 | Major Histocompatibility Complex, Class II, DP Alpha 1 | Protein Coding | 41 | GC06M033064 | 0.424227685 | https://www.genecards.org/cgi-bin/carddisp.pl?gene=HLA-DPA1 |
| HLA-DMA | Major Histocompatibility Complex, Class II, DM Alpha | Protein Coding | 41 | GC06M046951 | 0.424227685 | https://www.genecards.org/cgi-bin/carddisp.pl?gene=HLA-DMA |
| EPO | Erythropoietin | Protein Coding | 41 | GC07P100720 | 0.424227685 | https://www.genecards.org/cgi-bin/carddisp.pl?gene=EPO |
| GCG | Glucagon | Protein Coding | 41 | GC02M162142 | 0.424227685 | https://www.genecards.org/cgi-bin/carddisp.pl?gene=GCG |
| CARS2 | Cysteinyl-TRNA Synthetase 2, Mitochondrial | Protein Coding | 41 | GC13M110641 | 0.424227685 | https://www.genecards.org/cgi-bin/carddisp.pl?gene=CARS2 |
| PIBF1 | Progesterone Immunomodulatory Binding Factor 1 | Protein Coding | 41 | GC13P072782 | 0.424227685 | https://www.genecards.org/cgi-bin/carddisp.pl?gene=PIBF1 |
| NUP93 | Nucleoporin 93 | Protein Coding | 41 | GC16P056769 | 0.424227685 | https://www.genecards.org/cgi-bin/carddisp.pl?gene=NUP93 |
| PDLIM1 | PDZ And LIM Domain 1 | Protein Coding | 41 | GC10M095237 | 0.424227685 | https://www.genecards.org/cgi-bin/carddisp.pl?gene=PDLIM1 |
| RCOR1 | REST Corepressor 1 | Protein Coding | 41 | GC14P102592 | 0.424227685 | https://www.genecards.org/cgi-bin/carddisp.pl?gene=RCOR1 |
| ACMSD | Aminocarboxymuconate Semialdehyde Decarboxylase | Protein Coding | 40 | GC02P134838 | 0.424227685 | https://www.genecards.org/cgi-bin/carddisp.pl?gene=ACMSD |
| BAZ1A | Bromodomain Adjacent To Zinc Finger Domain 1A | Protein Coding | 40 | GC14M034752 | 0.424227685 | https://www.genecards.org/cgi-bin/carddisp.pl?gene=BAZ1A |
| CD7 | CD7 Molecule | Protein Coding | 40 | GC17M082314 | 0.424227685 | https://www.genecards.org/cgi-bin/carddisp.pl?gene=CD7 |
| ITGAE | Integrin Subunit Alpha E | Protein Coding | 40 | GC17M003722 | 0.424227685 | https://www.genecards.org/cgi-bin/carddisp.pl?gene=ITGAE |
| LALBA | Lactalbumin Alpha | Protein Coding | 40 | GC12M048567 | 0.424227685 | https://www.genecards.org/cgi-bin/carddisp.pl?gene=LALBA |
| HLTF | Helicase Like Transcription Factor | Protein Coding | 40 | GC03M149030 | 0.424227685 | https://www.genecards.org/cgi-bin/carddisp.pl?gene=HLTF |
| GAST | Gastrin | Protein Coding | 40 | GC17P041712 | 0.424227685 | https://www.genecards.org/cgi-bin/carddisp.pl?gene=GAST |
| UBASH3A | Ubiquitin Associated And SH3 Domain Containing A | Protein Coding | 40 | GC21P042403 | 0.424227685 | https://www.genecards.org/cgi-bin/carddisp.pl?gene=UBASH3A |
| TNRC6A | Trinucleotide Repeat Containing Adaptor 6A | Protein Coding | 40 | GC16P024611 | 0.424227685 | https://www.genecards.org/cgi-bin/carddisp.pl?gene=TNRC6A |
| VAMP5 | Vesicle Associated Membrane Protein 5 | Protein Coding | 40 | GC02P085589 | 0.424227685 | https://www.genecards.org/cgi-bin/carddisp.pl?gene=VAMP5 |
| TIPIN | TIMELESS Interacting Protein | Protein Coding | 40 | GC15M067420 | 0.424227685 | https://www.genecards.org/cgi-bin/carddisp.pl?gene=TIPIN |
| SMARCA1 | SWI/SNF Related, Matrix Associated, Actin Dependent Regulator Of Chromatin, Subfamily A, Member 1 | Protein Coding | 40 | GC0XM129447 | 0.424227685 | https://www.genecards.org/cgi-bin/carddisp.pl?gene=SMARCA1 |
| MYBBP1A | MYB Binding Protein 1a | Protein Coding | 40 | GC17M004538 | 0.424227685 | https://www.genecards.org/cgi-bin/carddisp.pl?gene=MYBBP1A |
| LMCD1 | LIM And Cysteine Rich Domains 1 | Protein Coding | 39 | GC03P008518 | 0.424227685 | https://www.genecards.org/cgi-bin/carddisp.pl?gene=LMCD1 |
| IL18RAP | Interleukin 18 Receptor Accessory Protein | Protein Coding | 39 | GC02P102418 | 0.424227685 | https://www.genecards.org/cgi-bin/carddisp.pl?gene=IL18RAP |
| DCHS1 | Dachsous Cadherin-Related 1 | Protein Coding | 39 | GC11M006621 | 0.424227685 | https://www.genecards.org/cgi-bin/carddisp.pl?gene=DCHS1 |
| ARMC9 | Armadillo Repeat Containing 9 | Protein Coding | 39 | GC02P231198 | 0.424227685 | https://www.genecards.org/cgi-bin/carddisp.pl?gene=ARMC9 |
| SCYL3 | SCY1 Like Pseudokinase 3 | Protein Coding | 39 | GC01M169821 | 0.424227685 | https://www.genecards.org/cgi-bin/carddisp.pl?gene=SCYL3 |
| RGS1 | Regulator Of G Protein Signaling 1 | Protein Coding | 39 | GC01P192575 | 0.424227685 | https://www.genecards.org/cgi-bin/carddisp.pl?gene=RGS1 |
| CELA3B | Chymotrypsin Like Elastase 3B | Protein Coding | 38 | GC01P021976 | 0.424227685 | https://www.genecards.org/cgi-bin/carddisp.pl?gene=CELA3B |
| ACKR2 | Atypical Chemokine Receptor 2 | Protein Coding | 38 | GC03P042804 | 0.424227685 | https://www.genecards.org/cgi-bin/carddisp.pl?gene=ACKR2 |
| NUP205 | Nucleoporin 205 | Protein Coding | 38 | GC07P135557 | 0.424227685 | https://www.genecards.org/cgi-bin/carddisp.pl?gene=NUP205 |
| TOR1B | Torsin Family 1 Member B | Protein Coding | 38 | GC09P129803 | 0.424227685 | https://www.genecards.org/cgi-bin/carddisp.pl?gene=TOR1B |
| TAGAP | T Cell Activation RhoGTPase Activating Protein | Protein Coding | 38 | GC06M159034 | 0.424227685 | https://www.genecards.org/cgi-bin/carddisp.pl?gene=TAGAP |
| TFB2M | Transcription Factor B2, Mitochondrial | Protein Coding | 38 | GC01M246540 | 0.424227685 | https://www.genecards.org/cgi-bin/carddisp.pl?gene=TFB2M |
| PKHD1 | PKHD1 Ciliary IPT Domain Containing Fibrocystin/Polyductin | Protein Coding | 38 | GC06M051588 | 0.424227685 | https://www.genecards.org/cgi-bin/carddisp.pl?gene=PKHD1 |
| KLRC2 | Killer Cell Lectin Like Receptor C2 | Protein Coding | 37 | GC12M015691 | 0.424227685 | https://www.genecards.org/cgi-bin/carddisp.pl?gene=KLRC2 |
| HLA-DQA2 | Major Histocompatibility Complex, Class II, DQ Alpha 2 | Protein Coding | 37 | GC06P032741 | 0.424227685 | https://www.genecards.org/cgi-bin/carddisp.pl?gene=HLA-DQA2 |
| CLEC16A | C-Type Lectin Domain Containing 16A | Protein Coding | 37 | GC16P010944 | 0.424227685 | https://www.genecards.org/cgi-bin/carddisp.pl?gene=CLEC16A |
| ADAD1 | Adenosine Deaminase Domain Containing 1 | Protein Coding | 37 | GC04P122378 | 0.424227685 | https://www.genecards.org/cgi-bin/carddisp.pl?gene=ADAD1 |
| AARS1 | Alanyl-TRNA Synthetase 1 | Protein Coding | 37 | GC16M070343 | 0.424227685 | https://www.genecards.org/cgi-bin/carddisp.pl?gene=AARS1 |
| MMEL1 | Membrane Metalloendopeptidase Like 1 | Protein Coding | 37 | GC01M002590 | 0.424227685 | https://www.genecards.org/cgi-bin/carddisp.pl?gene=MMEL1 |
| ZRANB3 | Zinc Finger RANBP2-Type Containing 3 | Protein Coding | 37 | GC02M135136 | 0.424227685 | https://www.genecards.org/cgi-bin/carddisp.pl?gene=ZRANB3 |
| SAMD12 | Sterile Alpha Motif Domain Containing 12 | Protein Coding | 37 | GC08M118131 | 0.424227685 | https://www.genecards.org/cgi-bin/carddisp.pl?gene=SAMD12 |
| SCHIP1 | Schwannomin Interacting Protein 1 | Protein Coding | 37 | GC03P159273 | 0.424227685 | https://www.genecards.org/cgi-bin/carddisp.pl?gene=SCHIP1 |
| YEATS2 | YEATS Domain Containing 2 | Protein Coding | 37 | GC03P183698 | 0.424227685 | https://www.genecards.org/cgi-bin/carddisp.pl?gene=YEATS2 |
| HERPUD2 | HERPUD Family Member 2 | Protein Coding | 36 | GC07M035632 | 0.424227685 | https://www.genecards.org/cgi-bin/carddisp.pl?gene=HERPUD2 |
| CLUAP1 | Clusterin Associated Protein 1 | Protein Coding | 36 | GC16P003500 | 0.424227685 | https://www.genecards.org/cgi-bin/carddisp.pl?gene=CLUAP1 |
| CELA1 | Chymotrypsin Like Elastase 1 | Protein Coding | 36 | GC12M051328 | 0.424227685 | https://www.genecards.org/cgi-bin/carddisp.pl?gene=CELA1 |
| PPP1R3F | Protein Phosphatase 1 Regulatory Subunit 3F | Protein Coding | 36 | GC0XP049269 | 0.424227685 | https://www.genecards.org/cgi-bin/carddisp.pl?gene=PPP1R3F |
| NEURL2 | Neuralized E3 Ubiquitin Protein Ligase 2 | Protein Coding | 36 | GC20M045888 | 0.424227685 | https://www.genecards.org/cgi-bin/carddisp.pl?gene=NEURL2 |
| NUDT10 | Nudix Hydrolase 10 | Protein Coding | 35 | GC0XP051332 | 0.424227685 | https://www.genecards.org/cgi-bin/carddisp.pl?gene=NUDT10 |
| PUS10 | Pseudouridine Synthase 10 | Protein Coding | 35 | GC02M060940 | 0.424227685 | https://www.genecards.org/cgi-bin/carddisp.pl?gene=PUS10 |
| FAM149B1 | Family With Sequence Similarity 149 Member B1 | Protein Coding | 34 | GC10P073168 | 0.424227685 | https://www.genecards.org/cgi-bin/carddisp.pl?gene=FAM149B1 |
| ASTN1 | Astrotactin 1 | Protein Coding | 34 | GC01M176857 | 0.424227685 | https://www.genecards.org/cgi-bin/carddisp.pl?gene=ASTN1 |
| KIAA1109 | KIAA1109 | Protein Coding | 34 | GC04P122152 | 0.424227685 | https://www.genecards.org/cgi-bin/carddisp.pl?gene=KIAA1109 |
| ANKRD55 | Ankyrin Repeat Domain 55 | Protein Coding | 33 | GC05M056099 | 0.424227685 | https://www.genecards.org/cgi-bin/carddisp.pl?gene=ANKRD55 |
| METTL18 | Methyltransferase Like 18 | Protein Coding | 33 | GC01M169792 | 0.424227685 | https://www.genecards.org/cgi-bin/carddisp.pl?gene=METTL18 |
| PRIMPOL | Primase And DNA Directed Polymerase | Protein Coding | 33 | GC04P184649 | 0.424227685 | https://www.genecards.org/cgi-bin/carddisp.pl?gene=PRIMPOL |
| YDJC | YdjC Chitooligosaccharide Deacetylase Homolog | Protein Coding | 33 | GC22M021628 | 0.424227685 | https://www.genecards.org/cgi-bin/carddisp.pl?gene=YDJC |
| SFTA3 | Surfactant Associated 3 | RNA Gene | 32 | GC14M036474 | 0.424227685 | https://www.genecards.org/cgi-bin/carddisp.pl?gene=SFTA3 |
| DNAJC22 | DnaJ Heat Shock Protein Family (Hsp40) Member C22 | Protein Coding | 31 | GC12P049346 | 0.424227685 | https://www.genecards.org/cgi-bin/carddisp.pl?gene=DNAJC22 |
| MTARC2 | Mitochondrial Amidoxime Reducing Component 2 | Protein Coding | 30 | GC01P220748 | 0.424227685 | https://www.genecards.org/cgi-bin/carddisp.pl?gene=MTARC2 |
| KATNIP | Katanin Interacting Protein | Protein Coding | 27 | GC16P027551 | 0.424227685 | https://www.genecards.org/cgi-bin/carddisp.pl?gene=KATNIP |
| RETREG3 | Reticulophagy Regulator Family Member 3 | Protein Coding | 27 | GC17M042581 | 0.424227685 | https://www.genecards.org/cgi-bin/carddisp.pl?gene=RETREG3 |
| ELAPOR2 | Endosome-Lysosome Associated Apoptosis And Autophagy Regulator Family Member 2 | Protein Coding | 26 | GC07M086877 | 0.424227685 | https://www.genecards.org/cgi-bin/carddisp.pl?gene=ELAPOR2 |
| RADX | RPA1 Related Single Stranded DNA Binding Protein, X-Linked | Protein Coding | 26 | GC0XP106612 | 0.424227685 | https://www.genecards.org/cgi-bin/carddisp.pl?gene=RADX |
| MIR192 | MicroRNA 192 | RNA Gene | 22 | GC11M064891 | 0.424227685 | https://www.genecards.org/cgi-bin/carddisp.pl?gene=MIR192 |
| MIR29B1 | MicroRNA 29b-1 | RNA Gene | 20 | GC07M130877 | 0.424227685 | https://www.genecards.org/cgi-bin/carddisp.pl?gene=MIR29B1 |
| MIR423 | MicroRNA 423 | RNA Gene | 18 | GC17P030117 | 0.424227685 | https://www.genecards.org/cgi-bin/carddisp.pl?gene=MIR423 |
| MIR379 | MicroRNA 379 | RNA Gene | 16 | GC14P106318 | 0.424227685 | https://www.genecards.org/cgi-bin/carddisp.pl?gene=MIR379 |
| TTN-AS1 | TTN Antisense RNA 1 | RNA Gene | 16 | GC02P178521 | 0.424227685 | https://www.genecards.org/cgi-bin/carddisp.pl?gene=TTN-AS1 |
| MIR3174 | MicroRNA 3174 | RNA Gene | 15 | GC15P090006 | 0.424227685 | https://www.genecards.org/cgi-bin/carddisp.pl?gene=MIR3174 |
| MIR4298 | MicroRNA 4298 | RNA Gene | 12 | GC11M001880 | 0.424227685 | https://www.genecards.org/cgi-bin/carddisp.pl?gene=MIR4298 |
| LINC01193 | Long Intergenic Non-Protein Coding RNA 1193 | RNA Gene | 12 | GC15P021472 | 0.424227685 | https://www.genecards.org/cgi-bin/carddisp.pl?gene=LINC01193 |
| MIR3671 | MicroRNA 3671 | RNA Gene | 10 | GC01M065057 | 0.424227685 | https://www.genecards.org/cgi-bin/carddisp.pl?gene=MIR3671 |
| MIR1193 | MicroRNA 1193 | RNA Gene | 9 | GC14P106301 | 0.424227685 | https://www.genecards.org/cgi-bin/carddisp.pl?gene=MIR1193 |
| MIR6766 | MicroRNA 6766 | RNA Gene | 8 | GC15M089326 | 0.424227685 | https://www.genecards.org/cgi-bin/carddisp.pl?gene=MIR6766 |
| ENSG00000265031 |  | RNA Gene | 4 | GC17M064426 | 0.424227685 | https://www.genecards.org/cgi-bin/carddisp.pl?gene=ENSG00000265031 |
| HDAC3 | Histone Deacetylase 3 | Protein Coding | 50 | GC05M141583 | 0.39950037 | https://www.genecards.org/cgi-bin/carddisp.pl?gene=HDAC3 |
| SDHB | Succinate Dehydrogenase Complex Iron Sulfur Subunit B | Protein Coding | 48 | GC01M017238 | 0.39950037 | https://www.genecards.org/cgi-bin/carddisp.pl?gene=SDHB |
| POU2F1 | POU Class 2 Homeobox 1 | Protein Coding | 45 | GC01P167190 | 0.39950037 | https://www.genecards.org/cgi-bin/carddisp.pl?gene=POU2F1 |
| TRAPPC4 | Trafficking Protein Particle Complex Subunit 4 | Protein Coding | 41 | GC11P119018 | 0.39950037 | https://www.genecards.org/cgi-bin/carddisp.pl?gene=TRAPPC4 |
| GAA | Alpha Glucosidase | Protein Coding | 48 | GC17P080101 | 0.385122657 | https://www.genecards.org/cgi-bin/carddisp.pl?gene=GAA |
| IGF1R | Insulin Like Growth Factor 1 Receptor | Protein Coding | 55 | GC15P098648 | 0.346795946 | https://www.genecards.org/cgi-bin/carddisp.pl?gene=IGF1R |
| FGFR1 | Fibroblast Growth Factor Receptor 1 | Protein Coding | 55 | GC08M038400 | 0.346795946 | https://www.genecards.org/cgi-bin/carddisp.pl?gene=FGFR1 |
| ATM | ATM Serine/Threonine Kinase | Protein Coding | 54 | GC11P108222 | 0.346795946 | https://www.genecards.org/cgi-bin/carddisp.pl?gene=ATM |
| EZH2 | Enhancer Of Zeste 2 Polycomb Repressive Complex 2 Subunit | Protein Coding | 54 | GC07M148807 | 0.346795946 | https://www.genecards.org/cgi-bin/carddisp.pl?gene=EZH2 |
| AKT1 | AKT Serine/Threonine Kinase 1 | Protein Coding | 54 | GC14M104769 | 0.346795946 | https://www.genecards.org/cgi-bin/carddisp.pl?gene=AKT1 |
| BTK | Bruton Tyrosine Kinase | Protein Coding | 54 | GC0XM101349 | 0.346795946 | https://www.genecards.org/cgi-bin/carddisp.pl?gene=BTK |
| CHEK2 | Checkpoint Kinase 2 | Protein Coding | 54 | GC22M028687 | 0.346795946 | https://www.genecards.org/cgi-bin/carddisp.pl?gene=CHEK2 |
| STAT1 | Signal Transducer And Activator Of Transcription 1 | Protein Coding | 54 | GC02M190908 | 0.346795946 | https://www.genecards.org/cgi-bin/carddisp.pl?gene=STAT1 |
| PPP3CA | Protein Phosphatase 3 Catalytic Subunit Alpha | Protein Coding | 54 | GC04M101024 | 0.346795946 | https://www.genecards.org/cgi-bin/carddisp.pl?gene=PPP3CA |
| IDH1 | Isocitrate Dehydrogenase (NADP(+)) 1 | Protein Coding | 53 | GC02M208236 | 0.346795946 | https://www.genecards.org/cgi-bin/carddisp.pl?gene=IDH1 |
| CDH2 | Cadherin 2 | Protein Coding | 53 | GC18M027950 | 0.346795946 | https://www.genecards.org/cgi-bin/carddisp.pl?gene=CDH2 |
| PTEN | Phosphatase And Tensin Homolog | Protein Coding | 53 | GC10P087863 | 0.346795946 | https://www.genecards.org/cgi-bin/carddisp.pl?gene=PTEN |
| MDM2 | MDM2 Proto-Oncogene | Protein Coding | 53 | GC12P068808 | 0.346795946 | https://www.genecards.org/cgi-bin/carddisp.pl?gene=MDM2 |
| MYLK | Myosin Light Chain Kinase | Protein Coding | 53 | GC03M123610 | 0.346795946 | https://www.genecards.org/cgi-bin/carddisp.pl?gene=MYLK |
| HRAS | HRas Proto-Oncogene, GTPase | Protein Coding | 52 | GC11M001078 | 0.346795946 | https://www.genecards.org/cgi-bin/carddisp.pl?gene=HRAS |
| NFKB2 | Nuclear Factor Kappa B Subunit 2 | Protein Coding | 52 | GC10P102394 | 0.346795946 | https://www.genecards.org/cgi-bin/carddisp.pl?gene=NFKB2 |
| CDKN2A | Cyclin Dependent Kinase Inhibitor 2A | Protein Coding | 52 | GC09M021967 | 0.346795946 | https://www.genecards.org/cgi-bin/carddisp.pl?gene=CDKN2A |
| TERT | Telomerase Reverse Transcriptase | Protein Coding | 52 | GC05M001253 | 0.346795946 | https://www.genecards.org/cgi-bin/carddisp.pl?gene=TERT |
| ZAP70 | Zeta Chain Of T Cell Receptor Associated Protein Kinase 70 | Protein Coding | 52 | GC02P097696 | 0.346795946 | https://www.genecards.org/cgi-bin/carddisp.pl?gene=ZAP70 |
| PRKAR1A | Protein Kinase CAMP-Dependent Type I Regulatory Subunit Alpha | Protein Coding | 52 | GC17P068545 | 0.346795946 | https://www.genecards.org/cgi-bin/carddisp.pl?gene=PRKAR1A |
| DNM1 | Dynamin 1 | Protein Coding | 51 | GC09P128191 | 0.346795946 | https://www.genecards.org/cgi-bin/carddisp.pl?gene=DNM1 |
| ITGA2B | Integrin Subunit Alpha 2b | Protein Coding | 51 | GC17M044442 | 0.346795946 | https://www.genecards.org/cgi-bin/carddisp.pl?gene=ITGA2B |
| CBL | Cbl Proto-Oncogene | Protein Coding | 51 | GC11P119206 | 0.346795946 | https://www.genecards.org/cgi-bin/carddisp.pl?gene=CBL |
| BRCA1 | BRCA1 DNA Repair Associated | Protein Coding | 51 | GC17M043044 | 0.346795946 | https://www.genecards.org/cgi-bin/carddisp.pl?gene=BRCA1 |
| BLK | BLK Proto-Oncogene, Src Family Tyrosine Kinase | Protein Coding | 51 | GC08P011486 | 0.346795946 | https://www.genecards.org/cgi-bin/carddisp.pl?gene=BLK |
| AHCY | Adenosylhomocysteinase | Protein Coding | 51 | GC20M034341 | 0.346795946 | https://www.genecards.org/cgi-bin/carddisp.pl?gene=AHCY |
| EP300 | E1A Binding Protein P300 | Protein Coding | 51 | GC22P041091 | 0.346795946 | https://www.genecards.org/cgi-bin/carddisp.pl?gene=EP300 |
| VIM | Vimentin | Protein Coding | 51 | GC10P017227 | 0.346795946 | https://www.genecards.org/cgi-bin/carddisp.pl?gene=VIM |
| PRKD1 | Protein Kinase D1 | Protein Coding | 51 | GC14M029576 | 0.346795946 | https://www.genecards.org/cgi-bin/carddisp.pl?gene=PRKD1 |
| RARA | Retinoic Acid Receptor Alpha | Protein Coding | 51 | GC17P040309 | 0.346795946 | https://www.genecards.org/cgi-bin/carddisp.pl?gene=RARA |
| GABRB3 | Gamma-Aminobutyric Acid Type A Receptor Subunit Beta3 | Protein Coding | 50 | GC15M026543 | 0.346795946 | https://www.genecards.org/cgi-bin/carddisp.pl?gene=GABRB3 |
| LEPR | Leptin Receptor | Protein Coding | 50 | GC01P065421 | 0.346795946 | https://www.genecards.org/cgi-bin/carddisp.pl?gene=LEPR |
| MGMT | O-6-Methylguanine-DNA Methyltransferase | Protein Coding | 50 | GC10P129467 | 0.346795946 | https://www.genecards.org/cgi-bin/carddisp.pl?gene=MGMT |
| IL6R | Interleukin 6 Receptor | Protein Coding | 50 | GC01P154405 | 0.346795946 | https://www.genecards.org/cgi-bin/carddisp.pl?gene=IL6R |
| CASP2 | Caspase 2 | Protein Coding | 50 | GC07P145813 | 0.346795946 | https://www.genecards.org/cgi-bin/carddisp.pl?gene=CASP2 |
| FASN | Fatty Acid Synthase | Protein Coding | 50 | GC17M082078 | 0.346795946 | https://www.genecards.org/cgi-bin/carddisp.pl?gene=FASN |
| AIFM1 | Apoptosis Inducing Factor Mitochondria Associated 1 | Protein Coding | 50 | GC0XM130129 | 0.346795946 | https://www.genecards.org/cgi-bin/carddisp.pl?gene=AIFM1 |
| ACTB | Actin Beta | Protein Coding | 50 | GC07M005527 | 0.346795946 | https://www.genecards.org/cgi-bin/carddisp.pl?gene=ACTB |
| ACTG1 | Actin Gamma 1 | Protein Coding | 50 | GC17M081509 | 0.346795946 | https://www.genecards.org/cgi-bin/carddisp.pl?gene=ACTG1 |
| CASP7 | Caspase 7 | Protein Coding | 50 | GC10P113679 | 0.346795946 | https://www.genecards.org/cgi-bin/carddisp.pl?gene=CASP7 |
| BUB1 | BUB1 Mitotic Checkpoint Serine/Threonine Kinase | Protein Coding | 50 | GC02M110637 | 0.346795946 | https://www.genecards.org/cgi-bin/carddisp.pl?gene=BUB1 |
| BUB1B | BUB1 Mitotic Checkpoint Serine/Threonine Kinase B | Protein Coding | 50 | GC15P040161 | 0.346795946 | https://www.genecards.org/cgi-bin/carddisp.pl?gene=BUB1B |
| ARG1 | Arginase 1 | Protein Coding | 50 | GC06P131473 | 0.346795946 | https://www.genecards.org/cgi-bin/carddisp.pl?gene=ARG1 |
| DNM2 | Dynamin 2 | Protein Coding | 50 | GC19P010718 | 0.346795946 | https://www.genecards.org/cgi-bin/carddisp.pl?gene=DNM2 |
| EIF2AK3 | Eukaryotic Translation Initiation Factor 2 Alpha Kinase 3 | Protein Coding | 50 | GC02M088556 | 0.346795946 | https://www.genecards.org/cgi-bin/carddisp.pl?gene=EIF2AK3 |
| WNT5A | Wnt Family Member 5A | Protein Coding | 50 | GC03M055465 | 0.346795946 | https://www.genecards.org/cgi-bin/carddisp.pl?gene=WNT5A |
| WNT7A | Wnt Family Member 7A | Protein Coding | 50 | GC03M016916 | 0.346795946 | https://www.genecards.org/cgi-bin/carddisp.pl?gene=WNT7A |
| STK11 | Serine/Threonine Kinase 11 | Protein Coding | 50 | GC19P001177 | 0.346795946 | https://www.genecards.org/cgi-bin/carddisp.pl?gene=STK11 |
| POLE | DNA Polymerase Epsilon, Catalytic Subunit | Protein Coding | 50 | GC12M132637 | 0.346795946 | https://www.genecards.org/cgi-bin/carddisp.pl?gene=POLE |
| PSEN2 | Presenilin 2 | Protein Coding | 50 | GC01P226870 | 0.346795946 | https://www.genecards.org/cgi-bin/carddisp.pl?gene=PSEN2 |
| SIRT1 | Sirtuin 1 | Protein Coding | 50 | GC10P067884 | 0.346795946 | https://www.genecards.org/cgi-bin/carddisp.pl?gene=SIRT1 |
| MYH9 | Myosin Heavy Chain 9 | Protein Coding | 50 | GC22M036281 | 0.346795946 | https://www.genecards.org/cgi-bin/carddisp.pl?gene=MYH9 |
| PBX1 | PBX Homeobox 1 | Protein Coding | 50 | GC01P164524 | 0.346795946 | https://www.genecards.org/cgi-bin/carddisp.pl?gene=PBX1 |
| RAD50 | RAD50 Double Strand Break Repair Protein | Protein Coding | 50 | GC05P132556 | 0.346795946 | https://www.genecards.org/cgi-bin/carddisp.pl?gene=RAD50 |
| SHH | Sonic Hedgehog Signaling Molecule | Protein Coding | 50 | GC07M155799 | 0.346795946 | https://www.genecards.org/cgi-bin/carddisp.pl?gene=SHH |
| NRAS | NRAS Proto-Oncogene, GTPase | Protein Coding | 50 | GC01M114704 | 0.346795946 | https://www.genecards.org/cgi-bin/carddisp.pl?gene=NRAS |
| COL2A1 | Collagen Type II Alpha 1 Chain | Protein Coding | 49 | GC12M047972 | 0.346795946 | https://www.genecards.org/cgi-bin/carddisp.pl?gene=COL2A1 |
| AXIN2 | Axin 2 | Protein Coding | 49 | GC17M065528 | 0.346795946 | https://www.genecards.org/cgi-bin/carddisp.pl?gene=AXIN2 |
| IDE | Insulin Degrading Enzyme | Protein Coding | 49 | GC10M092451 | 0.346795946 | https://www.genecards.org/cgi-bin/carddisp.pl?gene=IDE |
| GSN | Gelsolin | Protein Coding | 49 | GC09P121201 | 0.346795946 | https://www.genecards.org/cgi-bin/carddisp.pl?gene=GSN |
| ALDOA | Aldolase, Fructose-Bisphosphate A | Protein Coding | 49 | GC16P030064 | 0.346795946 | https://www.genecards.org/cgi-bin/carddisp.pl?gene=ALDOA |
| ACE2 | Angiotensin Converting Enzyme 2 | Protein Coding | 49 | GC0XM015494 | 0.346795946 | https://www.genecards.org/cgi-bin/carddisp.pl?gene=ACE2 |
| BAX | BCL2 Associated X, Apoptosis Regulator | Protein Coding | 49 | GC19P048954 | 0.346795946 | https://www.genecards.org/cgi-bin/carddisp.pl?gene=BAX |
| SLC12A6 | Solute Carrier Family 12 Member 6 | Protein Coding | 49 | GC15M034229 | 0.346795946 | https://www.genecards.org/cgi-bin/carddisp.pl?gene=SLC12A6 |
| RAB7A | RAB7A, Member RAS Oncogene Family | Protein Coding | 49 | GC03P131442 | 0.346795946 | https://www.genecards.org/cgi-bin/carddisp.pl?gene=RAB7A |
| TOP1 | DNA Topoisomerase I | Protein Coding | 49 | GC20P041028 | 0.346795946 | https://www.genecards.org/cgi-bin/carddisp.pl?gene=TOP1 |
| ARF1 | ADP Ribosylation Factor 1 | Protein Coding | 48 | GC01P228082 | 0.346795946 | https://www.genecards.org/cgi-bin/carddisp.pl?gene=ARF1 |
| FCGR2B | Fc Fragment Of IgG Receptor IIb | Protein Coding | 48 | GC01P161663 | 0.346795946 | https://www.genecards.org/cgi-bin/carddisp.pl?gene=FCGR2B |
| CP | Ceruloplasmin | Protein Coding | 48 | GC03M149162 | 0.346795946 | https://www.genecards.org/cgi-bin/carddisp.pl?gene=CP |
| LBR | Lamin B Receptor | Protein Coding | 48 | GC01M225401 | 0.346795946 | https://www.genecards.org/cgi-bin/carddisp.pl?gene=LBR |
| LIG4 | DNA Ligase 4 | Protein Coding | 48 | GC13M108207 | 0.346795946 | https://www.genecards.org/cgi-bin/carddisp.pl?gene=LIG4 |
| MFN2 | Mitofusin 2 | Protein Coding | 48 | GC01P011980 | 0.346795946 | https://www.genecards.org/cgi-bin/carddisp.pl?gene=MFN2 |
| HSP90AA1 | Heat Shock Protein 90 Alpha Family Class A Member 1 | Protein Coding | 48 | GC14M102080 | 0.346795946 | https://www.genecards.org/cgi-bin/carddisp.pl?gene=HSP90AA1 |
| HSPA9 | Heat Shock Protein Family A (Hsp70) Member 9 | Protein Coding | 48 | GC05M138554 | 0.346795946 | https://www.genecards.org/cgi-bin/carddisp.pl?gene=HSPA9 |
| NFATC1 | Nuclear Factor Of Activated T Cells 1 | Protein Coding | 48 | GC18P079395 | 0.346795946 | https://www.genecards.org/cgi-bin/carddisp.pl?gene=NFATC1 |
| GHSR | Growth Hormone Secretagogue Receptor | Protein Coding | 48 | GC03M172443 | 0.346795946 | https://www.genecards.org/cgi-bin/carddisp.pl?gene=GHSR |
| DES | Desmin | Protein Coding | 48 | GC02P219418 | 0.346795946 | https://www.genecards.org/cgi-bin/carddisp.pl?gene=DES |
| ESRRA | Estrogen Related Receptor Alpha | Protein Coding | 48 | GC11P064305 | 0.346795946 | https://www.genecards.org/cgi-bin/carddisp.pl?gene=ESRRA |
| APRT | Adenine Phosphoribosyltransferase | Protein Coding | 48 | GC16M088810 | 0.346795946 | https://www.genecards.org/cgi-bin/carddisp.pl?gene=APRT |
| CDKN2B | Cyclin Dependent Kinase Inhibitor 2B | Protein Coding | 48 | GC09M022002 | 0.346795946 | https://www.genecards.org/cgi-bin/carddisp.pl?gene=CDKN2B |
| DLG4 | Discs Large MAGUK Scaffold Protein 4 | Protein Coding | 48 | GC17M007189 | 0.346795946 | https://www.genecards.org/cgi-bin/carddisp.pl?gene=DLG4 |
| CXCR2 | C-X-C Motif Chemokine Receptor 2 | Protein Coding | 48 | GC02P218125 | 0.346795946 | https://www.genecards.org/cgi-bin/carddisp.pl?gene=CXCR2 |
| ENO1 | Enolase 1 | Protein Coding | 48 | GC01M008861 | 0.346795946 | https://www.genecards.org/cgi-bin/carddisp.pl?gene=ENO1 |
| EPHX1 | Epoxide Hydrolase 1 | Protein Coding | 48 | GC01P225810 | 0.346795946 | https://www.genecards.org/cgi-bin/carddisp.pl?gene=EPHX1 |
| TUBB1 | Tubulin Beta 1 Class VI | Protein Coding | 48 | GC20P059020 | 0.346795946 | https://www.genecards.org/cgi-bin/carddisp.pl?gene=TUBB1 |
| NBN | Nibrin | Protein Coding | 48 | GC08M089933 | 0.346795946 | https://www.genecards.org/cgi-bin/carddisp.pl?gene=NBN |
| WNT10B | Wnt Family Member 10B | Protein Coding | 48 | GC12M048965 | 0.346795946 | https://www.genecards.org/cgi-bin/carddisp.pl?gene=WNT10B |
| WNT3A | Wnt Family Member 3A | Protein Coding | 48 | GC01P228466 | 0.346795946 | https://www.genecards.org/cgi-bin/carddisp.pl?gene=WNT3A |
| WNT4 | Wnt Family Member 4 | Protein Coding | 48 | GC01M022190 | 0.346795946 | https://www.genecards.org/cgi-bin/carddisp.pl?gene=WNT4 |
| TFAP2A | Transcription Factor AP-2 Alpha | Protein Coding | 48 | GC06M010393 | 0.346795946 | https://www.genecards.org/cgi-bin/carddisp.pl?gene=TFAP2A |
| SSTR2 | Somatostatin Receptor 2 | Protein Coding | 48 | GC17P073165 | 0.346795946 | https://www.genecards.org/cgi-bin/carddisp.pl?gene=SSTR2 |
| STAT2 | Signal Transducer And Activator Of Transcription 2 | Protein Coding | 48 | GC12M056341 | 0.346795946 | https://www.genecards.org/cgi-bin/carddisp.pl?gene=STAT2 |
| PINK1 | PTEN Induced Kinase 1 | Protein Coding | 48 | GC01P020634 | 0.346795946 | https://www.genecards.org/cgi-bin/carddisp.pl?gene=PINK1 |
| PDE6A | Phosphodiesterase 6A | Protein Coding | 48 | GC05M149857 | 0.346795946 | https://www.genecards.org/cgi-bin/carddisp.pl?gene=PDE6A |
| PTPN12 | Protein Tyrosine Phosphatase Non-Receptor Type 12 | Protein Coding | 48 | GC07P077537 | 0.346795946 | https://www.genecards.org/cgi-bin/carddisp.pl?gene=PTPN12 |
| SLC3A1 | Solute Carrier Family 3 Member 1 | Protein Coding | 48 | GC02P044275 | 0.346795946 | https://www.genecards.org/cgi-bin/carddisp.pl?gene=SLC3A1 |
| OAT | Ornithine Aminotransferase | Protein Coding | 48 | GC10M124397 | 0.346795946 | https://www.genecards.org/cgi-bin/carddisp.pl?gene=OAT |
| PAX6 | Paired Box 6 | Protein Coding | 48 | GC11M031784 | 0.346795946 | https://www.genecards.org/cgi-bin/carddisp.pl?gene=PAX6 |
| RRM1 | Ribonucleotide Reductase Catalytic Subunit M1 | Protein Coding | 48 | GC11P004115 | 0.346795946 | https://www.genecards.org/cgi-bin/carddisp.pl?gene=RRM1 |
| PTPRJ | Protein Tyrosine Phosphatase Receptor Type J | Protein Coding | 48 | GC11P048002 | 0.346795946 | https://www.genecards.org/cgi-bin/carddisp.pl?gene=PTPRJ |
| SH2D1A | SH2 Domain Containing 1A | Protein Coding | 48 | GC0XP124227 | 0.346795946 | https://www.genecards.org/cgi-bin/carddisp.pl?gene=SH2D1A |
| SIRT3 | Sirtuin 3 | Protein Coding | 48 | GC11M000215 | 0.346795946 | https://www.genecards.org/cgi-bin/carddisp.pl?gene=SIRT3 |
| NRXN1 | Neurexin 1 | Protein Coding | 48 | GC02M049918 | 0.346795946 | https://www.genecards.org/cgi-bin/carddisp.pl?gene=NRXN1 |
| KIF11 | Kinesin Family Member 11 | Protein Coding | 48 | GC10P092574 | 0.346795946 | https://www.genecards.org/cgi-bin/carddisp.pl?gene=KIF11 |
| ARAF | A-Raf Proto-Oncogene, Serine/Threonine Kinase | Protein Coding | 47 | GC0XP047562 | 0.346795946 | https://www.genecards.org/cgi-bin/carddisp.pl?gene=ARAF |
| ABCC6 | ATP Binding Cassette Subfamily C Member 6 | Protein Coding | 47 | GC16M016148 | 0.346795946 | https://www.genecards.org/cgi-bin/carddisp.pl?gene=ABCC6 |
| ABCC8 | ATP Binding Cassette Subfamily C Member 8 | Protein Coding | 47 | GC11M017392 | 0.346795946 | https://www.genecards.org/cgi-bin/carddisp.pl?gene=ABCC8 |
| CHRNA2 | Cholinergic Receptor Nicotinic Alpha 2 Subunit | Protein Coding | 47 | GC08M027459 | 0.346795946 | https://www.genecards.org/cgi-bin/carddisp.pl?gene=CHRNA2 |
| ATF4 | Activating Transcription Factor 4 | Protein Coding | 47 | GC22P039547 | 0.346795946 | https://www.genecards.org/cgi-bin/carddisp.pl?gene=ATF4 |
| IRF7 | Interferon Regulatory Factor 7 | Protein Coding | 47 | GC11M000612 | 0.346795946 | https://www.genecards.org/cgi-bin/carddisp.pl?gene=IRF7 |
| IRF8 | Interferon Regulatory Factor 8 | Protein Coding | 47 | GC16P085898 | 0.346795946 | https://www.genecards.org/cgi-bin/carddisp.pl?gene=IRF8 |
| ILK | Integrin Linked Kinase | Protein Coding | 47 | GC11P006604 | 0.346795946 | https://www.genecards.org/cgi-bin/carddisp.pl?gene=ILK |
| LAT | Linker For Activation Of T Cells | Protein Coding | 47 | GC16P032276 | 0.346795946 | https://www.genecards.org/cgi-bin/carddisp.pl?gene=LAT |
| LRP2 | LDL Receptor Related Protein 2 | Protein Coding | 47 | GC02M169127 | 0.346795946 | https://www.genecards.org/cgi-bin/carddisp.pl?gene=LRP2 |
| MASP2 | MBL Associated Serine Protease 2 | Protein Coding | 47 | GC01M011026 | 0.346795946 | https://www.genecards.org/cgi-bin/carddisp.pl?gene=MASP2 |
| CAMK4 | Calcium/Calmodulin Dependent Protein Kinase IV | Protein Coding | 47 | GC05P111223 | 0.346795946 | https://www.genecards.org/cgi-bin/carddisp.pl?gene=CAMK4 |
| GATA1 | GATA Binding Protein 1 | Protein Coding | 47 | GC0XP048786 | 0.346795946 | https://www.genecards.org/cgi-bin/carddisp.pl?gene=GATA1 |
| FKBP1A | FKBP Prolyl Isomerase 1A | Protein Coding | 47 | GC20M001369 | 0.346795946 | https://www.genecards.org/cgi-bin/carddisp.pl?gene=FKBP1A |
| DCC | DCC Netrin 1 Receptor | Protein Coding | 47 | GC18P052340 | 0.346795946 | https://www.genecards.org/cgi-bin/carddisp.pl?gene=DCC |
| EFNB1 | Ephrin B1 | Protein Coding | 47 | GC0XP068828 | 0.346795946 | https://www.genecards.org/cgi-bin/carddisp.pl?gene=EFNB1 |
| CLCN2 | Chloride Voltage-Gated Channel 2 | Protein Coding | 47 | GC03M184346 | 0.346795946 | https://www.genecards.org/cgi-bin/carddisp.pl?gene=CLCN2 |
| ALPP | Alkaline Phosphatase, Placental | Protein Coding | 47 | GC02P232378 | 0.346795946 | https://www.genecards.org/cgi-bin/carddisp.pl?gene=ALPP |
| ABCC3 | ATP Binding Cassette Subfamily C Member 3 | Protein Coding | 47 | GC17P050634 | 0.346795946 | https://www.genecards.org/cgi-bin/carddisp.pl?gene=ABCC3 |
| ABCB11 | ATP Binding Cassette Subfamily B Member 11 | Protein Coding | 47 | GC02M168922 | 0.346795946 | https://www.genecards.org/cgi-bin/carddisp.pl?gene=ABCB11 |
| BARD1 | BRCA1 Associated RING Domain 1 | Protein Coding | 47 | GC02M214725 | 0.346795946 | https://www.genecards.org/cgi-bin/carddisp.pl?gene=BARD1 |
| BMP1 | Bone Morphogenetic Protein 1 | Protein Coding | 47 | GC08P022164 | 0.346795946 | https://www.genecards.org/cgi-bin/carddisp.pl?gene=BMP1 |
| ARSA | Arylsulfatase A | Protein Coding | 47 | GC22M050622 | 0.346795946 | https://www.genecards.org/cgi-bin/carddisp.pl?gene=ARSA |
| EIF4G1 | Eukaryotic Translation Initiation Factor 4 Gamma 1 | Protein Coding | 47 | GC03P184314 | 0.346795946 | https://www.genecards.org/cgi-bin/carddisp.pl?gene=EIF4G1 |
| TCF12 | Transcription Factor 12 | Protein Coding | 47 | GC15P056918 | 0.346795946 | https://www.genecards.org/cgi-bin/carddisp.pl?gene=TCF12 |
| WNT3 | Wnt Family Member 3 | Protein Coding | 47 | GC17M046762 | 0.346795946 | https://www.genecards.org/cgi-bin/carddisp.pl?gene=WNT3 |
| VDAC1 | Voltage Dependent Anion Channel 1 | Protein Coding | 47 | GC05M133975 | 0.346795946 | https://www.genecards.org/cgi-bin/carddisp.pl?gene=VDAC1 |
| SPP1 | Secreted Phosphoprotein 1 | Protein Coding | 47 | GC04P087975 | 0.346795946 | https://www.genecards.org/cgi-bin/carddisp.pl?gene=SPP1 |
| POLD1 | DNA Polymerase Delta 1, Catalytic Subunit | Protein Coding | 47 | GC19P050385 | 0.346795946 | https://www.genecards.org/cgi-bin/carddisp.pl?gene=POLD1 |
| PPARGC1A | PPARG Coactivator 1 Alpha | Protein Coding | 47 | GC04M023755 | 0.346795946 | https://www.genecards.org/cgi-bin/carddisp.pl?gene=PPARGC1A |
| PRSS1 | Serine Protease 1 | Protein Coding | 47 | GC07P145775 | 0.346795946 | https://www.genecards.org/cgi-bin/carddisp.pl?gene=PRSS1 |
| SLC25A12 | Solute Carrier Family 25 Member 12 | Protein Coding | 47 | GC02M171783 | 0.346795946 | https://www.genecards.org/cgi-bin/carddisp.pl?gene=SLC25A12 |
| SMAD6 | SMAD Family Member 6 | Protein Coding | 47 | GC15P066702 | 0.346795946 | https://www.genecards.org/cgi-bin/carddisp.pl?gene=SMAD6 |
| REL | REL Proto-Oncogene, NF-KB Subunit | Protein Coding | 47 | GC02P060881 | 0.346795946 | https://www.genecards.org/cgi-bin/carddisp.pl?gene=REL |
| SLC12A3 | Solute Carrier Family 12 Member 3 | Protein Coding | 47 | GC16P056865 | 0.346795946 | https://www.genecards.org/cgi-bin/carddisp.pl?gene=SLC12A3 |
| SLC6A5 | Solute Carrier Family 6 Member 5 | Protein Coding | 47 | GC11P020599 | 0.346795946 | https://www.genecards.org/cgi-bin/carddisp.pl?gene=SLC6A5 |
| MUC1 | Mucin 1, Cell Surface Associated | Protein Coding | 47 | GC01M155185 | 0.346795946 | https://www.genecards.org/cgi-bin/carddisp.pl?gene=MUC1 |
| MYCN | MYCN Proto-Oncogene, BHLH Transcription Factor | Protein Coding | 47 | GC02P015949 | 0.346795946 | https://www.genecards.org/cgi-bin/carddisp.pl?gene=MYCN |
| PARK7 | Parkinsonism Associated Deglycase | Protein Coding | 47 | GC01P008012 | 0.346795946 | https://www.genecards.org/cgi-bin/carddisp.pl?gene=PARK7 |
| RAB5A | RAB5A, Member RAS Oncogene Family | Protein Coding | 47 | GC03P019963 | 0.346795946 | https://www.genecards.org/cgi-bin/carddisp.pl?gene=RAB5A |
| GABRD | Gamma-Aminobutyric Acid Type A Receptor Subunit Delta | Protein Coding | 46 | GC01P002019 | 0.346795946 | https://www.genecards.org/cgi-bin/carddisp.pl?gene=GABRD |
| CPS1 | Carbamoyl-Phosphate Synthase 1 | Protein Coding | 46 | GC02P210477 | 0.346795946 | https://www.genecards.org/cgi-bin/carddisp.pl?gene=CPS1 |
| AFP | Alpha Fetoprotein | Protein Coding | 46 | GC04P073431 | 0.346795946 | https://www.genecards.org/cgi-bin/carddisp.pl?gene=AFP |
| MAG | Myelin Associated Glycoprotein | Protein Coding | 46 | GC19P035292 | 0.346795946 | https://www.genecards.org/cgi-bin/carddisp.pl?gene=MAG |
| NGFR | Nerve Growth Factor Receptor | Protein Coding | 46 | GC17P049495 | 0.346795946 | https://www.genecards.org/cgi-bin/carddisp.pl?gene=NGFR |
| GNMT | Glycine N-Methyltransferase | Protein Coding | 46 | GC06P042960 | 0.346795946 | https://www.genecards.org/cgi-bin/carddisp.pl?gene=GNMT |
| ARG2 | Arginase 2 | Protein Coding | 46 | GC14P067619 | 0.346795946 | https://www.genecards.org/cgi-bin/carddisp.pl?gene=ARG2 |
| EIF2S3 | Eukaryotic Translation Initiation Factor 2 Subunit Gamma | Protein Coding | 46 | GC0XP024054 | 0.346795946 | https://www.genecards.org/cgi-bin/carddisp.pl?gene=EIF2S3 |
| TCF3 | Transcription Factor 3 | Protein Coding | 46 | GC19M001609 | 0.346795946 | https://www.genecards.org/cgi-bin/carddisp.pl?gene=TCF3 |
| TXK | TXK Tyrosine Kinase | Protein Coding | 46 | GC04M048069 | 0.346795946 | https://www.genecards.org/cgi-bin/carddisp.pl?gene=TXK |
| MMAB | Metabolism Of Cobalamin Associated B | Protein Coding | 46 | GC12M109553 | 0.346795946 | https://www.genecards.org/cgi-bin/carddisp.pl?gene=MMAB |
| WNT1 | Wnt Family Member 1 | Protein Coding | 46 | GC12P049053 | 0.346795946 | https://www.genecards.org/cgi-bin/carddisp.pl?gene=WNT1 |
| WNT10A | Wnt Family Member 10A | Protein Coding | 46 | GC02P218880 | 0.346795946 | https://www.genecards.org/cgi-bin/carddisp.pl?gene=WNT10A |
| ZIC1 | Zic Family Member 1 | Protein Coding | 46 | GC03P147393 | 0.346795946 | https://www.genecards.org/cgi-bin/carddisp.pl?gene=ZIC1 |
| TP73 | Tumor Protein P73 | Protein Coding | 46 | GC01P003652 | 0.346795946 | https://www.genecards.org/cgi-bin/carddisp.pl?gene=TP73 |
| PML | PML Nuclear Body Scaffold | Protein Coding | 46 | GC15P073994 | 0.346795946 | https://www.genecards.org/cgi-bin/carddisp.pl?gene=PML |
| PPP3R1 | Protein Phosphatase 3 Regulatory Subunit B, Alpha | Protein Coding | 46 | GC02M068143 | 0.346795946 | https://www.genecards.org/cgi-bin/carddisp.pl?gene=PPP3R1 |
| SMARCE1 | SWI/SNF Related, Matrix Associated, Actin Dependent Regulator Of Chromatin, Subfamily E, Member 1 | Protein Coding | 46 | GC17M040624 | 0.346795946 | https://www.genecards.org/cgi-bin/carddisp.pl?gene=SMARCE1 |
| SMARCC2 | SWI/SNF Related, Matrix Associated, Actin Dependent Regulator Of Chromatin Subfamily C Member 2 | Protein Coding | 46 | GC12M056206 | 0.346795946 | https://www.genecards.org/cgi-bin/carddisp.pl?gene=SMARCC2 |
| RORC | RAR Related Orphan Receptor C | Protein Coding | 46 | GC01M151806 | 0.346795946 | https://www.genecards.org/cgi-bin/carddisp.pl?gene=RORC |
| MSX1 | Msh Homeobox 1 | Protein Coding | 46 | GC04P004861 | 0.346795946 | https://www.genecards.org/cgi-bin/carddisp.pl?gene=MSX1 |
| MCM5 | Minichromosome Maintenance Complex Component 5 | Protein Coding | 46 | GC22P035400 | 0.346795946 | https://www.genecards.org/cgi-bin/carddisp.pl?gene=MCM5 |
| FZD1 | Frizzled Class Receptor 1 | Protein Coding | 45 | GC07P091264 | 0.346795946 | https://www.genecards.org/cgi-bin/carddisp.pl?gene=FZD1 |
| COL11A2 | Collagen Type XI Alpha 2 Chain | Protein Coding | 45 | GC06M033162 | 0.346795946 | https://www.genecards.org/cgi-bin/carddisp.pl?gene=COL11A2 |
| CR1 | Complement C3b/C4b Receptor 1 (Knops Blood Group) | Protein Coding | 45 | GC01P207496 | 0.346795946 | https://www.genecards.org/cgi-bin/carddisp.pl?gene=CR1 |
| BCL6 | BCL6 Transcription Repressor | Protein Coding | 45 | GC03M187721 | 0.346795946 | https://www.genecards.org/cgi-bin/carddisp.pl?gene=BCL6 |
| CCR4 | C-C Motif Chemokine Receptor 4 | Protein Coding | 45 | GC03P032951 | 0.346795946 | https://www.genecards.org/cgi-bin/carddisp.pl?gene=CCR4 |
| HAND2 | Heart And Neural Crest Derivatives Expressed 2 | Protein Coding | 45 | GC04M173524 | 0.346795946 | https://www.genecards.org/cgi-bin/carddisp.pl?gene=HAND2 |
| MBP | Myelin Basic Protein | Protein Coding | 45 | GC18M076978 | 0.346795946 | https://www.genecards.org/cgi-bin/carddisp.pl?gene=MBP |
| GALE | UDP-Galactose-4-Epimerase | Protein Coding | 45 | GC01M023795 | 0.346795946 | https://www.genecards.org/cgi-bin/carddisp.pl?gene=GALE |
| KCND2 | Potassium Voltage-Gated Channel Subfamily D Member 2 | Protein Coding | 45 | GC07P120273 | 0.346795946 | https://www.genecards.org/cgi-bin/carddisp.pl?gene=KCND2 |
| KCNE1 | Potassium Voltage-Gated Channel Subfamily E Regulatory Subunit 1 | Protein Coding | 45 | GC21M034446 | 0.346795946 | https://www.genecards.org/cgi-bin/carddisp.pl?gene=KCNE1 |
| HMGA2 | High Mobility Group AT-Hook 2 | Protein Coding | 45 | GC12P065824 | 0.346795946 | https://www.genecards.org/cgi-bin/carddisp.pl?gene=HMGA2 |
| NDUFA12 | NADH:Ubiquinone Oxidoreductase Subunit A12 | Protein Coding | 45 | GC12M094898 | 0.346795946 | https://www.genecards.org/cgi-bin/carddisp.pl?gene=NDUFA12 |
| NFATC2 | Nuclear Factor Of Activated T Cells 2 | Protein Coding | 45 | GC20M051386 | 0.346795946 | https://www.genecards.org/cgi-bin/carddisp.pl?gene=NFATC2 |
| NFATC4 | Nuclear Factor Of Activated T Cells 4 | Protein Coding | 45 | GC14P024365 | 0.346795946 | https://www.genecards.org/cgi-bin/carddisp.pl?gene=NFATC4 |
| EIF2S1 | Eukaryotic Translation Initiation Factor 2 Subunit Alpha | Protein Coding | 45 | GC14P067359 | 0.346795946 | https://www.genecards.org/cgi-bin/carddisp.pl?gene=EIF2S1 |
| ETFDH | Electron Transfer Flavoprotein Dehydrogenase | Protein Coding | 45 | GC04P158672 | 0.346795946 | https://www.genecards.org/cgi-bin/carddisp.pl?gene=ETFDH |
| CD38 | CD38 Molecule | Protein Coding | 45 | GC04P015779 | 0.346795946 | https://www.genecards.org/cgi-bin/carddisp.pl?gene=CD38 |
| BRD2 | Bromodomain Containing 2 | Protein Coding | 45 | GC06P055236 | 0.346795946 | https://www.genecards.org/cgi-bin/carddisp.pl?gene=BRD2 |
| CHRNB2 | Cholinergic Receptor Nicotinic Beta 2 Subunit | Protein Coding | 45 | GC01P154568 | 0.346795946 | https://www.genecards.org/cgi-bin/carddisp.pl?gene=CHRNB2 |
| EIF2AK4 | Eukaryotic Translation Initiation Factor 2 Alpha Kinase 4 | Protein Coding | 45 | GC15P039934 | 0.346795946 | https://www.genecards.org/cgi-bin/carddisp.pl?gene=EIF2AK4 |
| CST3 | Cystatin C | Protein Coding | 45 | GC20M023627 | 0.346795946 | https://www.genecards.org/cgi-bin/carddisp.pl?gene=CST3 |
| RNASEL | Ribonuclease L | Protein Coding | 45 | GC01M182542 | 0.346795946 | https://www.genecards.org/cgi-bin/carddisp.pl?gene=RNASEL |
| RBP4 | Retinol Binding Protein 4 | Protein Coding | 45 | GC10M093591 | 0.346795946 | https://www.genecards.org/cgi-bin/carddisp.pl?gene=RBP4 |
| WNT11 | Wnt Family Member 11 | Protein Coding | 45 | GC11M076186 | 0.346795946 | https://www.genecards.org/cgi-bin/carddisp.pl?gene=WNT11 |
| WNT2B | Wnt Family Member 2B | Protein Coding | 45 | GC01P112466 | 0.346795946 | https://www.genecards.org/cgi-bin/carddisp.pl?gene=WNT2B |
| WNT5B | Wnt Family Member 5B | Protein Coding | 45 | GC12P001529 | 0.346795946 | https://www.genecards.org/cgi-bin/carddisp.pl?gene=WNT5B |
| WNT7B | Wnt Family Member 7B | Protein Coding | 45 | GC22M045920 | 0.346795946 | https://www.genecards.org/cgi-bin/carddisp.pl?gene=WNT7B |
| TLE1 | TLE Family Member 1, Transcriptional Corepressor | Protein Coding | 45 | GC09M081583 | 0.346795946 | https://www.genecards.org/cgi-bin/carddisp.pl?gene=TLE1 |
| SP1 | Sp1 Transcription Factor | Protein Coding | 45 | GC12P053380 | 0.346795946 | https://www.genecards.org/cgi-bin/carddisp.pl?gene=SP1 |
| SNAI1 | Snail Family Transcriptional Repressor 1 | Protein Coding | 45 | GC20P049982 | 0.346795946 | https://www.genecards.org/cgi-bin/carddisp.pl?gene=SNAI1 |
| PPP3CB | Protein Phosphatase 3 Catalytic Subunit Beta | Protein Coding | 45 | GC10M073436 | 0.346795946 | https://www.genecards.org/cgi-bin/carddisp.pl?gene=PPP3CB |
| PPP3CC | Protein Phosphatase 3 Catalytic Subunit Gamma | Protein Coding | 45 | GC08P022440 | 0.346795946 | https://www.genecards.org/cgi-bin/carddisp.pl?gene=PPP3CC |
| SLC15A1 | Solute Carrier Family 15 Member 1 | Protein Coding | 45 | GC13M098683 | 0.346795946 | https://www.genecards.org/cgi-bin/carddisp.pl?gene=SLC15A1 |
| MUTYH | MutY DNA Glycosylase | Protein Coding | 45 | GC01M045329 | 0.346795946 | https://www.genecards.org/cgi-bin/carddisp.pl?gene=MUTYH |
| ROS1 | ROS Proto-Oncogene 1, Receptor Tyrosine Kinase | Protein Coding | 45 | GC06M117287 | 0.346795946 | https://www.genecards.org/cgi-bin/carddisp.pl?gene=ROS1 |
| RPL10 | Ribosomal Protein L10 | Protein Coding | 45 | GC0XP154389 | 0.346795946 | https://www.genecards.org/cgi-bin/carddisp.pl?gene=RPL10 |
| RAD23B | RAD23 Homolog B, Nucleotide Excision Repair Protein | Protein Coding | 45 | GC09P107283 | 0.346795946 | https://www.genecards.org/cgi-bin/carddisp.pl?gene=RAD23B |
| PTGDS | Prostaglandin D2 Synthase | Protein Coding | 45 | GC09P137005 | 0.346795946 | https://www.genecards.org/cgi-bin/carddisp.pl?gene=PTGDS |
| SAG | S-Antigen Visual Arrestin | Protein Coding | 45 | GC02P233328 | 0.346795946 | https://www.genecards.org/cgi-bin/carddisp.pl?gene=SAG |
| NEU1 | Neuraminidase 1 | Protein Coding | 45 | GC06M031857 | 0.346795946 | https://www.genecards.org/cgi-bin/carddisp.pl?gene=NEU1 |
| ABCC5 | ATP Binding Cassette Subfamily C Member 5 | Protein Coding | 44 | GC03M183919 | 0.346795946 | https://www.genecards.org/cgi-bin/carddisp.pl?gene=ABCC5 |
| FOXP2 | Forkhead Box P2 | Protein Coding | 44 | GC07P114086 | 0.346795946 | https://www.genecards.org/cgi-bin/carddisp.pl?gene=FOXP2 |
| FSCN1 | Fascin Actin-Bundling Protein 1 | Protein Coding | 44 | GC07P005592 | 0.346795946 | https://www.genecards.org/cgi-bin/carddisp.pl?gene=FSCN1 |
| FZD8 | Frizzled Class Receptor 8 | Protein Coding | 44 | GC10M035638 | 0.346795946 | https://www.genecards.org/cgi-bin/carddisp.pl?gene=FZD8 |
| COL9A3 | Collagen Type IX Alpha 3 Chain | Protein Coding | 44 | GC20P062816 | 0.346795946 | https://www.genecards.org/cgi-bin/carddisp.pl?gene=COL9A3 |
| COL4A5 | Collagen Type IV Alpha 5 Chain | Protein Coding | 44 | GC0XP108439 | 0.346795946 | https://www.genecards.org/cgi-bin/carddisp.pl?gene=COL4A5 |
| IL3 | Interleukin 3 | Protein Coding | 44 | GC05P132060 | 0.346795946 | https://www.genecards.org/cgi-bin/carddisp.pl?gene=IL3 |
| ITGA9 | Integrin Subunit Alpha 9 | Protein Coding | 44 | GC03P037468 | 0.346795946 | https://www.genecards.org/cgi-bin/carddisp.pl?gene=ITGA9 |
| IGFBP2 | Insulin Like Growth Factor Binding Protein 2 | Protein Coding | 44 | GC02P216632 | 0.346795946 | https://www.genecards.org/cgi-bin/carddisp.pl?gene=IGFBP2 |
| GREM1 | Gremlin 1, DAN Family BMP Antagonist | Protein Coding | 44 | GC15P033128 | 0.346795946 | https://www.genecards.org/cgi-bin/carddisp.pl?gene=GREM1 |
| LRP4 | LDL Receptor Related Protein 4 | Protein Coding | 44 | GC11M068984 | 0.346795946 | https://www.genecards.org/cgi-bin/carddisp.pl?gene=LRP4 |
| ITM2B | Integral Membrane Protein 2B | Protein Coding | 44 | GC13P048233 | 0.346795946 | https://www.genecards.org/cgi-bin/carddisp.pl?gene=ITM2B |
| NDUFA9 | NADH:Ubiquinone Oxidoreductase Subunit A9 | Protein Coding | 44 | GC12P004649 | 0.346795946 | https://www.genecards.org/cgi-bin/carddisp.pl?gene=NDUFA9 |
| NFATC3 | Nuclear Factor Of Activated T Cells 3 | Protein Coding | 44 | GC16P068119 | 0.346795946 | https://www.genecards.org/cgi-bin/carddisp.pl?gene=NFATC3 |
| GH1 | Growth Hormone 1 | Protein Coding | 44 | GC17M063917 | 0.346795946 | https://www.genecards.org/cgi-bin/carddisp.pl?gene=GH1 |
| GHRHR | Growth Hormone Releasing Hormone Receptor | Protein Coding | 44 | GC07P030938 | 0.346795946 | https://www.genecards.org/cgi-bin/carddisp.pl?gene=GHRHR |
| EWSR1 | EWS RNA Binding Protein 1 | Protein Coding | 44 | GC22P029269 | 0.346795946 | https://www.genecards.org/cgi-bin/carddisp.pl?gene=EWSR1 |
| DSG1 | Desmoglein 1 | Protein Coding | 44 | GC18P031318 | 0.346795946 | https://www.genecards.org/cgi-bin/carddisp.pl?gene=DSG1 |
| EFEMP2 | EGF Containing Fibulin Extracellular Matrix Protein 2 | Protein Coding | 44 | GC11M069431 | 0.346795946 | https://www.genecards.org/cgi-bin/carddisp.pl?gene=EFEMP2 |
| CD34 | CD34 Molecule | Protein Coding | 44 | GC01M207880 | 0.346795946 | https://www.genecards.org/cgi-bin/carddisp.pl?gene=CD34 |
| CDA | Cytidine Deaminase | Protein Coding | 44 | GC01P020588 | 0.346795946 | https://www.genecards.org/cgi-bin/carddisp.pl?gene=CDA |
| CUX1 | Cut Like Homeobox 1 | Protein Coding | 44 | GC07P101815 | 0.346795946 | https://www.genecards.org/cgi-bin/carddisp.pl?gene=CUX1 |
| AKAP13 | A-Kinase Anchoring Protein 13 | Protein Coding | 44 | GC15P085388 | 0.346795946 | https://www.genecards.org/cgi-bin/carddisp.pl?gene=AKAP13 |
| ACAA1 | Acetyl-CoA Acyltransferase 1 | Protein Coding | 44 | GC03M038103 | 0.346795946 | https://www.genecards.org/cgi-bin/carddisp.pl?gene=ACAA1 |
| BCKDHB | Branched Chain Keto Acid Dehydrogenase E1 Subunit Beta | Protein Coding | 44 | GC06P080106 | 0.346795946 | https://www.genecards.org/cgi-bin/carddisp.pl?gene=BCKDHB |
| EEF1A1 | Eukaryotic Translation Elongation Factor 1 Alpha 1 | Protein Coding | 44 | GC06M073515 | 0.346795946 | https://www.genecards.org/cgi-bin/carddisp.pl?gene=EEF1A1 |
| EIF2AK1 | Eukaryotic Translation Initiation Factor 2 Alpha Kinase 1 | Protein Coding | 44 | GC07M006022 | 0.346795946 | https://www.genecards.org/cgi-bin/carddisp.pl?gene=EIF2AK1 |
| TSPAN7 | Tetraspanin 7 | Protein Coding | 44 | GC0XP038561 | 0.346795946 | https://www.genecards.org/cgi-bin/carddisp.pl?gene=TSPAN7 |
| UBE2G1 | Ubiquitin Conjugating Enzyme E2 G1 | Protein Coding | 44 | GC17M004270 | 0.346795946 | https://www.genecards.org/cgi-bin/carddisp.pl?gene=UBE2G1 |
| SPI1 | Spi-1 Proto-Oncogene | Protein Coding | 44 | GC11M068998 | 0.346795946 | https://www.genecards.org/cgi-bin/carddisp.pl?gene=SPI1 |
| WNT2 | Wnt Family Member 2 | Protein Coding | 44 | GC07M117276 | 0.346795946 | https://www.genecards.org/cgi-bin/carddisp.pl?gene=WNT2 |
| TRAF1 | TNF Receptor Associated Factor 1 | Protein Coding | 44 | GC09M120902 | 0.346795946 | https://www.genecards.org/cgi-bin/carddisp.pl?gene=TRAF1 |
| UNC13D | Unc-13 Homolog D | Protein Coding | 44 | GC17M075827 | 0.346795946 | https://www.genecards.org/cgi-bin/carddisp.pl?gene=UNC13D |
| TRH | Thyrotropin Releasing Hormone | Protein Coding | 44 | GC03P129974 | 0.346795946 | https://www.genecards.org/cgi-bin/carddisp.pl?gene=TRH |
| TRHR | Thyrotropin Releasing Hormone Receptor | Protein Coding | 44 | GC08P109084 | 0.346795946 | https://www.genecards.org/cgi-bin/carddisp.pl?gene=TRHR |
| XRCC1 | X-Ray Repair Cross Complementing 1 | Protein Coding | 44 | GC19M043543 | 0.346795946 | https://www.genecards.org/cgi-bin/carddisp.pl?gene=XRCC1 |
| SPINK1 | Serine Peptidase Inhibitor Kazal Type 1 | Protein Coding | 44 | GC05M147825 | 0.346795946 | https://www.genecards.org/cgi-bin/carddisp.pl?gene=SPINK1 |
| SMAD1 | SMAD Family Member 1 | Protein Coding | 44 | GC04P145481 | 0.346795946 | https://www.genecards.org/cgi-bin/carddisp.pl?gene=SMAD1 |
| POLR3A | RNA Polymerase III Subunit A | Protein Coding | 44 | GC10M077969 | 0.346795946 | https://www.genecards.org/cgi-bin/carddisp.pl?gene=POLR3A |
| POLR3B | RNA Polymerase III Subunit B | Protein Coding | 44 | GC12P106357 | 0.346795946 | https://www.genecards.org/cgi-bin/carddisp.pl?gene=POLR3B |
| PPP6C | Protein Phosphatase 6 Catalytic Subunit | Protein Coding | 44 | GC09M125147 | 0.346795946 | https://www.genecards.org/cgi-bin/carddisp.pl?gene=PPP6C |
| SLC25A15 | Solute Carrier Family 25 Member 15 | Protein Coding | 44 | GC13P040789 | 0.346795946 | https://www.genecards.org/cgi-bin/carddisp.pl?gene=SLC25A15 |
| SMARCC1 | SWI/SNF Related, Matrix Associated, Actin Dependent Regulator Of Chromatin Subfamily C Member 1 | Protein Coding | 44 | GC03M047585 | 0.346795946 | https://www.genecards.org/cgi-bin/carddisp.pl?gene=SMARCC1 |
| SLC1A5 | Solute Carrier Family 1 Member 5 | Protein Coding | 44 | GC19M047159 | 0.346795946 | https://www.genecards.org/cgi-bin/carddisp.pl?gene=SLC1A5 |
| SLC25A6 | Solute Carrier Family 25 Member 6 | Protein Coding | 44 | GC0XM001386 | 0.346795946 | https://www.genecards.org/cgi-bin/carddisp.pl?gene=SLC25A6 |
| PADI4 | Peptidyl Arginine Deiminase 4 | Protein Coding | 44 | GC01P017308 | 0.346795946 | https://www.genecards.org/cgi-bin/carddisp.pl?gene=PADI4 |
| NLRP7 | NLR Family Pyrin Domain Containing 7 | Protein Coding | 44 | GC19M054923 | 0.346795946 | https://www.genecards.org/cgi-bin/carddisp.pl?gene=NLRP7 |
| RSPO1 | R-Spondin 1 | Protein Coding | 44 | GC01M037612 | 0.346795946 | https://www.genecards.org/cgi-bin/carddisp.pl?gene=RSPO1 |
| RAD51C | RAD51 Paralog C | Protein Coding | 44 | GC17P058692 | 0.346795946 | https://www.genecards.org/cgi-bin/carddisp.pl?gene=RAD51C |
| PPIF | Peptidylprolyl Isomerase F | Protein Coding | 44 | GC10P085378 | 0.346795946 | https://www.genecards.org/cgi-bin/carddisp.pl?gene=PPIF |
| PPP1R1B | Protein Phosphatase 1 Regulatory Inhibitor Subunit 1B | Protein Coding | 44 | GC17P039626 | 0.346795946 | https://www.genecards.org/cgi-bin/carddisp.pl?gene=PPP1R1B |
| SDC1 | Syndecan 1 | Protein Coding | 44 | GC02M020200 | 0.346795946 | https://www.genecards.org/cgi-bin/carddisp.pl?gene=SDC1 |
| MSH3 | MutS Homolog 3 | Protein Coding | 44 | GC05P080654 | 0.346795946 | https://www.genecards.org/cgi-bin/carddisp.pl?gene=MSH3 |
| NRF1 | Nuclear Respiratory Factor 1 | Protein Coding | 44 | GC07P129611 | 0.346795946 | https://www.genecards.org/cgi-bin/carddisp.pl?gene=NRF1 |
| KCNQ5 | Potassium Voltage-Gated Channel Subfamily Q Member 5 | Protein Coding | 44 | GC06P072621 | 0.346795946 | https://www.genecards.org/cgi-bin/carddisp.pl?gene=KCNQ5 |
| RCAN1 | Regulator Of Calcineurin 1 | Protein Coding | 44 | GC21M034513 | 0.346795946 | https://www.genecards.org/cgi-bin/carddisp.pl?gene=RCAN1 |
| ZDHHC9 | Zinc Finger DHHC-Type Palmitoyltransferase 9 | Protein Coding | 44 | GC0XM129803 | 0.346795946 | https://www.genecards.org/cgi-bin/carddisp.pl?gene=ZDHHC9 |
| ARHGEF6 | Rac/Cdc42 Guanine Nucleotide Exchange Factor 6 | Protein Coding | 43 | GC0XM136665 | 0.346795946 | https://www.genecards.org/cgi-bin/carddisp.pl?gene=ARHGEF6 |
| FCN3 | Ficolin 3 | Protein Coding | 43 | GC01M027380 | 0.346795946 | https://www.genecards.org/cgi-bin/carddisp.pl?gene=FCN3 |
| COL9A1 | Collagen Type IX Alpha 1 Chain | Protein Coding | 43 | GC06M070215 | 0.346795946 | https://www.genecards.org/cgi-bin/carddisp.pl?gene=COL9A1 |
| COL9A2 | Collagen Type IX Alpha 2 Chain | Protein Coding | 43 | GC01M040300 | 0.346795946 | https://www.genecards.org/cgi-bin/carddisp.pl?gene=COL9A2 |
| LAMP1 | Lysosomal Associated Membrane Protein 1 | Protein Coding | 43 | GC13P113297 | 0.346795946 | https://www.genecards.org/cgi-bin/carddisp.pl?gene=LAMP1 |
| LIN28B | Lin-28 Homolog B | Protein Coding | 43 | GC06P104943 | 0.346795946 | https://www.genecards.org/cgi-bin/carddisp.pl?gene=LIN28B |
| MFN1 | Mitofusin 1 | Protein Coding | 43 | GC03P179347 | 0.346795946 | https://www.genecards.org/cgi-bin/carddisp.pl?gene=MFN1 |
| HSD3B7 | Hydroxy-Delta-5-Steroid Dehydrogenase, 3 Beta- And Steroid Delta-Isomerase 7 | Protein Coding | 43 | GC16P030985 | 0.346795946 | https://www.genecards.org/cgi-bin/carddisp.pl?gene=HSD3B7 |
| HBG2 | Hemoglobin Subunit Gamma 2 | Protein Coding | 43 | GC11M005442 | 0.346795946 | https://www.genecards.org/cgi-bin/carddisp.pl?gene=HBG2 |
| LMO2 | LIM Domain Only 2 | Protein Coding | 43 | GC11M033858 | 0.346795946 | https://www.genecards.org/cgi-bin/carddisp.pl?gene=LMO2 |
| JUNB | JunB Proto-Oncogene, AP-1 Transcription Factor Subunit | Protein Coding | 43 | GC19P012791 | 0.346795946 | https://www.genecards.org/cgi-bin/carddisp.pl?gene=JUNB |
| FBXO11 | F-Box Protein 11 | Protein Coding | 43 | GC02M047789 | 0.346795946 | https://www.genecards.org/cgi-bin/carddisp.pl?gene=FBXO11 |
| DROSHA | Drosha Ribonuclease III | Protein Coding | 43 | GC05M031401 | 0.346795946 | https://www.genecards.org/cgi-bin/carddisp.pl?gene=DROSHA |
| FCGR3B | Fc Fragment Of IgG Receptor IIIb | Protein Coding | 43 | GC01M161623 | 0.346795946 | https://www.genecards.org/cgi-bin/carddisp.pl?gene=FCGR3B |
| DYNLL1 | Dynein Light Chain LC8-Type 1 | Protein Coding | 43 | GC12P120469 | 0.346795946 | https://www.genecards.org/cgi-bin/carddisp.pl?gene=DYNLL1 |
| EFNA4 | Ephrin A4 | Protein Coding | 43 | GC01P155063 | 0.346795946 | https://www.genecards.org/cgi-bin/carddisp.pl?gene=EFNA4 |
| DIAPH3 | Diaphanous Related Formin 3 | Protein Coding | 43 | GC13M059665 | 0.346795946 | https://www.genecards.org/cgi-bin/carddisp.pl?gene=DIAPH3 |
| CXCL1 | C-X-C Motif Chemokine Ligand 1 | Protein Coding | 43 | GC04P073869 | 0.346795946 | https://www.genecards.org/cgi-bin/carddisp.pl?gene=CXCL1 |
| AIM2 | Absent In Melanoma 2 | Protein Coding | 43 | GC01M159062 | 0.346795946 | https://www.genecards.org/cgi-bin/carddisp.pl?gene=AIM2 |
| ALG6 | ALG6 Alpha-1,3-Glucosyltransferase | Protein Coding | 43 | GC01P063367 | 0.346795946 | https://www.genecards.org/cgi-bin/carddisp.pl?gene=ALG6 |
| ACTC1 | Actin Alpha Cardiac Muscle 1 | Protein Coding | 43 | GC15M034788 | 0.346795946 | https://www.genecards.org/cgi-bin/carddisp.pl?gene=ACTC1 |
| ABCC11 | ATP Binding Cassette Subfamily C Member 11 | Protein Coding | 43 | GC16M048166 | 0.346795946 | https://www.genecards.org/cgi-bin/carddisp.pl?gene=ABCC11 |
| BNIP3L | BCL2 Interacting Protein 3 Like | Protein Coding | 43 | GC08P026296 | 0.346795946 | https://www.genecards.org/cgi-bin/carddisp.pl?gene=BNIP3L |
| ANXA6 | Annexin A6 | Protein Coding | 43 | GC05M151100 | 0.346795946 | https://www.genecards.org/cgi-bin/carddisp.pl?gene=ANXA6 |
| EIF5 | Eukaryotic Translation Initiation Factor 5 | Protein Coding | 43 | GC14P103333 | 0.346795946 | https://www.genecards.org/cgi-bin/carddisp.pl?gene=EIF5 |
| ENDOG | Endonuclease G | Protein Coding | 43 | GC09P128818 | 0.346795946 | https://www.genecards.org/cgi-bin/carddisp.pl?gene=ENDOG |
| E2F2 | E2F Transcription Factor 2 | Protein Coding | 43 | GC01M023541 | 0.346795946 | https://www.genecards.org/cgi-bin/carddisp.pl?gene=E2F2 |
| OSGEP | O-Sialoglycoprotein Endopeptidase | Protein Coding | 43 | GC14M020446 | 0.346795946 | https://www.genecards.org/cgi-bin/carddisp.pl?gene=OSGEP |
| MLYCD | Malonyl-CoA Decarboxylase | Protein Coding | 43 | GC16P083899 | 0.346795946 | https://www.genecards.org/cgi-bin/carddisp.pl?gene=MLYCD |
| MMADHC | Metabolism Of Cobalamin Associated D | Protein Coding | 43 | GC02M149569 | 0.346795946 | https://www.genecards.org/cgi-bin/carddisp.pl?gene=MMADHC |
| TWIST2 | Twist Family BHLH Transcription Factor 2 | Protein Coding | 43 | GC02P238848 | 0.346795946 | https://www.genecards.org/cgi-bin/carddisp.pl?gene=TWIST2 |
| TRMT1 | TRNA Methyltransferase 1 | Protein Coding | 43 | GC19M013104 | 0.346795946 | https://www.genecards.org/cgi-bin/carddisp.pl?gene=TRMT1 |
| WNT6 | Wnt Family Member 6 | Protein Coding | 43 | GC02P218859 | 0.346795946 | https://www.genecards.org/cgi-bin/carddisp.pl?gene=WNT6 |
| WNT9A | Wnt Family Member 9A | Protein Coding | 43 | GC01M227920 | 0.346795946 | https://www.genecards.org/cgi-bin/carddisp.pl?gene=WNT9A |
| XRCC2 | X-Ray Repair Cross Complementing 2 | Protein Coding | 43 | GC07M152644 | 0.346795946 | https://www.genecards.org/cgi-bin/carddisp.pl?gene=XRCC2 |
| TEAD4 | TEA Domain Transcription Factor 4 | Protein Coding | 43 | GC12P002959 | 0.346795946 | https://www.genecards.org/cgi-bin/carddisp.pl?gene=TEAD4 |
| TMLHE | Trimethyllysine Hydroxylase, Epsilon | Protein Coding | 43 | GC0XM155489 | 0.346795946 | https://www.genecards.org/cgi-bin/carddisp.pl?gene=TMLHE |
| PTPN4 | Protein Tyrosine Phosphatase Non-Receptor Type 4 | Protein Coding | 43 | GC02P119759 | 0.346795946 | https://www.genecards.org/cgi-bin/carddisp.pl?gene=PTPN4 |
| PTPRG | Protein Tyrosine Phosphatase Receptor Type G | Protein Coding | 43 | GC03P061561 | 0.346795946 | https://www.genecards.org/cgi-bin/carddisp.pl?gene=PTPRG |
| SLC7A8 | Solute Carrier Family 7 Member 8 | Protein Coding | 43 | GC14M023125 | 0.346795946 | https://www.genecards.org/cgi-bin/carddisp.pl?gene=SLC7A8 |
| RECQL4 | RecQ Like Helicase 4 | Protein Coding | 43 | GC08M144512 | 0.346795946 | https://www.genecards.org/cgi-bin/carddisp.pl?gene=RECQL4 |
| SLC39A13 | Solute Carrier Family 39 Member 13 | Protein Coding | 43 | GC11P047407 | 0.346795946 | https://www.genecards.org/cgi-bin/carddisp.pl?gene=SLC39A13 |
| SLC38A2 | Solute Carrier Family 38 Member 2 | Protein Coding | 43 | GC12M046358 | 0.346795946 | https://www.genecards.org/cgi-bin/carddisp.pl?gene=SLC38A2 |
| SNAP29 | Synaptosome Associated Protein 29 | Protein Coding | 43 | GC22P020859 | 0.346795946 | https://www.genecards.org/cgi-bin/carddisp.pl?gene=SNAP29 |
| PDIA4 | Protein Disulfide Isomerase Family A Member 4 | Protein Coding | 43 | GC07M149003 | 0.346795946 | https://www.genecards.org/cgi-bin/carddisp.pl?gene=PDIA4 |
| PECR | Peroxisomal Trans-2-Enoyl-CoA Reductase | Protein Coding | 43 | GC02M215996 | 0.346795946 | https://www.genecards.org/cgi-bin/carddisp.pl?gene=PECR |
| PALB2 | Partner And Localizer Of BRCA2 | Protein Coding | 43 | GC16M023603 | 0.346795946 | https://www.genecards.org/cgi-bin/carddisp.pl?gene=PALB2 |
| OCA2 | OCA2 Melanosomal Transmembrane Protein | Protein Coding | 43 | GC15M027754 | 0.346795946 | https://www.genecards.org/cgi-bin/carddisp.pl?gene=OCA2 |
| RAB1A | RAB1A, Member RAS Oncogene Family | Protein Coding | 43 | GC02M065072 | 0.346795946 | https://www.genecards.org/cgi-bin/carddisp.pl?gene=RAB1A |
| RAB1B | RAB1B, Member RAS Oncogene Family | Protein Coding | 43 | GC11P066722 | 0.346795946 | https://www.genecards.org/cgi-bin/carddisp.pl?gene=RAB1B |
| RPS9 | Ribosomal Protein S9 | Protein Coding | 43 | GC19P056806 | 0.346795946 | https://www.genecards.org/cgi-bin/carddisp.pl?gene=RPS9 |
| RAD54B | RAD54 Homolog B | Protein Coding | 43 | GC08M094371 | 0.346795946 | https://www.genecards.org/cgi-bin/carddisp.pl?gene=RAD54B |
| RHOT1 | Ras Homolog Family Member T1 | Protein Coding | 43 | GC17P032142 | 0.346795946 | https://www.genecards.org/cgi-bin/carddisp.pl?gene=RHOT1 |
| RYK | Receptor Like Tyrosine Kinase | Protein Coding | 43 | GC03M134065 | 0.346795946 | https://www.genecards.org/cgi-bin/carddisp.pl?gene=RYK |
| MX1 | MX Dynamin Like GTPase 1 | Protein Coding | 43 | GC21P041420 | 0.346795946 | https://www.genecards.org/cgi-bin/carddisp.pl?gene=MX1 |
| NAGS | N-Acetylglutamate Synthase | Protein Coding | 43 | GC17P044004 | 0.346795946 | https://www.genecards.org/cgi-bin/carddisp.pl?gene=NAGS |
| SLC3A2 | Solute Carrier Family 3 Member 2 | Protein Coding | 43 | GC11P062856 | 0.346795946 | https://www.genecards.org/cgi-bin/carddisp.pl?gene=SLC3A2 |
| SAA1 | Serum Amyloid A1 | Protein Coding | 43 | GC11P018267 | 0.346795946 | https://www.genecards.org/cgi-bin/carddisp.pl?gene=SAA1 |
| TNFRSF8 | TNF Receptor Superfamily Member 8 | Protein Coding | 43 | GC01P012063 | 0.346795946 | https://www.genecards.org/cgi-bin/carddisp.pl?gene=TNFRSF8 |
| YME1L1 | YME1 Like 1 ATPase | Protein Coding | 43 | GC10M027110 | 0.346795946 | https://www.genecards.org/cgi-bin/carddisp.pl?gene=YME1L1 |
| FLCN | Folliculin | Protein Coding | 42 | GC17M017206 | 0.346795946 | https://www.genecards.org/cgi-bin/carddisp.pl?gene=FLCN |
| FTSJ1 | FtsJ RNA 2'-O-Methyltransferase 1 | Protein Coding | 42 | GC0XP048476 | 0.346795946 | https://www.genecards.org/cgi-bin/carddisp.pl?gene=FTSJ1 |
| GALNT12 | Polypeptide N-Acetylgalactosaminyltransferase 12 | Protein Coding | 42 | GC09P098807 | 0.346795946 | https://www.genecards.org/cgi-bin/carddisp.pl?gene=GALNT12 |
| C9orf72 | C9orf72-SMCR8 Complex Subunit | Protein Coding | 42 | GC09M027588 | 0.346795946 | https://www.genecards.org/cgi-bin/carddisp.pl?gene=C9orf72 |
| MFAP5 | Microfibril Associated Protein 5 | Protein Coding | 42 | GC12M008637 | 0.346795946 | https://www.genecards.org/cgi-bin/carddisp.pl?gene=MFAP5 |
| GSTM5 | Glutathione S-Transferase Mu 5 | Protein Coding | 42 | GC01P109711 | 0.346795946 | https://www.genecards.org/cgi-bin/carddisp.pl?gene=GSTM5 |
| IFNB1 | Interferon Beta 1 | Protein Coding | 42 | GC09M021077 | 0.346795946 | https://www.genecards.org/cgi-bin/carddisp.pl?gene=IFNB1 |
| IL22 | Interleukin 22 | Protein Coding | 42 | GC12M068248 | 0.346795946 | https://www.genecards.org/cgi-bin/carddisp.pl?gene=IL22 |
| GJC2 | Gap Junction Protein Gamma 2 | Protein Coding | 42 | GC01P228471 | 0.346795946 | https://www.genecards.org/cgi-bin/carddisp.pl?gene=GJC2 |
| FCN2 | Ficolin 2 | Protein Coding | 42 | GC09P134864 | 0.346795946 | https://www.genecards.org/cgi-bin/carddisp.pl?gene=FCN2 |
| AIMP2 | Aminoacyl TRNA Synthetase Complex Interacting Multifunctional Protein 2 | Protein Coding | 42 | GC07P006016 | 0.346795946 | https://www.genecards.org/cgi-bin/carddisp.pl?gene=AIMP2 |
| ALG3 | ALG3 Alpha-1,3- Mannosyltransferase | Protein Coding | 42 | GC03M184244 | 0.346795946 | https://www.genecards.org/cgi-bin/carddisp.pl?gene=ALG3 |
| CACNG3 | Calcium Voltage-Gated Channel Auxiliary Subunit Gamma 3 | Protein Coding | 42 | GC16P024267 | 0.346795946 | https://www.genecards.org/cgi-bin/carddisp.pl?gene=CACNG3 |
| CABIN1 | Calcineurin Binding Protein 1 | Protein Coding | 42 | GC22P024011 | 0.346795946 | https://www.genecards.org/cgi-bin/carddisp.pl?gene=CABIN1 |
| WNT16 | Wnt Family Member 16 | Protein Coding | 42 | GC07P121325 | 0.346795946 | https://www.genecards.org/cgi-bin/carddisp.pl?gene=WNT16 |
| WNT8B | Wnt Family Member 8B | Protein Coding | 42 | GC10P100463 | 0.346795946 | https://www.genecards.org/cgi-bin/carddisp.pl?gene=WNT8B |
| WNT8A | Wnt Family Member 8A | Protein Coding | 42 | GC05P138091 | 0.346795946 | https://www.genecards.org/cgi-bin/carddisp.pl?gene=WNT8A |
| WNT9B | Wnt Family Member 9B | Protein Coding | 42 | GC17P046833 | 0.346795946 | https://www.genecards.org/cgi-bin/carddisp.pl?gene=WNT9B |
| TK2 | Thymidine Kinase 2 | Protein Coding | 42 | GC16M066508 | 0.346795946 | https://www.genecards.org/cgi-bin/carddisp.pl?gene=TK2 |
| SSTR5 | Somatostatin Receptor 5 | Protein Coding | 42 | GC16P001072 | 0.346795946 | https://www.genecards.org/cgi-bin/carddisp.pl?gene=SSTR5 |
| PARL | Presenilin Associated Rhomboid Like | Protein Coding | 42 | GC03M183826 | 0.346795946 | https://www.genecards.org/cgi-bin/carddisp.pl?gene=PARL |
| SLC6A20 | Solute Carrier Family 6 Member 20 | Protein Coding | 42 | GC03M045755 | 0.346795946 | https://www.genecards.org/cgi-bin/carddisp.pl?gene=SLC6A20 |
| PFN2 | Profilin 2 | Protein Coding | 42 | GC03M149964 | 0.346795946 | https://www.genecards.org/cgi-bin/carddisp.pl?gene=PFN2 |
| S100A1 | S100 Calcium Binding Protein A1 | Protein Coding | 42 | GC01P153627 | 0.346795946 | https://www.genecards.org/cgi-bin/carddisp.pl?gene=S100A1 |
| MRE11 | MRE11 Homolog, Double Strand Break Repair Nuclease | Protein Coding | 42 | GC11M094875 | 0.346795946 | https://www.genecards.org/cgi-bin/carddisp.pl?gene=MRE11 |
| APCS | Amyloid P Component, Serum | Protein Coding | 41 | GC01P159587 | 0.346795946 | https://www.genecards.org/cgi-bin/carddisp.pl?gene=APCS |
| ACSL3 | Acyl-CoA Synthetase Long Chain Family Member 3 | Protein Coding | 41 | GC02P222860 | 0.346795946 | https://www.genecards.org/cgi-bin/carddisp.pl?gene=ACSL3 |
| FCAR | Fc Fragment Of IgA Receptor | Protein Coding | 41 | GC19P056455 | 0.346795946 | https://www.genecards.org/cgi-bin/carddisp.pl?gene=FCAR |
| C1GALT1 | Core 1 Synthase, Glycoprotein-N-Acetylgalactosamine 3-Beta-Galactosyltransferase 1 | Protein Coding | 41 | GC07P007156 | 0.346795946 | https://www.genecards.org/cgi-bin/carddisp.pl?gene=C1GALT1 |
| CD69 | CD69 Molecule | Protein Coding | 41 | GC12M015678 | 0.346795946 | https://www.genecards.org/cgi-bin/carddisp.pl?gene=CD69 |
| CD5 | CD5 Molecule | Protein Coding | 41 | GC11P061114 | 0.346795946 | https://www.genecards.org/cgi-bin/carddisp.pl?gene=CD5 |
| KIR3DL1 | Killer Cell Immunoglobulin Like Receptor, Three Ig Domains And Long Cytoplasmic Tail 1 | Protein Coding | 41 | GC19P056447 | 0.346795946 | https://www.genecards.org/cgi-bin/carddisp.pl?gene=KIR3DL1 |
| LGALS3BP | Galectin 3 Binding Protein | Protein Coding | 41 | GC17M078971 | 0.346795946 | https://www.genecards.org/cgi-bin/carddisp.pl?gene=LGALS3BP |
| MICAL1 | Microtubule Associated Monooxygenase, Calponin And LIM Domain Containing 1 | Protein Coding | 41 | GC06M109444 | 0.346795946 | https://www.genecards.org/cgi-bin/carddisp.pl?gene=MICAL1 |
| GREM2 | Gremlin 2, DAN Family BMP Antagonist | Protein Coding | 41 | GC01M240489 | 0.346795946 | https://www.genecards.org/cgi-bin/carddisp.pl?gene=GREM2 |
| IFNA2 | Interferon Alpha 2 | Protein Coding | 41 | GC09M021384 | 0.346795946 | https://www.genecards.org/cgi-bin/carddisp.pl?gene=IFNA2 |
| FMOD | Fibromodulin | Protein Coding | 41 | GC01M203340 | 0.346795946 | https://www.genecards.org/cgi-bin/carddisp.pl?gene=FMOD |
| AGR2 | Anterior Gradient 2, Protein Disulphide Isomerase Family Member | Protein Coding | 41 | GC07M016892 | 0.346795946 | https://www.genecards.org/cgi-bin/carddisp.pl?gene=AGR2 |
| CLDN11 | Claudin 11 | Protein Coding | 41 | GC03P170418 | 0.346795946 | https://www.genecards.org/cgi-bin/carddisp.pl?gene=CLDN11 |
| CLDN6 | Claudin 6 | Protein Coding | 41 | GC16M003014 | 0.346795946 | https://www.genecards.org/cgi-bin/carddisp.pl?gene=CLDN6 |
| CXCL2 | C-X-C Motif Chemokine Ligand 2 | Protein Coding | 41 | GC04M074097 | 0.346795946 | https://www.genecards.org/cgi-bin/carddisp.pl?gene=CXCL2 |
| ACOT7 | Acyl-CoA Thioesterase 7 | Protein Coding | 41 | GC01M006265 | 0.346795946 | https://www.genecards.org/cgi-bin/carddisp.pl?gene=ACOT7 |
| AAK1 | AP2 Associated Kinase 1 | Protein Coding | 41 | GC02M069459 | 0.346795946 | https://www.genecards.org/cgi-bin/carddisp.pl?gene=AAK1 |
| AKAP6 | A-Kinase Anchoring Protein 6 | Protein Coding | 41 | GC14P032203 | 0.346795946 | https://www.genecards.org/cgi-bin/carddisp.pl?gene=AKAP6 |
| ATAD1 | ATPase Family AAA Domain Containing 1 | Protein Coding | 41 | GC10M087751 | 0.346795946 | https://www.genecards.org/cgi-bin/carddisp.pl?gene=ATAD1 |
| EEF1G | Eukaryotic Translation Elongation Factor 1 Gamma | Protein Coding | 41 | GC11M069243 | 0.346795946 | https://www.genecards.org/cgi-bin/carddisp.pl?gene=EEF1G |
| EIF2S2 | Eukaryotic Translation Initiation Factor 2 Subunit Beta | Protein Coding | 41 | GC20M034088 | 0.346795946 | https://www.genecards.org/cgi-bin/carddisp.pl?gene=EIF2S2 |
| UBASH3B | Ubiquitin Associated And SH3 Domain Containing B | Protein Coding | 41 | GC11P122655 | 0.346795946 | https://www.genecards.org/cgi-bin/carddisp.pl?gene=UBASH3B |
| UBE2D4 | Ubiquitin Conjugating Enzyme E2 D4 (Putative) | Protein Coding | 41 | GC07P043926 | 0.346795946 | https://www.genecards.org/cgi-bin/carddisp.pl?gene=UBE2D4 |
| RBP3 | Retinol Binding Protein 3 | Protein Coding | 41 | GC10P047348 | 0.346795946 | https://www.genecards.org/cgi-bin/carddisp.pl?gene=RBP3 |
| TRAK1 | Trafficking Kinesin Protein 1 | Protein Coding | 41 | GC03P042016 | 0.346795946 | https://www.genecards.org/cgi-bin/carddisp.pl?gene=TRAK1 |
| TSEN2 | TRNA Splicing Endonuclease Subunit 2 | Protein Coding | 41 | GC03P012484 | 0.346795946 | https://www.genecards.org/cgi-bin/carddisp.pl?gene=TSEN2 |
| TOMM40 | Translocase Of Outer Mitochondrial Membrane 40 | Protein Coding | 41 | GC19P044890 | 0.346795946 | https://www.genecards.org/cgi-bin/carddisp.pl?gene=TOMM40 |
| REEP5 | Receptor Accessory Protein 5 | Protein Coding | 41 | GC05M112876 | 0.346795946 | https://www.genecards.org/cgi-bin/carddisp.pl?gene=REEP5 |
| SLC6A14 | Solute Carrier Family 6 Member 14 | Protein Coding | 41 | GC0XP116436 | 0.346795946 | https://www.genecards.org/cgi-bin/carddisp.pl?gene=SLC6A14 |
| SLC6A15 | Solute Carrier Family 6 Member 15 | Protein Coding | 41 | GC12M084859 | 0.346795946 | https://www.genecards.org/cgi-bin/carddisp.pl?gene=SLC6A15 |
| RYBP | RING1 And YY1 Binding Protein | Protein Coding | 41 | GC03M072371 | 0.346795946 | https://www.genecards.org/cgi-bin/carddisp.pl?gene=RYBP |
| PCGF2 | Polycomb Group Ring Finger 2 | Protein Coding | 41 | GC17M038733 | 0.346795946 | https://www.genecards.org/cgi-bin/carddisp.pl?gene=PCGF2 |
| PHB2 | Prohibitin 2 | Protein Coding | 41 | GC12M006965 | 0.346795946 | https://www.genecards.org/cgi-bin/carddisp.pl?gene=PHB2 |
| RAD51D | RAD51 Paralog D | Protein Coding | 41 | GC17M035092 | 0.346795946 | https://www.genecards.org/cgi-bin/carddisp.pl?gene=RAD51D |
| PPP1R15A | Protein Phosphatase 1 Regulatory Subunit 15A | Protein Coding | 41 | GC19P048872 | 0.346795946 | https://www.genecards.org/cgi-bin/carddisp.pl?gene=PPP1R15A |
| MCC | MCC Regulator Of WNT Signaling Pathway | Protein Coding | 41 | GC05M113022 | 0.346795946 | https://www.genecards.org/cgi-bin/carddisp.pl?gene=MCC |
| LIN28A | Lin-28 Homolog A | Protein Coding | 41 | GC01P026410 | 0.346795946 | https://www.genecards.org/cgi-bin/carddisp.pl?gene=LIN28A |
| GABRP | Gamma-Aminobutyric Acid Type A Receptor Subunit Pi | Protein Coding | 40 | GC05P170763 | 0.346795946 | https://www.genecards.org/cgi-bin/carddisp.pl?gene=GABRP |
| COLEC12 | Collectin Subfamily Member 12 | Protein Coding | 40 | GC18M000318 | 0.346795946 | https://www.genecards.org/cgi-bin/carddisp.pl?gene=COLEC12 |
| BACE2 | Beta-Secretase 2 | Protein Coding | 40 | GC21P041168 | 0.346795946 | https://www.genecards.org/cgi-bin/carddisp.pl?gene=BACE2 |
| CD68 | CD68 Molecule | Protein Coding | 40 | GC17P007579 | 0.346795946 | https://www.genecards.org/cgi-bin/carddisp.pl?gene=CD68 |
| HAS1 | Hyaluronan Synthase 1 | Protein Coding | 40 | GC19M051714 | 0.346795946 | https://www.genecards.org/cgi-bin/carddisp.pl?gene=HAS1 |
| IL33 | Interleukin 33 | Protein Coding | 40 | GC09P006206 | 0.346795946 | https://www.genecards.org/cgi-bin/carddisp.pl?gene=IL33 |
| LAT2 | Linker For Activation Of T Cells Family Member 2 | Protein Coding | 40 | GC07P074199 | 0.346795946 | https://www.genecards.org/cgi-bin/carddisp.pl?gene=LAT2 |
| LILRB3 | Leukocyte Immunoglobulin Like Receptor B3 | Protein Coding | 40 | GC19M054216 | 0.346795946 | https://www.genecards.org/cgi-bin/carddisp.pl?gene=LILRB3 |
| HEPACAM | Hepatic And Glial Cell Adhesion Molecule | Protein Coding | 40 | GC11M124919 | 0.346795946 | https://www.genecards.org/cgi-bin/carddisp.pl?gene=HEPACAM |
| HFM1 | Helicase For Meiosis 1 | Protein Coding | 40 | GC01M091260 | 0.346795946 | https://www.genecards.org/cgi-bin/carddisp.pl?gene=HFM1 |
| GSDMD | Gasdermin D | Protein Coding | 40 | GC08P143553 | 0.346795946 | https://www.genecards.org/cgi-bin/carddisp.pl?gene=GSDMD |
| KIAA0319 | KIAA0319 | Protein Coding | 40 | GC06M024544 | 0.346795946 | https://www.genecards.org/cgi-bin/carddisp.pl?gene=KIAA0319 |
| IMMT | Inner Membrane Mitochondrial Protein | Protein Coding | 40 | GC02M086144 | 0.346795946 | https://www.genecards.org/cgi-bin/carddisp.pl?gene=IMMT |
| GHRH | Growth Hormone Releasing Hormone | Protein Coding | 40 | GC20M037251 | 0.346795946 | https://www.genecards.org/cgi-bin/carddisp.pl?gene=GHRH |
| FCN1 | Ficolin 1 | Protein Coding | 40 | GC09M135029 | 0.346795946 | https://www.genecards.org/cgi-bin/carddisp.pl?gene=FCN1 |
| DGCR2 | DiGeorge Syndrome Critical Region Gene 2 | Protein Coding | 40 | GC22M019037 | 0.346795946 | https://www.genecards.org/cgi-bin/carddisp.pl?gene=DGCR2 |
| DSG3 | Desmoglein 3 | Protein Coding | 40 | GC18P031447 | 0.346795946 | https://www.genecards.org/cgi-bin/carddisp.pl?gene=DSG3 |
| CER1 | Cerberus 1, DAN Family BMP Antagonist | Protein Coding | 40 | GC09M014710 | 0.346795946 | https://www.genecards.org/cgi-bin/carddisp.pl?gene=CER1 |
| CCL3 | C-C Motif Chemokine Ligand 3 | Protein Coding | 40 | GC17M036088 | 0.346795946 | https://www.genecards.org/cgi-bin/carddisp.pl?gene=CCL3 |
| CCN2 | Cellular Communication Network Factor 2 | Protein Coding | 40 | GC06M131948 | 0.346795946 | https://www.genecards.org/cgi-bin/carddisp.pl?gene=CCN2 |
| CPVL | Carboxypeptidase Vitellogenic Like | Protein Coding | 40 | GC07M028995 | 0.346795946 | https://www.genecards.org/cgi-bin/carddisp.pl?gene=CPVL |
| CSF3 | Colony Stimulating Factor 3 | Protein Coding | 40 | GC17P040015 | 0.346795946 | https://www.genecards.org/cgi-bin/carddisp.pl?gene=CSF3 |
| ALX4 | ALX Homeobox 4 | Protein Coding | 40 | GC11M044238 | 0.346795946 | https://www.genecards.org/cgi-bin/carddisp.pl?gene=ALX4 |
| AKAP5 | A-Kinase Anchoring Protein 5 | Protein Coding | 40 | GC14P064465 | 0.346795946 | https://www.genecards.org/cgi-bin/carddisp.pl?gene=AKAP5 |
| ANGPTL6 | Angiopoietin Like 6 | Protein Coding | 40 | GC19M010092 | 0.346795946 | https://www.genecards.org/cgi-bin/carddisp.pl?gene=ANGPTL6 |
| ACR | Acrosin | Protein Coding | 40 | GC22P050738 | 0.346795946 | https://www.genecards.org/cgi-bin/carddisp.pl?gene=ACR |
| ABCC12 | ATP Binding Cassette Subfamily C Member 12 | Protein Coding | 40 | GC16M048081 | 0.346795946 | https://www.genecards.org/cgi-bin/carddisp.pl?gene=ABCC12 |
| ACOT11 | Acyl-CoA Thioesterase 11 | Protein Coding | 40 | GC01P054542 | 0.346795946 | https://www.genecards.org/cgi-bin/carddisp.pl?gene=ACOT11 |
| AUTS2 | Activator Of Transcription And Developmental Regulator AUTS2 | Protein Coding | 40 | GC07P069598 | 0.346795946 | https://www.genecards.org/cgi-bin/carddisp.pl?gene=AUTS2 |
| BAG5 | BAG Cochaperone 5 | Protein Coding | 40 | GC14M103556 | 0.346795946 | https://www.genecards.org/cgi-bin/carddisp.pl?gene=BAG5 |
| CDH9 | Cadherin 9 | Protein Coding | 40 | GC05M026916 | 0.346795946 | https://www.genecards.org/cgi-bin/carddisp.pl?gene=CDH9 |
| DTNB | Dystrobrevin Beta | Protein Coding | 40 | GC02M025378 | 0.346795946 | https://www.genecards.org/cgi-bin/carddisp.pl?gene=DTNB |
| ELP4 | Elongator Acetyltransferase Complex Subunit 4 | Protein Coding | 40 | GC11P031509 | 0.346795946 | https://www.genecards.org/cgi-bin/carddisp.pl?gene=ELP4 |
| ELP3 | Elongator Acetyltransferase Complex Subunit 3 | Protein Coding | 40 | GC08P028089 | 0.346795946 | https://www.genecards.org/cgi-bin/carddisp.pl?gene=ELP3 |
| MEST | Mesoderm Specific Transcript | Protein Coding | 40 | GC07P130486 | 0.346795946 | https://www.genecards.org/cgi-bin/carddisp.pl?gene=MEST |
| RNASE1 | Ribonuclease A Family Member 1, Pancreatic | Protein Coding | 40 | GC14M020801 | 0.346795946 | https://www.genecards.org/cgi-bin/carddisp.pl?gene=RNASE1 |
| RBFOX2 | RNA Binding Fox-1 Homolog 2 | Protein Coding | 40 | GC22M035738 | 0.346795946 | https://www.genecards.org/cgi-bin/carddisp.pl?gene=RBFOX2 |
| USP30 | Ubiquitin Specific Peptidase 30 | Protein Coding | 40 | GC12P109027 | 0.346795946 | https://www.genecards.org/cgi-bin/carddisp.pl?gene=USP30 |
| SUPT4H1 | SPT4 Homolog, DSIF Elongation Factor Subunit | Protein Coding | 40 | GC17M058345 | 0.346795946 | https://www.genecards.org/cgi-bin/carddisp.pl?gene=SUPT4H1 |
| TOMM20 | Translocase Of Outer Mitochondrial Membrane 20 | Protein Coding | 40 | GC01M235109 | 0.346795946 | https://www.genecards.org/cgi-bin/carddisp.pl?gene=TOMM20 |
| SRSF6 | Serine And Arginine Rich Splicing Factor 6 | Protein Coding | 40 | GC20P043457 | 0.346795946 | https://www.genecards.org/cgi-bin/carddisp.pl?gene=SRSF6 |
| POLR3K | RNA Polymerase III Subunit K | Protein Coding | 40 | GC16M000046 | 0.346795946 | https://www.genecards.org/cgi-bin/carddisp.pl?gene=POLR3K |
| POU3F4 | POU Class 3 Homeobox 4 | Protein Coding | 40 | GC0XP083508 | 0.346795946 | https://www.genecards.org/cgi-bin/carddisp.pl?gene=POU3F4 |
| PRKN | Parkin RBR E3 Ubiquitin Protein Ligase | Protein Coding | 40 | GC06M161348 | 0.346795946 | https://www.genecards.org/cgi-bin/carddisp.pl?gene=PRKN |
| PCTP | Phosphatidylcholine Transfer Protein | Protein Coding | 40 | GC17P055750 | 0.346795946 | https://www.genecards.org/cgi-bin/carddisp.pl?gene=PCTP |
| NPRL2 | NPR2 Like, GATOR1 Complex Subunit | Protein Coding | 40 | GC03M050432 | 0.346795946 | https://www.genecards.org/cgi-bin/carddisp.pl?gene=NPRL2 |
| PTPRH | Protein Tyrosine Phosphatase Receptor Type H | Protein Coding | 40 | GC19M055181 | 0.346795946 | https://www.genecards.org/cgi-bin/carddisp.pl?gene=PTPRH |
| NAIP | NLR Family Apoptosis Inhibitory Protein | Protein Coding | 40 | GC05M070968 | 0.346795946 | https://www.genecards.org/cgi-bin/carddisp.pl?gene=NAIP |
| LGR4 | Leucine Rich Repeat Containing G Protein-Coupled Receptor 4 | Protein Coding | 40 | GC11M027365 | 0.346795946 | https://www.genecards.org/cgi-bin/carddisp.pl?gene=LGR4 |
| AFAP1 | Actin Filament Associated Protein 1 | Protein Coding | 39 | GC04M007758 | 0.346795946 | https://www.genecards.org/cgi-bin/carddisp.pl?gene=AFAP1 |
| BCL2L13 | BCL2 Like 13 | Protein Coding | 39 | GC22P017628 | 0.346795946 | https://www.genecards.org/cgi-bin/carddisp.pl?gene=BCL2L13 |
| LZTS1 | Leucine Zipper Tumor Suppressor 1 | Protein Coding | 39 | GC08M020246 | 0.346795946 | https://www.genecards.org/cgi-bin/carddisp.pl?gene=LZTS1 |
| FIS1 | Fission, Mitochondrial 1 | Protein Coding | 39 | GC07M101239 | 0.346795946 | https://www.genecards.org/cgi-bin/carddisp.pl?gene=FIS1 |
| BBS9 | Bardet-Biedl Syndrome 9 | Protein Coding | 39 | GC07P033112 | 0.346795946 | https://www.genecards.org/cgi-bin/carddisp.pl?gene=BBS9 |
| DHX58 | DExH-Box Helicase 58 | Protein Coding | 39 | GC17M042101 | 0.346795946 | https://www.genecards.org/cgi-bin/carddisp.pl?gene=DHX58 |
| ACER3 | Alkaline Ceramidase 3 | Protein Coding | 39 | GC11P076860 | 0.346795946 | https://www.genecards.org/cgi-bin/carddisp.pl?gene=ACER3 |
| ACOT12 | Acyl-CoA Thioesterase 12 | Protein Coding | 39 | GC05M081309 | 0.346795946 | https://www.genecards.org/cgi-bin/carddisp.pl?gene=ACOT12 |
| ANKRD26 | Ankyrin Repeat Domain 26 | Protein Coding | 39 | GC10M026938 | 0.346795946 | https://www.genecards.org/cgi-bin/carddisp.pl?gene=ANKRD26 |
| MRPS7 | Mitochondrial Ribosomal Protein S7 | Protein Coding | 39 | GC17P075262 | 0.346795946 | https://www.genecards.org/cgi-bin/carddisp.pl?gene=MRPS7 |
| TSEN34 | TRNA Splicing Endonuclease Subunit 34 | Protein Coding | 39 | GC19P056807 | 0.346795946 | https://www.genecards.org/cgi-bin/carddisp.pl?gene=TSEN34 |
| STRADB | STE20 Related Adaptor Beta | Protein Coding | 39 | GC02P201387 | 0.346795946 | https://www.genecards.org/cgi-bin/carddisp.pl?gene=STRADB |
| SUMO4 | Small Ubiquitin Like Modifier 4 | Protein Coding | 39 | GC06P149401 | 0.346795946 | https://www.genecards.org/cgi-bin/carddisp.pl?gene=SUMO4 |
| SLC25A37 | Solute Carrier Family 25 Member 37 | Protein Coding | 39 | GC08P023528 | 0.346795946 | https://www.genecards.org/cgi-bin/carddisp.pl?gene=SLC25A37 |
| SLC6A18 | Solute Carrier Family 6 Member 18 | Protein Coding | 39 | GC05P001225 | 0.346795946 | https://www.genecards.org/cgi-bin/carddisp.pl?gene=SLC6A18 |
| MUL1 | Mitochondrial E3 Ubiquitin Protein Ligase 1 | Protein Coding | 39 | GC01M020499 | 0.346795946 | https://www.genecards.org/cgi-bin/carddisp.pl?gene=MUL1 |
| PCDH10 | Protocadherin 10 | Protein Coding | 39 | GC04P133149 | 0.346795946 | https://www.genecards.org/cgi-bin/carddisp.pl?gene=PCDH10 |
| SLC36A1 | Solute Carrier Family 36 Member 1 | Protein Coding | 39 | GC05P151413 | 0.346795946 | https://www.genecards.org/cgi-bin/carddisp.pl?gene=SLC36A1 |
| COPS7B | COP9 Signalosome Subunit 7B | Protein Coding | 38 | GC02P231781 | 0.346795946 | https://www.genecards.org/cgi-bin/carddisp.pl?gene=COPS7B |
| CEBPD | CCAAT Enhancer Binding Protein Delta | Protein Coding | 38 | GC08M047759 | 0.346795946 | https://www.genecards.org/cgi-bin/carddisp.pl?gene=CEBPD |
| COL15A1 | Collagen Type XV Alpha 1 Chain | Protein Coding | 38 | GC09P098943 | 0.346795946 | https://www.genecards.org/cgi-bin/carddisp.pl?gene=COL15A1 |
| DENR | Density Regulated Re-Initiation And Release Factor | Protein Coding | 38 | GC12P122752 | 0.346795946 | https://www.genecards.org/cgi-bin/carddisp.pl?gene=DENR |
| CCL17 | C-C Motif Chemokine Ligand 17 | Protein Coding | 38 | GC16P057403 | 0.346795946 | https://www.genecards.org/cgi-bin/carddisp.pl?gene=CCL17 |
| IQSEC2 | IQ Motif And Sec7 Domain ArfGEF 2 | Protein Coding | 38 | GC0XM053225 | 0.346795946 | https://www.genecards.org/cgi-bin/carddisp.pl?gene=IQSEC2 |
| GIMAP4 | GTPase, IMAP Family Member 4 | Protein Coding | 38 | GC07P150568 | 0.346795946 | https://www.genecards.org/cgi-bin/carddisp.pl?gene=GIMAP4 |
| MFF | Mitochondrial Fission Factor | Protein Coding | 38 | GC02P227325 | 0.346795946 | https://www.genecards.org/cgi-bin/carddisp.pl?gene=MFF |
| HSPA12A | Heat Shock Protein Family A (Hsp70) Member 12A | Protein Coding | 38 | GC10M116671 | 0.346795946 | https://www.genecards.org/cgi-bin/carddisp.pl?gene=HSPA12A |
| IBSP | Integrin Binding Sialoprotein | Protein Coding | 38 | GC04P087799 | 0.346795946 | https://www.genecards.org/cgi-bin/carddisp.pl?gene=IBSP |
| LRRFIP2 | LRR Binding FLII Interacting Protein 2 | Protein Coding | 38 | GC03M037052 | 0.346795946 | https://www.genecards.org/cgi-bin/carddisp.pl?gene=LRRFIP2 |
| ERAP2 | Endoplasmic Reticulum Aminopeptidase 2 | Protein Coding | 38 | GC05P096875 | 0.346795946 | https://www.genecards.org/cgi-bin/carddisp.pl?gene=ERAP2 |
| ACOT13 | Acyl-CoA Thioesterase 13 | Protein Coding | 38 | GC06P024667 | 0.346795946 | https://www.genecards.org/cgi-bin/carddisp.pl?gene=ACOT13 |
| CHCHD3 | Coiled-Coil-Helix-Coiled-Coil-Helix Domain Containing 3 | Protein Coding | 38 | GC07M132784 | 0.346795946 | https://www.genecards.org/cgi-bin/carddisp.pl?gene=CHCHD3 |
| DYNLT1 | Dynein Light Chain Tctex-Type 1 | Protein Coding | 38 | GC06M158636 | 0.346795946 | https://www.genecards.org/cgi-bin/carddisp.pl?gene=DYNLT1 |
| ELP2 | Elongator Acetyltransferase Complex Subunit 2 | Protein Coding | 38 | GC18P036129 | 0.346795946 | https://www.genecards.org/cgi-bin/carddisp.pl?gene=ELP2 |
| EIF1 | Eukaryotic Translation Initiation Factor 1 | Protein Coding | 38 | GC17P041688 | 0.346795946 | https://www.genecards.org/cgi-bin/carddisp.pl?gene=EIF1 |
| OTUD7A | OTU Deubiquitinase 7A | Protein Coding | 38 | GC15M031475 | 0.346795946 | https://www.genecards.org/cgi-bin/carddisp.pl?gene=OTUD7A |
| SYNGR2 | Synaptogyrin 2 | Protein Coding | 38 | GC17P078168 | 0.346795946 | https://www.genecards.org/cgi-bin/carddisp.pl?gene=SYNGR2 |
| RNF43 | Ring Finger Protein 43 | Protein Coding | 38 | GC17M058352 | 0.346795946 | https://www.genecards.org/cgi-bin/carddisp.pl?gene=RNF43 |
| UTP6 | UTP6 Small Subunit Processome Component | Protein Coding | 38 | GC17M031860 | 0.346795946 | https://www.genecards.org/cgi-bin/carddisp.pl?gene=UTP6 |
| ZFPM1 | Zinc Finger Protein, FOG Family Member 1 | Protein Coding | 38 | GC16P088451 | 0.346795946 | https://www.genecards.org/cgi-bin/carddisp.pl?gene=ZFPM1 |
| ST18 | ST18 C2H2C-Type Zinc Finger Transcription Factor | Protein Coding | 38 | GC08M052110 | 0.346795946 | https://www.genecards.org/cgi-bin/carddisp.pl?gene=ST18 |
| PKP3 | Plakophilin 3 | Protein Coding | 38 | GC11P000396 | 0.346795946 | https://www.genecards.org/cgi-bin/carddisp.pl?gene=PKP3 |
| PLEKHA7 | Pleckstrin Homology Domain Containing A7 | Protein Coding | 38 | GC11M016778 | 0.346795946 | https://www.genecards.org/cgi-bin/carddisp.pl?gene=PLEKHA7 |
| PLVAP | Plasmalemma Vesicle Associated Protein | Protein Coding | 38 | GC19M017351 | 0.346795946 | https://www.genecards.org/cgi-bin/carddisp.pl?gene=PLVAP |
| PPP1R15B | Protein Phosphatase 1 Regulatory Subunit 15B | Protein Coding | 38 | GC01M204400 | 0.346795946 | https://www.genecards.org/cgi-bin/carddisp.pl?gene=PPP1R15B |
| POU2AF1 | POU Class 2 Homeobox Associating Factor 1 | Protein Coding | 38 | GC11M111352 | 0.346795946 | https://www.genecards.org/cgi-bin/carddisp.pl?gene=POU2AF1 |
| SLC22A14 | Solute Carrier Family 22 Member 14 | Protein Coding | 38 | GC03P038281 | 0.346795946 | https://www.genecards.org/cgi-bin/carddisp.pl?gene=SLC22A14 |
| SMAP2 | Small ArfGAP2 | Protein Coding | 38 | GC01P040344 | 0.346795946 | https://www.genecards.org/cgi-bin/carddisp.pl?gene=SMAP2 |
| OTULIN | OTU Deubiquitinase With Linear Linkage Specificity | Protein Coding | 38 | GC05P014667 | 0.346795946 | https://www.genecards.org/cgi-bin/carddisp.pl?gene=OTULIN |
| NPRL3 | NPR3 Like, GATOR1 Complex Subunit | Protein Coding | 38 | GC16M000084 | 0.346795946 | https://www.genecards.org/cgi-bin/carddisp.pl?gene=NPRL3 |
| OMA1 | OMA1 Zinc Metallopeptidase | Protein Coding | 38 | GC01M058415 | 0.346795946 | https://www.genecards.org/cgi-bin/carddisp.pl?gene=OMA1 |
| NLRP6 | NLR Family Pyrin Domain Containing 6 | Protein Coding | 38 | GC11P000269 | 0.346795946 | https://www.genecards.org/cgi-bin/carddisp.pl?gene=NLRP6 |
| QTRT1 | Queuine TRNA-Ribosyltransferase Catalytic Subunit 1 | Protein Coding | 38 | GC19P010701 | 0.346795946 | https://www.genecards.org/cgi-bin/carddisp.pl?gene=QTRT1 |
| RAB11FIP4 | RAB11 Family Interacting Protein 4 | Protein Coding | 38 | GC17P031391 | 0.346795946 | https://www.genecards.org/cgi-bin/carddisp.pl?gene=RAB11FIP4 |
| PUS3 | Pseudouridine Synthase 3 | Protein Coding | 38 | GC11M125893 | 0.346795946 | https://www.genecards.org/cgi-bin/carddisp.pl?gene=PUS3 |
| ARHGAP11A | Rho GTPase Activating Protein 11A | Protein Coding | 37 | GC15P032615 | 0.346795946 | https://www.genecards.org/cgi-bin/carddisp.pl?gene=ARHGAP11A |
| ANXA10 | Annexin A10 | Protein Coding | 37 | GC04P168081 | 0.346795946 | https://www.genecards.org/cgi-bin/carddisp.pl?gene=ANXA10 |
| IMMP1L | Inner Mitochondrial Membrane Peptidase Subunit 1 | Protein Coding | 37 | GC11M031432 | 0.346795946 | https://www.genecards.org/cgi-bin/carddisp.pl?gene=IMMP1L |
| IL37 | Interleukin 37 | Protein Coding | 37 | GC02P116690 | 0.346795946 | https://www.genecards.org/cgi-bin/carddisp.pl?gene=IL37 |
| KLC4 | Kinesin Light Chain 4 | Protein Coding | 37 | GC06P043040 | 0.346795946 | https://www.genecards.org/cgi-bin/carddisp.pl?gene=KLC4 |
| LAX1 | Lymphocyte Transmembrane Adaptor 1 | Protein Coding | 37 | GC01P203768 | 0.346795946 | https://www.genecards.org/cgi-bin/carddisp.pl?gene=LAX1 |
| KIR2DL1 | Killer Cell Immunoglobulin Like Receptor, Two Ig Domains And Long Cytoplasmic Tail 1 | Protein Coding | 37 | GC19P056443 | 0.346795946 | https://www.genecards.org/cgi-bin/carddisp.pl?gene=KIR2DL1 |
| ERCC6L | ERCC Excision Repair 6 Like, Spindle Assembly Checkpoint Helicase | Protein Coding | 37 | GC0XM072204 | 0.346795946 | https://www.genecards.org/cgi-bin/carddisp.pl?gene=ERCC6L |
| CFHR2 | Complement Factor H Related 2 | Protein Coding | 37 | GC01P196943 | 0.346795946 | https://www.genecards.org/cgi-bin/carddisp.pl?gene=CFHR2 |
| CH25H | Cholesterol 25-Hydroxylase | Protein Coding | 37 | GC10M089205 | 0.346795946 | https://www.genecards.org/cgi-bin/carddisp.pl?gene=CH25H |
| C1GALT1C1 | C1GALT1 Specific Chaperone 1 | Protein Coding | 37 | GC0XM120625 | 0.346795946 | https://www.genecards.org/cgi-bin/carddisp.pl?gene=C1GALT1C1 |
| CCL18 | C-C Motif Chemokine Ligand 18 | Protein Coding | 37 | GC17P036064 | 0.346795946 | https://www.genecards.org/cgi-bin/carddisp.pl?gene=CCL18 |
| CRLF3 | Cytokine Receptor Like Factor 3 | Protein Coding | 37 | GC17M030769 | 0.346795946 | https://www.genecards.org/cgi-bin/carddisp.pl?gene=CRLF3 |
| ZNF408 | Zinc Finger Protein 408 | Protein Coding | 37 | GC11P046700 | 0.346795946 | https://www.genecards.org/cgi-bin/carddisp.pl?gene=ZNF408 |
| TBC1D10C | TBC1 Domain Family Member 10C | Protein Coding | 37 | GC11P067403 | 0.346795946 | https://www.genecards.org/cgi-bin/carddisp.pl?gene=TBC1D10C |
| STAC2 | SH3 And Cysteine Rich Domain 2 | Protein Coding | 37 | GC17M039236 | 0.346795946 | https://www.genecards.org/cgi-bin/carddisp.pl?gene=STAC2 |
| STAC3 | SH3 And Cysteine Rich Domain 3 | Protein Coding | 37 | GC12M057243 | 0.346795946 | https://www.genecards.org/cgi-bin/carddisp.pl?gene=STAC3 |
| MCU | Mitochondrial Calcium Uniporter | Protein Coding | 37 | GC10P072692 | 0.346795946 | https://www.genecards.org/cgi-bin/carddisp.pl?gene=MCU |
| PIF1 | PIF1 5'-To-3' DNA Helicase | Protein Coding | 37 | GC15M064815 | 0.346795946 | https://www.genecards.org/cgi-bin/carddisp.pl?gene=PIF1 |
| SLC9A5 | Solute Carrier Family 9 Member A5 | Protein Coding | 37 | GC16P067240 | 0.346795946 | https://www.genecards.org/cgi-bin/carddisp.pl?gene=SLC9A5 |
| SLC38A8 | Solute Carrier Family 38 Member 8 | Protein Coding | 37 | GC16M084009 | 0.346795946 | https://www.genecards.org/cgi-bin/carddisp.pl?gene=SLC38A8 |
| SETD6 | SET Domain Containing 6, Protein Lysine Methyltransferase | Protein Coding | 37 | GC16P058514 | 0.346795946 | https://www.genecards.org/cgi-bin/carddisp.pl?gene=SETD6 |
| PCMTD1 | Protein-L-Isoaspartate (D-Aspartate) O-Methyltransferase Domain Containing 1 | Protein Coding | 37 | GC08M051817 | 0.346795946 | https://www.genecards.org/cgi-bin/carddisp.pl?gene=PCMTD1 |
| OR11A1 | Olfactory Receptor Family 11 Subfamily A Member 1 | Protein Coding | 37 | GC06M029425 | 0.346795946 | https://www.genecards.org/cgi-bin/carddisp.pl?gene=OR11A1 |
| RINT1 | RAD50 Interactor 1 | Protein Coding | 37 | GC07P105532 | 0.346795946 | https://www.genecards.org/cgi-bin/carddisp.pl?gene=RINT1 |
| NAALADL2 | N-Acetylated Alpha-Linked Acidic Dipeptidase Like 2 | Protein Coding | 37 | GC03P174438 | 0.346795946 | https://www.genecards.org/cgi-bin/carddisp.pl?gene=NAALADL2 |
| SNX13 | Sorting Nexin 13 | Protein Coding | 37 | GC07M017798 | 0.346795946 | https://www.genecards.org/cgi-bin/carddisp.pl?gene=SNX13 |
| TNFSF8 | TNF Superfamily Member 8 | Protein Coding | 37 | GC09M114893 | 0.346795946 | https://www.genecards.org/cgi-bin/carddisp.pl?gene=TNFSF8 |
| FUNDC1 | FUN14 Domain Containing 1 | Protein Coding | 36 | GC0XM044523 | 0.346795946 | https://www.genecards.org/cgi-bin/carddisp.pl?gene=FUNDC1 |
| COX14 | Cytochrome C Oxidase Assembly Factor COX14 | Protein Coding | 36 | GC12P050111 | 0.346795946 | https://www.genecards.org/cgi-bin/carddisp.pl?gene=COX14 |
| ATP13A4 | ATPase 13A4 | Protein Coding | 36 | GC03M193401 | 0.346795946 | https://www.genecards.org/cgi-bin/carddisp.pl?gene=ATP13A4 |
| H3-3A | H3.3 Histone A | Protein Coding | 36 | GC01P226062 | 0.346795946 | https://www.genecards.org/cgi-bin/carddisp.pl?gene=H3-3A |
| GIMAP2 | GTPase, IMAP Family Member 2 | Protein Coding | 36 | GC07P150686 | 0.346795946 | https://www.genecards.org/cgi-bin/carddisp.pl?gene=GIMAP2 |
| GPHA2 | Glycoprotein Hormone Subunit Alpha 2 | Protein Coding | 36 | GC11M069353 | 0.346795946 | https://www.genecards.org/cgi-bin/carddisp.pl?gene=GPHA2 |
| NELFCD | Negative Elongation Factor Complex Member C/D | Protein Coding | 36 | GC20P058981 | 0.346795946 | https://www.genecards.org/cgi-bin/carddisp.pl?gene=NELFCD |
| CARD16 | Caspase Recruitment Domain Family Member 16 | Protein Coding | 36 | GC11M105041 | 0.346795946 | https://www.genecards.org/cgi-bin/carddisp.pl?gene=CARD16 |
| EFHC2 | EF-Hand Domain Containing 2 | Protein Coding | 36 | GC0XM044146 | 0.346795946 | https://www.genecards.org/cgi-bin/carddisp.pl?gene=EFHC2 |
| ADAP2 | ArfGAP With Dual PH Domains 2 | Protein Coding | 36 | GC17P030906 | 0.346795946 | https://www.genecards.org/cgi-bin/carddisp.pl?gene=ADAP2 |
| DAND5 | DAN Domain BMP Antagonist Family Member 5 | Protein Coding | 36 | GC19P012965 | 0.346795946 | https://www.genecards.org/cgi-bin/carddisp.pl?gene=DAND5 |
| EPDR1 | Ependymin Related 1 | Protein Coding | 36 | GC07P037694 | 0.346795946 | https://www.genecards.org/cgi-bin/carddisp.pl?gene=EPDR1 |
| EPM2AIP1 | EPM2A Interacting Protein 1 | Protein Coding | 36 | GC03M037006 | 0.346795946 | https://www.genecards.org/cgi-bin/carddisp.pl?gene=EPM2AIP1 |
| VIT | Vitrin | Protein Coding | 36 | GC02P036696 | 0.346795946 | https://www.genecards.org/cgi-bin/carddisp.pl?gene=VIT |
| SYNPO2 | Synaptopodin 2 | Protein Coding | 36 | GC04P118850 | 0.346795946 | https://www.genecards.org/cgi-bin/carddisp.pl?gene=SYNPO2 |
| PTPA | Protein Phosphatase 2 Phosphatase Activator | Protein Coding | 36 | GC09P129111 | 0.346795946 | https://www.genecards.org/cgi-bin/carddisp.pl?gene=PTPA |
| SLC25A45 | Solute Carrier Family 25 Member 45 | Protein Coding | 36 | GC11M065375 | 0.346795946 | https://www.genecards.org/cgi-bin/carddisp.pl?gene=SLC25A45 |
| SEC22B | SEC22 Homolog B, Vesicle Trafficking Protein | Protein Coding | 36 | GC01M120150 | 0.346795946 | https://www.genecards.org/cgi-bin/carddisp.pl?gene=SEC22B |
| MDGA2 | MAM Domain Containing Glycosylphosphatidylinositol Anchor 2 | Protein Coding | 36 | GC14M046839 | 0.346795946 | https://www.genecards.org/cgi-bin/carddisp.pl?gene=MDGA2 |
| PTCHD1 | Patched Domain Containing 1 | Protein Coding | 36 | GC0XP023334 | 0.346795946 | https://www.genecards.org/cgi-bin/carddisp.pl?gene=PTCHD1 |
| FCGBP | Fc Fragment Of IgG Binding Protein | Protein Coding | 35 | GC19M039863 | 0.346795946 | https://www.genecards.org/cgi-bin/carddisp.pl?gene=FCGBP |
| LRIT1 | Leucine Rich Repeat, Ig-Like And Transmembrane Domains 1 | Protein Coding | 35 | GC10M084231 | 0.346795946 | https://www.genecards.org/cgi-bin/carddisp.pl?gene=LRIT1 |
| EVI2A | Ecotropic Viral Integration Site 2A | Protein Coding | 35 | GC17M031316 | 0.346795946 | https://www.genecards.org/cgi-bin/carddisp.pl?gene=EVI2A |
| CCDC136 | Coiled-Coil Domain Containing 136 | Protein Coding | 35 | GC07P130050 | 0.346795946 | https://www.genecards.org/cgi-bin/carddisp.pl?gene=CCDC136 |
| CCDC148 | Coiled-Coil Domain Containing 148 | Protein Coding | 35 | GC02M158171 | 0.346795946 | https://www.genecards.org/cgi-bin/carddisp.pl?gene=CCDC148 |
| CMIP | C-Maf Inducing Protein | Protein Coding | 35 | GC16P081445 | 0.346795946 | https://www.genecards.org/cgi-bin/carddisp.pl?gene=CMIP |
| CTU1 | Cytosolic Thiouridylase Subunit 1 | Protein Coding | 35 | GC19M051097 | 0.346795946 | https://www.genecards.org/cgi-bin/carddisp.pl?gene=CTU1 |
| ADAT3 | Adenosine Deaminase TRNA Specific 3 | Protein Coding | 35 | GC19P001906 | 0.346795946 | https://www.genecards.org/cgi-bin/carddisp.pl?gene=ADAT3 |
| DNAJC24 | DnaJ Heat Shock Protein Family (Hsp40) Member C24 | Protein Coding | 35 | GC11P031349 | 0.346795946 | https://www.genecards.org/cgi-bin/carddisp.pl?gene=DNAJC24 |
| YPEL1 | Yippee Like 1 | Protein Coding | 35 | GC22M021697 | 0.346795946 | https://www.genecards.org/cgi-bin/carddisp.pl?gene=YPEL1 |
| ORMDL1 | ORMDL Sphingolipid Biosynthesis Regulator 1 | Protein Coding | 35 | GC02M189764 | 0.346795946 | https://www.genecards.org/cgi-bin/carddisp.pl?gene=ORMDL1 |
| MALL | Mal, T Cell Differentiation Protein Like | Protein Coding | 34 | GC02M110083 | 0.346795946 | https://www.genecards.org/cgi-bin/carddisp.pl?gene=MALL |
| GDAP1L1 | Ganglioside Induced Differentiation Associated Protein 1 Like 1 | Protein Coding | 34 | GC20P044247 | 0.346795946 | https://www.genecards.org/cgi-bin/carddisp.pl?gene=GDAP1L1 |
| MIEF2 | Mitochondrial Elongation Factor 2 | Protein Coding | 34 | GC17P018262 | 0.346795946 | https://www.genecards.org/cgi-bin/carddisp.pl?gene=MIEF2 |
| IFNA4 | Interferon Alpha 4 | Protein Coding | 34 | GC09M021186 | 0.346795946 | https://www.genecards.org/cgi-bin/carddisp.pl?gene=IFNA4 |
| LUZP4 | Leucine Zipper Protein 4 | Protein Coding | 34 | GC0XP115289 | 0.346795946 | https://www.genecards.org/cgi-bin/carddisp.pl?gene=LUZP4 |
| GCFC2 | GC-Rich Sequence DNA-Binding Factor 2 | Protein Coding | 34 | GC02M075652 | 0.346795946 | https://www.genecards.org/cgi-bin/carddisp.pl?gene=GCFC2 |
| GET4 | Guided Entry Of Tail-Anchored Proteins Factor 4 | Protein Coding | 34 | GC07P000876 | 0.346795946 | https://www.genecards.org/cgi-bin/carddisp.pl?gene=GET4 |
| ATAD5 | ATPase Family AAA Domain Containing 5 | Protein Coding | 34 | GC17P030832 | 0.346795946 | https://www.genecards.org/cgi-bin/carddisp.pl?gene=ATAD5 |
| ABHD16A | Abhydrolase Domain Containing 16A, Phospholipase | Protein Coding | 34 | GC06M046888 | 0.346795946 | https://www.genecards.org/cgi-bin/carddisp.pl?gene=ABHD16A |
| CAMSAP2 | Calmodulin Regulated Spectrin Associated Protein Family Member 2 | Protein Coding | 34 | GC01P200739 | 0.346795946 | https://www.genecards.org/cgi-bin/carddisp.pl?gene=CAMSAP2 |
| BRWD3 | Bromodomain And WD Repeat Domain Containing 3 | Protein Coding | 34 | GC0XM080669 | 0.346795946 | https://www.genecards.org/cgi-bin/carddisp.pl?gene=BRWD3 |
| ELP5 | Elongator Acetyltransferase Complex Subunit 5 | Protein Coding | 34 | GC17P007251 | 0.346795946 | https://www.genecards.org/cgi-bin/carddisp.pl?gene=ELP5 |
| ELP1 | Elongator Acetyltransferase Complex Subunit 1 | Protein Coding | 34 | GC09M108868 | 0.346795946 | https://www.genecards.org/cgi-bin/carddisp.pl?gene=ELP1 |
| MTMR10 | Myotubularin Related Protein 10 | Protein Coding | 34 | GC15M030919 | 0.346795946 | https://www.genecards.org/cgi-bin/carddisp.pl?gene=MTMR10 |
| RNF186 | Ring Finger Protein 186 | Protein Coding | 34 | GC01M019814 | 0.346795946 | https://www.genecards.org/cgi-bin/carddisp.pl?gene=RNF186 |
| URM1 | Ubiquitin Related Modifier 1 | Protein Coding | 34 | GC09P128371 | 0.346795946 | https://www.genecards.org/cgi-bin/carddisp.pl?gene=URM1 |
| PRSS36 | Serine Protease 36 | Protein Coding | 34 | GC16M031155 | 0.346795946 | https://www.genecards.org/cgi-bin/carddisp.pl?gene=PRSS36 |
| SLC25A2 | Solute Carrier Family 25 Member 2 | Protein Coding | 34 | GC05M141302 | 0.346795946 | https://www.genecards.org/cgi-bin/carddisp.pl?gene=SLC25A2 |
| PGAM5 | PGAM Family Member 5, Mitochondrial Serine/Threonine Protein Phosphatase | Protein Coding | 34 | GC12P132710 | 0.346795946 | https://www.genecards.org/cgi-bin/carddisp.pl?gene=PGAM5 |
| PAGR1 | PAXIP1 Associated Glutamate Rich Protein 1 | Protein Coding | 34 | GC16P032379 | 0.346795946 | https://www.genecards.org/cgi-bin/carddisp.pl?gene=PAGR1 |
| TMPRSS9 | Transmembrane Serine Protease 9 | Protein Coding | 34 | GC19P002360 | 0.346795946 | https://www.genecards.org/cgi-bin/carddisp.pl?gene=TMPRSS9 |
| COL6A5 | Collagen Type VI Alpha 5 Chain | Protein Coding | 33 | GC03P130345 | 0.346795946 | https://www.genecards.org/cgi-bin/carddisp.pl?gene=COL6A5 |
| MIEF1 | Mitochondrial Elongation Factor 1 | Protein Coding | 33 | GC22P039510 | 0.346795946 | https://www.genecards.org/cgi-bin/carddisp.pl?gene=MIEF1 |
| KCP | Kielin Cysteine Rich BMP Regulator | Protein Coding | 33 | GC07M128862 | 0.346795946 | https://www.genecards.org/cgi-bin/carddisp.pl?gene=KCP |
| LRRC37B | Leucine Rich Repeat Containing 37B | Protein Coding | 33 | GC17P032008 | 0.346795946 | https://www.genecards.org/cgi-bin/carddisp.pl?gene=LRRC37B |
| JRKL | JRK Like | Protein Coding | 33 | GC11P096389 | 0.346795946 | https://www.genecards.org/cgi-bin/carddisp.pl?gene=JRKL |
| HMX2 | H6 Family Homeobox 2 | Protein Coding | 33 | GC10P123142 | 0.346795946 | https://www.genecards.org/cgi-bin/carddisp.pl?gene=HMX2 |
| FERD3L | Fer3 Like BHLH Transcription Factor | Protein Coding | 33 | GC07M019144 | 0.346795946 | https://www.genecards.org/cgi-bin/carddisp.pl?gene=FERD3L |
| EVI2B | Ecotropic Viral Integration Site 2B | Protein Coding | 33 | GC17M031405 | 0.346795946 | https://www.genecards.org/cgi-bin/carddisp.pl?gene=EVI2B |
| FAM102A | Family With Sequence Similarity 102 Member A | Protein Coding | 33 | GC09M127941 | 0.346795946 | https://www.genecards.org/cgi-bin/carddisp.pl?gene=FAM102A |
| BEAN1 | Brain Expressed Associated With NEDD4 1 | Protein Coding | 33 | GC16P066428 | 0.346795946 | https://www.genecards.org/cgi-bin/carddisp.pl?gene=BEAN1 |
| C16orf54 | Chromosome 16 Open Reading Frame 54 | Protein Coding | 33 | GC16M031146 | 0.346795946 | https://www.genecards.org/cgi-bin/carddisp.pl?gene=C16orf54 |
| CHRFAM7A | CHRNA7 (Exons 5-10) And FAM7A (Exons A-E) Fusion | Protein Coding | 33 | GC15M030360 | 0.346795946 | https://www.genecards.org/cgi-bin/carddisp.pl?gene=CHRFAM7A |
| ELP6 | Elongator Acetyltransferase Complex Subunit 6 | Protein Coding | 33 | GC03M047495 | 0.346795946 | https://www.genecards.org/cgi-bin/carddisp.pl?gene=ELP6 |
| ZNF221 | Zinc Finger Protein 221 | Protein Coding | 33 | GC19P043951 | 0.346795946 | https://www.genecards.org/cgi-bin/carddisp.pl?gene=ZNF221 |
| ZNF385D | Zinc Finger Protein 385D | Protein Coding | 33 | GC03M021412 | 0.346795946 | https://www.genecards.org/cgi-bin/carddisp.pl?gene=ZNF385D |
| TMEM186 | Transmembrane Protein 186 | Protein Coding | 33 | GC16M008780 | 0.346795946 | https://www.genecards.org/cgi-bin/carddisp.pl?gene=TMEM186 |
| TTLL11 | Tubulin Tyrosine Ligase Like 11 | Protein Coding | 33 | GC09M121816 | 0.346795946 | https://www.genecards.org/cgi-bin/carddisp.pl?gene=TTLL11 |
| WBP1L | WW Domain Binding Protein 1 Like | Protein Coding | 33 | GC10P102743 | 0.346795946 | https://www.genecards.org/cgi-bin/carddisp.pl?gene=WBP1L |
| MTFP1 | Mitochondrial Fission Process 1 | Protein Coding | 33 | GC22P030426 | 0.346795946 | https://www.genecards.org/cgi-bin/carddisp.pl?gene=MTFP1 |
| SKOR1 | SKI Family Transcriptional Corepressor 1 | Protein Coding | 33 | GC15P067819 | 0.346795946 | https://www.genecards.org/cgi-bin/carddisp.pl?gene=SKOR1 |
| PHYHD1 | Phytanoyl-CoA Dioxygenase Domain Containing 1 | Protein Coding | 33 | GC09P128920 | 0.346795946 | https://www.genecards.org/cgi-bin/carddisp.pl?gene=PHYHD1 |
| SCNM1 | Sodium Channel Modifier 1 | Protein Coding | 33 | GC01P151159 | 0.346795946 | https://www.genecards.org/cgi-bin/carddisp.pl?gene=SCNM1 |
| PRSS53 | Serine Protease 53 | Protein Coding | 33 | GC16M031093 | 0.346795946 | https://www.genecards.org/cgi-bin/carddisp.pl?gene=PRSS53 |
| DPH3 | Diphthamide Biosynthesis 3 | Protein Coding | 32 | GC03M016257 | 0.346795946 | https://www.genecards.org/cgi-bin/carddisp.pl?gene=DPH3 |
| ASPHD1 | Aspartate Beta-Hydroxylase Domain Containing 1 | Protein Coding | 32 | GC16P032383 | 0.346795946 | https://www.genecards.org/cgi-bin/carddisp.pl?gene=ASPHD1 |
| MARCHF5 | Membrane Associated Ring-CH-Type Finger 5 | Protein Coding | 32 | GC10P092292 | 0.346795946 | https://www.genecards.org/cgi-bin/carddisp.pl?gene=MARCHF5 |
| SEPTIN5 | Septin 5 | Protein Coding | 32 | GC22P019714 | 0.346795946 | https://www.genecards.org/cgi-bin/carddisp.pl?gene=SEPTIN5 |
| OR10J1 | Olfactory Receptor Family 10 Subfamily J Member 1 | Protein Coding | 32 | GC01P159406 | 0.346795946 | https://www.genecards.org/cgi-bin/carddisp.pl?gene=OR10J1 |
| OR52B2 | Olfactory Receptor Family 52 Subfamily B Member 2 | Protein Coding | 32 | GC11M006169 | 0.346795946 | https://www.genecards.org/cgi-bin/carddisp.pl?gene=OR52B2 |
| PRSS58 | Serine Protease 58 | Protein Coding | 32 | GC07M142263 | 0.346795946 | https://www.genecards.org/cgi-bin/carddisp.pl?gene=PRSS58 |
| COPRS | Coordinator Of PRMT5 And Differentiation Stimulator | Protein Coding | 31 | GC17M031851 | 0.346795946 | https://www.genecards.org/cgi-bin/carddisp.pl?gene=COPRS |
| DNAAF4 | Dynein Axonemal Assembly Factor 4 | Protein Coding | 31 | GC15M067450 | 0.346795946 | https://www.genecards.org/cgi-bin/carddisp.pl?gene=DNAAF4 |
| DCDC1 | Doublecortin Domain Containing 1 | Protein Coding | 31 | GC11M030851 | 0.346795946 | https://www.genecards.org/cgi-bin/carddisp.pl?gene=DCDC1 |
| BEND6 | BEN Domain Containing 6 | Protein Coding | 31 | GC06P056954 | 0.346795946 | https://www.genecards.org/cgi-bin/carddisp.pl?gene=BEND6 |
| CCDC180 | Coiled-Coil Domain Containing 180 | Protein Coding | 31 | GC09P097307 | 0.346795946 | https://www.genecards.org/cgi-bin/carddisp.pl?gene=CCDC180 |
| VWC2L | Von Willebrand Factor C Domain Containing 2 Like | Protein Coding | 31 | GC02P214411 | 0.346795946 | https://www.genecards.org/cgi-bin/carddisp.pl?gene=VWC2L |
| PCDHGB3 | Protocadherin Gamma Subfamily B, 3 | Protein Coding | 31 | GC05P143820 | 0.346795946 | https://www.genecards.org/cgi-bin/carddisp.pl?gene=PCDHGB3 |
| PRRC2B | Proline Rich Coiled-Coil 2B | Protein Coding | 31 | GC09P131373 | 0.346795946 | https://www.genecards.org/cgi-bin/carddisp.pl?gene=PRRC2B |
| SMCR8 | SMCR8-C9orf72 Complex Subunit | Protein Coding | 31 | GC17P019210 | 0.346795946 | https://www.genecards.org/cgi-bin/carddisp.pl?gene=SMCR8 |
| NOP9 | NOP9 Nucleolar Protein | Protein Coding | 31 | GC14P024299 | 0.346795946 | https://www.genecards.org/cgi-bin/carddisp.pl?gene=NOP9 |
| SCIMP | SLP Adaptor And CSK Interacting Membrane Protein | Protein Coding | 31 | GC17M005208 | 0.346795946 | https://www.genecards.org/cgi-bin/carddisp.pl?gene=SCIMP |
| MROH7 | Maestro Heat Like Repeat Family Member 7 | Protein Coding | 31 | GC01P054643 | 0.346795946 | https://www.genecards.org/cgi-bin/carddisp.pl?gene=MROH7 |
| TOMM70 | Translocase Of Outer Mitochondrial Membrane 70 | Protein Coding | 31 | GC03M100364 | 0.346795946 | https://www.genecards.org/cgi-bin/carddisp.pl?gene=TOMM70 |
| DGAT2L6 | Diacylglycerol O-Acyltransferase 2 Like 6 | Protein Coding | 30 | GC0XP070177 | 0.346795946 | https://www.genecards.org/cgi-bin/carddisp.pl?gene=DGAT2L6 |
| C1orf122 | Chromosome 1 Open Reading Frame 122 | Protein Coding | 29 | GC01P037806 | 0.346795946 | https://www.genecards.org/cgi-bin/carddisp.pl?gene=C1orf122 |
| MILR1 | Mast Cell Immunoglobulin Like Receptor 1 | Protein Coding | 29 | GC17P064447 | 0.346795946 | https://www.genecards.org/cgi-bin/carddisp.pl?gene=MILR1 |
| ANKRD20A1 | Ankyrin Repeat Domain 20 Family Member A1 | Protein Coding | 29 | GC09P067832 | 0.346795946 | https://www.genecards.org/cgi-bin/carddisp.pl?gene=ANKRD20A1 |
| ZNF98 | Zinc Finger Protein 98 | Protein Coding | 29 | GC19M022391 | 0.346795946 | https://www.genecards.org/cgi-bin/carddisp.pl?gene=ZNF98 |
| ZSWIM1 | Zinc Finger SWIM-Type Containing 1 | Protein Coding | 29 | GC20P045880 | 0.346795946 | https://www.genecards.org/cgi-bin/carddisp.pl?gene=ZSWIM1 |
| POLR1F | RNA Polymerase I Subunit F | Protein Coding | 29 | GC07M019695 | 0.346795946 | https://www.genecards.org/cgi-bin/carddisp.pl?gene=POLR1F |
| PRR7 | Proline Rich 7, Synaptic | Protein Coding | 29 | GC05P177446 | 0.346795946 | https://www.genecards.org/cgi-bin/carddisp.pl?gene=PRR7 |
| OR5K1 | Olfactory Receptor Family 5 Subfamily K Member 1 | Protein Coding | 29 | GC03P098469 | 0.346795946 | https://www.genecards.org/cgi-bin/carddisp.pl?gene=OR5K1 |
| OR6P1 | Olfactory Receptor Family 6 Subfamily P Member 1 | Protein Coding | 29 | GC01M158532 | 0.346795946 | https://www.genecards.org/cgi-bin/carddisp.pl?gene=OR6P1 |
| EIF5AL1 | Eukaryotic Translation Initiation Factor 5A Like 1 | Protein Coding | 28 | GC10P079512 | 0.346795946 | https://www.genecards.org/cgi-bin/carddisp.pl?gene=EIF5AL1 |
| C1orf141 | Chromosome 1 Open Reading Frame 141 | Protein Coding | 27 | GC01M067092 | 0.346795946 | https://www.genecards.org/cgi-bin/carddisp.pl?gene=C1orf141 |
| C16orf92 | Chromosome 16 Open Reading Frame 92 | Protein Coding | 27 | GC16P032394 | 0.346795946 | https://www.genecards.org/cgi-bin/carddisp.pl?gene=C16orf92 |
| KRTAP5-2 | Keratin Associated Protein 5-2 | Protein Coding | 27 | GC11M001597 | 0.346795946 | https://www.genecards.org/cgi-bin/carddisp.pl?gene=KRTAP5-2 |
| NEXMIF | Neurite Extension And Migration Factor | Protein Coding | 27 | GC0XM074733 | 0.346795946 | https://www.genecards.org/cgi-bin/carddisp.pl?gene=NEXMIF |
| ARHGAP11B | Rho GTPase Activating Protein 11B | Protein Coding | 27 | GC15P030624 | 0.346795946 | https://www.genecards.org/cgi-bin/carddisp.pl?gene=ARHGAP11B |
| KRTAP9-9 | Keratin Associated Protein 9-9 | Protein Coding | 26 | GC17P041255 | 0.346795946 | https://www.genecards.org/cgi-bin/carddisp.pl?gene=KRTAP9-9 |
| CFAP47 | Cilia And Flagella Associated Protein 47 | Protein Coding | 26 | GC0XP035919 | 0.346795946 | https://www.genecards.org/cgi-bin/carddisp.pl?gene=CFAP47 |
| CCDC182 | Coiled-Coil Domain Containing 182 | Protein Coding | 26 | GC17M057744 | 0.346795946 | https://www.genecards.org/cgi-bin/carddisp.pl?gene=CCDC182 |
| TLCD3B | TLC Domain Containing 3B | Protein Coding | 26 | GC16M031181 | 0.346795946 | https://www.genecards.org/cgi-bin/carddisp.pl?gene=TLCD3B |
| BBOF1 | Basal Body Orientation Factor 1 | Protein Coding | 25 | GC14P074021 | 0.346795946 | https://www.genecards.org/cgi-bin/carddisp.pl?gene=BBOF1 |
| KRTAP5-8 | Keratin Associated Protein 5-8 | Protein Coding | 25 | GC11P071535 | 0.346795946 | https://www.genecards.org/cgi-bin/carddisp.pl?gene=KRTAP5-8 |
| C2orf27A | Chromosome 2 Open Reading Frame 27A | RNA Gene | 25 | GC02P133727 | 0.346795946 | https://www.genecards.org/cgi-bin/carddisp.pl?gene=C2orf27A |
| MIR21 | MicroRNA 21 | RNA Gene | 24 | GC17P059841 | 0.346795946 | https://www.genecards.org/cgi-bin/carddisp.pl?gene=MIR21 |
| PVT1 | Pvt1 Oncogene | RNA Gene | 24 | GC08P127822 | 0.346795946 | https://www.genecards.org/cgi-bin/carddisp.pl?gene=PVT1 |
| MIR184 | MicroRNA 184 | RNA Gene | 23 | GC15P079209 | 0.346795946 | https://www.genecards.org/cgi-bin/carddisp.pl?gene=MIR184 |
| LINC00336 | Long Intergenic Non-Protein Coding RNA 336 | RNA Gene | 23 | GC06M033586 | 0.346795946 | https://www.genecards.org/cgi-bin/carddisp.pl?gene=LINC00336 |
| MIR140 | MicroRNA 140 | RNA Gene | 22 | GC16P069934 | 0.346795946 | https://www.genecards.org/cgi-bin/carddisp.pl?gene=MIR140 |
| MIR200A | MicroRNA 200a | RNA Gene | 22 | GC01P002022 | 0.346795946 | https://www.genecards.org/cgi-bin/carddisp.pl?gene=MIR200A |
| MIR145 | MicroRNA 145 | RNA Gene | 22 | GC05P149430 | 0.346795946 | https://www.genecards.org/cgi-bin/carddisp.pl?gene=MIR145 |
| KRTAP6-3 | Keratin Associated Protein 6-3 | Protein Coding | 21 | GC21P030592 | 0.346795946 | https://www.genecards.org/cgi-bin/carddisp.pl?gene=KRTAP6-3 |
| MIR17 | MicroRNA 17 | RNA Gene | 21 | GC13P091350 | 0.346795946 | https://www.genecards.org/cgi-bin/carddisp.pl?gene=MIR17 |
| MIR210 | MicroRNA 210 | RNA Gene | 21 | GC11M001082 | 0.346795946 | https://www.genecards.org/cgi-bin/carddisp.pl?gene=MIR210 |
| MIR142 | MicroRNA 142 | RNA Gene | 20 | GC17M058331 | 0.346795946 | https://www.genecards.org/cgi-bin/carddisp.pl?gene=MIR142 |
| MIR30B | MicroRNA 30b | RNA Gene | 20 | GC08M134800 | 0.346795946 | https://www.genecards.org/cgi-bin/carddisp.pl?gene=MIR30B |
| MIR373 | MicroRNA 373 | RNA Gene | 20 | GC19P056408 | 0.346795946 | https://www.genecards.org/cgi-bin/carddisp.pl?gene=MIR373 |
| MIR206 | MicroRNA 206 | RNA Gene | 20 | GC06P052144 | 0.346795946 | https://www.genecards.org/cgi-bin/carddisp.pl?gene=MIR206 |
| LINC01554 | Long Intergenic Non-Protein Coding RNA 1554 | RNA Gene | 19 | GC05P095838 | 0.346795946 | https://www.genecards.org/cgi-bin/carddisp.pl?gene=LINC01554 |
| MIR20A | MicroRNA 20a | RNA Gene | 19 | GC13P091444 | 0.346795946 | https://www.genecards.org/cgi-bin/carddisp.pl?gene=MIR20A |
| MIRLET7A3 | MicroRNA Let-7a-3 | RNA Gene | 19 | GC22P046112 | 0.346795946 | https://www.genecards.org/cgi-bin/carddisp.pl?gene=MIRLET7A3 |
| SNHG15 | Small Nucleolar RNA Host Gene 15 | RNA Gene | 19 | GC07M044983 | 0.346795946 | https://www.genecards.org/cgi-bin/carddisp.pl?gene=SNHG15 |
| HAR1B | Highly Accelerated Region 1B | RNA Gene | 18 | GC20M063095 | 0.346795946 | https://www.genecards.org/cgi-bin/carddisp.pl?gene=HAR1B |
| MIR193A | MicroRNA 193a | RNA Gene | 18 | GC17P031559 | 0.346795946 | https://www.genecards.org/cgi-bin/carddisp.pl?gene=MIR193A |
| MIR186 | MicroRNA 186 | RNA Gene | 18 | GC01M071067 | 0.346795946 | https://www.genecards.org/cgi-bin/carddisp.pl?gene=MIR186 |
| MIR18A | MicroRNA 18a | RNA Gene | 18 | GC13P091464 | 0.346795946 | https://www.genecards.org/cgi-bin/carddisp.pl?gene=MIR18A |
| MIR520A | MicroRNA 520a | RNA Gene | 18 | GC19P053690 | 0.346795946 | https://www.genecards.org/cgi-bin/carddisp.pl?gene=MIR520A |
| MIR98 | MicroRNA 98 | RNA Gene | 17 | GC0XM053595 | 0.346795946 | https://www.genecards.org/cgi-bin/carddisp.pl?gene=MIR98 |
| MIR542 | MicroRNA 542 | RNA Gene | 17 | GC0XM134638 | 0.346795946 | https://www.genecards.org/cgi-bin/carddisp.pl?gene=MIR542 |
| LINC00667 | Long Intergenic Non-Protein Coding RNA 667 | RNA Gene | 17 | GC18P005233 | 0.346795946 | https://www.genecards.org/cgi-bin/carddisp.pl?gene=LINC00667 |
| LEF1-AS1 | LEF1 Antisense RNA 1 | RNA Gene | 16 | GC04P108167 | 0.346795946 | https://www.genecards.org/cgi-bin/carddisp.pl?gene=LEF1-AS1 |
| HNRNPCL2 | Heterogeneous Nuclear Ribonucleoprotein C Like 2 | Protein Coding | 16 | GC01M013115 | 0.346795946 | https://www.genecards.org/cgi-bin/carddisp.pl?gene=HNRNPCL2 |
| MIR374A | MicroRNA 374a | RNA Gene | 16 | GC0XM074296 | 0.346795946 | https://www.genecards.org/cgi-bin/carddisp.pl?gene=MIR374A |
| MIR361 | MicroRNA 361 | RNA Gene | 16 | GC0XM085903 | 0.346795946 | https://www.genecards.org/cgi-bin/carddisp.pl?gene=MIR361 |
| MIR494 | MicroRNA 494 | RNA Gene | 16 | GC14P106333 | 0.346795946 | https://www.genecards.org/cgi-bin/carddisp.pl?gene=MIR494 |
| LINC00051 | Long Intergenic Non-Protein Coding RNA 51 | RNA Gene | 16 | GC08P142198 | 0.346795946 | https://www.genecards.org/cgi-bin/carddisp.pl?gene=LINC00051 |
| LINC00323 | Long Intergenic Non-Protein Coding RNA 323 | RNA Gene | 16 | GC21M041141 | 0.346795946 | https://www.genecards.org/cgi-bin/carddisp.pl?gene=LINC00323 |
| LINC01139 | Long Intergenic Non-Protein Coding RNA 1139 | RNA Gene | 16 | GC01M238480 | 0.346795946 | https://www.genecards.org/cgi-bin/carddisp.pl?gene=LINC01139 |
| ZEB1-AS1 | ZEB1 Antisense RNA 1 | RNA Gene | 16 | GC10M031166 | 0.346795946 | https://www.genecards.org/cgi-bin/carddisp.pl?gene=ZEB1-AS1 |
| MIR532 | MicroRNA 532 | RNA Gene | 16 | GC0XP050004 | 0.346795946 | https://www.genecards.org/cgi-bin/carddisp.pl?gene=MIR532 |
| MIR614 | MicroRNA 614 | RNA Gene | 16 | GC12P012958 | 0.346795946 | https://www.genecards.org/cgi-bin/carddisp.pl?gene=MIR614 |
| SNHG6 | Small Nucleolar RNA Host Gene 6 | RNA Gene | 16 | GC08M066921 | 0.346795946 | https://www.genecards.org/cgi-bin/carddisp.pl?gene=SNHG6 |
| LINC01020 | Long Intergenic Non-Protein Coding RNA 1020 | RNA Gene | 15 | GC05P004987 | 0.346795946 | https://www.genecards.org/cgi-bin/carddisp.pl?gene=LINC01020 |
| FAM66B | Family With Sequence Similarity 66 Member B | RNA Gene | 15 | GC08M007303 | 0.346795946 | https://www.genecards.org/cgi-bin/carddisp.pl?gene=FAM66B |
| DLEU1-AS1 | DLEU1 Antisense RNA 1 | RNA Gene | 15 | GC13M050520 | 0.346795946 | https://www.genecards.org/cgi-bin/carddisp.pl?gene=DLEU1-AS1 |
| TTTY7 | Testis-Specific Transcript, Y-Linked 7 | RNA Gene | 15 | GC0YP006449 | 0.346795946 | https://www.genecards.org/cgi-bin/carddisp.pl?gene=TTTY7 |
| RNASEH2B-AS1 | RNASEH2B Antisense RNA 1 | RNA Gene | 15 | GC13M050876 | 0.346795946 | https://www.genecards.org/cgi-bin/carddisp.pl?gene=RNASEH2B-AS1 |
| SMIM2-IT1 | SMIM2 Intronic Transcript 1 | RNA Gene | 15 | GC13M044146 | 0.346795946 | https://www.genecards.org/cgi-bin/carddisp.pl?gene=SMIM2-IT1 |
| LINC00596 | Long Intergenic Non-Protein Coding RNA 596 | RNA Gene | 15 | GC14M023839 | 0.346795946 | https://www.genecards.org/cgi-bin/carddisp.pl?gene=LINC00596 |
| MIR3157 | MicroRNA 3157 | RNA Gene | 13 | GC10M096064 | 0.346795946 | https://www.genecards.org/cgi-bin/carddisp.pl?gene=MIR3157 |
| MIR4683 | MicroRNA 4683 | RNA Gene | 13 | GC10M035641 | 0.346795946 | https://www.genecards.org/cgi-bin/carddisp.pl?gene=MIR4683 |
| CDIPTOSP | CDIP Transferase Opposite Strand, Pseudogene | RNA Gene | 13 | GC16P032382 | 0.346795946 | https://www.genecards.org/cgi-bin/carddisp.pl?gene=CDIPTOSP |
| SPRY4-AS1 | SPRY4 Antisense RNA 1 | RNA Gene | 13 | GC05P143841 | 0.346795946 | https://www.genecards.org/cgi-bin/carddisp.pl?gene=SPRY4-AS1 |
| MIR664A | MicroRNA 664a | RNA Gene | 13 | GC01M220200 | 0.346795946 | https://www.genecards.org/cgi-bin/carddisp.pl?gene=MIR664A |
| LINC00929 | Long Intergenic Non-Protein Coding RNA 929 | RNA Gene | 13 | GC15P027309 | 0.346795946 | https://www.genecards.org/cgi-bin/carddisp.pl?gene=LINC00929 |
| LINC01204 | Long Intergenic Non-Protein Coding RNA 1204 | RNA Gene | 13 | GC0XP045476 | 0.346795946 | https://www.genecards.org/cgi-bin/carddisp.pl?gene=LINC01204 |
| LINC01538 | Long Intergenic Non-Protein Coding RNA 1538 | RNA Gene | 13 | GC18M064213 | 0.346795946 | https://www.genecards.org/cgi-bin/carddisp.pl?gene=LINC01538 |
| MIR4537 | MicroRNA 4537 | RNA Gene | 12 | GC14M110324 | 0.346795946 | https://www.genecards.org/cgi-bin/carddisp.pl?gene=MIR4537 |
| GATA2-AS1 | GATA2 Antisense RNA 1 | RNA Gene | 12 | GC03P128489 | 0.346795946 | https://www.genecards.org/cgi-bin/carddisp.pl?gene=GATA2-AS1 |
| THRIL | TNF And HNRNPL Related Immunoregulatory Long Non-Coding RNA | RNA Gene | 12 | GC12M125025 | 0.346795946 | https://www.genecards.org/cgi-bin/carddisp.pl?gene=THRIL |
| LOC642484 | Uncharacterized LOC642484 | RNA Gene | 12 | GC18M056003 | 0.346795946 | https://www.genecards.org/cgi-bin/carddisp.pl?gene=LOC642484 |
| LINC01798 | Long Intergenic Non-Protein Coding RNA 1798 | RNA Gene | 12 | GC02P066576 | 0.346795946 | https://www.genecards.org/cgi-bin/carddisp.pl?gene=LINC01798 |
| MIR4325 | MicroRNA 4325 | RNA Gene | 11 | GC20M057321 | 0.346795946 | https://www.genecards.org/cgi-bin/carddisp.pl?gene=MIR4325 |
| LOC101928858 | Uncharacterized LOC101928858 | RNA Gene | 11 | GC05P067379 | 0.346795946 | https://www.genecards.org/cgi-bin/carddisp.pl?gene=LOC101928858 |
| LOC100129434 | Uncharacterized LOC100129434 | RNA Gene | 11 | GC02M056173 | 0.346795946 | https://www.genecards.org/cgi-bin/carddisp.pl?gene=LOC100129434 |
| LINC01762 | Long Intergenic Non-Protein Coding RNA 1762 | RNA Gene | 11 | GC01M116424 | 0.346795946 | https://www.genecards.org/cgi-bin/carddisp.pl?gene=LINC01762 |
| MIR5186 | MicroRNA 5186 | RNA Gene | 10 | GC03M151566 | 0.346795946 | https://www.genecards.org/cgi-bin/carddisp.pl?gene=MIR5186 |
| LINC01721 | Long Intergenic Non-Protein Coding RNA 1721 | RNA Gene | 10 | GC20P024087 | 0.346795946 | https://www.genecards.org/cgi-bin/carddisp.pl?gene=LINC01721 |
| LOC101448202 | Uncharacterized LOC101448202 | RNA Gene | 9 | GC09M134819 | 0.346795946 | https://www.genecards.org/cgi-bin/carddisp.pl?gene=LOC101448202 |
| LOC105377448 | Uncharacterized LOC105377448 | RNA Gene | 9 | GC04M138721 | 0.346795946 | https://www.genecards.org/cgi-bin/carddisp.pl?gene=LOC105377448 |
| ENSG00000249772 |  | RNA Gene | 9 | GC05M081113 | 0.346795946 | https://www.genecards.org/cgi-bin/carddisp.pl?gene=ENSG00000249772 |
| RAD51L3-RFFL | RAD51L3-RFFL Readthrough | RNA Gene | 9 | GC17M035009 | 0.346795946 | https://www.genecards.org/cgi-bin/carddisp.pl?gene=RAD51L3-RFFL |
| ENSG00000270659 |  | RNA Gene | 8 | GC02P213152 | 0.346795946 | https://www.genecards.org/cgi-bin/carddisp.pl?gene=ENSG00000270659 |
| ENSG00000203325 |  | RNA Gene | 8 | GC01M032052 | 0.346795946 | https://www.genecards.org/cgi-bin/carddisp.pl?gene=ENSG00000203325 |
| ENSG00000241269 |  | RNA Gene | 8 | GC07P005419 | 0.346795946 | https://www.genecards.org/cgi-bin/carddisp.pl?gene=ENSG00000241269 |
| IGHV4-38-2 | Immunoglobulin Heavy Variable 4-38-2 | Protein Coding | 7 | GC14U901616 | 0.346795946 | https://www.genecards.org/cgi-bin/carddisp.pl?gene=IGHV4-38-2 |
| EIF2B5-DT | EIF2B5 Divergent Transcript | RNA Gene | 7 | GC03M184074 | 0.346795946 | https://www.genecards.org/cgi-bin/carddisp.pl?gene=EIF2B5-DT |
| MIR6134 | MicroRNA 6134 | RNA Gene | 7 | GC0XM028495 | 0.346795946 | https://www.genecards.org/cgi-bin/carddisp.pl?gene=MIR6134 |
| ENSG00000256218 |  | RNA Gene | 6 | GC12M005366 | 0.346795946 | https://www.genecards.org/cgi-bin/carddisp.pl?gene=ENSG00000256218 |
| ENSG00000248176 |  | RNA Gene | 6 | GC04P029119 | 0.346795946 | https://www.genecards.org/cgi-bin/carddisp.pl?gene=ENSG00000248176 |
| ENSG00000280339 |  | Uncategorized | 4 | GC11M086908 | 0.346795946 | https://www.genecards.org/cgi-bin/carddisp.pl?gene=ENSG00000280339 |
| PRD | Primary Retinal Dysplasia | Genetic Locus | 4 | GC0XU990188 | 0.346795946 | https://www.genecards.org/cgi-bin/carddisp.pl?gene=PRD |
| CNC2 | Carney Complex Type 2, Multiple Neoplasia And Lentiginosis | Genetic Locus | 3 | GC02U990267 | 0.346795946 | https://www.genecards.org/cgi-bin/carddisp.pl?gene=CNC2 |
| OPA6 | Optic Atrophy 6 (Autosomal Recessive) | Genetic Locus | 2 | GC08U900809 | 0.346795946 | https://www.genecards.org/cgi-bin/carddisp.pl?gene=OPA6 |
| KRIT1 | KRIT1 Ankyrin Repeat Containing | Protein Coding | 43 | GC07M092198 | 0.336480111 | https://www.genecards.org/cgi-bin/carddisp.pl?gene=KRIT1 |
| CCM2 | CCM2 Scaffold Protein | Protein Coding | 44 | GC07P044999 | 0.284377605 | https://www.genecards.org/cgi-bin/carddisp.pl?gene=CCM2 |
| SLC18A2 | Solute Carrier Family 18 Member A2 | Protein Coding | 48 | GC10P117241 | 0.272322834 | https://www.genecards.org/cgi-bin/carddisp.pl?gene=SLC18A2 |
| SVEP1 | Sushi, Von Willebrand Factor Type A, EGF And Pentraxin Domain Containing 1 | Protein Coding | 36 | GC09M110365 | 0.272322834 | https://www.genecards.org/cgi-bin/carddisp.pl?gene=SVEP1 |
| C11orf80 | Chromosome 11 Open Reading Frame 80 | Protein Coding | 35 | GC11P066744 | 0.272322834 | https://www.genecards.org/cgi-bin/carddisp.pl?gene=C11orf80 |
| MHRT | Myosin Heavy Chain Associated RNA Transcript | RNA Gene | 11 | GC14P026465 | 0.272322834 | https://www.genecards.org/cgi-bin/carddisp.pl?gene=MHRT |
| KCNJ8 | Potassium Inwardly Rectifying Channel Subfamily J Member 8 | Protein Coding | 45 | GC12M021764 | 0.220277935 | https://www.genecards.org/cgi-bin/carddisp.pl?gene=KCNJ8 |
| COCH | Cochlin | Protein Coding | 43 | GC14P030874 | 0.220277935 | https://www.genecards.org/cgi-bin/carddisp.pl?gene=COCH |
| MYO7A | Myosin VIIA | Protein Coding | 43 | GC11P077128 | 0.220277935 | https://www.genecards.org/cgi-bin/carddisp.pl?gene=MYO7A |
| PDCD10 | Programmed Cell Death 10 | Protein Coding | 43 | GC03M167683 | 0.220277935 | https://www.genecards.org/cgi-bin/carddisp.pl?gene=PDCD10 |
| POU4F3 | POU Class 4 Homeobox 3 | Protein Coding | 40 | GC05P146339 | 0.220277935 | https://www.genecards.org/cgi-bin/carddisp.pl?gene=POU4F3 |
| KARS1 | Lysyl-TRNA Synthetase 1 | Protein Coding | 38 | GC16M075628 | 0.220277935 | https://www.genecards.org/cgi-bin/carddisp.pl?gene=KARS1 |
| GPR182 | G Protein-Coupled Receptor 182 | Protein Coding | 37 | GC12P056994 | 0.220277935 | https://www.genecards.org/cgi-bin/carddisp.pl?gene=GPR182 |
| TACR3 | Tachykinin Receptor 3 | Protein Coding | 51 | GC04M103586 | 0.179856181 | https://www.genecards.org/cgi-bin/carddisp.pl?gene=TACR3 |
| LPL | Lipoprotein Lipase | Protein Coding | 50 | GC08P019901 | 0.179856181 | https://www.genecards.org/cgi-bin/carddisp.pl?gene=LPL |
| GCH1 | GTP Cyclohydrolase 1 | Protein Coding | 48 | GC14M054842 | 0.179856181 | https://www.genecards.org/cgi-bin/carddisp.pl?gene=GCH1 |
| ADRA1D | Adrenoceptor Alpha 1D | Protein Coding | 47 | GC20M004220 | 0.179856181 | https://www.genecards.org/cgi-bin/carddisp.pl?gene=ADRA1D |
| FMO3 | Flavin Containing Dimethylaniline Monoxygenase 3 | Protein Coding | 47 | GC01P171090 | 0.179856181 | https://www.genecards.org/cgi-bin/carddisp.pl?gene=FMO3 |
| ALDH5A1 | Aldehyde Dehydrogenase 5 Family Member A1 | Protein Coding | 47 | GC06P024494 | 0.179856181 | https://www.genecards.org/cgi-bin/carddisp.pl?gene=ALDH5A1 |
| OGDH | Oxoglutarate Dehydrogenase | Protein Coding | 46 | GC07P044606 | 0.179856181 | https://www.genecards.org/cgi-bin/carddisp.pl?gene=OGDH |
| ADCY10 | Adenylate Cyclase 10 | Protein Coding | 45 | GC01M167809 | 0.179856181 | https://www.genecards.org/cgi-bin/carddisp.pl?gene=ADCY10 |
| MYO5A | Myosin VA | Protein Coding | 45 | GC15M067802 | 0.179856181 | https://www.genecards.org/cgi-bin/carddisp.pl?gene=MYO5A |
| APOC3 | Apolipoprotein C3 | Protein Coding | 44 | GC11P116829 | 0.179856181 | https://www.genecards.org/cgi-bin/carddisp.pl?gene=APOC3 |
| GNE | Glucosamine (UDP-N-Acetyl)-2-Epimerase/N-Acetylmannosamine Kinase | Protein Coding | 44 | GC09M036214 | 0.179856181 | https://www.genecards.org/cgi-bin/carddisp.pl?gene=GNE |
| USF1 | Upstream Transcription Factor 1 | Protein Coding | 44 | GC01M161039 | 0.179856181 | https://www.genecards.org/cgi-bin/carddisp.pl?gene=USF1 |
| PEX2 | Peroxisomal Biogenesis Factor 2 | Protein Coding | 44 | GC08M076980 | 0.179856181 | https://www.genecards.org/cgi-bin/carddisp.pl?gene=PEX2 |
| ALDH9A1 | Aldehyde Dehydrogenase 9 Family Member A1 | Protein Coding | 42 | GC01M165670 | 0.179856181 | https://www.genecards.org/cgi-bin/carddisp.pl?gene=ALDH9A1 |
| GRHL2 | Grainyhead Like Transcription Factor 2 | Protein Coding | 41 | GC08P101492 | 0.179856181 | https://www.genecards.org/cgi-bin/carddisp.pl?gene=GRHL2 |
| HLA-DRB5 | Major Histocompatibility Complex, Class II, DR Beta 5 | Protein Coding | 41 | GC06M046926 | 0.179856181 | https://www.genecards.org/cgi-bin/carddisp.pl?gene=HLA-DRB5 |
| TNFSF12 | TNF Superfamily Member 12 | Protein Coding | 41 | GC17P009104 | 0.179856181 | https://www.genecards.org/cgi-bin/carddisp.pl?gene=TNFSF12 |
| USF2 | Upstream Transcription Factor 2, C-Fos Interacting | Protein Coding | 40 | GC19P035268 | 0.179856181 | https://www.genecards.org/cgi-bin/carddisp.pl?gene=USF2 |
| DNAH8 | Dynein Axonemal Heavy Chain 8 | Protein Coding | 38 | GC06P055369 | 0.179856181 | https://www.genecards.org/cgi-bin/carddisp.pl?gene=DNAH8 |
| FAM136A | Family With Sequence Similarity 136 Member A | Protein Coding | 37 | GC02M070296 | 0.179856181 | https://www.genecards.org/cgi-bin/carddisp.pl?gene=FAM136A |
| HLA-DRB4 | Major Histocompatibility Complex, Class II, DR Beta 4 | Protein Coding | 30 | GC06Mo03851 | 0.179856181 | https://www.genecards.org/cgi-bin/carddisp.pl?gene=HLA-DRB4 |
| HLA-DRB3 | Major Histocompatibility Complex, Class II, DR Beta 3 | Protein Coding | 29 | GC06Mn03715 | 0.179856181 | https://www.genecards.org/cgi-bin/carddisp.pl?gene=HLA-DRB3 |
| EIG3 | Epilepsy, Idiopathic Generalized, Susceptibility To 3 | Genetic Locus | 1 | GC09U900639 | 0.179856181 | https://www.genecards.org/cgi-bin/carddisp.pl?gene=EIG3 |
| LOC109461478 | ATXN8 Opposite Strand (Non-Protein Coding) Repeat Instability Region | Biological Region | 1 | GC13P070140 | 0.179856181 | https://www.genecards.org/cgi-bin/carddisp.pl?gene=LOC109461478 |
| ATP1A1 | ATPase Na+/K+ Transporting Subunit Alpha 1 | Protein Coding | 51 | GC01P116372 | 0.127177522 | https://www.genecards.org/cgi-bin/carddisp.pl?gene=ATP1A1 |
| KCNH2 | Potassium Voltage-Gated Channel Subfamily H Member 2 | Protein Coding | 50 | GC07M150944 | 0.127177522 | https://www.genecards.org/cgi-bin/carddisp.pl?gene=KCNH2 |
| GLUL | Glutamate-Ammonia Ligase | Protein Coding | 50 | GC01M182378 | 0.127177522 | https://www.genecards.org/cgi-bin/carddisp.pl?gene=GLUL |
| ABCC1 | ATP Binding Cassette Subfamily C Member 1 | Protein Coding | 50 | GC16P015949 | 0.127177522 | https://www.genecards.org/cgi-bin/carddisp.pl?gene=ABCC1 |
| KCNJ2 | Potassium Inwardly Rectifying Channel Subfamily J Member 2 | Protein Coding | 49 | GC17P070168 | 0.127177522 | https://www.genecards.org/cgi-bin/carddisp.pl?gene=KCNJ2 |
| GAPDH | Glyceraldehyde-3-Phosphate Dehydrogenase | Protein Coding | 48 | GC12P011841 | 0.127177522 | https://www.genecards.org/cgi-bin/carddisp.pl?gene=GAPDH |
| CA12 | Carbonic Anhydrase 12 | Protein Coding | 48 | GC15M063321 | 0.127177522 | https://www.genecards.org/cgi-bin/carddisp.pl?gene=CA12 |
| CAV1 | Caveolin 1 | Protein Coding | 48 | GC07P116524 | 0.127177522 | https://www.genecards.org/cgi-bin/carddisp.pl?gene=CAV1 |
| GCGR | Glucagon Receptor | Protein Coding | 48 | GC17P081804 | 0.127177522 | https://www.genecards.org/cgi-bin/carddisp.pl?gene=GCGR |
| DMD | Dystrophin | Protein Coding | 47 | GC0XM031097 | 0.127177522 | https://www.genecards.org/cgi-bin/carddisp.pl?gene=DMD |
| CA4 | Carbonic Anhydrase 4 | Protein Coding | 47 | GC17P060149 | 0.127177522 | https://www.genecards.org/cgi-bin/carddisp.pl?gene=CA4 |
| CUL3 | Cullin 3 | Protein Coding | 47 | GC02M224470 | 0.127177522 | https://www.genecards.org/cgi-bin/carddisp.pl?gene=CUL3 |
| TBX5 | T-Box Transcription Factor 5 | Protein Coding | 47 | GC12M114353 | 0.127177522 | https://www.genecards.org/cgi-bin/carddisp.pl?gene=TBX5 |
| CYP2E1 | Cytochrome P450 Family 2 Subfamily E Member 1 | Protein Coding | 46 | GC10P133520 | 0.127177522 | https://www.genecards.org/cgi-bin/carddisp.pl?gene=CYP2E1 |
| PDE2A | Phosphodiesterase 2A | Protein Coding | 46 | GC11M072576 | 0.127177522 | https://www.genecards.org/cgi-bin/carddisp.pl?gene=PDE2A |
| PTHLH | Parathyroid Hormone Like Hormone | Protein Coding | 46 | GC12M027959 | 0.127177522 | https://www.genecards.org/cgi-bin/carddisp.pl?gene=PTHLH |
| KCNA5 | Potassium Voltage-Gated Channel Subfamily A Member 5 | Protein Coding | 46 | GC12P005043 | 0.127177522 | https://www.genecards.org/cgi-bin/carddisp.pl?gene=KCNA5 |
| CA9 | Carbonic Anhydrase 9 | Protein Coding | 45 | GC09P035673 | 0.127177522 | https://www.genecards.org/cgi-bin/carddisp.pl?gene=CA9 |
| HSD17B2 | Hydroxysteroid 17-Beta Dehydrogenase 2 | Protein Coding | 45 | GC16P082068 | 0.127177522 | https://www.genecards.org/cgi-bin/carddisp.pl?gene=HSD17B2 |
| HMBS | Hydroxymethylbilane Synthase | Protein Coding | 45 | GC11P119084 | 0.127177522 | https://www.genecards.org/cgi-bin/carddisp.pl?gene=HMBS |
| NKX2-5 | NK2 Homeobox 5 | Protein Coding | 45 | GC05M173232 | 0.127177522 | https://www.genecards.org/cgi-bin/carddisp.pl?gene=NKX2-5 |
| CTSA | Cathepsin A | Protein Coding | 45 | GC20P045890 | 0.127177522 | https://www.genecards.org/cgi-bin/carddisp.pl?gene=CTSA |
| WIF1 | WNT Inhibitory Factor 1 | Protein Coding | 45 | GC12M065050 | 0.127177522 | https://www.genecards.org/cgi-bin/carddisp.pl?gene=WIF1 |
| CA5A | Carbonic Anhydrase 5A | Protein Coding | 44 | GC16M087882 | 0.127177522 | https://www.genecards.org/cgi-bin/carddisp.pl?gene=CA5A |
| LTF | Lactotransferrin | Protein Coding | 44 | GC03M046435 | 0.127177522 | https://www.genecards.org/cgi-bin/carddisp.pl?gene=LTF |
| HSD17B1 | Hydroxysteroid 17-Beta Dehydrogenase 1 | Protein Coding | 44 | GC17P042548 | 0.127177522 | https://www.genecards.org/cgi-bin/carddisp.pl?gene=HSD17B1 |
| DYSF | Dysferlin | Protein Coding | 44 | GC02P071453 | 0.127177522 | https://www.genecards.org/cgi-bin/carddisp.pl?gene=DYSF |
| TACR2 | Tachykinin Receptor 2 | Protein Coding | 44 | GC10M069403 | 0.127177522 | https://www.genecards.org/cgi-bin/carddisp.pl?gene=TACR2 |
| TIMM8A | Translocase Of Inner Mitochondrial Membrane 8A | Protein Coding | 44 | GC0XM101345 | 0.127177522 | https://www.genecards.org/cgi-bin/carddisp.pl?gene=TIMM8A |
| PDE3B | Phosphodiesterase 3B | Protein Coding | 44 | GC11P014643 | 0.127177522 | https://www.genecards.org/cgi-bin/carddisp.pl?gene=PDE3B |
| CA6 | Carbonic Anhydrase 6 | Protein Coding | 43 | GC01P008945 | 0.127177522 | https://www.genecards.org/cgi-bin/carddisp.pl?gene=CA6 |
| MAPRE2 | Microtubule Associated Protein RP/EB Family Member 2 | Protein Coding | 43 | GC18P034976 | 0.127177522 | https://www.genecards.org/cgi-bin/carddisp.pl?gene=MAPRE2 |
| CALB1 | Calbindin 1 | Protein Coding | 43 | GC08M090058 | 0.127177522 | https://www.genecards.org/cgi-bin/carddisp.pl?gene=CALB1 |
| ARX | Aristaless Related Homeobox | Protein Coding | 43 | GC0XM025003 | 0.127177522 | https://www.genecards.org/cgi-bin/carddisp.pl?gene=ARX |
| DAGLA | Diacylglycerol Lipase Alpha | Protein Coding | 43 | GC11P061680 | 0.127177522 | https://www.genecards.org/cgi-bin/carddisp.pl?gene=DAGLA |
| S100A9 | S100 Calcium Binding Protein A9 | Protein Coding | 43 | GC01P153357 | 0.127177522 | https://www.genecards.org/cgi-bin/carddisp.pl?gene=S100A9 |
| CA3 | Carbonic Anhydrase 3 | Protein Coding | 42 | GC08P085373 | 0.127177522 | https://www.genecards.org/cgi-bin/carddisp.pl?gene=CA3 |
| UGT1A4 | UDP Glucuronosyltransferase Family 1 Member A4 | Protein Coding | 42 | GC02P233718 | 0.127177522 | https://www.genecards.org/cgi-bin/carddisp.pl?gene=UGT1A4 |
| SLC17A7 | Solute Carrier Family 17 Member 7 | Protein Coding | 42 | GC19M049429 | 0.127177522 | https://www.genecards.org/cgi-bin/carddisp.pl?gene=SLC17A7 |
| ANO10 | Anoctamin 10 | Protein Coding | 41 | GC03M043355 | 0.127177522 | https://www.genecards.org/cgi-bin/carddisp.pl?gene=ANO10 |
| GBA2 | Glucosylceramidase Beta 2 | Protein Coding | 41 | GC09M035736 | 0.127177522 | https://www.genecards.org/cgi-bin/carddisp.pl?gene=GBA2 |
| ANK2 | Ankyrin 2 | Protein Coding | 41 | GC04P112706 | 0.127177522 | https://www.genecards.org/cgi-bin/carddisp.pl?gene=ANK2 |
| UGT1A10 | UDP Glucuronosyltransferase Family 1 Member A10 | Protein Coding | 41 | GC02P233636 | 0.127177522 | https://www.genecards.org/cgi-bin/carddisp.pl?gene=UGT1A10 |
| SLC17A6 | Solute Carrier Family 17 Member 6 | Protein Coding | 41 | GC11P022359 | 0.127177522 | https://www.genecards.org/cgi-bin/carddisp.pl?gene=SLC17A6 |
| MLNR | Motilin Receptor | Protein Coding | 41 | GC13P049220 | 0.127177522 | https://www.genecards.org/cgi-bin/carddisp.pl?gene=MLNR |
| CA10 | Carbonic Anhydrase 10 | Protein Coding | 40 | GC17M051630 | 0.127177522 | https://www.genecards.org/cgi-bin/carddisp.pl?gene=CA10 |
| ZMYM2 | Zinc Finger MYM-Type Containing 2 | Protein Coding | 40 | GC13P019958 | 0.127177522 | https://www.genecards.org/cgi-bin/carddisp.pl?gene=ZMYM2 |
| OPA3 | Outer Mitochondrial Membrane Lipid Metabolism Regulator OPA3 | Protein Coding | 40 | GC19M045527 | 0.127177522 | https://www.genecards.org/cgi-bin/carddisp.pl?gene=OPA3 |
| GPER1 | G Protein-Coupled Estrogen Receptor 1 | Protein Coding | 38 | GC07P001368 | 0.127177522 | https://www.genecards.org/cgi-bin/carddisp.pl?gene=GPER1 |
| SYT14 | Synaptotagmin 14 | Protein Coding | 38 | GC01P209900 | 0.127177522 | https://www.genecards.org/cgi-bin/carddisp.pl?gene=SYT14 |
| TAAR1 | Trace Amine Associated Receptor 1 | Protein Coding | 38 | GC06M132644 | 0.127177522 | https://www.genecards.org/cgi-bin/carddisp.pl?gene=TAAR1 |
| MAGEB2 | MAGE Family Member B2 | Protein Coding | 36 | GC0XP030215 | 0.127177522 | https://www.genecards.org/cgi-bin/carddisp.pl?gene=MAGEB2 |
| LACTB | Lactamase Beta | Protein Coding | 36 | GC15P077630 | 0.127177522 | https://www.genecards.org/cgi-bin/carddisp.pl?gene=LACTB |
| UGT1A8 | UDP Glucuronosyltransferase Family 1 Member A8 | Protein Coding | 36 | GC02P233618 | 0.127177522 | https://www.genecards.org/cgi-bin/carddisp.pl?gene=UGT1A8 |
| RLN1 | Relaxin 1 | Protein Coding | 36 | GC09M005334 | 0.127177522 | https://www.genecards.org/cgi-bin/carddisp.pl?gene=RLN1 |
| RHOBTB3 | Rho Related BTB Domain Containing 3 | Protein Coding | 36 | GC05P095713 | 0.127177522 | https://www.genecards.org/cgi-bin/carddisp.pl?gene=RHOBTB3 |
| FCRL6 | Fc Receptor Like 6 | Protein Coding | 34 | GC01P159800 | 0.127177522 | https://www.genecards.org/cgi-bin/carddisp.pl?gene=FCRL6 |
| ZACN | Zinc Activated Ion Channel | Protein Coding | 34 | GC17P076071 | 0.127177522 | https://www.genecards.org/cgi-bin/carddisp.pl?gene=ZACN |
| STRC | Stereocilin | Protein Coding | 34 | GC15M043599 | 0.127177522 | https://www.genecards.org/cgi-bin/carddisp.pl?gene=STRC |
| TAAR8 | Trace Amine Associated Receptor 8 | Protein Coding | 32 | GC06P132552 | 0.127177522 | https://www.genecards.org/cgi-bin/carddisp.pl?gene=TAAR8 |
| FDX2 | Ferredoxin 2 | Protein Coding | 27 | GC19M010326 | 0.127177522 | https://www.genecards.org/cgi-bin/carddisp.pl?gene=FDX2 |
| BPPV | Vestibulopathy, Familial | Genetic Locus | 1 | GC06U900806 | 0.127177522 | https://www.genecards.org/cgi-bin/carddisp.pl?gene=BPPV |
| STQTL2 | Stature Quantitative Trait Locus 2 | Genetic Locus | 1 | GC07U901815 | 0.127177522 | https://www.genecards.org/cgi-bin/carddisp.pl?gene=STQTL2 |
| LILRB2 | Leukocyte Immunoglobulin Like Receptor B2 | Protein Coding | 41 | GC19M054562 | 0.099895194 | https://www.genecards.org/cgi-bin/carddisp.pl?gene=LILRB2 |
|  |  |  |  |  |  |  |
| Cytogenetic location | Genomic coordinates (From NCBI/GRCh38) | Gene/Locus | Gene/Locus name | Gene/Locus MIM number | Approved Symbol | Entrez Gene ID |
| 1q23.2 | 1:160115758-160143590 | ATP1A2, FHM2, MHP2 | ATPase, Na+K+ transporting, alpha-2 polypeptide | 182340 | ATP1A2 | 477 |
| 1q23.2 | 1:160115758-160143590 | ATP1A2, FHM2, MHP2 | ATPase, Na+K+ transporting, alpha-2 polypeptide | 182340 | ATP1A2 | 477 |
| 1q23.2 | 1:160115758-160143590 | ATP1A2, FHM2, MHP2 | ATPase, Na+K+ transporting, alpha-2 polypeptide | 182340 | ATP1A2 | 477 |
| 1q31 | 1:185800000-198700000 | MGR6, FHM3 | Migraine, several forms | 607516 |  | 317773 |
| 1q31 | 1:185800000-198700000 | MGR6, FHM3 | Migraine, several forms | 607516 |  | 317773 |
| 2q24.3 | 2:165984640-166149160 | SCN1A, GEFSP2, SMEI, FEB3A, DEE6A, DRVT, FHM3, DEE6B | Sodium voltage-gated channel, alpha subunit 1 | 182389 | SCN1A | 6323 |
| 2q24.3 | 2:165984640-166149160 | SCN1A, GEFSP2, SMEI, FEB3A, DEE6A, DRVT, FHM3, DEE6B | Sodium voltage-gated channel, alpha subunit 1 | 182389 | SCN1A | 6323 |
| 2q24.3 | 2:165984640-166149160 | SCN1A, GEFSP2, SMEI, FEB3A, DEE6A, DRVT, FHM3, DEE6B | Sodium voltage-gated channel, alpha subunit 1 | 182389 | SCN1A | 6323 |
| 2q24.3 | 2:165984640-166149160 | SCN1A, GEFSP2, SMEI, FEB3A, DEE6A, DRVT, FHM3, DEE6B | Sodium voltage-gated channel, alpha subunit 1 | 182389 | SCN1A | 6323 |
| 2q24.3 | 2:165984640-166149160 | SCN1A, GEFSP2, SMEI, FEB3A, DEE6A, DRVT, FHM3, DEE6B | Sodium voltage-gated channel, alpha subunit 1 | 182389 | SCN1A | 6323 |
| 4q24 | 4:100100000-106700000 | MGR1, MA | Migraine with or without aura, susceptibility to, 1 | 157300 |  | 192115 |
| 4q31.22-q31.23 | 4:147481096-147544953 | EDNRA, MFDA | Endothelin receptor type A | 131243 | EDNRA | 1909 |
| 4q31.22-q31.23 | 4:147481096-147544953 | EDNRA, MFDA | Endothelin receptor type A | 131243 | EDNRA | 1909 |
| 5q21 | 5:98900000-110200000 | MGR8 | Migraine, susceptibility to, 8 | 609570 |  | 100188790 |
| 6p21.33 | 6:31575564-31578335 | TNF, TNFA | Tumor necrosis factor (cachectin) | 191160 | TNF | 7124 |
| 6p21.33 | 6:31575564-31578335 | TNF, TNFA | Tumor necrosis factor (cachectin) | 191160 | TNF | 7124 |
| 6p21.33 | 6:31575564-31578335 | TNF, TNFA | Tumor necrosis factor (cachectin) | 191160 | TNF | 7124 |
| 6p21.33 | 6:31575564-31578335 | TNF, TNFA | Tumor necrosis factor (cachectin) | 191160 | TNF | 7124 |
| 6p21.33 | 6:31575564-31578335 | TNF, TNFA | Tumor necrosis factor (cachectin) | 191160 | TNF | 7124 |
| 6p21.1-p12.2 | 6:40500000-53000000 | MGR3 | Migraine with or without aura, susceptibility to, 3 | 607498 |  | 337892 |
| 6q25.1-q25.2 | 6:151654147-152129618 | ESR1, ESR, ESTRR | Estrogen receptor 1 | 133430 | ESR1 | 2099 |
| 6q25.1-q25.2 | 6:151654147-152129618 | ESR1, ESR, ESTRR | Estrogen receptor 1 | 133430 | ESR1 | 2099 |
| 6q25.1-q25.2 | 6:151654147-152129618 | ESR1, ESR, ESTRR | Estrogen receptor 1 | 133430 | ESR1 | 2099 |
| 6q25.1-q25.2 | 6:151654147-152129618 | ESR1, ESR, ESTRR | Estrogen receptor 1 | 133430 | ESR1 | 2099 |
| 10q22-q23 | 10:68800000-95300000 | MGR12 | Migraine, with or without aura, susceptibility to, 12 | 611706 |  | 100188851 |
| 10q25.3 | 10:117197488-117210298 | KCNK18, TRESK, TRIK, MGR13 | Potassium channel, subfamily K, member 18 | 613655 | KCNK18 | 338567 |
| 11q24 | 11:121300000-130900000 | MGR9 | Migraine with aura, susceptibility to, 9 | 609670 |  | 100415943 |
| 14q21.2-q22.3 | 14:43000000-57600000 | MGR4, MGOA | Migraine without aura, susceptibility to, 4 | 607501 |  | 338342 |
| 15q11.2-q12 | 15:20500000-27800000 | MGR7 | Migraine with aura, susceptibility to, 7 | 609179 |  | 553992 |
| 17p13.1 | 17:6500000-10800000 | MGR10 | Migraine with or without aura, susceptibility to, 10 | 610208 |  | 117204002 |
| 18q12.1 | 18:27500000-35100000 | MGR11 | Migraine with or without aura, susceptibility to, 11 | 610209 |  | 117204003 |
| 19p13 |  | MGR5 | Migraine with or without aura, susceptibility to, 5 | 607508 |  | 387576 |
| 19p13.13 | 19:13206441-13506478 | CACNA1A, CACNL1A4, SCA6, DEE42 | Calcium channel, voltage-dependent, P/Q type, alpha 1A subunit | 601011 | CACNA1A | 773 |
| 19p13.13 | 19:13206441-13506478 | CACNA1A, CACNL1A4, SCA6, DEE42 | Calcium channel, voltage-dependent, P/Q type, alpha 1A subunit | 601011 | CACNA1A | 773 |
| 19p13.13 | 19:13206441-13506478 | CACNA1A, CACNL1A4, SCA6, DEE42 | Calcium channel, voltage-dependent, P/Q type, alpha 1A subunit | 601011 | CACNA1A | 773 |
| 19p13.13 | 19:13206441-13506478 | CACNA1A, CACNL1A4, SCA6, DEE42 | Calcium channel, voltage-dependent, P/Q type, alpha 1A subunit | 601011 | CACNA1A | 773 |
| 19p13.13 | 19:13206441-13506478 | CACNA1A, CACNL1A4, SCA6, DEE42 | Calcium channel, voltage-dependent, P/Q type, alpha 1A subunit | 601011 | CACNA1A | 773 |
| Xq | X:61000000-156040895 | MGR2 | Migraine, familial typical, susceptibility to, 2 | 300125 |  | 8249 |
|  |  |  |  |  |  |  |
| Targets come from the HPO database |  |  |  |  |  |  |
| ACSF3 |  |  |  |  |  |  |
| ACVRL1 |  |  |  |  |  |  |
| ADA2 |  |  |  |  |  |  |
| AIP |  |  |  |  |  |  |
| AMACR |  |  |  |  |  |  |
| APP |  |  |  |  |  |  |
| ATP1A2 |  |  |  |  |  |  |
| BMPR1A |  |  |  |  |  |  |
| C4A |  |  |  |  |  |  |
| CACNA1A |  |  |  |  |  |  |
| COL3A1 |  |  |  |  |  |  |
| COL4A1 |  |  |  |  |  |  |
| COX1 |  |  |  |  |  |  |
| COX2 |  |  |  |  |  |  |
| COX3 |  |  |  |  |  |  |
| CSNK1D |  |  |  |  |  |  |
| CYTB |  |  |  |  |  |  |
| DEPDC5 |  |  |  |  |  |  |
| DNM1L |  |  |  |  |  |  |
| EDNRA |  |  |  |  |  |  |
| ENG |  |  |  |  |  |  |
| EPCAM |  |  |  |  |  |  |
| ESR1 |  |  |  |  |  |  |
| FAN1 |  |  |  |  |  |  |
| FAS |  |  |  |  |  |  |
| FGFR2 |  |  |  |  |  |  |
| FGFR3 |  |  |  |  |  |  |
| GABRG2 |  |  |  |  |  |  |
| GATA2 |  |  |  |  |  |  |
| GDF2 |  |  |  |  |  |  |
| GP1BA |  |  |  |  |  |  |
| GP1BB |  |  |  |  |  |  |
| GP9 |  |  |  |  |  |  |
| GPR101 |  |  |  |  |  |  |
| GRIN2A |  |  |  |  |  |  |
| HLA-B |  |  |  |  |  |  |
| HTR1A |  |  |  |  |  |  |
| IL10 |  |  |  |  |  |  |
| IL12B |  |  |  |  |  |  |
| IL23R |  |  |  |  |  |  |
| KCNA1 |  |  |  |  |  |  |
| KCNK18 |  |  |  |  |  |  |
| KRAS |  |  |  |  |  |  |
| LGI1 |  |  |  |  |  |  |
| MEFV |  |  |  |  |  |  |
| MLH1 |  |  |  |  |  |  |
| MLH3 |  |  |  |  |  |  |
| MSH2 |  |  |  |  |  |  |
| MSH6 |  |  |  |  |  |  |
| MVK |  |  |  |  |  |  |
| MYD88 |  |  |  |  |  |  |
| MYORG |  |  |  |  |  |  |
| ND1 |  |  |  |  |  |  |
| ND4 |  |  |  |  |  |  |
| ND5 |  |  |  |  |  |  |
| ND6 |  |  |  |  |  |  |
| NDP |  |  |  |  |  |  |
| NF1 |  |  |  |  |  |  |
| NFIX |  |  |  |  |  |  |
| NLRP3 |  |  |  |  |  |  |
| NOP56 |  |  |  |  |  |  |
| NOTCH3 |  |  |  |  |  |  |
| OPA1 |  |  |  |  |  |  |
| PDGFB |  |  |  |  |  |  |
| PDGFRB |  |  |  |  |  |  |
| PEX11B |  |  |  |  |  |  |
| PGK1 |  |  |  |  |  |  |
| PIK3CA |  |  |  |  |  |  |
| PMS1 |  |  |  |  |  |  |
| PMS2 |  |  |  |  |  |  |
| POLG |  |  |  |  |  |  |
| POLG2 |  |  |  |  |  |  |
| PRRT2 |  |  |  |  |  |  |
| RASA1 |  |  |  |  |  |  |
| RELA |  |  |  |  |  |  |
| RELN |  |  |  |  |  |  |
| RNASEH1 |  |  |  |  |  |  |
| RRM2B |  |  |  |  |  |  |
| SCN1A |  |  |  |  |  |  |
| SEMA4A |  |  |  |  |  |  |
| SLC1A3 |  |  |  |  |  |  |
| SLC25A4 |  |  |  |  |  |  |
| SLC2A1 |  |  |  |  |  |  |
| SLC6A19 |  |  |  |  |  |  |
| SMAD3 |  |  |  |  |  |  |
| SMAD4 |  |  |  |  |  |  |
| SMARCB1 |  |  |  |  |  |  |
| SPOP |  |  |  |  |  |  |
| SRPX2 |  |  |  |  |  |  |
| STARD7 |  |  |  |  |  |  |
| STAT4 |  |  |  |  |  |  |
| STIM1 |  |  |  |  |  |  |
| TGFBR2 |  |  |  |  |  |  |
| TNF |  |  |  |  |  |  |
| TNFRSF1A |  |  |  |  |  |  |
| TREX1 |  |  |  |  |  |  |
| TRNC |  |  |  |  |  |  |
| TRNF |  |  |  |  |  |  |
| TRNK |  |  |  |  |  |  |
| TRNL1 |  |  |  |  |  |  |
| TRNQ |  |  |  |  |  |  |
| TRNS1 |  |  |  |  |  |  |
| TRNS2 |  |  |  |  |  |  |
| TRNV |  |  |  |  |  |  |
| TRNW |  |  |  |  |  |  |
| TWIST1 |  |  |  |  |  |  |
| TWNK |  |  |  |  |  |  |
