# Supplementary material for: Exploring the Potential Mechanism of Chuanxiong Rhizoma Treatment for Migraine Based on Systems Pharmacology
Source: Evid Based Complement Alternat Med. 2021 Dec 28;2021:2809004. doi: 10.1155/2021/2809004 (PMC8727101; doi:10.1155/2021/2809004)
Supplement: Supplementary Materials — Supplementary Table S1: active compounds of CR). A total of 248 candidate compounds were included. Supplementary Table S2: relevant targets of CR active compounds. Supplementary Table S3: targets for Migraine. Supplementary Table S4: overlapping targets between disease and drug. Supplementary Table S5: details of the active compounds of CR and overlapping targets. Supplementary Table S6: the details of the PPI network. Supplementary Table S7: details of the GO enrichment analysis. Supplementary Table S8: details of the KEGG pathway enrichment analysis. [file 2809004.f1.zip › 2809004.f1/Supplementary Table S4 The Overlapping targets between disease and drug (1).doc]

The Overlapping targets between disease and drug

PTGS2

CHRM2

CHRNA2

PTGS1

SLC6A2

TRPV1

HSP90AA1

SCN5A

ADA

ADRA1B

SLC6A3

ADRB2

SLC6A4

MAOB

NOS2

F2

KCNH2

ESR1

AR

PPARG

F7

ESR2

DPP4

GSK3B

GRIA2

NR3C1

PGR

F10

ADRA1D

PRSS3

PLAU

SOD1

ERBB2

LPL

SERPINE1

BDNF

HMGCR

MPO

PPARA

PPARD

CRP

PON1

INS

PLG

CCK

CETP

PYY

BCL2

IL10

SLC22A5

SP1

RELA

IL6

SHBG

AKR1C1

VDR

GABRA1

GABRA3

GABRA4

GABRA5

GABRB3

GABRD

GABRE

GABRG2

GABRP

GABRQ

GRIN1

GRIN2A

GRIN2B

GRIN2D

HSD17B1

ACHE

BCHE

AKT1

ALOX5

APP

ITGB3

SLC2A1

CASP3

HSD11B1

NFKB1

NFKB2

TNF

P2RY12

PTGIR

PTGIS

RBP3

RLBP1
