# Supplementary material for: Exploring the Potential Mechanism of Chuanxiong Rhizoma Treatment for Migraine Based on Systems Pharmacology
Source: Evid Based Complement Alternat Med. 2021 Dec 28;2021:2809004. doi: 10.1155/2021/2809004 (PMC8727101; doi:10.1155/2021/2809004)
Supplement: Supplementary Materials — Supplementary Table S1: active compounds of CR). A total of 248 candidate compounds were included. Supplementary Table S2: relevant targets of CR active compounds. Supplementary Table S3: targets for Migraine. Supplementary Table S4: overlapping targets between disease and drug. Supplementary Table S5: details of the active compounds of CR and overlapping targets. Supplementary Table S6: the details of the PPI network. Supplementary Table S7: details of the GO enrichment analysis. Supplementary Table S8: details of the KEGG pathway enrichment analysis. [file 2809004.f1.zip › 2809004.f1/Supplementary Table S5 Details of the active compounds of CR and overlapping targets (1).doc]

| No. | Name | Target |
| --- | --- | --- |
| CR01 | α-Cubebene | CHRM2 |
|  |  | PTGS2 |
|  |  | CHRNA2 |
| CR02 | Linoleic acid | TRPV1 |
|  |  | CHRM2 |
|  |  | PTGS2 |
|  |  | PTGS1 |
|  |  | SLC6A2 |
| CR03 | (-)-Globulol | CHRM2 |
|  |  | PTGS2 |
| CR04 | Mandenol | PTGS2 |
|  |  | CR04 |
| CR05 | Methyl linoleate | PTGS2 |
|  |  | PTGS1 |
| CR06 | 3-Methylchrysazin | SCN5A |
|  |  | HSP90AA1 |
|  |  | PTGS2 |
|  |  | PTGS1 |
| CR07 | Adenocard | ADA |
|  |  | PTGS2 |
|  |  | PTGS1 |
| CR08 | (+)-Aromadendrene | CHRM2 |
| CR09 | methanoazulene | ADRA1B |
|  |  | CHRM2 |
|  |  | PTGS2 |
|  |  | CHRNA2 |
| CR10 | α-Selinine | ADRA1B |
|  |  | CHRM2 |
|  |  | PTGS2 |
| CR11 | β-Cubebene | CHRM2 |
|  |  | PTGS2 |
| CR12 | Chuanxiongol | SCN5A |
|  |  | SLC6A3 |
|  |  | ADRA1B |
|  |  | HSP90AA1 |
|  |  | PTGS2 |
|  |  | SLC6A4 |
|  |  | PTGS1 |
|  |  | ADRB2 |
|  |  | MAOB |
|  |  | SLC6A2 |
| CR13 | Myricanone | SCN5A |
|  |  | NOS2 |
|  |  | HSP90AA1 |
|  |  | F7 |
|  |  | AR |
|  |  | PTGS2 |
|  |  | GSK3B |
|  |  | ESR1 |
|  |  | F2 |
|  |  | KCNH2 |
|  |  | PTGS1 |
|  |  | ADRB2 |
|  |  | PPARG |
|  |  | ESR2 |
|  |  | DPP4 |
| CR14 | Perlolidin | PTGS2 |
|  |  | F2 |
| CR15 | Senkyunolide D | PTGS2 |
|  |  | GRIA2 |
| CR16 | 1-Acetyl-beta-carboline | SCN5A |
|  |  | HSP90AA1 |
|  |  | PTGS2 |
|  |  | PTGS1 |
|  |  | MAOB |
| CR17 | Espatulenol | CHRM2 |
| CR18 | Wallichilide | PTGS2 |
|  |  | NR3C1 |
| CR19 | Pedatisectine C | HSP90AA1 |
|  |  | PTGS2 |
|  |  | PTGS1 |
| CR20 | Aromadendrene oxide | CHRM2 |
| CR21 | (-)-Cedrene | ADRA1B |
|  |  | CHRM2 |
|  |  | PTGS2 |
|  |  | CHRNA2 |
| CR22 | (Z)-9-Octadecenoic acid methyl ester | PTGS2 |
|  |  | PTGS1 |
| CR23 | Alexandrin | SCN5A |
|  |  | ADRA1D |
|  |  | ADRA1B |
|  |  | HSP90AA1 |
|  |  | PTGS2 |
|  |  | PGR |
|  |  | KCNH2 |
|  |  | PTGS1 |
|  |  | ADRB2 |
| CR24 | Angelicin | PGR |
| CR25 | Folacid | GSK3B |
|  |  | F2 |
| CR26 | Oleic acid | SERPINE1 |
|  |  | PRSS3 |
|  |  | BDNF |
|  |  | SOD1 |
|  |  | PLAU |
|  |  | PTGS2 |
|  |  | CETP |
|  |  | PYY |
|  |  | HMGCR |
|  |  | CRP |
|  |  | PPARA |
|  |  | LPL |
|  |  | PTGS1 |
|  |  | PPARD |
|  |  | PON1 |
|  |  | INS |
|  |  | PLG |
|  |  | MPO |
|  |  | CCK |
|  |  | PPARG |
|  |  | ERBB2 |
| CR27 | Cetostearic acid | BCL2 |
|  |  | SLC22A5 |
|  |  | IL10 |
|  |  | PTGS2 |
|  |  | PTGS1 |
| CR28 | Octadecanoic acid | PTGS2 |
|  |  | PTGS1 |
|  |  | SP1 |
| CR29 | Methyl hexadecanoate | IL10 |
|  |  | PTGS2 |
|  |  | RELA |
|  |  | PTGS1 |
|  |  | IL6 |
| CR30 | Isoledene | CHRM2 |
| CR31 | Ferulic acid | SHBG |
| CR32 | Clionasterol | HSD17B1 |
|  |  | AR |
|  |  | PGR |
|  |  | GABRA3 |
|  |  | GABRA5 |
|  |  | GRIN2A |
|  |  | ESR1 |
|  |  | PPARA |
|  |  | GABRB3 |
|  |  | ESR2 |
|  |  | GRIN2B |
|  |  | GABRA4 |
|  |  | GABRQ |
|  |  | GABRA1 |
|  |  | AKR1C1 |
|  |  | GABRD |
|  |  | GABRP |
|  |  | GRIN2D |
|  |  | GRIN1 |
|  |  | GABRE |
|  |  | GABRG2 |
|  |  | VDR |
|  |  | NR3C1 |
| CR33 | Choline | BCHE |
|  |  | ACHE |
| CR34 | Chrysophanic Acid | ALOX5 |
|  |  | AKT1 |
|  |  | APP |
|  |  | PTGS2 |
|  |  | ESR1 |
|  |  | PPARA |
|  |  | SLC2A1 |
|  |  | ITGB3 |
|  |  | PTGS1 |
|  |  | PPARG |
| CR35 | Biocolina | BCHE |
|  |  | ACHE |
| CR36 | Xiongterpene | HSD11B1 |
|  |  | NFKB2 |
|  |  | TNF |
|  |  | NFKB1 |
|  |  | LPL |
|  |  | CASP3 |
| CR37 | Senkyunolide I | PTGIR |
|  |  | PTGIS |
|  |  | P2RY12 |
| CR38 | Retinol | RLBP1 |
|  |  | RBP3 |
