# Supplementary material for: Exploring the Potential Mechanism of Chuanxiong Rhizoma Treatment for Migraine Based on Systems Pharmacology
Source: Evid Based Complement Alternat Med. 2021 Dec 28;2021:2809004. doi: 10.1155/2021/2809004 (PMC8727101; doi:10.1155/2021/2809004)
Supplement: Supplementary Materials — Supplementary Table S1: active compounds of CR). A total of 248 candidate compounds were included. Supplementary Table S2: relevant targets of CR active compounds. Supplementary Table S3: targets for Migraine. Supplementary Table S4: overlapping targets between disease and drug. Supplementary Table S5: details of the active compounds of CR and overlapping targets. Supplementary Table S6: the details of the PPI network. Supplementary Table S7: details of the GO enrichment analysis. Supplementary Table S8: details of the KEGG pathway enrichment analysis. [file 2809004.f1.zip › 2809004.f1/Supplementary Table S6 The details of the PPI network (1).doc]

| #node1 | node2 | node1_string_id | node2_string_id | neighborhood_on_chromosome | gene_fusion | phylogenetic_cooccurrence | homology | coexpression | experimentally_determined_interaction | database_annotated | automated_textmining | combined_score |
| --- | --- | --- | --- | --- | --- | --- | --- | --- | --- | --- | --- | --- |
| ACHE | PON1 | 9606.ENSP00000303211 | 9606.ENSP00000222381 | 0 | 0 | 0 | 0 | 0.062 | 0 | 0 | 0.674 | 0.681 |
| ACHE | MPO | 9606.ENSP00000303211 | 9606.ENSP00000225275 | 0 | 0 | 0 | 0 | 0 | 0.064 | 0 | 0.41 | 0.424 |
| ACHE | SLC6A4 | 9606.ENSP00000303211 | 9606.ENSP00000261707 | 0 | 0 | 0 | 0 | 0 | 0 | 0 | 0.433 | 0.433 |
| ACHE | SOD1 | 9606.ENSP00000303211 | 9606.ENSP00000270142 | 0 | 0 | 0 | 0 | 0.061 | 0 | 0 | 0.526 | 0.535 |
| ACHE | SLC6A3 | 9606.ENSP00000303211 | 9606.ENSP00000270349 | 0 | 0 | 0 | 0 | 0.061 | 0 | 0 | 0.479 | 0.489 |
| ACHE | APP | 9606.ENSP00000303211 | 9606.ENSP00000284981 | 0 | 0 | 0 | 0 | 0.091 | 0.379 | 0 | 0.73 | 0.834 |
| ACHE | CCK | 9606.ENSP00000303211 | 9606.ENSP00000379472 | 0 | 0 | 0 | 0 | 0.099 | 0 | 0 | 0.366 | 0.404 |
| ACHE | TNF | 9606.ENSP00000303211 | 9606.ENSP00000398698 | 0 | 0 | 0 | 0 | 0 | 0 | 0 | 0.405 | 0.405 |
| ACHE | ADRA1D | 9606.ENSP00000303211 | 9606.ENSP00000368766 | 0 | 0 | 0 | 0 | 0.061 | 0 | 0 | 0.4 | 0.412 |
| ACHE | ADA | 9606.ENSP00000303211 | 9606.ENSP00000361965 | 0 | 0 | 0 | 0 | 0 | 0 | 0 | 0.412 | 0.412 |
| ACHE | GRIN1 | 9606.ENSP00000303211 | 9606.ENSP00000360608 | 0 | 0 | 0 | 0 | 0.188 | 0 | 0 | 0.314 | 0.42 |
| ACHE | ADRA1B | 9606.ENSP00000303211 | 9606.ENSP00000306662 | 0 | 0 | 0 | 0 | 0.071 | 0 | 0 | 0.407 | 0.425 |
| ACHE | F2 | 9606.ENSP00000303211 | 9606.ENSP00000308541 | 0 | 0 | 0 | 0 | 0.062 | 0.052 | 0 | 0.41 | 0.429 |
| ACHE | PTGS2 | 9606.ENSP00000303211 | 9606.ENSP00000356438 | 0 | 0 | 0 | 0 | 0 | 0.064 | 0 | 0.437 | 0.451 |
| ACHE | AKT1 | 9606.ENSP00000303211 | 9606.ENSP00000451828 | 0 | 0 | 0 | 0 | 0 | 0.062 | 0 | 0.448 | 0.46 |
| ACHE | IL6 | 9606.ENSP00000303211 | 9606.ENSP00000385675 | 0 | 0 | 0 | 0 | 0 | 0 | 0 | 0.503 | 0.503 |
| ACHE | INS | 9606.ENSP00000303211 | 9606.ENSP00000380432 | 0 | 0 | 0 | 0 | 0 | 0 | 0 | 0.528 | 0.528 |
| ACHE | CASP3 | 9606.ENSP00000303211 | 9606.ENSP00000311032 | 0 | 0 | 0 | 0 | 0 | 0 | 0 | 0.533 | 0.533 |
| ACHE | ADRB2 | 9606.ENSP00000303211 | 9606.ENSP00000305372 | 0 | 0 | 0 | 0 | 0 | 0 | 0 | 0.563 | 0.563 |
| ACHE | CHRNA2 | 9606.ENSP00000303211 | 9606.ENSP00000385026 | 0 | 0 | 0 | 0 | 0.049 | 0 | 0 | 0.631 | 0.634 |
| ACHE | BDNF | 9606.ENSP00000303211 | 9606.ENSP00000414303 | 0 | 0 | 0 | 0 | 0.052 | 0 | 0 | 0.653 | 0.657 |
| ACHE | MAOB | 9606.ENSP00000303211 | 9606.ENSP00000367309 | 0.045 | 0 | 0 | 0 | 0.107 | 0 | 0 | 0.673 | 0.697 |
| ADA | MPO | 9606.ENSP00000361965 | 9606.ENSP00000225275 | 0 | 0 | 0 | 0 | 0 | 0 | 0 | 0.498 | 0.497 |
| ADA | NR3C1 | 9606.ENSP00000361965 | 9606.ENSP00000231509 | 0 | 0 | 0 | 0 | 0 | 0.379 | 0 | 0.094 | 0.413 |
| ADA | CRP | 9606.ENSP00000361965 | 9606.ENSP00000255030 | 0 | 0 | 0 | 0 | 0 | 0 | 0 | 0.53 | 0.53 |
| ADA | GRIA2 | 9606.ENSP00000361965 | 9606.ENSP00000296526 | 0 | 0 | 0 | 0 | 0 | 0 | 0 | 0.53 | 0.53 |
| ADA | DPP4 | 9606.ENSP00000361965 | 9606.ENSP00000353731 | 0 | 0 | 0 | 0 | 0.063 | 0.379 | 0 | 0.871 | 0.918 |
| ADA | GABRA3 | 9606.ENSP00000361965 | 9606.ENSP00000359337 | 0 | 0 | 0 | 0 | 0 | 0.05 | 0 | 0.492 | 0.497 |
| ADA | IL6 | 9606.ENSP00000361965 | 9606.ENSP00000385675 | 0 | 0 | 0 | 0 | 0 | 0 | 0 | 0.421 | 0.42 |
| ADA | TNF | 9606.ENSP00000361965 | 9606.ENSP00000398698 | 0 | 0 | 0 | 0 | 0 | 0 | 0 | 0.449 | 0.449 |
| ADA | INS | 9606.ENSP00000361965 | 9606.ENSP00000380432 | 0 | 0 | 0 | 0 | 0 | 0 | 0 | 0.515 | 0.515 |
| ADRA1B | SLC6A2 | 9606.ENSP00000306662 | 9606.ENSP00000219833 | 0 | 0 | 0 | 0 | 0 | 0.073 | 0 | 0.583 | 0.597 |
| ADRA1B | SLC6A4 | 9606.ENSP00000306662 | 9606.ENSP00000261707 | 0 | 0 | 0 | 0 | 0.062 | 0.073 | 0 | 0.519 | 0.545 |
| ADRA1B | BCHE | 9606.ENSP00000306662 | 9606.ENSP00000264381 | 0 | 0 | 0 | 0 | 0 | 0 | 0 | 0.441 | 0.441 |
| ADRA1B | SLC6A3 | 9606.ENSP00000306662 | 9606.ENSP00000270349 | 0 | 0 | 0 | 0 | 0 | 0.073 | 0 | 0.407 | 0.427 |
| ADRA1B | APP | 9606.ENSP00000306662 | 9606.ENSP00000284981 | 0 | 0 | 0 | 0 | 0.098 | 0 | 0.9 | 0.185 | 0.92 |
| ADRA1B | ADRB2 | 9606.ENSP00000306662 | 9606.ENSP00000305372 | 0 | 0 | 0 | 0.801 | 0 | 0 | 0.9 | 0.553 | 0.91 |
| ADRA1B | ENSG00000196689 | 9606.ENSP00000306662 | 9606.ENSP00000459962 | 0 | 0 | 0 | 0 | 0 | 0.075 | 0 | 0.4 | 0.421 |
| ADRA1B | BDNF | 9606.ENSP00000306662 | 9606.ENSP00000414303 | 0 | 0 | 0 | 0 | 0.098 | 0 | 0 | 0.395 | 0.43 |
| ADRA1B | MAOB | 9606.ENSP00000306662 | 9606.ENSP00000367309 | 0 | 0 | 0 | 0 | 0.088 | 0 | 0 | 0.411 | 0.439 |
| ADRA1B | INS | 9606.ENSP00000306662 | 9606.ENSP00000380432 | 0 | 0 | 0 | 0 | 0 | 0 | 0 | 0.635 | 0.635 |
| ADRA1B | F2 | 9606.ENSP00000306662 | 9606.ENSP00000308541 | 0 | 0 | 0 | 0 | 0.065 | 0 | 0.9 | 0.081 | 0.906 |
| ADRA1B | ADRA1D | 9606.ENSP00000306662 | 9606.ENSP00000368766 | 0 | 0 | 0 | 0.932 | 0.111 | 0 | 0.9 | 0.797 | 0.912 |
| ADRA1B | CCK | 9606.ENSP00000306662 | 9606.ENSP00000379472 | 0 | 0 | 0 | 0 | 0.098 | 0 | 0.9 | 0.429 | 0.943 |
| ADRA1D | SLC6A2 | 9606.ENSP00000368766 | 9606.ENSP00000219833 | 0 | 0 | 0 | 0 | 0 | 0.073 | 0 | 0.609 | 0.622 |
| ADRA1D | SLC6A4 | 9606.ENSP00000368766 | 9606.ENSP00000261707 | 0 | 0 | 0 | 0 | 0 | 0.073 | 0 | 0.527 | 0.543 |
| ADRA1D | BCHE | 9606.ENSP00000368766 | 9606.ENSP00000264381 | 0 | 0 | 0 | 0 | 0 | 0 | 0 | 0.442 | 0.442 |
| ADRA1D | SLC6A3 | 9606.ENSP00000368766 | 9606.ENSP00000270349 | 0 | 0 | 0 | 0 | 0 | 0.073 | 0 | 0.453 | 0.471 |
| ADRA1D | APP | 9606.ENSP00000368766 | 9606.ENSP00000284981 | 0 | 0 | 0 | 0 | 0.08 | 0 | 0.9 | 0.11 | 0.911 |
| ADRA1D | F2 | 9606.ENSP00000368766 | 9606.ENSP00000308541 | 0 | 0 | 0 | 0 | 0.063 | 0 | 0.9 | 0.105 | 0.908 |
| ADRA1D | MAOB | 9606.ENSP00000368766 | 9606.ENSP00000367309 | 0 | 0 | 0 | 0 | 0 | 0 | 0 | 0.41 | 0.41 |
| ADRA1D | ENSG00000196689 | 9606.ENSP00000368766 | 9606.ENSP00000459962 | 0 | 0 | 0 | 0 | 0 | 0.075 | 0 | 0.4 | 0.421 |
| ADRA1D | BDNF | 9606.ENSP00000368766 | 9606.ENSP00000414303 | 0 | 0 | 0 | 0 | 0.077 | 0 | 0 | 0.41 | 0.432 |
| ADRA1D | INS | 9606.ENSP00000368766 | 9606.ENSP00000380432 | 0 | 0 | 0 | 0 | 0 | 0 | 0 | 0.634 | 0.634 |
| ADRA1D | CCK | 9606.ENSP00000368766 | 9606.ENSP00000379472 | 0 | 0 | 0 | 0 | 0.095 | 0 | 0.9 | 0.426 | 0.943 |
| ADRB2 | SLC6A2 | 9606.ENSP00000305372 | 9606.ENSP00000219833 | 0 | 0 | 0 | 0 | 0 | 0.073 | 0 | 0.538 | 0.553 |
| ADRB2 | NR3C1 | 9606.ENSP00000305372 | 9606.ENSP00000231509 | 0 | 0 | 0 | 0 | 0 | 0 | 0 | 0.478 | 0.478 |
| ADRB2 | SLC6A4 | 9606.ENSP00000305372 | 9606.ENSP00000261707 | 0 | 0 | 0 | 0 | 0 | 0.073 | 0 | 0.511 | 0.527 |
| ADRB2 | BCHE | 9606.ENSP00000305372 | 9606.ENSP00000264381 | 0 | 0 | 0 | 0 | 0 | 0 | 0 | 0.59 | 0.59 |
| ADRB2 | ERBB2 | 9606.ENSP00000305372 | 9606.ENSP00000269571 | 0 | 0 | 0 | 0 | 0 | 0.299 | 0 | 0.83 | 0.876 |
| ADRB2 | PPARG | 9606.ENSP00000305372 | 9606.ENSP00000287820 | 0 | 0 | 0 | 0 | 0 | 0 | 0 | 0.416 | 0.416 |
| ADRB2 | PTGIR | 9606.ENSP00000305372 | 9606.ENSP00000291294 | 0 | 0 | 0 | 0 | 0 | 0 | 0.9 | 0.23 | 0.919 |
| ADRB2 | CCK | 9606.ENSP00000305372 | 9606.ENSP00000379472 | 0 | 0 | 0 | 0 | 0 | 0 | 0 | 0.421 | 0.42 |
| ADRB2 | ENSG00000196689 | 9606.ENSP00000305372 | 9606.ENSP00000459962 | 0 | 0 | 0 | 0 | 0 | 0.075 | 0 | 0.421 | 0.441 |
| ADRB2 | AKT1 | 9606.ENSP00000305372 | 9606.ENSP00000451828 | 0 | 0 | 0 | 0 | 0 | 0.072 | 0 | 0.44 | 0.458 |
| ADRB2 | IL6 | 9606.ENSP00000305372 | 9606.ENSP00000385675 | 0 | 0 | 0 | 0 | 0.063 | 0 | 0 | 0.447 | 0.459 |
| ADRB2 | TNF | 9606.ENSP00000305372 | 9606.ENSP00000398698 | 0 | 0 | 0 | 0 | 0.065 | 0 | 0 | 0.448 | 0.461 |
| ADRB2 | BDNF | 9606.ENSP00000305372 | 9606.ENSP00000414303 | 0 | 0 | 0 | 0 | 0 | 0 | 0 | 0.639 | 0.639 |
| ADRB2 | INS | 9606.ENSP00000305372 | 9606.ENSP00000380432 | 0 | 0 | 0 | 0 | 0 | 0 | 0 | 0.668 | 0.668 |
| ADRB2 | F2 | 9606.ENSP00000305372 | 9606.ENSP00000308541 | 0 | 0 | 0 | 0 | 0 | 0 | 0.9 | 0.143 | 0.91 |
| ADRB2 | CHRM2 | 9606.ENSP00000305372 | 9606.ENSP00000399745 | 0 | 0 | 0 | 0.652 | 0.052 | 0 | 0.9 | 0.563 | 0.919 |
| AKR1C1 | SP1 | 9606.ENSP00000370254 | 9606.ENSP00000329357 | 0 | 0 | 0 | 0 | 0 | 0.062 | 0 | 0.434 | 0.446 |
| AKR1C1 | HSD17B1 | 9606.ENSP00000370254 | 9606.ENSP00000466799 | 0.041 | 0 | 0 | 0 | 0.062 | 0 | 0 | 0.458 | 0.47 |
| AKT1 | SERPINE1 | 9606.ENSP00000451828 | 9606.ENSP00000223095 | 0 | 0 | 0 | 0 | 0 | 0 | 0 | 0.761 | 0.761 |
| AKT1 | MPO | 9606.ENSP00000451828 | 9606.ENSP00000225275 | 0 | 0 | 0 | 0 | 0 | 0.058 | 0 | 0.559 | 0.567 |
| AKT1 | NFKB1 | 9606.ENSP00000451828 | 9606.ENSP00000226574 | 0 | 0 | 0 | 0 | 0.065 | 0.261 | 0.9 | 0.525 | 0.962 |
| AKT1 | NR3C1 | 9606.ENSP00000451828 | 9606.ENSP00000231509 | 0 | 0 | 0 | 0 | 0 | 0.407 | 0 | 0.561 | 0.728 |
| AKT1 | CRP | 9606.ENSP00000451828 | 9606.ENSP00000255030 | 0 | 0 | 0 | 0 | 0 | 0 | 0 | 0.545 | 0.545 |
| AKT1 | ERBB2 | 9606.ENSP00000451828 | 9606.ENSP00000269571 | 0 | 0 | 0 | 0.556 | 0.053 | 0.543 | 0 | 0.943 | 0.737 |
| AKT1 | SOD1 | 9606.ENSP00000451828 | 9606.ENSP00000270142 | 0 | 0 | 0 | 0 | 0 | 0.064 | 0.9 | 0.64 | 0.963 |
| AKT1 | SLC6A3 | 9606.ENSP00000451828 | 9606.ENSP00000270349 | 0 | 0 | 0 | 0 | 0.062 | 0 | 0 | 0.4 | 0.413 |
| AKT1 | APP | 9606.ENSP00000451828 | 9606.ENSP00000284981 | 0 | 0 | 0 | 0 | 0.063 | 0.07 | 0 | 0.62 | 0.64 |
| AKT1 | PPARG | 9606.ENSP00000451828 | 9606.ENSP00000287820 | 0 | 0 | 0 | 0 | 0.061 | 0.085 | 0 | 0.674 | 0.695 |
| AKT1 | HMGCR | 9606.ENSP00000451828 | 9606.ENSP00000287936 | 0 | 0 | 0 | 0 | 0 | 0.052 | 0 | 0.521 | 0.526 |
| AKT1 | P2RY12 | 9606.ENSP00000451828 | 9606.ENSP00000307259 | 0 | 0 | 0 | 0 | 0 | 0 | 0 | 0.4 | 0.4 |
| AKT1 | F2 | 9606.ENSP00000451828 | 9606.ENSP00000308541 | 0 | 0 | 0 | 0 | 0 | 0.05 | 0 | 0.662 | 0.665 |
| AKT1 | PLG | 9606.ENSP00000451828 | 9606.ENSP00000308938 | 0 | 0 | 0 | 0 | 0.061 | 0.05 | 0 | 0.608 | 0.62 |
| AKT1 | LPL | 9606.ENSP00000451828 | 9606.ENSP00000309757 | 0 | 0 | 0 | 0 | 0 | 0 | 0 | 0.501 | 0.501 |
| AKT1 | CASP3 | 9606.ENSP00000451828 | 9606.ENSP00000311032 | 0 | 0 | 0 | 0 | 0.055 | 0.393 | 0 | 0.95 | 0.969 |
| AKT1 | GSK3B | 9606.ENSP00000451828 | 9606.ENSP00000324806 | 0 | 0 | 0.218 | 0.576 | 0.049 | 0.995 | 0.9 | 0.918 | 0.999 |
| AKT1 | PGR | 9606.ENSP00000451828 | 9606.ENSP00000325120 | 0 | 0 | 0 | 0 | 0.061 | 0.085 | 0 | 0.661 | 0.683 |
| AKT1 | NOS2 | 9606.ENSP00000451828 | 9606.ENSP00000327251 | 0 | 0 | 0 | 0 | 0.063 | 0.065 | 0.8 | 0.543 | 0.909 |
| AKT1 | SP1 | 9606.ENSP00000451828 | 9606.ENSP00000329357 | 0 | 0 | 0 | 0 | 0.055 | 0.08 | 0 | 0.707 | 0.723 |
| AKT1 | HSP90AA1 | 9606.ENSP00000451828 | 9606.ENSP00000335153 | 0 | 0 | 0 | 0 | 0.061 | 0.806 | 0.9 | 0.92 | 0.998 |
| AKT1 | ESR2 | 9606.ENSP00000451828 | 9606.ENSP00000343925 | 0 | 0 | 0 | 0 | 0 | 0.407 | 0 | 0.722 | 0.828 |
| AKT1 | DPP4 | 9606.ENSP00000451828 | 9606.ENSP00000353731 | 0 | 0 | 0 | 0 | 0.077 | 0 | 0 | 0.501 | 0.52 |
| AKT1 | PTGS2 | 9606.ENSP00000451828 | 9606.ENSP00000356438 | 0 | 0 | 0 | 0 | 0.062 | 0.058 | 0 | 0.848 | 0.854 |
| AKT1 | NFKB2 | 9606.ENSP00000451828 | 9606.ENSP00000358983 | 0 | 0 | 0 | 0 | 0.084 | 0.157 | 0 | 0.473 | 0.557 |
| AKT1 | GRIN1 | 9606.ENSP00000451828 | 9606.ENSP00000360608 | 0 | 0 | 0 | 0 | 0 | 0.128 | 0 | 0.375 | 0.432 |
| AKT1 | PLAU | 9606.ENSP00000451828 | 9606.ENSP00000361850 | 0 | 0 | 0 | 0 | 0 | 0.05 | 0 | 0.536 | 0.541 |
| AKT1 | ALOX5 | 9606.ENSP00000451828 | 9606.ENSP00000363512 | 0 | 0 | 0 | 0 | 0 | 0 | 0 | 0.429 | 0.429 |
| AKT1 | AR | 9606.ENSP00000451828 | 9606.ENSP00000363822 | 0 | 0 | 0 | 0 | 0 | 0.407 | 0 | 0.89 | 0.932 |
| AKT1 | GRIN2A | 9606.ENSP00000451828 | 9606.ENSP00000379818 | 0 | 0 | 0 | 0 | 0 | 0.105 | 0 | 0.442 | 0.48 |
| AKT1 | INS | 9606.ENSP00000451828 | 9606.ENSP00000380432 | 0 | 0 | 0 | 0 | 0 | 0.262 | 0.9 | 0.967 | 0.997 |
| AKT1 | BCL2 | 9606.ENSP00000451828 | 9606.ENSP00000381185 | 0 | 0 | 0 | 0 | 0.061 | 0 | 0.9 | 0.426 | 0.941 |
| AKT1 | RELA | 9606.ENSP00000451828 | 9606.ENSP00000384273 | 0 | 0 | 0 | 0 | 0.086 | 0 | 0 | 0.776 | 0.786 |
| AKT1 | PPARA | 9606.ENSP00000451828 | 9606.ENSP00000385523 | 0 | 0 | 0 | 0 | 0 | 0.085 | 0 | 0.471 | 0.495 |
| AKT1 | IL6 | 9606.ENSP00000451828 | 9606.ENSP00000385675 | 0 | 0 | 0 | 0 | 0.055 | 0 | 0 | 0.886 | 0.888 |
| AKT1 | TNF | 9606.ENSP00000451828 | 9606.ENSP00000398698 | 0 | 0 | 0 | 0 | 0 | 0.05 | 0 | 0.874 | 0.876 |
| AKT1 | ESR1 | 9606.ENSP00000451828 | 9606.ENSP00000405330 | 0 | 0 | 0 | 0 | 0.055 | 0.407 | 0.9 | 0.867 | 0.991 |
| AKT1 | IL10 | 9606.ENSP00000451828 | 9606.ENSP00000412237 | 0 | 0 | 0 | 0 | 0 | 0.079 | 0 | 0.821 | 0.828 |
| AKT1 | BDNF | 9606.ENSP00000451828 | 9606.ENSP00000414303 | 0 | 0 | 0 | 0 | 0 | 0.185 | 0 | 0.835 | 0.86 |
| AKT1 | SLC2A1 | 9606.ENSP00000451828 | 9606.ENSP00000416293 | 0 | 0 | 0 | 0 | 0 | 0.361 | 0 | 0.808 | 0.872 |
| AKT1 | VDR | 9606.ENSP00000451828 | 9606.ENSP00000447173 | 0 | 0 | 0 | 0 | 0 | 0.085 | 0 | 0.456 | 0.481 |
| AKT1 | ENSG00000196689 | 9606.ENSP00000451828 | 9606.ENSP00000459962 | 0 | 0 | 0 | 0 | 0 | 0 | 0 | 0.419 | 0.419 |
| AKT1 | GRIN2B | 9606.ENSP00000451828 | 9606.ENSP00000477455 | 0 | 0 | 0 | 0 | 0 | 0.127 | 0 | 0.675 | 0.704 |
| AKT1 | ITGB3 | 9606.ENSP00000451828 | 9606.ENSP00000452786 | 0 | 0 | 0 | 0 | 0.08 | 0.34 | 0.9 | 0.425 | 0.96 |
| ALOX5 | MPO | 9606.ENSP00000363512 | 9606.ENSP00000225275 | 0 | 0 | 0 | 0 | 0.12 | 0 | 0 | 0.543 | 0.581 |
| ALOX5 | PPARG | 9606.ENSP00000363512 | 9606.ENSP00000287820 | 0 | 0 | 0 | 0 | 0 | 0 | 0 | 0.421 | 0.42 |
| ALOX5 | CASP3 | 9606.ENSP00000363512 | 9606.ENSP00000311032 | 0 | 0 | 0 | 0 | 0 | 0 | 0 | 0.416 | 0.416 |
| ALOX5 | PTGS1 | 9606.ENSP00000363512 | 9606.ENSP00000354612 | 0 | 0 | 0 | 0 | 0.095 | 0 | 0.9 | 0.728 | 0.973 |
| ALOX5 | PTGS2 | 9606.ENSP00000363512 | 9606.ENSP00000356438 | 0 | 0 | 0 | 0 | 0.08 | 0 | 0.9 | 0.758 | 0.975 |
| ALOX5 | HSD17B1 | 9606.ENSP00000363512 | 9606.ENSP00000466799 | 0 | 0 | 0 | 0 | 0 | 0.426 | 0 | 0 | 0.426 |
| ALOX5 | RELA | 9606.ENSP00000363512 | 9606.ENSP00000384273 | 0 | 0 | 0 | 0 | 0 | 0.379 | 0 | 0.189 | 0.474 |
| ALOX5 | IL10 | 9606.ENSP00000363512 | 9606.ENSP00000412237 | 0 | 0 | 0 | 0 | 0 | 0 | 0 | 0.51 | 0.51 |
| ALOX5 | IL6 | 9606.ENSP00000363512 | 9606.ENSP00000385675 | 0 | 0 | 0 | 0 | 0 | 0 | 0 | 0.563 | 0.563 |
| ALOX5 | TNF | 9606.ENSP00000363512 | 9606.ENSP00000398698 | 0 | 0 | 0 | 0 | 0.121 | 0 | 0 | 0.56 | 0.597 |
| ALOX5 | INS | 9606.ENSP00000363512 | 9606.ENSP00000380432 | 0 | 0 | 0 | 0 | 0 | 0 | 0 | 0.64 | 0.64 |
| APP | CETP | 9606.ENSP00000284981 | 9606.ENSP00000200676 | 0 | 0 | 0 | 0 | 0 | 0 | 0.72 | 0.136 | 0.747 |
| APP | PON1 | 9606.ENSP00000284981 | 9606.ENSP00000222381 | 0 | 0 | 0 | 0 | 0 | 0 | 0.72 | 0.322 | 0.802 |
| APP | SERPINE1 | 9606.ENSP00000284981 | 9606.ENSP00000223095 | 0 | 0 | 0 | 0 | 0 | 0 | 0.9 | 0.25 | 0.921 |
| APP | NFKB1 | 9606.ENSP00000284981 | 9606.ENSP00000226574 | 0 | 0 | 0 | 0 | 0 | 0 | 0.9 | 0.21 | 0.917 |
| APP | CRP | 9606.ENSP00000284981 | 9606.ENSP00000255030 | 0 | 0 | 0 | 0 | 0 | 0 | 0 | 0.45 | 0.45 |
| APP | BCHE | 9606.ENSP00000284981 | 9606.ENSP00000264381 | 0 | 0 | 0 | 0 | 0.086 | 0 | 0 | 0.694 | 0.709 |
| APP | ERBB2 | 9606.ENSP00000284981 | 9606.ENSP00000269571 | 0 | 0 | 0 | 0 | 0.063 | 0.042 | 0 | 0.433 | 0.447 |
| APP | SOD1 | 9606.ENSP00000284981 | 9606.ENSP00000270142 | 0 | 0 | 0 | 0 | 0.05 | 0 | 0 | 0.735 | 0.738 |
| APP | PPARG | 9606.ENSP00000284981 | 9606.ENSP00000287820 | 0 | 0 | 0 | 0 | 0 | 0 | 0 | 0.417 | 0.416 |
| APP | ESR1 | 9606.ENSP00000284981 | 9606.ENSP00000405330 | 0 | 0 | 0 | 0 | 0 | 0 | 0 | 0.44 | 0.44 |
| APP | MAOB | 9606.ENSP00000284981 | 9606.ENSP00000367309 | 0 | 0 | 0 | 0 | 0.083 | 0 | 0 | 0.435 | 0.46 |
| APP | GRIA2 | 9606.ENSP00000284981 | 9606.ENSP00000296526 | 0 | 0 | 0 | 0 | 0.097 | 0 | 0 | 0.437 | 0.47 |
| APP | IL10 | 9606.ENSP00000284981 | 9606.ENSP00000412237 | 0 | 0 | 0 | 0 | 0 | 0 | 0 | 0.478 | 0.478 |
| APP | GRIN2A | 9606.ENSP00000284981 | 9606.ENSP00000379818 | 0 | 0 | 0 | 0 | 0.086 | 0 | 0 | 0.481 | 0.505 |
| APP | HMGCR | 9606.ENSP00000284981 | 9606.ENSP00000287936 | 0 | 0 | 0 | 0 | 0 | 0 | 0 | 0.576 | 0.577 |
| APP | GRIN2B | 9606.ENSP00000284981 | 9606.ENSP00000477455 | 0 | 0 | 0 | 0 | 0.062 | 0 | 0 | 0.571 | 0.58 |
| APP | HSP90AA1 | 9606.ENSP00000284981 | 9606.ENSP00000335153 | 0 | 0 | 0 | 0 | 0 | 0.32 | 0 | 0.419 | 0.588 |
| APP | GRIN1 | 9606.ENSP00000284981 | 9606.ENSP00000360608 | 0 | 0 | 0 | 0 | 0.112 | 0 | 0 | 0.599 | 0.629 |
| APP | LPL | 9606.ENSP00000284981 | 9606.ENSP00000309757 | 0 | 0 | 0 | 0 | 0.061 | 0 | 0.54 | 0.212 | 0.629 |
| APP | PTGS2 | 9606.ENSP00000284981 | 9606.ENSP00000356438 | 0 | 0 | 0 | 0 | 0.061 | 0.379 | 0 | 0.454 | 0.654 |
| APP | PRSS3 | 9606.ENSP00000284981 | 9606.ENSP00000354280 | 0 | 0 | 0 | 0 | 0 | 0.559 | 0 | 0.254 | 0.656 |
| APP | TNF | 9606.ENSP00000284981 | 9606.ENSP00000398698 | 0 | 0 | 0 | 0 | 0 | 0.302 | 0 | 0.725 | 0.8 |
| APP | BDNF | 9606.ENSP00000284981 | 9606.ENSP00000414303 | 0 | 0 | 0 | 0 | 0.092 | 0 | 0 | 0.818 | 0.828 |
| APP | GSK3B | 9606.ENSP00000284981 | 9606.ENSP00000324806 | 0 | 0 | 0 | 0 | 0.057 | 0.397 | 0 | 0.748 | 0.845 |
| APP | CASP3 | 9606.ENSP00000284981 | 9606.ENSP00000311032 | 0 | 0 | 0 | 0 | 0.062 | 0.682 | 0 | 0.665 | 0.891 |
| APP | CHRM2 | 9606.ENSP00000284981 | 9606.ENSP00000399745 | 0 | 0 | 0 | 0 | 0.062 | 0 | 0.9 | 0.144 | 0.912 |
| APP | PYY | 9606.ENSP00000284981 | 9606.ENSP00000353198 | 0 | 0 | 0 | 0 | 0 | 0 | 0.9 | 0.166 | 0.913 |
| APP | CCK | 9606.ENSP00000284981 | 9606.ENSP00000379472 | 0 | 0 | 0 | 0 | 0.091 | 0 | 0.9 | 0.136 | 0.914 |
| APP | NFKB2 | 9606.ENSP00000284981 | 9606.ENSP00000358983 | 0 | 0 | 0 | 0 | 0 | 0 | 0.9 | 0.19 | 0.915 |
| APP | P2RY12 | 9606.ENSP00000284981 | 9606.ENSP00000307259 | 0 | 0 | 0 | 0 | 0 | 0 | 0.9 | 0.194 | 0.915 |
| APP | RELA | 9606.ENSP00000284981 | 9606.ENSP00000384273 | 0 | 0 | 0 | 0 | 0 | 0 | 0.9 | 0.408 | 0.938 |
| APP | PLG | 9606.ENSP00000284981 | 9606.ENSP00000308938 | 0 | 0 | 0 | 0 | 0 | 0.132 | 0.9 | 0.384 | 0.941 |
| APP | F2 | 9606.ENSP00000284981 | 9606.ENSP00000308541 | 0 | 0 | 0 | 0 | 0 | 0.132 | 0.9 | 0.413 | 0.944 |
| APP | IL6 | 9606.ENSP00000284981 | 9606.ENSP00000385675 | 0 | 0 | 0 | 0 | 0 | 0 | 0.9 | 0.589 | 0.957 |
| APP | INS | 9606.ENSP00000284981 | 9606.ENSP00000380432 | 0 | 0 | 0 | 0 | 0 | 0 | 0.9 | 0.669 | 0.965 |
| AR | NFKB1 | 9606.ENSP00000363822 | 9606.ENSP00000226574 | 0 | 0 | 0 | 0 | 0 | 0.379 | 0 | 0.205 | 0.485 |
| AR | NR3C1 | 9606.ENSP00000363822 | 9606.ENSP00000231509 | 0 | 0 | 0 | 0.816 | 0 | 0.379 | 0.9 | 0.806 | 0.944 |
| AR | ERBB2 | 9606.ENSP00000363822 | 9606.ENSP00000269571 | 0 | 0 | 0 | 0 | 0.067 | 0.077 | 0 | 0.829 | 0.84 |
| AR | SOD1 | 9606.ENSP00000363822 | 9606.ENSP00000270142 | 0 | 0 | 0 | 0 | 0 | 0 | 0 | 0.625 | 0.625 |
| AR | GRIA2 | 9606.ENSP00000363822 | 9606.ENSP00000296526 | 0 | 0 | 0 | 0 | 0 | 0 | 0 | 0.456 | 0.456 |
| AR | CASP3 | 9606.ENSP00000363822 | 9606.ENSP00000311032 | 0 | 0 | 0 | 0 | 0 | 0.39 | 0 | 0.6 | 0.746 |
| AR | GSK3B | 9606.ENSP00000363822 | 9606.ENSP00000324806 | 0 | 0 | 0 | 0 | 0 | 0.407 | 0.9 | 0.49 | 0.967 |
| AR | PGR | 9606.ENSP00000363822 | 9606.ENSP00000325120 | 0 | 0 | 0 | 0.813 | 0.069 | 0 | 0.9 | 0.904 | 0.919 |
| AR | SP1 | 9606.ENSP00000363822 | 9606.ENSP00000329357 | 0 | 0 | 0 | 0 | 0 | 0.424 | 0 | 0.65 | 0.79 |
| AR | HSP90AA1 | 9606.ENSP00000363822 | 9606.ENSP00000335153 | 0 | 0 | 0 | 0 | 0 | 0.474 | 0.9 | 0.803 | 0.988 |
| AR | PTGS2 | 9606.ENSP00000363822 | 9606.ENSP00000356438 | 0 | 0 | 0 | 0 | 0 | 0.05 | 0 | 0.437 | 0.442 |
| AR | NFKB2 | 9606.ENSP00000363822 | 9606.ENSP00000358983 | 0 | 0 | 0 | 0 | 0 | 0 | 0 | 0.559 | 0.559 |
| AR | HSD17B1 | 9606.ENSP00000363822 | 9606.ENSP00000466799 | 0 | 0 | 0 | 0 | 0 | 0 | 0 | 0.499 | 0.499 |
| AR | ESR1 | 9606.ENSP00000363822 | 9606.ENSP00000405330 | 0 | 0 | 0 | 0.656 | 0.062 | 0.379 | 0 | 0.936 | 0.587 |
| AR | BDNF | 9606.ENSP00000363822 | 9606.ENSP00000414303 | 0 | 0 | 0 | 0 | 0 | 0 | 0 | 0.625 | 0.625 |
| AR | TNF | 9606.ENSP00000363822 | 9606.ENSP00000398698 | 0 | 0 | 0 | 0 | 0 | 0 | 0 | 0.639 | 0.639 |
| AR | INS | 9606.ENSP00000363822 | 9606.ENSP00000380432 | 0 | 0 | 0 | 0 | 0 | 0 | 0 | 0.669 | 0.669 |
| AR | SHBG | 9606.ENSP00000363822 | 9606.ENSP00000369816 | 0 | 0 | 0 | 0 | 0 | 0 | 0 | 0.742 | 0.742 |
| AR | RELA | 9606.ENSP00000363822 | 9606.ENSP00000384273 | 0 | 0 | 0 | 0 | 0 | 0.393 | 0 | 0.625 | 0.763 |
| AR | IL6 | 9606.ENSP00000363822 | 9606.ENSP00000385675 | 0 | 0 | 0 | 0 | 0 | 0 | 0 | 0.787 | 0.787 |
| BCHE | PON1 | 9606.ENSP00000264381 | 9606.ENSP00000222381 | 0 | 0 | 0 | 0 | 0.062 | 0 | 0 | 0.713 | 0.72 |
| BCHE | CRP | 9606.ENSP00000264381 | 9606.ENSP00000255030 | 0 | 0 | 0 | 0 | 0.051 | 0 | 0 | 0.555 | 0.559 |
| BCHE | SP1 | 9606.ENSP00000264381 | 9606.ENSP00000329357 | 0 | 0 | 0 | 0 | 0 | 0.379 | 0 | 0.076 | 0.402 |
| BCHE | IL6 | 9606.ENSP00000264381 | 9606.ENSP00000385675 | 0 | 0 | 0 | 0 | 0 | 0 | 0 | 0.437 | 0.437 |
| BCHE | SLC6A3 | 9606.ENSP00000264381 | 9606.ENSP00000270349 | 0 | 0 | 0 | 0 | 0 | 0 | 0 | 0.456 | 0.456 |
| BCHE | SOD1 | 9606.ENSP00000264381 | 9606.ENSP00000270142 | 0 | 0 | 0 | 0 | 0.061 | 0 | 0 | 0.488 | 0.499 |
| BCHE | BDNF | 9606.ENSP00000264381 | 9606.ENSP00000414303 | 0 | 0 | 0 | 0 | 0.052 | 0 | 0 | 0.512 | 0.518 |
| BCHE | INS | 9606.ENSP00000264381 | 9606.ENSP00000380432 | 0 | 0 | 0 | 0 | 0 | 0 | 0 | 0.54 | 0.54 |
| BCHE | F2 | 9606.ENSP00000264381 | 9606.ENSP00000308541 | 0 | 0 | 0 | 0 | 0.126 | 0.052 | 0 | 0.669 | 0.701 |
| BCHE | MAOB | 9606.ENSP00000264381 | 9606.ENSP00000367309 | 0.045 | 0 | 0 | 0 | 0.086 | 0 | 0 | 0.694 | 0.71 |
| BCL2 | SOD1 | 9606.ENSP00000381185 | 9606.ENSP00000270142 | 0 | 0 | 0 | 0 | 0 | 0.379 | 0.8 | 0.117 | 0.88 |
| BCL2 | CASP3 | 9606.ENSP00000381185 | 9606.ENSP00000311032 | 0 | 0 | 0 | 0 | 0 | 0.461 | 0 | 0.578 | 0.763 |
| BCL2 | HSP90AA1 | 9606.ENSP00000381185 | 9606.ENSP00000335153 | 0 | 0 | 0 | 0 | 0 | 0.379 | 0 | 0.13 | 0.437 |
| BCL2 | ESR1 | 9606.ENSP00000381185 | 9606.ENSP00000405330 | 0 | 0 | 0 | 0 | 0.069 | 0.064 | 0.9 | 0.244 | 0.925 |
| BDNF | SLC6A2 | 9606.ENSP00000414303 | 9606.ENSP00000219833 | 0 | 0 | 0 | 0 | 0.061 | 0 | 0 | 0.51 | 0.52 |
| BDNF | NR3C1 | 9606.ENSP00000414303 | 9606.ENSP00000231509 | 0 | 0 | 0 | 0 | 0 | 0 | 0 | 0.802 | 0.802 |
| BDNF | CRP | 9606.ENSP00000414303 | 9606.ENSP00000255030 | 0 | 0 | 0 | 0 | 0 | 0 | 0 | 0.671 | 0.671 |
| BDNF | SLC6A4 | 9606.ENSP00000414303 | 9606.ENSP00000261707 | 0 | 0 | 0 | 0 | 0.061 | 0 | 0 | 0.788 | 0.792 |
| BDNF | GRIN2D | 9606.ENSP00000414303 | 9606.ENSP00000263269 | 0 | 0 | 0 | 0 | 0.062 | 0 | 0 | 0.398 | 0.411 |
| BDNF | ERBB2 | 9606.ENSP00000414303 | 9606.ENSP00000269571 | 0 | 0 | 0 | 0 | 0 | 0 | 0.6 | 0.341 | 0.725 |
| BDNF | SOD1 | 9606.ENSP00000414303 | 9606.ENSP00000270142 | 0 | 0 | 0 | 0 | 0 | 0 | 0 | 0.56 | 0.56 |
| BDNF | SLC6A3 | 9606.ENSP00000414303 | 9606.ENSP00000270349 | 0 | 0 | 0 | 0 | 0.061 | 0 | 0 | 0.669 | 0.675 |
| BDNF | HMGCR | 9606.ENSP00000414303 | 9606.ENSP00000287936 | 0 | 0 | 0 | 0 | 0 | 0 | 0 | 0.504 | 0.504 |
| BDNF | GRIA2 | 9606.ENSP00000414303 | 9606.ENSP00000296526 | 0 | 0 | 0 | 0 | 0.082 | 0 | 0 | 0.577 | 0.595 |
| BDNF | PLG | 9606.ENSP00000414303 | 9606.ENSP00000308938 | 0 | 0 | 0 | 0 | 0 | 0.102 | 0.9 | 0.547 | 0.955 |
| BDNF | CASP3 | 9606.ENSP00000414303 | 9606.ENSP00000311032 | 0 | 0 | 0 | 0 | 0 | 0 | 0 | 0.683 | 0.683 |
| BDNF | GSK3B | 9606.ENSP00000414303 | 9606.ENSP00000324806 | 0 | 0 | 0 | 0 | 0 | 0.185 | 0 | 0.522 | 0.594 |
| BDNF | ESR2 | 9606.ENSP00000414303 | 9606.ENSP00000343925 | 0 | 0 | 0 | 0 | 0 | 0 | 0 | 0.405 | 0.405 |
| BDNF | PTGS2 | 9606.ENSP00000414303 | 9606.ENSP00000356438 | 0 | 0 | 0 | 0 | 0.061 | 0 | 0 | 0.521 | 0.531 |
| BDNF | GRIN1 | 9606.ENSP00000414303 | 9606.ENSP00000360608 | 0 | 0 | 0 | 0 | 0.083 | 0 | 0 | 0.617 | 0.634 |
| BDNF | MAOB | 9606.ENSP00000414303 | 9606.ENSP00000367309 | 0 | 0 | 0 | 0 | 0.061 | 0 | 0 | 0.511 | 0.521 |
| BDNF | CCK | 9606.ENSP00000414303 | 9606.ENSP00000379472 | 0 | 0 | 0 | 0 | 0.062 | 0 | 0 | 0.497 | 0.508 |
| BDNF | GRIN2A | 9606.ENSP00000414303 | 9606.ENSP00000379818 | 0 | 0 | 0 | 0 | 0.081 | 0 | 0 | 0.808 | 0.817 |
| BDNF | INS | 9606.ENSP00000414303 | 9606.ENSP00000380432 | 0 | 0 | 0 | 0 | 0 | 0 | 0 | 0.715 | 0.715 |
| BDNF | IL6 | 9606.ENSP00000414303 | 9606.ENSP00000385675 | 0 | 0 | 0 | 0 | 0 | 0 | 0 | 0.811 | 0.811 |
| BDNF | TNF | 9606.ENSP00000414303 | 9606.ENSP00000398698 | 0 | 0 | 0 | 0 | 0 | 0 | 0 | 0.805 | 0.805 |
| BDNF | ESR1 | 9606.ENSP00000414303 | 9606.ENSP00000405330 | 0 | 0 | 0 | 0 | 0 | 0.379 | 0 | 0.52 | 0.689 |
| BDNF | IL10 | 9606.ENSP00000414303 | 9606.ENSP00000412237 | 0 | 0 | 0 | 0 | 0 | 0 | 0 | 0.613 | 0.613 |
| BDNF | ENSG00000196689 | 9606.ENSP00000414303 | 9606.ENSP00000459962 | 0 | 0 | 0 | 0 | 0 | 0 | 0 | 0.589 | 0.589 |
| BDNF | GRIN2B | 9606.ENSP00000414303 | 9606.ENSP00000477455 | 0 | 0 | 0 | 0 | 0.062 | 0 | 0.9 | 0.829 | 0.982 |
| CASP3 | SERPINE1 | 9606.ENSP00000311032 | 9606.ENSP00000223095 | 0 | 0 | 0 | 0 | 0 | 0 | 0 | 0.455 | 0.455 |
| CASP3 | MPO | 9606.ENSP00000311032 | 9606.ENSP00000225275 | 0 | 0 | 0 | 0 | 0 | 0 | 0 | 0.699 | 0.699 |
| CASP3 | NFKB1 | 9606.ENSP00000311032 | 9606.ENSP00000226574 | 0 | 0 | 0 | 0 | 0 | 0.178 | 0 | 0.451 | 0.529 |
| CASP3 | NR3C1 | 9606.ENSP00000311032 | 9606.ENSP00000231509 | 0 | 0 | 0 | 0 | 0 | 0.058 | 0 | 0.415 | 0.425 |
| CASP3 | CRP | 9606.ENSP00000311032 | 9606.ENSP00000255030 | 0 | 0 | 0 | 0 | 0 | 0 | 0 | 0.471 | 0.471 |
| CASP3 | ERBB2 | 9606.ENSP00000311032 | 9606.ENSP00000269571 | 0 | 0 | 0 | 0 | 0 | 0.05 | 0 | 0.691 | 0.694 |
| CASP3 | SOD1 | 9606.ENSP00000311032 | 9606.ENSP00000270142 | 0 | 0 | 0 | 0 | 0 | 0.092 | 0 | 0.63 | 0.65 |
| CASP3 | PPARG | 9606.ENSP00000311032 | 9606.ENSP00000287820 | 0 | 0 | 0 | 0 | 0 | 0.058 | 0 | 0.571 | 0.578 |
| CASP3 | F2 | 9606.ENSP00000311032 | 9606.ENSP00000308541 | 0 | 0 | 0 | 0 | 0 | 0.056 | 0 | 0.443 | 0.451 |
| CASP3 | PLG | 9606.ENSP00000311032 | 9606.ENSP00000308938 | 0 | 0 | 0 | 0 | 0.063 | 0.056 | 0 | 0.477 | 0.497 |
| CASP3 | ENSG00000196689 | 9606.ENSP00000311032 | 9606.ENSP00000459962 | 0 | 0 | 0 | 0 | 0 | 0 | 0 | 0.412 | 0.412 |
| CASP3 | MAOB | 9606.ENSP00000311032 | 9606.ENSP00000367309 | 0 | 0 | 0 | 0 | 0 | 0 | 0 | 0.417 | 0.417 |
| CASP3 | GRIN2B | 9606.ENSP00000311032 | 9606.ENSP00000477455 | 0 | 0 | 0 | 0 | 0 | 0.056 | 0 | 0.42 | 0.429 |
| CASP3 | SLC2A1 | 9606.ENSP00000311032 | 9606.ENSP00000416293 | 0 | 0 | 0 | 0 | 0 | 0 | 0 | 0.441 | 0.441 |
| CASP3 | ESR2 | 9606.ENSP00000311032 | 9606.ENSP00000343925 | 0 | 0 | 0 | 0 | 0 | 0.058 | 0 | 0.443 | 0.452 |
| CASP3 | PGR | 9606.ENSP00000311032 | 9606.ENSP00000325120 | 0 | 0 | 0 | 0 | 0 | 0.058 | 0 | 0.461 | 0.471 |
| CASP3 | NFKB2 | 9606.ENSP00000311032 | 9606.ENSP00000358983 | 0 | 0 | 0 | 0 | 0 | 0.178 | 0 | 0.431 | 0.512 |
| CASP3 | NOS2 | 9606.ENSP00000311032 | 9606.ENSP00000327251 | 0 | 0 | 0 | 0 | 0 | 0 | 0 | 0.533 | 0.533 |
| CASP3 | RELA | 9606.ENSP00000311032 | 9606.ENSP00000384273 | 0 | 0 | 0 | 0 | 0 | 0 | 0 | 0.557 | 0.557 |
| CASP3 | SP1 | 9606.ENSP00000311032 | 9606.ENSP00000329357 | 0 | 0 | 0 | 0 | 0 | 0.384 | 0 | 0.417 | 0.626 |
| CASP3 | GSK3B | 9606.ENSP00000311032 | 9606.ENSP00000324806 | 0 | 0 | 0 | 0 | 0.059 | 0.097 | 0 | 0.605 | 0.636 |
| CASP3 | ESR1 | 9606.ENSP00000311032 | 9606.ENSP00000405330 | 0 | 0 | 0 | 0 | 0 | 0.058 | 0 | 0.659 | 0.665 |
| CASP3 | IL10 | 9606.ENSP00000311032 | 9606.ENSP00000412237 | 0 | 0 | 0 | 0 | 0 | 0 | 0 | 0.669 | 0.669 |
| CASP3 | HSP90AA1 | 9606.ENSP00000311032 | 9606.ENSP00000335153 | 0 | 0 | 0 | 0 | 0 | 0 | 0 | 0.676 | 0.676 |
| CASP3 | PTGS2 | 9606.ENSP00000311032 | 9606.ENSP00000356438 | 0 | 0 | 0 | 0 | 0 | 0 | 0 | 0.685 | 0.685 |
| CASP3 | IL6 | 9606.ENSP00000311032 | 9606.ENSP00000385675 | 0 | 0 | 0 | 0 | 0 | 0 | 0 | 0.771 | 0.771 |
| CASP3 | INS | 9606.ENSP00000311032 | 9606.ENSP00000380432 | 0 | 0 | 0 | 0 | 0 | 0 | 0 | 0.78 | 0.78 |
| CASP3 | TNF | 9606.ENSP00000311032 | 9606.ENSP00000398698 | 0 | 0 | 0 | 0 | 0 | 0.302 | 0 | 0.825 | 0.873 |
| CCK | GRIA2 | 9606.ENSP00000379472 | 9606.ENSP00000296526 | 0 | 0 | 0 | 0 | 0.35 | 0 | 0 | 0.158 | 0.43 |
| CCK | F2 | 9606.ENSP00000379472 | 9606.ENSP00000308541 | 0 | 0 | 0 | 0 | 0 | 0 | 0.9 | 0.087 | 0.904 |
| CCK | PYY | 9606.ENSP00000379472 | 9606.ENSP00000353198 | 0 | 0 | 0 | 0 | 0.062 | 0 | 0 | 0.908 | 0.91 |
| CCK | DPP4 | 9606.ENSP00000379472 | 9606.ENSP00000353731 | 0 | 0 | 0 | 0 | 0.053 | 0 | 0 | 0.498 | 0.505 |
| CCK | GRIN1 | 9606.ENSP00000379472 | 9606.ENSP00000360608 | 0 | 0 | 0 | 0 | 0.467 | 0 | 0 | 0.139 | 0.522 |
| CCK | GABRD | 9606.ENSP00000379472 | 9606.ENSP00000367848 | 0 | 0 | 0 | 0 | 0.486 | 0 | 0 | 0.052 | 0.492 |
| CCK | IL6 | 9606.ENSP00000379472 | 9606.ENSP00000385675 | 0 | 0 | 0 | 0 | 0 | 0 | 0 | 0.423 | 0.423 |
| CCK | ENSG00000196689 | 9606.ENSP00000379472 | 9606.ENSP00000459962 | 0 | 0 | 0 | 0 | 0 | 0 | 0 | 0.433 | 0.433 |
| CCK | GABRA1 | 9606.ENSP00000379472 | 9606.ENSP00000393097 | 0 | 0 | 0 | 0 | 0.377 | 0 | 0 | 0.182 | 0.469 |
| CCK | GABRG2 | 9606.ENSP00000379472 | 9606.ENSP00000410732 | 0 | 0 | 0 | 0 | 0.384 | 0 | 0 | 0.225 | 0.502 |
| CCK | INS | 9606.ENSP00000379472 | 9606.ENSP00000380432 | 0 | 0 | 0 | 0 | 0 | 0 | 0 | 0.775 | 0.775 |
| CETP | PPARA | 9606.ENSP00000200676 | 9606.ENSP00000385523 | 0 | 0 | 0 | 0 | 0 | 0 | 0 | 0.427 | 0.426 |
| CETP | CRP | 9606.ENSP00000200676 | 9606.ENSP00000255030 | 0 | 0 | 0 | 0 | 0.064 | 0 | 0 | 0.553 | 0.563 |
| CETP | HMGCR | 9606.ENSP00000200676 | 9606.ENSP00000287936 | 0 | 0 | 0 | 0 | 0 | 0 | 0 | 0.589 | 0.589 |
| CETP | INS | 9606.ENSP00000200676 | 9606.ENSP00000380432 | 0 | 0 | 0 | 0 | 0 | 0 | 0 | 0.778 | 0.778 |
| CETP | PON1 | 9606.ENSP00000200676 | 9606.ENSP00000222381 | 0 | 0 | 0 | 0 | 0 | 0 | 0.72 | 0.703 | 0.913 |
| CETP | LPL | 9606.ENSP00000200676 | 9606.ENSP00000309757 | 0 | 0 | 0 | 0 | 0.064 | 0.379 | 0.54 | 0.796 | 0.938 |
| CHRM2 | SLC6A4 | 9606.ENSP00000399745 | 9606.ENSP00000261707 | 0 | 0 | 0 | 0 | 0.061 | 0 | 0 | 0.428 | 0.439 |
| CHRM2 | P2RY12 | 9606.ENSP00000399745 | 9606.ENSP00000307259 | 0 | 0 | 0 | 0 | 0.061 | 0 | 0.9 | 0.21 | 0.919 |
| CHRM2 | PYY | 9606.ENSP00000399745 | 9606.ENSP00000353198 | 0 | 0 | 0 | 0 | 0 | 0 | 0.9 | 0.084 | 0.904 |
| CHRM2 | CHRNA2 | 9606.ENSP00000399745 | 9606.ENSP00000385026 | 0 | 0 | 0 | 0 | 0.063 | 0 | 0 | 0.454 | 0.467 |
| CRP | PON1 | 9606.ENSP00000255030 | 9606.ENSP00000222381 | 0 | 0 | 0 | 0 | 0.098 | 0 | 0 | 0.649 | 0.669 |
| CRP | SERPINE1 | 9606.ENSP00000255030 | 9606.ENSP00000223095 | 0 | 0 | 0 | 0 | 0.049 | 0 | 0 | 0.898 | 0.899 |
| CRP | MPO | 9606.ENSP00000255030 | 9606.ENSP00000225275 | 0 | 0 | 0 | 0 | 0 | 0 | 0 | 0.789 | 0.789 |
| CRP | NFKB1 | 9606.ENSP00000255030 | 9606.ENSP00000226574 | 0 | 0 | 0 | 0 | 0 | 0 | 0 | 0.513 | 0.513 |
| CRP | NR3C1 | 9606.ENSP00000255030 | 9606.ENSP00000231509 | 0 | 0 | 0 | 0 | 0 | 0 | 0 | 0.47 | 0.47 |
| CRP | ESR1 | 9606.ENSP00000255030 | 9606.ENSP00000405330 | 0 | 0 | 0 | 0 | 0.061 | 0.076 | 0 | 0.397 | 0.431 |
| CRP | VDR | 9606.ENSP00000255030 | 9606.ENSP00000447173 | 0 | 0 | 0 | 0 | 0 | 0 | 0 | 0.475 | 0.475 |
| CRP | HSP90AA1 | 9606.ENSP00000255030 | 9606.ENSP00000335153 | 0 | 0 | 0 | 0 | 0 | 0 | 0 | 0.496 | 0.496 |
| CRP | HMGCR | 9606.ENSP00000255030 | 9606.ENSP00000287936 | 0 | 0 | 0 | 0 | 0 | 0 | 0 | 0.498 | 0.497 |
| CRP | PPARG | 9606.ENSP00000255030 | 9606.ENSP00000287820 | 0 | 0 | 0 | 0 | 0 | 0 | 0 | 0.53 | 0.53 |
| CRP | DPP4 | 9606.ENSP00000255030 | 9606.ENSP00000353731 | 0 | 0 | 0 | 0 | 0.061 | 0 | 0 | 0.526 | 0.536 |
| CRP | LPL | 9606.ENSP00000255030 | 9606.ENSP00000309757 | 0 | 0 | 0 | 0 | 0 | 0 | 0 | 0.563 | 0.563 |
| CRP | PTGS2 | 9606.ENSP00000255030 | 9606.ENSP00000356438 | 0 | 0 | 0 | 0 | 0 | 0 | 0 | 0.614 | 0.614 |
| CRP | F7 | 9606.ENSP00000255030 | 9606.ENSP00000364731 | 0 | 0 | 0 | 0 | 0.085 | 0 | 0 | 0.631 | 0.648 |
| CRP | SHBG | 9606.ENSP00000255030 | 9606.ENSP00000369816 | 0 | 0 | 0 | 0 | 0.176 | 0 | 0 | 0.7 | 0.742 |
| CRP | PLG | 9606.ENSP00000255030 | 9606.ENSP00000308938 | 0 | 0 | 0 | 0 | 0.166 | 0 | 0 | 0.737 | 0.771 |
| CRP | F2 | 9606.ENSP00000255030 | 9606.ENSP00000308541 | 0 | 0 | 0 | 0 | 0.15 | 0 | 0 | 0.789 | 0.813 |
| CRP | IL10 | 9606.ENSP00000255030 | 9606.ENSP00000412237 | 0 | 0 | 0 | 0 | 0 | 0 | 0 | 0.873 | 0.873 |
| CRP | TNF | 9606.ENSP00000255030 | 9606.ENSP00000398698 | 0 | 0 | 0 | 0 | 0 | 0 | 0 | 0.899 | 0.899 |
| CRP | INS | 9606.ENSP00000255030 | 9606.ENSP00000380432 | 0 | 0 | 0 | 0 | 0.063 | 0 | 0 | 0.921 | 0.922 |
| CRP | IL6 | 9606.ENSP00000255030 | 9606.ENSP00000385675 | 0 | 0 | 0 | 0 | 0 | 0 | 0 | 0.97 | 0.97 |
| DPP4 | SERPINE1 | 9606.ENSP00000353731 | 9606.ENSP00000223095 | 0 | 0 | 0 | 0 | 0 | 0 | 0 | 0.454 | 0.454 |
| DPP4 | PPARG | 9606.ENSP00000353731 | 9606.ENSP00000287820 | 0 | 0 | 0 | 0 | 0.061 | 0.085 | 0 | 0.476 | 0.51 |
| DPP4 | PYY | 9606.ENSP00000353731 | 9606.ENSP00000353198 | 0 | 0 | 0 | 0 | 0 | 0.255 | 0 | 0.747 | 0.803 |
| DPP4 | IL10 | 9606.ENSP00000353731 | 9606.ENSP00000412237 | 0 | 0 | 0 | 0 | 0 | 0 | 0 | 0.411 | 0.41 |
| DPP4 | TNF | 9606.ENSP00000353731 | 9606.ENSP00000398698 | 0 | 0 | 0 | 0 | 0 | 0 | 0 | 0.473 | 0.473 |
| DPP4 | IL6 | 9606.ENSP00000353731 | 9606.ENSP00000385675 | 0 | 0 | 0 | 0 | 0 | 0 | 0 | 0.514 | 0.514 |
| DPP4 | INS | 9606.ENSP00000353731 | 9606.ENSP00000380432 | 0 | 0 | 0 | 0 | 0 | 0 | 0 | 0.942 | 0.942 |
| ENSG00000196689 | MPO | 9606.ENSP00000459962 | 9606.ENSP00000225275 | 0 | 0 | 0 | 0 | 0 | 0 | 0 | 0.456 | 0.456 |
| ENSG00000196689 | PTGS2 | 9606.ENSP00000459962 | 9606.ENSP00000356438 | 0 | 0 | 0 | 0 | 0 | 0 | 0 | 0.56 | 0.56 |
| ENSG00000196689 | INS | 9606.ENSP00000459962 | 9606.ENSP00000380432 | 0 | 0 | 0 | 0 | 0 | 0 | 0 | 0.69 | 0.69 |
| ENSG00000196689 | PPARA | 9606.ENSP00000459962 | 9606.ENSP00000385523 | 0 | 0 | 0 | 0 | 0 | 0 | 0 | 0.417 | 0.416 |
| ENSG00000196689 | IL6 | 9606.ENSP00000459962 | 9606.ENSP00000385675 | 0 | 0 | 0 | 0 | 0 | 0 | 0 | 0.567 | 0.567 |
| ENSG00000196689 | TNF | 9606.ENSP00000459962 | 9606.ENSP00000398698 | 0 | 0 | 0 | 0 | 0 | 0 | 0 | 0.692 | 0.692 |
| ENSG00000196689 | SCN5A | 9606.ENSP00000459962 | 9606.ENSP00000410257 | 0 | 0 | 0 | 0 | 0 | 0 | 0 | 0.49 | 0.49 |
| ENSG00000196689 | GRIN2B | 9606.ENSP00000459962 | 9606.ENSP00000477455 | 0 | 0 | 0 | 0 | 0 | 0 | 0 | 0.401 | 0.401 |
| ERBB2 | SERPINE1 | 9606.ENSP00000269571 | 9606.ENSP00000223095 | 0 | 0 | 0 | 0 | 0.061 | 0 | 0 | 0.475 | 0.486 |
| ERBB2 | SLC2A1 | 9606.ENSP00000269571 | 9606.ENSP00000416293 | 0 | 0 | 0 | 0 | 0 | 0 | 0 | 0.42 | 0.42 |
| ERBB2 | IL10 | 9606.ENSP00000269571 | 9606.ENSP00000412237 | 0 | 0 | 0 | 0 | 0 | 0 | 0 | 0.447 | 0.447 |
| ERBB2 | ITGB3 | 9606.ENSP00000269571 | 9606.ENSP00000452786 | 0 | 0 | 0 | 0 | 0.051 | 0.261 | 0 | 0.331 | 0.49 |
| ERBB2 | PLG | 9606.ENSP00000269571 | 9606.ENSP00000308938 | 0 | 0 | 0 | 0 | 0.049 | 0 | 0 | 0.491 | 0.495 |
| ERBB2 | PLAU | 9606.ENSP00000269571 | 9606.ENSP00000361850 | 0 | 0 | 0 | 0 | 0.055 | 0 | 0 | 0.491 | 0.498 |
| ERBB2 | TNF | 9606.ENSP00000269571 | 9606.ENSP00000398698 | 0 | 0 | 0 | 0 | 0.062 | 0 | 0 | 0.579 | 0.588 |
| ERBB2 | PPARG | 9606.ENSP00000269571 | 9606.ENSP00000287820 | 0 | 0 | 0 | 0 | 0 | 0.077 | 0 | 0.58 | 0.596 |
| ERBB2 | ESR2 | 9606.ENSP00000269571 | 9606.ENSP00000343925 | 0 | 0 | 0 | 0 | 0 | 0.077 | 0 | 0.659 | 0.671 |
| ERBB2 | PTGS2 | 9606.ENSP00000269571 | 9606.ENSP00000356438 | 0 | 0 | 0 | 0 | 0.061 | 0.305 | 0 | 0.75 | 0.822 |
| ERBB2 | INS | 9606.ENSP00000269571 | 9606.ENSP00000380432 | 0 | 0 | 0 | 0 | 0 | 0 | 0.6 | 0.702 | 0.875 |
| ERBB2 | ESR1 | 9606.ENSP00000269571 | 9606.ENSP00000405330 | 0 | 0 | 0 | 0 | 0 | 0.32 | 0 | 0.926 | 0.947 |
| ERBB2 | PGR | 9606.ENSP00000269571 | 9606.ENSP00000325120 | 0 | 0 | 0 | 0 | 0 | 0.36 | 0 | 0.931 | 0.954 |
| ERBB2 | IL6 | 9606.ENSP00000269571 | 9606.ENSP00000385675 | 0 | 0 | 0 | 0 | 0 | 0 | 0.9 | 0.739 | 0.972 |
| ERBB2 | HSP90AA1 | 9606.ENSP00000269571 | 9606.ENSP00000335153 | 0 | 0 | 0 | 0 | 0 | 0.526 | 0.9 | 0.879 | 0.993 |
| ESR1 | SERPINE1 | 9606.ENSP00000405330 | 9606.ENSP00000223095 | 0 | 0 | 0 | 0 | 0 | 0.051 | 0 | 0.45 | 0.455 |
| ESR1 | NFKB1 | 9606.ENSP00000405330 | 9606.ENSP00000226574 | 0 | 0 | 0 | 0 | 0 | 0.379 | 0 | 0.357 | 0.583 |
| ESR1 | NR3C1 | 9606.ENSP00000405330 | 9606.ENSP00000231509 | 0 | 0 | 0 | 0.66 | 0 | 0 | 0.9 | 0.751 | 0.925 |
| ESR1 | SOD1 | 9606.ENSP00000405330 | 9606.ENSP00000270142 | 0 | 0 | 0 | 0 | 0.061 | 0 | 0 | 0.586 | 0.595 |
| ESR1 | PLG | 9606.ENSP00000405330 | 9606.ENSP00000308938 | 0 | 0 | 0 | 0 | 0.061 | 0 | 0 | 0.44 | 0.451 |
| ESR1 | GSK3B | 9606.ENSP00000405330 | 9606.ENSP00000324806 | 0 | 0 | 0 | 0 | 0 | 0.085 | 0 | 0.38 | 0.409 |
| ESR1 | PGR | 9606.ENSP00000405330 | 9606.ENSP00000325120 | 0 | 0 | 0 | 0.679 | 0.102 | 0.472 | 0.9 | 0.964 | 0.964 |
| ESR1 | SP1 | 9606.ENSP00000405330 | 9606.ENSP00000329357 | 0 | 0 | 0 | 0 | 0.062 | 0.424 | 0.9 | 0.712 | 0.982 |
| ESR1 | HSP90AA1 | 9606.ENSP00000405330 | 9606.ENSP00000335153 | 0 | 0 | 0 | 0 | 0 | 0.474 | 0.9 | 0.633 | 0.979 |
| ESR1 | ESR2 | 9606.ENSP00000405330 | 9606.ENSP00000343925 | 0 | 0 | 0 | 0.925 | 0.061 | 0.379 | 0.9 | 0.967 | 0.941 |
| ESR1 | PTGS2 | 9606.ENSP00000405330 | 9606.ENSP00000356438 | 0 | 0 | 0 | 0 | 0 | 0.05 | 0 | 0.771 | 0.773 |
| ESR1 | PLAU | 9606.ENSP00000405330 | 9606.ENSP00000361850 | 0 | 0 | 0 | 0 | 0.061 | 0 | 0 | 0.44 | 0.451 |
| ESR1 | SHBG | 9606.ENSP00000405330 | 9606.ENSP00000369816 | 0 | 0 | 0 | 0 | 0 | 0 | 0 | 0.683 | 0.683 |
| ESR1 | INS | 9606.ENSP00000405330 | 9606.ENSP00000380432 | 0 | 0 | 0 | 0 | 0 | 0 | 0 | 0.77 | 0.77 |
| ESR1 | RELA | 9606.ENSP00000405330 | 9606.ENSP00000384273 | 0 | 0 | 0 | 0 | 0 | 0.393 | 0 | 0.473 | 0.666 |
| ESR1 | IL6 | 9606.ENSP00000405330 | 9606.ENSP00000385675 | 0 | 0 | 0 | 0 | 0 | 0 | 0 | 0.769 | 0.769 |
| ESR1 | TNF | 9606.ENSP00000405330 | 9606.ENSP00000398698 | 0 | 0 | 0 | 0 | 0 | 0 | 0 | 0.553 | 0.553 |
| ESR1 | IL10 | 9606.ENSP00000405330 | 9606.ENSP00000412237 | 0 | 0 | 0 | 0 | 0 | 0 | 0 | 0.422 | 0.422 |
| ESR1 | HSD17B1 | 9606.ENSP00000405330 | 9606.ENSP00000466799 | 0 | 0 | 0 | 0 | 0 | 0 | 0 | 0.59 | 0.59 |
| ESR2 | SP1 | 9606.ENSP00000343925 | 9606.ENSP00000329357 | 0 | 0 | 0 | 0 | 0 | 0.424 | 0.8 | 0.56 | 0.944 |
| ESR2 | HSP90AA1 | 9606.ENSP00000343925 | 9606.ENSP00000335153 | 0 | 0 | 0 | 0 | 0 | 0.474 | 0.9 | 0.293 | 0.959 |
| ESR2 | IL6 | 9606.ENSP00000343925 | 9606.ENSP00000385675 | 0 | 0 | 0 | 0 | 0 | 0 | 0 | 0.421 | 0.42 |
| ESR2 | PTGS2 | 9606.ENSP00000343925 | 9606.ENSP00000356438 | 0 | 0 | 0 | 0 | 0 | 0.05 | 0 | 0.439 | 0.444 |
| ESR2 | SHBG | 9606.ENSP00000343925 | 9606.ENSP00000369816 | 0 | 0 | 0 | 0 | 0 | 0 | 0 | 0.479 | 0.479 |
| ESR2 | INS | 9606.ENSP00000343925 | 9606.ENSP00000380432 | 0 | 0 | 0 | 0 | 0 | 0 | 0 | 0.51 | 0.51 |
| ESR2 | HSD17B1 | 9606.ENSP00000343925 | 9606.ENSP00000466799 | 0 | 0 | 0 | 0 | 0.062 | 0 | 0 | 0.632 | 0.64 |
| F10 | SERPINE1 | 9606.ENSP00000364709 | 9606.ENSP00000223095 | 0 | 0 | 0 | 0 | 0.065 | 0.129 | 0 | 0.447 | 0.51 |
| F10 | F2 | 9606.ENSP00000364709 | 9606.ENSP00000308541 | 0 | 0 | 0.391 | 0.763 | 0.2 | 0 | 0.9 | 0.806 | 0.938 |
| F10 | PLG | 9606.ENSP00000364709 | 9606.ENSP00000308938 | 0 | 0 | 0 | 0.658 | 0.162 | 0.379 | 0 | 0.582 | 0.562 |
| F10 | F7 | 9606.ENSP00000364709 | 9606.ENSP00000364731 | 0 | 0 | 0.423 | 0.897 | 0.404 | 0.379 | 0.9 | 0.786 | 0.964 |
| F2 | SERPINE1 | 9606.ENSP00000308541 | 9606.ENSP00000223095 | 0 | 0 | 0 | 0 | 0.072 | 0.435 | 0 | 0.736 | 0.849 |
| F2 | MPO | 9606.ENSP00000308541 | 9606.ENSP00000225275 | 0 | 0 | 0 | 0 | 0 | 0.057 | 0 | 0.479 | 0.488 |
| F2 | P2RY12 | 9606.ENSP00000308541 | 9606.ENSP00000307259 | 0 | 0 | 0 | 0 | 0.063 | 0 | 0 | 0.579 | 0.588 |
| F2 | IL10 | 9606.ENSP00000308541 | 9606.ENSP00000412237 | 0 | 0 | 0 | 0 | 0 | 0 | 0 | 0.451 | 0.451 |
| F2 | ITGB3 | 9606.ENSP00000308541 | 9606.ENSP00000452786 | 0 | 0 | 0 | 0 | 0.064 | 0 | 0 | 0.45 | 0.463 |
| F2 | PLG | 9606.ENSP00000308541 | 9606.ENSP00000308938 | 0 | 0 | 0 | 0.727 | 0.309 | 0 | 0 | 0.874 | 0.473 |
| F2 | PTGS2 | 9606.ENSP00000308541 | 9606.ENSP00000356438 | 0 | 0 | 0 | 0 | 0 | 0.057 | 0 | 0.502 | 0.511 |
| F2 | F7 | 9606.ENSP00000308541 | 9606.ENSP00000364731 | 0 | 0 | 0.376 | 0.705 | 0.264 | 0 | 0 | 0.921 | 0.518 |
| F2 | TNF | 9606.ENSP00000308541 | 9606.ENSP00000398698 | 0 | 0 | 0 | 0 | 0 | 0 | 0 | 0.581 | 0.581 |
| F2 | INS | 9606.ENSP00000308541 | 9606.ENSP00000380432 | 0 | 0 | 0 | 0 | 0 | 0 | 0 | 0.597 | 0.597 |
| F2 | IL6 | 9606.ENSP00000308541 | 9606.ENSP00000385675 | 0 | 0 | 0 | 0 | 0 | 0 | 0 | 0.724 | 0.724 |
| F2 | PPARA | 9606.ENSP00000308541 | 9606.ENSP00000385523 | 0 | 0 | 0 | 0 | 0.063 | 0 | 0.9 | 0.106 | 0.908 |
| F2 | PLAU | 9606.ENSP00000308541 | 9606.ENSP00000361850 | 0 | 0 | 0.415 | 0.645 | 0 | 0.379 | 0.9 | 0.434 | 0.952 |
| F7 | SERPINE1 | 9606.ENSP00000364731 | 9606.ENSP00000223095 | 0 | 0 | 0 | 0 | 0 | 0.129 | 0 | 0.788 | 0.807 |
| F7 | PLG | 9606.ENSP00000364731 | 9606.ENSP00000308938 | 0 | 0 | 0 | 0.681 | 0.318 | 0 | 0 | 0.795 | 0.489 |
| F7 | SHBG | 9606.ENSP00000364731 | 9606.ENSP00000369816 | 0 | 0 | 0 | 0 | 0.133 | 0.082 | 0 | 0.355 | 0.441 |
| F7 | IL6 | 9606.ENSP00000364731 | 9606.ENSP00000385675 | 0 | 0 | 0 | 0 | 0 | 0 | 0 | 0.473 | 0.473 |
| F7 | INS | 9606.ENSP00000364731 | 9606.ENSP00000380432 | 0 | 0 | 0 | 0 | 0 | 0 | 0 | 0.531 | 0.531 |
| GABRA1 | SLC6A4 | 9606.ENSP00000393097 | 9606.ENSP00000261707 | 0 | 0 | 0 | 0 | 0.058 | 0 | 0 | 0.398 | 0.409 |
| GABRA1 | GABRA4 | 9606.ENSP00000393097 | 9606.ENSP00000264318 | 0 | 0 | 0 | 0.953 | 0.325 | 0.26 | 0.9 | 0.684 | 0.947 |
| GABRA1 | GRIA2 | 9606.ENSP00000393097 | 9606.ENSP00000296526 | 0 | 0 | 0 | 0 | 0.371 | 0 | 0 | 0.438 | 0.632 |
| GABRA1 | GABRB3 | 9606.ENSP00000393097 | 9606.ENSP00000308725 | 0 | 0 | 0 | 0.844 | 0.172 | 0.743 | 0.9 | 0.769 | 0.979 |
| GABRA1 | GABRA5 | 9606.ENSP00000393097 | 9606.ENSP00000335592 | 0 | 0 | 0 | 0.972 | 0.231 | 0.26 | 0.9 | 0.702 | 0.939 |
| GABRA1 | GABRA3 | 9606.ENSP00000393097 | 9606.ENSP00000359337 | 0 | 0 | 0 | 0.972 | 0.144 | 0.26 | 0.9 | 0.678 | 0.932 |
| GABRA1 | GABRE | 9606.ENSP00000393097 | 9606.ENSP00000359353 | 0 | 0 | 0 | 0.875 | 0 | 0 | 0.72 | 0.536 | 0.738 |
| GABRA1 | GRIN1 | 9606.ENSP00000393097 | 9606.ENSP00000360608 | 0 | 0 | 0 | 0 | 0.366 | 0 | 0 | 0.347 | 0.568 |
| GABRA1 | GABRD | 9606.ENSP00000393097 | 9606.ENSP00000367848 | 0 | 0 | 0 | 0.833 | 0.374 | 0.333 | 0.54 | 0.717 | 0.816 |
| GABRA1 | GRIN2A | 9606.ENSP00000393097 | 9606.ENSP00000379818 | 0 | 0 | 0 | 0 | 0.232 | 0 | 0 | 0.516 | 0.612 |
| GABRA1 | GRIN2B | 9606.ENSP00000393097 | 9606.ENSP00000477455 | 0 | 0 | 0 | 0 | 0.231 | 0 | 0 | 0.479 | 0.582 |
| GABRA1 | GABRP | 9606.ENSP00000393097 | 9606.ENSP00000430100 | 0 | 0 | 0 | 0.819 | 0 | 0.333 | 0.54 | 0.711 | 0.72 |
| GABRA1 | GABRQ | 9606.ENSP00000393097 | 9606.ENSP00000469332 | 0 | 0 | 0 | 0.807 | 0.061 | 0.333 | 0.9 | 0.572 | 0.939 |
| GABRA1 | GABRG2 | 9606.ENSP00000393097 | 9606.ENSP00000410732 | 0 | 0 | 0 | 0.906 | 0.546 | 0.809 | 0.9 | 0.866 | 0.991 |
| GABRA3 | GABRA4 | 9606.ENSP00000359337 | 9606.ENSP00000264318 | 0 | 0 | 0 | 0.949 | 0.098 | 0 | 0.9 | 0.643 | 0.908 |
| GABRA3 | GRIA2 | 9606.ENSP00000359337 | 9606.ENSP00000296526 | 0 | 0 | 0 | 0 | 0.149 | 0 | 0 | 0.565 | 0.614 |
| GABRA3 | GABRB3 | 9606.ENSP00000359337 | 9606.ENSP00000308725 | 0 | 0 | 0 | 0.846 | 0.118 | 0.544 | 0.9 | 0.681 | 0.96 |
| GABRA3 | GABRA5 | 9606.ENSP00000359337 | 9606.ENSP00000335592 | 0 | 0 | 0 | 0.972 | 0.13 | 0.33 | 0.9 | 0.672 | 0.937 |
| GABRA3 | GABRE | 9606.ENSP00000359337 | 9606.ENSP00000359353 | 0 | 0 | 0 | 0.863 | 0 | 0 | 0.54 | 0.747 | 0.586 |
| GABRA3 | GABRP | 9606.ENSP00000359337 | 9606.ENSP00000430100 | 0 | 0 | 0 | 0.833 | 0 | 0.333 | 0.54 | 0.421 | 0.701 |
| GABRA3 | GABRD | 9606.ENSP00000359337 | 9606.ENSP00000367848 | 0 | 0 | 0 | 0.831 | 0.088 | 0.333 | 0.54 | 0.535 | 0.722 |
| GABRA3 | GABRQ | 9606.ENSP00000359337 | 9606.ENSP00000469332 | 0 | 0 | 0 | 0.779 | 0.213 | 0.333 | 0.9 | 0.673 | 0.951 |
| GABRA3 | GABRG2 | 9606.ENSP00000359337 | 9606.ENSP00000410732 | 0 | 0 | 0 | 0.896 | 0.2 | 0.73 | 0.9 | 0.598 | 0.977 |
| GABRA4 | GRIN2A | 9606.ENSP00000264318 | 9606.ENSP00000379818 | 0 | 0 | 0 | 0 | 0.172 | 0 | 0 | 0.358 | 0.446 |
| GABRA4 | GRIA2 | 9606.ENSP00000264318 | 9606.ENSP00000296526 | 0 | 0 | 0 | 0 | 0.292 | 0 | 0 | 0.333 | 0.508 |
| GABRA4 | GABRE | 9606.ENSP00000264318 | 9606.ENSP00000359353 | 0 | 0 | 0 | 0.866 | 0 | 0 | 0.54 | 0.676 | 0.58 |
| GABRA4 | GABRP | 9606.ENSP00000264318 | 9606.ENSP00000430100 | 0 | 0 | 0 | 0.825 | 0 | 0.333 | 0.54 | 0.548 | 0.709 |
| GABRA4 | GABRD | 9606.ENSP00000264318 | 9606.ENSP00000367848 | 0 | 0 | 0 | 0.838 | 0.231 | 0.333 | 0.54 | 0.675 | 0.77 |
| GABRA4 | GABRA5 | 9606.ENSP00000264318 | 9606.ENSP00000335592 | 0 | 0 | 0 | 0.95 | 0.158 | 0.181 | 0.9 | 0.68 | 0.927 |
| GABRA4 | GABRQ | 9606.ENSP00000264318 | 9606.ENSP00000469332 | 0 | 0 | 0 | 0.764 | 0.061 | 0.333 | 0.9 | 0.575 | 0.94 |
| GABRA4 | GABRB3 | 9606.ENSP00000264318 | 9606.ENSP00000308725 | 0 | 0 | 0 | 0.842 | 0.109 | 0.544 | 0.9 | 0.716 | 0.96 |
| GABRA4 | GABRG2 | 9606.ENSP00000264318 | 9606.ENSP00000410732 | 0 | 0 | 0 | 0.883 | 0.308 | 0.73 | 0.9 | 0.698 | 0.981 |
| GABRA5 | GABRB3 | 9606.ENSP00000335592 | 9606.ENSP00000308725 | 0 | 0 | 0 | 0.851 | 0.23 | 0.743 | 0.9 | 0.86 | 0.981 |
| GABRA5 | GRIN2B | 9606.ENSP00000335592 | 9606.ENSP00000477455 | 0 | 0 | 0 | 0 | 0.191 | 0 | 0 | 0.307 | 0.416 |
| GABRA5 | GRIN2A | 9606.ENSP00000335592 | 9606.ENSP00000379818 | 0 | 0 | 0 | 0 | 0.2 | 0 | 0 | 0.407 | 0.505 |
| GABRA5 | GABRE | 9606.ENSP00000335592 | 9606.ENSP00000359353 | 0 | 0 | 0 | 0.874 | 0 | 0 | 0.54 | 0.432 | 0.563 |
| GABRA5 | GABRP | 9606.ENSP00000335592 | 9606.ENSP00000430100 | 0 | 0 | 0 | 0.828 | 0 | 0.333 | 0.54 | 0.471 | 0.704 |
| GABRA5 | GABRD | 9606.ENSP00000335592 | 9606.ENSP00000367848 | 0 | 0 | 0 | 0.843 | 0.233 | 0.333 | 0.54 | 0.721 | 0.772 |
| GABRA5 | GABRQ | 9606.ENSP00000335592 | 9606.ENSP00000469332 | 0 | 0 | 0 | 0.8 | 0.098 | 0.333 | 0.9 | 0.452 | 0.94 |
| GABRA5 | GABRG2 | 9606.ENSP00000335592 | 9606.ENSP00000410732 | 0 | 0 | 0 | 0.898 | 0.334 | 0.799 | 0.9 | 0.684 | 0.986 |
| GABRB3 | SLC6A4 | 9606.ENSP00000308725 | 9606.ENSP00000261707 | 0 | 0 | 0 | 0 | 0 | 0 | 0 | 0.525 | 0.525 |
| GABRB3 | GRIA2 | 9606.ENSP00000308725 | 9606.ENSP00000296526 | 0 | 0 | 0 | 0 | 0.17 | 0 | 0 | 0.46 | 0.533 |
| GABRB3 | GRIN1 | 9606.ENSP00000308725 | 9606.ENSP00000360608 | 0 | 0 | 0 | 0 | 0.118 | 0 | 0 | 0.362 | 0.414 |
| GABRB3 | GRIN2B | 9606.ENSP00000308725 | 9606.ENSP00000477455 | 0 | 0 | 0 | 0 | 0.158 | 0 | 0 | 0.453 | 0.52 |
| GABRB3 | GRIN2A | 9606.ENSP00000308725 | 9606.ENSP00000379818 | 0 | 0 | 0 | 0 | 0.132 | 0 | 0 | 0.474 | 0.524 |
| GABRB3 | GABRP | 9606.ENSP00000308725 | 9606.ENSP00000430100 | 0 | 0 | 0 | 0.892 | 0 | 0 | 0.54 | 0.501 | 0.563 |
| GABRB3 | GABRD | 9606.ENSP00000308725 | 9606.ENSP00000367848 | 0 | 0 | 0 | 0.904 | 0.123 | 0 | 0.54 | 0.672 | 0.606 |
| GABRB3 | GABRE | 9606.ENSP00000308725 | 9606.ENSP00000359353 | 0 | 0 | 0 | 0.823 | 0 | 0.333 | 0.72 | 0.499 | 0.821 |
| GABRB3 | GABRQ | 9606.ENSP00000308725 | 9606.ENSP00000469332 | 0 | 0 | 0 | 0.905 | 0.076 | 0 | 0.9 | 0.5 | 0.908 |
| GABRB3 | GABRG2 | 9606.ENSP00000308725 | 9606.ENSP00000410732 | 0 | 0 | 0 | 0.834 | 0.174 | 0.798 | 0.9 | 0.754 | 0.984 |
| GABRD | GRIA2 | 9606.ENSP00000367848 | 9606.ENSP00000296526 | 0 | 0 | 0 | 0 | 0.352 | 0 | 0 | 0.308 | 0.533 |
| GABRD | GABRE | 9606.ENSP00000367848 | 9606.ENSP00000359353 | 0 | 0 | 0 | 0.834 | 0 | 0.333 | 0.54 | 0.647 | 0.713 |
| GABRD | GRIN1 | 9606.ENSP00000367848 | 9606.ENSP00000360608 | 0 | 0 | 0 | 0 | 0.537 | 0 | 0 | 0.166 | 0.597 |
| GABRD | GABRP | 9606.ENSP00000367848 | 9606.ENSP00000430100 | 0 | 0 | 0 | 0.901 | 0 | 0 | 0.54 | 0.601 | 0.566 |
| GABRD | GABRQ | 9606.ENSP00000367848 | 9606.ENSP00000469332 | 0 | 0 | 0 | 0.86 | 0.067 | 0 | 0.54 | 0.649 | 0.592 |
| GABRD | GABRG2 | 9606.ENSP00000367848 | 9606.ENSP00000410732 | 0 | 0 | 0 | 0.849 | 0.425 | 0.333 | 0.54 | 0.733 | 0.829 |
| GABRE | GABRP | 9606.ENSP00000359353 | 9606.ENSP00000430100 | 0 | 0 | 0 | 0.796 | 0 | 0.333 | 0.54 | 0.667 | 0.722 |
| GABRE | GABRG2 | 9606.ENSP00000359353 | 9606.ENSP00000410732 | 0 | 0 | 0 | 0.902 | 0 | 0 | 0.72 | 0.518 | 0.733 |
| GABRE | GABRQ | 9606.ENSP00000359353 | 9606.ENSP00000469332 | 0 | 0 | 0 | 0.778 | 0.077 | 0.333 | 0.54 | 0.801 | 0.746 |
| GABRG2 | GRIA2 | 9606.ENSP00000410732 | 9606.ENSP00000296526 | 0 | 0 | 0 | 0 | 0.463 | 0 | 0 | 0.438 | 0.685 |
| GABRG2 | GRIN1 | 9606.ENSP00000410732 | 9606.ENSP00000360608 | 0 | 0 | 0 | 0 | 0.447 | 0 | 0 | 0.589 | 0.763 |
| GABRG2 | GRIN2A | 9606.ENSP00000410732 | 9606.ENSP00000379818 | 0 | 0 | 0 | 0 | 0.247 | 0 | 0 | 0.41 | 0.537 |
| GABRG2 | GRIN2B | 9606.ENSP00000410732 | 9606.ENSP00000477455 | 0 | 0 | 0 | 0 | 0.232 | 0 | 0 | 0.41 | 0.527 |
| GABRG2 | GABRP | 9606.ENSP00000410732 | 9606.ENSP00000430100 | 0 | 0 | 0 | 0.824 | 0 | 0.333 | 0.54 | 0.514 | 0.707 |
| GABRG2 | GABRQ | 9606.ENSP00000410732 | 9606.ENSP00000469332 | 0 | 0 | 0 | 0.76 | 0.086 | 0.333 | 0.9 | 0.492 | 0.941 |
| GABRP | SLC6A3 | 9606.ENSP00000430100 | 9606.ENSP00000270349 | 0 | 0 | 0 | 0 | 0 | 0 | 0 | 0.468 | 0.468 |
| GABRP | GABRQ | 9606.ENSP00000430100 | 9606.ENSP00000469332 | 0 | 0 | 0 | 0.836 | 0 | 0 | 0.54 | 0.586 | 0.582 |
| GRIA2 | GRIN2D | 9606.ENSP00000296526 | 9606.ENSP00000263269 | 0 | 0 | 0 | 0.601 | 0 | 0.143 | 0.9 | 0.618 | 0.932 |
| GRIA2 | GRIN2A | 9606.ENSP00000296526 | 9606.ENSP00000379818 | 0 | 0 | 0 | 0.599 | 0.232 | 0.143 | 0.9 | 0.824 | 0.951 |
| GRIA2 | GRIN2B | 9606.ENSP00000296526 | 9606.ENSP00000477455 | 0 | 0 | 0 | 0.609 | 0.284 | 0.143 | 0.9 | 0.891 | 0.956 |
| GRIA2 | GRIN1 | 9606.ENSP00000296526 | 9606.ENSP00000360608 | 0 | 0 | 0 | 0.657 | 0.377 | 0.269 | 0.9 | 0.831 | 0.964 |
| GRIN1 | GRIN2D | 9606.ENSP00000360608 | 9606.ENSP00000263269 | 0 | 0 | 0 | 0.661 | 0.169 | 0.619 | 0.9 | 0.876 | 0.975 |
| GRIN1 | GRIN2A | 9606.ENSP00000360608 | 9606.ENSP00000379818 | 0 | 0 | 0 | 0.687 | 0.266 | 0.826 | 0.9 | 0.96 | 0.99 |
| GRIN1 | GRIN2B | 9606.ENSP00000360608 | 9606.ENSP00000477455 | 0 | 0 | 0 | 0.697 | 0.267 | 0.851 | 0.9 | 0.935 | 0.991 |
| GRIN2A | GRIN2D | 9606.ENSP00000379818 | 9606.ENSP00000263269 | 0 | 0 | 0 | 0.904 | 0.061 | 0 | 0.9 | 0.922 | 0.91 |
| GRIN2A | SLC6A3 | 9606.ENSP00000379818 | 9606.ENSP00000270349 | 0 | 0 | 0 | 0 | 0 | 0 | 0 | 0.414 | 0.414 |
| GRIN2A | GRIN2B | 9606.ENSP00000379818 | 9606.ENSP00000477455 | 0 | 0 | 0 | 0.945 | 0.175 | 0.268 | 0.9 | 0.961 | 0.937 |
| GRIN2B | NR3C1 | 9606.ENSP00000477455 | 9606.ENSP00000231509 | 0 | 0 | 0 | 0 | 0 | 0 | 0 | 0.4 | 0.4 |
| GRIN2B | SLC6A4 | 9606.ENSP00000477455 | 9606.ENSP00000261707 | 0 | 0 | 0 | 0 | 0 | 0 | 0 | 0.474 | 0.474 |
| GRIN2B | GRIN2D | 9606.ENSP00000477455 | 9606.ENSP00000263269 | 0 | 0 | 0 | 0.897 | 0.061 | 0 | 0.9 | 0.921 | 0.911 |
| GRIN2B | SLC6A3 | 9606.ENSP00000477455 | 9606.ENSP00000270349 | 0 | 0 | 0 | 0 | 0 | 0 | 0 | 0.474 | 0.474 |
| GRIN2B | GSK3B | 9606.ENSP00000477455 | 9606.ENSP00000324806 | 0 | 0 | 0 | 0 | 0.062 | 0.159 | 0 | 0.328 | 0.423 |
| GSK3B | NFKB1 | 9606.ENSP00000324806 | 9606.ENSP00000226574 | 0 | 0 | 0 | 0 | 0.07 | 0.433 | 0 | 0.208 | 0.547 |
| GSK3B | NR3C1 | 9606.ENSP00000324806 | 9606.ENSP00000231509 | 0 | 0 | 0 | 0 | 0 | 0.085 | 0.9 | 0.432 | 0.943 |
| GSK3B | SOD1 | 9606.ENSP00000324806 | 9606.ENSP00000270142 | 0 | 0 | 0 | 0 | 0 | 0.085 | 0 | 0.508 | 0.53 |
| GSK3B | PPARG | 9606.ENSP00000324806 | 9606.ENSP00000287820 | 0 | 0 | 0 | 0 | 0 | 0.085 | 0 | 0.558 | 0.578 |
| GSK3B | RELA | 9606.ENSP00000324806 | 9606.ENSP00000384273 | 0 | 0 | 0 | 0 | 0 | 0.294 | 0 | 0.244 | 0.443 |
| GSK3B | IL6 | 9606.ENSP00000324806 | 9606.ENSP00000385675 | 0 | 0 | 0 | 0 | 0 | 0 | 0 | 0.573 | 0.573 |
| GSK3B | TNF | 9606.ENSP00000324806 | 9606.ENSP00000398698 | 0 | 0 | 0 | 0 | 0 | 0.05 | 0 | 0.596 | 0.6 |
| GSK3B | PTGS2 | 9606.ENSP00000324806 | 9606.ENSP00000356438 | 0 | 0 | 0 | 0 | 0 | 0.058 | 0 | 0.63 | 0.637 |
| GSK3B | HSP90AA1 | 9606.ENSP00000324806 | 9606.ENSP00000335153 | 0 | 0 | 0 | 0 | 0 | 0.442 | 0 | 0.44 | 0.674 |
| GSK3B | INS | 9606.ENSP00000324806 | 9606.ENSP00000380432 | 0 | 0 | 0 | 0 | 0 | 0.262 | 0 | 0.6 | 0.692 |
| HMGCR | PON1 | 9606.ENSP00000287936 | 9606.ENSP00000222381 | 0 | 0 | 0 | 0 | 0 | 0 | 0 | 0.425 | 0.425 |
| HMGCR | PTGIS | 9606.ENSP00000287936 | 9606.ENSP00000244043 | 0 | 0 | 0 | 0 | 0.127 | 0 | 0 | 0.347 | 0.406 |
| HMGCR | PPARG | 9606.ENSP00000287936 | 9606.ENSP00000287820 | 0 | 0 | 0 | 0 | 0.061 | 0 | 0 | 0.485 | 0.496 |
| HMGCR | IL6 | 9606.ENSP00000287936 | 9606.ENSP00000385675 | 0 | 0 | 0 | 0 | 0 | 0 | 0 | 0.43 | 0.43 |
| HMGCR | NOS2 | 9606.ENSP00000287936 | 9606.ENSP00000327251 | 0 | 0 | 0 | 0 | 0.049 | 0 | 0 | 0.5 | 0.505 |
| HMGCR | PPARA | 9606.ENSP00000287936 | 9606.ENSP00000385523 | 0 | 0 | 0 | 0 | 0.061 | 0 | 0 | 0.562 | 0.571 |
| HMGCR | LPL | 9606.ENSP00000287936 | 9606.ENSP00000309757 | 0 | 0 | 0 | 0 | 0 | 0 | 0 | 0.585 | 0.585 |
| HMGCR | INS | 9606.ENSP00000287936 | 9606.ENSP00000380432 | 0 | 0 | 0 | 0 | 0 | 0 | 0 | 0.641 | 0.641 |
| HSD11B1 | NR3C1 | 9606.ENSP00000355995 | 9606.ENSP00000231509 | 0 | 0 | 0 | 0 | 0.061 | 0 | 0 | 0.749 | 0.754 |
| HSD11B1 | PPARG | 9606.ENSP00000355995 | 9606.ENSP00000287820 | 0 | 0 | 0 | 0 | 0.061 | 0 | 0.9 | 0.462 | 0.945 |
| HSD11B1 | INS | 9606.ENSP00000355995 | 9606.ENSP00000380432 | 0 | 0 | 0 | 0 | 0 | 0 | 0 | 0.631 | 0.631 |
| HSD17B1 | PGR | 9606.ENSP00000466799 | 9606.ENSP00000325120 | 0 | 0 | 0 | 0 | 0 | 0 | 0 | 0.49 | 0.489 |
| HSD17B1 | SHBG | 9606.ENSP00000466799 | 9606.ENSP00000369816 | 0 | 0 | 0 | 0 | 0 | 0 | 0 | 0.42 | 0.42 |
| HSP90AA1 | NFKB1 | 9606.ENSP00000335153 | 9606.ENSP00000226574 | 0 | 0 | 0 | 0 | 0.11 | 0.097 | 0 | 0.444 | 0.514 |
| HSP90AA1 | NR3C1 | 9606.ENSP00000335153 | 9606.ENSP00000231509 | 0 | 0 | 0 | 0 | 0 | 0.474 | 0.9 | 0.71 | 0.983 |
| HSP90AA1 | KCNH2 | 9606.ENSP00000335153 | 9606.ENSP00000262186 | 0 | 0 | 0 | 0 | 0 | 0.379 | 0 | 0.639 | 0.766 |
| HSP90AA1 | SOD1 | 9606.ENSP00000335153 | 9606.ENSP00000270142 | 0 | 0 | 0 | 0 | 0.234 | 0 | 0 | 0.657 | 0.726 |
| HSP90AA1 | PPARD | 9606.ENSP00000335153 | 9606.ENSP00000310928 | 0 | 0 | 0 | 0 | 0 | 0.474 | 0 | 0.122 | 0.518 |
| HSP90AA1 | PGR | 9606.ENSP00000335153 | 9606.ENSP00000325120 | 0 | 0 | 0 | 0 | 0 | 0.523 | 0.9 | 0.72 | 0.985 |
| HSP90AA1 | NOS2 | 9606.ENSP00000335153 | 9606.ENSP00000327251 | 0 | 0 | 0 | 0 | 0.06 | 0.379 | 0 | 0.54 | 0.708 |
| HSP90AA1 | SP1 | 9606.ENSP00000335153 | 9606.ENSP00000329357 | 0 | 0 | 0 | 0 | 0.082 | 0.407 | 0 | 0.554 | 0.736 |
| HSP90AA1 | IL10 | 9606.ENSP00000335153 | 9606.ENSP00000412237 | 0 | 0 | 0 | 0 | 0 | 0 | 0 | 0.401 | 0.4 |
| HSP90AA1 | PTGS2 | 9606.ENSP00000335153 | 9606.ENSP00000356438 | 0 | 0 | 0 | 0 | 0 | 0 | 0 | 0.409 | 0.409 |
| HSP90AA1 | RELA | 9606.ENSP00000335153 | 9606.ENSP00000384273 | 0 | 0 | 0 | 0 | 0.049 | 0.101 | 0 | 0.421 | 0.461 |
| HSP90AA1 | NFKB2 | 9606.ENSP00000335153 | 9606.ENSP00000358983 | 0 | 0 | 0 | 0 | 0 | 0.153 | 0 | 0.414 | 0.482 |
| HSP90AA1 | TNF | 9606.ENSP00000335153 | 9606.ENSP00000398698 | 0 | 0 | 0 | 0 | 0 | 0 | 0 | 0.526 | 0.526 |
| HSP90AA1 | INS | 9606.ENSP00000335153 | 9606.ENSP00000380432 | 0 | 0 | 0 | 0 | 0 | 0 | 0 | 0.552 | 0.552 |
| HSP90AA1 | IL6 | 9606.ENSP00000335153 | 9606.ENSP00000385675 | 0 | 0 | 0 | 0 | 0 | 0 | 0 | 0.709 | 0.709 |
| HSP90AA1 | ITGB3 | 9606.ENSP00000335153 | 9606.ENSP00000452786 | 0 | 0 | 0 | 0 | 0 | 0 | 0.9 | 0.114 | 0.907 |
| HSP90AA1 | PPARA | 9606.ENSP00000335153 | 9606.ENSP00000385523 | 0 | 0 | 0 | 0 | 0 | 0.474 | 0.9 | 0.239 | 0.956 |
| IL10 | SERPINE1 | 9606.ENSP00000412237 | 9606.ENSP00000223095 | 0 | 0 | 0 | 0 | 0 | 0 | 0 | 0.578 | 0.578 |
| IL10 | MPO | 9606.ENSP00000412237 | 9606.ENSP00000225275 | 0 | 0 | 0 | 0 | 0 | 0 | 0 | 0.772 | 0.772 |
| IL10 | NFKB1 | 9606.ENSP00000412237 | 9606.ENSP00000226574 | 0 | 0 | 0 | 0 | 0 | 0 | 0 | 0.502 | 0.502 |
| IL10 | NR3C1 | 9606.ENSP00000412237 | 9606.ENSP00000231509 | 0 | 0 | 0 | 0 | 0 | 0.328 | 0 | 0.671 | 0.77 |
| IL10 | SOD1 | 9606.ENSP00000412237 | 9606.ENSP00000270142 | 0 | 0 | 0 | 0 | 0 | 0 | 0 | 0.433 | 0.433 |
| IL10 | PPARG | 9606.ENSP00000412237 | 9606.ENSP00000287820 | 0 | 0 | 0 | 0 | 0 | 0 | 0 | 0.556 | 0.556 |
| IL10 | PLG | 9606.ENSP00000412237 | 9606.ENSP00000308938 | 0 | 0 | 0 | 0 | 0 | 0 | 0 | 0.555 | 0.555 |
| IL10 | NOS2 | 9606.ENSP00000412237 | 9606.ENSP00000327251 | 0 | 0 | 0 | 0 | 0 | 0 | 0 | 0.696 | 0.696 |
| IL10 | PTGS1 | 9606.ENSP00000412237 | 9606.ENSP00000354612 | 0 | 0 | 0 | 0 | 0.05 | 0 | 0 | 0.419 | 0.425 |
| IL10 | PTGS2 | 9606.ENSP00000412237 | 9606.ENSP00000356438 | 0 | 0 | 0 | 0 | 0.061 | 0 | 0 | 0.831 | 0.836 |
| IL10 | NFKB2 | 9606.ENSP00000412237 | 9606.ENSP00000358983 | 0 | 0 | 0 | 0 | 0.06 | 0 | 0 | 0.457 | 0.468 |
| IL10 | INS | 9606.ENSP00000412237 | 9606.ENSP00000380432 | 0 | 0 | 0 | 0 | 0.062 | 0 | 0 | 0.731 | 0.737 |
| IL10 | RELA | 9606.ENSP00000412237 | 9606.ENSP00000384273 | 0 | 0 | 0 | 0 | 0 | 0 | 0 | 0.547 | 0.547 |
| IL10 | IL6 | 9606.ENSP00000412237 | 9606.ENSP00000385675 | 0 | 0 | 0 | 0 | 0.077 | 0 | 0.9 | 0.974 | 0.997 |
| IL10 | TNF | 9606.ENSP00000412237 | 9606.ENSP00000398698 | 0 | 0 | 0 | 0 | 0.097 | 0 | 0.9 | 0.974 | 0.997 |
| IL10 | RBP3 | 9606.ENSP00000412237 | 9606.ENSP00000463151 | 0 | 0 | 0 | 0 | 0 | 0 | 0 | 0.473 | 0.473 |
| IL10 | VDR | 9606.ENSP00000412237 | 9606.ENSP00000447173 | 0 | 0 | 0 | 0 | 0 | 0 | 0 | 0.557 | 0.557 |
| IL6 | PON1 | 9606.ENSP00000385675 | 9606.ENSP00000222381 | 0 | 0 | 0 | 0 | 0 | 0 | 0 | 0.708 | 0.708 |
| IL6 | SERPINE1 | 9606.ENSP00000385675 | 9606.ENSP00000223095 | 0 | 0 | 0 | 0 | 0.159 | 0 | 0 | 0.789 | 0.815 |
| IL6 | MPO | 9606.ENSP00000385675 | 9606.ENSP00000225275 | 0 | 0 | 0 | 0 | 0.061 | 0 | 0 | 0.848 | 0.852 |
| IL6 | NFKB1 | 9606.ENSP00000385675 | 9606.ENSP00000226574 | 0 | 0 | 0 | 0 | 0.086 | 0 | 0.9 | 0.632 | 0.963 |
| IL6 | NR3C1 | 9606.ENSP00000385675 | 9606.ENSP00000231509 | 0 | 0 | 0 | 0 | 0 | 0 | 0 | 0.646 | 0.646 |
| IL6 | SLC6A4 | 9606.ENSP00000385675 | 9606.ENSP00000261707 | 0 | 0 | 0 | 0 | 0 | 0 | 0 | 0.419 | 0.418 |
| IL6 | SOD1 | 9606.ENSP00000385675 | 9606.ENSP00000270142 | 0 | 0 | 0 | 0 | 0 | 0 | 0 | 0.541 | 0.541 |
| IL6 | PPARG | 9606.ENSP00000385675 | 9606.ENSP00000287820 | 0 | 0 | 0 | 0 | 0 | 0 | 0 | 0.812 | 0.812 |
| IL6 | P2RY12 | 9606.ENSP00000385675 | 9606.ENSP00000307259 | 0 | 0 | 0 | 0 | 0 | 0 | 0 | 0.402 | 0.402 |
| IL6 | PLG | 9606.ENSP00000385675 | 9606.ENSP00000308938 | 0 | 0 | 0 | 0 | 0 | 0 | 0 | 0.704 | 0.704 |
| IL6 | LPL | 9606.ENSP00000385675 | 9606.ENSP00000309757 | 0 | 0 | 0 | 0 | 0 | 0 | 0 | 0.59 | 0.59 |
| IL6 | PGR | 9606.ENSP00000385675 | 9606.ENSP00000325120 | 0 | 0 | 0 | 0 | 0.062 | 0 | 0 | 0.43 | 0.442 |
| IL6 | NOS2 | 9606.ENSP00000385675 | 9606.ENSP00000327251 | 0 | 0 | 0 | 0 | 0.063 | 0 | 0 | 0.735 | 0.741 |
| IL6 | PYY | 9606.ENSP00000385675 | 9606.ENSP00000353198 | 0 | 0 | 0 | 0 | 0 | 0 | 0 | 0.41 | 0.41 |
| IL6 | PTGS1 | 9606.ENSP00000385675 | 9606.ENSP00000354612 | 0 | 0 | 0 | 0 | 0.094 | 0 | 0 | 0.53 | 0.556 |
| IL6 | PTGS2 | 9606.ENSP00000385675 | 9606.ENSP00000356438 | 0 | 0 | 0 | 0 | 0.314 | 0 | 0 | 0.922 | 0.945 |
| IL6 | NFKB2 | 9606.ENSP00000385675 | 9606.ENSP00000358983 | 0 | 0 | 0 | 0 | 0.096 | 0 | 0 | 0.593 | 0.617 |
| IL6 | PLAU | 9606.ENSP00000385675 | 9606.ENSP00000361850 | 0 | 0 | 0 | 0 | 0.134 | 0 | 0 | 0.496 | 0.544 |
| IL6 | SHBG | 9606.ENSP00000385675 | 9606.ENSP00000369816 | 0 | 0 | 0 | 0 | 0 | 0 | 0 | 0.474 | 0.474 |
| IL6 | INS | 9606.ENSP00000385675 | 9606.ENSP00000380432 | 0 | 0 | 0 | 0 | 0 | 0 | 0 | 0.947 | 0.947 |
| IL6 | RELA | 9606.ENSP00000385675 | 9606.ENSP00000384273 | 0 | 0 | 0 | 0 | 0 | 0.223 | 0.9 | 0.804 | 0.983 |
| IL6 | PPARA | 9606.ENSP00000385675 | 9606.ENSP00000385523 | 0 | 0 | 0 | 0 | 0 | 0 | 0 | 0.517 | 0.517 |
| IL6 | ITGB3 | 9606.ENSP00000385675 | 9606.ENSP00000452786 | 0 | 0 | 0 | 0 | 0.111 | 0 | 0 | 0.42 | 0.462 |
| IL6 | SLC2A1 | 9606.ENSP00000385675 | 9606.ENSP00000416293 | 0 | 0 | 0 | 0 | 0 | 0 | 0 | 0.472 | 0.472 |
| IL6 | VDR | 9606.ENSP00000385675 | 9606.ENSP00000447173 | 0 | 0 | 0 | 0 | 0 | 0 | 0 | 0.576 | 0.576 |
| IL6 | TNF | 9606.ENSP00000385675 | 9606.ENSP00000398698 | 0 | 0 | 0 | 0 | 0.123 | 0 | 0 | 0.982 | 0.984 |
| INS | PON1 | 9606.ENSP00000380432 | 9606.ENSP00000222381 | 0 | 0 | 0 | 0 | 0 | 0 | 0 | 0.559 | 0.559 |
| INS | SERPINE1 | 9606.ENSP00000380432 | 9606.ENSP00000223095 | 0 | 0 | 0 | 0 | 0 | 0 | 0 | 0.916 | 0.916 |
| INS | MPO | 9606.ENSP00000380432 | 9606.ENSP00000225275 | 0 | 0 | 0 | 0 | 0 | 0 | 0 | 0.723 | 0.723 |
| INS | NR3C1 | 9606.ENSP00000380432 | 9606.ENSP00000231509 | 0 | 0 | 0 | 0 | 0 | 0 | 0 | 0.673 | 0.673 |
| INS | SOD1 | 9606.ENSP00000380432 | 9606.ENSP00000270142 | 0 | 0 | 0 | 0 | 0 | 0 | 0 | 0.542 | 0.542 |
| INS | SLC6A3 | 9606.ENSP00000380432 | 9606.ENSP00000270349 | 0 | 0 | 0 | 0 | 0 | 0 | 0 | 0.408 | 0.408 |
| INS | PPARG | 9606.ENSP00000380432 | 9606.ENSP00000287820 | 0 | 0 | 0 | 0 | 0 | 0 | 0 | 0.944 | 0.945 |
| INS | PLG | 9606.ENSP00000380432 | 9606.ENSP00000308938 | 0 | 0 | 0 | 0 | 0 | 0 | 0 | 0.831 | 0.831 |
| INS | LPL | 9606.ENSP00000380432 | 9606.ENSP00000309757 | 0 | 0 | 0 | 0 | 0 | 0 | 0 | 0.866 | 0.866 |
| INS | PPARD | 9606.ENSP00000380432 | 9606.ENSP00000310928 | 0 | 0 | 0 | 0 | 0 | 0 | 0 | 0.492 | 0.491 |
| INS | PGR | 9606.ENSP00000380432 | 9606.ENSP00000325120 | 0 | 0 | 0 | 0 | 0 | 0 | 0 | 0.598 | 0.598 |
| INS | NOS2 | 9606.ENSP00000380432 | 9606.ENSP00000327251 | 0 | 0 | 0 | 0 | 0 | 0 | 0 | 0.511 | 0.511 |
| INS | SP1 | 9606.ENSP00000380432 | 9606.ENSP00000329357 | 0 | 0 | 0 | 0 | 0 | 0 | 0 | 0.656 | 0.656 |
| INS | PYY | 9606.ENSP00000380432 | 9606.ENSP00000353198 | 0 | 0 | 0 | 0 | 0.062 | 0 | 0 | 0.807 | 0.811 |
| INS | PRSS3 | 9606.ENSP00000380432 | 9606.ENSP00000354280 | 0 | 0 | 0 | 0 | 0.064 | 0 | 0 | 0.56 | 0.571 |
| INS | PTGS2 | 9606.ENSP00000380432 | 9606.ENSP00000356438 | 0 | 0 | 0 | 0 | 0 | 0 | 0 | 0.651 | 0.651 |
| INS | PLAU | 9606.ENSP00000380432 | 9606.ENSP00000361850 | 0 | 0 | 0 | 0 | 0 | 0 | 0 | 0.417 | 0.416 |
| INS | SHBG | 9606.ENSP00000380432 | 9606.ENSP00000369816 | 0 | 0 | 0 | 0 | 0 | 0 | 0 | 0.869 | 0.869 |
| INS | RELA | 9606.ENSP00000380432 | 9606.ENSP00000384273 | 0 | 0 | 0 | 0 | 0 | 0 | 0 | 0.444 | 0.444 |
| INS | VDR | 9606.ENSP00000380432 | 9606.ENSP00000447173 | 0 | 0 | 0 | 0 | 0 | 0 | 0 | 0.653 | 0.653 |
| INS | PPARA | 9606.ENSP00000380432 | 9606.ENSP00000385523 | 0 | 0 | 0 | 0 | 0 | 0 | 0 | 0.733 | 0.733 |
| INS | SLC2A1 | 9606.ENSP00000380432 | 9606.ENSP00000416293 | 0 | 0 | 0 | 0 | 0 | 0 | 0 | 0.873 | 0.873 |
| INS | TNF | 9606.ENSP00000380432 | 9606.ENSP00000398698 | 0 | 0 | 0 | 0 | 0 | 0 | 0 | 0.916 | 0.916 |
| ITGB3 | SERPINE1 | 9606.ENSP00000452786 | 9606.ENSP00000223095 | 0 | 0 | 0 | 0 | 0.126 | 0 | 0.9 | 0.448 | 0.947 |
| ITGB3 | MPO | 9606.ENSP00000452786 | 9606.ENSP00000225275 | 0 | 0 | 0 | 0 | 0.062 | 0.097 | 0 | 0.497 | 0.536 |
| ITGB3 | P2RY12 | 9606.ENSP00000452786 | 9606.ENSP00000307259 | 0 | 0 | 0 | 0 | 0.062 | 0 | 0 | 0.539 | 0.549 |
| ITGB3 | PTGS1 | 9606.ENSP00000452786 | 9606.ENSP00000354612 | 0 | 0 | 0 | 0 | 0.178 | 0.097 | 0 | 0.403 | 0.518 |
| ITGB3 | PLAU | 9606.ENSP00000452786 | 9606.ENSP00000361850 | 0 | 0 | 0 | 0 | 0.095 | 0 | 0.9 | 0.282 | 0.929 |
| KCNH2 | SCN5A | 9606.ENSP00000262186 | 9606.ENSP00000410257 | 0 | 0 | 0 | 0 | 0.063 | 0.094 | 0 | 0.927 | 0.933 |
| LPL | PON1 | 9606.ENSP00000309757 | 9606.ENSP00000222381 | 0 | 0 | 0 | 0 | 0 | 0 | 0.54 | 0.479 | 0.75 |
| LPL | SERPINE1 | 9606.ENSP00000309757 | 9606.ENSP00000223095 | 0 | 0 | 0 | 0 | 0 | 0 | 0 | 0.475 | 0.475 |
| LPL | NR3C1 | 9606.ENSP00000309757 | 9606.ENSP00000231509 | 0 | 0 | 0 | 0 | 0 | 0 | 0.9 | 0.408 | 0.938 |
| LPL | PPARG | 9606.ENSP00000309757 | 9606.ENSP00000287820 | 0 | 0 | 0 | 0 | 0.062 | 0 | 0.9 | 0.808 | 0.98 |
| LPL | PPARD | 9606.ENSP00000309757 | 9606.ENSP00000310928 | 0 | 0 | 0 | 0 | 0 | 0 | 0 | 0.426 | 0.426 |
| LPL | SLC2A1 | 9606.ENSP00000309757 | 9606.ENSP00000416293 | 0 | 0 | 0 | 0 | 0 | 0 | 0 | 0.44 | 0.44 |
| LPL | PYY | 9606.ENSP00000309757 | 9606.ENSP00000353198 | 0 | 0 | 0 | 0 | 0 | 0 | 0 | 0.444 | 0.444 |
| LPL | TNF | 9606.ENSP00000309757 | 9606.ENSP00000398698 | 0 | 0 | 0 | 0 | 0.061 | 0 | 0 | 0.619 | 0.627 |
| LPL | SP1 | 9606.ENSP00000309757 | 9606.ENSP00000329357 | 0 | 0 | 0 | 0 | 0 | 0 | 0.9 | 0.112 | 0.907 |
| LPL | RELA | 9606.ENSP00000309757 | 9606.ENSP00000384273 | 0 | 0 | 0 | 0 | 0 | 0 | 0.9 | 0.209 | 0.917 |
| LPL | PPARA | 9606.ENSP00000309757 | 9606.ENSP00000385523 | 0 | 0 | 0 | 0 | 0.061 | 0 | 0.9 | 0.673 | 0.966 |
| MAOB | SLC6A2 | 9606.ENSP00000367309 | 9606.ENSP00000219833 | 0 | 0 | 0 | 0 | 0 | 0 | 0 | 0.494 | 0.494 |
| MAOB | SLC6A4 | 9606.ENSP00000367309 | 9606.ENSP00000261707 | 0 | 0 | 0 | 0 | 0.062 | 0 | 0 | 0.67 | 0.677 |
| MAOB | SLC6A3 | 9606.ENSP00000367309 | 9606.ENSP00000270349 | 0 | 0 | 0 | 0 | 0 | 0 | 0 | 0.733 | 0.733 |
| MPO | PON1 | 9606.ENSP00000225275 | 9606.ENSP00000222381 | 0 | 0 | 0 | 0 | 0 | 0 | 0 | 0.575 | 0.575 |
| MPO | SERPINE1 | 9606.ENSP00000225275 | 9606.ENSP00000223095 | 0 | 0 | 0 | 0 | 0 | 0 | 0 | 0.51 | 0.51 |
| MPO | SOD1 | 9606.ENSP00000225275 | 9606.ENSP00000270142 | 0 | 0 | 0 | 0 | 0.061 | 0 | 0 | 0.417 | 0.429 |
| MPO | PPARG | 9606.ENSP00000225275 | 9606.ENSP00000287820 | 0 | 0 | 0 | 0 | 0.061 | 0.05 | 0 | 0.452 | 0.468 |
| MPO | PLG | 9606.ENSP00000225275 | 9606.ENSP00000308938 | 0 | 0 | 0 | 0 | 0 | 0.057 | 0 | 0.502 | 0.51 |
| MPO | NOS2 | 9606.ENSP00000225275 | 9606.ENSP00000327251 | 0 | 0 | 0 | 0 | 0 | 0.157 | 0 | 0.595 | 0.644 |
| MPO | PTGS2 | 9606.ENSP00000225275 | 9606.ENSP00000356438 | 0 | 0 | 0 | 0 | 0.065 | 0 | 0 | 0.691 | 0.699 |
| MPO | TNF | 9606.ENSP00000225275 | 9606.ENSP00000398698 | 0 | 0 | 0 | 0 | 0.074 | 0 | 0 | 0.843 | 0.848 |
| MPO | PTGS1 | 9606.ENSP00000225275 | 9606.ENSP00000354612 | 0 | 0 | 0 | 0 | 0.076 | 0 | 0.9 | 0.408 | 0.94 |
| NFKB1 | VDR | 9606.ENSP00000226574 | 9606.ENSP00000447173 | 0 | 0 | 0 | 0 | 0.069 | 0.305 | 0 | 0.189 | 0.429 |
| NFKB1 | PTGS2 | 9606.ENSP00000226574 | 9606.ENSP00000356438 | 0 | 0 | 0 | 0 | 0.096 | 0 | 0 | 0.511 | 0.539 |
| NFKB1 | SP1 | 9606.ENSP00000226574 | 9606.ENSP00000329357 | 0 | 0 | 0 | 0 | 0.072 | 0.432 | 0 | 0.535 | 0.734 |
| NFKB1 | NFKB2 | 9606.ENSP00000226574 | 9606.ENSP00000358983 | 0 | 0 | 0 | 0.9 | 0.192 | 0.336 | 0.9 | 0.921 | 0.947 |
| NFKB1 | NR3C1 | 9606.ENSP00000226574 | 9606.ENSP00000231509 | 0 | 0 | 0 | 0 | 0.064 | 0.379 | 0.9 | 0.348 | 0.957 |
| NFKB1 | PPARG | 9606.ENSP00000226574 | 9606.ENSP00000287820 | 0 | 0 | 0 | 0 | 0.049 | 0.379 | 0.9 | 0.378 | 0.958 |
| NFKB1 | TNF | 9606.ENSP00000226574 | 9606.ENSP00000398698 | 0 | 0 | 0 | 0 | 0.108 | 0 | 0.9 | 0.672 | 0.968 |
| NFKB1 | RELA | 9606.ENSP00000226574 | 9606.ENSP00000384273 | 0 | 0 | 0 | 0.776 | 0.133 | 0.9 | 0.9 | 0.886 | 0.992 |
| NFKB2 | NR3C1 | 9606.ENSP00000358983 | 9606.ENSP00000231509 | 0 | 0 | 0 | 0 | 0 | 0.379 | 0 | 0.245 | 0.511 |
| NFKB2 | SP1 | 9606.ENSP00000358983 | 9606.ENSP00000329357 | 0 | 0 | 0 | 0 | 0.042 | 0.371 | 0 | 0.127 | 0.428 |
| NFKB2 | PTGS2 | 9606.ENSP00000358983 | 9606.ENSP00000356438 | 0 | 0 | 0 | 0 | 0.088 | 0 | 0 | 0.463 | 0.489 |
| NFKB2 | TNF | 9606.ENSP00000358983 | 9606.ENSP00000398698 | 0 | 0 | 0 | 0 | 0.117 | 0 | 0 | 0.786 | 0.803 |
| NFKB2 | RELA | 9606.ENSP00000358983 | 9606.ENSP00000384273 | 0 | 0 | 0 | 0.786 | 0.129 | 0.472 | 0.9 | 0.941 | 0.96 |
| NOS2 | PTGIS | 9606.ENSP00000327251 | 9606.ENSP00000244043 | 0.042 | 0 | 0 | 0 | 0.062 | 0.066 | 0 | 0.391 | 0.42 |
| NOS2 | SOD1 | 9606.ENSP00000327251 | 9606.ENSP00000270142 | 0 | 0 | 0 | 0 | 0 | 0 | 0 | 0.425 | 0.425 |
| NOS2 | PPARG | 9606.ENSP00000327251 | 9606.ENSP00000287820 | 0 | 0 | 0 | 0 | 0.062 | 0.091 | 0 | 0.417 | 0.459 |
| NOS2 | PTGS1 | 9606.ENSP00000327251 | 9606.ENSP00000354612 | 0 | 0 | 0 | 0 | 0 | 0.187 | 0 | 0.356 | 0.454 |
| NOS2 | SP1 | 9606.ENSP00000327251 | 9606.ENSP00000329357 | 0 | 0 | 0 | 0 | 0 | 0 | 0.9 | 0.08 | 0.904 |
| NOS2 | PTGS2 | 9606.ENSP00000327251 | 9606.ENSP00000356438 | 0 | 0 | 0 | 0 | 0.063 | 0.299 | 0 | 0.868 | 0.906 |
| NOS2 | PPARA | 9606.ENSP00000327251 | 9606.ENSP00000385523 | 0 | 0 | 0 | 0 | 0 | 0.091 | 0.9 | 0.211 | 0.922 |
| NOS2 | RELA | 9606.ENSP00000327251 | 9606.ENSP00000384273 | 0 | 0 | 0 | 0 | 0 | 0.343 | 0.9 | 0.327 | 0.951 |
| NOS2 | TNF | 9606.ENSP00000327251 | 9606.ENSP00000398698 | 0 | 0 | 0 | 0 | 0.063 | 0 | 0.9 | 0.844 | 0.984 |
| NR3C1 | SP1 | 9606.ENSP00000231509 | 9606.ENSP00000329357 | 0 | 0 | 0 | 0 | 0 | 0.111 | 0 | 0.415 | 0.457 |
| NR3C1 | PTGS2 | 9606.ENSP00000231509 | 9606.ENSP00000356438 | 0 | 0 | 0 | 0 | 0.069 | 0.05 | 0 | 0.475 | 0.495 |
| NR3C1 | PPARG | 9606.ENSP00000231509 | 9606.ENSP00000287820 | 0 | 0 | 0 | 0.578 | 0.06 | 0.379 | 0 | 0.595 | 0.539 |
| NR3C1 | SLC6A4 | 9606.ENSP00000231509 | 9606.ENSP00000261707 | 0 | 0 | 0 | 0 | 0 | 0.05 | 0 | 0.669 | 0.672 |
| NR3C1 | PGR | 9606.ENSP00000231509 | 9606.ENSP00000325120 | 0 | 0 | 0 | 0.839 | 0 | 0 | 0.9 | 0.78 | 0.912 |
| NR3C1 | TNF | 9606.ENSP00000231509 | 9606.ENSP00000398698 | 0 | 0 | 0 | 0 | 0 | 0 | 0.9 | 0.577 | 0.955 |
| NR3C1 | RELA | 9606.ENSP00000231509 | 9606.ENSP00000384273 | 0 | 0 | 0 | 0 | 0 | 0.393 | 0.9 | 0.443 | 0.963 |
| P2RY12 | PON1 | 9606.ENSP00000307259 | 9606.ENSP00000222381 | 0 | 0 | 0 | 0 | 0 | 0 | 0 | 0.478 | 0.478 |
| P2RY12 | PTGS1 | 9606.ENSP00000307259 | 9606.ENSP00000354612 | 0 | 0 | 0 | 0 | 0.059 | 0 | 0 | 0.642 | 0.648 |
| P2RY12 | PYY | 9606.ENSP00000307259 | 9606.ENSP00000353198 | 0 | 0 | 0 | 0 | 0 | 0 | 0.9 | 0 | 0.9 |
| PGR | SERPINE1 | 9606.ENSP00000325120 | 9606.ENSP00000223095 | 0 | 0 | 0 | 0 | 0 | 0.051 | 0 | 0.408 | 0.414 |
| PGR | PLG | 9606.ENSP00000325120 | 9606.ENSP00000308938 | 0 | 0 | 0 | 0 | 0.053 | 0 | 0 | 0.4 | 0.407 |
| PGR | PLAU | 9606.ENSP00000325120 | 9606.ENSP00000361850 | 0 | 0 | 0 | 0 | 0 | 0 | 0 | 0.416 | 0.416 |
| PGR | RELA | 9606.ENSP00000325120 | 9606.ENSP00000384273 | 0 | 0 | 0 | 0 | 0 | 0.393 | 0 | 0.217 | 0.505 |
| PGR | SHBG | 9606.ENSP00000325120 | 9606.ENSP00000369816 | 0 | 0 | 0 | 0 | 0 | 0 | 0 | 0.55 | 0.55 |
| PGR | PTGS2 | 9606.ENSP00000325120 | 9606.ENSP00000356438 | 0 | 0 | 0 | 0 | 0.061 | 0.05 | 0 | 0.573 | 0.585 |
| PGR | SP1 | 9606.ENSP00000325120 | 9606.ENSP00000329357 | 0 | 0 | 0 | 0 | 0 | 0.424 | 0 | 0.348 | 0.608 |
| PLAU | PON1 | 9606.ENSP00000361850 | 9606.ENSP00000222381 | 0 | 0 | 0 | 0 | 0 | 0 | 0 | 0.501 | 0.501 |
| PLAU | SERPINE1 | 9606.ENSP00000361850 | 9606.ENSP00000223095 | 0 | 0 | 0 | 0 | 0.214 | 0.881 | 0.9 | 0.926 | 0.999 |
| PLAU | PLG | 9606.ENSP00000361850 | 9606.ENSP00000308938 | 0 | 0 | 0 | 0.745 | 0 | 0.379 | 0.9 | 0.961 | 0.951 |
| PLAU | PTGS2 | 9606.ENSP00000361850 | 9606.ENSP00000356438 | 0 | 0 | 0 | 0 | 0.102 | 0.057 | 0 | 0.481 | 0.522 |
| PLAU | TNF | 9606.ENSP00000361850 | 9606.ENSP00000398698 | 0 | 0 | 0 | 0 | 0.061 | 0 | 0 | 0.678 | 0.685 |
| PLG | PON1 | 9606.ENSP00000308938 | 9606.ENSP00000222381 | 0 | 0 | 0 | 0 | 0.148 | 0 | 0 | 0.33 | 0.405 |
| PLG | SERPINE1 | 9606.ENSP00000308938 | 9606.ENSP00000223095 | 0 | 0 | 0 | 0 | 0.063 | 0.435 | 0.9 | 0.978 | 0.998 |
| PLG | SHBG | 9606.ENSP00000308938 | 9606.ENSP00000369816 | 0 | 0 | 0 | 0 | 0.109 | 0.082 | 0 | 0.348 | 0.42 |
| PLG | PTGS2 | 9606.ENSP00000308938 | 9606.ENSP00000356438 | 0 | 0 | 0 | 0 | 0 | 0.057 | 0 | 0.501 | 0.509 |
| PLG | TNF | 9606.ENSP00000308938 | 9606.ENSP00000398698 | 0 | 0 | 0 | 0 | 0 | 0 | 0 | 0.68 | 0.68 |
| PON1 | SERPINE1 | 9606.ENSP00000222381 | 9606.ENSP00000223095 | 0 | 0 | 0 | 0 | 0 | 0 | 0 | 0.4 | 0.4 |
| PON1 | SOD1 | 9606.ENSP00000222381 | 9606.ENSP00000270142 | 0 | 0 | 0 | 0 | 0.057 | 0 | 0 | 0.39 | 0.4 |
| PON1 | TNF | 9606.ENSP00000222381 | 9606.ENSP00000398698 | 0 | 0 | 0 | 0 | 0 | 0 | 0 | 0.643 | 0.643 |
| PON1 | PPARG | 9606.ENSP00000222381 | 9606.ENSP00000287820 | 0 | 0 | 0 | 0 | 0.061 | 0 | 0 | 0.655 | 0.662 |
| PPARA | SLC22A5 | 9606.ENSP00000385523 | 9606.ENSP00000245407 | 0 | 0 | 0 | 0 | 0.061 | 0 | 0 | 0.421 | 0.433 |
| PPARA | SP1 | 9606.ENSP00000385523 | 9606.ENSP00000329357 | 0 | 0 | 0 | 0 | 0 | 0.111 | 0.9 | 0.19 | 0.921 |
| PPARA | PTGS2 | 9606.ENSP00000385523 | 9606.ENSP00000356438 | 0 | 0 | 0 | 0 | 0 | 0.05 | 0 | 0.686 | 0.689 |
| PPARA | RELA | 9606.ENSP00000385523 | 9606.ENSP00000384273 | 0 | 0 | 0 | 0 | 0 | 0.393 | 0.9 | 0.21 | 0.947 |
| PPARA | TNF | 9606.ENSP00000385523 | 9606.ENSP00000398698 | 0 | 0 | 0 | 0 | 0 | 0 | 0.9 | 0.538 | 0.951 |
| PPARD | PTGS2 | 9606.ENSP00000310928 | 9606.ENSP00000356438 | 0 | 0 | 0 | 0 | 0 | 0.05 | 0 | 0.41 | 0.416 |
| PPARD | RELA | 9606.ENSP00000310928 | 9606.ENSP00000384273 | 0 | 0 | 0 | 0 | 0 | 0.393 | 0 | 0.214 | 0.503 |
| PPARG | SERPINE1 | 9606.ENSP00000287820 | 9606.ENSP00000223095 | 0 | 0 | 0 | 0 | 0.057 | 0.051 | 0 | 0.711 | 0.719 |
| PPARG | SLC22A5 | 9606.ENSP00000287820 | 9606.ENSP00000245407 | 0 | 0 | 0 | 0 | 0 | 0 | 0 | 0.47 | 0.47 |
| PPARG | SOD1 | 9606.ENSP00000287820 | 9606.ENSP00000270142 | 0 | 0 | 0 | 0 | 0 | 0 | 0 | 0.443 | 0.443 |
| PPARG | PTGS1 | 9606.ENSP00000287820 | 9606.ENSP00000354612 | 0 | 0 | 0 | 0 | 0.059 | 0.05 | 0 | 0.407 | 0.423 |
| PPARG | VDR | 9606.ENSP00000287820 | 9606.ENSP00000447173 | 0 | 0 | 0 | 0.684 | 0 | 0.379 | 0 | 0.52 | 0.477 |
| PPARG | SLC2A1 | 9606.ENSP00000287820 | 9606.ENSP00000416293 | 0 | 0 | 0 | 0 | 0 | 0 | 0 | 0.527 | 0.527 |
| PPARG | SP1 | 9606.ENSP00000287820 | 9606.ENSP00000329357 | 0 | 0 | 0 | 0 | 0 | 0.472 | 0 | 0.597 | 0.778 |
| PPARG | PTGS2 | 9606.ENSP00000287820 | 9606.ENSP00000356438 | 0 | 0 | 0 | 0 | 0 | 0.05 | 0 | 0.817 | 0.819 |
| PPARG | RELA | 9606.ENSP00000287820 | 9606.ENSP00000384273 | 0 | 0 | 0 | 0 | 0 | 0.472 | 0.9 | 0.348 | 0.962 |
| PPARG | TNF | 9606.ENSP00000287820 | 9606.ENSP00000398698 | 0 | 0 | 0 | 0 | 0 | 0 | 0.9 | 0.827 | 0.982 |
| PTGIR | PTGIS | 9606.ENSP00000291294 | 9606.ENSP00000244043 | 0 | 0 | 0 | 0 | 0 | 0 | 0 | 0.735 | 0.735 |
| PTGIR | PTGS1 | 9606.ENSP00000291294 | 9606.ENSP00000354612 | 0 | 0 | 0 | 0 | 0.061 | 0.056 | 0 | 0.57 | 0.585 |
| PTGIR | PTGS2 | 9606.ENSP00000291294 | 9606.ENSP00000356438 | 0 | 0 | 0 | 0 | 0.057 | 0.056 | 0 | 0.63 | 0.641 |
| PTGIS | PTGS2 | 9606.ENSP00000244043 | 9606.ENSP00000356438 | 0 | 0 | 0 | 0 | 0.061 | 0.379 | 0.9 | 0.609 | 0.974 |
| PTGIS | PTGS1 | 9606.ENSP00000244043 | 9606.ENSP00000354612 | 0 | 0 | 0 | 0 | 0.05 | 0.379 | 0.9 | 0.724 | 0.981 |
| PTGS1 | TNF | 9606.ENSP00000354612 | 9606.ENSP00000398698 | 0 | 0 | 0 | 0 | 0.084 | 0 | 0 | 0.498 | 0.521 |
| PTGS1 | PTGS2 | 9606.ENSP00000354612 | 9606.ENSP00000356438 | 0 | 0 | 0.432 | 0.967 | 0.14 | 0.379 | 0.8 | 0.956 | 0.889 |
| PTGS2 | SERPINE1 | 9606.ENSP00000356438 | 9606.ENSP00000223095 | 0 | 0 | 0 | 0 | 0.118 | 0 | 0 | 0.517 | 0.556 |
| PTGS2 | SOD1 | 9606.ENSP00000356438 | 9606.ENSP00000270142 | 0 | 0 | 0 | 0 | 0.061 | 0 | 0 | 0.467 | 0.478 |
| PTGS2 | SLC2A1 | 9606.ENSP00000356438 | 9606.ENSP00000416293 | 0 | 0 | 0 | 0 | 0.063 | 0 | 0 | 0.393 | 0.407 |
| PTGS2 | RELA | 9606.ENSP00000356438 | 9606.ENSP00000384273 | 0 | 0 | 0 | 0 | 0 | 0 | 0 | 0.558 | 0.558 |
| PTGS2 | TNF | 9606.ENSP00000356438 | 9606.ENSP00000398698 | 0 | 0 | 0 | 0 | 0.096 | 0 | 0 | 0.921 | 0.926 |
| PYY | VDR | 9606.ENSP00000353198 | 9606.ENSP00000447173 | 0 | 0 | 0 | 0 | 0 | 0 | 0 | 0.409 | 0.409 |
| RBP3 | RLBP1 | 9606.ENSP00000463151 | 9606.ENSP00000268125 | 0 | 0 | 0 | 0 | 0.185 | 0 | 0 | 0.681 | 0.729 |
| RELA | SP1 | 9606.ENSP00000384273 | 9606.ENSP00000329357 | 0 | 0 | 0 | 0 | 0.048 | 0.457 | 0.9 | 0.377 | 0.963 |
| RELA | VDR | 9606.ENSP00000384273 | 9606.ENSP00000447173 | 0 | 0 | 0 | 0 | 0 | 0.321 | 0 | 0.167 | 0.41 |
| RELA | TNF | 9606.ENSP00000384273 | 9606.ENSP00000398698 | 0 | 0 | 0 | 0 | 0 | 0.428 | 0.9 | 0.849 | 0.99 |
| SERPINE1 | SHBG | 9606.ENSP00000223095 | 9606.ENSP00000369816 | 0 | 0 | 0 | 0 | 0 | 0 | 0 | 0.429 | 0.429 |
| SERPINE1 | TNF | 9606.ENSP00000223095 | 9606.ENSP00000398698 | 0 | 0 | 0 | 0 | 0.062 | 0 | 0 | 0.86 | 0.863 |
| SLC2A1 | SP1 | 9606.ENSP00000416293 | 9606.ENSP00000329357 | 0 | 0 | 0 | 0 | 0.055 | 0 | 0 | 0.514 | 0.521 |
| SLC2A1 | TNF | 9606.ENSP00000416293 | 9606.ENSP00000398698 | 0 | 0 | 0 | 0 | 0 | 0 | 0 | 0.44 | 0.44 |
| SOD1 | TNF | 9606.ENSP00000270142 | 9606.ENSP00000398698 | 0 | 0 | 0 | 0 | 0.05 | 0 | 0 | 0.541 | 0.545 |
| SP1 | VDR | 9606.ENSP00000329357 | 9606.ENSP00000447173 | 0 | 0 | 0 | 0 | 0 | 0.111 | 0 | 0.561 | 0.593 |
| SP1 | TNF | 9606.ENSP00000329357 | 9606.ENSP00000398698 | 0 | 0 | 0 | 0 | 0 | 0.084 | 0.9 | 0.376 | 0.937 |
| TNF | VDR | 9606.ENSP00000398698 | 9606.ENSP00000447173 | 0 | 0 | 0 | 0 | 0.065 | 0 | 0 | 0.54 | 0.552 |
