# Supplementary material for: Exploring the Potential Mechanism of Chuanxiong Rhizoma Treatment for Migraine Based on Systems Pharmacology
Source: Evid Based Complement Alternat Med. 2021 Dec 28;2021:2809004. doi: 10.1155/2021/2809004 (PMC8727101; doi:10.1155/2021/2809004)
Supplement: Supplementary Materials — Supplementary Table S1: active compounds of CR). A total of 248 candidate compounds were included. Supplementary Table S2: relevant targets of CR active compounds. Supplementary Table S3: targets for Migraine. Supplementary Table S4: overlapping targets between disease and drug. Supplementary Table S5: details of the active compounds of CR and overlapping targets. Supplementary Table S6: the details of the PPI network. Supplementary Table S7: details of the GO enrichment analysis. Supplementary Table S8: details of the KEGG pathway enrichment analysis. [file 2809004.f1.zip › 2809004.f1/Supplementary Table S7 Details of the GO enrichment analysis (1).doc]

| ONTOLOGY | ID | Description | GeneRatio | BgRatio | pvalue | p.adjust | qvalue | geneID | Count |
| --- | --- | --- | --- | --- | --- | --- | --- | --- | --- |
| BP | GO:0060078 | regulation of postsynaptic membrane potential | 19/87 | 139/18493 | 5.62E-23 | 1.74E-19 | 9.64E-20 | CHRNA2/TRPV1/ADRB2/GSK3B/GRIA2/GABRA1/GABRA3/GABRA4/GABRA5/GABRB3/GABRD/GABRE/GABRG2/GRIN1/GRIN2A/GRIN2B/GRIN2D/AKT1/APP | 19 |
| BP | GO:0042391 | regulation of membrane potential | 26/87 | 424/18493 | 3.12E-22 | 4.84E-19 | 2.67E-19 | CHRNA2/TRPV1/SCN5A/ADRB2/KCNH2/GSK3B/GRIA2/SOD1/CCK/BCL2/GABRA1/GABRA3/GABRA4/GABRA5/GABRB3/GABRD/GABRE/GABRG2/GABRP/GABRQ/GRIN1/GRIN2A/GRIN2B/GRIN2D/AKT1/APP | 26 |
| BP | GO:0031667 | response to nutrient levels | 22/87 | 481/18493 | 4.02E-16 | 4.16E-13 | 2.30E-13 | PTGS2/TRPV1/ADA/ADRB2/SLC6A4/PPARG/F7/SOD1/HMGCR/MPO/PPARA/PPARD/PON1/CCK/BCL2/RELA/VDR/GRIN1/BCHE/AKT1/SLC2A1/P2RY12 | 22 |
| BP | GO:1903409 | reactive oxygen species biosynthetic process | 13/87 | 115/18493 | 7.22E-15 | 4.82E-12 | 2.66E-12 | PTGS2/TRPV1/HSP90AA1/MAOB/NOS2/SOD1/MPO/INS/IL10/GRIN1/AKT1/TNF/PTGIS | 13 |
| BP | GO:0042133 | neurotransmitter metabolic process | 14/87 | 148/18493 | 7.76E-15 | 4.82E-12 | 2.66E-12 | PTGS2/TRPV1/HSP90AA1/SLC6A3/SLC6A4/MAOB/NOS2/IL10/GRIN2A/ACHE/BCHE/AKT1/TNF/PTGIS | 14 |
| BP | GO:0099565 | chemical synaptic transmission, postsynaptic | 12/87 | 111/18493 | 1.45E-13 | 7.50E-11 | 4.14E-11 | CHRNA2/TRPV1/ADRB2/GSK3B/GRIA2/GABRB3/GRIN1/GRIN2A/GRIN2B/GRIN2D/AKT1/APP | 12 |
| BP | GO:0072593 | reactive oxygen species metabolic process | 16/87 | 276/18493 | 1.80E-13 | 7.98E-11 | 4.41E-11 | PTGS2/TRPV1/HSP90AA1/MAOB/NOS2/F2/SOD1/MPO/CRP/INS/BCL2/IL10/GRIN1/AKT1/TNF/PTGIS | 16 |
| BP | GO:0048545 | response to steroid hormone | 18/87 | 390/18493 | 2.35E-13 | 9.11E-11 | 5.03E-11 | PTGS2/MAOB/ESR1/AR/PPARG/ESR2/NR3C1/PGR/PPARA/PPARD/BCL2/IL10/RELA/IL6/VDR/BCHE/CASP3/TNF | 18 |
| BP | GO:0001505 | regulation of neurotransmitter levels | 17/87 | 346/18493 | 4.30E-13 | 1.48E-10 | 8.20E-11 | PTGS2/CHRM2/SLC6A2/TRPV1/HSP90AA1/SLC6A3/SLC6A4/MAOB/NOS2/GSK3B/IL10/GRIN2A/ACHE/BCHE/AKT1/TNF/PTGIS | 17 |
| BP | GO:0010883 | regulation of lipid storage | 9/87 | 45/18493 | 5.70E-13 | 1.77E-10 | 9.77E-11 | PPARG/LPL/PPARA/PPARD/CRP/IL6/ITGB3/NFKB1/TNF | 9 |
| BP | GO:0050727 | regulation of inflammatory response | 18/87 | 425/18493 | 1.01E-12 | 2.84E-10 | 1.57E-10 | PTGS2/ADA/NOS2/F2/ESR1/PPARG/LPL/SERPINE1/PPARA/PPARD/INS/IL10/RELA/IL6/APP/NFKB1/TNF/PTGIS | 18 |
| BP | GO:0008015 | blood circulation | 19/87 | 492/18493 | 1.10E-12 | 2.86E-10 | 1.58E-10 | PTGS2/CHRM2/PTGS1/SCN5A/ADA/ADRA1B/ADRB2/SLC6A4/NOS2/KCNH2/AR/PPARG/SOD1/HMGCR/PPARA/PPARD/CRP/INS/AKT1 | 19 |
| BP | GO:0003013 | circulatory system process | 19/87 | 500/18493 | 1.47E-12 | 3.26E-10 | 1.80E-10 | PTGS2/CHRM2/PTGS1/SCN5A/ADA/ADRA1B/ADRB2/SLC6A4/NOS2/KCNH2/AR/PPARG/SOD1/HMGCR/PPARA/PPARD/CRP/INS/AKT1 | 19 |
| BP | GO:0007584 | response to nutrient | 14/87 | 216/18493 | 1.47E-12 | 3.26E-10 | 1.80E-10 | PTGS2/TRPV1/ADA/SLC6A4/PPARG/F7/SOD1/HMGCR/PPARD/RELA/VDR/GRIN1/BCHE/P2RY12 | 14 |
| BP | GO:0042136 | neurotransmitter biosynthetic process | 11/87 | 102/18493 | 1.64E-12 | 3.39E-10 | 1.87E-10 | PTGS2/TRPV1/HSP90AA1/SLC6A3/SLC6A4/NOS2/IL10/ACHE/AKT1/TNF/PTGIS | 11 |
| BP | GO:0060079 | excitatory postsynaptic potential | 11/87 | 103/18493 | 1.83E-12 | 3.51E-10 | 1.94E-10 | CHRNA2/TRPV1/ADRB2/GSK3B/GRIA2/GRIN1/GRIN2A/GRIN2B/GRIN2D/AKT1/APP | 11 |
| BP | GO:0010888 | negative regulation of lipid storage | 7/87 | 19/18493 | 1.92E-12 | 3.51E-10 | 1.94E-10 | PPARG/PPARA/PPARD/CRP/IL6/ITGB3/TNF | 7 |
| BP | GO:0032102 | negative regulation of response to external stimulus | 16/87 | 329/18493 | 2.65E-12 | 4.57E-10 | 2.52E-10 | ADA/SLC6A3/F2/PPARG/PLAU/SERPINE1/HMGCR/PPARA/PPARD/INS/PLG/CCK/IL10/GRIN1/NFKB1/PTGIS | 16 |
| BP | GO:2000377 | regulation of reactive oxygen species metabolic process | 13/87 | 185/18493 | 3.53E-12 | 5.77E-10 | 3.18E-10 | PTGS2/TRPV1/HSP90AA1/F2/SOD1/CRP/INS/BCL2/IL10/GRIN1/AKT1/TNF/PTGIS | 13 |
| BP | GO:1905952 | regulation of lipid localization | 12/87 | 152/18493 | 6.40E-12 | 9.63E-10 | 5.32E-10 | PPARG/LPL/PPARA/PPARD/CRP/PON1/CETP/IL6/AKT1/ITGB3/NFKB1/TNF | 12 |
| BP | GO:1905953 | negative regulation of lipid localization | 9/87 | 58/18493 | 6.51E-12 | 9.63E-10 | 5.32E-10 | PPARG/PPARA/PPARD/CRP/IL6/AKT1/ITGB3/NFKB1/TNF | 9 |
| BP | GO:1902476 | chloride transmembrane transport | 10/87 | 87/18493 | 9.29E-12 | 1.31E-09 | 7.24E-10 | GABRA1/GABRA3/GABRA4/GABRA5/GABRB3/GABRD/GABRE/GABRG2/GABRP/GABRQ | 10 |
| BP | GO:0007214 | gamma-aminobutyric acid signaling pathway | 7/87 | 25/18493 | 1.79E-11 | 2.42E-09 | 1.33E-09 | GABRA1/GABRA3/GABRA4/GABRA5/GABRB3/GABRE/GABRG2 | 7 |
| BP | GO:0019915 | lipid storage | 9/87 | 66/18493 | 2.20E-11 | 2.84E-09 | 1.57E-09 | PPARG/LPL/PPARA/PPARD/CRP/IL6/ITGB3/NFKB1/TNF | 9 |
| BP | GO:0032496 | response to lipopolysaccharide | 15/87 | 324/18493 | 2.75E-11 | 3.41E-09 | 1.89E-09 | PTGS2/MAOB/NOS2/SERPINE1/MPO/PPARD/IL10/RELA/IL6/AKT1/CASP3/NFKB1/NFKB2/TNF/PTGIR | 15 |
| BP | GO:0002237 | response to molecule of bacterial origin | 15/87 | 337/18493 | 4.79E-11 | 5.72E-09 | 3.16E-09 | PTGS2/MAOB/NOS2/SERPINE1/MPO/PPARD/IL10/RELA/IL6/AKT1/CASP3/NFKB1/NFKB2/TNF/PTGIR | 15 |
| BP | GO:0050890 | cognition | 14/87 | 285/18493 | 6.13E-11 | 7.05E-09 | 3.89E-09 | PTGS2/SLC6A4/BDNF/HMGCR/INS/CCK/GABRA5/GRIN1/GRIN2A/GRIN2B/BCHE/APP/CASP3/TNF | 14 |
| BP | GO:0010743 | regulation of macrophage derived foam cell differentiation | 7/87 | 30/18493 | 7.44E-11 | 8.10E-09 | 4.47E-09 | PPARG/LPL/PPARA/CRP/CETP/ITGB3/NFKB1 | 7 |
| BP | GO:0006821 | chloride transport | 10/87 | 107/18493 | 7.56E-11 | 8.10E-09 | 4.47E-09 | GABRA1/GABRA3/GABRA4/GABRA5/GABRB3/GABRD/GABRE/GABRG2/GABRP/GABRQ | 10 |
| BP | GO:0070482 | response to oxygen levels | 15/87 | 349/18493 | 7.83E-11 | 8.10E-09 | 4.47E-09 | PTGS2/ADA/SLC6A4/NOS2/PPARG/F7/DPP4/PLAU/PPARA/PPARD/BCL2/AKT1/SLC2A1/CASP3/PTGIS | 15 |
| BP | GO:0098661 | inorganic anion transmembrane transport | 10/87 | 110/18493 | 9.98E-11 | 1.00E-08 | 5.52E-09 | GABRA1/GABRA3/GABRA4/GABRA5/GABRB3/GABRD/GABRE/GABRG2/GABRP/GABRQ | 10 |
| BP | GO:0051091 | positive regulation of DNA binding transcription factor activity | 13/87 | 255/18493 | 1.98E-10 | 1.93E-08 | 1.06E-08 | ESR1/AR/PPARG/ESR2/INS/IL10/RELA/IL6/AKT1/APP/NFKB1/NFKB2/TNF | 13 |
| BP | GO:0001666 | response to hypoxia | 14/87 | 314/18493 | 2.20E-10 | 2.07E-08 | 1.14E-08 | PTGS2/ADA/SLC6A4/NOS2/F7/DPP4/PLAU/PPARA/PPARD/BCL2/AKT1/SLC2A1/CASP3/PTGIS | 14 |
| BP | GO:0008202 | steroid metabolic process | 14/87 | 318/18493 | 2.60E-10 | 2.37E-08 | 1.31E-08 | ESR1/SOD1/HMGCR/PPARD/PON1/CETP/SP1/AKR1C1/VDR/HSD17B1/APP/HSD11B1/NFKB1/TNF | 14 |
| BP | GO:0010742 | macrophage derived foam cell differentiation | 7/87 | 36/18493 | 2.98E-10 | 2.57E-08 | 1.42E-08 | PPARG/LPL/PPARA/CRP/CETP/ITGB3/NFKB1 | 7 |
| BP | GO:0090077 | foam cell differentiation | 7/87 | 36/18493 | 2.98E-10 | 2.57E-08 | 1.42E-08 | PPARG/LPL/PPARA/CRP/CETP/ITGB3/NFKB1 | 7 |
| BP | GO:0046677 | response to antibiotic | 14/87 | 323/18493 | 3.19E-10 | 2.68E-08 | 1.48E-08 | HSP90AA1/ADA/SLC6A3/MAOB/SOD1/HMGCR/BCL2/IL10/RELA/IL6/GRIN1/GRIN2A/GRIN2B/CASP3 | 14 |
| BP | GO:0036293 | response to decreased oxygen levels | 14/87 | 325/18493 | 3.46E-10 | 2.82E-08 | 1.56E-08 | PTGS2/ADA/SLC6A4/NOS2/F7/DPP4/PLAU/PPARA/PPARD/BCL2/AKT1/SLC2A1/CASP3/PTGIS | 14 |
| BP | GO:0060135 | maternal process involved in female pregnancy | 8/87 | 60/18493 | 3.62E-10 | 2.88E-08 | 1.59E-08 | PTGS2/ESR1/AR/PGR/PPARD/VDR/AKT1/PTGIS | 8 |
| BP | GO:0035296 | regulation of tube diameter | 10/87 | 129/18493 | 4.87E-10 | 3.69E-08 | 2.04E-08 | PTGS2/ADRA1B/ADRB2/SLC6A4/SOD1/HMGCR/PPARD/CRP/INS/AKT1 | 10 |
| BP | GO:0097746 | regulation of blood vessel diameter | 10/87 | 129/18493 | 4.87E-10 | 3.69E-08 | 2.04E-08 | PTGS2/ADRA1B/ADRB2/SLC6A4/SOD1/HMGCR/PPARD/CRP/INS/AKT1 | 10 |
| BP | GO:0044706 | multi-multicellular organism process | 12/87 | 221/18493 | 5.07E-10 | 3.75E-08 | 2.07E-08 | PTGS2/SLC6A4/ESR1/AR/PGR/SOD1/PPARD/BCL2/VDR/AKT1/SLC2A1/PTGIS | 12 |
| BP | GO:0048511 | rhythmic process | 13/87 | 276/18493 | 5.26E-10 | 3.80E-08 | 2.10E-08 | ADA/SLC6A4/NOS2/ESR1/PPARG/F7/GSK3B/PGR/SERPINE1/PPARA/SP1/CASP3/NFKB2 | 13 |
| BP | GO:1903426 | regulation of reactive oxygen species biosynthetic process | 9/87 | 94/18493 | 5.67E-10 | 4.00E-08 | 2.21E-08 | PTGS2/TRPV1/HSP90AA1/INS/IL10/GRIN1/AKT1/TNF/PTGIS | 9 |
| BP | GO:0050880 | regulation of blood vessel size | 10/87 | 136/18493 | 8.19E-10 | 5.58E-08 | 3.08E-08 | PTGS2/ADRA1B/ADRB2/SLC6A4/SOD1/HMGCR/PPARD/CRP/INS/AKT1 | 10 |
| BP | GO:2000379 | positive regulation of reactive oxygen species metabolic process | 9/87 | 98/18493 | 8.27E-10 | 5.58E-08 | 3.08E-08 | PTGS2/TRPV1/HSP90AA1/F2/SOD1/CRP/GRIN1/AKT1/TNF | 9 |
| BP | GO:0035150 | regulation of tube size | 10/87 | 137/18493 | 8.81E-10 | 5.82E-08 | 3.21E-08 | PTGS2/ADRA1B/ADRB2/SLC6A4/SOD1/HMGCR/PPARD/CRP/INS/AKT1 | 10 |
| BP | GO:0050804 | modulation of chemical synaptic transmission | 15/87 | 418/18493 | 9.55E-10 | 6.17E-08 | 3.41E-08 | PTGS2/CHRM2/ADRB2/SLC6A4/GSK3B/BDNF/INS/GRIN1/GRIN2A/GRIN2B/GRIN2D/ACHE/BCHE/APP/TNF | 15 |
| BP | GO:0099177 | regulation of trans-synaptic signaling | 15/87 | 419/18493 | 9.86E-10 | 6.25E-08 | 3.45E-08 | PTGS2/CHRM2/ADRB2/SLC6A4/GSK3B/BDNF/INS/GRIN1/GRIN2A/GRIN2B/GRIN2D/ACHE/BCHE/APP/TNF | 15 |
| BP | GO:0032800 | receptor biosynthetic process | 6/87 | 24/18493 | 1.14E-09 | 7.11E-08 | 3.92E-08 | PPARG/PPARA/IL10/ACHE/ITGB3/TNF | 6 |
| BP | GO:0035235 | ionotropic glutamate receptor signaling pathway | 6/87 | 25/18493 | 1.50E-09 | 9.03E-08 | 4.99E-08 | GRIA2/GRIN1/GRIN2A/GRIN2B/GRIN2D/APP | 6 |
| BP | GO:0007565 | female pregnancy | 11/87 | 191/18493 | 1.51E-09 | 9.03E-08 | 4.99E-08 | PTGS2/ESR1/AR/PGR/SOD1/PPARD/BCL2/VDR/AKT1/SLC2A1/PTGIS | 11 |
| BP | GO:0007611 | learning or memory | 12/87 | 247/18493 | 1.81E-09 | 1.04E-07 | 5.75E-08 | PTGS2/SLC6A4/BDNF/HMGCR/CCK/GABRA5/GRIN1/GRIN2A/GRIN2B/BCHE/APP/CASP3 | 12 |
| BP | GO:0006809 | nitric oxide biosynthetic process | 8/87 | 73/18493 | 1.81E-09 | 1.04E-07 | 5.75E-08 | PTGS2/TRPV1/HSP90AA1/NOS2/IL10/AKT1/TNF/PTGIS | 8 |
| BP | GO:0006979 | response to oxidative stress | 15/87 | 441/18493 | 1.98E-09 | 1.12E-07 | 6.18E-08 | PTGS2/PTGS1/ADA/SOD1/MPO/INS/BCL2/IL10/SP1/RELA/IL6/AKT1/APP/CASP3/TNF | 15 |
| BP | GO:0006953 | acute-phase response | 7/87 | 47/18493 | 2.15E-09 | 1.17E-07 | 6.48E-08 | PTGS2/TRPV1/F2/CRP/INS/IL6/TNF | 7 |
| BP | GO:0035094 | response to nicotine | 7/87 | 47/18493 | 2.15E-09 | 1.17E-07 | 6.48E-08 | SLC6A3/PPARA/BCL2/RELA/CASP3/NFKB1/TNF | 7 |
| BP | GO:0007568 | aging | 13/87 | 311/18493 | 2.25E-09 | 1.20E-07 | 6.65E-08 | PTGS2/ADA/SLC6A3/SOD1/SERPINE1/HMGCR/MPO/BCL2/IL10/RELA/AKT1/APP/NFKB2 | 13 |
| BP | GO:0010745 | negative regulation of macrophage derived foam cell differentiation | 5/87 | 13/18493 | 2.56E-09 | 1.35E-07 | 7.45E-08 | PPARG/PPARA/CRP/CETP/ITGB3 | 5 |
| BP | GO:0046209 | nitric oxide metabolic process | 8/87 | 77/18493 | 2.79E-09 | 1.44E-07 | 7.98E-08 | PTGS2/TRPV1/HSP90AA1/NOS2/IL10/AKT1/TNF/PTGIS | 8 |
| BP | GO:0051235 | maintenance of location | 13/87 | 318/18493 | 2.94E-09 | 1.50E-07 | 8.27E-08 | TRPV1/F2/PPARG/LPL/PPARA/PPARD/CRP/IL10/IL6/AKT1/ITGB3/NFKB1/TNF | 13 |
| BP | GO:0019216 | regulation of lipid metabolic process | 14/87 | 388/18493 | 3.42E-09 | 1.71E-07 | 9.46E-08 | PTGS2/F2/PPARG/SOD1/HMGCR/PPARA/PPARD/INS/SP1/VDR/AKT1/NFKB1/TNF/P2RY12 | 14 |
| BP | GO:2001057 | reactive nitrogen species metabolic process | 8/87 | 80/18493 | 3.80E-09 | 1.87E-07 | 1.03E-07 | PTGS2/TRPV1/HSP90AA1/NOS2/IL10/AKT1/TNF/PTGIS | 8 |
| BP | GO:0010876 | lipid localization | 14/87 | 393/18493 | 4.03E-09 | 1.95E-07 | 1.08E-07 | NOS2/PPARG/LPL/PPARA/PPARD/CRP/PON1/CETP/IL6/AKR1C1/AKT1/ITGB3/NFKB1/TNF | 14 |
| BP | GO:0003018 | vascular process in circulatory system | 10/87 | 161/18493 | 4.25E-09 | 2.03E-07 | 1.12E-07 | PTGS2/ADRA1B/ADRB2/SLC6A4/SOD1/HMGCR/PPARD/CRP/INS/AKT1 | 10 |
| BP | GO:0070997 | neuron death | 13/87 | 335/18493 | 5.50E-09 | 2.59E-07 | 1.43E-07 | GSK3B/SOD1/BDNF/PPARA/BCL2/IL10/GABRA5/GABRB3/GRIN1/GRIN2B/AKT1/APP/CASP3 | 13 |
| BP | GO:1901652 | response to peptide | 15/87 | 481/18493 | 6.42E-09 | 2.97E-07 | 1.64E-07 | PTGS2/TRPV1/ADRB2/PPARG/F7/GSK3B/PPARA/INS/IL10/SP1/RELA/AKT1/APP/SLC2A1/NFKB1 | 15 |
| BP | GO:0051090 | regulation of DNA binding transcription factor activity | 14/87 | 412/18493 | 7.34E-09 | 3.35E-07 | 1.85E-07 | ESR1/AR/PPARG/ESR2/INS/IL10/RELA/IL6/AKT1/APP/NFKB1/NFKB2/TNF/PTGIS | 14 |
| BP | GO:0009410 | response to xenobiotic stimulus | 12/87 | 282/18493 | 8.03E-09 | 3.61E-07 | 2.00E-07 | PTGS1/ADA/SLC6A3/PPARG/F7/NR3C1/SOD1/RELA/AKR1C1/GRIN1/GRIN2A/BCHE | 12 |
| BP | GO:0060359 | response to ammonium ion | 9/87 | 128/18493 | 8.93E-09 | 3.91E-07 | 2.16E-07 | CHRM2/ADA/SLC6A3/RELA/GABRA1/GABRB3/GABRG2/GRIN1/CASP3 | 9 |
| BP | GO:0033273 | response to vitamin | 8/87 | 89/18493 | 8.95E-09 | 3.91E-07 | 2.16E-07 | PTGS2/ADA/PPARG/F7/PPARD/RELA/VDR/BCHE | 8 |
| BP | GO:0015698 | inorganic anion transport | 10/87 | 177/18493 | 1.06E-08 | 4.57E-07 | 2.52E-07 | GABRA1/GABRA3/GABRA4/GABRA5/GABRB3/GABRD/GABRE/GABRG2/GABRP/GABRQ | 10 |
| BP | GO:0035690 | cellular response to drug | 13/87 | 355/18493 | 1.10E-08 | 4.61E-07 | 2.55E-07 | PTGS2/CHRM2/TRPV1/NOS2/KCNH2/NR3C1/SOD1/IL10/RELA/IL6/NFKB1/TNF/P2RY12 | 13 |
| BP | GO:0062012 | regulation of small molecule metabolic process | 13/87 | 355/18493 | 1.10E-08 | 4.61E-07 | 2.55E-07 | PTGS2/NOS2/PPARG/GSK3B/SOD1/HMGCR/PPARA/INS/SP1/VDR/AKT1/NFKB1/TNF | 13 |
| BP | GO:0048608 | reproductive structure development | 14/87 | 428/18493 | 1.19E-08 | 4.92E-07 | 2.72E-07 | PTGS2/ADA/ESR1/AR/PPARG/PGR/SOD1/PPARD/BCL2/IL10/VDR/AKT1/CASP3/PTGIS | 14 |
| BP | GO:0061458 | reproductive system development | 14/87 | 431/18493 | 1.30E-08 | 5.31E-07 | 2.93E-07 | PTGS2/ADA/ESR1/AR/PPARG/PGR/SOD1/PPARD/BCL2/IL10/VDR/AKT1/CASP3/PTGIS | 14 |
| BP | GO:1901214 | regulation of neuron death | 12/87 | 296/18493 | 1.38E-08 | 5.55E-07 | 3.07E-07 | GSK3B/SOD1/BDNF/PPARA/BCL2/IL10/GABRA5/GABRB3/GRIN1/GRIN2B/AKT1/CASP3 | 12 |
| BP | GO:0045428 | regulation of nitric oxide biosynthetic process | 7/87 | 62/18493 | 1.59E-08 | 6.33E-07 | 3.50E-07 | PTGS2/TRPV1/HSP90AA1/IL10/AKT1/TNF/PTGIS | 7 |
| BP | GO:0046683 | response to organophosphorus | 9/87 | 137/18493 | 1.62E-08 | 6.35E-07 | 3.51E-07 | PTGS2/TRPV1/SLC6A3/SLC6A4/SOD1/RELA/AKR1C1/APP/P2RY12 | 9 |
| BP | GO:0019932 | second-messenger-mediated signaling | 14/87 | 439/18493 | 1.64E-08 | 6.35E-07 | 3.51E-07 | ADA/ADRA1B/ADRB2/NOS2/GSK3B/ADRA1D/INS/GRIN1/GRIN2A/GRIN2B/GRIN2D/TNF/P2RY12/PTGIR | 14 |
| BP | GO:0050728 | negative regulation of inflammatory response | 9/87 | 139/18493 | 1.84E-08 | 7.06E-07 | 3.90E-07 | ADA/F2/PPARG/PPARA/PPARD/INS/IL10/NFKB1/PTGIS | 9 |
| BP | GO:0031348 | negative regulation of defense response | 10/87 | 199/18493 | 3.24E-08 | 1.23E-06 | 6.77E-07 | ADA/F2/PPARG/PPARA/PPARD/INS/CCK/IL10/NFKB1/PTGIS | 10 |
| BP | GO:0001890 | placenta development | 9/87 | 149/18493 | 3.37E-08 | 1.25E-06 | 6.89E-07 | PTGS2/ADA/PPARG/SOD1/PPARD/IL10/VDR/AKT1/PTGIS | 9 |
| BP | GO:0051384 | response to glucocorticoid | 9/87 | 149/18493 | 3.37E-08 | 1.25E-06 | 6.89E-07 | PTGS2/MAOB/NR3C1/BCL2/IL10/IL6/BCHE/CASP3/TNF | 9 |
| BP | GO:1901215 | negative regulation of neuron death | 10/87 | 201/18493 | 3.56E-08 | 1.29E-06 | 7.14E-07 | GSK3B/SOD1/BDNF/PPARA/BCL2/IL10/GABRA5/GABRB3/GRIN1/AKT1 | 10 |
| BP | GO:0043279 | response to alkaloid | 8/87 | 106/18493 | 3.58E-08 | 1.29E-06 | 7.14E-07 | TRPV1/ADA/SLC6A3/PPARG/RELA/GRIN1/BCHE/CASP3 | 8 |
| BP | GO:0014074 | response to purine-containing compound | 9/87 | 151/18493 | 3.79E-08 | 1.35E-06 | 7.46E-07 | PTGS2/TRPV1/SLC6A3/SLC6A4/PPARG/SOD1/RELA/APP/P2RY12 | 9 |
| BP | GO:0001101 | response to acid chemical | 12/87 | 332/18493 | 4.88E-08 | 1.72E-06 | 9.52E-07 | PTGS2/SLC6A4/PPARG/F7/PON1/RELA/AKR1C1/GRIN1/BCHE/AKT1/CASP3/TNF | 12 |
| BP | GO:0098656 | anion transmembrane transport | 11/87 | 268/18493 | 5.10E-08 | 1.78E-06 | 9.83E-07 | GABRA1/GABRA3/GABRA4/GABRA5/GABRB3/GABRD/GABRE/GABRG2/GABRP/GABRQ/AKT1 | 11 |
| BP | GO:0048638 | regulation of developmental growth | 12/87 | 334/18493 | 5.22E-08 | 1.80E-06 | 9.94E-07 | SLC6A3/ADRB2/SLC6A4/AR/GSK3B/SOD1/BDNF/PPARA/PPARD/BCL2/AKT1/APP | 12 |
| BP | GO:0050806 | positive regulation of synaptic transmission | 9/87 | 159/18493 | 5.92E-08 | 2.02E-06 | 1.12E-06 | PTGS2/ADRB2/INS/GRIN1/GRIN2A/GRIN2B/GRIN2D/APP/TNF | 9 |
| BP | GO:0051932 | synaptic transmission, GABAergic | 6/87 | 45/18493 | 6.40E-08 | 2.16E-06 | 1.19E-06 | GABRA1/GABRA3/GABRA4/GABRA5/GABRE/GABRG2 | 6 |
| BP | GO:0051051 | negative regulation of transport | 14/87 | 495/18493 | 7.30E-08 | 2.44E-06 | 1.35E-06 | PTGS2/ADA/MAOB/KCNH2/HMGCR/INS/BCL2/IL10/GABRE/AKT1/ITGB3/NFKB1/TNF/P2RY12 | 14 |
| BP | GO:0050673 | epithelial cell proliferation | 13/87 | 420/18493 | 7.93E-08 | 2.62E-06 | 1.45E-06 | SCN5A/ESR1/AR/PPARG/PGR/ERBB2/PPARD/IL10/SP1/VDR/AKT1/ITGB3/TNF | 13 |
| BP | GO:0031960 | response to corticosteroid | 9/87 | 166/18493 | 8.58E-08 | 2.80E-06 | 1.55E-06 | PTGS2/MAOB/NR3C1/BCL2/IL10/IL6/BCHE/CASP3/TNF | 9 |
| BP | GO:0048660 | regulation of smooth muscle cell proliferation | 9/87 | 168/18493 | 9.51E-08 | 3.07E-06 | 1.70E-06 | PTGS2/PPARG/HMGCR/PPARD/IL10/IL6/AKT1/TNF/PTGIR | 9 |
| BP | GO:0000302 | response to reactive oxygen species | 10/87 | 224/18493 | 9.86E-08 | 3.16E-06 | 1.74E-06 | ADA/SOD1/MPO/BCL2/IL10/RELA/IL6/AKT1/CASP3/TNF | 10 |
| BP | GO:0097756 | negative regulation of blood vessel diameter | 7/87 | 81/18493 | 1.05E-07 | 3.29E-06 | 1.82E-06 | PTGS2/ADRA1B/SLC6A4/HMGCR/CRP/INS/AKT1 | 7 |
| BP | GO:0048659 | smooth muscle cell proliferation | 9/87 | 170/18493 | 1.05E-07 | 3.29E-06 | 1.82E-06 | PTGS2/PPARG/HMGCR/PPARD/IL10/IL6/AKT1/TNF/PTGIR | 9 |
| BP | GO:0030522 | intracellular receptor signaling pathway | 11/87 | 288/18493 | 1.06E-07 | 3.29E-06 | 1.82E-06 | ESR1/AR/PPARG/ESR2/NR3C1/PGR/PPARA/PPARD/RELA/VDR/PTGIS | 11 |
| BP | GO:0071216 | cellular response to biotic stimulus | 10/87 | 229/18493 | 1.21E-07 | 3.73E-06 | 2.06E-06 | NOS2/GSK3B/SERPINE1/PPARD/IL10/RELA/IL6/AKT1/NFKB1/TNF | 10 |
| BP | GO:0097305 | response to alcohol | 10/87 | 231/18493 | 1.31E-07 | 4.00E-06 | 2.21E-06 | SLC6A3/MAOB/PPARG/F7/SOD1/HMGCR/GRIN1/GRIN2A/GRIN2B/AKT1 | 10 |
| BP | GO:0008217 | regulation of blood pressure | 9/87 | 177/18493 | 1.49E-07 | 4.47E-06 | 2.47E-06 | PTGS2/PTGS1/ADRA1B/ADRB2/NOS2/AR/PPARG/SOD1/PPARA | 9 |
| BP | GO:0050678 | regulation of epithelial cell proliferation | 12/87 | 368/18493 | 1.50E-07 | 4.47E-06 | 2.47E-06 | SCN5A/AR/PPARG/PGR/ERBB2/PPARD/IL10/SP1/VDR/AKT1/ITGB3/TNF | 12 |
| BP | GO:0046890 | regulation of lipid biosynthetic process | 9/87 | 183/18493 | 1.97E-07 | 5.84E-06 | 3.22E-06 | PTGS2/SOD1/HMGCR/INS/SP1/VDR/AKT1/NFKB1/TNF | 9 |
| BP | GO:0007215 | glutamate receptor signaling pathway | 7/87 | 90/18493 | 2.17E-07 | 6.37E-06 | 3.52E-06 | ADRB2/GRIA2/GRIN1/GRIN2A/GRIN2B/GRIN2D/APP | 7 |
| BP | GO:1903428 | positive regulation of reactive oxygen species biosynthetic process | 6/87 | 55/18493 | 2.19E-07 | 6.37E-06 | 3.52E-06 | PTGS2/TRPV1/HSP90AA1/GRIN1/AKT1/TNF | 6 |
| BP | GO:0006367 | transcription initiation from RNA polymerase II promoter | 9/87 | 186/18493 | 2.27E-07 | 6.52E-06 | 3.60E-06 | ESR1/AR/PPARG/ESR2/NR3C1/PGR/PPARA/PPARD/VDR | 9 |
| BP | GO:0045834 | positive regulation of lipid metabolic process | 8/87 | 138/18493 | 2.79E-07 | 7.95E-06 | 4.39E-06 | PTGS2/F2/PPARG/PPARA/INS/AKT1/TNF/P2RY12 | 8 |
| BP | GO:0043401 | steroid hormone mediated signaling pathway | 9/87 | 191/18493 | 2.84E-07 | 8.01E-06 | 4.42E-06 | ESR1/AR/PPARG/ESR2/NR3C1/PGR/PPARA/PPARD/VDR | 9 |
| BP | GO:0001893 | maternal placenta development | 5/87 | 31/18493 | 3.17E-07 | 8.85E-06 | 4.89E-06 | PTGS2/PPARD/VDR/AKT1/PTGIS | 5 |
| BP | GO:0015850 | organic hydroxy compound transport | 10/87 | 255/18493 | 3.28E-07 | 9.04E-06 | 4.99E-06 | SLC6A2/SLC6A3/SLC6A4/MAOB/PPARG/PON1/CETP/AKR1C1/NFKB1/P2RY12 | 10 |
| BP | GO:0007612 | learning | 8/87 | 141/18493 | 3.29E-07 | 9.04E-06 | 4.99E-06 | PTGS2/HMGCR/CCK/GABRA5/GRIN1/GRIN2A/BCHE/APP | 8 |
| BP | GO:0062013 | positive regulation of small molecule metabolic process | 8/87 | 142/18493 | 3.47E-07 | 9.46E-06 | 5.23E-06 | PTGS2/NOS2/PPARG/PPARA/INS/AKT1/NFKB1/TNF | 8 |
| BP | GO:0045926 | negative regulation of growth | 10/87 | 260/18493 | 3.93E-07 | 1.06E-05 | 5.85E-06 | ADRB2/SLC6A4/PPARG/ESR2/MPO/PPARA/PPARD/BCL2/IL10/TNF | 10 |
| BP | GO:0071222 | cellular response to lipopolysaccharide | 9/87 | 199/18493 | 4.01E-07 | 1.07E-05 | 5.93E-06 | NOS2/SERPINE1/PPARD/IL10/RELA/IL6/AKT1/NFKB1/TNF | 9 |
| BP | GO:0016049 | cell growth | 13/87 | 485/18493 | 4.13E-07 | 1.10E-05 | 6.05E-06 | HSP90AA1/F2/PPARG/ESR2/GSK3B/ERBB2/BDNF/PPARA/PPARD/INS/BCL2/AKT1/APP | 13 |
| BP | GO:0006940 | regulation of smooth muscle contraction | 6/87 | 62/18493 | 4.53E-07 | 1.18E-05 | 6.53E-06 | PTGS2/CHRM2/ADA/ADRA1B/ADRB2/SOD1 | 6 |
| BP | GO:0032768 | regulation of monooxygenase activity | 6/87 | 62/18493 | 4.53E-07 | 1.18E-05 | 6.53E-06 | HSP90AA1/INS/VDR/AKT1/NFKB1/TNF | 6 |
| BP | GO:0042063 | gliogenesis | 10/87 | 266/18493 | 4.84E-07 | 1.25E-05 | 6.92E-06 | F2/PPARG/SOD1/ERBB2/RELA/IL6/AKT1/APP/TNF/P2RY12 | 10 |
| BP | GO:0019233 | sensory perception of pain | 7/87 | 102/18493 | 5.13E-07 | 1.32E-05 | 7.28E-06 | PTGS2/TRPV1/CCK/IL10/GRIN1/GRIN2A/GRIN2D | 7 |
| BP | GO:0071219 | cellular response to molecule of bacterial origin | 9/87 | 206/18493 | 5.37E-07 | 1.37E-05 | 7.55E-06 | NOS2/SERPINE1/PPARD/IL10/RELA/IL6/AKT1/NFKB1/TNF | 9 |
| BP | GO:0033138 | positive regulation of peptidyl-serine phosphorylation | 7/87 | 103/18493 | 5.49E-07 | 1.39E-05 | 7.65E-06 | PTGS2/HSP90AA1/BCL2/IL6/AKT1/APP/TNF | 7 |
| BP | GO:0043434 | response to peptide hormone | 12/87 | 417/18493 | 5.69E-07 | 1.42E-05 | 7.87E-06 | PTGS2/TRPV1/PPARG/F7/PPARA/INS/IL10/SP1/RELA/AKT1/SLC2A1/NFKB1 | 12 |
| BP | GO:0009266 | response to temperature stimulus | 9/87 | 208/18493 | 5.82E-07 | 1.45E-05 | 7.99E-06 | PTGS2/TRPV1/HSP90AA1/ADRB2/PPARG/GSK3B/SOD1/LPL/AKT1 | 9 |
| BP | GO:0010885 | regulation of cholesterol storage | 4/87 | 15/18493 | 5.99E-07 | 1.46E-05 | 8.06E-06 | PPARG/LPL/PPARA/PPARD | 4 |
| BP | GO:0030730 | sequestering of triglyceride | 4/87 | 15/18493 | 5.99E-07 | 1.46E-05 | 8.06E-06 | PPARG/LPL/PPARA/TNF | 4 |
| BP | GO:0033627 | cell adhesion mediated by integrin | 6/87 | 65/18493 | 6.02E-07 | 1.46E-05 | 8.06E-06 | ADA/DPP4/PLAU/SERPINE1/ITGB3/P2RY12 | 6 |
| BP | GO:0006939 | smooth muscle contraction | 7/87 | 105/18493 | 6.26E-07 | 1.51E-05 | 8.32E-06 | PTGS2/CHRM2/TRPV1/ADA/ADRA1B/ADRB2/SOD1 | 7 |
| BP | GO:0014015 | positive regulation of gliogenesis | 6/87 | 66/18493 | 6.60E-07 | 1.58E-05 | 8.70E-06 | PPARG/RELA/IL6/APP/TNF/P2RY12 | 6 |
| BP | GO:0006066 | alcohol metabolic process | 11/87 | 348/18493 | 6.99E-07 | 1.66E-05 | 9.16E-06 | SOD1/HMGCR/PPARD/PON1/CETP/SP1/AKR1C1/VDR/APP/NFKB1/TNF | 11 |
| BP | GO:0031670 | cellular response to nutrient | 6/87 | 68/18493 | 7.89E-07 | 1.86E-05 | 1.02E-05 | PTGS2/TRPV1/PPARG/SOD1/VDR/P2RY12 | 6 |
| BP | GO:1903034 | regulation of response to wounding | 8/87 | 160/18493 | 8.63E-07 | 2.01E-05 | 1.11E-05 | F2/F7/PLAU/SERPINE1/HMGCR/PPARD/PLG/IL10 | 8 |
| BP | GO:0048732 | gland development | 12/87 | 434/18493 | 8.67E-07 | 2.01E-05 | 1.11E-05 | ADA/SLC6A3/ESR1/AR/PGR/SOD1/BCL2/IL10/RELA/VDR/AKT1/TNF | 12 |
| BP | GO:0050708 | regulation of protein secretion | 12/87 | 438/18493 | 9.55E-07 | 2.20E-05 | 1.21E-05 | NOS2/DPP4/LPL/HMGCR/PPARD/CRP/INS/IL10/IL6/ACHE/SLC2A1/TNF | 12 |
| BP | GO:0014013 | regulation of gliogenesis | 7/87 | 112/18493 | 9.70E-07 | 2.21E-05 | 1.22E-05 | F2/PPARG/RELA/IL6/APP/TNF/P2RY12 | 7 |
| BP | GO:0010878 | cholesterol storage | 4/87 | 17/18493 | 1.04E-06 | 2.34E-05 | 1.29E-05 | PPARG/LPL/PPARA/PPARD | 4 |
| BP | GO:0031649 | heat generation | 4/87 | 17/18493 | 1.04E-06 | 2.34E-05 | 1.29E-05 | PTGS2/TRPV1/ADRB2/TNF | 4 |
| BP | GO:0033555 | multicellular organismal response to stress | 6/87 | 72/18493 | 1.11E-06 | 2.48E-05 | 1.37E-05 | SLC6A2/TRPV1/DPP4/CCK/BCL2/GABRA5 | 6 |
| BP | GO:0006874 | cellular calcium ion homeostasis | 12/87 | 448/18493 | 1.21E-06 | 2.68E-05 | 1.48E-05 | TRPV1/ADRA1B/F2/ESR1/BCL2/VDR/GRIN1/GRIN2A/GRIN2B/GRIN2D/APP/PTGIR | 12 |
| BP | GO:0006631 | fatty acid metabolic process | 11/87 | 369/18493 | 1.24E-06 | 2.72E-05 | 1.50E-05 | PTGS2/PTGS1/PPARG/LPL/PPARA/PPARD/PON1/INS/AKT1/ALOX5/PTGIS | 11 |
| BP | GO:0051897 | positive regulation of protein kinase B signaling | 8/87 | 168/18493 | 1.25E-06 | 2.72E-05 | 1.50E-05 | HSP90AA1/ESR1/F7/F10/ERBB2/INS/TNF/P2RY12 | 8 |
| BP | GO:0051896 | regulation of protein kinase B signaling | 9/87 | 228/18493 | 1.25E-06 | 2.72E-05 | 1.50E-05 | HSP90AA1/ESR1/F7/F10/ERBB2/INS/AKT1/TNF/P2RY12 | 9 |
| BP | GO:0032103 | positive regulation of response to external stimulus | 10/87 | 297/18493 | 1.32E-06 | 2.84E-05 | 1.57E-05 | PTGS2/F2/F7/LPL/SERPINE1/PLG/IL6/APP/TNF/P2RY12 | 10 |
| BP | GO:0071318 | cellular response to ATP | 4/87 | 18/18493 | 1.33E-06 | 2.85E-05 | 1.57E-05 | PTGS2/TRPV1/SOD1/P2RY12 | 4 |
| BP | GO:2000116 | regulation of cysteine-type endopeptidase activity | 9/87 | 232/18493 | 1.45E-06 | 3.07E-05 | 1.70E-05 | PTGS2/PPARG/CCK/GRIN1/GRIN2A/GRIN2B/AKT1/CASP3/TNF | 9 |
| BP | GO:0006775 | fat-soluble vitamin metabolic process | 5/87 | 42/18493 | 1.52E-06 | 3.17E-05 | 1.75E-05 | PPARD/VDR/NFKB1/TNF/RLBP1 | 5 |
| BP | GO:0045429 | positive regulation of nitric oxide biosynthetic process | 5/87 | 42/18493 | 1.52E-06 | 3.17E-05 | 1.75E-05 | PTGS2/TRPV1/HSP90AA1/AKT1/TNF | 5 |
| BP | GO:1904407 | positive regulation of nitric oxide metabolic process | 5/87 | 42/18493 | 1.52E-06 | 3.17E-05 | 1.75E-05 | PTGS2/TRPV1/HSP90AA1/AKT1/TNF | 5 |
| BP | GO:0097237 | cellular response to toxic substance | 9/87 | 235/18493 | 1.61E-06 | 3.33E-05 | 1.84E-05 | PTGS2/PTGS1/SOD1/MPO/IL10/RELA/IL6/NFKB1/TNF | 9 |
| BP | GO:0055074 | calcium ion homeostasis | 12/87 | 461/18493 | 1.63E-06 | 3.35E-05 | 1.85E-05 | TRPV1/ADRA1B/F2/ESR1/BCL2/VDR/GRIN1/GRIN2A/GRIN2B/GRIN2D/APP/PTGIR | 12 |
| BP | GO:0008306 | associative learning | 6/87 | 77/18493 | 1.65E-06 | 3.37E-05 | 1.86E-05 | HMGCR/CCK/GABRA5/GRIN1/GRIN2A/APP | 6 |
| BP | GO:0033002 | muscle cell proliferation | 9/87 | 236/18493 | 1.67E-06 | 3.38E-05 | 1.87E-05 | PTGS2/PPARG/HMGCR/PPARD/IL10/IL6/AKT1/TNF/PTGIR | 9 |
| BP | GO:0032368 | regulation of lipid transport | 7/87 | 122/18493 | 1.73E-06 | 3.48E-05 | 1.92E-05 | PPARG/PPARA/PON1/CETP/AKT1/ITGB3/NFKB1 | 7 |
| BP | GO:0002791 | regulation of peptide secretion | 12/87 | 465/18493 | 1.78E-06 | 3.57E-05 | 1.97E-05 | NOS2/DPP4/LPL/HMGCR/PPARD/CRP/INS/IL10/IL6/ACHE/SLC2A1/TNF | 12 |
| BP | GO:0009416 | response to light stimulus | 10/87 | 308/18493 | 1.83E-06 | 3.64E-05 | 2.01E-05 | PTGS2/HMGCR/CCK/BCL2/RELA/GRIN1/GRIN2A/AKT1/APP/CASP3 | 10 |
| BP | GO:0007204 | positive regulation of cytosolic calcium ion concentration | 10/87 | 309/18493 | 1.88E-06 | 3.72E-05 | 2.06E-05 | TRPV1/ADRA1B/F2/ESR1/BCL2/GRIN1/GRIN2A/GRIN2B/GRIN2D/PTGIR | 10 |
| BP | GO:0009755 | hormone-mediated signaling pathway | 9/87 | 240/18493 | 1.91E-06 | 3.74E-05 | 2.07E-05 | ESR1/AR/PPARG/ESR2/NR3C1/PGR/PPARA/PPARD/VDR | 9 |
| BP | GO:0033628 | regulation of cell adhesion mediated by integrin | 5/87 | 44/18493 | 1.93E-06 | 3.74E-05 | 2.07E-05 | ADA/DPP4/PLAU/SERPINE1/P2RY12 | 5 |
| BP | GO:0061614 | pri-miRNA transcription by RNA polymerase II | 5/87 | 44/18493 | 1.93E-06 | 3.74E-05 | 2.07E-05 | PPARG/PPARA/PPARD/IL10/RELA | 5 |
| BP | GO:1902930 | regulation of alcohol biosynthetic process | 6/87 | 80/18493 | 2.07E-06 | 3.99E-05 | 2.20E-05 | SOD1/HMGCR/SP1/VDR/NFKB1/TNF | 6 |
| BP | GO:0046697 | decidualization | 4/87 | 20/18493 | 2.09E-06 | 3.99E-05 | 2.20E-05 | PTGS2/PPARD/VDR/PTGIS | 4 |
| BP | GO:0002685 | regulation of leukocyte migration | 8/87 | 180/18493 | 2.09E-06 | 3.99E-05 | 2.20E-05 | ADA/F7/SERPINE1/IL6/AKT1/APP/TNF/P2RY12 | 8 |
| BP | GO:0031644 | regulation of neurological system process | 7/87 | 127/18493 | 2.26E-06 | 4.25E-05 | 2.35E-05 | ADRB2/CCK/IL10/GRIN1/GRIN2A/GRIN2D/APP | 7 |
| BP | GO:0045471 | response to ethanol | 7/87 | 127/18493 | 2.26E-06 | 4.25E-05 | 2.35E-05 | SLC6A3/MAOB/SOD1/HMGCR/GRIN1/GRIN2A/GRIN2B | 7 |
| BP | GO:0045598 | regulation of fat cell differentiation | 7/87 | 128/18493 | 2.38E-06 | 4.45E-05 | 2.46E-05 | PTGS2/PPARG/PPARD/INS/IL6/AKT1/TNF | 7 |
| BP | GO:0006352 | DNA-templated transcription, initiation | 9/87 | 248/18493 | 2.50E-06 | 4.66E-05 | 2.57E-05 | ESR1/AR/PPARG/ESR2/NR3C1/PGR/PPARA/PPARD/VDR | 9 |
| BP | GO:0072503 | cellular divalent inorganic cation homeostasis | 12/87 | 483/18493 | 2.64E-06 | 4.85E-05 | 2.68E-05 | TRPV1/ADRA1B/F2/ESR1/BCL2/VDR/GRIN1/GRIN2A/GRIN2B/GRIN2D/APP/PTGIR | 12 |
| BP | GO:0006766 | vitamin metabolic process | 7/87 | 130/18493 | 2.64E-06 | 4.85E-05 | 2.68E-05 | PRSS3/PPARD/VDR/SLC2A1/NFKB1/TNF/RLBP1 | 7 |
| BP | GO:0043491 | protein kinase B signaling | 9/87 | 253/18493 | 2.95E-06 | 5.39E-05 | 2.98E-05 | HSP90AA1/ESR1/F7/F10/ERBB2/INS/AKT1/TNF/P2RY12 | 9 |
| BP | GO:0008542 | visual learning | 5/87 | 48/18493 | 3.00E-06 | 5.43E-05 | 3.00E-05 | HMGCR/CCK/GRIN1/GRIN2A/APP | 5 |
| BP | GO:0010952 | positive regulation of peptidase activity | 8/87 | 189/18493 | 3.01E-06 | 5.43E-05 | 3.00E-05 | PPARG/CCK/GRIN1/GRIN2A/GRIN2B/APP/CASP3/TNF | 8 |
| BP | GO:0071496 | cellular response to external stimulus | 10/87 | 328/18493 | 3.21E-06 | 5.76E-05 | 3.18E-05 | PTGS2/TRPV1/PPARG/SOD1/BCL2/VDR/AKT1/SLC2A1/NFKB1/P2RY12 | 10 |
| BP | GO:0061041 | regulation of wound healing | 7/87 | 134/18493 | 3.23E-06 | 5.77E-05 | 3.19E-05 | F2/F7/PLAU/SERPINE1/HMGCR/PPARD/PLG | 7 |
| BP | GO:0050878 | regulation of body fluid levels | 12/87 | 493/18493 | 3.26E-06 | 5.78E-05 | 3.19E-05 | ADA/SLC6A3/F2/F7/F10/PLAU/SERPINE1/PLG/IL6/VDR/ITGB3/P2RY12 | 12 |
| BP | GO:0033135 | regulation of peptidyl-serine phosphorylation | 7/87 | 135/18493 | 3.40E-06 | 5.99E-05 | 3.31E-05 | PTGS2/HSP90AA1/BCL2/IL6/AKT1/APP/TNF | 7 |
| BP | GO:0006694 | steroid biosynthetic process | 8/87 | 193/18493 | 3.52E-06 | 6.13E-05 | 3.39E-05 | SOD1/HMGCR/SP1/VDR/HSD17B1/HSD11B1/NFKB1/TNF | 8 |
| BP | GO:0007623 | circadian rhythm | 8/87 | 193/18493 | 3.52E-06 | 6.13E-05 | 3.39E-05 | ADA/SLC6A4/NOS2/PPARG/F7/GSK3B/SERPINE1/PPARA | 8 |
| BP | GO:0090066 | regulation of anatomical structure size | 12/87 | 497/18493 | 3.54E-06 | 6.14E-05 | 3.39E-05 | PTGS2/ADRA1B/ADRB2/SLC6A4/GSK3B/SOD1/BDNF/HMGCR/PPARD/CRP/INS/AKT1 | 12 |
| BP | GO:0071383 | cellular response to steroid hormone stimulus | 9/87 | 259/18493 | 3.57E-06 | 6.16E-05 | 3.40E-05 | ESR1/AR/PPARG/ESR2/NR3C1/PGR/PPARA/PPARD/VDR | 9 |
| BP | GO:0010001 | glial cell differentiation | 8/87 | 194/18493 | 3.65E-06 | 6.27E-05 | 3.46E-05 | F2/PPARG/SOD1/ERBB2/RELA/AKT1/APP/TNF | 8 |
| BP | GO:0043270 | positive regulation of ion transport | 9/87 | 260/18493 | 3.69E-06 | 6.29E-05 | 3.47E-05 | SCN5A/ADRB2/SLC6A4/F2/KCNH2/CCK/GRIN1/AKT1/P2RY12 | 9 |
| BP | GO:0001558 | regulation of cell growth | 11/87 | 416/18493 | 3.95E-06 | 6.70E-05 | 3.70E-05 | F2/PPARG/ESR2/GSK3B/ERBB2/BDNF/PPARA/PPARD/INS/BCL2/AKT1 | 11 |
| BP | GO:0052548 | regulation of endopeptidase activity | 11/87 | 417/18493 | 4.04E-06 | 6.82E-05 | 3.77E-05 | PTGS2/PPARG/SERPINE1/CCK/GRIN1/GRIN2A/GRIN2B/AKT1/APP/CASP3/TNF | 11 |
| BP | GO:0050810 | regulation of steroid biosynthetic process | 6/87 | 91/18493 | 4.41E-06 | 7.36E-05 | 4.07E-05 | SOD1/HMGCR/SP1/VDR/NFKB1/TNF | 6 |
| BP | GO:0097164 | ammonium ion metabolic process | 8/87 | 199/18493 | 4.41E-06 | 7.36E-05 | 4.07E-05 | SLC6A3/MAOB/PON1/CETP/AKR1C1/GRIN2A/ACHE/BCHE | 8 |
| BP | GO:0001819 | positive regulation of cytokine production | 11/87 | 422/18493 | 4.53E-06 | 7.49E-05 | 4.13E-05 | PTGS2/SOD1/LPL/SERPINE1/INS/IL10/RELA/IL6/NFKB1/NFKB2/TNF | 11 |
| BP | GO:0042542 | response to hydrogen peroxide | 7/87 | 141/18493 | 4.53E-06 | 7.49E-05 | 4.13E-05 | ADA/SOD1/BCL2/IL10/RELA/IL6/CASP3 | 7 |
| BP | GO:0007632 | visual behavior | 5/87 | 53/18493 | 4.93E-06 | 8.10E-05 | 4.47E-05 | HMGCR/CCK/GRIN1/GRIN2A/APP | 5 |
| BP | GO:2001056 | positive regulation of cysteine-type endopeptidase activity | 7/87 | 143/18493 | 4.98E-06 | 8.13E-05 | 4.49E-05 | PPARG/CCK/GRIN1/GRIN2A/GRIN2B/CASP3/TNF | 7 |
| BP | GO:0008203 | cholesterol metabolic process | 7/87 | 144/18493 | 5.21E-06 | 8.47E-05 | 4.68E-05 | SOD1/HMGCR/PPARD/PON1/CETP/SP1/APP | 7 |
| BP | GO:0030194 | positive regulation of blood coagulation | 4/87 | 25/18493 | 5.36E-06 | 8.58E-05 | 4.74E-05 | F2/F7/SERPINE1/PLG | 4 |
| BP | GO:0060444 | branching involved in mammary gland duct morphogenesis | 4/87 | 25/18493 | 5.36E-06 | 8.58E-05 | 4.74E-05 | ESR1/AR/PGR/VDR | 4 |
| BP | GO:1900048 | positive regulation of hemostasis | 4/87 | 25/18493 | 5.36E-06 | 8.58E-05 | 4.74E-05 | F2/F7/SERPINE1/PLG | 4 |
| BP | GO:0051480 | regulation of cytosolic calcium ion concentration | 10/87 | 350/18493 | 5.70E-06 | 9.04E-05 | 4.99E-05 | TRPV1/ADRA1B/F2/ESR1/BCL2/GRIN1/GRIN2A/GRIN2B/GRIN2D/PTGIR | 10 |
| BP | GO:0051092 | positive regulation of NF-kappaB transcription factor activity | 7/87 | 146/18493 | 5.71E-06 | 9.04E-05 | 4.99E-05 | AR/INS/RELA/APP/NFKB1/NFKB2/TNF | 7 |
| BP | GO:0090303 | positive regulation of wound healing | 5/87 | 55/18493 | 5.93E-06 | 9.35E-05 | 5.16E-05 | F2/F7/SERPINE1/PPARD/PLG | 5 |
| BP | GO:0045862 | positive regulation of proteolysis | 10/87 | 352/18493 | 5.99E-06 | 9.39E-05 | 5.19E-05 | PPARG/GSK3B/CCK/GRIN1/GRIN2A/GRIN2B/AKT1/APP/CASP3/TNF | 10 |
| BP | GO:1904951 | positive regulation of establishment of protein localization | 11/87 | 437/18493 | 6.31E-06 | 9.80E-05 | 5.41E-05 | PTGS2/GSK3B/ERBB2/LPL/PPARD/INS/BCL2/IL10/IL6/ACHE/TNF | 11 |
| BP | GO:0050820 | positive regulation of coagulation | 4/87 | 26/18493 | 6.31E-06 | 9.80E-05 | 5.41E-05 | F2/F7/SERPINE1/PLG | 4 |
| BP | GO:0060402 | calcium ion transport into cytosol | 7/87 | 149/18493 | 6.52E-06 | 0.000100294 | 5.54E-05 | TRPV1/F2/BCL2/GRIN1/GRIN2A/GRIN2B/GRIN2D | 7 |
| BP | GO:1902652 | secondary alcohol metabolic process | 7/87 | 149/18493 | 6.52E-06 | 0.000100294 | 5.54E-05 | SOD1/HMGCR/PPARD/PON1/CETP/SP1/APP | 7 |
| BP | GO:0048661 | positive regulation of smooth muscle cell proliferation | 6/87 | 98/18493 | 6.78E-06 | 0.000103306 | 5.71E-05 | PTGS2/HMGCR/IL10/IL6/AKT1/TNF | 6 |
| BP | GO:0002526 | acute inflammatory response | 8/87 | 211/18493 | 6.79E-06 | 0.000103306 | 5.71E-05 | PTGS2/TRPV1/F2/PPARG/CRP/INS/IL6/TNF | 8 |
| BP | GO:0052547 | regulation of peptidase activity | 11/87 | 442/18493 | 7.02E-06 | 0.000106373 | 5.87E-05 | PTGS2/PPARG/SERPINE1/CCK/GRIN1/GRIN2A/GRIN2B/AKT1/APP/CASP3/TNF | 11 |
| BP | GO:0006869 | lipid transport | 10/87 | 359/18493 | 7.12E-06 | 0.000107321 | 5.93E-05 | NOS2/PPARG/PPARA/PPARD/PON1/CETP/AKR1C1/AKT1/ITGB3/NFKB1 | 10 |
| BP | GO:0051341 | regulation of oxidoreductase activity | 6/87 | 99/18493 | 7.19E-06 | 0.000107819 | 5.95E-05 | HSP90AA1/INS/VDR/AKT1/NFKB1/TNF | 6 |
| BP | GO:0009895 | negative regulation of catabolic process | 9/87 | 287/18493 | 8.20E-06 | 0.000122413 | 6.76E-05 | NOS2/PPARA/INS/BCL2/IL10/RELA/GRIN2A/AKT1/TNF | 9 |
| BP | GO:0043393 | regulation of protein binding | 8/87 | 217/18493 | 8.33E-06 | 0.000123218 | 6.80E-05 | ADRB2/GSK3B/BDNF/PPARA/BCL2/IL10/AKT1/APP | 8 |
| BP | GO:0045444 | fat cell differentiation | 8/87 | 217/18493 | 8.33E-06 | 0.000123218 | 6.80E-05 | PTGS2/ADRB2/PPARG/PPARD/INS/IL6/AKT1/TNF | 8 |
| BP | GO:0001964 | startle response | 4/87 | 28/18493 | 8.58E-06 | 0.000125632 | 6.94E-05 | SLC6A3/GRIN1/GRIN2A/GRIN2D | 4 |
| BP | GO:0042730 | fibrinolysis | 4/87 | 28/18493 | 8.58E-06 | 0.000125632 | 6.94E-05 | F2/PLAU/SERPINE1/PLG | 4 |
| BP | GO:0042445 | hormone metabolic process | 8/87 | 218/18493 | 8.62E-06 | 0.000125632 | 6.94E-05 | ESR1/AKR1C1/VDR/HSD17B1/ACHE/HSD11B1/NFKB1/TNF | 8 |
| BP | GO:0034599 | cellular response to oxidative stress | 9/87 | 289/18493 | 8.67E-06 | 0.000125804 | 6.95E-05 | SOD1/MPO/INS/BCL2/IL10/RELA/IL6/AKT1/TNF | 9 |
| BP | GO:0051098 | regulation of binding | 10/87 | 369/18493 | 9.05E-06 | 0.000130729 | 7.22E-05 | ADRB2/PPARG/GSK3B/BDNF/PPARA/PON1/BCL2/IL10/AKT1/APP | 10 |
| BP | GO:0019722 | calcium-mediated signaling | 8/87 | 220/18493 | 9.21E-06 | 0.000132425 | 7.31E-05 | ADA/GSK3B/GRIN1/GRIN2A/GRIN2B/GRIN2D/TNF/P2RY12 | 8 |
| BP | GO:2001237 | negative regulation of extrinsic apoptotic signaling pathway | 6/87 | 104/18493 | 9.55E-06 | 0.000136689 | 7.55E-05 | AR/SERPINE1/BCL2/RELA/AKT1/TNF | 6 |
| BP | GO:1903037 | regulation of leukocyte cell-cell adhesion | 9/87 | 293/18493 | 9.68E-06 | 0.000137883 | 7.61E-05 | ADA/DPP4/ERBB2/PPARA/IL10/IL6/AKT1/CASP3/TNF | 9 |
| BP | GO:2001234 | negative regulation of apoptotic signaling pathway | 8/87 | 222/18493 | 9.84E-06 | 0.000139517 | 7.70E-05 | PTGS2/AR/SERPINE1/INS/BCL2/RELA/AKT1/TNF | 8 |
| BP | GO:0097191 | extrinsic apoptotic signaling pathway | 8/87 | 224/18493 | 1.05E-05 | 0.000148254 | 8.19E-05 | AR/GSK3B/SERPINE1/BCL2/RELA/AKT1/CASP3/TNF | 8 |
| BP | GO:0060401 | cytosolic calcium ion transport | 7/87 | 161/18493 | 1.08E-05 | 0.000152294 | 8.41E-05 | TRPV1/F2/BCL2/GRIN1/GRIN2A/GRIN2B/GRIN2D | 7 |
| BP | GO:0016125 | sterol metabolic process | 7/87 | 162/18493 | 1.13E-05 | 0.000157148 | 8.68E-05 | SOD1/HMGCR/PPARD/PON1/CETP/SP1/APP | 7 |
| BP | GO:0034614 | cellular response to reactive oxygen species | 7/87 | 162/18493 | 1.13E-05 | 0.000157148 | 8.68E-05 | SOD1/MPO/IL10/RELA/IL6/AKT1/TNF | 7 |
| BP | GO:0002532 | production of molecular mediator involved in inflammatory response | 5/87 | 63/18493 | 1.16E-05 | 0.00016128 | 8.91E-05 | NOS2/F2/SERPINE1/INS/ALOX5 | 5 |
| BP | GO:0001660 | fever generation | 3/87 | 10/18493 | 1.18E-05 | 0.000161901 | 8.94E-05 | PTGS2/TRPV1/TNF | 3 |
| BP | GO:0051918 | negative regulation of fibrinolysis | 3/87 | 10/18493 | 1.18E-05 | 0.000161901 | 8.94E-05 | F2/SERPINE1/PLG | 3 |
| BP | GO:0031669 | cellular response to nutrient levels | 8/87 | 228/18493 | 1.19E-05 | 0.000163405 | 9.02E-05 | PTGS2/TRPV1/PPARG/SOD1/BCL2/VDR/SLC2A1/P2RY12 | 8 |
| BP | GO:0006937 | regulation of muscle contraction | 7/87 | 164/18493 | 1.22E-05 | 0.000166495 | 9.19E-05 | PTGS2/CHRM2/SCN5A/ADA/ADRA1B/ADRB2/SOD1 | 7 |
| BP | GO:0015696 | ammonium transport | 6/87 | 109/18493 | 1.25E-05 | 0.000168293 | 9.29E-05 | SLC6A2/ADA/SLC6A3/SLC6A4/MAOB/SLC22A5 | 6 |
| BP | GO:0045600 | positive regulation of fat cell differentiation | 5/87 | 64/18493 | 1.26E-05 | 0.000168293 | 9.29E-05 | PTGS2/PPARG/PPARD/INS/AKT1 | 5 |
| BP | GO:0071242 | cellular response to ammonium ion | 5/87 | 64/18493 | 1.26E-05 | 0.000168293 | 9.29E-05 | CHRM2/GABRA1/GABRB3/GABRG2/CASP3 | 5 |
| BP | GO:1903036 | positive regulation of response to wounding | 5/87 | 64/18493 | 1.26E-05 | 0.000168293 | 9.29E-05 | F2/F7/SERPINE1/PPARD/PLG | 5 |
| BP | GO:0051402 | neuron apoptotic process | 8/87 | 230/18493 | 1.27E-05 | 0.000169609 | 9.37E-05 | SOD1/BDNF/BCL2/GABRA5/GABRB3/GRIN1/APP/CASP3 | 8 |
| BP | GO:0022407 | regulation of cell-cell adhesion | 10/87 | 388/18493 | 1.40E-05 | 0.000185703 | 0.000102554 | ADA/DPP4/ERBB2/PPARA/PLG/IL10/IL6/AKT1/CASP3/TNF | 10 |
| BP | GO:0007613 | memory | 6/87 | 112/18493 | 1.46E-05 | 0.000193126 | 0.000106654 | PTGS2/SLC6A4/BDNF/CCK/GRIN1/GRIN2A | 6 |
| BP | GO:2001233 | regulation of apoptotic signaling pathway | 10/87 | 391/18493 | 1.50E-05 | 0.000196787 | 0.000108676 | PTGS2/AR/GSK3B/SOD1/SERPINE1/INS/BCL2/RELA/AKT1/TNF | 10 |
| BP | GO:0010950 | positive regulation of endopeptidase activity | 7/87 | 171/18493 | 1.60E-05 | 0.000208915 | 0.000115373 | PPARG/CCK/GRIN1/GRIN2A/GRIN2B/CASP3/TNF | 7 |
| BP | GO:0019371 | cyclooxygenase pathway | 3/87 | 11/18493 | 1.61E-05 | 0.000208915 | 0.000115373 | PTGS2/PTGS1/PTGIS | 3 |
| BP | GO:0034776 | response to histamine | 3/87 | 11/18493 | 1.61E-05 | 0.000208915 | 0.000115373 | GABRA1/GABRB3/GABRG2 | 3 |
| BP | GO:0042368 | vitamin D biosynthetic process | 3/87 | 11/18493 | 1.61E-05 | 0.000208915 | 0.000115373 | VDR/NFKB1/TNF | 3 |
| BP | GO:0033198 | response to ATP | 4/87 | 33/18493 | 1.68E-05 | 0.000215265 | 0.00011888 | PTGS2/TRPV1/SOD1/P2RY12 | 4 |
| BP | GO:0060603 | mammary gland duct morphogenesis | 4/87 | 33/18493 | 1.68E-05 | 0.000215265 | 0.00011888 | ESR1/AR/PGR/VDR | 4 |
| BP | GO:0071354 | cellular response to interleukin-6 | 4/87 | 33/18493 | 1.68E-05 | 0.000215265 | 0.00011888 | RELA/IL6/NFKB1/PTGIS | 4 |
| BP | GO:0048167 | regulation of synaptic plasticity | 7/87 | 173/18493 | 1.73E-05 | 0.00022006 | 0.000121528 | PTGS2/INS/GRIN1/GRIN2A/GRIN2B/GRIN2D/APP | 7 |
| BP | GO:0006801 | superoxide metabolic process | 5/87 | 69/18493 | 1.82E-05 | 0.000229671 | 0.000126836 | NOS2/SOD1/MPO/CRP/TNF | 5 |
| BP | GO:0040014 | regulation of multicellular organism growth | 5/87 | 69/18493 | 1.82E-05 | 0.000229671 | 0.000126836 | SLC6A3/ADRB2/SOD1/BCL2/APP | 5 |
| BP | GO:0043062 | extracellular structure organization | 10/87 | 402/18493 | 1.90E-05 | 0.000237749 | 0.000131297 | DPP4/LPL/SERPINE1/MPO/PLG/CETP/APP/ITGB3/NFKB2/TNF | 10 |
| BP | GO:0051222 | positive regulation of protein transport | 10/87 | 402/18493 | 1.90E-05 | 0.000237749 | 0.000131297 | PTGS2/GSK3B/ERBB2/LPL/PPARD/INS/IL10/IL6/ACHE/TNF | 10 |
| BP | GO:0030072 | peptide hormone secretion | 8/87 | 244/18493 | 1.95E-05 | 0.000243152 | 0.000134281 | NOS2/DPP4/HMGCR/PPARD/INS/IL6/SLC2A1/TNF | 8 |
| BP | GO:0051047 | positive regulation of secretion | 10/87 | 404/18493 | 1.98E-05 | 0.000246102 | 0.00013591 | TRPV1/SLC6A4/LPL/PPARD/INS/CCK/IL10/IL6/ACHE/TNF | 10 |
| BP | GO:0019218 | regulation of steroid metabolic process | 6/87 | 119/18493 | 2.07E-05 | 0.000255474 | 0.000141086 | SOD1/HMGCR/SP1/VDR/NFKB1/TNF | 6 |
| BP | GO:0038034 | signal transduction in absence of ligand | 5/87 | 71/18493 | 2.09E-05 | 0.000255801 | 0.000141266 | GSK3B/BCL2/AKT1/CASP3/TNF | 5 |
| BP | GO:0061180 | mammary gland epithelium development | 5/87 | 71/18493 | 2.09E-05 | 0.000255801 | 0.000141266 | ESR1/AR/PGR/VDR/AKT1 | 5 |
| BP | GO:0097192 | extrinsic apoptotic signaling pathway in absence of ligand | 5/87 | 71/18493 | 2.09E-05 | 0.000255801 | 0.000141266 | GSK3B/BCL2/AKT1/CASP3/TNF | 5 |
| BP | GO:0032885 | regulation of polysaccharide biosynthetic process | 4/87 | 35/18493 | 2.14E-05 | 0.000259242 | 0.000143167 | GSK3B/INS/AKT1/NFKB1 | 4 |
| BP | GO:0010889 | regulation of sequestering of triglyceride | 3/87 | 12/18493 | 2.15E-05 | 0.000259242 | 0.000143167 | PPARG/LPL/PPARA | 3 |
| BP | GO:0030656 | regulation of vitamin metabolic process | 3/87 | 12/18493 | 2.15E-05 | 0.000259242 | 0.000143167 | VDR/NFKB1/TNF | 3 |
| BP | GO:0022612 | gland morphogenesis | 6/87 | 120/18493 | 2.17E-05 | 0.000260645 | 0.000143941 | ESR1/AR/PGR/BCL2/VDR/TNF | 6 |
| BP | GO:0061045 | negative regulation of wound healing | 5/87 | 72/18493 | 2.24E-05 | 0.000268592 | 0.00014833 | F2/PLAU/SERPINE1/HMGCR/PLG | 5 |
| BP | GO:0010721 | negative regulation of cell development | 9/87 | 326/18493 | 2.26E-05 | 0.000269781 | 0.000148986 | TRPV1/SLC6A4/F2/GSK3B/PPARA/BCL2/IL6/APP/TNF | 9 |
| BP | GO:0002687 | positive regulation of leukocyte migration | 6/87 | 121/18493 | 2.27E-05 | 0.000270078 | 0.000149151 | F7/SERPINE1/IL6/APP/TNF/P2RY12 | 6 |
| BP | GO:0007159 | leukocyte cell-cell adhesion | 9/87 | 327/18493 | 2.31E-05 | 0.000274251 | 0.000151455 | ADA/DPP4/ERBB2/PPARA/IL10/IL6/AKT1/CASP3/TNF | 9 |
| BP | GO:0006816 | calcium ion transport | 10/87 | 413/18493 | 2.39E-05 | 0.000279005 | 0.000154081 | PTGS2/TRPV1/F2/BCL2/VDR/GRIN1/GRIN2A/GRIN2B/GRIN2D/P2RY12 | 10 |
| BP | GO:0032637 | interleukin-8 production | 5/87 | 73/18493 | 2.40E-05 | 0.000279005 | 0.000154081 | NOS2/SERPINE1/CRP/IL10/TNF | 5 |
| BP | GO:0061138 | morphogenesis of a branching epithelium | 7/87 | 182/18493 | 2.40E-05 | 0.000279005 | 0.000154081 | ESR1/AR/PGR/BCL2/IL10/VDR/TNF | 7 |
| BP | GO:0001662 | behavioral fear response | 4/87 | 36/18493 | 2.40E-05 | 0.000279005 | 0.000154081 | DPP4/CCK/BCL2/GABRA5 | 4 |
| BP | GO:0002209 | behavioral defense response | 4/87 | 36/18493 | 2.40E-05 | 0.000279005 | 0.000154081 | DPP4/CCK/BCL2/GABRA5 | 4 |
| BP | GO:0090257 | regulation of muscle system process | 8/87 | 252/18493 | 2.46E-05 | 0.000284729 | 0.000157242 | PTGS2/CHRM2/SCN5A/ADA/ADRA1B/ADRB2/SOD1/PPARA | 8 |
| BP | GO:0072347 | response to anesthetic | 5/87 | 74/18493 | 2.56E-05 | 0.000295552 | 0.000163219 | ADA/SLC6A3/F7/RELA/GRIN1 | 5 |
| BP | GO:0007618 | mating | 4/87 | 37/18493 | 2.68E-05 | 0.000304854 | 0.000168356 | ADA/SLC6A4/GRIN1/APP | 4 |
| BP | GO:0032094 | response to food | 4/87 | 37/18493 | 2.68E-05 | 0.000304854 | 0.000168356 | MPO/PPARA/CCK/AKT1 | 4 |
| BP | GO:0070741 | response to interleukin-6 | 4/87 | 37/18493 | 2.68E-05 | 0.000304854 | 0.000168356 | RELA/IL6/NFKB1/PTGIS | 4 |
| BP | GO:1902893 | regulation of pri-miRNA transcription by RNA polymerase II | 4/87 | 37/18493 | 2.68E-05 | 0.000304854 | 0.000168356 | PPARA/PPARD/IL10/RELA | 4 |
| BP | GO:0007596 | blood coagulation | 9/87 | 334/18493 | 2.73E-05 | 0.000308614 | 0.000170432 | F2/F7/F10/PLAU/SERPINE1/PLG/IL6/ITGB3/P2RY12 | 9 |
| BP | GO:0007422 | peripheral nervous system development | 5/87 | 75/18493 | 2.73E-05 | 0.000308614 | 0.000170432 | SOD1/ERBB2/BDNF/RELA/AKT1 | 5 |
| BP | GO:0044130 | negative regulation of growth of symbiont in host | 3/87 | 13/18493 | 2.78E-05 | 0.000311618 | 0.000172091 | MPO/IL10/TNF | 3 |
| BP | GO:1902894 | negative regulation of pri-miRNA transcription by RNA polymerase II | 3/87 | 13/18493 | 2.78E-05 | 0.000311618 | 0.000172091 | PPARA/PPARD/RELA | 3 |
| BP | GO:0031668 | cellular response to extracellular stimulus | 8/87 | 258/18493 | 2.91E-05 | 0.000324722 | 0.000179328 | PTGS2/TRPV1/PPARG/SOD1/BCL2/VDR/SLC2A1/P2RY12 | 8 |
| BP | GO:0042596 | fear response | 4/87 | 38/18493 | 2.98E-05 | 0.000330236 | 0.000182373 | DPP4/CCK/BCL2/GABRA5 | 4 |
| BP | GO:0032355 | response to estradiol | 6/87 | 127/18493 | 2.99E-05 | 0.000330236 | 0.000182373 | PTGS2/SLC6A4/ESR1/F7/IL10/CASP3 | 6 |
| BP | GO:0032868 | response to insulin | 8/87 | 259/18493 | 2.99E-05 | 0.000330236 | 0.000182373 | PPARG/PPARA/INS/IL10/SP1/RELA/AKT1/SLC2A1 | 8 |
| BP | GO:0007599 | hemostasis | 9/87 | 339/18493 | 3.07E-05 | 0.000338004 | 0.000186663 | F2/F7/F10/PLAU/SERPINE1/PLG/IL6/ITGB3/P2RY12 | 9 |
| BP | GO:0030193 | regulation of blood coagulation | 5/87 | 77/18493 | 3.11E-05 | 0.000338388 | 0.000186875 | F2/F7/PLAU/SERPINE1/PLG | 5 |
| BP | GO:0060291 | long-term synaptic potentiation | 5/87 | 77/18493 | 3.11E-05 | 0.000338388 | 0.000186875 | INS/GRIN2A/GRIN2B/GRIN2D/APP | 5 |
| BP | GO:1900046 | regulation of hemostasis | 5/87 | 77/18493 | 3.11E-05 | 0.000338388 | 0.000186875 | F2/F7/PLAU/SERPINE1/PLG | 5 |
| BP | GO:0050817 | coagulation | 9/87 | 340/18493 | 3.14E-05 | 0.000340719 | 0.000188162 | F2/F7/F10/PLAU/SERPINE1/PLG/IL6/ITGB3/P2RY12 | 9 |
| BP | GO:0042180 | cellular ketone metabolic process | 7/87 | 190/18493 | 3.16E-05 | 0.000340719 | 0.000188162 | PTGS2/PPARG/HMGCR/PPARA/INS/AKR1C1/AKT1 | 7 |
| BP | GO:1901654 | response to ketone | 7/87 | 190/18493 | 3.16E-05 | 0.000340719 | 0.000188162 | MAOB/AR/PPARG/F7/NR3C1/RELA/AKT1 | 7 |
| BP | GO:0031349 | positive regulation of defense response | 10/87 | 427/18493 | 3.18E-05 | 0.000341322 | 0.000188495 | PTGS2/ESR1/LPL/SERPINE1/CCK/RELA/IL6/APP/NFKB1/TNF | 10 |
| BP | GO:0051930 | regulation of sensory perception of pain | 4/87 | 39/18493 | 3.31E-05 | 0.000354849 | 0.000195966 | CCK/IL10/GRIN2A/GRIN2D | 4 |
| BP | GO:0019372 | lipoxygenase pathway | 3/87 | 14/18493 | 3.53E-05 | 0.000369885 | 0.000204269 | PTGS2/PON1/ALOX5 | 3 |
| BP | GO:0042362 | fat-soluble vitamin biosynthetic process | 3/87 | 14/18493 | 3.53E-05 | 0.000369885 | 0.000204269 | VDR/NFKB1/TNF | 3 |
| BP | GO:0044126 | regulation of growth of symbiont in host | 3/87 | 14/18493 | 3.53E-05 | 0.000369885 | 0.000204269 | MPO/IL10/TNF | 3 |
| BP | GO:0044146 | negative regulation of growth of symbiont involved in interaction with host | 3/87 | 14/18493 | 3.53E-05 | 0.000369885 | 0.000204269 | MPO/IL10/TNF | 3 |
| BP | GO:0051917 | regulation of fibrinolysis | 3/87 | 14/18493 | 3.53E-05 | 0.000369885 | 0.000204269 | F2/SERPINE1/PLG | 3 |
| BP | GO:0150078 | NA | 3/87 | 14/18493 | 3.53E-05 | 0.000369885 | 0.000204269 | IL6/APP/TNF | 3 |
| BP | GO:0032881 | regulation of polysaccharide metabolic process | 4/87 | 40/18493 | 3.67E-05 | 0.00038232 | 0.000211136 | GSK3B/INS/AKT1/NFKB1 | 4 |
| BP | GO:0051931 | regulation of sensory perception | 4/87 | 40/18493 | 3.67E-05 | 0.00038232 | 0.000211136 | CCK/IL10/GRIN2A/GRIN2D | 4 |
| BP | GO:0045927 | positive regulation of growth | 8/87 | 267/18493 | 3.71E-05 | 0.000384117 | 0.000212128 | SLC6A3/F2/ERBB2/BDNF/PPARD/INS/BCL2/AKT1 | 8 |
| BP | GO:0097553 | calcium ion transmembrane import into cytosol | 6/87 | 132/18493 | 3.71E-05 | 0.000384117 | 0.000212128 | TRPV1/F2/GRIN1/GRIN2A/GRIN2B/GRIN2D | 6 |
| BP | GO:0035265 | organ growth | 7/87 | 195/18493 | 3.73E-05 | 0.000384802 | 0.000212507 | SLC6A4/ESR1/AR/SOD1/PPARA/BCL2/AKT1 | 7 |
| BP | GO:0006836 | neurotransmitter transport | 8/87 | 268/18493 | 3.81E-05 | 0.000391697 | 0.000216315 | CHRM2/SLC6A2/ADA/SLC6A3/SLC6A4/MAOB/GSK3B/GABRQ | 8 |
| BP | GO:0001763 | morphogenesis of a branching structure | 7/87 | 196/18493 | 3.85E-05 | 0.000394923 | 0.000218096 | ESR1/AR/PGR/BCL2/IL10/VDR/TNF | 7 |
| BP | GO:0050729 | positive regulation of inflammatory response | 6/87 | 133/18493 | 3.87E-05 | 0.000395487 | 0.000218408 | PTGS2/LPL/SERPINE1/IL6/APP/TNF | 6 |
| BP | GO:0006936 | muscle contraction | 9/87 | 351/18493 | 4.03E-05 | 0.000409802 | 0.000226313 | PTGS2/CHRM2/TRPV1/SCN5A/ADA/ADRA1B/ADRB2/KCNH2/SOD1 | 9 |
| BP | GO:0007586 | digestion | 6/87 | 135/18493 | 4.21E-05 | 0.000425901 | 0.000235204 | TRPV1/PRSS3/CCK/SLC22A5/AKR1C1/VDR | 6 |
| BP | GO:0050818 | regulation of coagulation | 5/87 | 82/18493 | 4.21E-05 | 0.000425901 | 0.000235204 | F2/F7/PLAU/SERPINE1/PLG | 5 |
| BP | GO:0009314 | response to radiation | 10/87 | 443/18493 | 4.33E-05 | 0.000436955 | 0.000241308 | PTGS2/HMGCR/CCK/BCL2/RELA/GRIN1/GRIN2A/AKT1/APP/CASP3 | 10 |
| BP | GO:0014065 | phosphatidylinositol 3-kinase signaling | 6/87 | 136/18493 | 4.39E-05 | 0.000439974 | 0.000242976 | F2/ERBB2/PPARD/INS/AKT1/TNF | 6 |
| BP | GO:0044144 | modulation of growth of symbiont involved in interaction with host | 3/87 | 15/18493 | 4.39E-05 | 0.000439974 | 0.000242976 | MPO/IL10/TNF | 3 |
| BP | GO:1901653 | cellular response to peptide | 9/87 | 356/18493 | 4.49E-05 | 0.000448494 | 0.000247681 | ADRB2/PPARG/GSK3B/INS/SP1/RELA/AKT1/APP/NFKB1 | 9 |
| BP | GO:0043523 | regulation of neuron apoptotic process | 7/87 | 202/18493 | 4.67E-05 | 0.000464545 | 0.000256545 | SOD1/BDNF/BCL2/GABRA5/GABRB3/GRIN1/CASP3 | 7 |
| BP | GO:0010038 | response to metal ion | 9/87 | 358/18493 | 4.69E-05 | 0.000465381 | 0.000257007 | PTGS2/SCN5A/SLC6A3/MAOB/SOD1/BCL2/AKT1/APP/CASP3 | 9 |
| BP | GO:0042446 | hormone biosynthetic process | 5/87 | 84/18493 | 4.73E-05 | 0.0004662 | 0.000257459 | VDR/HSD17B1/HSD11B1/NFKB1/TNF | 5 |
| BP | GO:1903035 | negative regulation of response to wounding | 5/87 | 84/18493 | 4.73E-05 | 0.0004662 | 0.000257459 | F2/PLAU/SERPINE1/HMGCR/PLG | 5 |
| BP | GO:0050679 | positive regulation of epithelial cell proliferation | 7/87 | 203/18493 | 4.82E-05 | 0.00047176 | 0.00026053 | SCN5A/AR/ERBB2/IL10/SP1/AKT1/ITGB3 | 7 |
| BP | GO:0090276 | regulation of peptide hormone secretion | 7/87 | 203/18493 | 4.82E-05 | 0.00047176 | 0.00026053 | NOS2/DPP4/HMGCR/PPARD/INS/SLC2A1/TNF | 7 |
| BP | GO:0032965 | regulation of collagen biosynthetic process | 4/87 | 43/18493 | 4.90E-05 | 0.000478629 | 0.000264323 | F2/PPARG/PPARD/IL6 | 4 |
| BP | GO:0009612 | response to mechanical stimulus | 7/87 | 204/18493 | 4.97E-05 | 0.000483628 | 0.000267084 | PTGS2/PPARG/MPO/RELA/AKT1/SLC2A1/NFKB1 | 7 |
| BP | GO:0045833 | negative regulation of lipid metabolic process | 5/87 | 85/18493 | 5.01E-05 | 0.000485805 | 0.000268286 | SOD1/INS/AKT1/NFKB1/TNF | 5 |
| BP | GO:0042110 | T cell activation | 10/87 | 451/18493 | 5.04E-05 | 0.000487245 | 0.000269081 | ADA/DPP4/SOD1/ERBB2/INS/BCL2/IL10/IL6/AKT1/CASP3 | 10 |
| BP | GO:0023061 | signal release | 10/87 | 453/18493 | 5.23E-05 | 0.000504072 | 0.000278374 | CHRM2/NOS2/DPP4/GSK3B/HMGCR/PPARD/INS/IL6/SLC2A1/TNF | 10 |
| BP | GO:0030879 | mammary gland development | 6/87 | 141/18493 | 5.37E-05 | 0.000514758 | 0.000284275 | SLC6A3/ESR1/AR/PGR/VDR/AKT1 | 6 |
| BP | GO:0045986 | negative regulation of smooth muscle contraction | 3/87 | 16/18493 | 5.39E-05 | 0.000514758 | 0.000284275 | PTGS2/ADRB2/SOD1 | 3 |
| BP | GO:0048521 | negative regulation of behavior | 3/87 | 16/18493 | 5.39E-05 | 0.000514758 | 0.000284275 | ADA/INS/CCK | 3 |
| BP | GO:0018105 | peptidyl-serine phosphorylation | 8/87 | 282/18493 | 5.46E-05 | 0.000519797 | 0.000287058 | PTGS2/HSP90AA1/GSK3B/BCL2/IL6/AKT1/APP/TNF | 8 |
| BP | GO:0003012 | muscle system process | 10/87 | 456/18493 | 5.52E-05 | 0.000524546 | 0.000289681 | PTGS2/CHRM2/TRPV1/SCN5A/ADA/ADRA1B/ADRB2/KCNH2/SOD1/PPARA | 10 |
| BP | GO:1900407 | regulation of cellular response to oxidative stress | 5/87 | 87/18493 | 5.60E-05 | 0.000529977 | 0.00029268 | SOD1/INS/IL10/AKT1/TNF | 5 |
| BP | GO:0070838 | divalent metal ion transport | 10/87 | 459/18493 | 5.84E-05 | 0.000550714 | 0.000304132 | PTGS2/TRPV1/F2/BCL2/VDR/GRIN1/GRIN2A/GRIN2B/GRIN2D/P2RY12 | 10 |
| BP | GO:0008631 | intrinsic apoptotic signaling pathway in response to oxidative stress | 4/87 | 45/18493 | 5.88E-05 | 0.000551431 | 0.000304528 | SOD1/INS/BCL2/AKT1 | 4 |
| BP | GO:0001776 | leukocyte homeostasis | 5/87 | 88/18493 | 5.91E-05 | 0.000551431 | 0.000304528 | ADA/BCL2/IL6/AKT1/CASP3 | 5 |
| BP | GO:0015844 | monoamine transport | 5/87 | 88/18493 | 5.91E-05 | 0.000551431 | 0.000304528 | SLC6A2/SLC6A3/SLC6A4/MAOB/P2RY12 | 5 |
| BP | GO:0019217 | regulation of fatty acid metabolic process | 5/87 | 88/18493 | 5.91E-05 | 0.000551431 | 0.000304528 | PTGS2/PPARG/PPARA/INS/AKT1 | 5 |
| BP | GO:0015711 | organic anion transport | 10/87 | 460/18493 | 5.94E-05 | 0.000552413 | 0.00030507 | TRPV1/NOS2/PPARG/PPARA/PPARD/CCK/CETP/AKR1C1/AKT1/SLC2A1 | 10 |
| BP | GO:0072511 | divalent inorganic cation transport | 10/87 | 462/18493 | 6.16E-05 | 0.000571057 | 0.000315366 | PTGS2/TRPV1/F2/BCL2/VDR/GRIN1/GRIN2A/GRIN2B/GRIN2D/P2RY12 | 10 |
| BP | GO:0009408 | response to heat | 6/87 | 145/18493 | 6.28E-05 | 0.00057998 | 0.000320294 | PTGS2/TRPV1/HSP90AA1/GSK3B/SOD1/AKT1 | 6 |
| BP | GO:0060443 | mammary gland morphogenesis | 4/87 | 46/18493 | 6.41E-05 | 0.000590825 | 0.000326283 | ESR1/AR/PGR/VDR | 4 |
| BP | GO:1903532 | positive regulation of secretion by cell | 9/87 | 373/18493 | 6.44E-05 | 0.000591314 | 0.000326553 | SLC6A4/LPL/PPARD/INS/CCK/IL10/IL6/ACHE/TNF | 9 |
| BP | GO:0032966 | negative regulation of collagen biosynthetic process | 3/87 | 17/18493 | 6.52E-05 | 0.000595456 | 0.000328841 | PPARG/PPARD/IL6 | 3 |
| BP | GO:0044117 | growth of symbiont in host | 3/87 | 17/18493 | 6.52E-05 | 0.000595456 | 0.000328841 | MPO/IL10/TNF | 3 |
| BP | GO:0014909 | smooth muscle cell migration | 5/87 | 90/18493 | 6.59E-05 | 0.000599675 | 0.000331171 | PLAU/SERPINE1/PPARD/BCL2/ITGB3 | 5 |
| BP | GO:0042176 | regulation of protein catabolic process | 9/87 | 375/18493 | 6.71E-05 | 0.000608428 | 0.000336005 | HSP90AA1/NOS2/GSK3B/INS/IL10/RELA/GRIN2A/AKT1/TNF | 9 |
| BP | GO:0050807 | regulation of synapse organization | 7/87 | 214/18493 | 6.72E-05 | 0.000608428 | 0.000336005 | BDNF/INS/IL10/GRIN1/GRIN2B/APP/TNF | 7 |
| BP | GO:0035264 | multicellular organism growth | 6/87 | 147/18493 | 6.77E-05 | 0.000609544 | 0.000336621 | SLC6A3/ADRB2/AR/SOD1/BCL2/APP | 6 |
| BP | GO:0043524 | negative regulation of neuron apoptotic process | 6/87 | 147/18493 | 6.77E-05 | 0.000609544 | 0.000336621 | SOD1/BDNF/BCL2/GABRA5/GABRB3/GRIN1 | 6 |
| BP | GO:0010959 | regulation of metal ion transport | 9/87 | 376/18493 | 6.84E-05 | 0.000614247 | 0.000339218 | PTGS2/SCN5A/ADRB2/F2/KCNH2/BCL2/GRIN1/AKT1/P2RY12 | 9 |
| BP | GO:0150076 | NA | 4/87 | 47/18493 | 6.98E-05 | 0.000624932 | 0.000345119 | TRPV1/IL6/APP/TNF | 4 |
| BP | GO:0048871 | multicellular organismal homeostasis | 10/87 | 471/18493 | 7.23E-05 | 0.000645413 | 0.00035643 | PTGS2/TRPV1/ADRB2/SOD1/BCL2/SLC22A5/IL6/ACHE/ITGB3/TNF | 10 |
| BP | GO:0002673 | regulation of acute inflammatory response | 6/87 | 149/18493 | 7.30E-05 | 0.000648956 | 0.000358386 | PTGS2/F2/PPARG/INS/IL6/TNF | 6 |
| BP | GO:0043255 | regulation of carbohydrate biosynthetic process | 5/87 | 92/18493 | 7.32E-05 | 0.000648956 | 0.000358386 | GSK3B/PPARA/INS/AKT1/NFKB1 | 5 |
| BP | GO:0048754 | branching morphogenesis of an epithelial tube | 6/87 | 150/18493 | 7.58E-05 | 0.000667761 | 0.000368771 | ESR1/AR/PGR/BCL2/VDR/TNF | 6 |
| BP | GO:0009409 | response to cold | 4/87 | 48/18493 | 7.59E-05 | 0.000667761 | 0.000368771 | HSP90AA1/ADRB2/PPARG/LPL | 4 |
| BP | GO:0010712 | regulation of collagen metabolic process | 4/87 | 48/18493 | 7.59E-05 | 0.000667761 | 0.000368771 | F2/PPARG/PPARD/IL6 | 4 |
| BP | GO:0015908 | fatty acid transport | 5/87 | 93/18493 | 7.70E-05 | 0.000673684 | 0.000372042 | NOS2/PPARG/PPARA/PPARD/AKT1 | 5 |
| BP | GO:0036473 | cell death in response to oxidative stress | 5/87 | 93/18493 | 7.70E-05 | 0.000673684 | 0.000372042 | SOD1/INS/BCL2/IL10/AKT1 | 5 |
| BP | GO:0010713 | negative regulation of collagen metabolic process | 3/87 | 18/18493 | 7.80E-05 | 0.00067443 | 0.000372454 | PPARG/PPARD/IL6 | 3 |
| BP | GO:0044110 | growth involved in symbiotic interaction | 3/87 | 18/18493 | 7.80E-05 | 0.00067443 | 0.000372454 | MPO/IL10/TNF | 3 |
| BP | GO:0044116 | growth of symbiont involved in interaction with host | 3/87 | 18/18493 | 7.80E-05 | 0.00067443 | 0.000372454 | MPO/IL10/TNF | 3 |
| BP | GO:1900273 | positive regulation of long-term synaptic potentiation | 3/87 | 18/18493 | 7.80E-05 | 0.00067443 | 0.000372454 | INS/GRIN2A/APP | 3 |
| BP | GO:0050803 | regulation of synapse structure or activity | 7/87 | 220/18493 | 8.00E-05 | 0.000689605 | 0.000380835 | BDNF/INS/IL10/GRIN1/GRIN2B/APP/TNF | 7 |
| BP | GO:0050808 | synapse organization | 9/87 | 384/18493 | 8.04E-05 | 0.000691571 | 0.00038192 | ERBB2/BDNF/INS/IL10/GRIN1/GRIN2B/ACHE/APP/TNF | 9 |
| BP | GO:0042035 | regulation of cytokine biosynthetic process | 5/87 | 94/18493 | 8.11E-05 | 0.000695208 | 0.000383929 | IL10/RELA/IL6/NFKB1/TNF | 5 |
| BP | GO:0050900 | leukocyte migration | 10/87 | 478/18493 | 8.17E-05 | 0.000699189 | 0.000386127 | ADA/F7/SERPINE1/IL10/IL6/AKT1/APP/ITGB3/TNF/P2RY12 | 10 |
| BP | GO:0046456 | icosanoid biosynthetic process | 4/87 | 49/18493 | 8.24E-05 | 0.000700699 | 0.000386961 | PTGS2/PTGS1/ALOX5/PTGIS | 4 |
| BP | GO:1990090 | cellular response to nerve growth factor stimulus | 4/87 | 49/18493 | 8.24E-05 | 0.000700699 | 0.000386961 | TRPV1/BDNF/AKT1/APP | 4 |
| BP | GO:0051048 | negative regulation of secretion | 7/87 | 222/18493 | 8.46E-05 | 0.000717848 | 0.000396432 | ADA/MAOB/HMGCR/INS/IL10/TNF/P2RY12 | 7 |
| BP | GO:0018209 | peptidyl-serine modification | 8/87 | 301/18493 | 8.61E-05 | 0.000728332 | 0.000402221 | PTGS2/HSP90AA1/GSK3B/BCL2/IL6/AKT1/APP/TNF | 8 |
| BP | GO:0043271 | negative regulation of ion transport | 6/87 | 154/18493 | 8.76E-05 | 0.000739406 | 0.000408337 | PTGS2/MAOB/KCNH2/BCL2/GABRE/AKT1 | 6 |
| BP | GO:1902882 | regulation of response to oxidative stress | 5/87 | 96/18493 | 8.96E-05 | 0.000753893 | 0.000416338 | SOD1/INS/IL10/AKT1/TNF | 5 |
| BP | GO:2001236 | regulation of extrinsic apoptotic signaling pathway | 6/87 | 156/18493 | 9.41E-05 | 0.000785398 | 0.000433736 | AR/SERPINE1/BCL2/RELA/AKT1/TNF | 6 |
| BP | GO:0007200 | phospholipase C-activating G-protein coupled receptor signaling pathway | 5/87 | 97/18493 | 9.41E-05 | 0.000785398 | 0.000433736 | CHRM2/ADRA1B/F2/ESR1/P2RY12 | 5 |
| BP | GO:0062014 | negative regulation of small molecule metabolic process | 5/87 | 97/18493 | 9.41E-05 | 0.000785398 | 0.000433736 | SOD1/PPARA/INS/AKT1/NFKB1 | 5 |
| BP | GO:0050863 | regulation of T cell activation | 8/87 | 305/18493 | 9.43E-05 | 0.000785398 | 0.000433736 | ADA/DPP4/SOD1/ERBB2/IL10/IL6/AKT1/CASP3 | 8 |
| BP | GO:0046879 | hormone secretion | 8/87 | 306/18493 | 9.65E-05 | 0.000801275 | 0.000442504 | NOS2/DPP4/HMGCR/PPARD/INS/IL6/SLC2A1/TNF | 8 |
| BP | GO:0008585 | female gonad development | 5/87 | 98/18493 | 9.88E-05 | 0.000815977 | 0.000450624 | ESR1/PGR/SOD1/BCL2/CASP3 | 5 |
| BP | GO:0030301 | cholesterol transport | 5/87 | 98/18493 | 9.88E-05 | 0.000815977 | 0.000450624 | PPARG/PON1/CETP/AKR1C1/NFKB1 | 5 |
| BP | GO:0015718 | monocarboxylic acid transport | 6/87 | 158/18493 | 0.000100945 | 0.000829189 | 0.00045792 | NOS2/PPARG/PPARA/PPARD/AKR1C1/AKT1 | 6 |
| BP | GO:0046165 | alcohol biosynthetic process | 6/87 | 158/18493 | 0.000100945 | 0.000829189 | 0.00045792 | SOD1/HMGCR/SP1/VDR/NFKB1/TNF | 6 |
| BP | GO:0045785 | positive regulation of cell adhesion | 9/87 | 397/18493 | 0.000103573 | 0.000835403 | 0.000461351 | ADA/DPP4/GSK3B/ERBB2/IL10/IL6/AKT1/TNF/P2RY12 | 9 |
| BP | GO:0007566 | embryo implantation | 4/87 | 52/18493 | 0.000104123 | 0.000835403 | 0.000461351 | PTGS2/SOD1/PPARD/PTGIS | 4 |
| BP | GO:0030195 | negative regulation of blood coagulation | 4/87 | 52/18493 | 0.000104123 | 0.000835403 | 0.000461351 | F2/PLAU/SERPINE1/PLG | 4 |
| BP | GO:0031638 | zymogen activation | 4/87 | 52/18493 | 0.000104123 | 0.000835403 | 0.000461351 | PRSS3/PLAU/SERPINE1/GRIN2A | 4 |
| BP | GO:0032964 | collagen biosynthetic process | 4/87 | 52/18493 | 0.000104123 | 0.000835403 | 0.000461351 | F2/PPARG/PPARD/IL6 | 4 |
| BP | GO:0050994 | regulation of lipid catabolic process | 4/87 | 52/18493 | 0.000104123 | 0.000835403 | 0.000461351 | PPARA/INS/AKT1/TNF | 4 |
| BP | GO:0051353 | positive regulation of oxidoreductase activity | 4/87 | 52/18493 | 0.000104123 | 0.000835403 | 0.000461351 | INS/VDR/AKT1/TNF | 4 |
| BP | GO:1900047 | negative regulation of hemostasis | 4/87 | 52/18493 | 0.000104123 | 0.000835403 | 0.000461351 | F2/PLAU/SERPINE1/PLG | 4 |
| BP | GO:1990089 | response to nerve growth factor | 4/87 | 52/18493 | 0.000104123 | 0.000835403 | 0.000461351 | TRPV1/BDNF/AKT1/APP | 4 |
| BP | GO:0010869 | regulation of receptor biosynthetic process | 3/87 | 20/18493 | 0.0001082 | 0.000865882 | 0.000478183 | PPARG/PPARA/ITGB3 | 3 |
| BP | GO:0007631 | feeding behavior | 5/87 | 101/18493 | 0.000113984 | 0.000905165 | 0.000499878 | INS/CCK/PYY/GRIN1/APP | 5 |
| BP | GO:0098869 | cellular oxidant detoxification | 5/87 | 101/18493 | 0.000113984 | 0.000905165 | 0.000499878 | PTGS2/PTGS1/SOD1/MPO/TNF | 5 |
| BP | GO:1904659 | glucose transmembrane transport | 5/87 | 101/18493 | 0.000113984 | 0.000905165 | 0.000499878 | PPARD/INS/AKT1/SLC2A1/TNF | 5 |
| BP | GO:0030307 | positive regulation of cell growth | 6/87 | 163/18493 | 0.000119795 | 0.000947449 | 0.000523229 | F2/ERBB2/BDNF/INS/BCL2/AKT1 | 6 |
| BP | GO:0009914 | hormone transport | 8/87 | 316/18493 | 0.000120529 | 0.000947449 | 0.000523229 | NOS2/DPP4/HMGCR/PPARD/INS/IL6/SLC2A1/TNF | 8 |
| BP | GO:0015849 | organic acid transport | 8/87 | 316/18493 | 0.000120529 | 0.000947449 | 0.000523229 | TRPV1/NOS2/PPARG/PPARA/PPARD/CCK/AKR1C1/AKT1 | 8 |
| BP | GO:0046942 | carboxylic acid transport | 8/87 | 316/18493 | 0.000120529 | 0.000947449 | 0.000523229 | TRPV1/NOS2/PPARG/PPARA/PPARD/CCK/AKR1C1/AKT1 | 8 |
| BP | GO:0048015 | phosphatidylinositol-mediated signaling | 6/87 | 164/18493 | 0.00012388 | 0.000971328 | 0.000536416 | F2/ERBB2/PPARD/INS/AKT1/TNF | 6 |
| BP | GO:0014812 | muscle cell migration | 5/87 | 103/18493 | 0.000125051 | 0.00097314 | 0.000537417 | PLAU/SERPINE1/PPARD/BCL2/ITGB3 | 5 |
| BP | GO:0032091 | negative regulation of protein binding | 5/87 | 103/18493 | 0.000125051 | 0.00097314 | 0.000537417 | ADRB2/GSK3B/PPARA/IL10/AKT1 | 5 |
| BP | GO:0046545 | development of primary female sexual characteristics | 5/87 | 103/18493 | 0.000125051 | 0.00097314 | 0.000537417 | ESR1/PGR/SOD1/BCL2/CASP3 | 5 |
| BP | GO:0042359 | vitamin D metabolic process | 3/87 | 21/18493 | 0.000125805 | 0.000974707 | 0.000538282 | VDR/NFKB1/TNF | 3 |
| BP | GO:1903829 | positive regulation of cellular protein localization | 8/87 | 318/18493 | 0.00012588 | 0.000974707 | 0.000538282 | PTGS2/F2/GSK3B/ERBB2/INS/BCL2/AKT1/TNF | 8 |
| BP | GO:0042742 | defense response to bacterium | 8/87 | 319/18493 | 0.000128628 | 0.00099351 | 0.000548666 | NOS2/F2/SERPINE1/MPO/CRP/IL10/IL6/TNF | 8 |
| BP | GO:0048017 | inositol lipid-mediated signaling | 6/87 | 167/18493 | 0.000136802 | 0.001044589 | 0.000576874 | F2/ERBB2/PPARD/INS/AKT1/TNF | 6 |
| BP | GO:0042089 | cytokine biosynthetic process | 5/87 | 105/18493 | 0.000136924 | 0.001044589 | 0.000576874 | IL10/RELA/IL6/NFKB1/TNF | 5 |
| BP | GO:0043467 | regulation of generation of precursor metabolites and energy | 5/87 | 105/18493 | 0.000136924 | 0.001044589 | 0.000576874 | NOS2/GSK3B/PPARA/INS/AKT1 | 5 |
| BP | GO:0050905 | neuromuscular process | 5/87 | 105/18493 | 0.000136924 | 0.001044589 | 0.000576874 | SLC6A3/GRIN1/GRIN2A/GRIN2D/APP | 5 |
| BP | GO:1990748 | cellular detoxification | 5/87 | 105/18493 | 0.000136924 | 0.001044589 | 0.000576874 | PTGS2/PTGS1/SOD1/MPO/TNF | 5 |
| BP | GO:0050819 | negative regulation of coagulation | 4/87 | 56/18493 | 0.000139255 | 0.001059769 | 0.000585258 | F2/PLAU/SERPINE1/PLG | 4 |
| BP | GO:0042107 | cytokine metabolic process | 5/87 | 106/18493 | 0.000143174 | 0.001086934 | 0.000600259 | IL10/RELA/IL6/NFKB1/TNF | 5 |
| BP | GO:0050995 | negative regulation of lipid catabolic process | 3/87 | 22/18493 | 0.000145174 | 0.00109943 | 0.00060716 | INS/AKT1/TNF | 3 |
| BP | GO:0097755 | positive regulation of blood vessel diameter | 4/87 | 57/18493 | 0.00014923 | 0.001127392 | 0.000622602 | ADRB2/SOD1/PPARD/INS | 4 |
| BP | GO:0071887 | leukocyte apoptotic process | 5/87 | 107/18493 | 0.000149641 | 0.001127756 | 0.000622804 | ADA/IL10/IL6/AKT1/CASP3 | 5 |
| BP | GO:0022408 | negative regulation of cell-cell adhesion | 6/87 | 170/18493 | 0.000150771 | 0.00113352 | 0.000625986 | ERBB2/PPARA/PLG/IL10/AKT1/CASP3 | 6 |
| BP | GO:0006690 | icosanoid metabolic process | 5/87 | 108/18493 | 0.000156329 | 0.001166831 | 0.000644383 | PTGS2/PTGS1/PON1/ALOX5/PTGIS | 5 |
| BP | GO:0008645 | hexose transmembrane transport | 5/87 | 108/18493 | 0.000156329 | 0.001166831 | 0.000644383 | PPARD/INS/AKT1/SLC2A1/TNF | 5 |
| BP | GO:0015918 | sterol transport | 5/87 | 108/18493 | 0.000156329 | 0.001166831 | 0.000644383 | PPARG/PON1/CETP/AKR1C1/NFKB1 | 5 |
| BP | GO:2000378 | negative regulation of reactive oxygen species metabolic process | 4/87 | 58/18493 | 0.000159711 | 0.001188824 | 0.000656528 | INS/BCL2/IL10/PTGIS | 4 |
| BP | GO:0050714 | positive regulation of protein secretion | 7/87 | 246/18493 | 0.000160041 | 0.001188824 | 0.000656528 | LPL/PPARD/INS/IL10/IL6/ACHE/TNF | 7 |
| BP | GO:0050796 | regulation of insulin secretion | 6/87 | 172/18493 | 0.000160695 | 0.001190829 | 0.000657636 | NOS2/DPP4/HMGCR/PPARD/SLC2A1/TNF | 6 |
| BP | GO:0009110 | vitamin biosynthetic process | 3/87 | 23/18493 | 0.000166384 | 0.00123005 | 0.000679295 | VDR/NFKB1/TNF | 3 |
| BP | GO:0015749 | monosaccharide transmembrane transport | 5/87 | 110/18493 | 0.000170389 | 0.001253122 | 0.000692037 | PPARD/INS/AKT1/SLC2A1/TNF | 5 |
| BP | GO:0030888 | regulation of B cell proliferation | 4/87 | 59/18493 | 0.000170715 | 0.001253122 | 0.000692037 | ADA/BCL2/IL10/CASP3 | 4 |
| BP | GO:0033619 | membrane protein proteolysis | 4/87 | 59/18493 | 0.000170715 | 0.001253122 | 0.000692037 | IL10/RELA/NFKB1/TNF | 4 |
| BP | GO:0001936 | regulation of endothelial cell proliferation | 6/87 | 175/18493 | 0.000176543 | 0.001292847 | 0.000713975 | PPARG/IL10/SP1/AKT1/ITGB3/TNF | 6 |
| BP | GO:0043200 | response to amino acid | 5/87 | 111/18493 | 0.000177771 | 0.001298776 | 0.000717249 | F7/RELA/GRIN1/CASP3/TNF | 5 |
| BP | GO:1901617 | organic hydroxy compound biosynthetic process | 7/87 | 251/18493 | 0.000181115 | 0.001320096 | 0.000729023 | SLC6A3/SOD1/HMGCR/SP1/VDR/NFKB1/TNF | 7 |
| BP | GO:0034219 | carbohydrate transmembrane transport | 5/87 | 112/18493 | 0.000185395 | 0.001348132 | 0.000744506 | PPARD/INS/AKT1/SLC2A1/TNF | 5 |
| BP | GO:1905475 | regulation of protein localization to membrane | 6/87 | 177/18493 | 0.000187778 | 0.001362268 | 0.000752313 | AR/ERBB2/INS/BCL2/AKT1/TNF | 6 |
| BP | GO:0001516 | prostaglandin biosynthetic process | 3/87 | 24/18493 | 0.000189507 | 0.001365243 | 0.000753956 | PTGS2/PTGS1/PTGIS | 3 |
| BP | GO:0033081 | regulation of T cell differentiation in thymus | 3/87 | 24/18493 | 0.000189507 | 0.001365243 | 0.000753956 | ADA/SOD1/ERBB2 | 3 |
| BP | GO:0046457 | prostanoid biosynthetic process | 3/87 | 24/18493 | 0.000189507 | 0.001365243 | 0.000753956 | PTGS2/PTGS1/PTGIS | 3 |
| BP | GO:0032371 | regulation of sterol transport | 4/87 | 61/18493 | 0.00019435 | 0.001393663 | 0.000769651 | PPARG/PON1/CETP/NFKB1 | 4 |
| BP | GO:0032374 | regulation of cholesterol transport | 4/87 | 61/18493 | 0.00019435 | 0.001393663 | 0.000769651 | PPARG/PON1/CETP/NFKB1 | 4 |
| BP | GO:0045685 | regulation of glial cell differentiation | 4/87 | 62/18493 | 0.000207012 | 0.001481041 | 0.000817905 | F2/PPARG/RELA/APP | 4 |
| BP | GO:0034612 | response to tumor necrosis factor | 7/87 | 257/18493 | 0.000209311 | 0.00149405 | 0.000825089 | PTGS2/TRPV1/RELA/AKT1/CASP3/NFKB1/TNF | 7 |
| BP | GO:0019430 | removal of superoxide radicals | 3/87 | 25/18493 | 0.000214618 | 0.001521437 | 0.000840214 | SOD1/MPO/TNF | 3 |
| BP | GO:0046885 | regulation of hormone biosynthetic process | 3/87 | 25/18493 | 0.000214618 | 0.001521437 | 0.000840214 | VDR/NFKB1/TNF | 3 |
| BP | GO:0150077 | NA | 3/87 | 25/18493 | 0.000214618 | 0.001521437 | 0.000840214 | IL6/APP/TNF | 3 |
| BP | GO:0010631 | epithelial cell migration | 8/87 | 346/18493 | 0.000223919 | 0.001583758 | 0.00087463 | PTGS2/PPARG/DPP4/PRSS3/PPARD/SP1/AKT1/ITGB3 | 8 |
| BP | GO:0046883 | regulation of hormone secretion | 7/87 | 261/18493 | 0.000230006 | 0.001623109 | 0.000896362 | NOS2/DPP4/HMGCR/PPARD/INS/SLC2A1/TNF | 7 |
| BP | GO:0043112 | receptor metabolic process | 6/87 | 184/18493 | 0.000231621 | 0.001630801 | 0.00090061 | PPARG/PPARA/IL10/ACHE/ITGB3/TNF | 6 |
| BP | GO:0002260 | lymphocyte homeostasis | 4/87 | 64/18493 | 0.000234103 | 0.001644549 | 0.000908202 | ADA/BCL2/AKT1/CASP3 | 4 |
| BP | GO:1903522 | regulation of blood circulation | 7/87 | 262/18493 | 0.000235429 | 0.00165013 | 0.000911285 | PTGS2/CHRM2/SCN5A/ADA/ADRA1B/KCNH2/AKT1 | 7 |
| BP | GO:0046660 | female sex differentiation | 5/87 | 118/18493 | 0.000236518 | 0.001654025 | 0.000913436 | ESR1/PGR/SOD1/BCL2/CASP3 | 5 |
| BP | GO:0090132 | epithelium migration | 8/87 | 349/18493 | 0.00023738 | 0.001656325 | 0.000914706 | PTGS2/PPARG/DPP4/PRSS3/PPARD/SP1/AKT1/ITGB3 | 8 |
| BP | GO:0002793 | positive regulation of peptide secretion | 7/87 | 265/18493 | 0.000252322 | 0.001755437 | 0.00096944 | LPL/PPARD/INS/IL10/IL6/ACHE/TNF | 7 |
| BP | GO:0071453 | cellular response to oxygen levels | 6/87 | 187/18493 | 0.000252715 | 0.001755437 | 0.00096944 | PTGS2/PPARG/PPARD/BCL2/AKT1/PTGIS | 6 |
| BP | GO:0038061 | NIK/NF-kappaB signaling | 5/87 | 120/18493 | 0.000255739 | 0.001772476 | 0.00097885 | RELA/AKT1/APP/NFKB2/TNF | 5 |
| BP | GO:0043542 | endothelial cell migration | 7/87 | 266/18493 | 0.000258165 | 0.001785304 | 0.000985935 | PTGS2/PPARG/DPP4/PRSS3/SP1/AKT1/ITGB3 | 7 |
| BP | GO:0032677 | regulation of interleukin-8 production | 4/87 | 66/18493 | 0.000263657 | 0.001819232 | 0.001004671 | SERPINE1/CRP/IL10/TNF | 4 |
| BP | GO:1900180 | regulation of protein localization to nucleus | 5/87 | 121/18493 | 0.000265784 | 0.001829324 | 0.001010245 | PTGS2/F2/GSK3B/INS/AKT1 | 5 |
| BP | GO:0090130 | tissue migration | 8/87 | 355/18493 | 0.000266298 | 0.001829324 | 0.001010245 | PTGS2/PPARG/DPP4/PRSS3/PPARD/SP1/AKT1/ITGB3 | 8 |
| BP | GO:0045932 | negative regulation of muscle contraction | 3/87 | 27/18493 | 0.000271089 | 0.001845905 | 0.001019401 | PTGS2/ADRB2/SOD1 | 3 |
| BP | GO:0071450 | cellular response to oxygen radical | 3/87 | 27/18493 | 0.000271089 | 0.001845905 | 0.001019401 | SOD1/MPO/TNF | 3 |
| BP | GO:0071451 | cellular response to superoxide | 3/87 | 27/18493 | 0.000271089 | 0.001845905 | 0.001019401 | SOD1/MPO/TNF | 3 |
| BP | GO:1990776 | response to angiotensin | 3/87 | 27/18493 | 0.000271089 | 0.001845905 | 0.001019401 | PTGS2/RELA/NFKB1 | 3 |
| BP | GO:0001935 | endothelial cell proliferation | 6/87 | 190/18493 | 0.000275293 | 0.001870429 | 0.001032945 | PPARG/IL10/SP1/AKT1/ITGB3/TNF | 6 |
| BP | GO:1903038 | negative regulation of leukocyte cell-cell adhesion | 5/87 | 122/18493 | 0.000276125 | 0.001871984 | 0.001033804 | ERBB2/PPARA/IL10/AKT1/CASP3 | 5 |
| BP | GO:0006720 | isoprenoid metabolic process | 5/87 | 123/18493 | 0.00028677 | 0.001935696 | 0.001068989 | HMGCR/PPARD/AKR1C1/RBP3/RLBP1 | 5 |
| BP | GO:0098754 | detoxification | 5/87 | 123/18493 | 0.00028677 | 0.001935696 | 0.001068989 | PTGS2/PTGS1/SOD1/MPO/TNF | 5 |
| BP | GO:0001667 | ameboidal-type cell migration | 9/87 | 456/18493 | 0.000291282 | 0.001961889 | 0.001083454 | PTGS2/PPARG/DPP4/PRSS3/PPARD/SP1/AKT1/ITGB3/P2RY12 | 9 |
| BP | GO:0050805 | negative regulation of synaptic transmission | 4/87 | 68/18493 | 0.0002958 | 0.001983715 | 0.001095507 | PTGS2/SLC6A4/ACHE/BCHE | 4 |
| BP | GO:0051881 | regulation of mitochondrial membrane potential | 4/87 | 68/18493 | 0.0002958 | 0.001983715 | 0.001095507 | SOD1/CCK/BCL2/AKT1 | 4 |
| BP | GO:0002675 | positive regulation of acute inflammatory response | 3/87 | 28/18493 | 0.00030259 | 0.001999027 | 0.001103963 | PTGS2/IL6/TNF | 3 |
| BP | GO:0005979 | regulation of glycogen biosynthetic process | 3/87 | 28/18493 | 0.00030259 | 0.001999027 | 0.001103963 | GSK3B/INS/AKT1 | 3 |
| BP | GO:0010962 | regulation of glucan biosynthetic process | 3/87 | 28/18493 | 0.00030259 | 0.001999027 | 0.001103963 | GSK3B/INS/AKT1 | 3 |
| BP | GO:0034368 | protein-lipid complex remodeling | 3/87 | 28/18493 | 0.00030259 | 0.001999027 | 0.001103963 | LPL/MPO/CETP | 3 |
| BP | GO:0034369 | plasma lipoprotein particle remodeling | 3/87 | 28/18493 | 0.00030259 | 0.001999027 | 0.001103963 | LPL/MPO/CETP | 3 |
| BP | GO:0072378 | blood coagulation, fibrin clot formation | 3/87 | 28/18493 | 0.00030259 | 0.001999027 | 0.001103963 | F2/F7/F10 | 3 |
| BP | GO:1904646 | cellular response to amyloid-beta | 3/87 | 28/18493 | 0.00030259 | 0.001999027 | 0.001103963 | ADRB2/GSK3B/APP | 3 |
| BP | GO:0007626 | locomotory behavior | 6/87 | 194/18493 | 0.000307833 | 0.002029345 | 0.001120706 | SLC6A3/DPP4/SOD1/GRIN1/GRIN2D/APP | 6 |
| BP | GO:0010565 | regulation of cellular ketone metabolic process | 5/87 | 125/18493 | 0.000308991 | 0.002032663 | 0.001122538 | PTGS2/PPARG/PPARA/INS/AKT1 | 5 |
| BP | GO:0033077 | T cell differentiation in thymus | 4/87 | 69/18493 | 0.000312884 | 0.002049586 | 0.001131884 | ADA/SOD1/ERBB2/BCL2 | 4 |
| BP | GO:1903524 | positive regulation of blood circulation | 4/87 | 69/18493 | 0.000312884 | 0.002049586 | 0.001131884 | PTGS2/ADA/ADRA1B/AKT1 | 4 |
| BP | GO:0007162 | negative regulation of cell adhesion | 7/87 | 276/18493 | 0.00032282 | 0.00211022 | 0.00116537 | ERBB2/SERPINE1/PPARA/PLG/IL10/AKT1/CASP3 | 7 |
| BP | GO:1903531 | negative regulation of secretion by cell | 6/87 | 196/18493 | 0.000325197 | 0.002121292 | 0.001171484 | MAOB/HMGCR/INS/IL10/TNF/P2RY12 | 6 |
| BP | GO:0014068 | positive regulation of phosphatidylinositol 3-kinase signaling | 4/87 | 70/18493 | 0.000330663 | 0.002147926 | 0.001186193 | F2/PPARD/INS/TNF | 4 |
| BP | GO:0048662 | negative regulation of smooth muscle cell proliferation | 4/87 | 70/18493 | 0.000330663 | 0.002147926 | 0.001186193 | PPARG/PPARD/IL10/PTGIR | 4 |
| BP | GO:0032770 | positive regulation of monooxygenase activity | 3/87 | 29/18493 | 0.00033636 | 0.0021668 | 0.001196616 | INS/AKT1/TNF | 3 |
| BP | GO:0042036 | negative regulation of cytokine biosynthetic process | 3/87 | 29/18493 | 0.00033636 | 0.0021668 | 0.001196616 | IL10/IL6/NFKB1 | 3 |
| BP | GO:0046320 | regulation of fatty acid oxidation | 3/87 | 29/18493 | 0.00033636 | 0.0021668 | 0.001196616 | PPARG/PPARA/AKT1 | 3 |
| BP | GO:1902175 | regulation of oxidative stress-induced intrinsic apoptotic signaling pathway | 3/87 | 29/18493 | 0.00033636 | 0.0021668 | 0.001196616 | SOD1/INS/AKT1 | 3 |
| BP | GO:0002576 | platelet degranulation | 5/87 | 128/18493 | 0.000344742 | 0.002211617 | 0.001221366 | SOD1/SERPINE1/PLG/APP/ITGB3 | 5 |
| BP | GO:0042177 | negative regulation of protein catabolic process | 5/87 | 128/18493 | 0.000344742 | 0.002211617 | 0.001221366 | NOS2/INS/IL10/RELA/GRIN2A | 5 |
| BP | GO:0000303 | response to superoxide | 3/87 | 30/18493 | 0.000372466 | 0.002369892 | 0.001308773 | SOD1/MPO/TNF | 3 |
| BP | GO:0001782 | B cell homeostasis | 3/87 | 30/18493 | 0.000372466 | 0.002369892 | 0.001308773 | ADA/BCL2/CASP3 | 3 |
| BP | GO:0034367 | protein-containing complex remodeling | 3/87 | 30/18493 | 0.000372466 | 0.002369892 | 0.001308773 | LPL/MPO/CETP | 3 |
| BP | GO:0045907 | positive regulation of vasoconstriction | 3/87 | 30/18493 | 0.000372466 | 0.002369892 | 0.001308773 | PTGS2/ADRA1B/AKT1 | 3 |
| BP | GO:0051249 | regulation of lymphocyte activation | 9/87 | 472/18493 | 0.000374803 | 0.002379883 | 0.001314291 | ADA/DPP4/SOD1/ERBB2/BCL2/IL10/IL6/AKT1/CASP3 | 9 |
| BP | GO:0030900 | forebrain development | 8/87 | 374/18493 | 0.000377522 | 0.002392259 | 0.001321126 | SCN5A/SLC6A3/GSK3B/GRIN1/APP/SLC2A1/CASP3/P2RY12 | 8 |
| BP | GO:0002064 | epithelial cell development | 6/87 | 202/18493 | 0.000381937 | 0.002415303 | 0.001333852 | ESR1/AR/GSK3B/PGR/SOD1/TNF | 6 |
| BP | GO:0042310 | vasoconstriction | 4/87 | 73/18493 | 0.000388341 | 0.002450813 | 0.001353462 | PTGS2/ADRA1B/SLC6A4/AKT1 | 4 |
| BP | GO:0030073 | insulin secretion | 6/87 | 203/18493 | 0.000392103 | 0.00246953 | 0.001363799 | NOS2/DPP4/HMGCR/PPARD/SLC2A1/TNF | 6 |
| BP | GO:0006006 | glucose metabolic process | 6/87 | 204/18493 | 0.000402479 | 0.00252975 | 0.001397055 | GSK3B/PPARA/PPARD/INS/AKT1/TNF | 6 |
| BP | GO:0005996 | monosaccharide metabolic process | 7/87 | 287/18493 | 0.000408348 | 0.002536935 | 0.001401023 | GSK3B/PPARA/PPARD/INS/AKT1/SLC2A1/TNF | 7 |
| BP | GO:0097193 | intrinsic apoptotic signaling pathway | 7/87 | 287/18493 | 0.000408348 | 0.002536935 | 0.001401023 | PTGS2/SOD1/INS/BCL2/AKT1/CASP3/TNF | 7 |
| BP | GO:0000271 | polysaccharide biosynthetic process | 4/87 | 74/18493 | 0.000409069 | 0.002536935 | 0.001401023 | GSK3B/INS/AKT1/NFKB1 | 4 |
| BP | GO:0010827 | regulation of glucose transmembrane transport | 4/87 | 74/18493 | 0.000409069 | 0.002536935 | 0.001401023 | INS/AKT1/SLC2A1/TNF | 4 |
| BP | GO:0000305 | response to oxygen radical | 3/87 | 31/18493 | 0.000410975 | 0.002536935 | 0.001401023 | SOD1/MPO/TNF | 3 |
| BP | GO:0001963 | synaptic transmission, dopaminergic | 3/87 | 31/18493 | 0.000410975 | 0.002536935 | 0.001401023 | PTGS2/SLC6A2/SLC6A4 | 3 |
| BP | GO:0055094 | response to lipoprotein particle | 3/87 | 31/18493 | 0.000410975 | 0.002536935 | 0.001401023 | PPARG/LPL/AKT1 | 3 |
| BP | GO:0071880 | adenylate cyclase-activating adrenergic receptor signaling pathway | 3/87 | 31/18493 | 0.000410975 | 0.002536935 | 0.001401023 | ADRA1B/ADRB2/ADRA1D | 3 |
| BP | GO:1904645 | response to amyloid-beta | 3/87 | 31/18493 | 0.000410975 | 0.002536935 | 0.001401023 | ADRB2/GSK3B/APP | 3 |
| BP | GO:0050670 | regulation of lymphocyte proliferation | 6/87 | 205/18493 | 0.000413069 | 0.002544798 | 0.001405365 | ADA/ERBB2/BCL2/IL10/IL6/CASP3 | 6 |
| BP | GO:0032944 | regulation of mononuclear cell proliferation | 6/87 | 206/18493 | 0.000423875 | 0.002606202 | 0.001439276 | ADA/ERBB2/BCL2/IL10/IL6/CASP3 | 6 |
| BP | GO:0045765 | regulation of angiogenesis | 8/87 | 381/18493 | 0.000427005 | 0.002620257 | 0.001447037 | PTGS2/PPARG/ERBB2/SERPINE1/IL10/SP1/IL6/PTGIS | 8 |
| BP | GO:0032642 | regulation of chemokine production | 4/87 | 75/18493 | 0.000430575 | 0.002631765 | 0.001453393 | LPL/IL10/IL6/TNF | 4 |
| BP | GO:1903201 | regulation of oxidative stress-induced cell death | 4/87 | 75/18493 | 0.000430575 | 0.002631765 | 0.001453393 | SOD1/INS/IL10/AKT1 | 4 |
| BP | GO:0014072 | response to isoquinoline alkaloid | 3/87 | 32/18493 | 0.000451953 | 0.002746211 | 0.001516596 | ADA/RELA/GRIN1 | 3 |
| BP | GO:0043278 | response to morphine | 3/87 | 32/18493 | 0.000451953 | 0.002746211 | 0.001516596 | ADA/RELA/GRIN1 | 3 |
| BP | GO:0045987 | positive regulation of smooth muscle contraction | 3/87 | 32/18493 | 0.000451953 | 0.002746211 | 0.001516596 | PTGS2/ADA/ADRA1B | 3 |
| BP | GO:0006970 | response to osmotic stress | 4/87 | 76/18493 | 0.000452877 | 0.00274645 | 0.001516728 | PTGS2/SLC2A1/CASP3/TNF | 4 |
| BP | GO:0009411 | response to UV | 5/87 | 136/18493 | 0.000455432 | 0.002756561 | 0.001522311 | PTGS2/BCL2/RELA/AKT1/CASP3 | 5 |
| BP | GO:0016051 | carbohydrate biosynthetic process | 6/87 | 209/18493 | 0.000457626 | 0.002759083 | 0.001523704 | GSK3B/PPARA/INS/AKT1/SLC2A1/NFKB1 | 6 |
| BP | GO:0043281 | regulation of cysteine-type endopeptidase activity involved in apoptotic process | 6/87 | 209/18493 | 0.000457626 | 0.002759083 | 0.001523704 | PTGS2/PPARG/CCK/AKT1/CASP3/TNF | 6 |
| BP | GO:0072330 | monocarboxylic acid biosynthetic process | 7/87 | 293/18493 | 0.000462127 | 0.002780821 | 0.001535709 | PTGS2/PTGS1/LPL/PPARA/INS/ALOX5/PTGIS | 7 |
| BP | GO:0050663 | cytokine secretion | 6/87 | 210/18493 | 0.00046933 | 0.002818703 | 0.001556629 | NOS2/LPL/CRP/INS/IL10/TNF | 6 |
| BP | GO:0001774 | microglial cell activation | 3/87 | 33/18493 | 0.000495464 | 0.002941519 | 0.001624454 | TRPV1/APP/TNF | 3 |
| BP | GO:0001975 | response to amphetamine | 3/87 | 33/18493 | 0.000495464 | 0.002941519 | 0.001624454 | SOD1/GRIN1/GRIN2A | 3 |
| BP | GO:0002269 | leukocyte activation involved in inflammatory response | 3/87 | 33/18493 | 0.000495464 | 0.002941519 | 0.001624454 | TRPV1/APP/TNF | 3 |
| BP | GO:0070873 | regulation of glycogen metabolic process | 3/87 | 33/18493 | 0.000495464 | 0.002941519 | 0.001624454 | GSK3B/INS/AKT1 | 3 |
| BP | GO:0071402 | cellular response to lipoprotein particle stimulus | 3/87 | 33/18493 | 0.000495464 | 0.002941519 | 0.001624454 | PPARG/LPL/AKT1 | 3 |
| BP | GO:1903427 | negative regulation of reactive oxygen species biosynthetic process | 3/87 | 33/18493 | 0.000495464 | 0.002941519 | 0.001624454 | INS/IL10/PTGIS | 3 |
| BP | GO:0046889 | positive regulation of lipid biosynthetic process | 4/87 | 78/18493 | 0.000499932 | 0.002951119 | 0.001629756 | PTGS2/INS/AKT1/TNF | 4 |
| BP | GO:0071260 | cellular response to mechanical stimulus | 4/87 | 78/18493 | 0.000499932 | 0.002951119 | 0.001629756 | PTGS2/AKT1/SLC2A1/NFKB1 | 4 |
| BP | GO:1900182 | positive regulation of protein localization to nucleus | 4/87 | 78/18493 | 0.000499932 | 0.002951119 | 0.001629756 | PTGS2/F2/INS/AKT1 | 4 |
| BP | GO:0061564 | axon development | 9/87 | 493/18493 | 0.000513595 | 0.003026021 | 0.00167112 | HSP90AA1/GSK3B/ERBB2/BDNF/CCK/BCL2/GRIN1/APP/CASP3 | 9 |
| BP | GO:0042737 | drug catabolic process | 5/87 | 140/18493 | 0.000519907 | 0.003057408 | 0.001688454 | ADA/SLC6A3/MAOB/MPO/ACHE | 5 |
| BP | GO:0008643 | carbohydrate transport | 5/87 | 141/18493 | 0.000537047 | 0.003146285 | 0.001737536 | PPARD/INS/AKT1/SLC2A1/TNF | 5 |
| BP | GO:0030534 | adult behavior | 5/87 | 141/18493 | 0.000537047 | 0.003146285 | 0.001737536 | PPARA/GABRG2/GRIN1/GRIN2D/APP | 5 |
| BP | GO:0071875 | adrenergic receptor signaling pathway | 3/87 | 34/18493 | 0.000541571 | 0.003160858 | 0.001745584 | ADRA1B/ADRB2/ADRA1D | 3 |
| BP | GO:0090322 | regulation of superoxide metabolic process | 3/87 | 34/18493 | 0.000541571 | 0.003160858 | 0.001745584 | SOD1/CRP/TNF | 3 |
| BP | GO:0008406 | gonad development | 6/87 | 216/18493 | 0.000544548 | 0.003166334 | 0.001748608 | ESR1/AR/PGR/SOD1/BCL2/CASP3 | 6 |
| BP | GO:0070663 | regulation of leukocyte proliferation | 6/87 | 216/18493 | 0.000544548 | 0.003166334 | 0.001748608 | ADA/ERBB2/BCL2/IL10/IL6/CASP3 | 6 |
| BP | GO:0042116 | macrophage activation | 4/87 | 80/18493 | 0.000550369 | 0.003188238 | 0.001760705 | TRPV1/IL10/APP/TNF | 4 |
| BP | GO:0045913 | positive regulation of carbohydrate metabolic process | 4/87 | 80/18493 | 0.000550369 | 0.003188238 | 0.001760705 | PPARA/INS/AKT1/NFKB1 | 4 |
| BP | GO:0070588 | calcium ion transmembrane transport | 7/87 | 302/18493 | 0.000553269 | 0.003199069 | 0.001766686 | TRPV1/F2/GRIN1/GRIN2A/GRIN2B/GRIN2D/P2RY12 | 7 |
| BP | GO:0030518 | intracellular steroid hormone receptor signaling pathway | 5/87 | 142/18493 | 0.000554609 | 0.003200854 | 0.001767672 | ESR1/AR/ESR2/NR3C1/PGR | 5 |
| BP | GO:0045923 | positive regulation of fatty acid metabolic process | 3/87 | 35/18493 | 0.000590336 | 0.003379607 | 0.001866389 | PTGS2/PPARG/PPARA | 3 |
| BP | GO:0071470 | cellular response to osmotic stress | 3/87 | 35/18493 | 0.000590336 | 0.003379607 | 0.001866389 | PTGS2/SLC2A1/CASP3 | 3 |
| BP | GO:1901099 | negative regulation of signal transduction in absence of ligand | 3/87 | 35/18493 | 0.000590336 | 0.003379607 | 0.001866389 | BCL2/AKT1/TNF | 3 |
| BP | GO:2001240 | negative regulation of extrinsic apoptotic signaling pathway in absence of ligand | 3/87 | 35/18493 | 0.000590336 | 0.003379607 | 0.001866389 | BCL2/AKT1/TNF | 3 |
| BP | GO:0055088 | lipid homeostasis | 5/87 | 144/18493 | 0.000591023 | 0.003379607 | 0.001866389 | PPARG/LPL/INS/CETP/AKR1C1 | 5 |
| BP | GO:0032602 | chemokine production | 4/87 | 82/18493 | 0.000604323 | 0.003449306 | 0.00190488 | LPL/IL10/IL6/TNF | 4 |
| BP | GO:0001894 | tissue homeostasis | 6/87 | 221/18493 | 0.000614128 | 0.003492432 | 0.001928696 | ADRB2/SOD1/BCL2/SLC22A5/IL6/ITGB3 | 6 |
| BP | GO:0007188 | adenylate cyclase-modulating G-protein coupled receptor signaling pathway | 6/87 | 221/18493 | 0.000614128 | 0.003492432 | 0.001928696 | CHRM2/ADRA1B/ADRB2/ADRA1D/P2RY12/PTGIR | 6 |
| BP | GO:0045137 | development of primary sexual characteristics | 6/87 | 222/18493 | 0.000628837 | 0.00356954 | 0.001971279 | ESR1/AR/PGR/SOD1/BCL2/CASP3 | 6 |
| BP | GO:0014910 | regulation of smooth muscle cell migration | 4/87 | 83/18493 | 0.000632661 | 0.003571659 | 0.001972449 | PLAU/SERPINE1/PPARD/BCL2 | 4 |
| BP | GO:0034637 | cellular carbohydrate biosynthetic process | 4/87 | 83/18493 | 0.000632661 | 0.003571659 | 0.001972449 | GSK3B/INS/AKT1/SLC2A1 | 4 |
| BP | GO:0106027 | neuron projection organization | 4/87 | 83/18493 | 0.000632661 | 0.003571659 | 0.001972449 | GSK3B/INS/GRIN2B/APP | 4 |
| BP | GO:0006692 | prostanoid metabolic process | 3/87 | 36/18493 | 0.000641822 | 0.003590735 | 0.001982984 | PTGS2/PTGS1/PTGIS | 3 |
| BP | GO:0006693 | prostaglandin metabolic process | 3/87 | 36/18493 | 0.000641822 | 0.003590735 | 0.001982984 | PTGS2/PTGS1/PTGIS | 3 |
| BP | GO:0032350 | regulation of hormone metabolic process | 3/87 | 36/18493 | 0.000641822 | 0.003590735 | 0.001982984 | VDR/NFKB1/TNF | 3 |
| BP | GO:1900271 | regulation of long-term synaptic potentiation | 3/87 | 36/18493 | 0.000641822 | 0.003590735 | 0.001982984 | INS/GRIN2A/APP | 3 |
| BP | GO:2000008 | regulation of protein localization to cell surface | 3/87 | 36/18493 | 0.000641822 | 0.003590735 | 0.001982984 | BDNF/AKT1/TNF | 3 |
| BP | GO:0016485 | protein processing | 7/87 | 310/18493 | 0.000645817 | 0.003606589 | 0.001991739 | F2/F7/PRSS3/PLAU/SERPINE1/GRIN2A/CASP3 | 7 |
| BP | GO:0070542 | response to fatty acid | 4/87 | 84/18493 | 0.000661929 | 0.003689929 | 0.002037764 | PTGS2/PPARG/PON1/AKT1 | 4 |
| BP | GO:0014037 | Schwann cell differentiation | 3/87 | 37/18493 | 0.000696088 | 0.003866466 | 0.002135257 | SOD1/RELA/AKT1 | 3 |
| BP | GO:0045684 | positive regulation of epidermis development | 3/87 | 37/18493 | 0.000696088 | 0.003866466 | 0.002135257 | PPARD/VDR/TNF | 3 |
| BP | GO:0046394 | carboxylic acid biosynthetic process | 8/87 | 412/18493 | 0.000713744 | 0.003957455 | 0.002185505 | PTGS2/PTGS1/LPL/PPARA/INS/ALOX5/NFKB1/PTGIS | 8 |
| BP | GO:0001523 | retinoid metabolic process | 4/87 | 86/18493 | 0.000723324 | 0.004003426 | 0.002210893 | PPARD/AKR1C1/RBP3/RLBP1 | 4 |
| BP | GO:0016053 | organic acid biosynthetic process | 8/87 | 413/18493 | 0.000725086 | 0.004006035 | 0.002212334 | PTGS2/PTGS1/LPL/PPARA/INS/ALOX5/NFKB1/PTGIS | 8 |
| BP | GO:0033238 | regulation of cellular amine metabolic process | 3/87 | 38/18493 | 0.000753195 | 0.004146577 | 0.002289948 | SLC6A3/MAOB/INS | 3 |
| BP | GO:0045687 | positive regulation of glial cell differentiation | 3/87 | 38/18493 | 0.000753195 | 0.004146577 | 0.002289948 | PPARG/RELA/APP | 3 |
| BP | GO:0050829 | defense response to Gram-negative bacterium | 4/87 | 87/18493 | 0.000755485 | 0.004151826 | 0.002292847 | NOS2/F2/SERPINE1/IL6 | 4 |
| BP | GO:0051262 | protein tetramerization | 5/87 | 153/18493 | 0.000777545 | 0.004265507 | 0.002355627 | TRPV1/HMGCR/GRIN1/GRIN2B/ACHE | 5 |
| BP | GO:0006919 | activation of cysteine-type endopeptidase activity involved in apoptotic process | 4/87 | 88/18493 | 0.000788645 | 0.004318768 | 0.00238504 | PPARG/CCK/CASP3/TNF | 4 |
| BP | GO:0010677 | negative regulation of cellular carbohydrate metabolic process | 3/87 | 39/18493 | 0.000813199 | 0.004414306 | 0.002437801 | GSK3B/PPARA/INS | 3 |
| BP | GO:0042417 | dopamine metabolic process | 3/87 | 39/18493 | 0.000813199 | 0.004414306 | 0.002437801 | SLC6A3/MAOB/GRIN2A | 3 |
| BP | GO:0043029 | T cell homeostasis | 3/87 | 39/18493 | 0.000813199 | 0.004414306 | 0.002437801 | BCL2/AKT1/CASP3 | 3 |
| BP | GO:0060119 | inner ear receptor cell development | 3/87 | 39/18493 | 0.000813199 | 0.004414306 | 0.002437801 | SOD1/GABRA5/GABRB3 | 3 |
| BP | GO:0098815 | modulation of excitatory postsynaptic potential | 3/87 | 39/18493 | 0.000813199 | 0.004414306 | 0.002437801 | ADRB2/GRIN1/APP | 3 |
| BP | GO:1901342 | regulation of vasculature development | 8/87 | 421/18493 | 0.000821163 | 0.004449758 | 0.00245738 | PTGS2/PPARG/ERBB2/SERPINE1/IL10/SP1/IL6/PTGIS | 8 |
| BP | GO:0006633 | fatty acid biosynthetic process | 5/87 | 156/18493 | 0.000848539 | 0.004590096 | 0.002534881 | PTGS2/PTGS1/LPL/ALOX5/PTGIS | 5 |
| BP | GO:0035249 | synaptic transmission, glutamatergic | 4/87 | 90/18493 | 0.000858027 | 0.004625302 | 0.002554324 | PTGS2/GRIA2/GRIN1/TNF | 4 |
| BP | GO:0051899 | membrane depolarization | 4/87 | 90/18493 | 0.000858027 | 0.004625302 | 0.002554324 | SCN5A/KCNH2/CCK/BCL2 | 4 |
| BP | GO:0060249 | anatomical structure homeostasis | 8/87 | 425/18493 | 0.000872908 | 0.004697365 | 0.002594121 | SLC6A2/HSP90AA1/ADRB2/SOD1/BCL2/SLC22A5/IL6/ITGB3 | 8 |
| BP | GO:0050873 | brown fat cell differentiation | 3/87 | 40/18493 | 0.000876159 | 0.004698573 | 0.002594788 | PTGS2/ADRB2/INS | 3 |
| BP | GO:0061900 | glial cell activation | 3/87 | 40/18493 | 0.000876159 | 0.004698573 | 0.002594788 | TRPV1/APP/TNF | 3 |
| BP | GO:0042100 | B cell proliferation | 4/87 | 91/18493 | 0.000894284 | 0.004779264 | 0.00263935 | ADA/BCL2/IL10/CASP3 | 4 |
| BP | GO:1901570 | fatty acid derivative biosynthetic process | 4/87 | 91/18493 | 0.000894284 | 0.004779264 | 0.00263935 | PTGS2/PTGS1/ALOX5/PTGIS | 4 |
| BP | GO:0016101 | diterpenoid metabolic process | 4/87 | 92/18493 | 0.000931608 | 0.004970179 | 0.002744782 | PPARD/AKR1C1/RBP3/RLBP1 | 4 |
| BP | GO:0071456 | cellular response to hypoxia | 5/87 | 160/18493 | 0.000950588 | 0.005030313 | 0.002777992 | PTGS2/PPARD/BCL2/AKT1/PTGIS | 5 |
| BP | GO:1901568 | fatty acid derivative metabolic process | 5/87 | 160/18493 | 0.000950588 | 0.005030313 | 0.002777992 | PTGS2/PTGS1/PON1/ALOX5/PTGIS | 5 |
| BP | GO:0001820 | serotonin secretion | 2/87 | 10/18493 | 0.000960701 | 0.005030313 | 0.002777992 | SLC6A4/MAOB | 2 |
| BP | GO:0002679 | respiratory burst involved in defense response | 2/87 | 10/18493 | 0.000960701 | 0.005030313 | 0.002777992 | MPO/INS | 2 |
| BP | GO:0021548 | pons development | 2/87 | 10/18493 | 0.000960701 | 0.005030313 | 0.002777992 | BCL2/GRIN1 | 2 |
| BP | GO:0045073 | regulation of chemokine biosynthetic process | 2/87 | 10/18493 | 0.000960701 | 0.005030313 | 0.002777992 | IL6/TNF | 2 |
| BP | GO:0045713 | low-density lipoprotein particle receptor biosynthetic process | 2/87 | 10/18493 | 0.000960701 | 0.005030313 | 0.002777992 | PPARG/ITGB3 | 2 |
| BP | GO:0060736 | prostate gland growth | 2/87 | 10/18493 | 0.000960701 | 0.005030313 | 0.002777992 | ESR1/AR | 2 |
| BP | GO:0090336 | positive regulation of brown fat cell differentiation | 2/87 | 10/18493 | 0.000960701 | 0.005030313 | 0.002777992 | PTGS2/INS | 2 |
| BP | GO:1990535 | neuron projection maintenance | 2/87 | 10/18493 | 0.000960701 | 0.005030313 | 0.002777992 | INS/APP | 2 |
| BP | GO:2000628 | regulation of miRNA metabolic process | 2/87 | 10/18493 | 0.000960701 | 0.005030313 | 0.002777992 | RELA/NFKB1 | 2 |
| BP | GO:1901655 | cellular response to ketone | 4/87 | 93/18493 | 0.000970017 | 0.00506202 | 0.002795502 | AR/PPARG/NR3C1/AKT1 | 4 |
| BP | GO:1903076 | regulation of protein localization to plasma membrane | 4/87 | 93/18493 | 0.000970017 | 0.00506202 | 0.002795502 | AR/INS/AKT1/TNF | 4 |
| BP | GO:0050954 | sensory perception of mechanical stimulus | 5/87 | 161/18493 | 0.000977467 | 0.005083812 | 0.002807536 | TRPV1/SOD1/GABRA5/GABRB3/CASP3 | 5 |
| BP | GO:2001242 | regulation of intrinsic apoptotic signaling pathway | 5/87 | 161/18493 | 0.000977467 | 0.005083812 | 0.002807536 | PTGS2/SOD1/INS/BCL2/AKT1 | 5 |
| BP | GO:0006109 | regulation of carbohydrate metabolic process | 5/87 | 162/18493 | 0.001004908 | 0.005200401 | 0.002871922 | GSK3B/PPARA/INS/AKT1/NFKB1 | 5 |
| BP | GO:0006733 | oxidoreduction coenzyme metabolic process | 5/87 | 162/18493 | 0.001004908 | 0.005200401 | 0.002871922 | PTGS2/HMGCR/PPARA/INS/PTGIS | 5 |
| BP | GO:0050680 | negative regulation of epithelial cell proliferation | 5/87 | 162/18493 | 0.001004908 | 0.005200401 | 0.002871922 | AR/PPARG/PPARD/VDR/TNF | 5 |
| BP | GO:0001504 | neurotransmitter uptake | 3/87 | 42/18493 | 0.00101117 | 0.005215422 | 0.002880218 | SLC6A2/SLC6A3/SLC6A4 | 3 |
| BP | GO:0010863 | positive regulation of phospholipase C activity | 3/87 | 42/18493 | 0.00101117 | 0.005215422 | 0.002880218 | ESR1/BDNF/P2RY12 | 3 |
| BP | GO:0019318 | hexose metabolic process | 6/87 | 244/18493 | 0.001027594 | 0.005282583 | 0.002917307 | GSK3B/PPARA/PPARD/INS/AKT1/TNF | 6 |
| BP | GO:0060047 | heart contraction | 6/87 | 244/18493 | 0.001027594 | 0.005282583 | 0.002917307 | CHRM2/SCN5A/ADA/ADRA1B/KCNH2/SOD1 | 6 |
| BP | GO:0002683 | negative regulation of immune system process | 8/87 | 437/18493 | 0.001044082 | 0.005358468 | 0.002959215 | ADA/PPARG/ERBB2/INS/IL10/AKT1/CASP3/TNF | 8 |
| BP | GO:0060191 | regulation of lipase activity | 4/87 | 95/18493 | 0.001050154 | 0.005380741 | 0.002971515 | ESR1/LPL/BDNF/P2RY12 | 4 |
| BP | GO:0031589 | cell-substrate adhesion | 7/87 | 338/18493 | 0.00107063 | 0.005476617 | 0.003024463 | GSK3B/PLAU/SERPINE1/PPARD/PLG/BCL2/ITGB3 | 7 |
| BP | GO:0010907 | positive regulation of glucose metabolic process | 3/87 | 43/18493 | 0.001083332 | 0.005514336 | 0.003045293 | PPARA/INS/AKT1 | 3 |
| BP | GO:0032369 | negative regulation of lipid transport | 3/87 | 43/18493 | 0.001083332 | 0.005514336 | 0.003045293 | AKT1/ITGB3/NFKB1 | 3 |
| BP | GO:0045912 | negative regulation of carbohydrate metabolic process | 3/87 | 43/18493 | 0.001083332 | 0.005514336 | 0.003045293 | GSK3B/PPARA/INS | 3 |
| BP | GO:0051100 | negative regulation of binding | 5/87 | 165/18493 | 0.001090674 | 0.005539877 | 0.003059398 | ADRB2/GSK3B/PPARA/IL10/AKT1 | 5 |
| BP | GO:2001243 | negative regulation of intrinsic apoptotic signaling pathway | 4/87 | 96/18493 | 0.001091918 | 0.005539877 | 0.003059398 | PTGS2/INS/BCL2/AKT1 | 4 |
| BP | GO:0021537 | telencephalon development | 6/87 | 248/18493 | 0.001117266 | 0.00565002 | 0.003120224 | SCN5A/GSK3B/GRIN1/SLC2A1/CASP3/P2RY12 | 6 |
| BP | GO:0048872 | homeostasis of number of cells | 6/87 | 248/18493 | 0.001117266 | 0.00565002 | 0.003120224 | ADA/SOD1/BCL2/IL6/AKT1/CASP3 | 6 |
| BP | GO:0021782 | glial cell development | 4/87 | 97/18493 | 0.001134834 | 0.005720227 | 0.003158997 | SOD1/AKT1/APP/TNF | 4 |
| BP | GO:0022600 | digestive system process | 4/87 | 97/18493 | 0.001134834 | 0.005720227 | 0.003158997 | TRPV1/SLC22A5/AKR1C1/VDR | 4 |
| BP | GO:1900274 | regulation of phospholipase C activity | 3/87 | 44/18493 | 0.001158669 | 0.005778565 | 0.003191214 | ESR1/BDNF/P2RY12 | 3 |
| BP | GO:0019336 | phenol-containing compound catabolic process | 2/87 | 11/18493 | 0.001170601 | 0.005778565 | 0.003191214 | SLC6A3/MAOB | 2 |
| BP | GO:0031652 | positive regulation of heat generation | 2/87 | 11/18493 | 0.001170601 | 0.005778565 | 0.003191214 | PTGS2/TNF | 2 |
| BP | GO:0032494 | response to peptidoglycan | 2/87 | 11/18493 | 0.001170601 | 0.005778565 | 0.003191214 | RELA/IL6 | 2 |
| BP | GO:0033197 | response to vitamin E | 2/87 | 11/18493 | 0.001170601 | 0.005778565 | 0.003191214 | ADA/PPARG | 2 |
| BP | GO:0033632 | regulation of cell-cell adhesion mediated by integrin | 2/87 | 11/18493 | 0.001170601 | 0.005778565 | 0.003191214 | ADA/DPP4 | 2 |
| BP | GO:0034372 | very-low-density lipoprotein particle remodeling | 2/87 | 11/18493 | 0.001170601 | 0.005778565 | 0.003191214 | LPL/CETP | 2 |
| BP | GO:0042033 | chemokine biosynthetic process | 2/87 | 11/18493 | 0.001170601 | 0.005778565 | 0.003191214 | IL6/TNF | 2 |
| BP | GO:0045945 | positive regulation of transcription by RNA polymerase III | 2/87 | 11/18493 | 0.001170601 | 0.005778565 | 0.003191214 | AR/ERBB2 | 2 |
| BP | GO:0050755 | chemokine metabolic process | 2/87 | 11/18493 | 0.001170601 | 0.005778565 | 0.003191214 | IL6/TNF | 2 |
| BP | GO:0060456 | positive regulation of digestive system process | 2/87 | 11/18493 | 0.001170601 | 0.005778565 | 0.003191214 | TRPV1/SLC22A5 | 2 |
| BP | GO:0070673 | response to interleukin-18 | 2/87 | 11/18493 | 0.001170601 | 0.005778565 | 0.003191214 | AKT1/ALOX5 | 2 |
| BP | GO:1902950 | regulation of dendritic spine maintenance | 2/87 | 11/18493 | 0.001170601 | 0.005778565 | 0.003191214 | INS/GRIN2B | 2 |
| BP | GO:0019395 | fatty acid oxidation | 4/87 | 98/18493 | 0.00117892 | 0.005810392 | 0.00320879 | PPARG/PPARA/PPARD/AKT1 | 4 |
| BP | GO:0022409 | positive regulation of cell-cell adhesion | 6/87 | 251/18493 | 0.001188375 | 0.00584771 | 0.003229399 | ADA/DPP4/IL10/IL6/AKT1/TNF | 6 |
| BP | GO:0003015 | heart process | 6/87 | 252/18493 | 0.001212833 | 0.005951676 | 0.003286814 | CHRM2/SCN5A/ADA/ADRA1B/KCNH2/SOD1 | 6 |
| BP | GO:0021543 | pallium development | 5/87 | 169/18493 | 0.001213337 | 0.005951676 | 0.003286814 | GSK3B/GRIN1/SLC2A1/CASP3/P2RY12 | 5 |
| BP | GO:0018958 | phenol-containing compound metabolic process | 4/87 | 99/18493 | 0.001224193 | 0.005995458 | 0.003310993 | SLC6A3/MAOB/BCL2/GRIN2A | 4 |
| BP | GO:0014075 | response to amine | 3/87 | 45/18493 | 0.001237234 | 0.006021332 | 0.003325282 | SOD1/GRIN1/GRIN2A | 3 |
| BP | GO:0031018 | endocrine pancreas development | 3/87 | 45/18493 | 0.001237234 | 0.006021332 | 0.003325282 | GSK3B/IL6/AKT1 | 3 |
| BP | GO:0045776 | negative regulation of blood pressure | 3/87 | 45/18493 | 0.001237234 | 0.006021332 | 0.003325282 | ADRB2/NOS2/PPARA | 3 |
| BP | GO:0071827 | plasma lipoprotein particle organization | 3/87 | 45/18493 | 0.001237234 | 0.006021332 | 0.003325282 | LPL/MPO/CETP | 3 |
| BP | GO:0007409 | axonogenesis | 8/87 | 449/18493 | 0.001241198 | 0.006026196 | 0.003327968 | HSP90AA1/GSK3B/ERBB2/BDNF/CCK/BCL2/GRIN1/APP | 8 |
| BP | GO:0036294 | cellular response to decreased oxygen levels | 5/87 | 170/18493 | 0.001245536 | 0.006026196 | 0.003327968 | PTGS2/PPARD/BCL2/AKT1/PTGIS | 5 |
| BP | GO:0048771 | tissue remodeling | 5/87 | 170/18493 | 0.001245536 | 0.006026196 | 0.003327968 | ADRB2/PLG/IL6/VDR/ITGB3 | 5 |
| BP | GO:0031331 | positive regulation of cellular catabolic process | 7/87 | 347/18493 | 0.001245996 | 0.006026196 | 0.003327968 | HSP90AA1/ADRB2/GSK3B/PPARA/INS/AKT1/TNF | 7 |
| BP | GO:0030198 | extracellular matrix organization | 7/87 | 348/18493 | 0.001266794 | 0.006117257 | 0.003378256 | DPP4/SERPINE1/PLG/APP/ITGB3/NFKB2/TNF | 7 |
| BP | GO:0034440 | lipid oxidation | 4/87 | 100/18493 | 0.001270671 | 0.006126447 | 0.003383332 | PPARG/PPARA/PPARD/AKT1 | 4 |
| BP | GO:0001659 | temperature homeostasis | 5/87 | 171/18493 | 0.001278363 | 0.006144454 | 0.003393276 | PTGS2/TRPV1/ADRB2/ACHE/TNF | 5 |
| BP | GO:0071466 | cellular response to xenobiotic stimulus | 5/87 | 171/18493 | 0.001278363 | 0.006144454 | 0.003393276 | PTGS1/NR3C1/AKR1C1/GRIN1/BCHE | 5 |
| BP | GO:0050769 | positive regulation of neurogenesis | 8/87 | 453/18493 | 0.001313139 | 0.006291456 | 0.003474458 | PPARG/BDNF/BCL2/RELA/IL6/APP/TNF/P2RY12 | 8 |
| BP | GO:0007187 | G-protein coupled receptor signaling pathway, coupled to cyclic nucleotide second messenger | 6/87 | 256/18493 | 0.00131455 | 0.006291456 | 0.003474458 | CHRM2/ADRA1B/ADRB2/ADRA1D/P2RY12/PTGIR | 6 |
| BP | GO:0005978 | glycogen biosynthetic process | 3/87 | 46/18493 | 0.001319078 | 0.006291456 | 0.003474458 | GSK3B/INS/AKT1 | 3 |
| BP | GO:0009250 | glucan biosynthetic process | 3/87 | 46/18493 | 0.001319078 | 0.006291456 | 0.003474458 | GSK3B/INS/AKT1 | 3 |
| BP | GO:2001239 | regulation of extrinsic apoptotic signaling pathway in absence of ligand | 3/87 | 46/18493 | 0.001319078 | 0.006291456 | 0.003474458 | BCL2/AKT1/TNF | 3 |
| BP | GO:0031334 | positive regulation of protein complex assembly | 6/87 | 259/18493 | 0.001395027 | 0.006626455 | 0.003659461 | HSP90AA1/ESR1/GSK3B/CCK/TNF/P2RY12 | 6 |
| BP | GO:0002674 | negative regulation of acute inflammatory response | 2/87 | 12/18493 | 0.00140043 | 0.006626455 | 0.003659461 | PPARG/INS | 2 |
| BP | GO:0032096 | negative regulation of response to food | 2/87 | 12/18493 | 0.00140043 | 0.006626455 | 0.003659461 | PPARA/CCK | 2 |
| BP | GO:0032099 | negative regulation of appetite | 2/87 | 12/18493 | 0.00140043 | 0.006626455 | 0.003659461 | PPARA/CCK | 2 |
| BP | GO:0034370 | triglyceride-rich lipoprotein particle remodeling | 2/87 | 12/18493 | 0.00140043 | 0.006626455 | 0.003659461 | LPL/CETP | 2 |
| BP | GO:0006636 | unsaturated fatty acid biosynthetic process | 3/87 | 47/18493 | 0.001404254 | 0.006626455 | 0.003659461 | PTGS2/PTGS1/PTGIS | 3 |
| BP | GO:0022602 | ovulation cycle process | 3/87 | 47/18493 | 0.001404254 | 0.006626455 | 0.003659461 | ESR1/PGR/CASP3 | 3 |
| BP | GO:0008544 | epidermis development | 8/87 | 458/18493 | 0.001407706 | 0.006632665 | 0.003662891 | SOD1/PPARA/PPARD/BCL2/RELA/VDR/CASP3/TNF | 8 |
| BP | GO:0032409 | regulation of transporter activity | 6/87 | 260/18493 | 0.001422673 | 0.006693031 | 0.003696228 | ADRB2/PPARG/PON1/INS/BCL2/APP | 6 |
| BP | GO:0006721 | terpenoid metabolic process | 4/87 | 104/18493 | 0.001468962 | 0.006889921 | 0.00380496 | PPARD/AKR1C1/RBP3/RLBP1 | 4 |
| BP | GO:0034605 | cellular response to heat | 4/87 | 104/18493 | 0.001468962 | 0.006889921 | 0.00380496 | PTGS2/TRPV1/HSP90AA1/GSK3B | 4 |
| BP | GO:0003254 | regulation of membrane depolarization | 3/87 | 48/18493 | 0.00149281 | 0.006970186 | 0.003849286 | SCN5A/CCK/BCL2 | 3 |
| BP | GO:0045933 | positive regulation of muscle contraction | 3/87 | 48/18493 | 0.00149281 | 0.006970186 | 0.003849286 | PTGS2/ADA/ADRA1B | 3 |
| BP | GO:0050999 | regulation of nitric-oxide synthase activity | 3/87 | 48/18493 | 0.00149281 | 0.006970186 | 0.003849286 | HSP90AA1/INS/AKT1 | 3 |
| BP | GO:0045861 | negative regulation of proteolysis | 7/87 | 359/18493 | 0.001514048 | 0.007058736 | 0.003898189 | PTGS2/F2/SERPINE1/INS/IL10/AKT1/APP | 7 |
| BP | GO:0046651 | lymphocyte proliferation | 6/87 | 264/18493 | 0.001537467 | 0.007157174 | 0.003952551 | ADA/ERBB2/BCL2/IL10/IL6/CASP3 | 6 |
| BP | GO:0014066 | regulation of phosphatidylinositol 3-kinase signaling | 4/87 | 106/18493 | 0.001575777 | 0.007291784 | 0.004026889 | F2/PPARD/INS/TNF | 4 |
| BP | GO:0019359 | nicotinamide nucleotide biosynthetic process | 4/87 | 106/18493 | 0.001575777 | 0.007291784 | 0.004026889 | PTGS2/PPARA/INS/PTGIS | 4 |
| BP | GO:0019363 | pyridine nucleotide biosynthetic process | 4/87 | 106/18493 | 0.001575777 | 0.007291784 | 0.004026889 | PTGS2/PPARA/INS/PTGIS | 4 |
| BP | GO:0032963 | collagen metabolic process | 4/87 | 106/18493 | 0.001575777 | 0.007291784 | 0.004026889 | F2/PPARG/PPARD/IL6 | 4 |
| BP | GO:0071825 | protein-lipid complex subunit organization | 3/87 | 49/18493 | 0.001584795 | 0.007322601 | 0.004043908 | LPL/MPO/CETP | 3 |
| BP | GO:0032943 | mononuclear cell proliferation | 6/87 | 266/18493 | 0.001597448 | 0.007370098 | 0.004070138 | ADA/ERBB2/BCL2/IL10/IL6/CASP3 | 6 |
| BP | GO:0048639 | positive regulation of developmental growth | 5/87 | 180/18493 | 0.001603333 | 0.007386273 | 0.004079071 | SLC6A3/BDNF/PPARD/BCL2/AKT1 | 5 |
| BP | GO:0051099 | positive regulation of binding | 5/87 | 181/18493 | 0.001642861 | 0.007523125 | 0.004154647 | PPARG/GSK3B/BDNF/PON1/APP | 5 |
| BP | GO:0001967 | suckling behavior | 2/87 | 13/18493 | 0.001649999 | 0.007523125 | 0.004154647 | GRIN1/APP | 2 |
| BP | GO:0001973 | adenosine receptor signaling pathway | 2/87 | 13/18493 | 0.001649999 | 0.007523125 | 0.004154647 | ADA/P2RY12 | 2 |
| BP | GO:0031650 | regulation of heat generation | 2/87 | 13/18493 | 0.001649999 | 0.007523125 | 0.004154647 | PTGS2/TNF | 2 |
| BP | GO:0050862 | positive regulation of T cell receptor signaling pathway | 2/87 | 13/18493 | 0.001649999 | 0.007523125 | 0.004154647 | ADA/RELA | 2 |
| BP | GO:0060965 | negative regulation of gene silencing by miRNA | 2/87 | 13/18493 | 0.001649999 | 0.007523125 | 0.004154647 | ESR1/PPARG | 2 |
| BP | GO:0090197 | positive regulation of chemokine secretion | 2/87 | 13/18493 | 0.001649999 | 0.007523125 | 0.004154647 | LPL/TNF | 2 |
| BP | GO:0007548 | sex differentiation | 6/87 | 268/18493 | 0.001659197 | 0.007553968 | 0.00417168 | ESR1/AR/PGR/SOD1/BCL2/CASP3 | 6 |
| BP | GO:0032655 | regulation of interleukin-12 production | 3/87 | 50/18493 | 0.001680258 | 0.00761635 | 0.004206131 | IL10/RELA/NFKB1 | 3 |
| BP | GO:0045540 | regulation of cholesterol biosynthetic process | 3/87 | 50/18493 | 0.001680258 | 0.00761635 | 0.004206131 | SOD1/HMGCR/SP1 | 3 |
| BP | GO:0106118 | NA | 3/87 | 50/18493 | 0.001680258 | 0.00761635 | 0.004206131 | SOD1/HMGCR/SP1 | 3 |
| BP | GO:0001676 | long-chain fatty acid metabolic process | 4/87 | 108/18493 | 0.001687885 | 0.007617562 | 0.0042068 | PTGS2/PTGS1/ALOX5/PTGIS | 4 |
| BP | GO:0046620 | regulation of organ growth | 4/87 | 108/18493 | 0.001687885 | 0.007617562 | 0.0042068 | SLC6A4/SOD1/PPARA/AKT1 | 4 |
| BP | GO:1904019 | epithelial cell apoptotic process | 4/87 | 108/18493 | 0.001687885 | 0.007617562 | 0.0042068 | SERPINE1/IL10/IL6/TNF | 4 |
| BP | GO:0034504 | protein localization to nucleus | 6/87 | 269/18493 | 0.001690745 | 0.007619393 | 0.004207811 | PTGS2/F2/GSK3B/INS/AKT1/TNF | 6 |
| BP | GO:0050864 | regulation of B cell activation | 5/87 | 183/18493 | 0.001724062 | 0.007748487 | 0.004279104 | ADA/BCL2/IL10/IL6/CASP3 | 5 |
| BP | GO:0006644 | phospholipid metabolic process | 8/87 | 473/18493 | 0.001724382 | 0.007748487 | 0.004279104 | ESR1/ERBB2/LPL/PPARD/PON1/CETP/ACHE/P2RY12 | 8 |
| BP | GO:0005976 | polysaccharide metabolic process | 4/87 | 109/18493 | 0.001745967 | 0.007811565 | 0.004313938 | GSK3B/INS/AKT1/NFKB1 | 4 |
| BP | GO:0018107 | peptidyl-threonine phosphorylation | 4/87 | 109/18493 | 0.001745967 | 0.007811565 | 0.004313938 | GSK3B/BCL2/AKT1/APP | 4 |
| BP | GO:0072525 | pyridine-containing compound biosynthetic process | 4/87 | 109/18493 | 0.001745967 | 0.007811565 | 0.004313938 | PTGS2/PPARA/INS/PTGIS | 4 |
| BP | GO:0030308 | negative regulation of cell growth | 5/87 | 184/18493 | 0.00176575 | 0.007866075 | 0.004344042 | PPARG/ESR2/PPARA/PPARD/BCL2 | 5 |
| BP | GO:0050707 | regulation of cytokine secretion | 5/87 | 184/18493 | 0.00176575 | 0.007866075 | 0.004344042 | LPL/CRP/INS/IL10/TNF | 5 |
| BP | GO:0071248 | cellular response to metal ion | 5/87 | 184/18493 | 0.00176575 | 0.007866075 | 0.004344042 | PTGS2/SCN5A/SOD1/AKT1/APP | 5 |
| BP | GO:0019369 | arachidonic acid metabolic process | 3/87 | 51/18493 | 0.001779245 | 0.007914836 | 0.00437097 | PTGS2/PTGS1/PTGIS | 3 |
| BP | GO:0043900 | regulation of multi-organism process | 7/87 | 371/18493 | 0.001825215 | 0.008107713 | 0.004477486 | ADA/NOS2/MPO/BCL2/IL10/SP1/TNF | 7 |
| BP | GO:0002696 | positive regulation of leukocyte activation | 7/87 | 372/18493 | 0.001853233 | 0.008220411 | 0.004539723 | ADA/DPP4/BCL2/IL10/IL6/AKT1/APP | 7 |
| BP | GO:0010906 | regulation of glucose metabolic process | 4/87 | 111/18493 | 0.001866269 | 0.00824291 | 0.004552149 | GSK3B/PPARA/INS/AKT1 | 4 |
| BP | GO:0021987 | cerebral cortex development | 4/87 | 111/18493 | 0.001866269 | 0.00824291 | 0.004552149 | GSK3B/GRIN1/SLC2A1/P2RY12 | 4 |
| BP | GO:1904375 | regulation of protein localization to cell periphery | 4/87 | 111/18493 | 0.001866269 | 0.00824291 | 0.004552149 | AR/INS/AKT1/TNF | 4 |
| BP | GO:0032615 | interleukin-12 production | 3/87 | 52/18493 | 0.001881804 | 0.008276204 | 0.004570535 | IL10/RELA/NFKB1 | 3 |
| BP | GO:0043030 | regulation of macrophage activation | 3/87 | 52/18493 | 0.001881804 | 0.008276204 | 0.004570535 | IL10/APP/TNF | 3 |
| BP | GO:0097366 | response to bronchodilator | 3/87 | 52/18493 | 0.001881804 | 0.008276204 | 0.004570535 | SOD1/GRIN1/GRIN2A | 3 |
| BP | GO:0014854 | response to inactivity | 2/87 | 14/18493 | 0.001919122 | 0.008369206 | 0.004621896 | SCN5A/IL10 | 2 |
| BP | GO:0032105 | negative regulation of response to extracellular stimulus | 2/87 | 14/18493 | 0.001919122 | 0.008369206 | 0.004621896 | PPARA/CCK | 2 |
| BP | GO:0032108 | negative regulation of response to nutrient levels | 2/87 | 14/18493 | 0.001919122 | 0.008369206 | 0.004621896 | PPARA/CCK | 2 |
| BP | GO:0034374 | low-density lipoprotein particle remodeling | 2/87 | 14/18493 | 0.001919122 | 0.008369206 | 0.004621896 | MPO/CETP | 2 |
| BP | GO:0043217 | myelin maintenance | 2/87 | 14/18493 | 0.001919122 | 0.008369206 | 0.004621896 | SOD1/AKT1 | 2 |
| BP | GO:0048143 | astrocyte activation | 2/87 | 14/18493 | 0.001919122 | 0.008369206 | 0.004621896 | APP/TNF | 2 |
| BP | GO:0001938 | positive regulation of endothelial cell proliferation | 4/87 | 112/18493 | 0.001928524 | 0.00839841 | 0.004638023 | IL10/SP1/AKT1/ITGB3 | 4 |
| BP | GO:0031099 | regeneration | 5/87 | 188/18493 | 0.001939937 | 0.00843628 | 0.004658937 | PPARG/F7/PPARD/BCL2/IL10 | 5 |
| BP | GO:0032722 | positive regulation of chemokine production | 3/87 | 53/18493 | 0.001987979 | 0.008615306 | 0.004757805 | LPL/IL6/TNF | 3 |
| BP | GO:0032479 | regulation of type I interferon production | 4/87 | 113/18493 | 0.001992203 | 0.008615306 | 0.004757805 | IL10/RELA/NFKB1/NFKB2 | 4 |
| BP | GO:0042303 | molting cycle | 4/87 | 113/18493 | 0.001992203 | 0.008615306 | 0.004757805 | PTGS2/BCL2/RELA/TNF | 4 |
| BP | GO:0042633 | hair cycle | 4/87 | 113/18493 | 0.001992203 | 0.008615306 | 0.004757805 | PTGS2/BCL2/RELA/TNF | 4 |
| BP | GO:0051604 | protein maturation | 7/87 | 378/18493 | 0.002028441 | 0.008750566 | 0.004832502 | F2/F7/PRSS3/PLAU/SERPINE1/GRIN2A/CASP3 | 7 |
| BP | GO:0050768 | negative regulation of neurogenesis | 6/87 | 279/18493 | 0.002031935 | 0.008750566 | 0.004832502 | SLC6A4/F2/GSK3B/IL6/APP/TNF | 6 |
| BP | GO:0051188 | cofactor biosynthetic process | 6/87 | 279/18493 | 0.002031935 | 0.008750566 | 0.004832502 | PTGS2/MAOB/SOD1/PPARA/INS/PTGIS | 6 |
| BP | GO:0032606 | type I interferon production | 4/87 | 114/18493 | 0.002057323 | 0.00884763 | 0.004886105 | IL10/RELA/NFKB1/NFKB2 | 4 |
| BP | GO:0001503 | ossification | 7/87 | 380/18493 | 0.002089614 | 0.008959717 | 0.004948006 | PTGS2/ADRB2/BCL2/IL6/ACHE/AKT1/TNF | 7 |
| BP | GO:0006584 | catecholamine metabolic process | 3/87 | 54/18493 | 0.002097815 | 0.008959717 | 0.004948006 | SLC6A3/MAOB/GRIN2A | 3 |
| BP | GO:0009712 | catechol-containing compound metabolic process | 3/87 | 54/18493 | 0.002097815 | 0.008959717 | 0.004948006 | SLC6A3/MAOB/GRIN2A | 3 |
| BP | GO:0034394 | protein localization to cell surface | 3/87 | 54/18493 | 0.002097815 | 0.008959717 | 0.004948006 | BDNF/AKT1/TNF | 3 |
| BP | GO:0060113 | inner ear receptor cell differentiation | 3/87 | 54/18493 | 0.002097815 | 0.008959717 | 0.004948006 | SOD1/GABRA5/GABRB3 | 3 |
| BP | GO:0034764 | positive regulation of transmembrane transport | 5/87 | 192/18493 | 0.002126392 | 0.009069294 | 0.00500852 | ADRB2/F2/KCNH2/INS/AKT1 | 5 |
| BP | GO:0070661 | leukocyte proliferation | 6/87 | 283/18493 | 0.002182108 | 0.009118538 | 0.005035714 | ADA/ERBB2/BCL2/IL10/IL6/CASP3 | 6 |
| BP | GO:0007272 | ensheathment of neurons | 4/87 | 116/18493 | 0.002191955 | 0.009118538 | 0.005035714 | SOD1/ERBB2/PPARD/AKT1 | 4 |
| BP | GO:0008366 | axon ensheathment | 4/87 | 116/18493 | 0.002191955 | 0.009118538 | 0.005035714 | SOD1/ERBB2/PPARD/AKT1 | 4 |
| BP | GO:0018210 | peptidyl-threonine modification | 4/87 | 116/18493 | 0.002191955 | 0.009118538 | 0.005035714 | GSK3B/BCL2/AKT1/APP | 4 |
| BP | GO:0002467 | germinal center formation | 2/87 | 15/18493 | 0.002207614 | 0.009118538 | 0.005035714 | ADA/NFKB2 | 2 |
| BP | GO:0006837 | serotonin transport | 2/87 | 15/18493 | 0.002207614 | 0.009118538 | 0.005035714 | SLC6A4/MAOB | 2 |
| BP | GO:0007635 | chemosensory behavior | 2/87 | 15/18493 | 0.002207614 | 0.009118538 | 0.005035714 | TRPV1/GRIN1 | 2 |
| BP | GO:0030889 | negative regulation of B cell proliferation | 2/87 | 15/18493 | 0.002207614 | 0.009118538 | 0.005035714 | IL10/CASP3 | 2 |
| BP | GO:0032695 | negative regulation of interleukin-12 production | 2/87 | 15/18493 | 0.002207614 | 0.009118538 | 0.005035714 | IL10/NFKB1 | 2 |
| BP | GO:0033631 | cell-cell adhesion mediated by integrin | 2/87 | 15/18493 | 0.002207614 | 0.009118538 | 0.005035714 | ADA/DPP4 | 2 |
| BP | GO:0034116 | positive regulation of heterotypic cell-cell adhesion | 2/87 | 15/18493 | 0.002207614 | 0.009118538 | 0.005035714 | IL10/TNF | 2 |
| BP | GO:0034356 | NAD biosynthesis via nicotinamide riboside salvage pathway | 2/87 | 15/18493 | 0.002207614 | 0.009118538 | 0.005035714 | PTGS2/PTGIS | 2 |
| BP | GO:0035729 | cellular response to hepatocyte growth factor stimulus | 2/87 | 15/18493 | 0.002207614 | 0.009118538 | 0.005035714 | IL10/RELA | 2 |
| BP | GO:0046321 | positive regulation of fatty acid oxidation | 2/87 | 15/18493 | 0.002207614 | 0.009118538 | 0.005035714 | PPARG/PPARA | 2 |
| BP | GO:0050665 | hydrogen peroxide biosynthetic process | 2/87 | 15/18493 | 0.002207614 | 0.009118538 | 0.005035714 | MAOB/SOD1 | 2 |
| BP | GO:0060134 | prepulse inhibition | 2/87 | 15/18493 | 0.002207614 | 0.009118538 | 0.005035714 | SLC6A3/GRIN1 | 2 |
| BP | GO:0060149 | negative regulation of posttranscriptional gene silencing | 2/87 | 15/18493 | 0.002207614 | 0.009118538 | 0.005035714 | ESR1/PPARG | 2 |
| BP | GO:0060967 | negative regulation of gene silencing by RNA | 2/87 | 15/18493 | 0.002207614 | 0.009118538 | 0.005035714 | ESR1/PPARG | 2 |
| BP | GO:0090196 | regulation of chemokine secretion | 2/87 | 15/18493 | 0.002207614 | 0.009118538 | 0.005035714 | LPL/TNF | 2 |
| BP | GO:0090494 | dopamine uptake | 2/87 | 15/18493 | 0.002207614 | 0.009118538 | 0.005035714 | SLC6A2/SLC6A3 | 2 |
| BP | GO:2000010 | positive regulation of protein localization to cell surface | 2/87 | 15/18493 | 0.002207614 | 0.009118538 | 0.005035714 | AKT1/TNF | 2 |
| BP | GO:0046324 | regulation of glucose import | 3/87 | 55/18493 | 0.002211356 | 0.009118538 | 0.005035714 | INS/AKT1/TNF | 3 |
| BP | GO:0070498 | interleukin-1-mediated signaling pathway | 3/87 | 55/18493 | 0.002211356 | 0.009118538 | 0.005035714 | RELA/IL6/NFKB1 | 3 |
| BP | GO:1900408 | negative regulation of cellular response to oxidative stress | 3/87 | 55/18493 | 0.002211356 | 0.009118538 | 0.005035714 | INS/IL10/AKT1 | 3 |
| BP | GO:1903202 | negative regulation of oxidative stress-induced cell death | 3/87 | 55/18493 | 0.002211356 | 0.009118538 | 0.005035714 | INS/IL10/AKT1 | 3 |
| BP | GO:0050867 | positive regulation of cell activation | 7/87 | 384/18493 | 0.002216248 | 0.00912659 | 0.005040161 | ADA/DPP4/BCL2/IL10/IL6/AKT1/APP | 7 |
| BP | GO:0001952 | regulation of cell-matrix adhesion | 4/87 | 117/18493 | 0.002261499 | 0.0092883 | 0.005129465 | GSK3B/PLAU/SERPINE1/BCL2 | 4 |
| BP | GO:0006805 | xenobiotic metabolic process | 4/87 | 117/18493 | 0.002261499 | 0.0092883 | 0.005129465 | PTGS1/AKR1C1/GRIN1/BCHE | 4 |
| BP | GO:0018108 | peptidyl-tyrosine phosphorylation | 7/87 | 387/18493 | 0.002315063 | 0.009488767 | 0.005240173 | HSP90AA1/ERBB2/BDNF/CCK/IL6/APP/ITGB3 | 7 |
| BP | GO:0009746 | response to hexose | 5/87 | 196/18493 | 0.002325617 | 0.009488767 | 0.005240173 | PTGS2/LPL/HMGCR/PPARD/CASP3 | 5 |
| BP | GO:0001541 | ovarian follicle development | 3/87 | 56/18493 | 0.002328644 | 0.009488767 | 0.005240173 | ESR1/SOD1/BCL2 | 3 |
| BP | GO:0019229 | regulation of vasoconstriction | 3/87 | 56/18493 | 0.002328644 | 0.009488767 | 0.005240173 | PTGS2/ADRA1B/AKT1 | 3 |
| BP | GO:0042130 | negative regulation of T cell proliferation | 3/87 | 56/18493 | 0.002328644 | 0.009488767 | 0.005240173 | ERBB2/IL10/CASP3 | 3 |
| BP | GO:0060688 | regulation of morphogenesis of a branching structure | 3/87 | 56/18493 | 0.002328644 | 0.009488767 | 0.005240173 | ESR1/AR/TNF | 3 |
| BP | GO:0002446 | neutrophil mediated immunity | 8/87 | 499/18493 | 0.002405004 | 0.009774765 | 0.005398116 | HSP90AA1/F2/PRSS3/PLAU/MPO/IL6/ALOX5/NFKB1 | 8 |
| BP | GO:1905477 | positive regulation of protein localization to membrane | 4/87 | 119/18493 | 0.002405127 | 0.009774765 | 0.005398116 | ERBB2/BCL2/AKT1/TNF | 4 |
| BP | GO:0018212 | peptidyl-tyrosine modification | 7/87 | 390/18493 | 0.002417254 | 0.009811207 | 0.005418241 | HSP90AA1/ERBB2/BDNF/CCK/IL6/APP/ITGB3 | 7 |
| BP | GO:0042743 | hydrogen peroxide metabolic process | 3/87 | 57/18493 | 0.002449723 | 0.009917066 | 0.005476702 | MAOB/SOD1/MPO | 3 |
| BP | GO:1902883 | negative regulation of response to oxidative stress | 3/87 | 57/18493 | 0.002449723 | 0.009917066 | 0.005476702 | INS/IL10/AKT1 | 3 |
| BP | GO:0030258 | lipid modification | 6/87 | 291/18493 | 0.002507483 | 0.010090409 | 0.00557243 | ESR1/PPARG/ERBB2/PPARA/PPARD/AKT1 | 6 |
| BP | GO:0002739 | regulation of cytokine secretion involved in immune response | 2/87 | 16/18493 | 0.00251529 | 0.010090409 | 0.00557243 | IL10/TNF | 2 |
| BP | GO:0032225 | regulation of synaptic transmission, dopaminergic | 2/87 | 16/18493 | 0.00251529 | 0.010090409 | 0.00557243 | PTGS2/SLC6A4 | 2 |
| BP | GO:0032930 | positive regulation of superoxide anion generation | 2/87 | 16/18493 | 0.00251529 | 0.010090409 | 0.00557243 | SOD1/CRP | 2 |
| BP | GO:0071380 | cellular response to prostaglandin E stimulus | 2/87 | 16/18493 | 0.00251529 | 0.010090409 | 0.00557243 | PPARG/AKT1 | 2 |
| BP | GO:0090335 | regulation of brown fat cell differentiation | 2/87 | 16/18493 | 0.00251529 | 0.010090409 | 0.00557243 | PTGS2/INS | 2 |
| BP | GO:0090493 | catecholamine uptake | 2/87 | 16/18493 | 0.00251529 | 0.010090409 | 0.00557243 | SLC6A2/SLC6A3 | 2 |
| BP | GO:0045766 | positive regulation of angiogenesis | 5/87 | 200/18493 | 0.002538118 | 0.010168845 | 0.005615747 | PTGS2/SERPINE1/IL10/SP1/PTGIS | 5 |
| BP | GO:0008637 | apoptotic mitochondrial changes | 4/87 | 121/18493 | 0.002554918 | 0.010222964 | 0.005645634 | GSK3B/CCK/BCL2/AKT1 | 4 |
| BP | GO:0001836 | release of cytochrome c from mitochondria | 3/87 | 58/18493 | 0.002574633 | 0.010235895 | 0.005652775 | CCK/BCL2/AKT1 | 3 |
| BP | GO:0010518 | positive regulation of phospholipase activity | 3/87 | 58/18493 | 0.002574633 | 0.010235895 | 0.005652775 | ESR1/BDNF/P2RY12 | 3 |
| BP | GO:0030520 | intracellular estrogen receptor signaling pathway | 3/87 | 58/18493 | 0.002574633 | 0.010235895 | 0.005652775 | ESR1/AR/ESR2 | 3 |
| BP | GO:0031646 | positive regulation of neurological system process | 3/87 | 58/18493 | 0.002574633 | 0.010235895 | 0.005652775 | ADRB2/CCK/GRIN1 | 3 |
| BP | GO:0045453 | bone resorption | 3/87 | 58/18493 | 0.002574633 | 0.010235895 | 0.005652775 | ADRB2/IL6/ITGB3 | 3 |
| BP | GO:0034284 | response to monosaccharide | 5/87 | 201/18493 | 0.002593376 | 0.010297229 | 0.005686647 | PTGS2/LPL/HMGCR/PPARD/CASP3 | 5 |
| BP | GO:0010595 | positive regulation of endothelial cell migration | 4/87 | 122/18493 | 0.002632165 | 0.010437896 | 0.00576433 | PTGS2/SP1/AKT1/ITGB3 | 4 |
| BP | GO:1990778 | protein localization to cell periphery | 6/87 | 294/18493 | 0.002638471 | 0.010449556 | 0.005770769 | AR/INS/GRIN1/GRIN2A/AKT1/TNF | 6 |
| BP | GO:0034113 | heterotypic cell-cell adhesion | 3/87 | 59/18493 | 0.002703414 | 0.010678159 | 0.005897015 | IL10/ITGB3/TNF | 3 |
| BP | GO:0042490 | mechanoreceptor differentiation | 3/87 | 59/18493 | 0.002703414 | 0.010678159 | 0.005897015 | SOD1/GABRA5/GABRB3 | 3 |
| BP | GO:0043903 | regulation of symbiosis, encompassing mutualism through parasitism | 5/87 | 203/18493 | 0.002706509 | 0.010678159 | 0.005897015 | MPO/BCL2/IL10/SP1/TNF | 5 |
| BP | GO:0050715 | positive regulation of cytokine secretion | 4/87 | 123/18493 | 0.002711002 | 0.010682311 | 0.005899308 | LPL/INS/IL10/TNF | 4 |
| BP | GO:0032869 | cellular response to insulin stimulus | 5/87 | 204/18493 | 0.002764399 | 0.01087891 | 0.00600788 | PPARG/INS/SP1/RELA/AKT1 | 5 |
| BP | GO:0051961 | negative regulation of nervous system development | 6/87 | 298/18493 | 0.002821051 | 0.011058036 | 0.006106803 | SLC6A4/F2/GSK3B/IL6/APP/TNF | 6 |
| BP | GO:0010744 | positive regulation of macrophage derived foam cell differentiation | 2/87 | 17/18493 | 0.002841969 | 0.011058036 | 0.006106803 | LPL/NFKB1 | 2 |
| BP | GO:0035728 | response to hepatocyte growth factor | 2/87 | 17/18493 | 0.002841969 | 0.011058036 | 0.006106803 | IL10/RELA | 2 |
| BP | GO:0045019 | negative regulation of nitric oxide biosynthetic process | 2/87 | 17/18493 | 0.002841969 | 0.011058036 | 0.006106803 | IL10/PTGIS | 2 |
| BP | GO:0045725 | positive regulation of glycogen biosynthetic process | 2/87 | 17/18493 | 0.002841969 | 0.011058036 | 0.006106803 | INS/AKT1 | 2 |
| BP | GO:0090195 | chemokine secretion | 2/87 | 17/18493 | 0.002841969 | 0.011058036 | 0.006106803 | LPL/TNF | 2 |
| BP | GO:1902931 | negative regulation of alcohol biosynthetic process | 2/87 | 17/18493 | 0.002841969 | 0.011058036 | 0.006106803 | SOD1/NFKB1 | 2 |
| BP | GO:1903978 | regulation of microglial cell activation | 2/87 | 17/18493 | 0.002841969 | 0.011058036 | 0.006106803 | APP/TNF | 2 |
| BP | GO:1904406 | negative regulation of nitric oxide metabolic process | 2/87 | 17/18493 | 0.002841969 | 0.011058036 | 0.006106803 | IL10/PTGIS | 2 |
| BP | GO:0071229 | cellular response to acid chemical | 5/87 | 206/18493 | 0.002882867 | 0.011203131 | 0.006186931 | SLC6A4/PPARG/AKR1C1/AKT1/TNF | 5 |
| BP | GO:0071241 | cellular response to inorganic substance | 5/87 | 207/18493 | 0.00294346 | 0.011424305 | 0.006309074 | PTGS2/SCN5A/SOD1/AKT1/APP | 5 |
| BP | GO:0043280 | positive regulation of cysteine-type endopeptidase activity involved in apoptotic process | 4/87 | 126/18493 | 0.002957212 | 0.011463349 | 0.006330637 | PPARG/CCK/CASP3/TNF | 4 |
| BP | GO:2000027 | regulation of organ morphogenesis | 5/87 | 208/18493 | 0.00300497 | 0.011633956 | 0.006424854 | ESR1/AR/BCL2/VDR/TNF | 5 |
| BP | GO:0009896 | positive regulation of catabolic process | 7/87 | 407/18493 | 0.003063835 | 0.011846135 | 0.006542031 | HSP90AA1/ADRB2/GSK3B/PPARA/INS/AKT1/TNF | 7 |
| BP | GO:0010810 | regulation of cell-substrate adhesion | 5/87 | 209/18493 | 0.003067405 | 0.011846135 | 0.006542031 | GSK3B/PLAU/SERPINE1/PLG/BCL2 | 5 |
| BP | GO:0019748 | secondary metabolic process | 3/87 | 62/18493 | 0.00311338 | 0.01200875 | 0.006631835 | BCL2/SP1/AKR1C1 | 3 |
| BP | GO:0035270 | endocrine system development | 4/87 | 128/18493 | 0.003129595 | 0.012041379 | 0.006649854 | SLC6A3/GSK3B/IL6/AKT1 | 4 |
| BP | GO:0050709 | negative regulation of protein secretion | 4/87 | 128/18493 | 0.003129595 | 0.012041379 | 0.006649854 | HMGCR/INS/IL10/TNF | 4 |
| BP | GO:0002544 | chronic inflammatory response | 2/87 | 18/18493 | 0.003187468 | 0.012099128 | 0.006681746 | IL10/TNF | 2 |
| BP | GO:0010224 | response to UV-B | 2/87 | 18/18493 | 0.003187468 | 0.012099128 | 0.006681746 | BCL2/RELA | 2 |
| BP | GO:0031293 | membrane protein intracellular domain proteolysis | 2/87 | 18/18493 | 0.003187468 | 0.012099128 | 0.006681746 | RELA/NFKB1 | 2 |
| BP | GO:0031998 | regulation of fatty acid beta-oxidation | 2/87 | 18/18493 | 0.003187468 | 0.012099128 | 0.006681746 | PPARA/AKT1 | 2 |
| BP | GO:0032095 | regulation of response to food | 2/87 | 18/18493 | 0.003187468 | 0.012099128 | 0.006681746 | PPARA/CCK | 2 |
| BP | GO:0033189 | response to vitamin A | 2/87 | 18/18493 | 0.003187468 | 0.012099128 | 0.006681746 | PPARG/PPARD | 2 |
| BP | GO:0051023 | regulation of immunoglobulin secretion | 2/87 | 18/18493 | 0.003187468 | 0.012099128 | 0.006681746 | IL6/TNF | 2 |
| BP | GO:0060252 | positive regulation of glial cell proliferation | 2/87 | 18/18493 | 0.003187468 | 0.012099128 | 0.006681746 | IL6/TNF | 2 |
| BP | GO:0060749 | mammary gland alveolus development | 2/87 | 18/18493 | 0.003187468 | 0.012099128 | 0.006681746 | ESR1/AR | 2 |
| BP | GO:0061377 | mammary gland lobule development | 2/87 | 18/18493 | 0.003187468 | 0.012099128 | 0.006681746 | ESR1/AR | 2 |
| BP | GO:1900409 | positive regulation of cellular response to oxidative stress | 2/87 | 18/18493 | 0.003187468 | 0.012099128 | 0.006681746 | SOD1/TNF | 2 |
| BP | GO:0002637 | regulation of immunoglobulin production | 3/87 | 63/18493 | 0.003258036 | 0.012306814 | 0.00679644 | IL10/IL6/TNF | 3 |
| BP | GO:0010676 | positive regulation of cellular carbohydrate metabolic process | 3/87 | 63/18493 | 0.003258036 | 0.012306814 | 0.00679644 | PPARA/INS/AKT1 | 3 |
| BP | GO:0046323 | glucose import | 3/87 | 63/18493 | 0.003258036 | 0.012306814 | 0.00679644 | INS/AKT1/TNF | 3 |
| BP | GO:0090181 | regulation of cholesterol metabolic process | 3/87 | 63/18493 | 0.003258036 | 0.012306814 | 0.00679644 | SOD1/HMGCR/SP1 | 3 |
| BP | GO:0071375 | cellular response to peptide hormone stimulus | 6/87 | 307/18493 | 0.003266414 | 0.012323471 | 0.006805639 | PPARG/INS/SP1/RELA/AKT1/NFKB1 | 6 |
| BP | GO:0050921 | positive regulation of chemotaxis | 4/87 | 130/18493 | 0.003308722 | 0.012452827 | 0.006877076 | F7/SERPINE1/IL6/P2RY12 | 4 |
| BP | GO:0071347 | cellular response to interleukin-1 | 4/87 | 130/18493 | 0.003308722 | 0.012452827 | 0.006877076 | RELA/IL6/NFKB1/PTGIS | 4 |
| BP | GO:1903039 | positive regulation of leukocyte cell-cell adhesion | 5/87 | 214/18493 | 0.00339373 | 0.012757303 | 0.007045223 | ADA/DPP4/IL6/AKT1/TNF | 5 |
| BP | GO:0050777 | negative regulation of immune response | 4/87 | 131/18493 | 0.003400854 | 0.012759916 | 0.007046666 | PPARG/INS/IL10/TNF | 4 |
| BP | GO:0050795 | regulation of behavior | 3/87 | 64/18493 | 0.003406754 | 0.012759916 | 0.007046666 | ADA/INS/CCK | 3 |
| BP | GO:0072577 | endothelial cell apoptotic process | 3/87 | 64/18493 | 0.003406754 | 0.012759916 | 0.007046666 | SERPINE1/IL10/TNF | 3 |
| BP | GO:0032635 | interleukin-6 production | 4/87 | 132/18493 | 0.003494719 | 0.013073616 | 0.007219907 | NOS2/IL10/IL6/TNF | 4 |
| BP | GO:0002363 | alpha-beta T cell lineage commitment | 2/87 | 19/18493 | 0.003551607 | 0.013159593 | 0.007267388 | BCL2/IL6 | 2 |
| BP | GO:0002374 | cytokine secretion involved in immune response | 2/87 | 19/18493 | 0.003551607 | 0.013159593 | 0.007267388 | IL10/TNF | 2 |
| BP | GO:0002902 | regulation of B cell apoptotic process | 2/87 | 19/18493 | 0.003551607 | 0.013159593 | 0.007267388 | ADA/IL10 | 2 |
| BP | GO:0017000 | antibiotic biosynthetic process | 2/87 | 19/18493 | 0.003551607 | 0.013159593 | 0.007267388 | MAOB/SOD1 | 2 |
| BP | GO:0035994 | response to muscle stretch | 2/87 | 19/18493 | 0.003551607 | 0.013159593 | 0.007267388 | RELA/NFKB1 | 2 |
| BP | GO:0070875 | positive regulation of glycogen metabolic process | 2/87 | 19/18493 | 0.003551607 | 0.013159593 | 0.007267388 | INS/AKT1 | 2 |
| BP | GO:0097062 | dendritic spine maintenance | 2/87 | 19/18493 | 0.003551607 | 0.013159593 | 0.007267388 | INS/GRIN2B | 2 |
| BP | GO:1902176 | negative regulation of oxidative stress-induced intrinsic apoptotic signaling pathway | 2/87 | 19/18493 | 0.003551607 | 0.013159593 | 0.007267388 | INS/AKT1 | 2 |
| BP | GO:0032481 | positive regulation of type I interferon production | 3/87 | 65/18493 | 0.003559569 | 0.013173377 | 0.007275 | RELA/NFKB1/NFKB2 | 3 |
| BP | GO:0060562 | epithelial tube morphogenesis | 6/87 | 313/18493 | 0.00359122 | 0.01327469 | 0.00733095 | ESR1/AR/PGR/BCL2/VDR/TNF | 6 |
| BP | GO:0008016 | regulation of heart contraction | 5/87 | 217/18493 | 0.003601131 | 0.013295497 | 0.007342441 | CHRM2/SCN5A/ADA/ADRA1B/KCNH2 | 5 |
| BP | GO:0010675 | regulation of cellular carbohydrate metabolic process | 4/87 | 134/18493 | 0.00368771 | 0.013582846 | 0.007501129 | GSK3B/PPARA/INS/AKT1 | 4 |
| BP | GO:0046631 | alpha-beta T cell activation | 4/87 | 134/18493 | 0.00368771 | 0.013582846 | 0.007501129 | ADA/INS/BCL2/IL6 | 4 |
| BP | GO:0002792 | negative regulation of peptide secretion | 4/87 | 135/18493 | 0.003786867 | 0.013931544 | 0.007693698 | HMGCR/INS/IL10/TNF | 4 |
| BP | GO:0007617 | mating behavior | 2/87 | 20/18493 | 0.003934208 | 0.014306664 | 0.007900858 | GRIN1/APP | 2 |
| BP | GO:0007620 | copulation | 2/87 | 20/18493 | 0.003934208 | 0.014306664 | 0.007900858 | ADA/SLC6A4 | 2 |
| BP | GO:0010042 | response to manganese ion | 2/87 | 20/18493 | 0.003934208 | 0.014306664 | 0.007900858 | PTGS2/APP | 2 |
| BP | GO:0032098 | regulation of appetite | 2/87 | 20/18493 | 0.003934208 | 0.014306664 | 0.007900858 | PPARA/CCK | 2 |
| BP | GO:0032682 | negative regulation of chemokine production | 2/87 | 20/18493 | 0.003934208 | 0.014306664 | 0.007900858 | IL10/IL6 | 2 |
| BP | GO:0032928 | regulation of superoxide anion generation | 2/87 | 20/18493 | 0.003934208 | 0.014306664 | 0.007900858 | SOD1/CRP | 2 |
| BP | GO:0042053 | regulation of dopamine metabolic process | 2/87 | 20/18493 | 0.003934208 | 0.014306664 | 0.007900858 | SLC6A3/MAOB | 2 |
| BP | GO:0042069 | regulation of catecholamine metabolic process | 2/87 | 20/18493 | 0.003934208 | 0.014306664 | 0.007900858 | SLC6A3/MAOB | 2 |
| BP | GO:1902884 | positive regulation of response to oxidative stress | 2/87 | 20/18493 | 0.003934208 | 0.014306664 | 0.007900858 | SOD1/TNF | 2 |
| BP | GO:0071214 | cellular response to abiotic stimulus | 6/87 | 319/18493 | 0.003939516 | 0.014306664 | 0.007900858 | PTGS2/TRPV1/AKT1/SLC2A1/CASP3/NFKB1 | 6 |
| BP | GO:0104004 | cellular response to environmental stimulus | 6/87 | 319/18493 | 0.003939516 | 0.014306664 | 0.007900858 | PTGS2/TRPV1/AKT1/SLC2A1/CASP3/NFKB1 | 6 |
| BP | GO:0007160 | cell-matrix adhesion | 5/87 | 222/18493 | 0.003966831 | 0.014372242 | 0.007937073 | GSK3B/PLAU/SERPINE1/BCL2/ITGB3 | 5 |
| BP | GO:0010594 | regulation of endothelial cell migration | 5/87 | 222/18493 | 0.003966831 | 0.014372242 | 0.007937073 | PTGS2/PPARG/SP1/AKT1/ITGB3 | 5 |
| BP | GO:0033692 | cellular polysaccharide biosynthetic process | 3/87 | 68/18493 | 0.004042948 | 0.014580327 | 0.008051988 | GSK3B/INS/AKT1 | 3 |
| BP | GO:0042698 | ovulation cycle | 3/87 | 68/18493 | 0.004042948 | 0.014580327 | 0.008051988 | ESR1/PGR/CASP3 | 3 |
| BP | GO:0051705 | multi-organism behavior | 3/87 | 68/18493 | 0.004042948 | 0.014580327 | 0.008051988 | SLC6A4/GRIN1/APP | 3 |
| BP | GO:0009743 | response to carbohydrate | 5/87 | 223/18493 | 0.004043047 | 0.014580327 | 0.008051988 | PTGS2/LPL/HMGCR/PPARD/CASP3 | 5 |
| BP | GO:0016042 | lipid catabolic process | 6/87 | 321/18493 | 0.004061009 | 0.014628114 | 0.008078379 | LPL/PPARA/PPARD/INS/AKT1/TNF | 6 |
| BP | GO:0032147 | activation of protein kinase activity | 6/87 | 323/18493 | 0.004185263 | 0.015058216 | 0.008315903 | ADRB2/SOD1/INS/AKT1/ITGB3/TNF | 6 |
| BP | GO:0007189 | adenylate cyclase-activating G-protein coupled receptor signaling pathway | 4/87 | 139/18493 | 0.004201601 | 0.015099503 | 0.008338704 | ADRA1B/ADRB2/ADRA1D/PTGIR | 4 |
| BP | GO:0010517 | regulation of phospholipase activity | 3/87 | 69/18493 | 0.004212496 | 0.015103697 | 0.008341019 | ESR1/BDNF/P2RY12 | 3 |
| BP | GO:0048708 | astrocyte differentiation | 3/87 | 69/18493 | 0.004212496 | 0.015103697 | 0.008341019 | F2/APP/TNF | 3 |
| BP | GO:0030336 | negative regulation of cell migration | 6/87 | 324/18493 | 0.004248436 | 0.015214987 | 0.008402479 | ADA/PPARG/SERPINE1/PPARD/BCL2/AKT1 | 6 |
| BP | GO:0048588 | developmental cell growth | 5/87 | 226/18493 | 0.004277989 | 0.015303175 | 0.008451181 | HSP90AA1/GSK3B/BDNF/PPARA/APP | 5 |
| BP | GO:0034250 | positive regulation of cellular amide metabolic process | 4/87 | 140/18493 | 0.004309886 | 0.015330821 | 0.008466449 | ERBB2/IL6/APP/TNF | 4 |
| BP | GO:0002861 | regulation of inflammatory response to antigenic stimulus | 2/87 | 21/18493 | 0.004335092 | 0.015330821 | 0.008466449 | IL10/TNF | 2 |
| BP | GO:0015874 | norepinephrine transport | 2/87 | 21/18493 | 0.004335092 | 0.015330821 | 0.008466449 | SLC6A2/P2RY12 | 2 |
| BP | GO:0030728 | ovulation | 2/87 | 21/18493 | 0.004335092 | 0.015330821 | 0.008466449 | PTGS2/PGR | 2 |
| BP | GO:0035357 | peroxisome proliferator activated receptor signaling pathway | 2/87 | 21/18493 | 0.004335092 | 0.015330821 | 0.008466449 | PPARG/PTGIS | 2 |
| BP | GO:0042135 | neurotransmitter catabolic process | 2/87 | 21/18493 | 0.004335092 | 0.015330821 | 0.008466449 | MAOB/ACHE | 2 |
| BP | GO:0043369 | CD4-positive or CD8-positive, alpha-beta T cell lineage commitment | 2/87 | 21/18493 | 0.004335092 | 0.015330821 | 0.008466449 | BCL2/IL6 | 2 |
| BP | GO:0048305 | immunoglobulin secretion | 2/87 | 21/18493 | 0.004335092 | 0.015330821 | 0.008466449 | IL6/TNF | 2 |
| BP | GO:0050857 | positive regulation of antigen receptor-mediated signaling pathway | 2/87 | 21/18493 | 0.004335092 | 0.015330821 | 0.008466449 | ADA/RELA | 2 |
| BP | GO:0051900 | regulation of mitochondrial depolarization | 2/87 | 21/18493 | 0.004335092 | 0.015330821 | 0.008466449 | CCK/BCL2 | 2 |
| BP | GO:0060560 | developmental growth involved in morphogenesis | 5/87 | 227/18493 | 0.004358427 | 0.015395809 | 0.008502339 | HSP90AA1/ESR1/GSK3B/BDNF/APP | 5 |
| BP | GO:0032945 | negative regulation of mononuclear cell proliferation | 3/87 | 70/18493 | 0.00438631 | 0.015406665 | 0.008508334 | ERBB2/IL10/CASP3 | 3 |
| BP | GO:0048678 | response to axon injury | 3/87 | 70/18493 | 0.00438631 | 0.015406665 | 0.008508334 | SOD1/BCL2/P2RY12 | 3 |
| BP | GO:0050672 | negative regulation of lymphocyte proliferation | 3/87 | 70/18493 | 0.00438631 | 0.015406665 | 0.008508334 | ERBB2/IL10/CASP3 | 3 |
| BP | GO:0060193 | positive regulation of lipase activity | 3/87 | 70/18493 | 0.00438631 | 0.015406665 | 0.008508334 | ESR1/BDNF/P2RY12 | 3 |
| BP | GO:1901224 | positive regulation of NIK/NF-kappaB signaling | 3/87 | 70/18493 | 0.00438631 | 0.015406665 | 0.008508334 | RELA/APP/TNF | 3 |
| BP | GO:0051588 | regulation of neurotransmitter transport | 4/87 | 141/18493 | 0.004420042 | 0.015507605 | 0.008564078 | CHRM2/SLC6A4/MAOB/GSK3B | 4 |
| BP | GO:1904018 | positive regulation of vasculature development | 5/87 | 228/18493 | 0.004439941 | 0.015559837 | 0.008592923 | PTGS2/SERPINE1/IL10/SP1/PTGIS | 5 |
| BP | GO:0007605 | sensory perception of sound | 4/87 | 142/18493 | 0.004532083 | 0.015864847 | 0.008761365 | SOD1/GABRA5/GABRB3/CASP3 | 4 |
| BP | GO:0038127 | ERBB signaling pathway | 4/87 | 143/18493 | 0.004646025 | 0.016245393 | 0.008971521 | HSP90AA1/ERBB2/AKT1/APP | 4 |
| BP | GO:0006635 | fatty acid beta-oxidation | 3/87 | 72/18493 | 0.004746856 | 0.016493218 | 0.009108383 | PPARA/PPARD/AKT1 | 3 |
| BP | GO:0070227 | lymphocyte apoptotic process | 3/87 | 72/18493 | 0.004746856 | 0.016493218 | 0.009108383 | ADA/IL10/AKT1 | 3 |
| BP | GO:0010884 | positive regulation of lipid storage | 2/87 | 22/18493 | 0.004754084 | 0.016493218 | 0.009108383 | LPL/NFKB1 | 2 |
| BP | GO:0032469 | endoplasmic reticulum calcium ion homeostasis | 2/87 | 22/18493 | 0.004754084 | 0.016493218 | 0.009108383 | BCL2/APP | 2 |
| BP | GO:0051000 | positive regulation of nitric-oxide synthase activity | 2/87 | 22/18493 | 0.004754084 | 0.016493218 | 0.009108383 | INS/AKT1 | 2 |
| BP | GO:0071379 | cellular response to prostaglandin stimulus | 2/87 | 22/18493 | 0.004754084 | 0.016493218 | 0.009108383 | PPARG/AKT1 | 2 |
| BP | GO:0071677 | positive regulation of mononuclear cell migration | 2/87 | 22/18493 | 0.004754084 | 0.016493218 | 0.009108383 | SERPINE1/TNF | 2 |
| BP | GO:0002822 | regulation of adaptive immune response based on somatic recombination of immune receptors built from immunoglobulin superfamily domains | 4/87 | 144/18493 | 0.004761883 | 0.016501837 | 0.009113143 | ADA/IL10/IL6/TNF | 4 |
| BP | GO:0042593 | glucose homeostasis | 5/87 | 233/18493 | 0.004863911 | 0.016836616 | 0.009298025 | ADRA1B/PPARG/HMGCR/INS/AKT1 | 5 |
| BP | GO:0019362 | pyridine nucleotide metabolic process | 4/87 | 145/18493 | 0.00487967 | 0.016853587 | 0.009307397 | PTGS2/PPARA/INS/PTGIS | 4 |
| BP | GO:0046496 | nicotinamide nucleotide metabolic process | 4/87 | 145/18493 | 0.00487967 | 0.016853587 | 0.009307397 | PTGS2/PPARA/INS/PTGIS | 4 |
| BP | GO:0005977 | glycogen metabolic process | 3/87 | 73/18493 | 0.00493365 | 0.016983351 | 0.009379059 | GSK3B/INS/AKT1 | 3 |
| BP | GO:0043627 | response to estrogen | 3/87 | 73/18493 | 0.00493365 | 0.016983351 | 0.009379059 | ESR1/PPARG/F7 | 3 |
| BP | GO:1900371 | regulation of purine nucleotide biosynthetic process | 3/87 | 73/18493 | 0.00493365 | 0.016983351 | 0.009379059 | NOS2/PPARA/INS | 3 |
| BP | GO:0030217 | T cell differentiation | 5/87 | 234/18493 | 0.004952041 | 0.017008946 | 0.009393194 | ADA/SOD1/ERBB2/BCL2/IL6 | 5 |
| BP | GO:0033500 | carbohydrate homeostasis | 5/87 | 234/18493 | 0.004952041 | 0.017008946 | 0.009393194 | ADRA1B/PPARG/HMGCR/INS/AKT1 | 5 |
| BP | GO:0071356 | cellular response to tumor necrosis factor | 5/87 | 235/18493 | 0.005041301 | 0.017296396 | 0.009551938 | TRPV1/RELA/AKT1/NFKB1/TNF | 5 |
| BP | GO:0030902 | hindbrain development | 4/87 | 147/18493 | 0.005121091 | 0.017505605 | 0.009667474 | SCN5A/SLC6A4/BCL2/GRIN1 | 4 |
| BP | GO:0006073 | cellular glucan metabolic process | 3/87 | 74/18493 | 0.005124829 | 0.017505605 | 0.009667474 | GSK3B/INS/AKT1 | 3 |
| BP | GO:0030808 | regulation of nucleotide biosynthetic process | 3/87 | 74/18493 | 0.005124829 | 0.017505605 | 0.009667474 | NOS2/PPARA/INS | 3 |
| BP | GO:0044042 | glucan metabolic process | 3/87 | 74/18493 | 0.005124829 | 0.017505605 | 0.009667474 | GSK3B/INS/AKT1 | 3 |
| BP | GO:0002021 | response to dietary excess | 2/87 | 23/18493 | 0.005191008 | 0.017576967 | 0.009706883 | TRPV1/ADRB2 | 2 |
| BP | GO:0010640 | regulation of platelet-derived growth factor receptor signaling pathway | 2/87 | 23/18493 | 0.005191008 | 0.017576967 | 0.009706883 | F7/PTGIR | 2 |
| BP | GO:0032799 | low-density lipoprotein receptor particle metabolic process | 2/87 | 23/18493 | 0.005191008 | 0.017576967 | 0.009706883 | PPARG/ITGB3 | 2 |
| BP | GO:0034695 | response to prostaglandin E | 2/87 | 23/18493 | 0.005191008 | 0.017576967 | 0.009706883 | PPARG/AKT1 | 2 |
| BP | GO:0051043 | regulation of membrane protein ectodomain proteolysis | 2/87 | 23/18493 | 0.005191008 | 0.017576967 | 0.009706883 | IL10/TNF | 2 |
| BP | GO:0051882 | mitochondrial depolarization | 2/87 | 23/18493 | 0.005191008 | 0.017576967 | 0.009706883 | CCK/BCL2 | 2 |
| BP | GO:0060055 | angiogenesis involved in wound healing | 2/87 | 23/18493 | 0.005191008 | 0.017576967 | 0.009706883 | SERPINE1/ITGB3 | 2 |
| BP | GO:0071404 | cellular response to low-density lipoprotein particle stimulus | 2/87 | 23/18493 | 0.005191008 | 0.017576967 | 0.009706883 | PPARG/LPL | 2 |
| BP | GO:2000146 | negative regulation of cell motility | 6/87 | 339/18493 | 0.005283177 | 0.017869571 | 0.009868474 | ADA/PPARG/SERPINE1/PPARD/BCL2/AKT1 | 6 |
| BP | GO:0006695 | cholesterol biosynthetic process | 3/87 | 75/18493 | 0.005320423 | 0.017956428 | 0.009916441 | SOD1/HMGCR/SP1 | 3 |
| BP | GO:0070664 | negative regulation of leukocyte proliferation | 3/87 | 75/18493 | 0.005320423 | 0.017956428 | 0.009916441 | ERBB2/IL10/CASP3 | 3 |
| BP | GO:0043254 | regulation of protein complex assembly | 7/87 | 451/18493 | 0.005355503 | 0.018055196 | 0.009970986 | HSP90AA1/ESR1/GSK3B/INS/CCK/TNF/P2RY12 | 7 |
| BP | GO:0051924 | regulation of calcium ion transport | 5/87 | 239/18493 | 0.005409795 | 0.018218451 | 0.010061143 | PTGS2/F2/BCL2/GRIN1/P2RY12 | 5 |
| BP | GO:0072524 | pyridine-containing compound metabolic process | 4/87 | 150/18493 | 0.005498058 | 0.018495635 | 0.010214218 | PTGS2/PPARA/INS/PTGIS | 4 |
| BP | GO:0051851 | modification by host of symbiont morphology or physiology | 3/87 | 76/18493 | 0.005520459 | 0.018510828 | 0.010222608 | F2/PLG/SP1 | 3 |
| BP | GO:0051937 | catecholamine transport | 3/87 | 76/18493 | 0.005520459 | 0.018510828 | 0.010222608 | SLC6A2/SLC6A3/P2RY12 | 3 |
| BP | GO:1902653 | secondary alcohol biosynthetic process | 3/87 | 76/18493 | 0.005520459 | 0.018510828 | 0.010222608 | SOD1/HMGCR/SP1 | 3 |
| BP | GO:0030856 | regulation of epithelial cell differentiation | 4/87 | 151/18493 | 0.005627727 | 0.018688557 | 0.010320759 | GSK3B/SERPINE1/VDR/TNF | 4 |
| BP | GO:0001783 | B cell apoptotic process | 2/87 | 24/18493 | 0.00564569 | 0.018688557 | 0.010320759 | ADA/IL10 | 2 |
| BP | GO:0002719 | negative regulation of cytokine production involved in immune response | 2/87 | 24/18493 | 0.00564569 | 0.018688557 | 0.010320759 | IL10/TNF | 2 |
| BP | GO:0006359 | regulation of transcription by RNA polymerase III | 2/87 | 24/18493 | 0.00564569 | 0.018688557 | 0.010320759 | AR/ERBB2 | 2 |
| BP | GO:0010288 | response to lead ion | 2/87 | 24/18493 | 0.00564569 | 0.018688557 | 0.010320759 | PTGS2/APP | 2 |
| BP | GO:0031639 | plasminogen activation | 2/87 | 24/18493 | 0.00564569 | 0.018688557 | 0.010320759 | PLAU/SERPINE1 | 2 |
| BP | GO:0032104 | regulation of response to extracellular stimulus | 2/87 | 24/18493 | 0.00564569 | 0.018688557 | 0.010320759 | PPARA/CCK | 2 |
| BP | GO:0032107 | regulation of response to nutrient levels | 2/87 | 24/18493 | 0.00564569 | 0.018688557 | 0.010320759 | PPARA/CCK | 2 |
| BP | GO:0035588 | G-protein coupled purinergic receptor signaling pathway | 2/87 | 24/18493 | 0.00564569 | 0.018688557 | 0.010320759 | ADA/P2RY12 | 2 |
| BP | GO:0098810 | neurotransmitter reuptake | 2/87 | 24/18493 | 0.00564569 | 0.018688557 | 0.010320759 | SLC6A2/SLC6A4 | 2 |
| BP | GO:1900120 | regulation of receptor binding | 2/87 | 24/18493 | 0.00564569 | 0.018688557 | 0.010320759 | BDNF/IL10 | 2 |
| BP | GO:1904385 | cellular response to angiotensin | 2/87 | 24/18493 | 0.00564569 | 0.018688557 | 0.010320759 | RELA/NFKB1 | 2 |
| BP | GO:0009165 | nucleotide biosynthetic process | 6/87 | 344/18493 | 0.005666056 | 0.018716069 | 0.010335953 | PTGS2/ADA/NOS2/PPARA/INS/PTGIS | 6 |
| BP | GO:0030098 | lymphocyte differentiation | 6/87 | 344/18493 | 0.005666056 | 0.018716069 | 0.010335953 | ADA/SOD1/ERBB2/BCL2/IL10/IL6 | 6 |
| BP | GO:0002718 | regulation of cytokine production involved in immune response | 3/87 | 77/18493 | 0.005724965 | 0.018850494 | 0.010410189 | IL10/IL6/TNF | 3 |
| BP | GO:0021675 | nerve development | 3/87 | 77/18493 | 0.005724965 | 0.018850494 | 0.010410189 | BDNF/GABRA5/GABRB3 | 3 |
| BP | GO:0031016 | pancreas development | 3/87 | 77/18493 | 0.005724965 | 0.018850494 | 0.010410189 | GSK3B/IL6/AKT1 | 3 |
| BP | GO:0016999 | antibiotic metabolic process | 4/87 | 152/18493 | 0.005759425 | 0.01890382 | 0.010439638 | MAOB/SOD1/MPO/AKR1C1 | 4 |
| BP | GO:0030168 | platelet activation | 4/87 | 152/18493 | 0.005759425 | 0.01890382 | 0.010439638 | F2/IL6/ITGB3/P2RY12 | 4 |
| BP | GO:0070555 | response to interleukin-1 | 4/87 | 152/18493 | 0.005759425 | 0.01890382 | 0.010439638 | RELA/IL6/NFKB1/PTGIS | 4 |
| BP | GO:0042129 | regulation of T cell proliferation | 4/87 | 153/18493 | 0.005893167 | 0.019322367 | 0.010670781 | ERBB2/IL10/IL6/CASP3 | 4 |
| BP | GO:1901293 | nucleoside phosphate biosynthetic process | 6/87 | 347/18493 | 0.005905331 | 0.019341828 | 0.010681528 | PTGS2/ADA/NOS2/PPARA/INS/PTGIS | 6 |
| BP | GO:0008344 | adult locomotory behavior | 3/87 | 78/18493 | 0.005933966 | 0.0193947 | 0.010710727 | GRIN1/GRIN2D/APP | 3 |
| BP | GO:0031100 | animal organ regeneration | 3/87 | 78/18493 | 0.005933966 | 0.0193947 | 0.010710727 | PPARG/F7/IL10 | 3 |
| BP | GO:0031330 | negative regulation of cellular catabolic process | 5/87 | 245/18493 | 0.005997752 | 0.019561996 | 0.010803116 | PPARA/INS/BCL2/IL10/AKT1 | 5 |
| BP | GO:0050730 | regulation of peptidyl-tyrosine phosphorylation | 5/87 | 245/18493 | 0.005997752 | 0.019561996 | 0.010803116 | BDNF/CCK/IL6/APP/ITGB3 | 5 |
| BP | GO:0006959 | humoral immune response | 6/87 | 349/18493 | 0.006068904 | 0.019773293 | 0.010919805 | F2/PRSS3/CRP/BCL2/IL6/TNF | 6 |
| BP | GO:0034114 | regulation of heterotypic cell-cell adhesion | 2/87 | 25/18493 | 0.006117957 | 0.019891367 | 0.010985011 | IL10/TNF | 2 |
| BP | GO:0045662 | negative regulation of myoblast differentiation | 2/87 | 25/18493 | 0.006117957 | 0.019891367 | 0.010985011 | PPARD/TNF | 2 |
| BP | GO:0043536 | positive regulation of blood vessel endothelial cell migration | 3/87 | 79/18493 | 0.006147489 | 0.019924795 | 0.011003472 | PTGS2/SP1/AKT1 | 3 |
| BP | GO:0046470 | phosphatidylcholine metabolic process | 3/87 | 79/18493 | 0.006147489 | 0.019924795 | 0.011003472 | PON1/CETP/ACHE | 3 |
| BP | GO:1905954 | positive regulation of lipid localization | 3/87 | 79/18493 | 0.006147489 | 0.019924795 | 0.011003472 | LPL/PON1/NFKB1 | 3 |
| BP | GO:0071695 | anatomical structure maturation | 4/87 | 155/18493 | 0.006166836 | 0.019966659 | 0.011026591 | PGR/BCL2/GRIN1/APP | 4 |
| BP | GO:0006732 | coenzyme metabolic process | 6/87 | 351/18493 | 0.006235767 | 0.02016881 | 0.011138229 | PTGS2/HMGCR/PPARA/INS/SLC2A1/PTGIS | 6 |
| BP | GO:0051702 | interaction with symbiont | 3/87 | 80/18493 | 0.006365559 | 0.02056718 | 0.011358229 | F2/PLG/SP1 | 3 |
| BP | GO:0002819 | regulation of adaptive immune response | 4/87 | 157/18493 | 0.006448845 | 0.020814618 | 0.011494877 | ADA/IL10/IL6/TNF | 4 |
| BP | GO:0002460 | adaptive immune response based on somatic recombination of immune receptors built from immunoglobulin superfamily domains | 6/87 | 354/18493 | 0.006492313 | 0.020933158 | 0.011560341 | ADA/CRP/IL10/IL6/NFKB2/TNF | 6 |
| BP | GO:0016126 | sterol biosynthetic process | 3/87 | 81/18493 | 0.0065882 | 0.020999707 | 0.011597092 | SOD1/HMGCR/SP1 | 3 |
| BP | GO:0044106 | cellular amine metabolic process | 3/87 | 81/18493 | 0.0065882 | 0.020999707 | 0.011597092 | SLC6A3/MAOB/INS | 3 |
| BP | GO:0045682 | regulation of epidermis development | 3/87 | 81/18493 | 0.0065882 | 0.020999707 | 0.011597092 | PPARD/VDR/TNF | 3 |
| BP | GO:1901216 | positive regulation of neuron death | 3/87 | 81/18493 | 0.0065882 | 0.020999707 | 0.011597092 | GSK3B/GRIN2B/CASP3 | 3 |
| BP | GO:0043535 | regulation of blood vessel endothelial cell migration | 4/87 | 158/18493 | 0.00659301 | 0.020999707 | 0.011597092 | PTGS2/PPARG/SP1/AKT1 | 4 |
| BP | GO:0002360 | T cell lineage commitment | 2/87 | 26/18493 | 0.006607637 | 0.020999707 | 0.011597092 | BCL2/IL6 | 2 |
| BP | GO:0022011 | myelination in peripheral nervous system | 2/87 | 26/18493 | 0.006607637 | 0.020999707 | 0.011597092 | SOD1/AKT1 | 2 |
| BP | GO:0032292 | peripheral nervous system axon ensheathment | 2/87 | 26/18493 | 0.006607637 | 0.020999707 | 0.011597092 | SOD1/AKT1 | 2 |
| BP | GO:0043032 | positive regulation of macrophage activation | 2/87 | 26/18493 | 0.006607637 | 0.020999707 | 0.011597092 | IL10/APP | 2 |
| BP | GO:0048169 | regulation of long-term neuronal synaptic plasticity | 2/87 | 26/18493 | 0.006607637 | 0.020999707 | 0.011597092 | GRIN1/APP | 2 |
| BP | GO:0048668 | collateral sprouting | 2/87 | 26/18493 | 0.006607637 | 0.020999707 | 0.011597092 | BDNF/APP | 2 |
| BP | GO:0060259 | regulation of feeding behavior | 2/87 | 26/18493 | 0.006607637 | 0.020999707 | 0.011597092 | INS/CCK | 2 |
| BP | GO:0060307 | regulation of ventricular cardiac muscle cell membrane repolarization | 2/87 | 26/18493 | 0.006607637 | 0.020999707 | 0.011597092 | SCN5A/KCNH2 | 2 |
| BP | GO:0060740 | prostate gland epithelium morphogenesis | 2/87 | 26/18493 | 0.006607637 | 0.020999707 | 0.011597092 | ESR1/AR | 2 |
| BP | GO:0006091 | generation of precursor metabolites and energy | 7/87 | 471/18493 | 0.006745375 | 0.021415531 | 0.011826731 | MAOB/NOS2/GSK3B/PPARA/PPARD/INS/AKT1 | 7 |
| BP | GO:0097194 | execution phase of apoptosis | 3/87 | 82/18493 | 0.006815435 | 0.02161586 | 0.011937363 | AKT1/CASP3/PTGIS | 3 |
| BP | GO:0035821 | modification of morphology or physiology of other organism | 4/87 | 161/18493 | 0.007038309 | 0.022282926 | 0.01230575 | NOS2/F2/PLG/SP1 | 4 |
| BP | GO:0007009 | plasma membrane organization | 3/87 | 83/18493 | 0.007047289 | 0.022282926 | 0.01230575 | AR/SOD1/AKT1 | 3 |
| BP | GO:0046849 | bone remodeling | 3/87 | 83/18493 | 0.007047289 | 0.022282926 | 0.01230575 | ADRB2/IL6/ITGB3 | 3 |
| BP | GO:0048710 | regulation of astrocyte differentiation | 2/87 | 27/18493 | 0.007114561 | 0.022381674 | 0.012360284 | F2/APP | 2 |
| BP | GO:0060384 | innervation | 2/87 | 27/18493 | 0.007114561 | 0.022381674 | 0.012360284 | GABRA5/GABRB3 | 2 |
| BP | GO:1902895 | positive regulation of pri-miRNA transcription by RNA polymerase II | 2/87 | 27/18493 | 0.007114561 | 0.022381674 | 0.012360284 | IL10/RELA | 2 |
| BP | GO:1903792 | negative regulation of anion transport | 2/87 | 27/18493 | 0.007114561 | 0.022381674 | 0.012360284 | GABRE/AKT1 | 2 |
| BP | GO:2000463 | positive regulation of excitatory postsynaptic potential | 2/87 | 27/18493 | 0.007114561 | 0.022381674 | 0.012360284 | ADRB2/GRIN1 | 2 |
| BP | GO:0001818 | negative regulation of cytokine production | 5/87 | 256/18493 | 0.007190431 | 0.022597457 | 0.01247945 | F2/IL10/IL6/NFKB1/TNF | 5 |
| BP | GO:0002690 | positive regulation of leukocyte chemotaxis | 3/87 | 84/18493 | 0.007283784 | 0.022821544 | 0.012603202 | F7/SERPINE1/IL6 | 3 |
| BP | GO:0008625 | extrinsic apoptotic signaling pathway via death domain receptors | 3/87 | 84/18493 | 0.007283784 | 0.022821544 | 0.012603202 | SERPINE1/BCL2/TNF | 3 |
| BP | GO:0034103 | regulation of tissue remodeling | 3/87 | 84/18493 | 0.007283784 | 0.022821544 | 0.012603202 | IL6/VDR/ITGB3 | 3 |
| BP | GO:0045165 | cell fate commitment | 5/87 | 257/18493 | 0.007306491 | 0.022869613 | 0.012629748 | AR/PPARG/BCL2/IL6/CASP3 | 5 |
| BP | GO:0002009 | morphogenesis of an epithelium | 7/87 | 479/18493 | 0.007370784 | 0.023047619 | 0.012728052 | ESR1/AR/PGR/BCL2/IL10/VDR/TNF | 7 |
| BP | GO:0002028 | regulation of sodium ion transport | 3/87 | 85/18493 | 0.007524942 | 0.02343525 | 0.012942121 | SCN5A/ADRB2/AKT1 | 3 |
| BP | GO:0045445 | myoblast differentiation | 3/87 | 85/18493 | 0.007524942 | 0.02343525 | 0.012942121 | HMGCR/PPARD/TNF | 3 |
| BP | GO:0071674 | mononuclear cell migration | 3/87 | 85/18493 | 0.007524942 | 0.02343525 | 0.012942121 | SERPINE1/IL6/TNF | 3 |
| BP | GO:1904035 | regulation of epithelial cell apoptotic process | 3/87 | 85/18493 | 0.007524942 | 0.02343525 | 0.012942121 | SERPINE1/IL6/TNF | 3 |
| BP | GO:0007263 | nitric oxide mediated signal transduction | 2/87 | 28/18493 | 0.007638559 | 0.023623232 | 0.013045935 | NOS2/INS | 2 |
| BP | GO:0010894 | negative regulation of steroid biosynthetic process | 2/87 | 28/18493 | 0.007638559 | 0.023623232 | 0.013045935 | SOD1/NFKB1 | 2 |
| BP | GO:0045922 | negative regulation of fatty acid metabolic process | 2/87 | 28/18493 | 0.007638559 | 0.023623232 | 0.013045935 | INS/AKT1 | 2 |
| BP | GO:0048265 | response to pain | 2/87 | 28/18493 | 0.007638559 | 0.023623232 | 0.013045935 | SLC6A2/TRPV1 | 2 |
| BP | GO:0048643 | positive regulation of skeletal muscle tissue development | 2/87 | 28/18493 | 0.007638559 | 0.023623232 | 0.013045935 | HMGCR/BCL2 | 2 |
| BP | GO:0060512 | prostate gland morphogenesis | 2/87 | 28/18493 | 0.007638559 | 0.023623232 | 0.013045935 | ESR1/AR | 2 |
| BP | GO:0072606 | interleukin-8 secretion | 2/87 | 28/18493 | 0.007638559 | 0.023623232 | 0.013045935 | NOS2/CRP | 2 |
| BP | GO:0007416 | synapse assembly | 4/87 | 165/18493 | 0.007662401 | 0.023649856 | 0.013060637 | BDNF/GRIN1/ACHE/APP | 4 |
| BP | GO:0010634 | positive regulation of epithelial cell migration | 4/87 | 165/18493 | 0.007662401 | 0.023649856 | 0.013060637 | PTGS2/SP1/AKT1/ITGB3 | 4 |
| BP | GO:0001942 | hair follicle development | 3/87 | 87/18493 | 0.00802133 | 0.024635241 | 0.013604817 | BCL2/RELA/TNF | 3 |
| BP | GO:0006112 | energy reserve metabolic process | 3/87 | 87/18493 | 0.00802133 | 0.024635241 | 0.013604817 | GSK3B/INS/AKT1 | 3 |
| BP | GO:0042475 | odontogenesis of dentin-containing tooth | 3/87 | 87/18493 | 0.00802133 | 0.024635241 | 0.013604817 | SCN5A/SERPINE1/PPARA | 3 |
| BP | GO:1904705 | regulation of vascular smooth muscle cell proliferation | 3/87 | 87/18493 | 0.00802133 | 0.024635241 | 0.013604817 | PPARG/IL10/TNF | 3 |
| BP | GO:1990874 | vascular smooth muscle cell proliferation | 3/87 | 87/18493 | 0.00802133 | 0.024635241 | 0.013604817 | PPARG/IL10/TNF | 3 |
| BP | GO:0010586 | miRNA metabolic process | 2/87 | 29/18493 | 0.008179463 | 0.024972695 | 0.013791176 | RELA/NFKB1 | 2 |
| BP | GO:0014044 | Schwann cell development | 2/87 | 29/18493 | 0.008179463 | 0.024972695 | 0.013791176 | SOD1/AKT1 | 2 |
| BP | GO:0019098 | reproductive behavior | 2/87 | 29/18493 | 0.008179463 | 0.024972695 | 0.013791176 | GRIN1/APP | 2 |
| BP | GO:0042759 | long-chain fatty acid biosynthetic process | 2/87 | 29/18493 | 0.008179463 | 0.024972695 | 0.013791176 | PTGS2/ALOX5 | 2 |
| BP | GO:0050869 | negative regulation of B cell activation | 2/87 | 29/18493 | 0.008179463 | 0.024972695 | 0.013791176 | IL10/CASP3 | 2 |
| BP | GO:0071295 | cellular response to vitamin | 2/87 | 29/18493 | 0.008179463 | 0.024972695 | 0.013791176 | PPARG/VDR | 2 |
| BP | GO:0016054 | organic acid catabolic process | 5/87 | 265/18493 | 0.008282567 | 0.025237851 | 0.013937608 | NOS2/PPARA/PPARD/PON1/AKT1 | 5 |
| BP | GO:0046395 | carboxylic acid catabolic process | 5/87 | 265/18493 | 0.008282567 | 0.025237851 | 0.013937608 | NOS2/PPARA/PPARD/PON1/AKT1 | 5 |
| BP | GO:0030324 | lung development | 4/87 | 169/18493 | 0.008321938 | 0.025283384 | 0.013962753 | ADA/PGR/HSD11B1/TNF | 4 |
| BP | GO:0043901 | negative regulation of multi-organism process | 4/87 | 169/18493 | 0.008321938 | 0.025283384 | 0.013962753 | ADA/MPO/IL10/TNF | 4 |
| BP | GO:0048469 | cell maturation | 4/87 | 169/18493 | 0.008321938 | 0.025283384 | 0.013962753 | PPARG/PGR/BCL2/APP | 4 |
| BP | GO:0051271 | negative regulation of cellular component movement | 6/87 | 374/18493 | 0.008403324 | 0.02550569 | 0.014085522 | ADA/PPARG/SERPINE1/PPARD/BCL2/AKT1 | 6 |
| BP | GO:0022404 | molting cycle process | 3/87 | 89/18493 | 0.008536617 | 0.025834498 | 0.014267106 | BCL2/RELA/TNF | 3 |
| BP | GO:0022405 | hair cycle process | 3/87 | 89/18493 | 0.008536617 | 0.025834498 | 0.014267106 | BCL2/RELA/TNF | 3 |
| BP | GO:0098773 | skin epidermis development | 3/87 | 89/18493 | 0.008536617 | 0.025834498 | 0.014267106 | BCL2/RELA/TNF | 3 |
| BP | GO:0007202 | activation of phospholipase C activity | 2/87 | 30/18493 | 0.008737105 | 0.026338555 | 0.014545472 | BDNF/P2RY12 | 2 |
| BP | GO:0045939 | negative regulation of steroid metabolic process | 2/87 | 30/18493 | 0.008737105 | 0.026338555 | 0.014545472 | SOD1/NFKB1 | 2 |
| BP | GO:0099625 | ventricular cardiac muscle cell membrane repolarization | 2/87 | 30/18493 | 0.008737105 | 0.026338555 | 0.014545472 | SCN5A/KCNH2 | 2 |
| BP | GO:1901661 | quinone metabolic process | 2/87 | 30/18493 | 0.008737105 | 0.026338555 | 0.014545472 | HMGCR/AKR1C1 | 2 |
| BP | GO:0003073 | regulation of systemic arterial blood pressure | 3/87 | 90/18493 | 0.008801396 | 0.026455308 | 0.014609949 | ADRA1B/ADRB2/AR | 3 |
| BP | GO:0009308 | amine metabolic process | 3/87 | 90/18493 | 0.008801396 | 0.026455308 | 0.014609949 | SLC6A3/MAOB/INS | 3 |
| BP | GO:1901184 | regulation of ERBB signaling pathway | 3/87 | 90/18493 | 0.008801396 | 0.026455308 | 0.014609949 | ERBB2/AKT1/APP | 3 |
| BP | GO:0071900 | regulation of protein serine/threonine kinase activity | 7/87 | 496/18493 | 0.008841795 | 0.026551038 | 0.014662816 | ADRB2/SOD1/ERBB2/HMGCR/AKT1/CASP3/TNF | 7 |
| BP | GO:0030323 | respiratory tube development | 4/87 | 173/18493 | 0.00901771 | 0.02705313 | 0.014940096 | ADA/PGR/HSD11B1/TNF | 4 |
| BP | GO:0044070 | regulation of anion transport | 3/87 | 91/18493 | 0.009070956 | 0.027162516 | 0.015000505 | CCK/GABRE/AKT1 | 3 |
| BP | GO:0021700 | developmental maturation | 5/87 | 271/18493 | 0.009071668 | 0.027162516 | 0.015000505 | PPARG/PGR/BCL2/GRIN1/APP | 5 |
| BP | GO:0030431 | sleep | 2/87 | 31/18493 | 0.009311321 | 0.027719704 | 0.015308212 | ADA/GRIN2A | 2 |
| BP | GO:0034694 | response to prostaglandin | 2/87 | 31/18493 | 0.009311321 | 0.027719704 | 0.015308212 | PPARG/AKT1 | 2 |
| BP | GO:0038128 | ERBB2 signaling pathway | 2/87 | 31/18493 | 0.009311321 | 0.027719704 | 0.015308212 | HSP90AA1/ERBB2 | 2 |
| BP | GO:0042554 | superoxide anion generation | 2/87 | 31/18493 | 0.009311321 | 0.027719704 | 0.015308212 | SOD1/CRP | 2 |
| BP | GO:0048011 | neurotrophin TRK receptor signaling pathway | 2/87 | 31/18493 | 0.009311321 | 0.027719704 | 0.015308212 | BDNF/CASP3 | 2 |
| BP | GO:0099623 | regulation of cardiac muscle cell membrane repolarization | 2/87 | 31/18493 | 0.009311321 | 0.027719704 | 0.015308212 | SCN5A/KCNH2 | 2 |
| BP | GO:0042632 | cholesterol homeostasis | 3/87 | 92/18493 | 0.009345316 | 0.027794259 | 0.015349385 | LPL/CETP/AKR1C1 | 3 |
| BP | GO:0051348 | negative regulation of transferase activity | 5/87 | 274/18493 | 0.009485101 | 0.028183003 | 0.015564069 | PPARG/GSK3B/HMGCR/AKT1/CASP3 | 5 |
| BP | GO:0007589 | body fluid secretion | 3/87 | 93/18493 | 0.009624493 | 0.02854255 | 0.015762629 | ADA/SLC6A3/VDR | 3 |
| BP | GO:0055092 | sterol homeostasis | 3/87 | 93/18493 | 0.009624493 | 0.02854255 | 0.015762629 | LPL/CETP/AKR1C1 | 3 |
| BP | GO:0040013 | negative regulation of locomotion | 6/87 | 386/18493 | 0.009727918 | 0.028821743 | 0.015916813 | ADA/PPARG/SERPINE1/PPARD/BCL2/AKT1 | 6 |
| BP | GO:0010039 | response to iron ion | 2/87 | 32/18493 | 0.009901945 | 0.02906026 | 0.016048534 | SLC6A3/BCL2 | 2 |
| BP | GO:0010574 | regulation of vascular endothelial growth factor production | 2/87 | 32/18493 | 0.009901945 | 0.02906026 | 0.016048534 | PTGS2/IL6 | 2 |
| BP | GO:0014002 | astrocyte development | 2/87 | 32/18493 | 0.009901945 | 0.02906026 | 0.016048534 | APP/TNF | 2 |
| BP | GO:0035587 | purinergic receptor signaling pathway | 2/87 | 32/18493 | 0.009901945 | 0.02906026 | 0.016048534 | ADA/P2RY12 | 2 |
| BP | GO:0042311 | vasodilation | 2/87 | 32/18493 | 0.009901945 | 0.02906026 | 0.016048534 | ADRB2/SOD1 | 2 |
| BP | GO:1901030 | positive regulation of mitochondrial outer membrane permeabilization involved in apoptotic signaling pathway | 2/87 | 32/18493 | 0.009901945 | 0.02906026 | 0.016048534 | GSK3B/BCL2 | 2 |
| BP | GO:0032092 | positive regulation of protein binding | 3/87 | 94/18493 | 0.009908503 | 0.02906026 | 0.016048534 | GSK3B/BDNF/APP | 3 |
| BP | GO:0050830 | defense response to Gram-positive bacterium | 3/87 | 94/18493 | 0.009908503 | 0.02906026 | 0.016048534 | CRP/IL6/TNF | 3 |
| BP | GO:0070301 | cellular response to hydrogen peroxide | 3/87 | 94/18493 | 0.009908503 | 0.02906026 | 0.016048534 | IL10/RELA/IL6 | 3 |
| BP | GO:1900542 | regulation of purine nucleotide metabolic process | 3/87 | 94/18493 | 0.009908503 | 0.02906026 | 0.016048534 | NOS2/PPARA/INS | 3 |
| BP | GO:0051146 | striated muscle cell differentiation | 5/87 | 277/18493 | 0.009911374 | 0.02906026 | 0.016048534 | BDNF/PPARA/BCL2/AKT1/CASP3 | 5 |
| BP | GO:0042098 | T cell proliferation | 4/87 | 178/18493 | 0.009939539 | 0.029115347 | 0.016078956 | ERBB2/IL10/IL6/CASP3 | 4 |
| BP | GO:0044262 | cellular carbohydrate metabolic process | 5/87 | 278/18493 | 0.01005635 | 0.029429753 | 0.016252587 | GSK3B/PPARA/INS/AKT1/SLC2A1 | 5 |
| BP | GO:0002367 | cytokine production involved in immune response | 3/87 | 95/18493 | 0.010197362 | 0.029814321 | 0.016464964 | IL10/IL6/TNF | 3 |
| BP | GO:0033028 | myeloid cell apoptotic process | 2/87 | 33/18493 | 0.010508815 | 0.030523734 | 0.016856738 | BCL2/IL6 | 2 |
| BP | GO:0040018 | positive regulation of multicellular organism growth | 2/87 | 33/18493 | 0.010508815 | 0.030523734 | 0.016856738 | SLC6A3/BCL2 | 2 |
| BP | GO:0045730 | respiratory burst | 2/87 | 33/18493 | 0.010508815 | 0.030523734 | 0.016856738 | MPO/INS | 2 |
| BP | GO:0046326 | positive regulation of glucose import | 2/87 | 33/18493 | 0.010508815 | 0.030523734 | 0.016856738 | INS/AKT1 | 2 |
| BP | GO:0051354 | negative regulation of oxidoreductase activity | 2/87 | 33/18493 | 0.010508815 | 0.030523734 | 0.016856738 | INS/NFKB1 | 2 |
| BP | GO:0060251 | regulation of glial cell proliferation | 2/87 | 33/18493 | 0.010508815 | 0.030523734 | 0.016856738 | IL6/TNF | 2 |
| BP | GO:0071312 | cellular response to alkaloid | 2/87 | 33/18493 | 0.010508815 | 0.030523734 | 0.016856738 | TRPV1/CASP3 | 2 |
| BP | GO:0051224 | negative regulation of protein transport | 4/87 | 181/18493 | 0.010520979 | 0.030530505 | 0.016860477 | HMGCR/INS/IL10/TNF | 4 |
| BP | GO:0043534 | blood vessel endothelial cell migration | 4/87 | 182/18493 | 0.010719583 | 0.031077783 | 0.017162712 | PTGS2/PPARG/SP1/AKT1 | 4 |
| BP | GO:0042752 | regulation of circadian rhythm | 3/87 | 97/18493 | 0.01078969 | 0.031251853 | 0.017258842 | ADA/PPARG/PPARA | 3 |
| BP | GO:0010632 | regulation of epithelial cell migration | 5/87 | 284/18493 | 0.010956987 | 0.031677324 | 0.017493808 | PTGS2/PPARG/SP1/AKT1/ITGB3 | 5 |
| BP | GO:0051403 | stress-activated MAPK cascade | 5/87 | 284/18493 | 0.010956987 | 0.031677324 | 0.017493808 | HMGCR/AKT1/APP/NFKB1/TNF | 5 |
| BP | GO:0006140 | regulation of nucleotide metabolic process | 3/87 | 98/18493 | 0.011093188 | 0.031797737 | 0.017560306 | NOS2/PPARA/INS | 3 |
| BP | GO:0051591 | response to cAMP | 3/87 | 98/18493 | 0.011093188 | 0.031797737 | 0.017560306 | SLC6A3/RELA/APP | 3 |
| BP | GO:0050866 | negative regulation of cell activation | 4/87 | 184/18493 | 0.011124042 | 0.031797737 | 0.017560306 | F2/ERBB2/IL10/CASP3 | 4 |
| BP | GO:1904950 | negative regulation of establishment of protein localization | 4/87 | 184/18493 | 0.011124042 | 0.031797737 | 0.017560306 | HMGCR/INS/IL10/TNF | 4 |
| BP | GO:0002701 | negative regulation of production of molecular mediator of immune response | 2/87 | 34/18493 | 0.011131768 | 0.031797737 | 0.017560306 | IL10/TNF | 2 |
| BP | GO:0010573 | vascular endothelial growth factor production | 2/87 | 34/18493 | 0.011131768 | 0.031797737 | 0.017560306 | PTGS2/IL6 | 2 |
| BP | GO:0010765 | positive regulation of sodium ion transport | 2/87 | 34/18493 | 0.011131768 | 0.031797737 | 0.017560306 | SCN5A/AKT1 | 2 |
| BP | GO:0014047 | glutamate secretion | 2/87 | 34/18493 | 0.011131768 | 0.031797737 | 0.017560306 | TRPV1/CCK | 2 |
| BP | GO:0032148 | activation of protein kinase B activity | 2/87 | 34/18493 | 0.011131768 | 0.031797737 | 0.017560306 | INS/AKT1 | 2 |
| BP | GO:0043276 | anoikis | 2/87 | 34/18493 | 0.011131768 | 0.031797737 | 0.017560306 | BCL2/AKT1 | 2 |
| BP | GO:0048536 | spleen development | 2/87 | 34/18493 | 0.011131768 | 0.031797737 | 0.017560306 | BCL2/NFKB2 | 2 |
| BP | GO:0048566 | embryonic digestive tract development | 2/87 | 34/18493 | 0.011131768 | 0.031797737 | 0.017560306 | ADA/TNF | 2 |
| BP | GO:0071392 | cellular response to estradiol stimulus | 2/87 | 34/18493 | 0.011131768 | 0.031797737 | 0.017560306 | ESR1/IL10 | 2 |
| BP | GO:0002027 | regulation of heart rate | 3/87 | 99/18493 | 0.011401594 | 0.032508676 | 0.017952923 | SCN5A/ADA/KCNH2 | 3 |
| BP | GO:0044264 | cellular polysaccharide metabolic process | 3/87 | 99/18493 | 0.011401594 | 0.032508676 | 0.017952923 | GSK3B/INS/AKT1 | 3 |
| BP | GO:0019933 | cAMP-mediated signaling | 4/87 | 186/18493 | 0.011538237 | 0.032837972 | 0.018134776 | ADRA1B/ADRB2/ADRA1D/PTGIR | 4 |
| BP | GO:0050731 | positive regulation of peptidyl-tyrosine phosphorylation | 4/87 | 186/18493 | 0.011538237 | 0.032837972 | 0.018134776 | BDNF/CCK/IL6/ITGB3 | 4 |
| BP | GO:0002824 | positive regulation of adaptive immune response based on somatic recombination of immune receptors built from immunoglobulin superfamily domains | 3/87 | 100/18493 | 0.011714921 | 0.033165014 | 0.018315385 | ADA/IL6/TNF | 3 |
| BP | GO:0010522 | regulation of calcium ion transport into cytosol | 3/87 | 100/18493 | 0.011714921 | 0.033165014 | 0.018315385 | F2/BCL2/GRIN1 | 3 |
| BP | GO:0050848 | regulation of calcium-mediated signaling | 3/87 | 100/18493 | 0.011714921 | 0.033165014 | 0.018315385 | ADA/GSK3B/TNF | 3 |
| BP | GO:0033280 | response to vitamin D | 2/87 | 35/18493 | 0.011770643 | 0.033165014 | 0.018315385 | PTGS2/VDR | 2 |
| BP | GO:0043243 | positive regulation of protein complex disassembly | 2/87 | 35/18493 | 0.011770643 | 0.033165014 | 0.018315385 | ADRB2/TNF | 2 |
| BP | GO:0043368 | positive T cell selection | 2/87 | 35/18493 | 0.011770643 | 0.033165014 | 0.018315385 | BCL2/IL6 | 2 |
| BP | GO:0050892 | intestinal absorption | 2/87 | 35/18493 | 0.011770643 | 0.033165014 | 0.018315385 | AKR1C1/VDR | 2 |
| BP | GO:0060969 | negative regulation of gene silencing | 2/87 | 35/18493 | 0.011770643 | 0.033165014 | 0.018315385 | ESR1/PPARG | 2 |
| BP | GO:0090218 | positive regulation of lipid kinase activity | 2/87 | 35/18493 | 0.011770643 | 0.033165014 | 0.018315385 | F2/P2RY12 | 2 |
| BP | GO:0110111 | NA | 2/87 | 35/18493 | 0.011770643 | 0.033165014 | 0.018315385 | BCL2/TNF | 2 |
| BP | GO:1903539 | protein localization to postsynaptic membrane | 2/87 | 35/18493 | 0.011770643 | 0.033165014 | 0.018315385 | GRIN1/GRIN2A | 2 |
| BP | GO:0046632 | alpha-beta T cell differentiation | 3/87 | 101/18493 | 0.012033183 | 0.03387401 | 0.018706929 | ADA/BCL2/IL6 | 3 |
| BP | GO:0033559 | unsaturated fatty acid metabolic process | 3/87 | 102/18493 | 0.012356391 | 0.034720901 | 0.019174624 | PTGS2/PTGS1/PTGIS | 3 |
| BP | GO:1901222 | regulation of NIK/NF-kappaB signaling | 3/87 | 102/18493 | 0.012356391 | 0.034720901 | 0.019174624 | RELA/APP/TNF | 3 |
| BP | GO:0030212 | hyaluronan metabolic process | 2/87 | 36/18493 | 0.012425279 | 0.034725915 | 0.019177393 | AKT1/NFKB1 | 2 |
| BP | GO:0034405 | response to fluid shear stress | 2/87 | 36/18493 | 0.012425279 | 0.034725915 | 0.019177393 | PTGS2/AKT1 | 2 |
| BP | GO:0042092 | type 2 immune response | 2/87 | 36/18493 | 0.012425279 | 0.034725915 | 0.019177393 | IL10/IL6 | 2 |
| BP | GO:0060416 | response to growth hormone | 2/87 | 36/18493 | 0.012425279 | 0.034725915 | 0.019177393 | F7/AKT1 | 2 |
| BP | GO:0070884 | regulation of calcineurin-NFAT signaling cascade | 2/87 | 36/18493 | 0.012425279 | 0.034725915 | 0.019177393 | GSK3B/TNF | 2 |
| BP | GO:0106056 | regulation of calcineurin-mediated signaling | 2/87 | 36/18493 | 0.012425279 | 0.034725915 | 0.019177393 | GSK3B/TNF | 2 |
| BP | GO:0009749 | response to glucose | 4/87 | 191/18493 | 0.012616868 | 0.035229654 | 0.019455583 | LPL/HMGCR/PPARD/CASP3 | 4 |
| BP | GO:0051963 | regulation of synapse assembly | 3/87 | 104/18493 | 0.013017695 | 0.036175788 | 0.019978086 | BDNF/GRIN1/APP | 3 |
| BP | GO:0030278 | regulation of ossification | 4/87 | 193/18493 | 0.013065786 | 0.036175788 | 0.019978086 | ADRB2/BCL2/IL6/TNF | 4 |
| BP | GO:0002251 | organ or tissue specific immune response | 2/87 | 37/18493 | 0.013095519 | 0.036175788 | 0.019978086 | NOS2/IL6 | 2 |
| BP | GO:0010874 | regulation of cholesterol efflux | 2/87 | 37/18493 | 0.013095519 | 0.036175788 | 0.019978086 | PON1/CETP | 2 |
| BP | GO:0014912 | negative regulation of smooth muscle cell migration | 2/87 | 37/18493 | 0.013095519 | 0.036175788 | 0.019978086 | SERPINE1/PPARD | 2 |
| BP | GO:0046621 | negative regulation of organ growth | 2/87 | 37/18493 | 0.013095519 | 0.036175788 | 0.019978086 | SLC6A4/PPARA | 2 |
| BP | GO:0048009 | insulin-like growth factor receptor signaling pathway | 2/87 | 37/18493 | 0.013095519 | 0.036175788 | 0.019978086 | AR/AKT1 | 2 |
| BP | GO:0050856 | regulation of T cell receptor signaling pathway | 2/87 | 37/18493 | 0.013095519 | 0.036175788 | 0.019978086 | ADA/RELA | 2 |
| BP | GO:0055090 | acylglycerol homeostasis | 2/87 | 37/18493 | 0.013095519 | 0.036175788 | 0.019978086 | LPL/CETP | 2 |
| BP | GO:0070328 | triglyceride homeostasis | 2/87 | 37/18493 | 0.013095519 | 0.036175788 | 0.019978086 | LPL/CETP | 2 |
| BP | GO:0071276 | cellular response to cadmium ion | 2/87 | 37/18493 | 0.013095519 | 0.036175788 | 0.019978086 | SOD1/AKT1 | 2 |
| BP | GO:0086010 | membrane depolarization during action potential | 2/87 | 37/18493 | 0.013095519 | 0.036175788 | 0.019978086 | SCN5A/KCNH2 | 2 |
| BP | GO:0060541 | respiratory system development | 4/87 | 194/18493 | 0.013294026 | 0.036658926 | 0.020244899 | ADA/PGR/HSD11B1/TNF | 4 |
| BP | GO:0072376 | protein activation cascade | 4/87 | 194/18493 | 0.013294026 | 0.036658926 | 0.020244899 | F2/F7/F10/CRP | 4 |
| BP | GO:0002821 | positive regulation of adaptive immune response | 3/87 | 105/18493 | 0.013355812 | 0.03673144 | 0.020284945 | ADA/IL6/TNF | 3 |
| BP | GO:0022037 | metencephalon development | 3/87 | 105/18493 | 0.013355812 | 0.03673144 | 0.020284945 | SCN5A/BCL2/GRIN1 | 3 |
| BP | GO:0097006 | regulation of plasma lipoprotein particle levels | 3/87 | 105/18493 | 0.013355812 | 0.03673144 | 0.020284945 | LPL/MPO/CETP | 3 |
| BP | GO:0009062 | fatty acid catabolic process | 3/87 | 106/18493 | 0.01369892 | 0.03760844 | 0.020769268 | PPARA/PPARD/AKT1 | 3 |
| BP | GO:0050868 | negative regulation of T cell activation | 3/87 | 106/18493 | 0.01369892 | 0.03760844 | 0.020769268 | ERBB2/IL10/CASP3 | 3 |
| BP | GO:0000060 | protein import into nucleus, translocation | 2/87 | 38/18493 | 0.013781203 | 0.037634685 | 0.020783762 | AKT1/TNF | 2 |
| BP | GO:0010661 | positive regulation of muscle cell apoptotic process | 2/87 | 38/18493 | 0.013781203 | 0.037634685 | 0.020783762 | PPARG/HMGCR | 2 |
| BP | GO:0045124 | regulation of bone resorption | 2/87 | 38/18493 | 0.013781203 | 0.037634685 | 0.020783762 | IL6/ITGB3 | 2 |
| BP | GO:0060306 | regulation of membrane repolarization | 2/87 | 38/18493 | 0.013781203 | 0.037634685 | 0.020783762 | SCN5A/KCNH2 | 2 |
| BP | GO:0086005 | ventricular cardiac muscle cell action potential | 2/87 | 38/18493 | 0.013781203 | 0.037634685 | 0.020783762 | SCN5A/KCNH2 | 2 |
| BP | GO:0086091 | regulation of heart rate by cardiac conduction | 2/87 | 38/18493 | 0.013781203 | 0.037634685 | 0.020783762 | SCN5A/KCNH2 | 2 |
| BP | GO:0048640 | negative regulation of developmental growth | 3/87 | 107/18493 | 0.014047028 | 0.038326909 | 0.021166043 | ADRB2/SLC6A4/PPARA | 3 |
| BP | GO:0042113 | B cell activation | 5/87 | 303/18493 | 0.014170645 | 0.038630249 | 0.021333563 | ADA/BCL2/IL10/IL6/CASP3 | 5 |
| BP | GO:0033146 | regulation of intracellular estrogen receptor signaling pathway | 2/87 | 39/18493 | 0.014482177 | 0.039375795 | 0.021745291 | ESR1/AR | 2 |
| BP | GO:0038179 | neurotrophin signaling pathway | 2/87 | 39/18493 | 0.014482177 | 0.039375795 | 0.021745291 | BDNF/CASP3 | 2 |
| BP | GO:0051154 | negative regulation of striated muscle cell differentiation | 2/87 | 39/18493 | 0.014482177 | 0.039375795 | 0.021745291 | BDNF/PPARA | 2 |
| BP | GO:0002688 | regulation of leukocyte chemotaxis | 3/87 | 109/18493 | 0.014758284 | 0.0400914 | 0.022140484 | F7/SERPINE1/IL6 | 3 |
| BP | GO:0010828 | positive regulation of glucose transmembrane transport | 2/87 | 40/18493 | 0.015198283 | 0.040999712 | 0.022642099 | INS/AKT1 | 2 |
| BP | GO:0030810 | positive regulation of nucleotide biosynthetic process | 2/87 | 40/18493 | 0.015198283 | 0.040999712 | 0.022642099 | NOS2/INS | 2 |
| BP | GO:0030890 | positive regulation of B cell proliferation | 2/87 | 40/18493 | 0.015198283 | 0.040999712 | 0.022642099 | ADA/BCL2 | 2 |
| BP | GO:0043616 | keratinocyte proliferation | 2/87 | 40/18493 | 0.015198283 | 0.040999712 | 0.022642099 | PPARD/VDR | 2 |
| BP | GO:0044058 | regulation of digestive system process | 2/87 | 40/18493 | 0.015198283 | 0.040999712 | 0.022642099 | TRPV1/SLC22A5 | 2 |
| BP | GO:0048286 | lung alveolus development | 2/87 | 40/18493 | 0.015198283 | 0.040999712 | 0.022642099 | ADA/PGR | 2 |
| BP | GO:1900015 | regulation of cytokine production involved in inflammatory response | 2/87 | 40/18493 | 0.015198283 | 0.040999712 | 0.022642099 | NOS2/F2 | 2 |
| BP | GO:1900373 | positive regulation of purine nucleotide biosynthetic process | 2/87 | 40/18493 | 0.015198283 | 0.040999712 | 0.022642099 | NOS2/INS | 2 |
| BP | GO:0050870 | positive regulation of T cell activation | 4/87 | 202/18493 | 0.015211915 | 0.041000864 | 0.022642735 | ADA/DPP4/IL6/AKT1 | 4 |
| BP | GO:0046434 | organophosphate catabolic process | 4/87 | 203/18493 | 0.01546328 | 0.041641 | 0.02299625 | ADA/PPARA/PON1/INS | 4 |
| BP | GO:0002065 | columnar/cuboidal epithelial cell differentiation | 3/87 | 111/18493 | 0.015489647 | 0.041641 | 0.02299625 | GSK3B/SOD1/SERPINE1 | 3 |
| BP | GO:0002698 | negative regulation of immune effector process | 3/87 | 111/18493 | 0.015489647 | 0.041641 | 0.02299625 | INS/IL10/TNF | 3 |
| BP | GO:0046717 | acid secretion | 3/87 | 112/18493 | 0.015862889 | 0.042565134 | 0.023506604 | TRPV1/NOS2/CCK | 3 |
| BP | GO:0051817 | modification of morphology or physiology of other organism involved in symbiotic interaction | 3/87 | 112/18493 | 0.015862889 | 0.042565134 | 0.023506604 | F2/PLG/SP1 | 3 |
| BP | GO:0006110 | regulation of glycolytic process | 2/87 | 41/18493 | 0.015929368 | 0.042565134 | 0.023506604 | PPARA/INS | 2 |
| BP | GO:0032715 | negative regulation of interleukin-6 production | 2/87 | 41/18493 | 0.015929368 | 0.042565134 | 0.023506604 | IL10/TNF | 2 |
| BP | GO:0099622 | cardiac muscle cell membrane repolarization | 2/87 | 41/18493 | 0.015929368 | 0.042565134 | 0.023506604 | SCN5A/KCNH2 | 2 |
| BP | GO:1901028 | regulation of mitochondrial outer membrane permeabilization involved in apoptotic signaling pathway | 2/87 | 41/18493 | 0.015929368 | 0.042565134 | 0.023506604 | GSK3B/BCL2 | 2 |
| BP | GO:1904706 | negative regulation of vascular smooth muscle cell proliferation | 2/87 | 41/18493 | 0.015929368 | 0.042565134 | 0.023506604 | PPARG/IL10 | 2 |
| BP | GO:0044282 | small molecule catabolic process | 6/87 | 431/18493 | 0.016032503 | 0.042803887 | 0.023638455 | ADA/NOS2/PPARA/PPARD/PON1/AKT1 | 6 |
| BP | GO:0031098 | stress-activated protein kinase signaling cascade | 5/87 | 313/18493 | 0.016093473 | 0.042929755 | 0.023707966 | HMGCR/AKT1/APP/NFKB1/TNF | 5 |
| BP | GO:0007569 | cell aging | 3/87 | 113/18493 | 0.016241181 | 0.043249456 | 0.023884521 | SOD1/SERPINE1/BCL2 | 3 |
| BP | GO:0051153 | regulation of striated muscle cell differentiation | 3/87 | 113/18493 | 0.016241181 | 0.043249456 | 0.023884521 | BDNF/PPARA/BCL2 | 3 |
| BP | GO:1904062 | regulation of cation transmembrane transport | 5/87 | 314/18493 | 0.016294863 | 0.043355226 | 0.023942932 | SCN5A/ADRB2/F2/KCNH2/APP | 5 |
| BP | GO:0044242 | cellular lipid catabolic process | 4/87 | 207/18493 | 0.016494948 | 0.043820423 | 0.024199837 | LPL/PPARA/PPARD/AKT1 | 4 |
| BP | GO:0009615 | response to virus | 5/87 | 315/18493 | 0.016497931 | 0.043820423 | 0.024199837 | CHRM2/BCL2/RELA/IL6/TNF | 5 |
| BP | GO:0042552 | myelination | 3/87 | 114/18493 | 0.016624528 | 0.04399043 | 0.024293723 | SOD1/ERBB2/AKT1 | 3 |
| BP | GO:0002437 | inflammatory response to antigenic stimulus | 2/87 | 42/18493 | 0.016675277 | 0.04399043 | 0.024293723 | IL10/TNF | 2 |
| BP | GO:0006509 | membrane protein ectodomain proteolysis | 2/87 | 42/18493 | 0.016675277 | 0.04399043 | 0.024293723 | IL10/TNF | 2 |
| BP | GO:0030811 | regulation of nucleotide catabolic process | 2/87 | 42/18493 | 0.016675277 | 0.04399043 | 0.024293723 | PPARA/INS | 2 |
| BP | GO:0032459 | regulation of protein oligomerization | 2/87 | 42/18493 | 0.016675277 | 0.04399043 | 0.024293723 | INS/CCK | 2 |
| BP | GO:0042149 | cellular response to glucose starvation | 2/87 | 42/18493 | 0.016675277 | 0.04399043 | 0.024293723 | BCL2/SLC2A1 | 2 |
| BP | GO:0042551 | neuron maturation | 2/87 | 42/18493 | 0.016675277 | 0.04399043 | 0.024293723 | BCL2/APP | 2 |
| BP | GO:0071675 | regulation of mononuclear cell migration | 2/87 | 42/18493 | 0.016675277 | 0.04399043 | 0.024293723 | SERPINE1/TNF | 2 |
| BP | GO:0034754 | cellular hormone metabolic process | 3/87 | 115/18493 | 0.017012938 | 0.0448431 | 0.024764611 | ESR1/AKR1C1/HSD17B1 | 3 |
| BP | GO:0043583 | ear development | 4/87 | 210/18493 | 0.017296442 | 0.045513094 | 0.025134615 | SOD1/BCL2/GABRA5/GABRB3 | 4 |
| BP | GO:0050920 | regulation of chemotaxis | 4/87 | 210/18493 | 0.017296442 | 0.045513094 | 0.025134615 | F7/SERPINE1/IL6/P2RY12 | 4 |
| BP | GO:0002534 | cytokine production involved in inflammatory response | 2/87 | 43/18493 | 0.01743586 | 0.045763606 | 0.02527296 | NOS2/F2 | 2 |
| BP | GO:0014009 | glial cell proliferation | 2/87 | 43/18493 | 0.01743586 | 0.045763606 | 0.02527296 | IL6/TNF | 2 |
| BP | GO:0046688 | response to copper ion | 2/87 | 43/18493 | 0.01743586 | 0.045763606 | 0.02527296 | SOD1/APP | 2 |
| BP | GO:0006888 | ER to Golgi vesicle-mediated transport | 4/87 | 211/18493 | 0.017568932 | 0.046073929 | 0.025444336 | F2/F7/F10/INS | 4 |
| BP | GO:0045732 | positive regulation of protein catabolic process | 4/87 | 212/18493 | 0.017844097 | 0.046756051 | 0.025821038 | HSP90AA1/GSK3B/AKT1/TNF | 4 |
| BP | GO:0033173 | calcineurin-NFAT signaling cascade | 2/87 | 44/18493 | 0.018210964 | 0.047516844 | 0.026241186 | GSK3B/TNF | 2 |
| BP | GO:0046850 | regulation of bone remodeling | 2/87 | 44/18493 | 0.018210964 | 0.047516844 | 0.026241186 | IL6/ITGB3 | 2 |
| BP | GO:0050850 | positive regulation of calcium-mediated signaling | 2/87 | 44/18493 | 0.018210964 | 0.047516844 | 0.026241186 | ADA/TNF | 2 |
| BP | GO:0071364 | cellular response to epidermal growth factor stimulus | 2/87 | 44/18493 | 0.018210964 | 0.047516844 | 0.026241186 | ERBB2/AKT1 | 2 |
| BP | GO:1901031 | regulation of response to reactive oxygen species | 2/87 | 44/18493 | 0.018210964 | 0.047516844 | 0.026241186 | IL10/TNF | 2 |
| BP | GO:0001649 | osteoblast differentiation | 4/87 | 214/18493 | 0.018402482 | 0.047895814 | 0.026450473 | IL6/ACHE/AKT1/TNF | 4 |
| BP | GO:0017038 | protein import | 4/87 | 214/18493 | 0.018402482 | 0.047895814 | 0.026450473 | PTGS2/HSP90AA1/AKT1/TNF | 4 |
| BP | GO:0019935 | cyclic-nucleotide-mediated signaling | 4/87 | 214/18493 | 0.018402482 | 0.047895814 | 0.026450473 | ADRA1B/ADRB2/ADRA1D/PTGIR | 4 |
| BP | GO:0006814 | sodium ion transport | 4/87 | 215/18493 | 0.018685716 | 0.048592252 | 0.02683508 | SCN5A/ADRB2/SLC22A5/AKT1 | 4 |
| BP | GO:0002686 | negative regulation of leukocyte migration | 2/87 | 45/18493 | 0.01900044 | 0.049204643 | 0.027173274 | ADA/AKT1 | 2 |
| BP | GO:0006111 | regulation of gluconeogenesis | 2/87 | 45/18493 | 0.01900044 | 0.049204643 | 0.027173274 | PPARA/INS | 2 |
| BP | GO:0044764 | multi-organism cellular process | 2/87 | 45/18493 | 0.01900044 | 0.049204643 | 0.027173274 | F2/SLC22A5 | 2 |
| BP | GO:0048538 | thymus development | 2/87 | 45/18493 | 0.01900044 | 0.049204643 | 0.027173274 | SOD1/BCL2 | 2 |
| BP | GO:1900034 | regulation of cellular response to heat | 2/87 | 45/18493 | 0.01900044 | 0.049204643 | 0.027173274 | HSP90AA1/GSK3B | 2 |
| BP | GO:0045727 | positive regulation of translation | 3/87 | 120/18493 | 0.019031101 | 0.049242973 | 0.027194441 | ERBB2/IL6/TNF | 3 |
| BP | GO:0009108 | coenzyme biosynthetic process | 4/87 | 217/18493 | 0.019260302 | 0.04975311 | 0.027476165 | PTGS2/PPARA/INS/PTGIS | 4 |
| BP | GO:0030595 | leukocyte chemotaxis | 4/87 | 217/18493 | 0.019260302 | 0.04975311 | 0.027476165 | F7/SERPINE1/IL10/IL6 | 4 |
| CC | GO:1902711 | GABA-A receptor complex | 10/88 | 19/19659 | 1.70E-19 | 4.04E-17 | 2.54E-17 | GABRA1/GABRA3/GABRA4/GABRA5/GABRB3/GABRD/GABRE/GABRG2/GABRP/GABRQ | 10 |
| CC | GO:1902710 | GABA receptor complex | 10/88 | 20/19659 | 3.39E-19 | 4.04E-17 | 2.54E-17 | GABRA1/GABRA3/GABRA4/GABRA5/GABRB3/GABRD/GABRE/GABRG2/GABRP/GABRQ | 10 |
| CC | GO:0043235 | receptor complex | 22/88 | 396/19659 | 2.47E-18 | 1.96E-16 | 1.23E-16 | CHRNA2/ADRB2/GRIA2/ERBB2/IL6/VDR/GABRA1/GABRA3/GABRA4/GABRA5/GABRB3/GABRD/GABRE/GABRG2/GABRP/GABRQ/GRIN1/GRIN2A/GRIN2B/GRIN2D/APP/ITGB3 | 22 |
| CC | GO:0045211 | postsynaptic membrane | 20/88 | 322/19659 | 1.20E-17 | 7.16E-16 | 4.50E-16 | CHRM2/CHRNA2/TRPV1/SLC6A3/SLC6A4/GRIA2/GABRA1/GABRA3/GABRA4/GABRA5/GABRB3/GABRD/GABRE/GABRG2/GABRP/GABRQ/GRIN1/GRIN2A/GRIN2B/GRIN2D | 20 |
| CC | GO:0097060 | synaptic membrane | 21/88 | 431/19659 | 2.30E-16 | 1.09E-14 | 6.86E-15 | CHRM2/CHRNA2/SLC6A2/TRPV1/SLC6A3/SLC6A4/GRIA2/GABRA1/GABRA3/GABRA4/GABRA5/GABRB3/GABRD/GABRE/GABRG2/GABRP/GABRQ/GRIN1/GRIN2A/GRIN2B/GRIN2D | 21 |
| CC | GO:0034702 | ion channel complex | 18/88 | 301/19659 | 1.19E-15 | 4.70E-14 | 2.95E-14 | CHRNA2/SCN5A/KCNH2/GRIA2/GABRA1/GABRA3/GABRA4/GABRA5/GABRB3/GABRD/GABRE/GABRG2/GABRP/GABRQ/GRIN1/GRIN2A/GRIN2B/GRIN2D | 18 |
| CC | GO:1902495 | transmembrane transporter complex | 18/88 | 325/19659 | 4.52E-15 | 1.54E-13 | 9.65E-14 | CHRNA2/SCN5A/KCNH2/GRIA2/GABRA1/GABRA3/GABRA4/GABRA5/GABRB3/GABRD/GABRE/GABRG2/GABRP/GABRQ/GRIN1/GRIN2A/GRIN2B/GRIN2D | 18 |
| CC | GO:1990351 | transporter complex | 18/88 | 333/19659 | 6.89E-15 | 2.05E-13 | 1.29E-13 | CHRNA2/SCN5A/KCNH2/GRIA2/GABRA1/GABRA3/GABRA4/GABRA5/GABRB3/GABRD/GABRE/GABRG2/GABRP/GABRQ/GRIN1/GRIN2A/GRIN2B/GRIN2D | 18 |
| CC | GO:0034707 | chloride channel complex | 10/88 | 50/19659 | 1.69E-14 | 4.48E-13 | 2.81E-13 | GABRA1/GABRA3/GABRA4/GABRA5/GABRB3/GABRD/GABRE/GABRG2/GABRP/GABRQ | 10 |
| CC | GO:0099055 | integral component of postsynaptic membrane | 10/88 | 117/19659 | 1.15E-10 | 2.74E-09 | 1.72E-09 | CHRM2/SLC6A3/SLC6A4/GABRA1/GABRA3/GABRA4/GABRA5/GABRD/GRIN1/GRIN2A | 10 |
| CC | GO:0098936 | intrinsic component of postsynaptic membrane | 10/88 | 122/19659 | 1.75E-10 | 3.79E-09 | 2.38E-09 | CHRM2/SLC6A3/SLC6A4/GABRA1/GABRA3/GABRA4/GABRA5/GABRD/GRIN1/GRIN2A | 10 |
| CC | GO:0032590 | dendrite membrane | 7/88 | 40/19659 | 4.67E-10 | 9.26E-09 | 5.82E-09 | TRPV1/GABRA1/GABRA3/GABRA4/GABRA5/GABRE/GABRG2 | 7 |
| CC | GO:0045121 | membrane raft | 13/88 | 304/19659 | 9.52E-10 | 1.68E-08 | 1.06E-08 | PTGS2/SLC6A2/SCN5A/ADRA1B/SLC6A3/SLC6A4/DPP4/APP/SLC2A1/CASP3/TNF/P2RY12/PTGIS | 13 |
| CC | GO:0098857 | membrane microdomain | 13/88 | 305/19659 | 9.91E-10 | 1.68E-08 | 1.06E-08 | PTGS2/SLC6A2/SCN5A/ADRA1B/SLC6A3/SLC6A4/DPP4/APP/SLC2A1/CASP3/TNF/P2RY12/PTGIS | 13 |
| CC | GO:0098589 | membrane region | 13/88 | 316/19659 | 1.52E-09 | 2.42E-08 | 1.52E-08 | PTGS2/SLC6A2/SCN5A/ADRA1B/SLC6A3/SLC6A4/DPP4/APP/SLC2A1/CASP3/TNF/P2RY12/PTGIS | 13 |
| CC | GO:0099699 | integral component of synaptic membrane | 10/88 | 154/19659 | 1.73E-09 | 2.58E-08 | 1.62E-08 | CHRM2/SLC6A3/SLC6A4/GABRA1/GABRA3/GABRA4/GABRA5/GABRD/GRIN1/GRIN2A | 10 |
| CC | GO:0099240 | intrinsic component of synaptic membrane | 10/88 | 166/19659 | 3.60E-09 | 5.03E-08 | 3.16E-08 | CHRM2/SLC6A3/SLC6A4/GABRA1/GABRA3/GABRA4/GABRA5/GABRD/GRIN1/GRIN2A | 10 |
| CC | GO:0032589 | neuron projection membrane | 7/88 | 57/19659 | 6.23E-09 | 8.24E-08 | 5.17E-08 | TRPV1/GABRA1/GABRA3/GABRA4/GABRA5/GABRE/GABRG2 | 7 |
| CC | GO:0099634 | postsynaptic specialization membrane | 8/88 | 102/19659 | 1.81E-08 | 2.27E-07 | 1.43E-07 | GABRA1/GABRA3/GABRA4/GABRA5/GRIN1/GRIN2A/GRIN2B/GRIN2D | 8 |
| CC | GO:0098982 | GABA-ergic synapse | 7/88 | 75/19659 | 4.38E-08 | 5.21E-07 | 3.28E-07 | GABRA1/GABRA3/GABRA4/GABRA5/GABRB3/GABRD/GABRG2 | 7 |
| CC | GO:0005901 | caveola | 7/88 | 77/19659 | 5.27E-08 | 5.97E-07 | 3.75E-07 | PTGS2/SCN5A/ADRA1B/SLC6A3/SLC2A1/P2RY12/PTGIS | 7 |
| CC | GO:0031256 | leading edge membrane | 9/88 | 167/19659 | 5.97E-08 | 6.46E-07 | 4.06E-07 | TRPV1/DPP4/GABRA1/GABRA3/GABRA4/GABRA5/GABRE/GABRG2/ITGB3 | 9 |
| CC | GO:0017146 | NMDA selective glutamate receptor complex | 4/88 | 11/19659 | 1.21E-07 | 1.25E-06 | 7.85E-07 | GRIN1/GRIN2A/GRIN2B/GRIN2D | 4 |
| CC | GO:0044853 | plasma membrane raft | 7/88 | 102/19659 | 3.70E-07 | 3.67E-06 | 2.30E-06 | PTGS2/SCN5A/ADRA1B/SLC6A3/SLC2A1/P2RY12/PTGIS | 7 |
| CC | GO:0099060 | integral component of postsynaptic specialization membrane | 6/88 | 74/19659 | 9.82E-07 | 9.35E-06 | 5.87E-06 | GABRA1/GABRA3/GABRA4/GABRA5/GRIN1/GRIN2A | 6 |
| CC | GO:0098948 | intrinsic component of postsynaptic specialization membrane | 6/88 | 77/19659 | 1.24E-06 | 1.14E-05 | 7.15E-06 | GABRA1/GABRA3/GABRA4/GABRA5/GRIN1/GRIN2A | 6 |
| CC | GO:0098802 | plasma membrane receptor complex | 8/88 | 184/19659 | 1.72E-06 | 1.51E-05 | 9.50E-06 | CHRNA2/GRIA2/IL6/GRIN1/GRIN2A/GRIN2B/GRIN2D/ITGB3 | 8 |
| CC | GO:0031253 | cell projection membrane | 10/88 | 334/19659 | 2.45E-06 | 2.08E-05 | 1.31E-05 | TRPV1/DPP4/SLC22A5/GABRA1/GABRA3/GABRA4/GABRA5/GABRE/GABRG2/ITGB3 | 10 |
| CC | GO:0008328 | ionotropic glutamate receptor complex | 5/88 | 52/19659 | 3.53E-06 | 2.90E-05 | 1.82E-05 | GRIA2/GRIN1/GRIN2A/GRIN2B/GRIN2D | 5 |
| CC | GO:0098878 | neurotransmitter receptor complex | 5/88 | 54/19659 | 4.26E-06 | 3.38E-05 | 2.12E-05 | GRIA2/GRIN1/GRIN2A/GRIN2B/GRIN2D | 5 |
| CC | GO:0031252 | cell leading edge | 10/88 | 390/19659 | 9.59E-06 | 7.36E-05 | 4.62E-05 | TRPV1/DPP4/GABRA1/GABRA3/GABRA4/GABRA5/GABRE/GABRG2/APP/ITGB3 | 10 |
| CC | GO:0060205 | cytoplasmic vesicle lumen | 9/88 | 338/19659 | 2.05E-05 | 0.00015098 | 9.48E-05 | HSP90AA1/ADA/SERPINE1/MPO/INS/PLG/ALOX5/APP/NFKB1 | 9 |
| CC | GO:0031983 | vesicle lumen | 9/88 | 339/19659 | 2.09E-05 | 0.00015098 | 9.48E-05 | HSP90AA1/ADA/SERPINE1/MPO/INS/PLG/ALOX5/APP/NFKB1 | 9 |
| CC | GO:0034703 | cation channel complex | 7/88 | 219/19659 | 5.72E-05 | 0.000392597 | 0.000246567 | SCN5A/KCNH2/GRIA2/GRIN1/GRIN2A/GRIN2B/GRIN2D | 7 |
| CC | GO:0098793 | presynapse | 10/88 | 482/19659 | 5.84E-05 | 0.000392597 | 0.000246567 | CHRM2/SLC6A2/SLC6A3/SLC6A4/BDNF/CCK/GABRA5/GRIN1/GRIN2A/APP | 10 |
| CC | GO:0043025 | neuronal cell body | 10/88 | 483/19659 | 5.94E-05 | 0.000392597 | 0.000246567 | CHRM2/SLC6A2/TRPV1/HSP90AA1/ADA/SLC6A3/SOD1/CCK/GABRA5/CASP3 | 10 |
| CC | GO:0005788 | endoplasmic reticulum lumen | 8/88 | 301/19659 | 6.12E-05 | 0.000393776 | 0.000247307 | PTGS2/F2/F7/F10/INS/IL6/BCHE/APP | 8 |
| CC | GO:0042734 | presynaptic membrane | 6/88 | 161/19659 | 8.56E-05 | 0.000536013 | 0.000336638 | CHRM2/SLC6A2/SLC6A3/SLC6A4/GABRA5/GRIN2A | 6 |
| CC | GO:0034774 | secretory granule lumen | 8/88 | 321/19659 | 9.58E-05 | 0.000584808 | 0.000367284 | HSP90AA1/SERPINE1/MPO/INS/PLG/ALOX5/APP/NFKB1 | 8 |
| CC | GO:0005796 | Golgi lumen | 5/88 | 103/19659 | 9.94E-05 | 0.000591203 | 0.000371299 | F2/F7/F10/INS/APP | 5 |
| CC | GO:0099572 | postsynaptic specialization | 8/88 | 340/19659 | 0.000142423 | 0.00082675 | 0.000519233 | GABRA1/GABRA3/GABRA4/GABRA5/GRIN1/GRIN2A/GRIN2B/GRIN2D | 8 |
| CC | GO:0009897 | external side of plasma membrane | 8/88 | 359/19659 | 0.000206335 | 0.00116923 | 0.000734324 | TRPV1/ADA/F2/GRIA2/F10/PLG/TNF/P2RY12 | 8 |
| CC | GO:0098839 | postsynaptic density membrane | 4/88 | 75/19659 | 0.000357538 | 0.001933957 | 0.001214604 | GRIN1/GRIN2A/GRIN2B/GRIN2D | 4 |
| CC | GO:0099056 | integral component of presynaptic membrane | 4/88 | 75/19659 | 0.000357538 | 0.001933957 | 0.001214604 | CHRM2/SLC6A3/SLC6A4/GABRA5 | 4 |
| CC | GO:0072562 | blood microparticle | 5/88 | 147/19659 | 0.00051964 | 0.002748318 | 0.001726056 | F2/PON1/PLG/BCHE/SLC2A1 | 5 |
| CC | GO:0098889 | intrinsic component of presynaptic membrane | 4/88 | 84/19659 | 0.000550491 | 0.002848192 | 0.001788781 | CHRM2/SLC6A3/SLC6A4/GABRA5 | 4 |
| CC | GO:0031970 | organelle envelope lumen | 4/88 | 85/19659 | 0.000575717 | 0.002915335 | 0.001830949 | SOD1/BCHE/ALOX5/APP | 4 |
| CC | GO:0034358 | plasma lipoprotein particle | 3/88 | 38/19659 | 0.000652815 | 0.003170814 | 0.0019914 | LPL/PON1/CETP | 3 |
| CC | GO:1990777 | lipoprotein particle | 3/88 | 38/19659 | 0.000652815 | 0.003170814 | 0.0019914 | LPL/PON1/CETP | 3 |
| CC | GO:0031091 | platelet alpha granule | 4/88 | 91/19659 | 0.000744613 | 0.003544358 | 0.002226001 | SERPINE1/PLG/APP/ITGB3 | 4 |
| CC | GO:0032994 | protein-lipid complex | 3/88 | 40/19659 | 0.000759638 | 0.003544977 | 0.00222639 | LPL/PON1/CETP | 3 |
| CC | GO:0098978 | glutamatergic synapse | 7/88 | 350/19659 | 0.000986027 | 0.004512968 | 0.002834328 | CHRM2/GSK3B/RELA/GABRD/GRIN1/GRIN2A/ITGB3 | 7 |
| CC | GO:0043083 | synaptic cleft | 2/88 | 14/19659 | 0.001740794 | 0.007817152 | 0.00490949 | GRIN1/ACHE | 2 |
| CC | GO:0031093 | platelet alpha granule lumen | 3/88 | 67/19659 | 0.003376634 | 0.014882201 | 0.009346628 | SERPINE1/PLG/APP | 3 |
| CC | GO:0031258 | lamellipodium membrane | 2/88 | 22/19659 | 0.004317393 | 0.018682536 | 0.011733393 | DPP4/ITGB3 | 2 |
| CC | GO:0090575 | RNA polymerase II transcription factor complex | 4/88 | 161/19659 | 0.00592978 | 0.024035925 | 0.015095539 | PPARG/PPARA/PPARD/VDR | 4 |
| CC | GO:0032809 | neuronal cell body membrane | 2/88 | 26/19659 | 0.006004196 | 0.024035925 | 0.015095539 | SLC6A2/GABRA5 | 2 |
| CC | GO:0034364 | high-density lipoprotein particle | 2/88 | 26/19659 | 0.006004196 | 0.024035925 | 0.015095539 | PON1/CETP | 2 |
| CC | GO:0044298 | cell body membrane | 2/88 | 26/19659 | 0.006004196 | 0.024035925 | 0.015095539 | SLC6A2/GABRA5 | 2 |
| CC | GO:0043209 | myelin sheath | 4/88 | 162/19659 | 0.006059477 | 0.024035925 | 0.015095539 | HSP90AA1/SOD1/ERBB2/BCL2 | 4 |
| CC | GO:0043197 | dendritic spine | 4/88 | 164/19659 | 0.006324516 | 0.024533189 | 0.015407841 | TRPV1/GRIA2/GRIN1/APP | 4 |
| CC | GO:0033267 | axon part | 6/88 | 373/19659 | 0.006585895 | 0.024533189 | 0.015407841 | CHRM2/HSP90AA1/SOD1/CCK/GRIN1/APP | 6 |
| CC | GO:0045177 | apical part of cell | 6/88 | 373/19659 | 0.006585895 | 0.024533189 | 0.015407841 | ADRB2/DPP4/ERBB2/SLC22A5/APP/SLC2A1 | 6 |
| CC | GO:0044309 | neuron spine | 4/88 | 166/19659 | 0.00659716 | 0.024533189 | 0.015407841 | TRPV1/GRIA2/GRIN1/APP | 4 |
| CC | GO:0150034 | distal axon | 5/88 | 280/19659 | 0.008480034 | 0.031049972 | 0.019500646 | CHRM2/HSP90AA1/CCK/GRIN1/APP | 5 |
| CC | GO:0009925 | basal plasma membrane | 2/88 | 34/19659 | 0.010126946 | 0.035444311 | 0.022260469 | ERBB2/P2RY12 | 2 |
| CC | GO:0031904 | endosome lumen | 2/88 | 34/19659 | 0.010126946 | 0.035444311 | 0.022260469 | INS/APP | 2 |
| CC | GO:0032839 | dendrite cytoplasm | 2/88 | 34/19659 | 0.010126946 | 0.035444311 | 0.022260469 | ADA/SOD1 | 2 |
| CC | GO:0042470 | melanosome | 3/88 | 106/19659 | 0.012003346 | 0.04044196 | 0.025399197 | HSP90AA1/ITGB3/SLC2A1 | 3 |
| CC | GO:0048770 | pigment granule | 3/88 | 106/19659 | 0.012003346 | 0.04044196 | 0.025399197 | HSP90AA1/ITGB3/SLC2A1 | 3 |
| CC | GO:0044798 | nuclear transcription factor complex | 4/88 | 198/19659 | 0.012064618 | 0.04044196 | 0.025399197 | PPARG/PPARA/PPARD/VDR | 4 |
| CC | GO:0016324 | apical plasma membrane | 5/88 | 308/19659 | 0.01243942 | 0.041119193 | 0.025824527 | ADRB2/DPP4/ERBB2/SLC22A5/SLC2A1 | 5 |
| CC | GO:0032279 | asymmetric synapse | 5/88 | 320/19659 | 0.014466675 | 0.047165323 | 0.029621742 | CHRM2/GRIN1/GRIN2A/GRIN2B/GRIN2D | 5 |
| MF | GO:0005230 | extracellular ligand-gated ion channel activity | 17/88 | 75/17632 | 3.40E-24 | 1.28E-21 | 7.54E-22 | CHRNA2/TRPV1/GRIA2/GABRA1/GABRA3/GABRA4/GABRA5/GABRB3/GABRD/GABRE/GABRG2/GABRP/GABRQ/GRIN1/GRIN2A/GRIN2B/GRIN2D | 17 |
| MF | GO:0015276 | ligand-gated ion channel activity | 18/88 | 144/17632 | 1.31E-20 | 1.65E-18 | 9.67E-19 | CHRNA2/TRPV1/KCNH2/GRIA2/GABRA1/GABRA3/GABRA4/GABRA5/GABRB3/GABRD/GABRE/GABRG2/GABRP/GABRQ/GRIN1/GRIN2A/GRIN2B/GRIN2D | 18 |
| MF | GO:0022834 | ligand-gated channel activity | 18/88 | 144/17632 | 1.31E-20 | 1.65E-18 | 9.67E-19 | CHRNA2/TRPV1/KCNH2/GRIA2/GABRA1/GABRA3/GABRA4/GABRA5/GABRB3/GABRD/GABRE/GABRG2/GABRP/GABRQ/GRIN1/GRIN2A/GRIN2B/GRIN2D | 18 |
| MF | GO:0022824 | transmitter-gated ion channel activity | 14/88 | 61/17632 | 3.76E-20 | 2.84E-18 | 1.67E-18 | CHRNA2/GRIA2/GABRA1/GABRA3/GABRA4/GABRA5/GABRB3/GABRD/GABRE/GABRG2/GRIN1/GRIN2A/GRIN2B/GRIN2D | 14 |
| MF | GO:0022835 | transmitter-gated channel activity | 14/88 | 61/17632 | 3.76E-20 | 2.84E-18 | 1.67E-18 | CHRNA2/GRIA2/GABRA1/GABRA3/GABRA4/GABRA5/GABRB3/GABRD/GABRE/GABRG2/GRIN1/GRIN2A/GRIN2B/GRIN2D | 14 |
| MF | GO:0004890 | GABA-A receptor activity | 10/88 | 19/17632 | 5.04E-19 | 3.17E-17 | 1.86E-17 | GABRA1/GABRA3/GABRA4/GABRA5/GABRB3/GABRD/GABRE/GABRG2/GABRP/GABRQ | 10 |
| MF | GO:0016917 | GABA receptor activity | 10/88 | 22/17632 | 3.48E-18 | 1.88E-16 | 1.11E-16 | GABRA1/GABRA3/GABRA4/GABRA5/GABRB3/GABRD/GABRE/GABRG2/GABRP/GABRQ | 10 |
| MF | GO:0030594 | neurotransmitter receptor activity | 15/88 | 117/17632 | 1.79E-17 | 8.45E-16 | 4.97E-16 | CHRM2/CHRNA2/GRIA2/GABRA1/GABRA3/GABRA4/GABRA5/GABRB3/GABRD/GABRE/GABRG2/GRIN1/GRIN2A/GRIN2B/GRIN2D | 15 |
| MF | GO:0015108 | chloride transmembrane transporter activity | 13/88 | 100/17632 | 2.38E-15 | 9.98E-14 | 5.86E-14 | SLC6A2/SLC6A3/SLC6A4/GABRA1/GABRA3/GABRA4/GABRA5/GABRB3/GABRD/GABRE/GABRG2/GABRP/GABRQ | 13 |
| MF | GO:0022839 | ion gated channel activity | 19/88 | 342/17632 | 4.71E-15 | 1.78E-13 | 1.05E-13 | CHRNA2/TRPV1/SCN5A/KCNH2/GRIA2/GABRA1/GABRA3/GABRA4/GABRA5/GABRB3/GABRD/GABRE/GABRG2/GABRP/GABRQ/GRIN1/GRIN2A/GRIN2B/GRIN2D | 19 |
| MF | GO:1904315 | transmitter-gated ion channel activity involved in regulation of postsynaptic membrane potential | 10/88 | 42/17632 | 7.31E-15 | 2.38E-13 | 1.40E-13 | CHRNA2/GABRA1/GABRA3/GABRA4/GABRA5/GABRB3/GABRD/GABRE/GABRG2/GRIN1 | 10 |
| MF | GO:0022836 | gated channel activity | 19/88 | 351/17632 | 7.55E-15 | 2.38E-13 | 1.40E-13 | CHRNA2/TRPV1/SCN5A/KCNH2/GRIA2/GABRA1/GABRA3/GABRA4/GABRA5/GABRB3/GABRD/GABRE/GABRG2/GABRP/GABRQ/GRIN1/GRIN2A/GRIN2B/GRIN2D | 19 |
| MF | GO:0099529 | neurotransmitter receptor activity involved in regulation of postsynaptic membrane potential | 10/88 | 45/17632 | 1.57E-14 | 4.55E-13 | 2.68E-13 | CHRNA2/GABRA1/GABRA3/GABRA4/GABRA5/GABRB3/GABRD/GABRE/GABRG2/GRIN1 | 10 |
| MF | GO:0098960 | postsynaptic neurotransmitter receptor activity | 10/88 | 47/17632 | 2.52E-14 | 6.81E-13 | 4.00E-13 | CHRNA2/GABRA1/GABRA3/GABRA4/GABRA5/GABRB3/GABRD/GABRE/GABRG2/GRIN1 | 10 |
| MF | GO:0022851 | GABA-gated chloride ion channel activity | 7/88 | 12/17632 | 4.69E-14 | 1.18E-12 | 6.95E-13 | GABRA1/GABRA3/GABRA4/GABRA5/GABRB3/GABRE/GABRG2 | 7 |
| MF | GO:0015267 | channel activity | 20/88 | 467/17632 | 1.12E-13 | 2.60E-12 | 1.53E-12 | CHRNA2/TRPV1/SCN5A/KCNH2/GRIA2/BCL2/GABRA1/GABRA3/GABRA4/GABRA5/GABRB3/GABRD/GABRE/GABRG2/GABRP/GABRQ/GRIN1/GRIN2A/GRIN2B/GRIN2D | 20 |
| MF | GO:0022803 | passive transmembrane transporter activity | 20/88 | 468/17632 | 1.17E-13 | 2.60E-12 | 1.53E-12 | CHRNA2/TRPV1/SCN5A/KCNH2/GRIA2/BCL2/GABRA1/GABRA3/GABRA4/GABRA5/GABRB3/GABRD/GABRE/GABRG2/GABRP/GABRQ/GRIN1/GRIN2A/GRIN2B/GRIN2D | 20 |
| MF | GO:0005216 | ion channel activity | 19/88 | 427/17632 | 2.57E-13 | 5.39E-12 | 3.17E-12 | CHRNA2/TRPV1/SCN5A/KCNH2/GRIA2/GABRA1/GABRA3/GABRA4/GABRA5/GABRB3/GABRD/GABRE/GABRG2/GABRP/GABRQ/GRIN1/GRIN2A/GRIN2B/GRIN2D | 19 |
| MF | GO:0022838 | substrate-specific channel activity | 19/88 | 442/17632 | 4.74E-13 | 9.43E-12 | 5.54E-12 | CHRNA2/TRPV1/SCN5A/KCNH2/GRIA2/GABRA1/GABRA3/GABRA4/GABRA5/GABRB3/GABRD/GABRE/GABRG2/GABRP/GABRQ/GRIN1/GRIN2A/GRIN2B/GRIN2D | 19 |
| MF | GO:0015103 | inorganic anion transmembrane transporter activity | 13/88 | 157/17632 | 9.04E-13 | 1.71E-11 | 1.00E-11 | SLC6A2/SLC6A3/SLC6A4/GABRA1/GABRA3/GABRA4/GABRA5/GABRB3/GABRD/GABRE/GABRG2/GABRP/GABRQ | 13 |
| MF | GO:0004879 | nuclear receptor activity | 9/88 | 50/17632 | 2.68E-12 | 4.60E-11 | 2.70E-11 | ESR1/AR/PPARG/ESR2/NR3C1/PGR/PPARA/PPARD/VDR | 9 |
| MF | GO:0098531 | transcription factor activity, direct ligand regulated sequence-specific DNA binding | 9/88 | 50/17632 | 2.68E-12 | 4.60E-11 | 2.70E-11 | ESR1/AR/PPARG/ESR2/NR3C1/PGR/PPARA/PPARD/VDR | 9 |
| MF | GO:0099095 | ligand-gated anion channel activity | 7/88 | 19/17632 | 2.90E-12 | 4.77E-11 | 2.80E-11 | GABRA1/GABRA3/GABRA4/GABRA5/GABRB3/GABRE/GABRG2 | 7 |
| MF | GO:0005254 | chloride channel activity | 10/88 | 75/17632 | 3.61E-12 | 5.68E-11 | 3.34E-11 | GABRA1/GABRA3/GABRA4/GABRA5/GABRB3/GABRD/GABRE/GABRG2/GABRP/GABRQ | 10 |
| MF | GO:0003707 | steroid hormone receptor activity | 9/88 | 54/17632 | 5.59E-12 | 8.45E-11 | 4.97E-11 | ESR1/AR/PPARG/ESR2/NR3C1/PGR/PPARA/PPARD/VDR | 9 |
| MF | GO:0008503 | benzodiazepine receptor activity | 6/88 | 11/17632 | 5.89E-12 | 8.56E-11 | 5.03E-11 | GABRA1/GABRA3/GABRA4/GABRA5/GABRE/GABRG2 | 6 |
| MF | GO:0005253 | anion channel activity | 10/88 | 89/17632 | 2.09E-11 | 2.82E-10 | 1.66E-10 | GABRA1/GABRA3/GABRA4/GABRA5/GABRB3/GABRD/GABRE/GABRG2/GABRP/GABRQ | 10 |
| MF | GO:0005496 | steroid binding | 10/88 | 89/17632 | 2.09E-11 | 2.82E-10 | 1.66E-10 | ESR1/AR/ESR2/NR3C1/PGR/CETP/SHBG/VDR/HSD17B1/HSD11B1 | 10 |
| MF | GO:0005237 | inhibitory extracellular ligand-gated ion channel activity | 6/88 | 16/17632 | 1.00E-10 | 1.30E-09 | 7.66E-10 | GABRA1/GABRA3/GABRA4/GABRA5/GABRE/GABRG2 | 6 |
| MF | GO:0008509 | anion transmembrane transporter activity | 14/88 | 330/17632 | 9.08E-10 | 1.14E-08 | 6.72E-09 | SLC6A2/SLC6A3/SLC6A4/GABRA1/GABRA3/GABRA4/GABRA5/GABRB3/GABRD/GABRE/GABRG2/GABRP/GABRQ/SLC2A1 | 14 |
| MF | GO:0042165 | neurotransmitter binding | 7/88 | 55/17632 | 1.01E-08 | 1.23E-07 | 7.25E-08 | CHRNA2/SLC6A4/CRP/GRIN1/GRIN2B/ACHE/BCHE | 7 |
| MF | GO:0002020 | protease binding | 9/88 | 125/17632 | 1.21E-08 | 1.43E-07 | 8.37E-08 | SLC6A3/DPP4/GSK3B/SERPINE1/INS/BCL2/ITGB3/CASP3/TNF | 9 |
| MF | GO:0004970 | ionotropic glutamate receptor activity | 5/88 | 19/17632 | 3.04E-08 | 3.48E-07 | 2.04E-07 | GRIA2/GRIN1/GRIN2A/GRIN2B/GRIN2D | 5 |
| MF | GO:0099094 | ligand-gated cation channel activity | 8/88 | 106/17632 | 5.64E-08 | 6.27E-07 | 3.68E-07 | CHRNA2/TRPV1/KCNH2/GRIA2/GRIN1/GRIN2A/GRIN2B/GRIN2D | 8 |
| MF | GO:0070405 | ammonium ion binding | 7/88 | 74/17632 | 8.31E-08 | 8.97E-07 | 5.27E-07 | CHRNA2/SLC6A3/SLC6A4/CRP/CETP/ACHE/BCHE | 7 |
| MF | GO:0008066 | glutamate receptor activity | 5/88 | 27/17632 | 2.04E-07 | 2.09E-06 | 1.23E-06 | GRIA2/GRIN1/GRIN2A/GRIN2B/GRIN2D | 5 |
| MF | GO:0099604 | ligand-gated calcium channel activity | 5/88 | 27/17632 | 2.04E-07 | 2.09E-06 | 1.23E-06 | TRPV1/GRIN1/GRIN2A/GRIN2B/GRIN2D | 5 |
| MF | GO:0042562 | hormone binding | 7/88 | 104/17632 | 8.69E-07 | 8.65E-06 | 5.08E-06 | CHRNA2/AR/NR3C1/SHBG/VDR/HSD17B1/ACHE | 7 |
| MF | GO:0019902 | phosphatase binding | 8/88 | 178/17632 | 2.98E-06 | 2.89E-05 | 1.70E-05 | SLC6A3/PPARG/SOD1/ERBB2/HMGCR/PPARA/BCL2/AKT1 | 8 |
| MF | GO:0019903 | protein phosphatase binding | 7/88 | 133/17632 | 4.53E-06 | 4.28E-05 | 2.52E-05 | SLC6A3/PPARG/SOD1/ERBB2/HMGCR/BCL2/AKT1 | 7 |
| MF | GO:0031406 | carboxylic acid binding | 8/88 | 192/17632 | 5.22E-06 | 4.81E-05 | 2.83E-05 | NOS2/PPARG/PPARA/PPARD/AKR1C1/VDR/GRIN1/GRIN2B | 8 |
| MF | GO:0001085 | RNA polymerase II transcription factor binding | 7/88 | 140/17632 | 6.36E-06 | 5.72E-05 | 3.36E-05 | ESR1/AR/PPARG/GSK3B/PPARA/PPARD/SP1 | 7 |
| MF | GO:0043177 | organic acid binding | 8/88 | 204/17632 | 8.15E-06 | 7.16E-05 | 4.21E-05 | NOS2/PPARG/PPARA/PPARD/AKR1C1/VDR/GRIN1/GRIN2B | 8 |
| MF | GO:0017171 | serine hydrolase activity | 9/88 | 277/17632 | 9.89E-06 | 8.50E-05 | 4.99E-05 | F2/F7/DPP4/F10/PRSS3/PLAU/PLG/ACHE/RBP3 | 9 |
| MF | GO:0051721 | protein phosphatase 2A binding | 4/88 | 29/17632 | 1.25E-05 | 0.000105053 | 6.17E-05 | SLC6A3/HMGCR/BCL2/AKT1 | 4 |
| MF | GO:0015378 | sodium:chloride symporter activity | 3/88 | 10/17632 | 1.41E-05 | 0.000115499 | 6.79E-05 | SLC6A2/SLC6A3/SLC6A4 | 3 |
| MF | GO:0033293 | monocarboxylic acid binding | 5/88 | 64/17632 | 1.67E-05 | 0.000134275 | 7.89E-05 | PPARG/PPARA/PPARD/AKR1C1/VDR | 5 |
| MF | GO:0008519 | ammonium transmembrane transporter activity | 4/88 | 32/17632 | 1.87E-05 | 0.00014742 | 8.66E-05 | SLC6A2/SLC6A3/SLC6A4/SLC22A5 | 4 |
| MF | GO:0030235 | nitric-oxide synthase regulator activity | 3/88 | 11/17632 | 1.93E-05 | 0.000148549 | 8.73E-05 | HSP90AA1/ESR1/AKT1 | 3 |
| MF | GO:0005261 | cation channel activity | 9/88 | 320/17632 | 3.10E-05 | 0.000234431 | 0.000137747 | CHRNA2/TRPV1/SCN5A/KCNH2/GRIA2/GRIN1/GRIN2A/GRIN2B/GRIN2D | 9 |
| MF | GO:0015373 | anion:sodium symporter activity | 3/88 | 13/17632 | 3.31E-05 | 0.000245605 | 0.000144313 | SLC6A2/SLC6A3/SLC6A4 | 3 |
| MF | GO:0070491 | repressing transcription factor binding | 5/88 | 74/17632 | 3.39E-05 | 0.000246569 | 0.000144879 | PPARG/PPARA/PPARD/SP1/RELA | 5 |
| MF | GO:0001103 | RNA polymerase II repressing transcription factor binding | 4/88 | 38/17632 | 3.76E-05 | 0.000267874 | 0.000157397 | PPARG/PPARA/PPARD/SP1 | 4 |
| MF | GO:0015377 | cation:chloride symporter activity | 3/88 | 16/17632 | 6.42E-05 | 0.00044929 | 0.000263994 | SLC6A2/SLC6A3/SLC6A4 | 3 |
| MF | GO:0008236 | serine-type peptidase activity | 8/88 | 273/17632 | 6.57E-05 | 0.000451557 | 0.000265326 | F2/F7/DPP4/F10/PRSS3/PLAU/PLG/RBP3 | 8 |
| MF | GO:0046873 | metal ion transmembrane transporter activity | 10/88 | 444/17632 | 7.24E-05 | 0.000488706 | 0.000287154 | SLC6A2/TRPV1/SCN5A/SLC6A3/SLC6A4/KCNH2/GRIN1/GRIN2A/GRIN2B/GRIN2D | 10 |
| MF | GO:0000982 | transcription factor activity, RNA polymerase II proximal promoter sequence-specific DNA binding | 10/88 | 447/17632 | 7.66E-05 | 0.000506109 | 0.000297379 | ESR1/AR/PPARG/NR3C1/PGR/PPARA/PPARD/SP1/RELA/NFKB2 | 10 |
| MF | GO:0004935 | adrenergic receptor activity | 3/88 | 17/17632 | 7.77E-05 | 0.000506109 | 0.000297379 | ADRA1B/ADRB2/ADRA1D | 3 |
| MF | GO:0015296 | anion:cation symporter activity | 3/88 | 19/17632 | 0.000109864 | 0.000703877 | 0.000413584 | SLC6A2/SLC6A3/SLC6A4 | 3 |
| MF | GO:0001077 | transcriptional activator activity, RNA polymerase II proximal promoter sequence-specific DNA binding | 8/88 | 298/17632 | 0.000120779 | 0.000760907 | 0.000447094 | ESR1/AR/NR3C1/PGR/PPARA/SP1/RELA/NFKB2 | 8 |
| MF | GO:0005326 | neurotransmitter transporter activity | 4/88 | 54/17632 | 0.000151422 | 0.000938321 | 0.000551339 | SLC6A2/SLC6A3/SLC6A4/GABRQ | 4 |
| MF | GO:0008227 | G-protein coupled amine receptor activity | 4/88 | 59/17632 | 0.000213819 | 0.001303604 | 0.000765972 | CHRM2/ADRA1B/ADRB2/ADRA1D | 4 |
| MF | GO:0001540 | amyloid-beta binding | 4/88 | 61/17632 | 0.000243314 | 0.001459887 | 0.0008578 | ADRB2/GRIA2/ACHE/BCHE | 4 |
| MF | GO:0004252 | serine-type endopeptidase activity | 7/88 | 250/17632 | 0.000253259 | 0.001495812 | 0.000878909 | F2/F7/DPP4/F10/PRSS3/PLAU/PLG | 7 |
| MF | GO:0005328 | neurotransmitter:sodium symporter activity | 3/88 | 27/17632 | 0.000322197 | 0.00184531 | 0.001084267 | SLC6A2/SLC6A3/SLC6A4 | 3 |
| MF | GO:0033764 | steroid dehydrogenase activity, acting on the CH-OH group of donors, NAD or NADP as acceptor | 3/88 | 27/17632 | 0.000322197 | 0.00184531 | 0.001084267 | AKR1C1/HSD17B1/HSD11B1 | 3 |
| MF | GO:0030374 | ligand-dependent nuclear receptor transcription coactivator activity | 4/88 | 66/17632 | 0.000329722 | 0.001860224 | 0.001093031 | PPARG/PPARA/PPARD/VDR | 4 |
| MF | GO:0051059 | NF-kappaB binding | 3/88 | 28/17632 | 0.000359562 | 0.001998741 | 0.001174421 | GSK3B/PPARD/RELA | 3 |
| MF | GO:0005262 | calcium channel activity | 5/88 | 123/17632 | 0.000375716 | 0.00205827 | 0.001209398 | TRPV1/GRIN1/GRIN2A/GRIN2B/GRIN2D | 5 |
| MF | GO:0001228 | transcriptional activator activity, RNA polymerase II transcription regulatory region sequence-specific DNA binding | 9/88 | 449/17632 | 0.000400673 | 0.002163634 | 0.001271308 | ESR1/AR/NR3C1/PGR/PPARA/SP1/RELA/NFKB1/NFKB2 | 9 |
| MF | GO:0005244 | voltage-gated ion channel activity | 6/88 | 199/17632 | 0.000481875 | 0.002529842 | 0.001486485 | SCN5A/KCNH2/GRIN1/GRIN2A/GRIN2B/GRIN2D | 6 |
| MF | GO:0022832 | voltage-gated channel activity | 6/88 | 199/17632 | 0.000481875 | 0.002529842 | 0.001486485 | SCN5A/KCNH2/GRIN1/GRIN2A/GRIN2B/GRIN2D | 6 |
| MF | GO:0020037 | heme binding | 5/88 | 131/17632 | 0.0005016 | 0.002597325 | 0.001526136 | PTGS2/PTGS1/NOS2/MPO/PTGIS | 5 |
| MF | GO:0005504 | fatty acid binding | 3/88 | 33/17632 | 0.000588139 | 0.002964221 | 0.001741717 | PPARG/PPARA/PPARD | 3 |
| MF | GO:0016229 | steroid dehydrogenase activity | 3/88 | 33/17632 | 0.000588139 | 0.002964221 | 0.001741717 | AKR1C1/HSD17B1/HSD11B1 | 3 |
| MF | GO:0015085 | calcium ion transmembrane transporter activity | 5/88 | 139/17632 | 0.000656891 | 0.003267168 | 0.001919723 | TRPV1/GRIN1/GRIN2A/GRIN2B/GRIN2D | 5 |
| MF | GO:0005231 | excitatory extracellular ligand-gated ion channel activity | 3/88 | 35/17632 | 0.000700467 | 0.00339612 | 0.001995492 | CHRNA2/TRPV1/GRIA2 | 3 |
| MF | GO:0046906 | tetrapyrrole binding | 5/88 | 141/17632 | 0.000700787 | 0.00339612 | 0.001995492 | PTGS2/PTGS1/NOS2/MPO/PTGIS | 5 |
| MF | GO:0022843 | voltage-gated cation channel activity | 5/88 | 143/17632 | 0.000746843 | 0.0035735 | 0.002099718 | KCNH2/GRIN1/GRIN2A/GRIN2B/GRIN2D | 5 |
| MF | GO:0016209 | antioxidant activity | 4/88 | 86/17632 | 0.00090064 | 0.004255523 | 0.002500461 | PTGS2/PTGS1/SOD1/MPO | 4 |
| MF | GO:0042805 | actinin binding | 3/88 | 39/17632 | 0.000964109 | 0.004499173 | 0.002643624 | PPARG/RELA/NFKB1 | 3 |
| MF | GO:0016595 | glutamate binding | 2/88 | 10/17632 | 0.001079775 | 0.0049775 | 0.00292468 | GRIN1/GRIN2B | 2 |
| MF | GO:0005085 | guanyl-nucleotide exchange factor activity | 7/88 | 327/17632 | 0.001243515 | 0.005663237 | 0.003327605 | ERBB2/GRIN1/GRIN2A/GRIN2B/GRIN2D/P2RY12/PTGIR | 7 |
| MF | GO:0016705 | oxidoreductase activity, acting on paired donors, with incorporation or reduction of molecular oxygen | 5/88 | 161/17632 | 0.001269799 | 0.005714097 | 0.003357489 | PTGS2/PTGS1/NOS2/AKR1C1/PTGIS | 5 |
| MF | GO:1901618 | organic hydroxy compound transmembrane transporter activity | 3/88 | 44/17632 | 0.001372271 | 0.006102571 | 0.003585749 | SLC6A2/SLC6A3/SLC6A4 | 3 |
| MF | GO:0036041 | long-chain fatty acid binding | 2/88 | 13/17632 | 0.00185347 | 0.008146646 | 0.004786807 | PPARG/PPARD | 2 |
| MF | GO:0050661 | NADP binding | 3/88 | 50/17632 | 0.001987558 | 0.008635597 | 0.005074105 | NOS2/HMGCR/HSD17B1 | 3 |
| MF | GO:0004601 | peroxidase activity | 3/88 | 52/17632 | 0.002225049 | 0.009557599 | 0.005615854 | PTGS2/PTGS1/MPO | 3 |
| MF | GO:0016594 | glycine binding | 2/88 | 15/17632 | 0.002478922 | 0.010185137 | 0.005984583 | GRIN1/GRIN2B | 2 |
| MF | GO:0019841 | retinol binding | 2/88 | 15/17632 | 0.002478922 | 0.010185137 | 0.005984583 | RBP3/RLBP1 | 2 |
| MF | GO:0046965 | retinoid X receptor binding | 2/88 | 15/17632 | 0.002478922 | 0.010185137 | 0.005984583 | PPARG/VDR | 2 |
| MF | GO:0050998 | nitric-oxide synthase binding | 2/88 | 15/17632 | 0.002478922 | 0.010185137 | 0.005984583 | SCN5A/SLC6A4 | 2 |
| MF | GO:0043621 | protein self-association | 3/88 | 55/17632 | 0.002613102 | 0.010620995 | 0.006240685 | PPARG/ACHE/SLC2A1 | 3 |
| MF | GO:0048018 | receptor ligand activity | 8/88 | 478/17632 | 0.0026562 | 0.010681315 | 0.006276128 | F2/BDNF/INS/CCK/PYY/IL10/IL6/TNF | 8 |
| MF | GO:0016684 | oxidoreductase activity, acting on peroxide as acceptor | 3/88 | 56/17632 | 0.002751134 | 0.010946619 | 0.006432015 | PTGS2/PTGS1/MPO | 3 |
| MF | GO:0016918 | retinal binding | 2/88 | 16/17632 | 0.002823885 | 0.011004417 | 0.006465976 | RBP3/RLBP1 | 2 |
| MF | GO:0034185 | apolipoprotein binding | 2/88 | 16/17632 | 0.002823885 | 0.011004417 | 0.006465976 | LPL/PLG | 2 |
| MF | GO:0097110 | scaffold protein binding | 3/88 | 58/17632 | 0.003040505 | 0.011727663 | 0.006890941 | HSP90AA1/SCN5A/KCNH2 | 3 |
| MF | GO:0031625 | ubiquitin protein ligase binding | 6/88 | 286/17632 | 0.003081268 | 0.011764842 | 0.006912787 | HSP90AA1/SCN5A/KCNH2/GSK3B/BCL2/RELA | 6 |
| MF | GO:0016616 | oxidoreductase activity, acting on the CH-OH group of donors, NAD or NADP as acceptor | 4/88 | 121/17632 | 0.00315726 | 0.011828935 | 0.006950447 | HMGCR/AKR1C1/HSD17B1/HSD11B1 | 4 |
| MF | GO:0042166 | acetylcholine binding | 2/88 | 17/17632 | 0.003190048 | 0.011828935 | 0.006950447 | CHRNA2/ACHE | 2 |
| MF | GO:0016597 | amino acid binding | 3/88 | 59/17632 | 0.003191935 | 0.011828935 | 0.006950447 | NOS2/GRIN1/GRIN2B | 3 |
| MF | GO:0048037 | cofactor binding | 8/88 | 495/17632 | 0.003287561 | 0.012065028 | 0.00708917 | PTGS2/PTGS1/MAOB/NOS2/HMGCR/MPO/HSD17B1/PTGIS | 8 |
| MF | GO:0044389 | ubiquitin-like protein ligase binding | 6/88 | 301/17632 | 0.003957297 | 0.014383253 | 0.008451313 | HSP90AA1/SCN5A/KCNH2/GSK3B/BCL2/RELA | 6 |
| MF | GO:0016614 | oxidoreductase activity, acting on CH-OH group of donors | 4/88 | 132/17632 | 0.004308439 | 0.015364057 | 0.009027614 | HMGCR/AKR1C1/HSD17B1/HSD11B1 | 4 |
| MF | GO:0070851 | growth factor receptor binding | 4/88 | 132/17632 | 0.004308439 | 0.015364057 | 0.009027614 | IL10/IL6/APP/ITGB3 | 4 |
| MF | GO:0001091 | RNA polymerase II basal transcription factor binding | 2/88 | 20/17632 | 0.004413601 | 0.015447603 | 0.009076704 | ESR1/AR | 2 |
| MF | GO:1901338 | catecholamine binding | 2/88 | 20/17632 | 0.004413601 | 0.015447603 | 0.009076704 | SLC6A3/ADRB2 | 2 |
| MF | GO:0001223 | transcription coactivator binding | 2/88 | 21/17632 | 0.004862432 | 0.016862378 | 0.009907997 | ESR1/PPARA | 2 |
| MF | GO:0052689 | carboxylic ester hydrolase activity | 4/88 | 139/17632 | 0.005172157 | 0.017773413 | 0.010443303 | LPL/PON1/ACHE/BCHE | 4 |
| MF | GO:0003713 | transcription coactivator activity | 6/88 | 321/17632 | 0.005397433 | 0.018380448 | 0.010799985 | PPARG/ESR2/PPARA/PPARD/VDR/NFKB2 | 6 |
| MF | GO:0015293 | symporter activity | 4/88 | 142/17632 | 0.005575403 | 0.018698526 | 0.010986881 | SLC6A2/SLC6A3/SLC6A4/SLC22A5 | 4 |
| MF | GO:0015370 | solute:sodium symporter activity | 3/88 | 72/17632 | 0.005589771 | 0.018698526 | 0.010986881 | SLC6A2/SLC6A3/SLC6A4 | 3 |
| MF | GO:0015464 | acetylcholine receptor activity | 2/88 | 23/17632 | 0.005820306 | 0.019131093 | 0.011241049 | CHRM2/CHRNA2 | 2 |
| MF | GO:0017134 | fibroblast growth factor binding | 2/88 | 23/17632 | 0.005820306 | 0.019131093 | 0.011241049 | SCN5A/ITGB3 | 2 |
| MF | GO:0042974 | retinoic acid receptor binding | 2/88 | 24/17632 | 0.006328936 | 0.020623602 | 0.012118017 | PPARG/VDR | 2 |
| MF | GO:0015081 | sodium ion transmembrane transporter activity | 4/88 | 150/17632 | 0.006752215 | 0.021814849 | 0.01281797 | SLC6A2/SCN5A/SLC6A3/SLC6A4 | 4 |
| MF | GO:0043178 | alcohol binding | 3/88 | 79/17632 | 0.007228819 | 0.023156724 | 0.013606429 | CETP/RBP3/RLBP1 | 3 |
| MF | GO:0051117 | ATPase binding | 3/88 | 80/17632 | 0.007483727 | 0.023771838 | 0.013967858 | ESR1/AR/PGR | 3 |
| MF | GO:0016702 | oxidoreductase activity, acting on single donors with incorporation of molecular oxygen, incorporation of two atoms of oxygen | 2/88 | 27/17632 | 0.007971149 | 0.02502103 | 0.014701859 | PTGS2/ALOX5 | 2 |
| MF | GO:0008013 | beta-catenin binding | 3/88 | 82/17632 | 0.008009377 | 0.02502103 | 0.014701859 | ESR1/AR/GSK3B | 3 |
| MF | GO:0005184 | neuropeptide hormone activity | 2/88 | 28/17632 | 0.008556655 | 0.026296063 | 0.015451042 | CCK/PYY | 2 |
| MF | GO:0016701 | oxidoreductase activity, acting on single donors with incorporation of molecular oxygen | 2/88 | 28/17632 | 0.008556655 | 0.026296063 | 0.015451042 | PTGS2/ALOX5 | 2 |
| MF | GO:0005088 | Ras guanyl-nucleotide exchange factor activity | 5/88 | 254/17632 | 0.00886049 | 0.027010202 | 0.015870656 | ERBB2/GRIN1/GRIN2A/GRIN2B/GRIN2D | 5 |
| MF | GO:0008201 | heparin binding | 4/88 | 163/17632 | 0.008996745 | 0.027206157 | 0.015985796 | F2/LPL/MPO/APP | 4 |
| MF | GO:0008083 | growth factor activity | 4/88 | 166/17632 | 0.00957601 | 0.028728029 | 0.016880017 | F2/BDNF/IL10/IL6 | 4 |
| MF | GO:0001078 | transcriptional repressor activity, RNA polymerase II proximal promoter sequence-specific DNA binding | 4/88 | 169/17632 | 0.010179065 | 0.030296744 | 0.017801763 | PPARG/PPARA/PPARD/RELA | 4 |
| MF | GO:0001227 | transcriptional repressor activity, RNA polymerase II transcription regulatory region sequence-specific DNA binding | 5/88 | 267/17632 | 0.010837788 | 0.032005343 | 0.018805702 | PPARG/PPARA/PPARD/RELA/NFKB1 | 5 |
| MF | GO:0051213 | dioxygenase activity | 3/88 | 92/17632 | 0.01096025 | 0.032116081 | 0.018870769 | PTGS2/PTGS1/ALOX5 | 3 |
| MF | GO:0004497 | monooxygenase activity | 3/88 | 98/17632 | 0.01299444 | 0.037783834 | 0.022201027 | NOS2/AKR1C1/PTGIS | 3 |
| MF | GO:0005501 | retinoid binding | 2/88 | 35/17632 | 0.013168406 | 0.037997384 | 0.022326505 | RBP3/RLBP1 | 2 |
| MF | GO:0005126 | cytokine receptor binding | 5/88 | 281/17632 | 0.01328455 | 0.03804212 | 0.022352791 | IL10/IL6/ITGB3/CASP3/TNF | 5 |
| MF | GO:0051427 | hormone receptor binding | 4/88 | 185/17632 | 0.01381153 | 0.039253821 | 0.023064762 | ESR1/PPARG/CCK/VDR | 4 |
| MF | GO:0042277 | peptide binding | 5/88 | 285/17632 | 0.014046963 | 0.039443706 | 0.023176335 | ADRB2/PPARG/GRIA2/ACHE/BCHE | 5 |
| MF | GO:0015238 | drug transmembrane transporter activity | 3/88 | 101/17632 | 0.014087038 | 0.039443706 | 0.023176335 | SLC6A2/SLC6A3/SLC22A5 | 3 |
| MF | GO:0019840 | isoprenoid binding | 2/88 | 37/17632 | 0.014645236 | 0.040705142 | 0.02391753 | RBP3/RLBP1 | 2 |
| MF | GO:0015294 | solute:cation symporter activity | 3/88 | 103/17632 | 0.014843626 | 0.040955406 | 0.02406458 | SLC6A2/SLC6A3/SLC6A4 | 3 |
| MF | GO:0001205 | transcriptional activator activity, RNA polymerase II distal enhancer sequence-specific DNA binding | 2/88 | 38/17632 | 0.015409241 | 0.042207921 | 0.024800533 | RELA/NFKB1 | 2 |
| MF | GO:0005516 | calmodulin binding | 4/88 | 192/17632 | 0.015629406 | 0.042502989 | 0.024973909 | TRPV1/SCN5A/NOS2/GRIN1 | 4 |
| MF | GO:0016504 | peptidase activator activity | 2/88 | 39/17632 | 0.016190059 | 0.043403137 | 0.025502818 | APP/CASP3 | 2 |
| MF | GO:0016709 | oxidoreductase activity, acting on paired donors, with incorporation or reduction of molecular oxygen, NAD(P)H as one donor, and incorporation of one atom of oxygen | 2/88 | 39/17632 | 0.016190059 | 0.043403137 | 0.025502818 | NOS2/AKR1C1 | 2 |
| MF | GO:0030331 | estrogen receptor binding | 2/88 | 40/17632 | 0.016987507 | 0.045220266 | 0.026570527 | ESR1/PPARG | 2 |
| MF | GO:0001221 | transcription cofactor binding | 2/88 | 41/17632 | 0.017801405 | 0.047055462 | 0.027648851 | ESR1/PPARA | 2 |
| MF | GO:0042826 | histone deacetylase binding | 3/88 | 112/17632 | 0.018529504 | 0.048639948 | 0.028579864 | HSP90AA1/SP1/RELA | 3 |
