# Supplementary material for: Exploring the Potential Mechanism of Chuanxiong Rhizoma Treatment for Migraine Based on Systems Pharmacology
Source: Evid Based Complement Alternat Med. 2021 Dec 28;2021:2809004. doi: 10.1155/2021/2809004 (PMC8727101; doi:10.1155/2021/2809004)
Supplement: Supplementary Materials — Supplementary Table S1: active compounds of CR). A total of 248 candidate compounds were included. Supplementary Table S2: relevant targets of CR active compounds. Supplementary Table S3: targets for Migraine. Supplementary Table S4: overlapping targets between disease and drug. Supplementary Table S5: details of the active compounds of CR and overlapping targets. Supplementary Table S6: the details of the PPI network. Supplementary Table S7: details of the GO enrichment analysis. Supplementary Table S8: details of the KEGG pathway enrichment analysis. [file 2809004.f1.zip › 2809004.f1/Supplementary Table S8 Details of the KEGG pathway enrichment analysis (1).doc]

| ID | Description | GeneRatio | BgRatio | pvalue | p.adjust | qvalue | geneID | Count |
| --- | --- | --- | --- | --- | --- | --- | --- | --- |
| hsa05033 | Nicotine addiction | 15/81 | 40/8108 | 8.32E-21 | 1.82E-18 | 8.85E-19 | GRIA2/GABRA1/GABRA3/GABRA4/GABRA5/GABRB3/GABRD/GABRE/GABRG2/GABRP/GABRQ/GRIN1/GRIN2A/GRIN2B/GRIN2D | 15 |
| hsa04080 | Neuroactive ligand-receptor interaction | 28/81 | 341/8108 | 5.78E-19 | 6.33E-17 | 3.07E-17 | CHRM2/CHRNA2/TRPV1/ADRA1B/ADRB2/F2/GRIA2/NR3C1/ADRA1D/PRSS3/PLG/CCK/PYY/GABRA1/GABRA3/GABRA4/GABRA5/GABRB3/GABRD/GABRE/GABRG2/GABRP/GABRQ/GRIN1/GRIN2A/GRIN2B/GRIN2D/PTGIR | 28 |
| hsa05030 | Cocaine addiction | 10/81 | 49/8108 | 3.36E-11 | 2.45E-09 | 1.19E-09 | SLC6A3/MAOB/GRIA2/BDNF/RELA/GRIN1/GRIN2A/GRIN2B/GRIN2D/NFKB1 | 10 |
| hsa04727 | GABAergic synapse | 10/81 | 89/8108 | 1.51E-08 | 7.66E-07 | 3.72E-07 | GABRA1/GABRA3/GABRA4/GABRA5/GABRB3/GABRD/GABRE/GABRG2/GABRP/GABRQ | 10 |
| hsa05032 | Morphine addiction | 10/81 | 91/8108 | 1.87E-08 | 7.66E-07 | 3.72E-07 | GABRA1/GABRA3/GABRA4/GABRA5/GABRB3/GABRD/GABRE/GABRG2/GABRP/GABRQ | 10 |
| hsa04723 | Retrograde endocannabinoid signaling | 12/81 | 148/8108 | 2.10E-08 | 7.66E-07 | 3.72E-07 | PTGS2/GRIA2/GABRA1/GABRA3/GABRA4/GABRA5/GABRB3/GABRD/GABRE/GABRG2/GABRP/GABRQ | 12 |
| hsa05215 | Prostate cancer | 10/81 | 97/8108 | 3.50E-08 | 1.09E-06 | 5.31E-07 | HSP90AA1/AR/GSK3B/PLAU/ERBB2/INS/BCL2/RELA/AKT1/NFKB1 | 10 |
| hsa04066 | HIF-1 signaling pathway | 10/81 | 109/8108 | 1.08E-07 | 2.95E-06 | 1.43E-06 | NOS2/ERBB2/SERPINE1/INS/BCL2/RELA/IL6/AKT1/SLC2A1/NFKB1 | 10 |
| hsa05010 | Alzheimer disease | 16/81 | 369/8108 | 5.47E-07 | 1.33E-05 | 6.46E-06 | PTGS2/NOS2/GSK3B/LPL/INS/RELA/IL6/GRIN1/GRIN2A/GRIN2B/GRIN2D/AKT1/APP/CASP3/NFKB1/TNF | 16 |
| hsa05022 | Pathways of neurodegeneration - multiple diseases | 18/81 | 476/8108 | 7.20E-07 | 1.58E-05 | 7.66E-06 | PTGS2/SLC6A3/NOS2/GSK3B/GRIA2/SOD1/BDNF/BCL2/RELA/IL6/GRIN1/GRIN2A/GRIN2B/GRIN2D/APP/CASP3/NFKB1/TNF | 18 |
| hsa05207 | Chemical carcinogenesis - receptor activation | 12/81 | 212/8108 | 1.08E-06 | 2.02E-05 | 9.81E-06 | HSP90AA1/ADRB2/ESR1/AR/ESR2/PGR/PPARA/BCL2/RELA/VDR/AKT1/NFKB1 | 12 |
| hsa04931 | Insulin resistance | 9/81 | 108/8108 | 1.11E-06 | 2.02E-05 | 9.81E-06 | GSK3B/PPARA/INS/RELA/IL6/AKT1/SLC2A1/NFKB1/TNF | 9 |
| hsa05145 | Toxoplasmosis | 9/81 | 112/8108 | 1.51E-06 | 2.39E-05 | 1.16E-05 | NOS2/BCL2/IL10/RELA/AKT1/ALOX5/CASP3/NFKB1/TNF | 9 |
| hsa04024 | cAMP signaling pathway | 12/81 | 219/8108 | 1.53E-06 | 2.39E-05 | 1.16E-05 | CHRM2/ADRB2/GRIA2/BDNF/PPARA/RELA/GRIN1/GRIN2A/GRIN2B/GRIN2D/AKT1/NFKB1 | 12 |
| hsa04932 | Non-alcoholic fatty liver disease | 10/81 | 155/8108 | 2.86E-06 | 4.17E-05 | 2.03E-05 | PPARG/GSK3B/PPARA/INS/RELA/IL6/AKT1/CASP3/NFKB1/TNF | 10 |
| hsa04657 | IL-17 signaling pathway | 8/81 | 94/8108 | 3.88E-06 | 5.31E-05 | 2.58E-05 | PTGS2/HSP90AA1/GSK3B/RELA/IL6/CASP3/NFKB1/TNF | 8 |
| hsa05031 | Amphetamine addiction | 7/81 | 69/8108 | 5.01E-06 | 6.45E-05 | 3.13E-05 | SLC6A3/MAOB/GRIA2/GRIN1/GRIN2A/GRIN2B/GRIN2D | 7 |
| hsa04917 | Prolactin signaling pathway | 7/81 | 70/8108 | 5.52E-06 | 6.71E-05 | 3.26E-05 | ESR1/ESR2/GSK3B/INS/RELA/AKT1/NFKB1 | 7 |
| hsa04933 | AGE-RAGE signaling pathway in diabetic complications | 8/81 | 100/8108 | 6.19E-06 | 7.13E-05 | 3.46E-05 | SERPINE1/BCL2/RELA/IL6/AKT1/CASP3/NFKB1/TNF | 8 |
| hsa05142 | Chagas disease | 8/81 | 102/8108 | 7.17E-06 | 7.85E-05 | 3.81E-05 | NOS2/SERPINE1/IL10/RELA/IL6/AKT1/NFKB1/TNF | 8 |
| hsa04625 | C-type lectin receptor signaling pathway | 8/81 | 104/8108 | 8.29E-06 | 8.65E-05 | 4.20E-05 | PTGS2/IL10/RELA/IL6/AKT1/NFKB1/NFKB2/TNF | 8 |
| hsa05133 | Pertussis | 7/81 | 76/8108 | 9.59E-06 | 9.55E-05 | 4.63E-05 | NOS2/IL10/RELA/IL6/CASP3/NFKB1/TNF | 7 |
| hsa05152 | Tuberculosis | 10/81 | 180/8108 | 1.09E-05 | 0.00010354 | 5.03E-05 | NOS2/BCL2/IL10/RELA/IL6/VDR/AKT1/CASP3/NFKB1/TNF | 10 |
| hsa04726 | Serotonergic synapse | 8/81 | 115/8108 | 1.74E-05 | 0.000159008 | 7.72E-05 | PTGS2/PTGS1/SLC6A4/MAOB/GABRB3/ALOX5/APP/CASP3 | 8 |
| hsa05134 | Legionellosis | 6/81 | 57/8108 | 1.99E-05 | 0.000174595 | 8.48E-05 | RELA/IL6/CASP3/NFKB1/NFKB2/TNF | 6 |
| hsa05222 | Small cell lung cancer | 7/81 | 92/8108 | 3.38E-05 | 0.000284749 | 0.000138235 | PTGS2/NOS2/BCL2/RELA/AKT1/CASP3/NFKB1 | 7 |
| hsa05417 | Lipid and atherosclerosis | 10/81 | 215/8108 | 5.06E-05 | 0.000410137 | 0.000199105 | HSP90AA1/PPARG/GSK3B/BCL2/RELA/IL6/AKT1/CASP3/NFKB1/TNF | 10 |
| hsa04920 | Adipocytokine signaling pathway | 6/81 | 69/8108 | 5.99E-05 | 0.000468199 | 0.000227292 | PPARA/RELA/AKT1/SLC2A1/NFKB1/TNF | 6 |
| hsa05146 | Amoebiasis | 7/81 | 102/8108 | 6.58E-05 | 0.000496753 | 0.000241154 | NOS2/IL10/RELA/IL6/CASP3/NFKB1/TNF | 7 |
| hsa05163 | Human cytomegalovirus infection | 10/81 | 225/8108 | 7.42E-05 | 0.000526083 | 0.000255392 | PTGS2/GSK3B/SP1/RELA/IL6/AKT1/ITGB3/CASP3/NFKB1/TNF | 10 |
| hsa04064 | NF-kappa B signaling pathway | 7/81 | 104/8108 | 7.45E-05 | 0.000526083 | 0.000255392 | PTGS2/PLAU/BCL2/RELA/NFKB1/NFKB2/TNF | 7 |
| hsa05224 | Breast cancer | 8/81 | 147/8108 | 0.000101467 | 0.000694418 | 0.000337112 | ESR1/ESR2/GSK3B/PGR/ERBB2/SP1/AKT1/NFKB2 | 8 |
| hsa05140 | Leishmaniasis | 6/81 | 77/8108 | 0.000111116 | 0.000737409 | 0.000357983 | PTGS2/NOS2/IL10/RELA/NFKB1/TNF | 6 |
| hsa04668 | TNF signaling pathway | 7/81 | 112/8108 | 0.000119123 | 0.000767293 | 0.00037249 | PTGS2/RELA/IL6/AKT1/CASP3/NFKB1/TNF | 7 |
| hsa04151 | PI3K-Akt signaling pathway | 12/81 | 354/8108 | 0.000184953 | 0.001157279 | 0.000561813 | CHRM2/HSP90AA1/GSK3B/ERBB2/BDNF/INS/BCL2/RELA/IL6/AKT1/ITGB3/NFKB1 | 12 |
| hsa04610 | Complement and coagulation cascades | 6/81 | 85/8108 | 0.00019238 | 0.001170313 | 0.000568141 | F2/F7/F10/PLAU/SERPINE1/PLG | 6 |
| hsa01523 | Antifolate resistance | 4/81 | 31/8108 | 0.000236907 | 0.001402236 | 0.00068073 | RELA/IL6/NFKB1/TNF | 4 |
| hsa04923 | Regulation of lipolysis in adipocytes | 5/81 | 57/8108 | 0.000244929 | 0.001411565 | 0.000685258 | PTGS2/PTGS1/ADRB2/INS/AKT1 | 5 |
| hsa04380 | Osteoclast differentiation | 7/81 | 128/8108 | 0.000273491 | 0.001535755 | 0.000745548 | PPARG/RELA/AKT1/ITGB3/NFKB1/NFKB2/TNF | 7 |
| hsa05164 | Influenza A | 8/81 | 172/8108 | 0.000300024 | 0.00164263 | 0.000797431 | PRSS3/PLG/RELA/IL6/AKT1/CASP3/NFKB1/TNF | 8 |
| hsa04728 | Dopaminergic synapse | 7/81 | 132/8108 | 0.000330248 | 0.001764009 | 0.000856356 | SLC6A3/MAOB/GSK3B/GRIA2/GRIN2A/GRIN2B/AKT1 | 7 |
| hsa05135 | Yersinia infection | 7/81 | 137/8108 | 0.000414117 | 0.002115365 | 0.001026926 | GSK3B/IL10/RELA/IL6/AKT1/NFKB1/TNF | 7 |
| hsa01522 | Endocrine resistance | 6/81 | 98/8108 | 0.000417737 | 0.002115365 | 0.001026926 | ESR1/ESR2/ERBB2/BCL2/SP1/AKT1 | 6 |
| hsa04915 | Estrogen signaling pathway | 7/81 | 138/8108 | 0.000432774 | 0.002115365 | 0.001026926 | HSP90AA1/ESR1/ESR2/PGR/BCL2/SP1/AKT1 | 7 |
| hsa05162 | Measles | 7/81 | 139/8108 | 0.000452098 | 0.002115365 | 0.001026926 | GSK3B/BCL2/RELA/IL6/AKT1/CASP3/NFKB1 | 7 |
| hsa05418 | Fluid shear stress and atherosclerosis | 7/81 | 139/8108 | 0.000452098 | 0.002115365 | 0.001026926 | HSP90AA1/BCL2/RELA/AKT1/ITGB3/NFKB1/TNF | 7 |
| hsa05321 | Inflammatory bowel disease | 5/81 | 65/8108 | 0.000453982 | 0.002115365 | 0.001026926 | IL10/RELA/IL6/NFKB1/TNF | 5 |
| hsa04720 | Long-term potentiation | 5/81 | 67/8108 | 0.000522608 | 0.002335737 | 0.001133908 | GRIA2/GRIN1/GRIN2A/GRIN2B/GRIN2D | 5 |
| hsa05221 | Acute myeloid leukemia | 5/81 | 67/8108 | 0.000522608 | 0.002335737 | 0.001133908 | MPO/PPARD/RELA/AKT1/NFKB1 | 5 |
| hsa05017 | Spinocerebellar ataxia | 7/81 | 143/8108 | 0.000536413 | 0.002349488 | 0.001140583 | GRIA2/SP1/GRIN1/GRIN2A/GRIN2B/GRIN2D/AKT1 | 7 |
| hsa04660 | T cell receptor signaling pathway | 6/81 | 104/8108 | 0.00057427 | 0.002465982 | 0.001197136 | GSK3B/IL10/RELA/AKT1/NFKB1/TNF | 6 |
| hsa04020 | Calcium signaling pathway | 9/81 | 240/8108 | 0.000611452 | 0.002575153 | 0.001250135 | CHRM2/ADRA1B/ADRB2/NOS2/ADRA1D/ERBB2/GRIN1/GRIN2A/GRIN2D | 9 |
| hsa05169 | Epstein-Barr virus infection | 8/81 | 202/8108 | 0.000874708 | 0.00361436 | 0.001754628 | BCL2/RELA/IL6/AKT1/CASP3/NFKB1/NFKB2/TNF | 8 |
| hsa05415 | Diabetic cardiomyopathy | 8/81 | 203/8108 | 0.000903311 | 0.003663427 | 0.001778448 | GSK3B/PPARA/INS/SP1/RELA/AKT1/SLC2A1/NFKB1 | 8 |
| hsa05160 | Hepatitis C | 7/81 | 157/8108 | 0.000934892 | 0.00372257 | 0.00180716 | GSK3B/PPARA/RELA/AKT1/CASP3/NFKB1/TNF | 7 |
| hsa01521 | EGFR tyrosine kinase inhibitor resistance | 5/81 | 79/8108 | 0.001110944 | 0.004316731 | 0.002095601 | GSK3B/ERBB2/BCL2/IL6/AKT1 | 5 |
| hsa05161 | Hepatitis B | 7/81 | 162/8108 | 0.001123533 | 0.004316731 | 0.002095601 | BCL2/RELA/IL6/AKT1/CASP3/NFKB1/TNF | 7 |
| hsa04722 | Neurotrophin signaling pathway | 6/81 | 119/8108 | 0.001166509 | 0.004404578 | 0.002138247 | GSK3B/BDNF/BCL2/RELA/AKT1/NFKB1 | 6 |
| hsa04611 | Platelet activation | 6/81 | 124/8108 | 0.001443016 | 0.005356279 | 0.00260026 | PTGS1/F2/AKT1/ITGB3/P2RY12/PTGIR | 6 |
| hsa05020 | Prion disease | 9/81 | 273/8108 | 0.001522268 | 0.005556279 | 0.002697353 | GSK3B/SOD1/IL6/GRIN1/GRIN2A/GRIN2B/GRIN2D/CASP3/TNF | 9 |
| hsa04913 | Ovarian steroidogenesis | 4/81 | 51/8108 | 0.001617327 | 0.005806469 | 0.00281881 | PTGS2/INS/HSD17B1/ALOX5 | 4 |
| hsa04211 | Longevity regulating pathway | 5/81 | 89/8108 | 0.001893245 | 0.00668743 | 0.003246481 | PPARG/INS/RELA/AKT1/NFKB1 | 5 |
| hsa04014 | Ras signaling pathway | 8/81 | 232/8108 | 0.002120077 | 0.00736979 | 0.00357774 | BDNF/INS/RELA/GRIN1/GRIN2A/GRIN2B/AKT1/NFKB1 | 8 |
| hsa04210 | Apoptosis | 6/81 | 136/8108 | 0.002310154 | 0.007905059 | 0.003837592 | BCL2/RELA/AKT1/CASP3/NFKB1/TNF | 6 |
| hsa04010 | MAPK signaling pathway | 9/81 | 294/8108 | 0.002528476 | 0.00849065 | 0.004121873 | ERBB2/BDNF/INS/RELA/AKT1/CASP3/NFKB1/NFKB2/TNF | 9 |
| hsa05034 | Alcoholism | 7/81 | 187/8108 | 0.002558826 | 0.00849065 | 0.004121873 | SLC6A3/MAOB/BDNF/GRIN1/GRIN2A/GRIN2B/GRIN2D | 7 |
| hsa04713 | Circadian entrainment | 5/81 | 97/8108 | 0.002762794 | 0.009030626 | 0.00438401 | GRIA2/GRIN1/GRIN2A/GRIN2B/GRIN2D | 5 |
| hsa05202 | Transcriptional misregulation in cancer | 7/81 | 192/8108 | 0.002966641 | 0.009554329 | 0.004638247 | PPARG/PLAU/MPO/SP1/RELA/IL6/NFKB1 | 7 |
| hsa00590 | Arachidonic acid metabolism | 4/81 | 61/8108 | 0.003132164 | 0.009833317 | 0.004773684 | PTGS2/PTGS1/ALOX5/PTGIS | 4 |
| hsa05167 | Kaposi sarcoma-associated herpesvirus infection | 7/81 | 194/8108 | 0.003143069 | 0.009833317 | 0.004773684 | PTGS2/GSK3B/RELA/IL6/AKT1/CASP3/NFKB1 | 7 |
| hsa05014 | Amyotrophic lateral sclerosis | 10/81 | 365/8108 | 0.00327304 | 0.009990811 | 0.004850141 | NOS2/GRIA2/SOD1/BCL2/GRIN1/GRIN2A/GRIN2B/GRIN2D/CASP3/TNF | 10 |
| hsa05132 | Salmonella infection | 8/81 | 249/8108 | 0.00328465 | 0.009990811 | 0.004850141 | HSP90AA1/BCL2/RELA/IL6/AKT1/CASP3/NFKB1/TNF | 8 |
| hsa04620 | Toll-like receptor signaling pathway | 5/81 | 104/8108 | 0.003734498 | 0.011139572 | 0.005407819 | RELA/IL6/AKT1/NFKB1/TNF | 5 |
| hsa04261 | Adrenergic signaling in cardiomyocytes | 6/81 | 150/8108 | 0.003764056 | 0.011139572 | 0.005407819 | SCN5A/ADRA1B/ADRB2/ADRA1D/BCL2/AKT1 | 6 |
| hsa05205 | Proteoglycans in cancer | 7/81 | 205/8108 | 0.004261673 | 0.012444084 | 0.006041108 | ESR1/PLAU/ERBB2/AKT1/ITGB3/CASP3/TNF | 7 |
| hsa04724 | Glutamatergic synapse | 5/81 | 114/8108 | 0.005518111 | 0.015900872 | 0.007719241 | GRIA2/GRIN1/GRIN2A/GRIN2B/GRIN2D | 5 |
| hsa05143 | African trypanosomiasis | 3/81 | 37/8108 | 0.00584496 | 0.016623978 | 0.00807028 | IL10/IL6/TNF | 3 |
| hsa01524 | Platinum drug resistance | 4/81 | 73/8108 | 0.005969298 | 0.016759953 | 0.00813629 | ERBB2/BCL2/AKT1/CASP3 | 4 |
| hsa05166 | Human T-cell leukemia virus 1 infection | 7/81 | 219/8108 | 0.006095014 | 0.016896305 | 0.008202484 | RELA/IL6/AKT1/SLC2A1/NFKB1/NFKB2/TNF | 7 |
| hsa04071 | Sphingolipid signaling pathway | 5/81 | 119/8108 | 0.006605154 | 0.018081609 | 0.008777902 | BCL2/RELA/AKT1/NFKB1/TNF | 5 |
| hsa03320 | PPAR signaling pathway | 4/81 | 76/8108 | 0.00687967 | 0.018373754 | 0.008919727 | PPARG/LPL/PPARA/PPARD | 4 |
| hsa05212 | Pancreatic cancer | 4/81 | 76/8108 | 0.00687967 | 0.018373754 | 0.008919727 | ERBB2/RELA/AKT1/NFKB1 | 4 |
| hsa04919 | Thyroid hormone signaling pathway | 5/81 | 121/8108 | 0.007079352 | 0.018679253 | 0.009068035 | ESR1/GSK3B/AKT1/ITGB3/SLC2A1 | 5 |
| hsa04662 | B cell receptor signaling pathway | 4/81 | 82/8108 | 0.008966676 | 0.023377405 | 0.0113488 | GSK3B/RELA/AKT1/NFKB1 | 4 |
| hsa04621 | NOD-like receptor signaling pathway | 6/81 | 185/8108 | 0.010260361 | 0.026435518 | 0.012833392 | HSP90AA1/BCL2/RELA/IL6/NFKB1/TNF | 6 |
| hsa04742 | Taste transduction | 4/81 | 86/8108 | 0.010565576 | 0.026596104 | 0.012911351 | GABRA1/GABRA3/GABRA4/GABRA5 | 4 |
| hsa05210 | Colorectal cancer | 4/81 | 86/8108 | 0.010565576 | 0.026596104 | 0.012911351 | GSK3B/BCL2/AKT1/CASP3 | 4 |
| hsa05016 | Huntington disease | 8/81 | 306/8108 | 0.011059773 | 0.027523754 | 0.013361688 | PPARG/GRIA2/SOD1/BDNF/SP1/GRIN1/GRIN2B/CASP3 | 8 |
| hsa05144 | Malaria | 3/81 | 50/8108 | 0.013439565 | 0.033070391 | 0.01605436 | IL10/IL6/TNF | 3 |
| hsa05130 | Pathogenic Escherichia coli infection | 6/81 | 197/8108 | 0.013686972 | 0.033304966 | 0.016168236 | F2/RELA/IL6/CASP3/NFKB1/TNF | 6 |
| hsa05165 | Human papillomavirus infection | 8/81 | 331/8108 | 0.017097084 | 0.04114573 | 0.019974615 | PTGS2/GSK3B/RELA/AKT1/ITGB3/CASP3/NFKB1/TNF | 8 |
| hsa04914 | Progesterone-mediated oocyte maturation | 4/81 | 100/8108 | 0.017574276 | 0.041834417 | 0.020308945 | HSP90AA1/PGR/INS/AKT1 | 4 |
| hsa04015 | Rap1 signaling pathway | 6/81 | 210/8108 | 0.018229512 | 0.04292756 | 0.020839623 | INS/GRIN1/GRIN2A/GRIN2B/AKT1/ITGB3 | 6 |
| hsa05170 | Human immunodeficiency virus 1 infection | 6/81 | 212/8108 | 0.019010314 | 0.044289987 | 0.021501027 | BCL2/RELA/AKT1/CASP3/NFKB1/TNF | 6 |
| hsa04218 | Cellular senescence | 5/81 | 156/8108 | 0.019637099 | 0.045268682 | 0.021976145 | SERPINE1/RELA/IL6/AKT1/NFKB1 | 5 |
| hsa05213 | Endometrial cancer | 3/81 | 58/8108 | 0.019990578 | 0.045603507 | 0.022138689 | GSK3B/ERBB2/AKT1 | 3 |
| hsa04659 | Th17 cell differentiation | 4/81 | 107/8108 | 0.021955275 | 0.049569125 | 0.024063839 | HSP90AA1/RELA/IL6/NFKB1 | 4 |
